# Supplementary material for: Single-cell trajectories reconstruction, exploration and mapping of omics data with STREAM
Source: Nat Commun. 2019 Apr 23;10:1903. doi: 10.1038/s41467-019-09670-4 (PMC6478907; doi:10.1038/s41467-019-09670-4)
Supplement: Supplementary file 1 — Supplementary Information [file 41467_2019_9670_MOESM1_ESM.pdf]

Supplementary Information

Single-cell Trajectories Reconstruction Exploration And Mapping of omics data with STREAM

Chen et al.

Supplementary Note 1: STREAM analysis on scRNA-seq from the mouse hematopoietic system

We used STREAM to reanalyze scRNA-seq data from Nestorowa et al. 2016[1], which sorted and profiled 1656 single cells including hematopoietic stem cells (HSCs), multipotent progenitors (MPPs), lymphoid multipotent progenitors (LMPPs), common myeloid progenitors (CMPs), granulocyte-monocyte progenitors (GMPs) and megakaryocyte-erythrocyte progenitors (MEPs), to study the mouse hematopoietic stem and progenitor cell differentiation processes.

STREAM recovers two bifurcation events (**Fig. 1b**) and three trajectories leading to myeloid, erythroid and lymphoid precursors. First, to check the validity of the structure, we used the assigned labels in the original study, which were derived by FACS sorting the different populations (when multiple labels were assigned based on different gates, we gave priority to narrow gates to obtain a unique label for each cell). As expected, HSCs progress into MPPs and then bifurcate into LMPPs and CMPs. Then CMPs differentiate into MEPs and GMPs respectively, hence accurately recapitulating known bifurcation events.

Second, using STREAM we rediscovered known marker genes, including diverging genes between two branches and transition genes along each branch. On the CMPs bifurcation which leads to GMPs (S3,S4) and MEPs (S3,S5) populations, STREAM detects diverging genes including GMPs-specific genes like *Epx*, *Prtn3*, *Mpo* , and MEPs-specific genes like *Car2*, *Gata1*, *Mfsd2b*[2, 3, 4](**Fig.1c, Supplementary Figure 2a**). Along the MEP-committed trajectory (S3, S4), STREAM also recovered genes whose expression significantly correlates ($p<1E-4$, Spearman correlation) with the pseudotime progression. We recovered genes previously described [5, 6, 7, 8] that are progressively and precisely downregulated such as *Tmsb4x*, *Coro1a* or upregulated like *Blvrb*, *Ces2g* (**Fig. 1c, Supplementary Figure 2b**). We also identified root and terminal branches specific genes (either upregulated or downregulated), defined as *leaf genes*. For example, *Flt3*, *Procr* are specifically upregulated on lymphoid branch and HSC respectively[1, 9], while *Txnip* and *Emb* are specifically downregulated on myeloid and erythroid branch respectively[10, 11].

Along the lymphoid differentiation trajectory (S2,S1,S0), STREAM identified HSCs-specific genes like *Mpl*[12],*Tgm2*[13] (**Supplementary Figure 2**), whose expressions are repressed towards lymphoid differentiation, and LMPPs-specific genes like *lghv1-81*, *Ccl3* (**Supplementary Figure 2**), whose expressions are activated during the differentiation as discussed in the original study[1]. Taken together, these analyses validate the accuracy of trajectory reconstruction of STREAM in recapitulating key bifurcation events and regulators of early blood development differentiation at single-cell resolution.

Supplementary Note 2: STREAM trajectory inference in high dimensional spaces

As discussed in the main text, we were able to recover four different lineages in zebrafish hematopoietic inDrop data. We hypothesized that we cannot separate B and T cells since the number of dimensions used to infer the trajectories may be insufficient to fully recapitulate all the lineages. Since Monocle2 is one of the state-of-the-art methods designed to capture complex trajectories, we decided to qualitatively compare the inferred trajectories of STREAM and Monocle2 for this challenging dataset. We observed indeed that with less than 4 components both methods failed to recover the B and T lymphoid branches (**Supplementary Figure 4b,c**) but accurately identified the neutrophil, macrophage and lymphoid branches. However, Monocle2 failed to recover a reasonable starting branch for stem cells that are instead mixed with neutrophils. We also observed that with Monocle2 the progenitor cells are misplaced in the developmental tree since they are assigned after the first branching event that gives raise instead to three separate erythroid branches. For STREAM, we observed that simply increasing the number of dimensions may introduce artifacts (trivial branches). Based on the revised seeding strategy (**Methods** and **Supplementary Figure 4a**) and using 20 dimensions as in the original study by Tang et al. [14], we obtained more detailed trajectories and lineage separation (**Supplementary Figure**

4b). First, we were able to clearly separate lymphoid cells into B cells and T/NK cells. Second, we uncovered a rare and well separated branch that contains only the cell type labeled as "Macrophages/Myeloid" by Tang et al (comprising less than 1.36% of the marrow). The previous original study could not assign these unique cell types as arising in either the myeloid or lymphoid cell lineages. By contrast, we observed that the branch capturing these cells was closer to the branch capturing the lymphoid cells. These results were verified by independent analyses using Monocle2 and UMAP (**Supplementary Figure 4c-d**), each of which confirmed shared lineage with lymphoid cells. As reported previously[14], we also observe that these cells express well-known macrophage marker genes including *mpeg1.1*, *ccr9b*, *tlr7*, *ccl39.3*, and *p2rx3b*. Notably, our marker gene detection analysis uncovered genes that are exquisitely specific for this branch such as *irf8* and *ctsbb* (**Supplementary Figure 5**). We also observed genes in the main Macrophages clade that are not expressed in this rare subpopulation such as *mfap4*, *grn1* or *marco* (**Supplementary Figure 5**). As such, we believe that these cells correspond to a separate rare cell type/state. Third, we were able to uncover novel sub-branches within both the macrophages and neutrophil groups. Diversity in these lineages is well-known in mouse and human. For example, N1 and N2 neutrophils exert a wide diversity of cellular responses in cancer following polarization into each cell state by TGF-b and type 1 interferons, respectively. In addition, several cell states for neutrophils have been previously described such as immature, resting, primed, and active[15]. It is also well-appreciated that macrophages have a diversity of cellular states that depends on the age of animal, infection/disease type, and tissue in which macrophages become active[16, 17, 18], and several studies have identified in zebrafish macrophage subsets with important functional differences[19, 20]. Notably, several genes that are uniquely expressed in these sub-branches of the neutrophil and macrophage lineages are shown in **Supplementary Figure 5**. Taken together we believe that these new sub-branches correspond to separate cell types/states and that further experimental analyses are necessary to fully characterize the identity of these populations.

Also, in this case, we compared our results in the 20-dimensional space with Monocle2. Both methods separated lymphoid cells into B cells and T cells. However, Monocle2 does not map the progenitor cells to an intermediate branch toward more defined lineages, instead these cells are placed on a separate and terminal branch that does not progress further. In addition, Monocle2 detects three erythroid branches with 20 dimensions while with 4 dimensions four separate erythroid branches were recovered, making the interpretation of this refined analysis not intuitive. STREAM instead maintains a single branch for the erythroid cells in both spaces. Apart from these differences, both methods further detected multiple sub-branches for the macrophage and neutrophil lineages.

To further validate the generalizability of our approach to learn complex trajectories using STREAM, we also re-analyzed a challenging dataset generated by Paul et al.[3] , where ~2700 myeloid cells were separated into CMP, MEP and GMP based on the surface markers and profiled using MARS-seq. In this study, clustering analysis uncovered 6 main cell types, including erythrocytes, megakaryocytes, DC, basophil/eosinophil progenitors, monocytes, neutrophils. Here, our STREAM analysis identified a simple bifurcation, which corresponds to the differentiation of CMP cells to GMP cells and erythroid cells. More refined analysis (by changing the dimensions from 2 to 10) recovers all the six different committed cell branches defined by the original study (**Supplementary Figure 6**).

Supplementary Note 3: Comparison of STREAM with existing methods

First, we discuss the core algorithms and limitations of all method considered in comparison with STREAM. Monocle2 [21]uses reversed graph embedding (by default DDRtree) to learn an explicit principal graph to describe the single-cell transcriptomic data. During the principal graph learning step, in each iteration, DDRtree moves cells to the nearest vertex, hence distorting the original configuration of cells in the manifold that may result in an uneven distribution with more cells close to vertices and fewer cells in between (**Supplementary Figure 7,8**). Mpath[22] first clusters cells and designates landmarks. Then, based on the landmarks a weighted neighborhood network is constructed and

subsequently trimmed to obtain a state transition network. Mpath requires prior information (e.g. FACS sorting and time points) to designate landmarks and the final transition network is sensitive to the chosen landmarks. Wishbone[23] builds a kNN graph based on the most relevant diffusion components by using gene set enrichment analysis (GSEA), then a random sample of cells termed waypoints are used for ordering cells and branch identification based on the inconsistency between "waypoints". By default, Wishbone can only detect a single bifurcation (i.e. two cell fates). DPT[24] first reduces the dimensionality using a diffusion map approach. Then in the diffusion map space a random-walk-based distance is computed. Branching points are identified by comparing two DPT orderings over cells. SLICER[25] uses locally linear embedding (LLE) to reduce the dimension and then uses a KNN graph to order cells by the shortest path distance from root cell. Branches are detected based on geodesic entropy. All these three methods, i.e., Wishbone, DPT and SLICER cannot infer trajectories without specifying a start cell. In addition, they cannot explicitly show cellular trajectories with a specific topological structure. Instead they simply visualize cells using the first two components of the dimensionality reduction method adopted. scTDA[26] uses the Mapper algorithm[27] to build a topological representation. Briefly, a low dimensional space (obtained for example by multidimensional scaling (MDS)) is first divided into overlapping bins and cells within each bin are clustered in the original space. The topological structure is obtained by connecting clusters that share at least one cell. However, spurious edges may appear since an edge could be formed as long as two clusters have a non-empty intersection. In addition, the obtained topology may also depend on the binning strategy used. TSCAN[28] applies principal component analysis to a gene-cluster level expression matrix. Then top principal components (PCs) are selected to cluster cells and a minimum spanning tree (MST) is constructed based on the clustering solution. Finally, cells are projected and ordered on the tree structure. By default, TSCAN does not report branching events, in fact reports only the linear path with the largest number of clusters. SCUBA[29] first constructs a binary tree by iteratively clustering and refines the tree based on a penalized likelihood function. Then it models gene dynamics using bifurcation theory. This method can infer multiple bifurcation events however cannot model branching events with more than two cells fates. GPfates[30] uses OMGP (Overlapping Mixture of Gaussian Processes) to model the temporal dynamics. However, it requires a prespecified number of trends/trajectories. In addition, both SCUBA and GPfates requires temporal information as input to model the dynamics. SCUBA uses principal curve in tSNE space to infer cell order. GPfates uses GPLVM (Gaussian Process Latent Variable Model) to infer pseudotime. PHATE[31] is a visualization method that preserves well single-cell trajectory structure based on data diffusion but does not provide branch assignment and pseudotime information.

Next, to qualitatively compare different trajectory inference methods, we have considered several features summarized in **Supplementary Table 1** and explained below:

Structure refers to the hypothetical structures different methods are able to infer without modifying their original codes.

Deterministic refers to the ability of a method to consistently produce the same output if applied multiple times on the same input. For both SCUBA and Wishbone, the indeterminism is caused by the tSNE step since SCUBA learns pseudotime in the tSNE space and Wishbone visualizes the final trajectories in the tSNE plot. In scTDA, the topological analysis *RootedGraph()* returns different results on different runs. In GPfates, both dimensionality reduction (Bayesian GPLVM) and trajectory inference steps (Overlapping Mixture of Gaussian Processes (OMGP)) often do not converge to the same solution.

Ease of installation refers to the ability to install a method via a public package management system (Bioconda, pypi, CRAN, Bioconductor, etc.) or to use it through an accessible website (++) or through a local custom setup script that may need extra packages to be installed ('+').

Extra input indicates that extra information is required in order to perform trajectory inference.

Explicit trajectory visualization: refers to the ability of a method to provide explicitly a tree (or graph) skeleton based on a set of curves to describe the cellular developmental trajectories.

Single-cell-level trajectory visualization refers to the ability of a method to explicitly align single cells to the trajectories. Some methods provide instead only an abstract tree (or graph) where each node represent either the cluster size or the expression variance, however cells cannot be displayed individually.

Density level trajectory visualization refers to the ability of a method to display the cell density along a given trajectory.

Automatic marker gene detection indicates whether the tool supports automatic marker gene discovery for the different branches or paths.

Mapping feature refers to the ability to fix a reference structure and to map new cells to it without recomputing trajectories.

Tested on epigenomic data means that the method has been previously applied to epigenomic data.

End-to-end pipeline for scATAC-seq means that the method provides all the functions necessary to perform trajectory inference starting from raw count data.
Next we describe in detail the comparison based on the synthetic dataset by Rizvi et al [26], presented in the main text.

PHATE, a dimensionality reduction method, qualitatively preserves cellular trajectories but does not provide branch assignment information for cells. STREAM, scTDA, Monocle2, and Mpath can accurately reconstruct two bifurcation events. SLICER detects two bifurcation events, but the branching node is not accurately located. Wishbone instead can only detect one simple bifurcation even though the obtained 2D manifold clearly shows two bifurcations. DPT detects too many branches and their positions are difficult to assess from the proposed visualization. GPFates requires to pre-specify the number of trajectories (referred to as trends in the original study and manually set for this dataset to 3). Although the generated curves initially follow the correct branches, they incorrectly converge at the end. SCUBA fails to detect bifurcation events in this dataset. We also noticed that scTDA, SCUBA, and Mpath do not provide single-cell-resolution visualization. Wishbone, DPT and SLICER do not provide explicit trajectory visualization, so they cannot visualize both time points and recovered branch assignments in the same plot. Monocle2 provides an explicit trajectory visualization at single-cell resolution, however it doesn't provide information about cell density and the population composition.

To make our comparison analyses for both synthetic and real datasets reproducible, we also provide Jupyter notebooks documenting them (**Supplementary Data 2**). In addition, we summarize below some important details on the data and procedures used to run each method and if it was necessary to run them with non-standard parameters.

For all datasets, we kept all the cells during the comparison analyses. For the synthetic and qPCR datasets, we used all the genes (500 genes and 175 genes respectively). For the scRNA-seq dataset, to fairly assess the trajectory inference procedure and to mitigate the effect of different gene selection methods, we used the same set of variable genes (835 genes) selected using the function *select_variable_genes()* in STREAM with default parameters. By default, we log2-transformed the gene expression matrices unless a method explicitly requires the raw count data. As mentioned above,

we tried to follow the method documentation and used the default parameters as much as possible. GPFates requires time information to perform trajectory inference. The default pseudotime inference method (Bayesian GPLVM) that should be used initially to recover the time information does not work well when the selected genes are highly independent. As suggested by the author we used DPT to infer pseudotime before inferring trajectories with GPFates. For indeterministic methods, we randomly picked one result from several runs.

Supplementary Note 4: STREAM interactive website

In order to make STREAM user-friendly and accessible to non-bioinformatician, we have created an interactive website: http://stream.pinellolab.org. The website implements the main features of the command line version and provides interactive and exploratory panels to zoom and visualize single-cells on any given branch.

The website offers two functions: 1) to run STREAM on single-cell transcriptomic or epigenomic data provided by the users and 2) the first interactive database of precomputed trajectories with results for seven published datasets[1, 14, 32, 33, 34, 35, 36]. The users can visualize and explore cells' developmental trajectories, subpopulations and their gene expression patterns at single-cell level. (**Supplementary Figure 11**).

The website can also run on a local machine using the provided Docker image we have created.

To get the Docker image, simply execute the following command:

```
docker pull pinellolab/stream_web
```

After this step, to run the website on the local machine execute the following command:

```
docker run -p 10001:10001 pinellolab/stream_web
```

After the execution of this command the user will have a local instance of the website that can be opened with any browser (Chrome, Firefox, Safari, etc.) and accessible at the URL:

http://localhost:10001

As in the hosted version, using the local version of the STREAM webpage, users can compute and explore trajectories on the page 'Compute'. On this page the users can also optionally create and save a summary report in a .zip file. The obtained report can be shared with other users and/or visualized interactively and without recomputing trajectories on the page 'Visualize'.

Supplementary Note 5: STREAM bioconda package

In order to provide more control to advanced users, we have created also a Python package that can be used to perform fully customizable step-by-step analysis. This package can be easily installed using the Bioconda package channel[37]. The STREAM bioconda package can be installed following these three simple steps:

1) If Anaconda (or miniconda) is already installed with Python 3 skip to 2) otherwise download and install Python3 Anaconda from here: https://www.anaconda.com/download/

2) Add the Bioconda channel with the following commands:

```
conda config --add channels defaults
conda config --add channels bioconda
```

```
conda config --add channels conda-forge
```

3) Create an environment named 'stream_env', install stream, jupyter and activate it with the following commands:

For single cell RNA-seq analysis:

```
conda create -n stream_env python=3.6 stream jupyter
conda activate stream_env
```

For single cell ATAC-seq analysis:

```
conda create -n stream_env python=3.6 stream stream_atac jupyter
conda activate stream_env
```

4. To perform STREAM analyses in Jupyter Notebook as illustrated in the **Tutorials**, type jupyter notebook within the stream_env environment i.e.:

```
jupyter notebook
```

Tutorials:

1. Example for scRNA-seq: https://nbviewer.jupyter.org/github/pinellolab/STREAM/blob/master/tutorial/1.STREAM_scRNA-seq.ipynb
2. Example for *mapping* feature: https://nbviewer.jupyter.org/github/pinellolab/STREAM/blob/master/tutorial/2.STREAM_mapping.ipynb
3. Example for complex trajectories: https://nbviewer.jupyter.org/github/pinellolab/STREAM/blob/master/tutorial/3.STREAM_complex_trajectories.ipynb
4. Example for scATAC-seq: https://nbviewer.jupyter.org/github/pinellolab/STREAM/blob/master/tutorial/4.STREAM_scATAC-seq.ipynb

All the analysis results can be saved to a single .zip file by executing the following command:

```
save_web_report()
```

Later, the saved zip file can be imported and interactively visualized on the STREAM webpage.

The package stream contains several functions that cover all the analyses presented in the paper and the single cell specific extension of ElPiGraph.

For transcriptomic data analysis, the main functions are:

- Read in gene expression matrix*, read()*
- Feature selection: *select_variable_genes()*
- Dimensionality reduction: *dimension_reduction()*
- Seeding initial tree structure in low dimension space: *seed_elastic_principal_graph()*
- Seeding initial tree structure in high dimension space: *infer_initial_structure()*
- Elastic principal graph: *elastic_principal_graph()*

- Prune branches: *prune_elastic_principal_graph()*
- Finetune branching nodes: *optimize_branching()*
- Shift branching nodes: *shift_branching()*
- Extend leaf nodes: *extend_elastic_principal_graph()*
- Flat Tree Plot: *plot_flat_tree()*
- Subway map plot: *subwaymap_plot()* and *subwaymap_plot_gene()*
- Stream plot: *stream_plot()* and *stream_plot_gene()*
- Diverging gene detection: *detect_de_genes()*
- Transition gene detection: *detect_transistion_genes()*
- Leaf gene detection: *detect_leaf_genes()*
- Mapping procedure: *map_new_data()*

To load and use 10x Genomics single cell RNA-seq data processed with Cell Ranger it is necessary to set the parameter 'file_format' to 'mtx', e.g.:

```
adata=st.read(file_name='./filtered_gene_bc_matrices/hg19/matrix.mtx',file_format
='mtx')
```

For the scATAC-seq data analysis, a separate preprocessing function from the stream_atac is required before running the main trajectory inference step:

Read in count file, sample file and region file and convert read count to k-mer z-score:

```
import stream_atac

adata = stream_atac.preprocess_atac(file_count='./count_file.tsv.gz',

file_region='./region_file.bed.gz',file_sample='./sample_file.tsv.gz')
```

To load and use 10x Genomics single cell ATAC-seq data processed with Cell Ranger, as in the case of the scRNA-seq, it is necessary to set the parameter 'file_format' to 'mtx', e.g.:

```
import stream_atac

adata =
stream_atac .preprocess_atac(file_count='./filtered_peak_bc_matrix/matrix.mtx',

file_sample='./filtered_peak_bc_matrix/barcodes.tsv',
file_region='./filtered_peak_bc_matrix/peaks.bed',file_format='mtx')
```

We provide all the scripts used for the analyses in this paper as Jupyter Notebooks (**Supplementary Data 1**). These notebooks can be used as tutorials since they nicely show how to use and combine the functions listed in this section and also illustrate how to set the different parameters to perform different analyses with STREAM.

Supplementary Note 6: STREAM command line interface

STREAM can be easily used also with a simple command line interface. It is possible to install the command line version of STREAM with Docker.

Installation with Docker

With Docker no installation is required, the only dependence is Docker itself. Docker can be downloaded freely from here: https://store.docker.com/search?offering=community&type=edition

To get a local copy of STREAM execute the following command:

```
docker pull pinellolab/stream
```

STREAM usage and example dataset

The main and required input file is a tab-separated gene expression matrix (raw counts or scaled) in. tsv format. Each row represents a unique gene and each column is one cell.

The following table shows the first 5 rows (genes) and 5 columns (cells) of the provided example dataset

	HSC1	HSC1.1	HSC1.2	HSC1.3	HSC1.4
CD52	6.479620	0.000000	0.000000	5.550051	0.000000
Ifitm1	11.688533	11.390682	10.561844	11.874295	8.976571
Cdkn3	0.000000	0.000000	0.000000	0.000000	8.293616
Ly6a	10.417026	11.452145	0.000000	8.158840	8.945882
Bax	6.911608	10.201157	0.000000	9.396073	0.000000

In addition, it is possible to provide these optional files in .tsv format:

1. **Cell labels**: Each item can be a putative cell type or sampling time point obtained from experiments. Cell labels are helpful for visually validating the inferred trajectory. This file must be in .tsv format. The order of labels should be consistent with cell order in the gene expression matrix file. No header is necessary:

    HSC
    HSC
    GMP
    MEP
    MEP
    MPP
    GMP
    GMP
    ⋮

2. **Cell label color**: Customized colors to use for the different cell labels. The first column specifies cell labels and the second column specifies the color in the format of hex. No header is necessary:

```
HSC	#7DD2D9
MPP	#FFA500
CMP	#e55b54
GMP	#5dab5a
MEP	#166FD5
CLP	#989797
⋮
```

3. **Gene list:** It contains genes that users may be interested in visualizing in subway map and stream plot in addition to the genes detected by STREAM. Genes are listed in one column. No header is necessary:

```
Ifitm1
Cdkn3
Ly6a
CD52
Foxo1
⋮
```

4. **Feature genes:** It contains genes that the user can specify and that are used as features to infer trajectories. instead of using the automatic feature selection of STREAM. No header is necessary:

```
Gata1
Pax5
CD63
Klf1
Lmo2
⋮
```

To run STREAM, after the installation at the command-line interface execute:

```
$ docker run -v ${PWD}:/data -w /data pinellolab/stream --help [options]
```

Users can specify the following options:

```
-m, --matrix
input file name. Matrix is in .tsv or tsv.gz format in which each row represents a unique gene and
each column is one cell. (default: None)
-l, --cell_labels
file name of cell labels (default: None)
-c, --cell_labels_colors
file name of cell label colors (default: None)
-s, --select_features
LOESS, PCA, all: Select variable genes using LOESS or top principal components using PCA or keep
all the gene (default: LOESS)
--TG
detect transition genes automatically
--DE
detect DE genes automatically
--LG
etect leaf genes automatically
-g, --gene_list
genes to visualize, it can either be filename which contains all the genes in one column or a set
of gene names separated by comma (default: None)
-p, --use_precomputed
use precomputed data files without re-computing structure learning part
--log2
perform log2 transformation
--norm
```

```
normalize data based on library size
--atac
indicate scATAC-seq data
--n_processes
Specify the number of processes to use. (default, all the available cores).
--loess_frac
The fraction of the data used in LOESS regression (default: 0.1)
--pca_first_PC
keep first PC
--pca_n_PC
The number of selected PCs (default: 15)
--n_processes
Specify the number of processes to use. The default uses all the cores available
--lle_neighbours
LLE neighbour percent (default: 0.1)
--lle_components
Number of components for LLE space (default: 3)
--clustering
Clustering method used for seeding the intial structure, choose from 'ap','kmeans','sc'.
--damping
Affinity Propagation: damping factor (default: 0.75)
--n_clusters
Number of clusters for spectral clustering or kmeans
--EPG_n_nodes
Number of nodes for elastic principal graph (default: 50)
--EPG_lambda
lambda parameter used to compute the elastic energy (default: 0.02)
--EPG_mu
mu parameter used to compute the elastic energy (default: 0.1)
--EPG_trimmingradius
maximal distance of point from a node to affect its embedment (default: Inf)
--EPG_alpha
positive numeric, alpha parameter of the penalized elastic energy (default: 0.02)
--disable_EPG_optimize
disable optimizing branching
--EPG_collapse
Collapsing small branches
--EPG_collapse_mode
the mode used to collapse branches. It can be 'PointNumber','PointNumber_Extrema',
'PointNumber_Leaves','EdgesNumber' or 'EdgesLength' (default:'PointNumber')
--EPG_collapse_par
the control parameter used for collapsing small branches
--EPG_shift
shift branching point
--EPG_shift_mode
the mode to use to shift the branching points 'NodePoints' or 'NodeDensity' (default: NodeDensity)
--EPG_shift_DR
positive numeric, the radius used when computing point density if EPG_shift_mode is 'NodeDensity'
(default:0.05)
--EPG_shift_maxshift
positive integer, the maximum distance (number of edges) to consider when exploring the branching
point neighborhood (default:5)
--disable_EPG_ext
disable extending leaves with additional nodes
--EPG_ext_mode
the mode used to extend the graph. It can be 'QuantDists', 'QuantCentroid' or 'WeigthedCentroid'.
(default: QuantDists)
--EPG_ext_par
the control parameter used for contribution of the different data points when extending leaves with
nodes (default: 0.5)
--DE_zscore_cutoff
Differentially Expressed Genes z-score cutoff (default: 2)
--DE_logfc_cutoff
Differentially Expressed Genes log fold change cutoff (default: 0.25)
--TG_spearman_cutoff
Transition Genes Spearman correlation cutoff (default: 0.4)
--TG_logfc_cutoff
Transition Genes log fold change cutoff (default: 0.25)
--LG_zscore_cutoff
```

```
Leaf Genes z-score cutoff (default: 1.5)
--LG_pvalue_cutoff
Leaf Genes p value cutoff (default: 1e-2)
--umap
Whether to use UMAP for visualization (default: No)
-r
root node for subwaymap_plot and stream_plot (default:None)
--stream_log_view
use log2 scale for y axis of stream_plot
--for_web
Output files for website
-o, --output_folder
Output folder (default: None)
--new
file name of data to be mapped (default: None)
--new_l
filename of new cell labels (default: None)
--new_c
filename of new cell label colors (default: None)
```

Example with transcriptomic data: Here we we take a single cell RNA-seq dataset as an example, including `data_Nestorowa.tsv.gz, cell_label.tsv.gz` and `cell_label_color.tsv.gz` (Nestorowa, S. et al.,2016), and assuming that they are in the **current folder**, to perform trajectory inference analysis, users can simply run a single command:

Using Bioconda:

```
$ stream -m data_Nestorowa.tsv.gz -l cell_label.tsv.gz -c cell_label_color.tsv.gz
```

Using Docker:

```
$ docker run  -v ${PWD}:/data -w /data  pinellolab/stream -m
data_Nestorowa.tsv.gz -l cell_label.tsv.gz -c cell_label_color.tsv.gz
```

If cell labels are not available or no customized cell label color file is available, `-l` or `-c` can also be omitted

Using Bioconda:

```
$ stream -m data_Nestorowa.tsv.gz
```

Using Docker:

```
$ docker run -v ${PWD}:/data -w /data pinellolab/stream -m data_Nestorowa.tsv.gz
```

To visualize genes of interest, user can provide a gene list file by adding `-g`, for example: `gene_list.tsv.gz`. Meanwhile, by adding the flag `-p`, STREAM will use the precomputed file obtained from the first running (In this way, STREAM will import precomupted pkl file so the analysis will skip structure learning part and only execute the step of visualizing genes):

Using Bioconda:

```
$ stream -m data_Nestorowa.tsv.gz -l cell_label.tsv.gz -c cell_label_color.tsv.gz
-g gene_list.tsv.gz -p
```

Using Docker:

```
$ docker run -v ${PWD}:/data -w /data pinellolab/stream -m data_Nestorowa.tsv.gz
-l cell_label.tsv.gz -c cell_label_color.tsv.gz -g gene_list.tsv.gz -p
```

Users can also provide a set of gene names separated by comma or specify the root by adding `-r`:

Using Bioconda:

```
$ stream -m data_Nestorowa.tsv.gz -l cell_label.tsv.gz -c cell_label_color.tsv.gz
-g Gata1,Mpo -r S1 -p
```

Using Docker:

```
$ docker run -v ${PWD}:/data -w /data pinellolab/stream -m data_Nestorowa.tsv.gz
-l cell_label.tsv.gz -c cell_label_color.tsv.gz -g Gata1,Mpo -r S1 -p
```

To explore potential marker genes, it is possible to add the flags `--DE`, `--TG`, or `--LG` to detect DE (differentially expressed) genes, transition gens, and leaf genes respectively:

Using Bioconda:

```
$ stream -m data_Nestorowa.tsv.gz -l cell_label.tsv.gz -c cell_label_color.tsv.gz
--DE --TG --LG -p
```

Using Docker:

```
$ docker run -v ${PWD}:/data -w /data pinellolab/stream -m data_Nestorowa.tsv.gz
-l cell_label.tsv.gz -c cell_label_color.tsv.gz --DE --TG --LG -p
```

To save a STREAM report zip file that later can be imported and interactively visualized in the STREAM webpage, use the following command (Note the flag `--for_web`):

Using Bioconda:

```
$ stream -m data_Nestorowa.tsv.gz -l cell_label.tsv.gz -c cell_label_color.tsv.gz
--DE --TG --LG --for_web
```

Using Docker:

```
$ docker run  -v ${PWD}:/data -w /data pinellolab/stream -m data_Nestorowa.tsv.gz
-l cell_label.tsv.gz -c cell_label_color.tsv.gz --DE --TG --LG --for_web
```

Example of the mapping feature: To explore the feature mapping, users need to provide two datasets, one is used for inferring trajectories. The other is the dataset that is going to be mapped to the inferred trajectories. Here we take `data_Olsson.tsv.gz`, `data_perturbation.tsv` (Olsson, A. et al.,2016) as an example. We assume that all the datasets are in the current folder.

Users first need to run the following command to get initial inferred trajectories from wild-type cells:

Using Bioconda:

```
$ stream -m data_Olsson.tsv.gz -l cell_label.tsv.gz -c cell_label_color.tsv.gz --
lle_components 4 --EPG_shift
```

Using Docker:

```
$ docker run -v ${PWD}:/data -w /data pinellolab/stream -m data_Olsson.tsv.gz -l
cell_label.tsv.gz -c cell_label_color.tsv.gz --lle_components 4 --EPG_shift
```

To map the genetically perturbed cells to the inferred trajectories, users can execute the following command:

Using Bioconda:

```
$ stream --new data_perturbation.tsv.gz --new_l cell_perturbation_label.tsv.gz --
new_c cell_perturbation_label_color.tsv.gz
```

Using Docker:

```
$ docker run -v ${PWD}:/data -w /data pinellolab/stream --new
data_perturbation.tsv.gz --new_l cell_perturbation_label.tsv.gz --new_c
cell_perturbation_label_color.tsv.gz
```

After running this command, a folder named '`mapping_result`' will be created under the current directory along with all the mapping analysis results.

Example with scATAC-seq data: To perform scATAC-seq trajectory inference analysis, three files are necessary, a .tsv file of counts in compressed sparse format, a sample file in .tsv format and a region file in .bed format:

1. **Count file**: a tab-delimited compressed matrix in sparse format (column-oriented). It contains three columns. The first column specifies the rows indices (the regions) for non-zero entry. The second column specifies the columns indices (the sample) for non-zero entry. The last column contains the number of reads in a given region for a particular cell. No header is necessary:

3735	96	1
432739	171	2
133126	292	1
219297	359	1
284936	1222	1
442588	1580	2

2. **Sample file**: It has one column. Each row is a cell name. The order of the cells should be consistent with the sample indices in count file. No header is necessary:

singles-BM0828-HSC-fresh-151027-1
singles-BM0828-HSC-fresh-151027-2

```
singles-BM0828-HSC-fresh-151027-3
singles-BM0828-HSC-fresh-151027-4
singles-BM0828-HSC-fresh-151027-5
⋮
```

3. **Region file**: a tab-delimited .bed file with three columns. The first column specifies chromosome names. The second column specifies the start position of the region. The third column specifies the end position of the region. The order of regions should be consistent with the region indices in the count file. No header is necessary:

```
chr1    10279   10779
chr1    13252   13752
chr1    16019   16519
chr1    29026   29526
chr1    96364   96864
⋮
```

To perform scATAC-seq trajectory inference analysis, three files are necessary, a .tsv file of counts in compressed sparse format, a sample file in .tsv format and a region file in .bed format. (Buenrostro, J.D. et al., 2018). We assume that **they are in the current folder**.

Using these three files, users can run stream_atac with the following command to preprocess sc-atac-seq data and get a z_score matrix file named `'zscore.tsv.gz'` (This step may take a couple of hours with a modest machine):

Using Bioconda:

```
$ stream_atac -c count_file.tsv.gz -s sample_file.tsv.gz -r region_file.bed.gz
```

Then, take z-score file as input to infer trajectories using stream:

Using Bioconda:

```
$ stream --atac -m zscore.tsv.gz -l cell_label.tsv.gz -c cell_label_color.tsv.gz
--lle_components 4
```

Using Docker:

```
$ docker run -v ${PWD}:/data -w /data pinellolab/stream --atac -m zscore.tsv.gz -
l cell_label.tsv.gz -c cell_label_color.tsv.gz --lle_components 4
```

Output description

STREAM write all the results by default in the folder stream_result, unless a different directory is specified by the user with the flag `-o`. This folder mainly contains the following files and directories:

- **std_vs_means.pdf**: selected most variable genes.
- **dimension_reduction.pdf**: projected cells in the MLLE 3D space.
- **seed_elastic_principal_graph_skeleton.pdf**: the initial structure skeleton with all the nodes and edges.
- **seed_elastic_principal_graph.pdf**: the initial structure with cells.
- **ElPiGraph_analysis.pdf**: the log of ElPiGraph strucuture learning.
- **elastic_principal_graph_skeleton.pdf**: the elastic principal graph skeleton.

- **elastic_principal_graph.pdf**: the elastic principal graph with cells.
- **optimizing_elastic_principal_graph_skeleton.pdf**: the elastic principal graph skeleton after optimizing branching.
- **optimizing_elastic_principal_graph.pdf**: the elastic principal graph with cells after optimizing branching.
- **extending_elastic_principal_graph_skeleton.pdf**: the elastic principal graph with cells after extending leaf nodes.
- **extending_elastic_principal_graph.pdf**: the elastic principal graph skeleton after extending leaf nodes.
- **finalized_elastic_principal_graph_skeleton.pdf**: the finalized elastic principal graph skeleton.
- **finalized_elastic_principal_graph.pdf**: the finalized elastic principal graph with cells.
- **flat_tree.pdf**: flat tree plot.
- **cell_info.tsv**: cell information file containing branch assignment id and pseudotime.
- **stream_result.pkl**: stores anndata object from the analysis. It can be imported later to reproduce the whole analysis.
- sub-folder **'transition_genes'** contains several files, one for each branch id, for example for (S0,S1):
    - **transition_genes_S0_S1.pdf**: Detected transition genes plot for branch S0_S1. Orange bars are genes whose expression values increase from state S0 to S1 and green bars are genes whose expression values decrease from S0 to S1
    - **transition_genes_S0_S1.tsv**: Table that stores information of detected transition genes for branch S1_S2.
- sub-folder **'de_genes'** contains several files, one for each pair of branches, for example for (S0,S1) and (S0,S2):
    - **de_genes_S0_S1 and S0_S2.pdf**: Detected differentially expressed top 15 genes plot. Red bars are genes that have higher gene expression in branch S0_S1, blue bars are genes that have higher gene expression in branch S0_S2
    - **de_genes_greater_S0_S1 and S0_S2.tsv**: Table that stores information of DE genes that have higher expression in branch S0_S1.
    - **de_genes_less_S0_S1 and S0_S2.tsv**: Table that stores information of DE genes that have higher expression in branch S0_S2.
- sub-folder **'leaf_genes'** contains several files:
    - **leaf_genes.tsv**: Table that stores information of leaf genes from all branches.
    - **leaf_genesS0_S1.tsv**: Table that stores information of leaf genes from branch S0_S1.
- sub-folder **'S0'**: contains subway and stream plots for each of the cell states, for example, choosing S0 state as root state:
    - **subway_map.pdf**: single-cell level cellular branches plot
    - **stream_plot.pdf**: density level cellular branches plot
    - **subway_map_gene.pdf**: gene expression pattern on subway map plot
    - **stream_plot_gene.pdf**: gene expression pattern on stream plot

Supplementary Figures

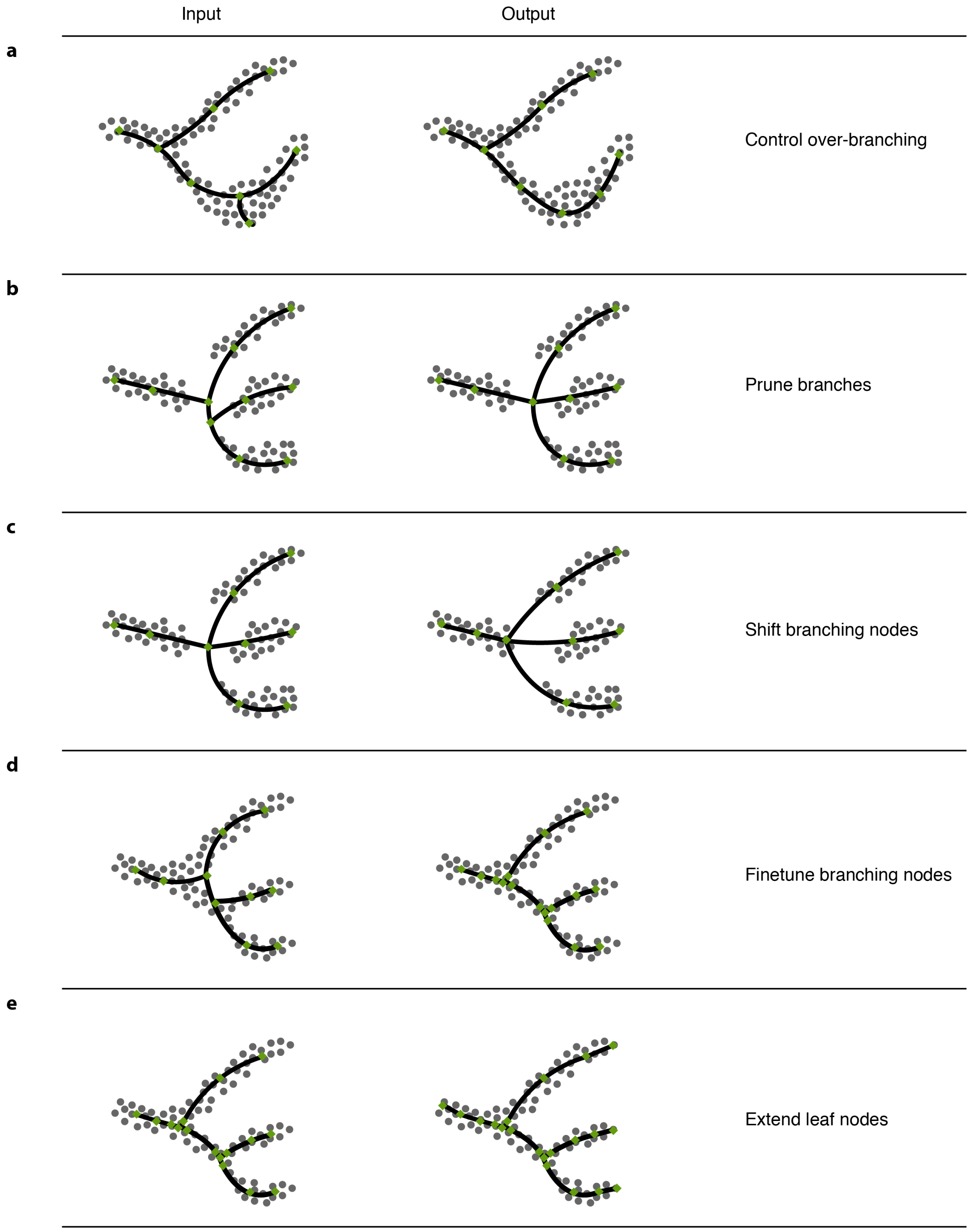

Supplementary Figure 1 Toy examples of five domain specific elastic principal graph optimization steps introduced to model single-cell data. Each grey dot is one single cell. Green square and dark curves represent nodes and edges of principal tree respectively. Panel on the left shows the input configuration. Panel on the right shows the output configuration after the optimization steps. (a) Control over-branching. (b) Pruning branches. (c) Shift branching nodes. (d) Finetune branching nodes. (e) Extend leaf nodes.

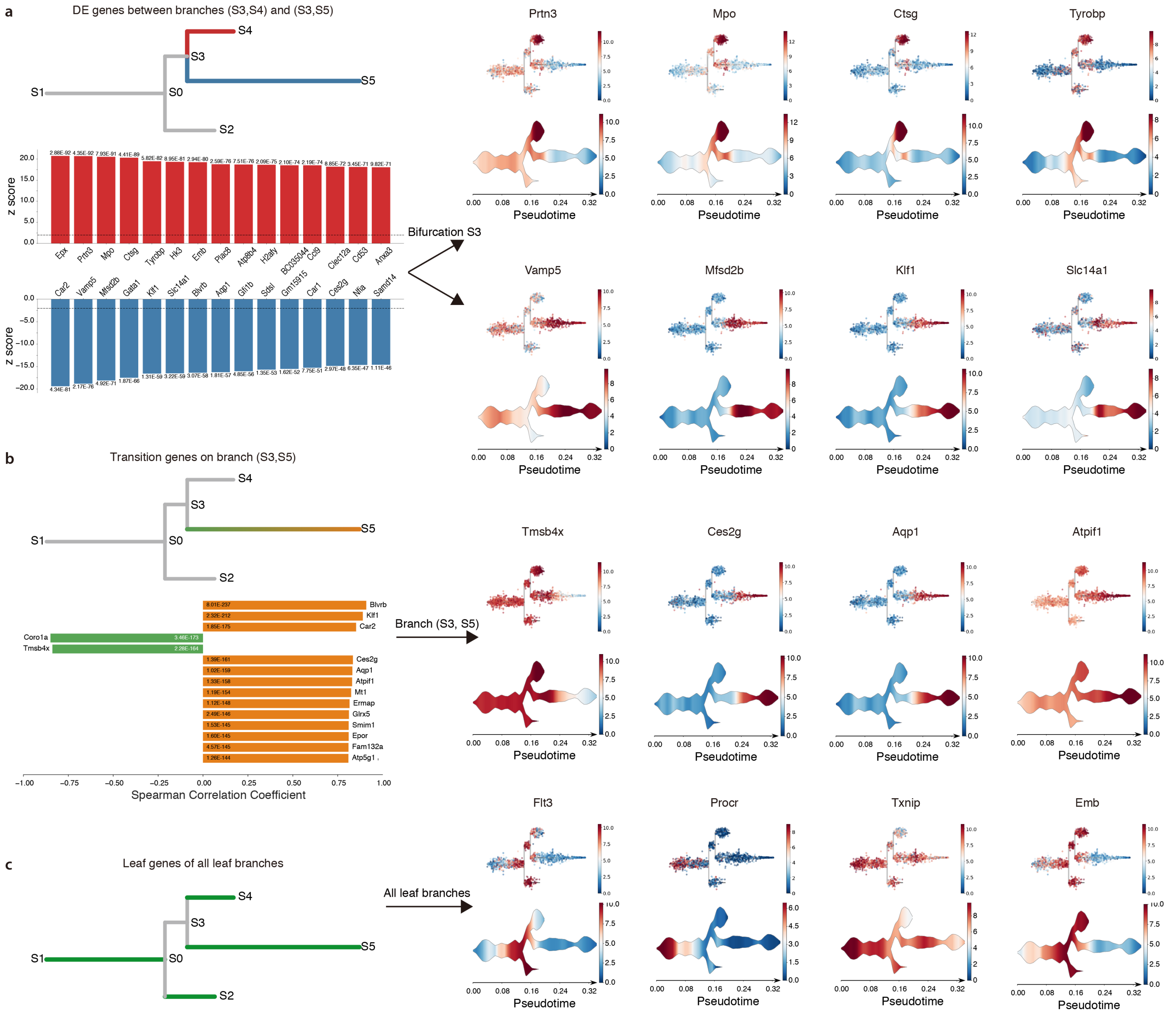

Supplementary Figure 2 STREAM automatically discovers differentially expressed genes and transition genes around in the mouse hematopoietic system. (a)Top left, subway map schematic to highlight the branches (S3,S4)(red), (S3,S5)(blue) used to calculate DE genes. Bottom left, genes highly expressed on branch (S3,S4) (red part) and genes highly expressed on branch (S3,S5) (blue part), sorted by significance. Top right, top detected marker genes for (S3,S4) are visualized on both subway map plots and stream plots. Bottom right, top detected marker genes for (S3,S5) are visualized on both subway map plots and stream plots. (b)Top left, subway map schematic used to highlight the branch (S3,S5) (green to orange gradient) used to calculate transition genes. Bottom left, genes monotonically increasing (orange part) or decreasing (green part) when progressing along branch (S3,S5), sorted by significance. On the right, top detected upregulated and downregulated transition genes along branch (S3,S5) are visualized on both subway map and stream plots. (c) On the left, subway map schematic used to highlight all the leaf branches, that is, (S1,S0),(S3,S4),(S3,S5),(S0,S2). On the right, top leaf gene from each leaf branch is visualized on both subway map plots and stream plots.

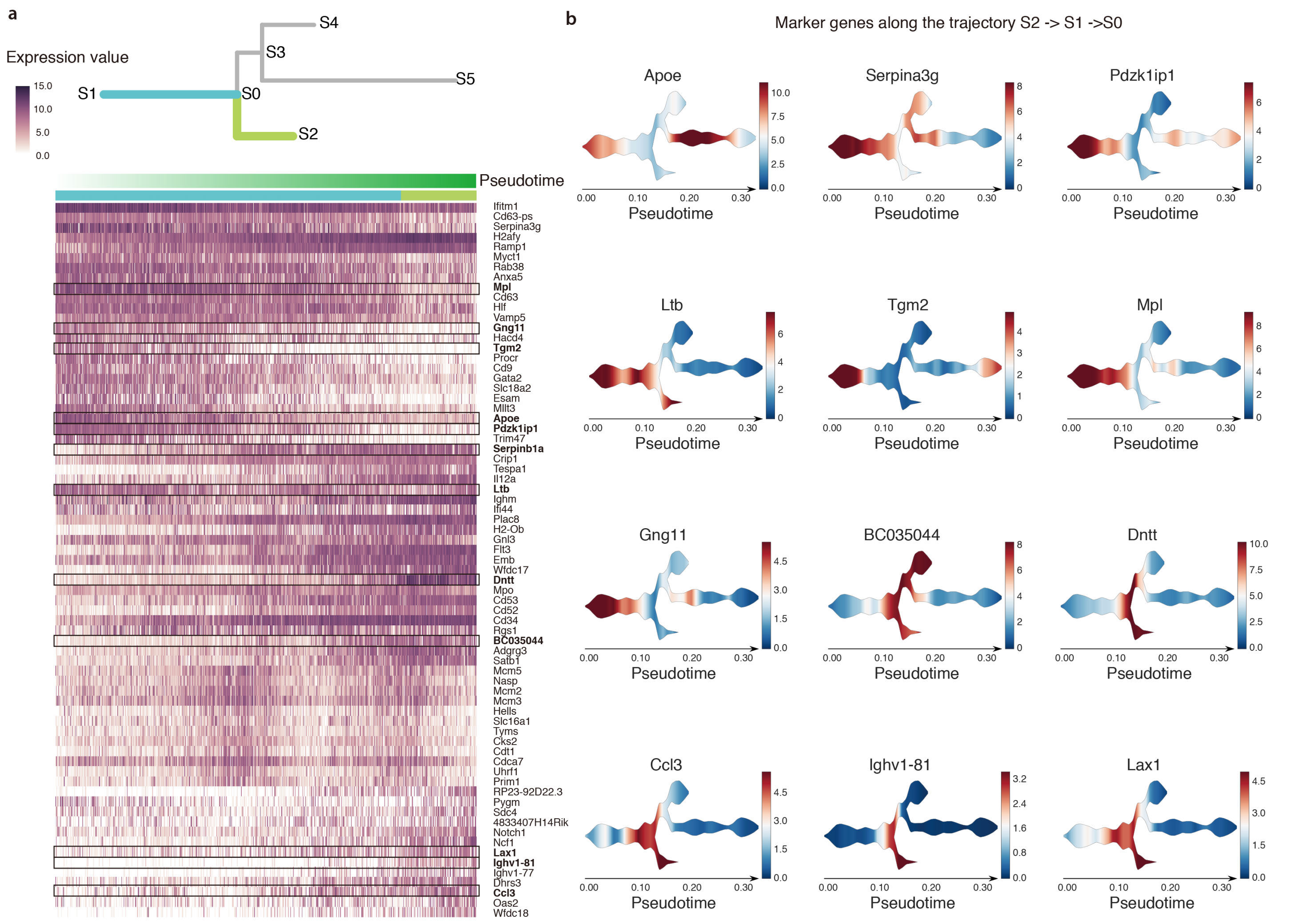

Supplementary Figure 3 STREAM automatically discovers marker genes along lymphoid cells differentiation trajectory in the mouse hematopoietic system. (a)Top left, subway map schematic to highlight the lymphoid cells differentiation trajectory (S1,S0,S2) consisting of two branches (S1,S0)(cyan), (S0,S2)(yellow green), whose related marker genes are calculated. Bottom left, heatmap showing the detected marker genes expression. Each row indicates one marker gene. Each column indicates one cell on the trajectory (S1,S0,S2). Cells are ordered by the inferred pseudotime from STREAM. The two colors represent cells' branch ID assignment, either branch (S1,S0) or branch (S0,S2). (b) Twelve marker genes selected from left heatmap are visualized on stream plots.

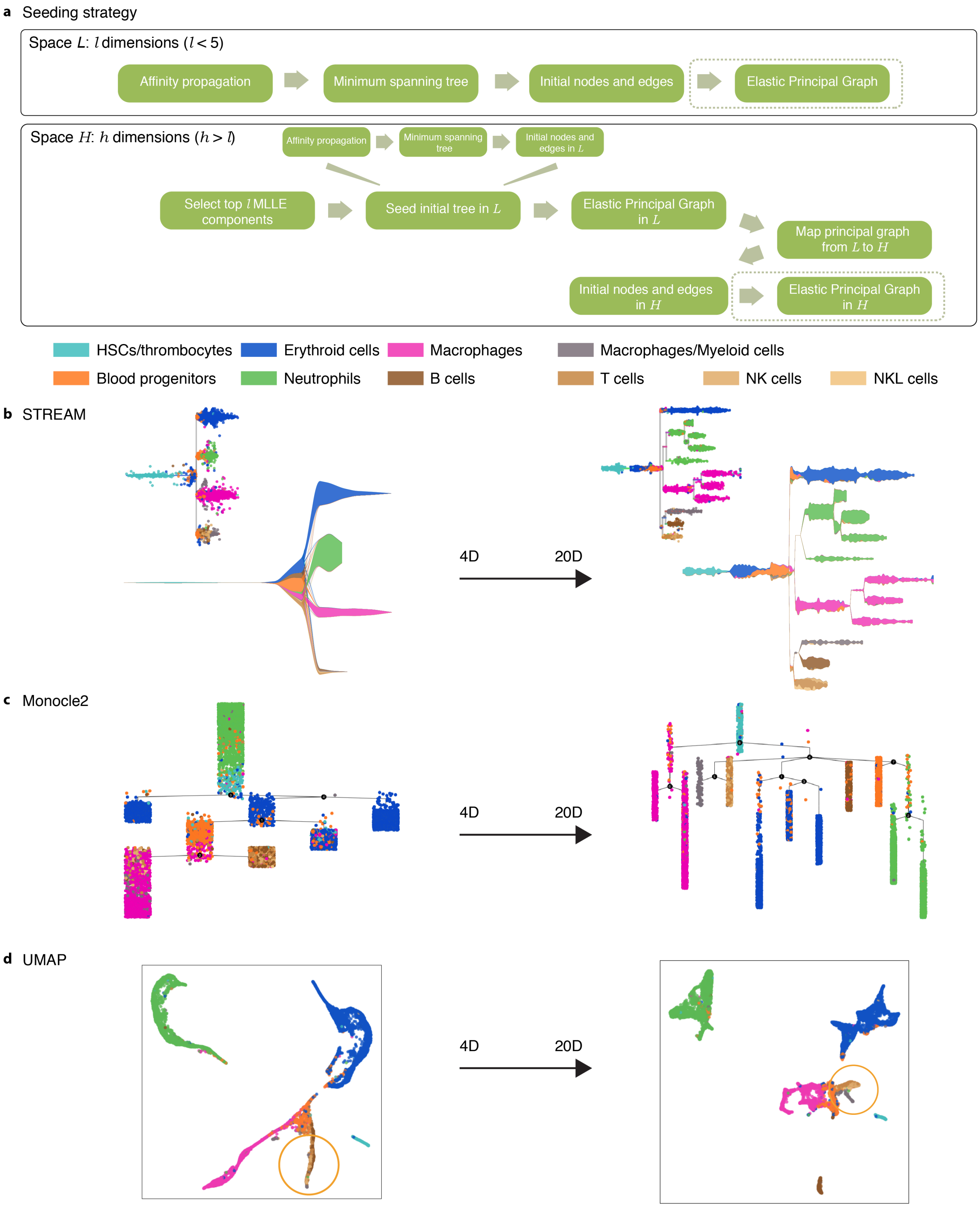

Supplementary Figure 4 STREAM trajectory inference in high-dimensional space using zebrafish inDrop data. (a) Seeding strategy in STREAM. Top panel shows the workflow of seeding initial tree structure in low dimensional space. Bottom panel shows the workflow of seeding initial tree structure in high dimensional space. (b) Left, trajectories inferred by STREAM in 4-dimension space, right, trajectories inferred by STREAM in 20-dimension. (c) Left, trajectories inferred by Monocle2 in 4-dimension space, right, trajectories inferred by Monocle2 in 20-dimension space. (d) Left, visualization of cells by UMAP based on top 4 MLLE components, right, visualization of cells by UMAP based on top 20 MLLE components. The circles indicate the location of ‘Macrophages/Myeloid cells’.

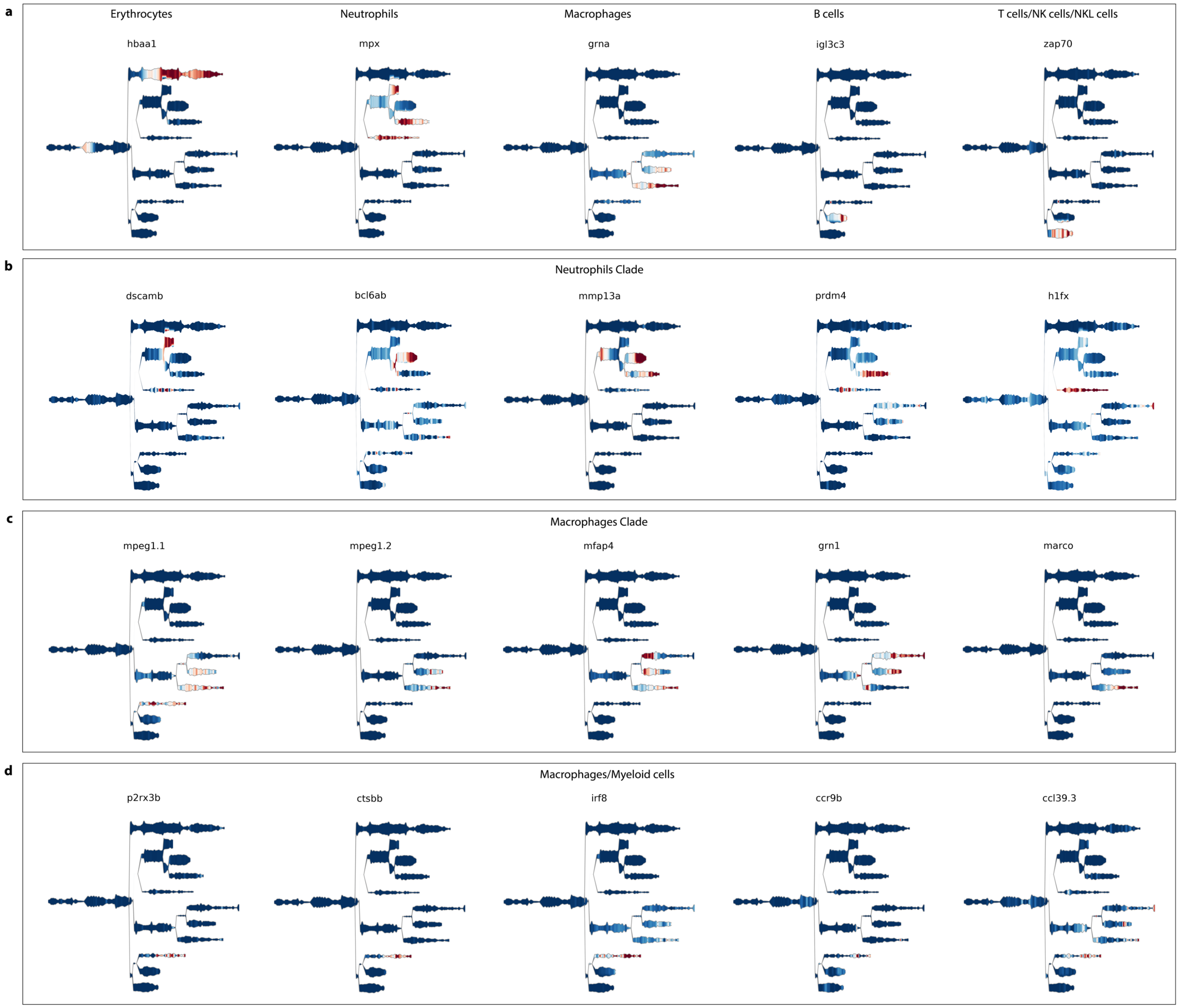

Supplementary Figure 5 STREAM automatically discovers marker genes in high-dimensional space in zebrafish inDrop data. **(**a) Stream plots of marker genes recovered for the different lineages. (b) Stream plots of marker genes discovered within the neutrophils clade. (c) Stream plots of marker genes discovered within the macrophages clade. (d) Stream plots of marker genes discovered for the branch corresponding to the Macrophages/Myeloid cells.

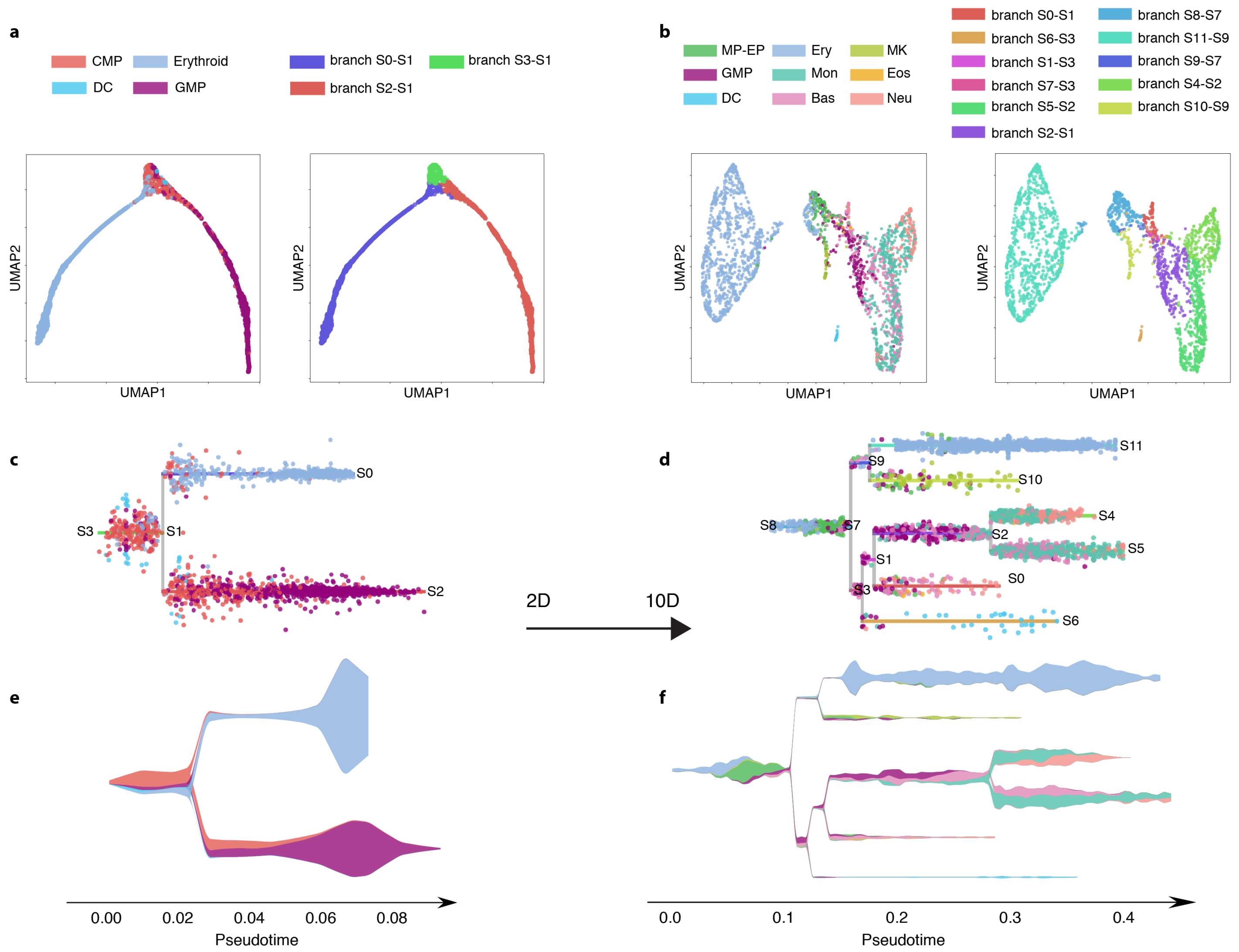

Supplementary Figure 6 STREAM trajectory inference in high-dimensional space in mouse MARS-seq data. (a) Visualization of cells by UMAP based on top two MLLE components. Left, cells are colored based on the labels from original study. Right, cells are colored based on the branches inferred by STREAM. (b) Visualization of cells by UMAP based on top ten MLLE components. Left, cells are colored based on the labels from original study. Right, cells are colored based on the branches inferred by STREAM. (c,d) Subway map trajectory visualization plots by STREAM in two-dimension and ten-dimension space respectively. (e,f) Stream trajectory visualization plots by STREAM in two-dimension and ten-dimension space respectively.

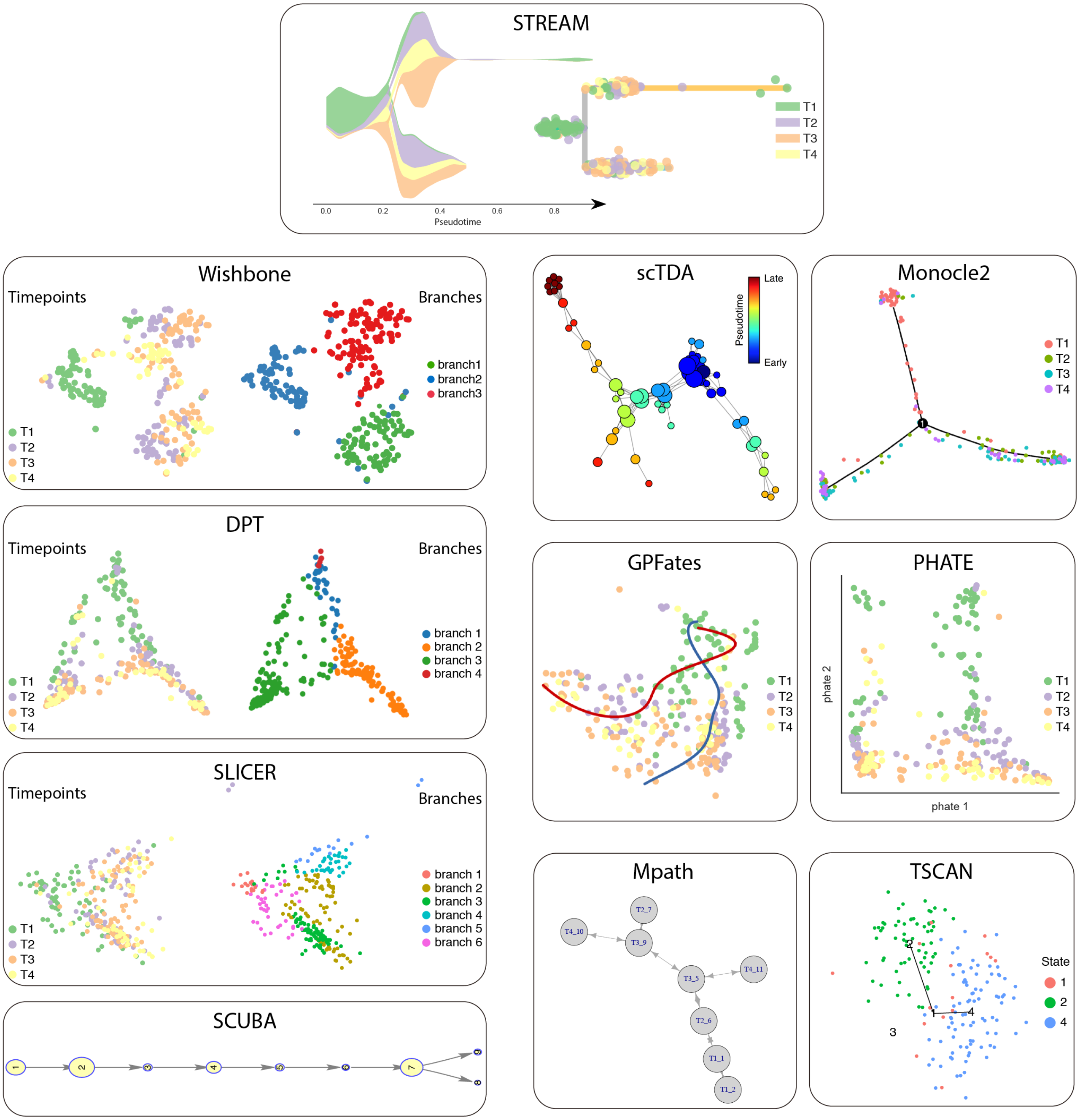

Supplementary Figure 7 Output of different trajectories inference methods on HSMM scRNA-seq data. Output provided by different methods on HSMM scRNA-seq data, as described in Figure 4c.

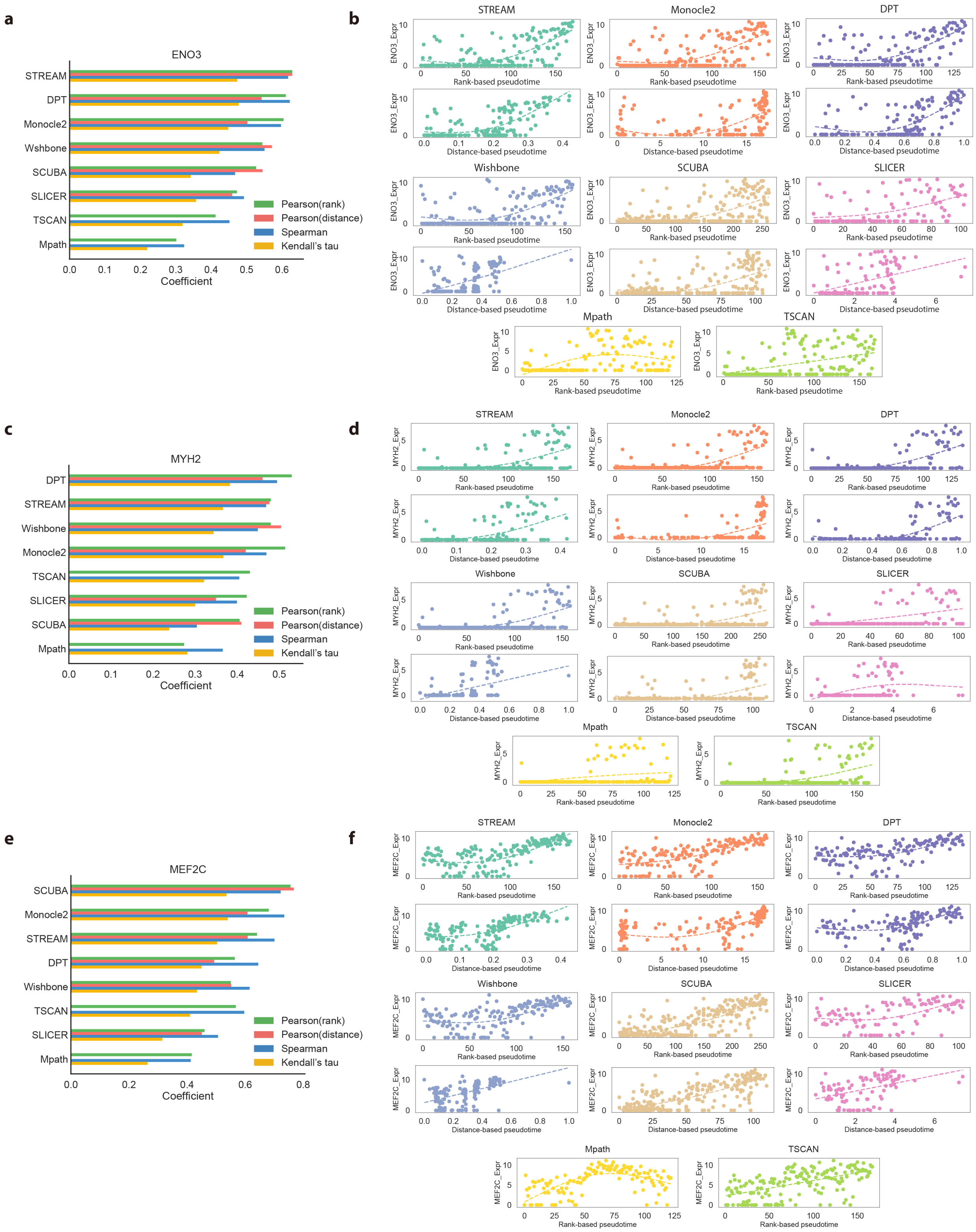

Supplementary Figure 8 Analysis results of different methods on HSMM scRNA-seq data. Same analysis as presented in Figure 5b-c for all three marker genes of myoblast differentiation, i.e., ENO3, MYH2 and MEF2C, and for all methods. (Only methods that successfully detect the correct bifurcation are included). (a-b) Correlation analysis and scatter plots between inferred distance-based or rank-based pseudotime and *ENO3* expression along myoblast differentiation. (c-d) Correlation analysis and scatter plots between inferred distance-based or rank-based pseudotime and *MYH2* expression along myoblast differentiation. (e-f) Correlation analysis and scatter plots between inferred distance-based or rank-based pseudotime and *MEF2C* expression along myoblast differentiation.

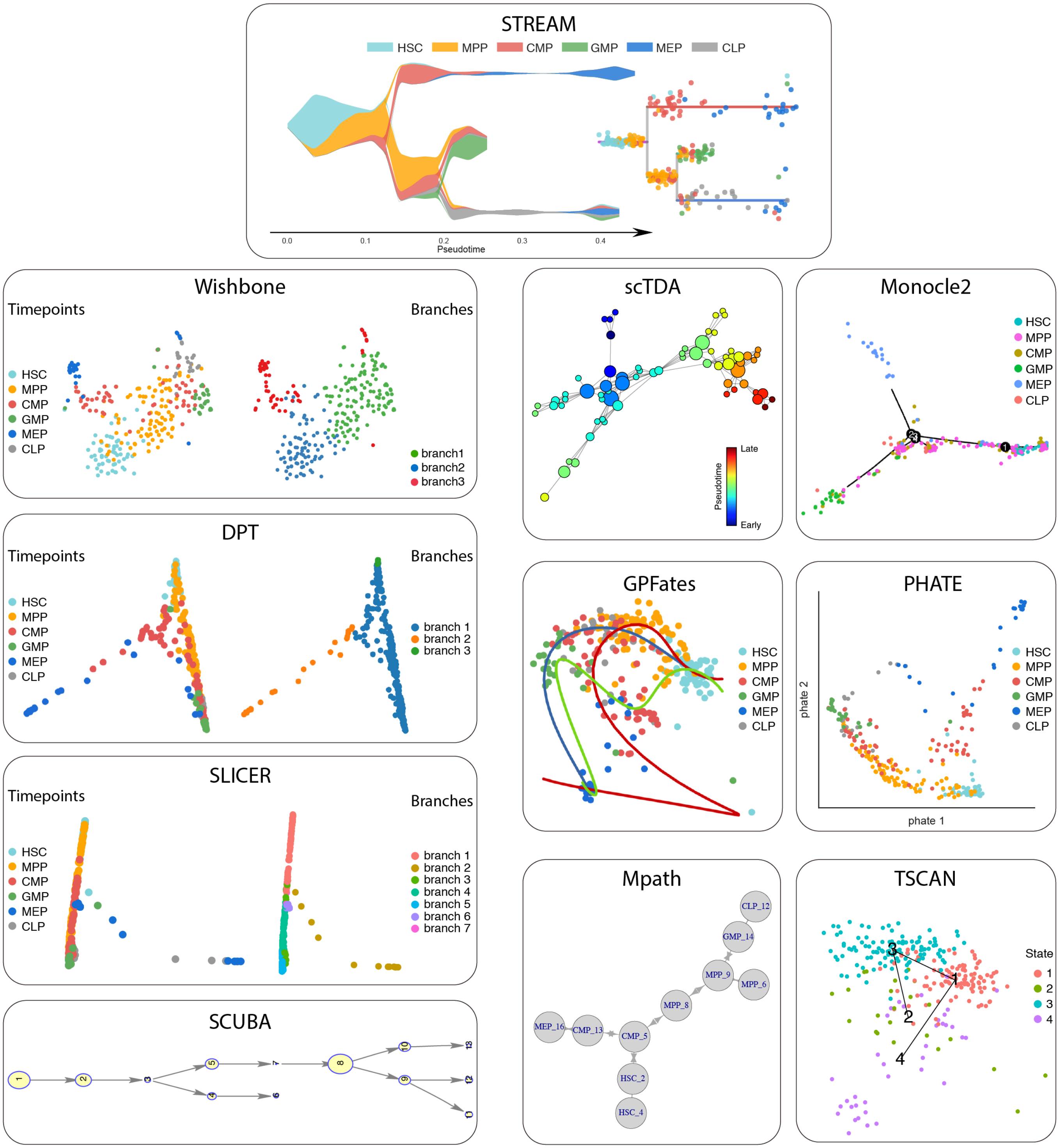

Supplementary Figure 9 Output of different trajectories inference methods on mouse hematopoietic sc-qPCR data. Output provided by different methods on mouse hematopoietic sc-qPCR data, as described in Figure 4c.

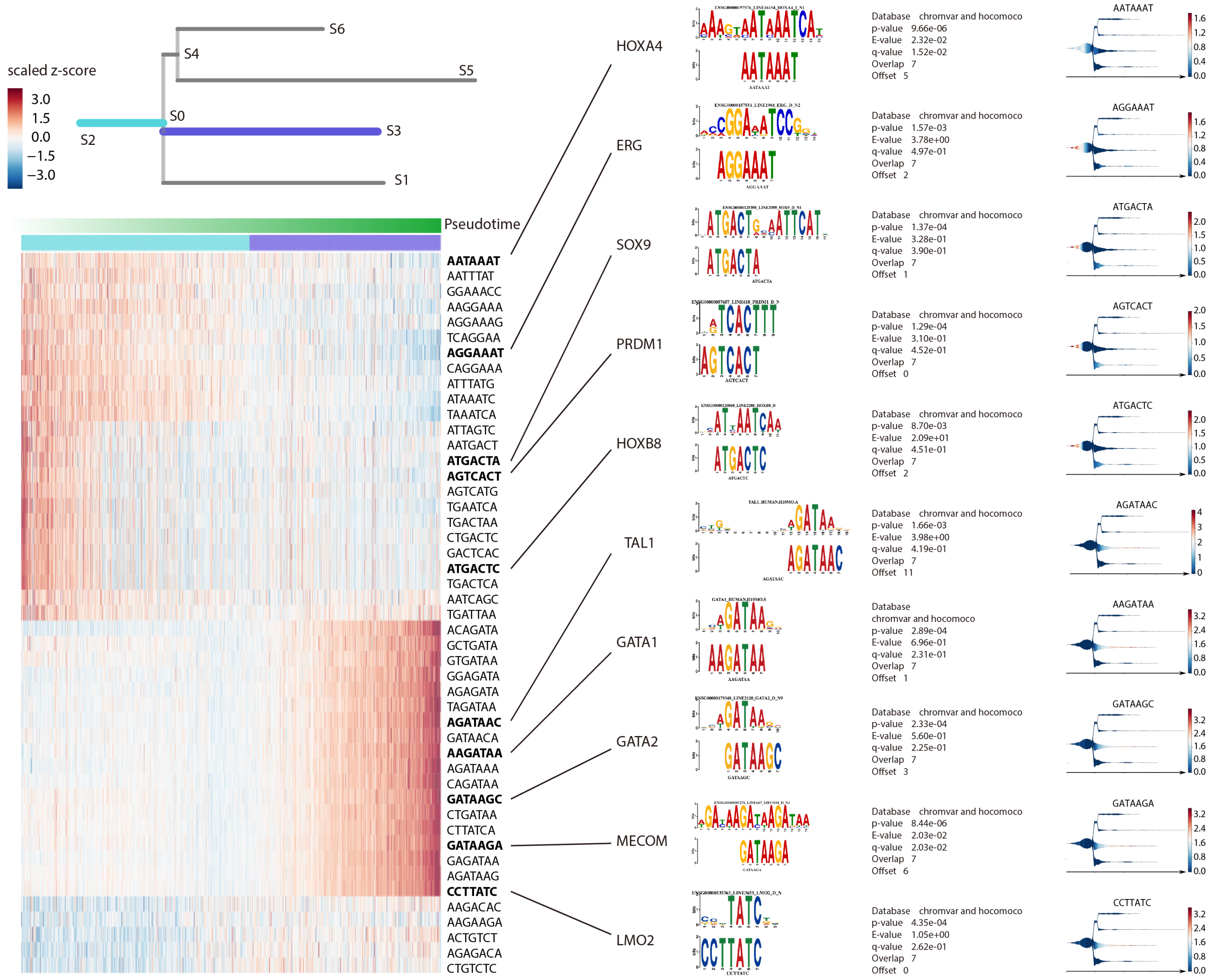

Supplementary Figure 10 STREAM automatically discovers important k-mers along the erythroid cells differentiation trajectory for the human hematopoietic system. Top left, subway map schematic to highlight the erythroid cells differentiation trajectory (S2,S0,S3) consisting of three branches (S2,S0)(cyan), (S0,S3)(purple), whose related marker k-mers are calculated. Bottom left, heatmap showing the standardized Z-scores of detected important k-mers. Each row indicates one k-mer. Each column indicates one cell on the trajectory (S2,S0,S3) and cells are ordered by the inferred pseudotime from STREAM. The two colors represent different cell branch ID assignments including branch (S2,S0) and branch(S0,S3). Right, ten detected k-mers selected from left heatmap and their target transcription factor motifs along with output information reported by the motif comparison tool Tomtom. These k-mers are further visualized on stream plots.

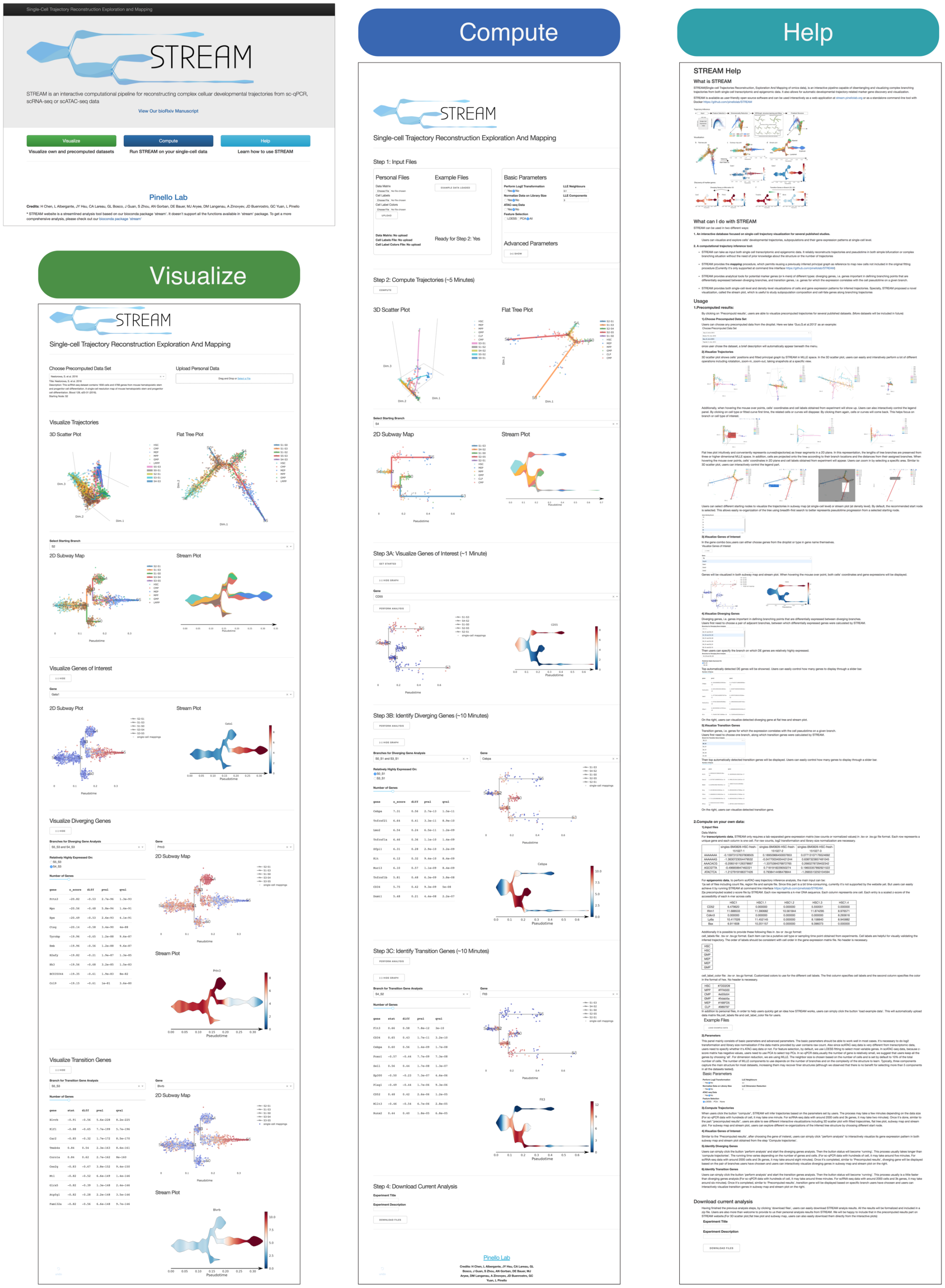

Supplementary Figure 11 STREAM interactive website. The website consists of three parts: ‘Visualize’, ‘Compute on Your Data’, and ‘Help’. In the ‘Visualize’’ page, users can visualize single cell trajectories from several published datasets or import the STREAM report zip file from their own STREAM analysis. In the ‘Compute’ page users can infer and visualize trajectories using STREAM using their own scRNA-seq or scATAC-seq datasets. In the ‘Help’ page, users can easily learn how to use STREAM website with the assistance of a detailed guide.

Supplementary Table1 Overview of trajectories inference methods included in the comparison

Features	SCUBA	TSCAN	Monocle2	Wishbone	Mpath	scTDA	SLICER	DPT	GPFates	PHATE	STREAM
Structure	Multiple branches	Linear	Multiple branches	Simple bifurcation	Multiple branches	Multiple branches	Multiple branches	Multiple branches	Multiple branches	Multiple branches	Multiple branches
Deterministic	✘	✓	✓	✘	✓	✘	✓	✓	✘	✓	✓
Ease of installation	+	++	++	+	+	++	++	++	+	++	++
Extra-input	Time points	None	None	Starting cell	Starting Cell and time points	Time points	Starting cell	Starting Cell and the number of branchings	Times points and number of trends	None	None
Explicit trajectory visualization	✓	✓	✓	✘	✓	✓	✘	✘	✓	✘	✓
Single-cell-level trajectory visualization	✘	✓	✓	✘	✘	✘	✘	✘	✓	✘	✓
Density level trajectory visualization	✘	✘	✘	✘	✘	✘	✘	✘	✘	✘	✓
Automatic marker gene detection	✘	✓	✓	✘	✓	✘	✘	✘	✘	✘	✓
The mapping feature	✘	✘	✘	✘	✘	✘	✘	✘	✘	✘	✓
Tested on epigenomic data	✘	✘	✓	✘	✘	✘	✘	✘	✘	✘	✓
End-to-end pipeline for scATAC-seq	✘	✘	✘	✘	✘	✘	✘	✘	✘	✘	✓

Summary table to compare features available in different methods.

Supplementary Table2 Dataset summary and execution time of trajectories inference methods included in the comparison

Method	Datasets used in the original study			Execution time (seconds)[4]		
	Technique	#Cells	Organism	sc-qPCR (271cells,175genes)	scRNA-seq (271 cells,47192 genes)	Synthetic (700 cells,500 genes)
**STREAM**	scRNA-seq	1656	Mouse	23.02	28.05	81.65
	scRNA-seq	382	Mouse			
	sc-qPCR	271	Mouse			
	MARS-seq	2699	Mouse			
	sc-qPCR	168	Zebrafish			
	inDrop	9628	Zebrafish			
	scRNA-seq	271	Human			
	scATAC-seq	2034	Human			
**Monocle2**	scRNA-seq	271	Human	26.46	88.03	67.42
	scRNA-seq	183	Mouse			
	MARS-seq	2699	Mouse			
	scRNA-seq	382	Mouse			
**Wishbone**	Mass cytometry	Replicate1: 201916<br>Replicate2: 178420<br>Replicate3: 156187	Mouse	12.37	42.77	34.14
	Mass cytometry	22850	Human			
	Mass cytometry	19813	Human			
	scRNA-seq	4423	Mouse			
**Mpath[1]**	scRNA-seq	251	Mouse	28.33	3942.24	117
	scRNA-seq	372	Human			
**scTDA**	scRNA-seq	373	Mouse	21.6	28.18	50.33
	scRNA-seq	1963	Mouse			
	scRNA-seq	80	Mouse			
	scRNA-seq	1529	Human			
	scRNA-seq	272	Mouse			
**SLICER**	scRNA-seq	183	Mouse	49.12	168.51	359.08
	scRNA-seq	271	Mouse			
**DPT**	sc-qPCR	3934	Mouse	4.02	6.02	4.05
	inDrop	2717	Mouse			
	MARS-seq	2730	Mouse			
**GPFates**	scRNA-seq	408	Mouse	22.6	21.38	91.47
	scRNA-seq	183	Mouse			
	scRNA-seq	233	Human			
	RNA-seq	193	Frog			
**PHATE[2]**	scRNA-seq	2730	Mouse	4.26	5.27	5.11
	scRNA-seq	6174	Mouse			
	scRNA-seq	3005	Mouse			
	scRNA-seq	16825	Human			
	Mass Cytometry	220450	Mouse			
**SCUBA**	RT-PCR	438	Mouse	10.75	16.73	22.03
	scRNA-seq	294	Mouse			
	Mass cytometry	~20K	Human			
**TSCAN[3]**	scRNA-seq	271	Human	~30	~60	~40
	scRNA-seq	306	Mouse			
	scRNA-seq	172	Mouse			

1.For Mpath, when analyzing scRNA, it needs to run ANOVA test to identify differentially expressed genes to build networks. So it takes more time than the other methods

2.For PHATE, it's a general visualization method and explores different types of datasets. Here we only consider biological datasets

3.For TSCAN, the execution time is approximated and based on the trajectory analysis using the web interface (we timed and excluded the user interactions)

4.The test platform information: Linux version 3.10.0-693.17.1.el7.x86_64, Architecture: x86_64 CPU op-mode(s): 32-bit, 64-bit, Byte Order: Little Endian, CPU(s): 44, On-line CPU(s) list: 0-43, Thread(s) per core: 1, Core(s) per socket: 22, Model name: Intel(R) Xeon(R) CPU E5-2699 v4 @ 2.20GHz, CPU MHz: 3523.351, RAM: 1.0T

Supplementary References

1. Nestorowa S*, et al.* A single-cell resolution map of mouse hematopoietic stem and progenitor cell differentiation. *Blood* **128**, e20-31 (2016).

2. Kurz T, Weiner M, Skoglund C, Basnet S, Eriksson P, Segelmark M. A myelopoiesis gene signature during remission in anti-neutrophil cytoplasm antibody-associated vasculitis does not predict relapses but seems to reflect ongoing prednisolone therapy. *Clin Exp Immunol* **175**, 215-226 (2014).

3. Paul F*, et al.* Transcriptional Heterogeneity and Lineage Commitment in Myeloid Progenitors. *Cell* **163**, 1663-1677 (2015).

4. Johnson KD*, et al.* Cis-regulatory mechanisms governing stem and progenitor cell transitions. *Sci Adv* **1**, e1500503 (2015).

5. Ryu YK, Lee JW, Moon EY. Thymosin Beta-4, Actin-Sequestering Protein Regulates Vascular Endothelial Growth Factor Expression via Hypoxia-Inducible Nitric Oxide Production in HeLa Cervical Cancer Cells. *Biomol Ther (Seoul)* **23**, 19-25 (2015).

6. Pick R*, et al.* Coronin 1A, a novel player in integrin biology, controls neutrophil trafficking in innate immunity. *Blood* **130**, 847-858 (2017).

7. Wu S*, et al.* BLVRB redox mutation defines heme degradation in a metabolic pathway of enhanced thrombopoiesis in humans. *Blood* **128**, 699-709 (2016).

8. Behrens K*, et al.* Runx1 downregulates stem cell and megakaryocytic transcription programs that support niche interactions. *Blood* **127**, 3369-3381 (2016).

9. Gilliland DG, Griffin JD. The roles of FLT3 in hematopoiesis and leukemia. *Blood* **100**, 1532-1542 (2002).

10. Jung H*, et al.* Thioredoxin-interacting protein regulates haematopoietic stem cell ageing and rejuvenation by inhibiting p38 kinase activity. *Nature communications* **7**, 13674 (2016).

11. Silberstein L*, et al.* Embigin Regulates HSPC Homing and Quiescence and Acts As a Cell Surface Marker for a Niche Factor-Enriched Subset of Osteolineage Cells. *Blood* **126**, 663 (2015).

12. Seita J, Weissman IL. Hematopoietic stem cell: self-renewal versus differentiation. *Wiley Interdiscip Rev Syst Biol Med* **2**, 640-653 (2010).

13. Forsberg EC, Prohaska SS, Katzman S, Heffner GC, Stuart JM, Weissman IL. Differential expression of novel potential regulators in hematopoietic stem cells. *PLoS Genet* **1**, e28 (2005).

14. Tang Q*, et al.* Dissecting hematopoietic and renal cell heterogeneity in adult zebrafish at single-cell resolution using RNA sequencing. *J Exp Med* **214**, 2875-2887 (2017).

15. Hong C-W. Current understanding in neutrophil differentiation and heterogeneity. *Immune network* **17**, 298-306 (2017).

16. Cochain C*, et al.* Single-cell RNA-seq reveals the transcriptional landscape and heterogeneity of aortic macrophages in murine atherosclerosis. *Circ Res* **122**, 1661-1674 (2018).

17. Bonnardel J, Guilliams M. Developmental control of macrophage function. *Current opinion in immunology* **50**, 64-74 (2018).

18. Kiss M, Van Gassen S, Movahedi K, Saeys Y, Laoui D. Myeloid cell heterogeneity in cancer: not a single cell alike. *Cellular immunology* **330**, 188-201 (2018).

19. Lu X-J, Chen Q, Rong Y-J, Chen F, Chen J. CXCR3. 1 and CXCR3. 2 differentially contribute to macrophage polarization in teleost fish. *J Immunol* **198**, 4692-4706 (2017).

20. Nguyen-Chi M*, et al.* Identification of polarized macrophage subsets in zebrafish. *Elife* **4**, e07288 (2015).

21. Qiu X*, et al.* Reversed graph embedding resolves complex single-cell trajectories. *Nat Methods* **14**, 979-982 (2017).

22. Chen J, Schlitzer A, Chakarov S, Ginhoux F, Poidinger M. Mpath maps multi-branching single-cell trajectories revealing progenitor cell progression during development. *Nat Commun* **7**, 11988 (2016).

23. Setty M*, et al.* Wishbone identifies bifurcating developmental trajectories from single-cell data. *Nat Biotechnol* **34**, 637-645 (2016).

24. Haghverdi L, Buttner M, Wolf FA, Buettner F, Theis FJ. Diffusion pseudotime robustly reconstructs lineage branching. *Nat Methods* **13**, 845-848 (2016).

25. Welch JD, Hartemink AJ, Prins JF. SLICER: inferring branched, nonlinear cellular trajectories from single cell RNA-seq data. *Genome Biol* **17**, 106 (2016).

26. Rizvi AH*, et al.* Single-cell topological RNA-seq analysis reveals insights into cellular differentiation and development. *Nat Biotechnol* **35**, 551-560 (2017).

27. Singh G, Mémoli F, Carlsson GE. Topological methods for the analysis of high dimensional data sets and 3d object recognition. *SPBG*, 91-100 (2007).

28. Ji Z, Ji H. TSCAN: Pseudo-time reconstruction and evaluation in single-cell RNA-seq analysis. *Nucleic Acids Res* **44**, e117 (2016).

29. Marco E*, et al.* Bifurcation analysis of single-cell gene expression data reveals epigenetic landscape. *Proc Natl Acad Sci U S A* **111**, E5643-5650 (2014).

30. Lonnberg T*, et al.* Single-cell RNA-seq and computational analysis using temporal mixture modelling resolves Th1/Tfh fate bifurcation in malaria. *Science immunology* **2**, eaal2192 (2017).

31. Moon KR*, et al.* Visualizing Structure and Transitions for Biological Data Exploration. Preprint at https://www.biorxiv.org/content/10.1101/120378v3 (2018).

32. Olsson A*, et al.* Single-cell analysis of mixed-lineage states leading to a binary cell fate choice. *Nature* **537**, 698-702 (2016).

33. Moore FE*, et al.* Single-cell transcriptional analysis of normal, aberrant, and malignant hematopoiesis in zebrafish. *J Exp Med* **213**, 979-992 (2016).

34. Trapnell C*, et al.* The dynamics and regulators of cell fate decisions are revealed by pseudotemporal ordering of single cells. *Nat Biotechnol* **32**, 381-386 (2014).

35. Guo G*, et al.* Mapping cellular hierarchy by single-cell analysis of the cell surface repertoire. *Cell Stem Cell* **13**, 492-505 (2013).

36. Buenrostro JD*, et al.* Integrated Single-Cell Analysis Maps the Continuous Regulatory Landscape of Human Hematopoietic Differentiation. *Cell* **173**, 1535-1548 (2018).

37. Grüning B*, et al.* Bioconda: sustainable and comprehensive software distribution for the life sciences. *Nature methods* **15**, 475 (2018).
