# Supplementary material for: Single-cell trajectories reconstruction, exploration and mapping of omics data with STREAM
Source: Nat Commun. 2019 Apr 23;10:1903. doi: 10.1038/s41467-019-09670-4 (PMC6478907; doi:10.1038/s41467-019-09670-4)
Supplement: Supplementary file 3 — Description of Additional Supplementary Files [file 41467_2019_9670_MOESM3_ESM.pdf]

Description of Additional Supplementary Files

File Name: Supplementary Data 1
Description: Datasets and Jupyter notebooks used to reproduce all the STREAM analysis.

File Name: Supplementary Data 2
Description: Datasets and Jupyter notebooks used to reproduce the analyses from other methods included in the method comparison section.
