# Supplementary figures and images for: Single-cell trajectories reconstruction, exploration and mapping of omics data with STREAM
Source: Nat Commun. 2019 Apr 23;10:1903. doi: 10.1038/s41467-019-09670-4 (PMC6478907; doi:10.1038/s41467-019-09670-4)

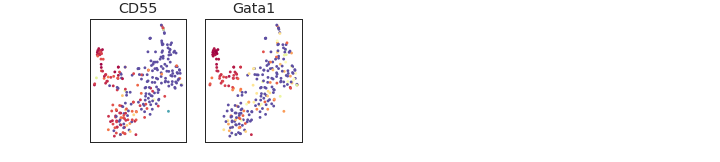

Supplement: Supplementary file 5 — Supplementary Data 2 [file 41467_2019_9670_MOESM5_ESM.zip › Sup_data2/Guo_2013/wishbone/tSNE_by_gene.png]

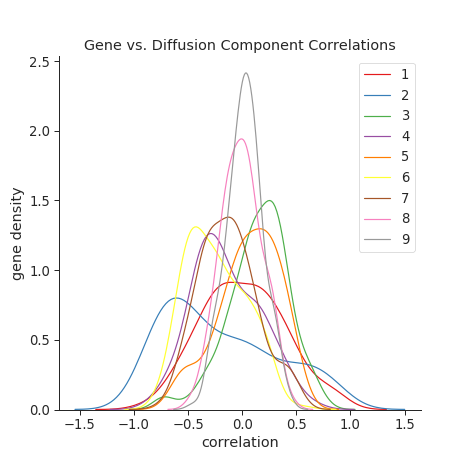

Supplement: Supplementary file 5 — Supplementary Data 2 [file 41467_2019_9670_MOESM5_ESM.zip › Sup_data2/Guo_2013/wishbone/gene_component_correlations.png]

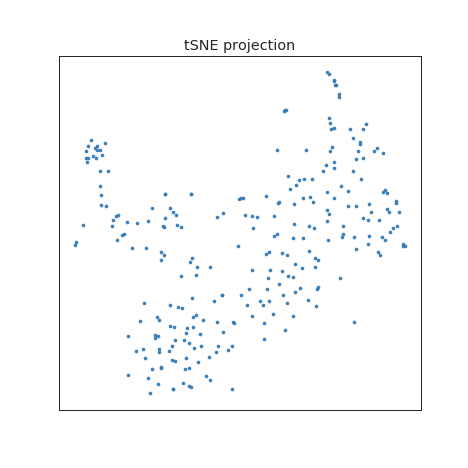

Supplement: Supplementary file 5 — Supplementary Data 2 [file 41467_2019_9670_MOESM5_ESM.zip › Sup_data2/Guo_2013/wishbone/tSNE.png]

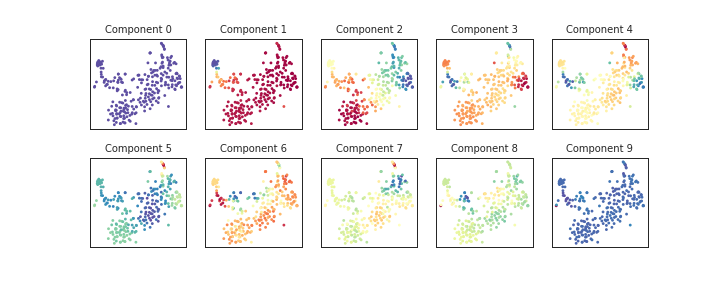

Supplement: Supplementary file 5 — Supplementary Data 2 [file 41467_2019_9670_MOESM5_ESM.zip › Sup_data2/Guo_2013/wishbone/diffusion_components.png]

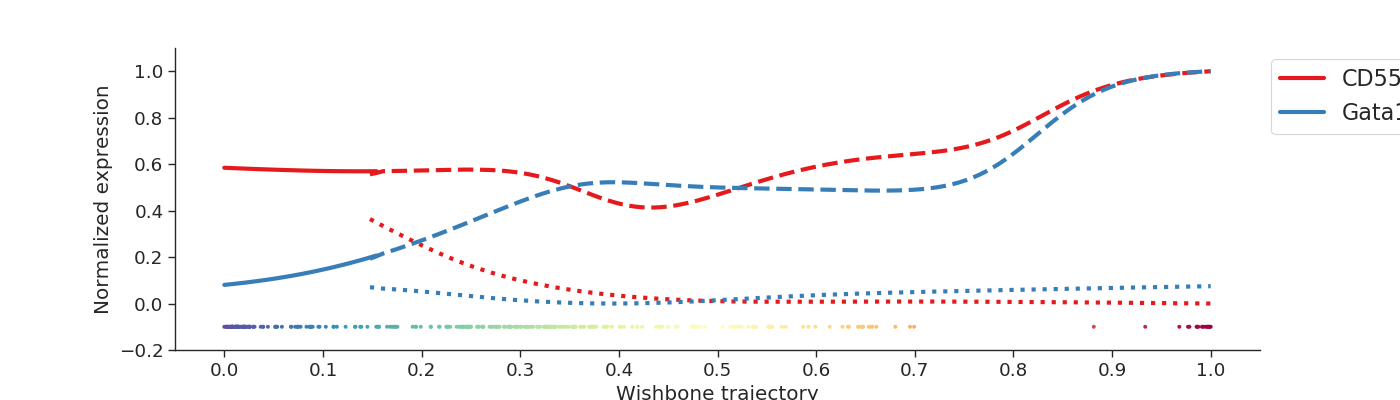

Supplement: Supplementary file 5 — Supplementary Data 2 [file 41467_2019_9670_MOESM5_ESM.zip › Sup_data2/Guo_2013/wishbone/marker_trajectory_CD55_Gata1.png]

Wishbone trajectory

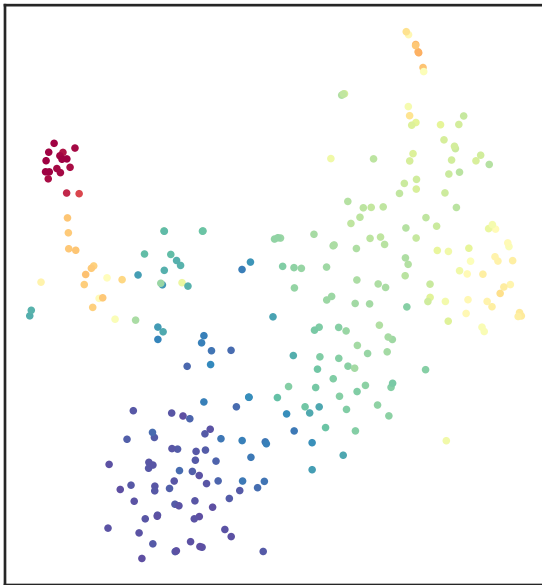

Branch associations

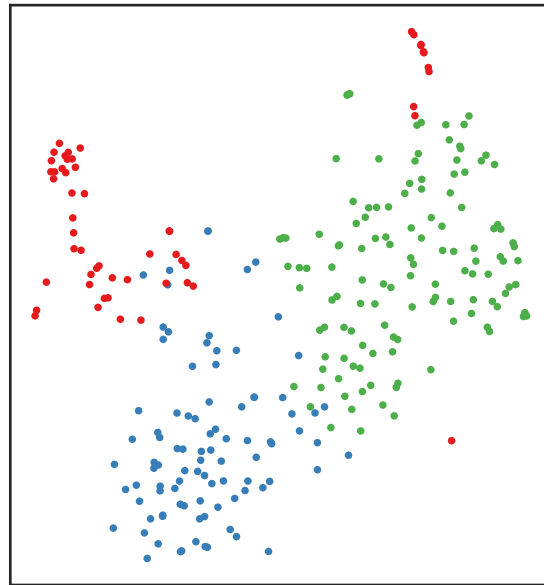

Supplement: Supplementary file 5 — Supplementary Data 2 [file 41467_2019_9670_MOESM5_ESM.zip › Sup_data2/Guo_2013/wishbone/wishbone_on_tsne.pdf]

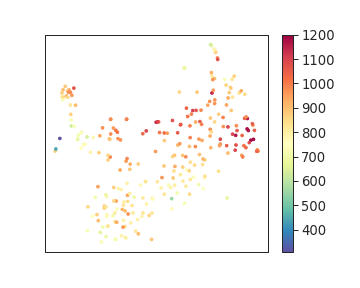

Supplement: Supplementary file 5 — Supplementary Data 2 [file 41467_2019_9670_MOESM5_ESM.zip › Sup_data2/Guo_2013/wishbone/tSNE_by_cell_sizes.png]

tSNE projection

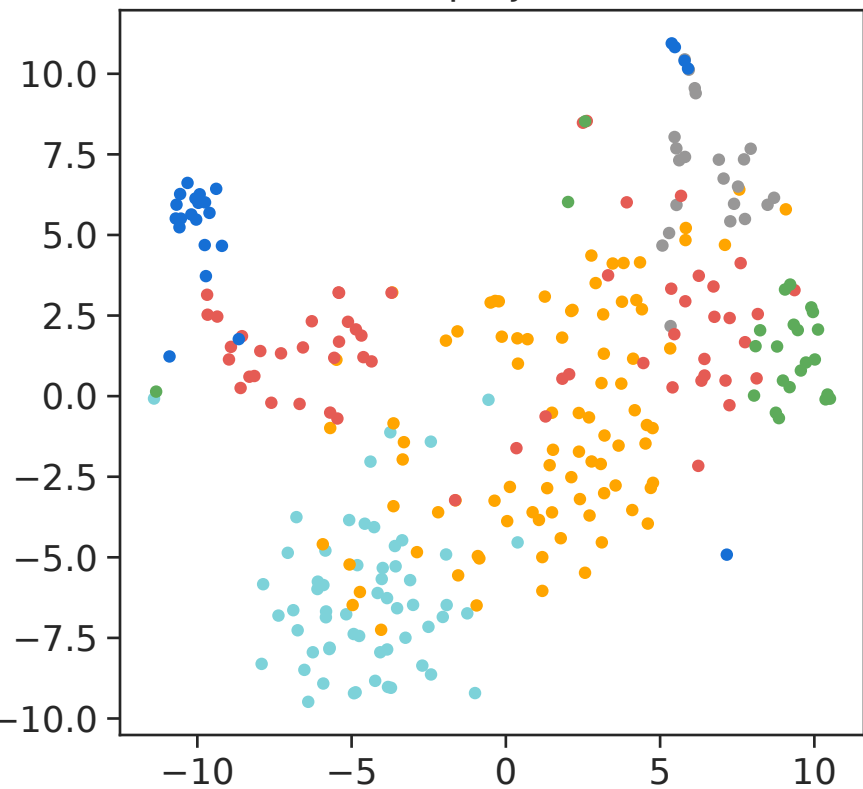

Supplement: Supplementary file 5 — Supplementary Data 2 [file 41467_2019_9670_MOESM5_ESM.zip › Sup_data2/Guo_2013/wishbone/tSNE_by_labels.pdf]

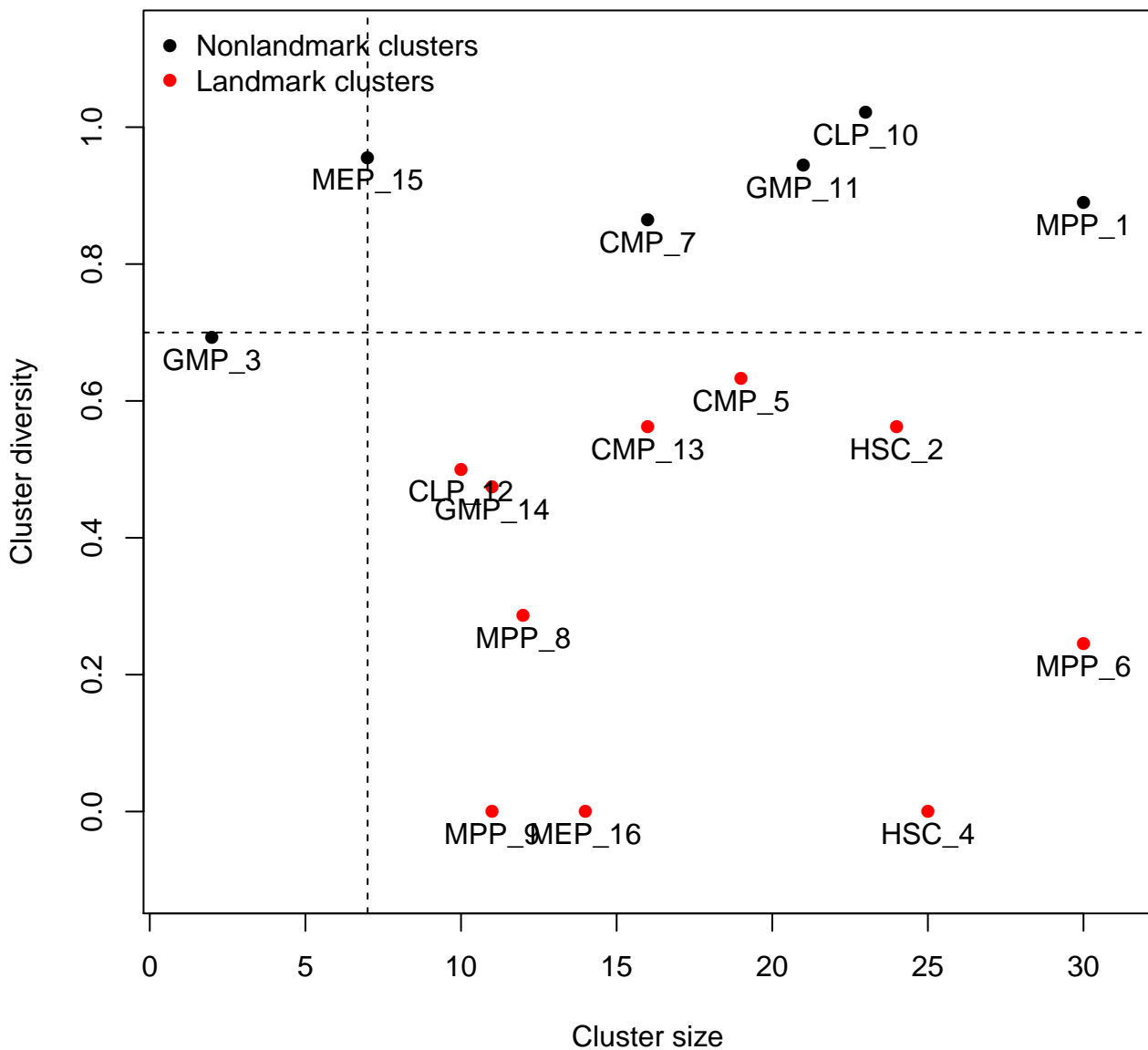

Supplement: Supplementary file 5 — Supplementary Data 2 [file 41467_2019_9670_MOESM5_ESM.zip › Sup_data2/Guo_2013/mpath/qPCR_rpkm_landmark_cluster.pdf]

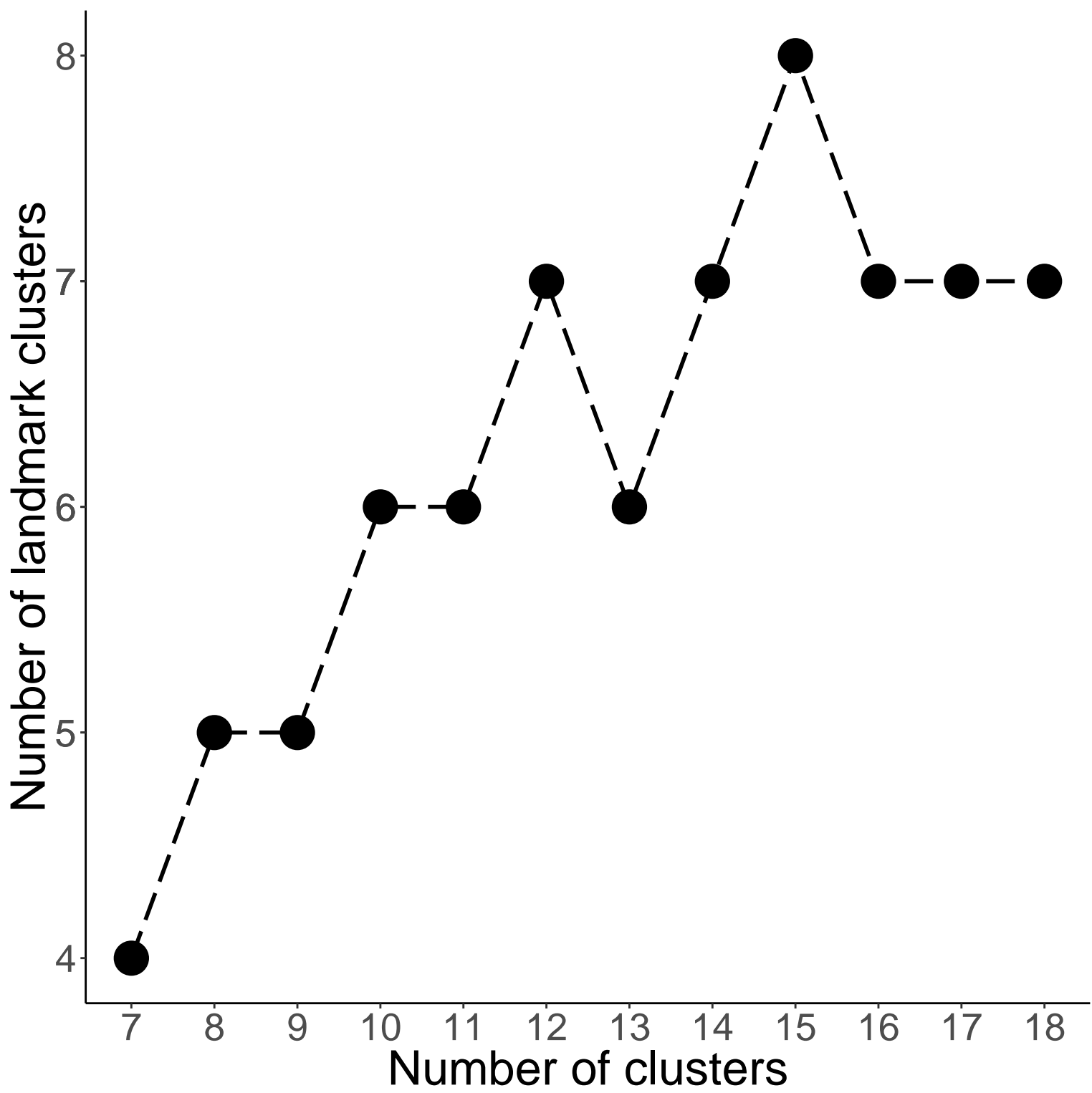

Supplement: Supplementary file 5 — Supplementary Data 2 [file 41467_2019_9670_MOESM5_ESM.zip › Sup_data2/Guo_2013/mpath/qPCR_rpkm_ncluster_vs_nlm.pdf]

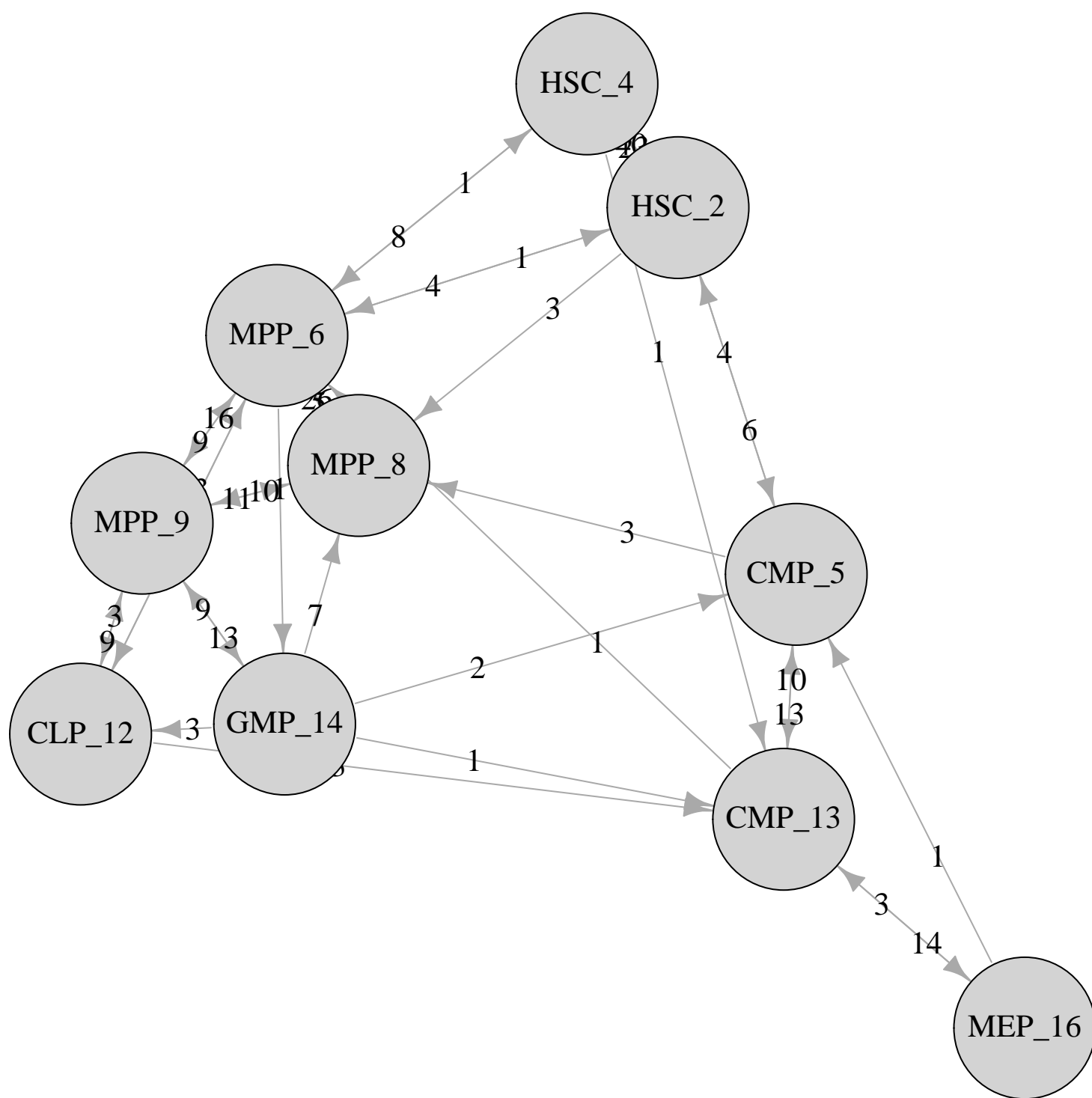

Supplement: Supplementary file 5 — Supplementary Data 2 [file 41467_2019_9670_MOESM5_ESM.zip › Sup_data2/Guo_2013/mpath/qPCR_rpkm_state_transition.pdf]

# CD55

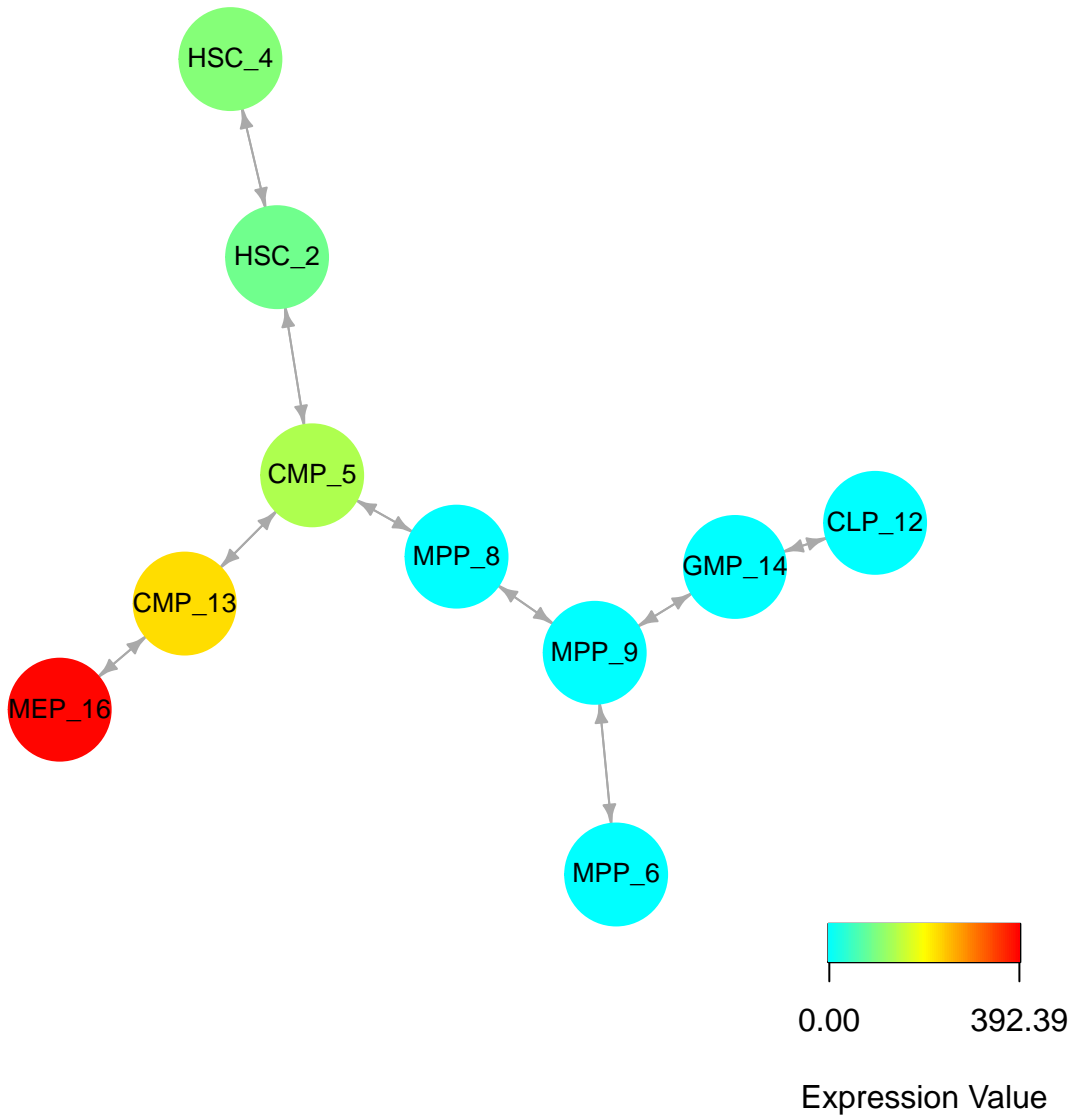

Supplement: Supplementary file 5 — Supplementary Data 2 [file 41467_2019_9670_MOESM5_ESM.zip › Sup_data2/Guo_2013/mpath/Marker_CD55.pdf]

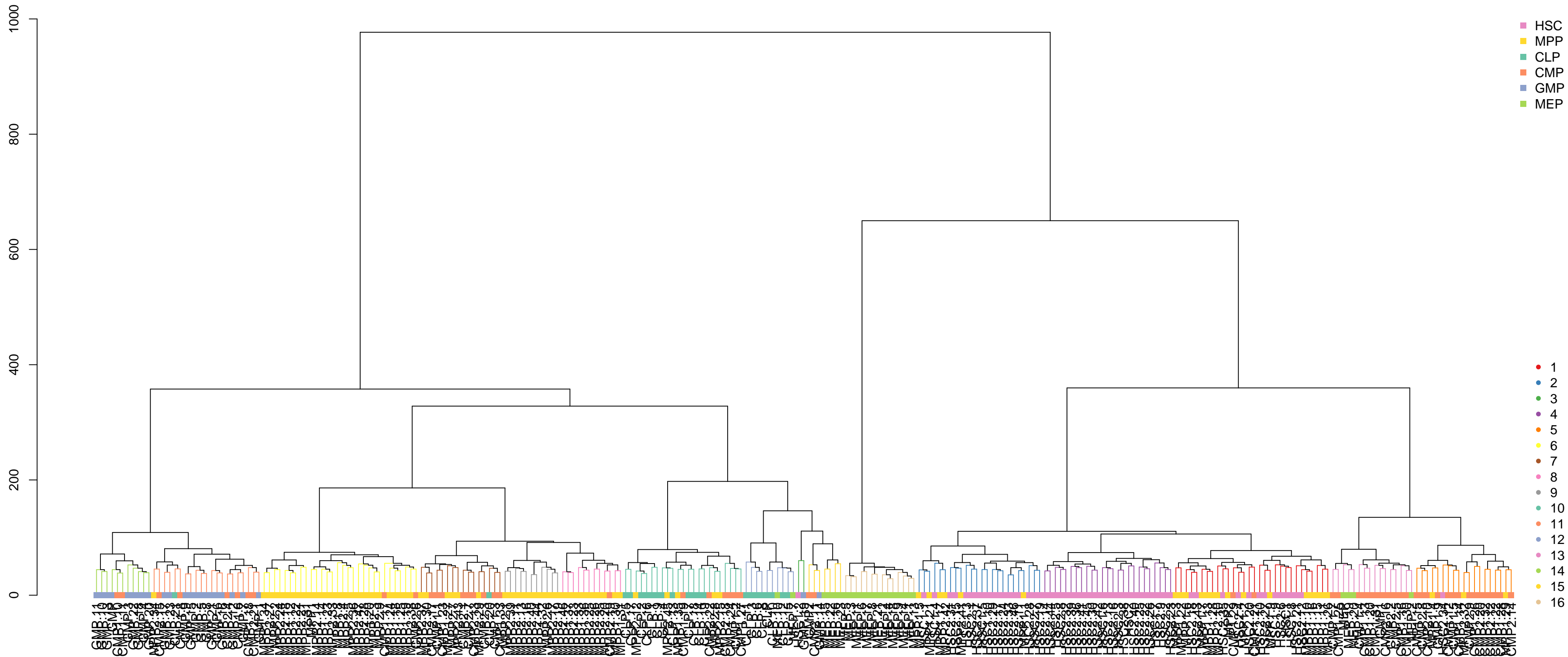

Supplement: Supplementary file 5 — Supplementary Data 2 [file 41467_2019_9670_MOESM5_ESM.zip › Sup_data2/Guo_2013/mpath/qPCR_rpkm_hc.pdf]

# Gata1

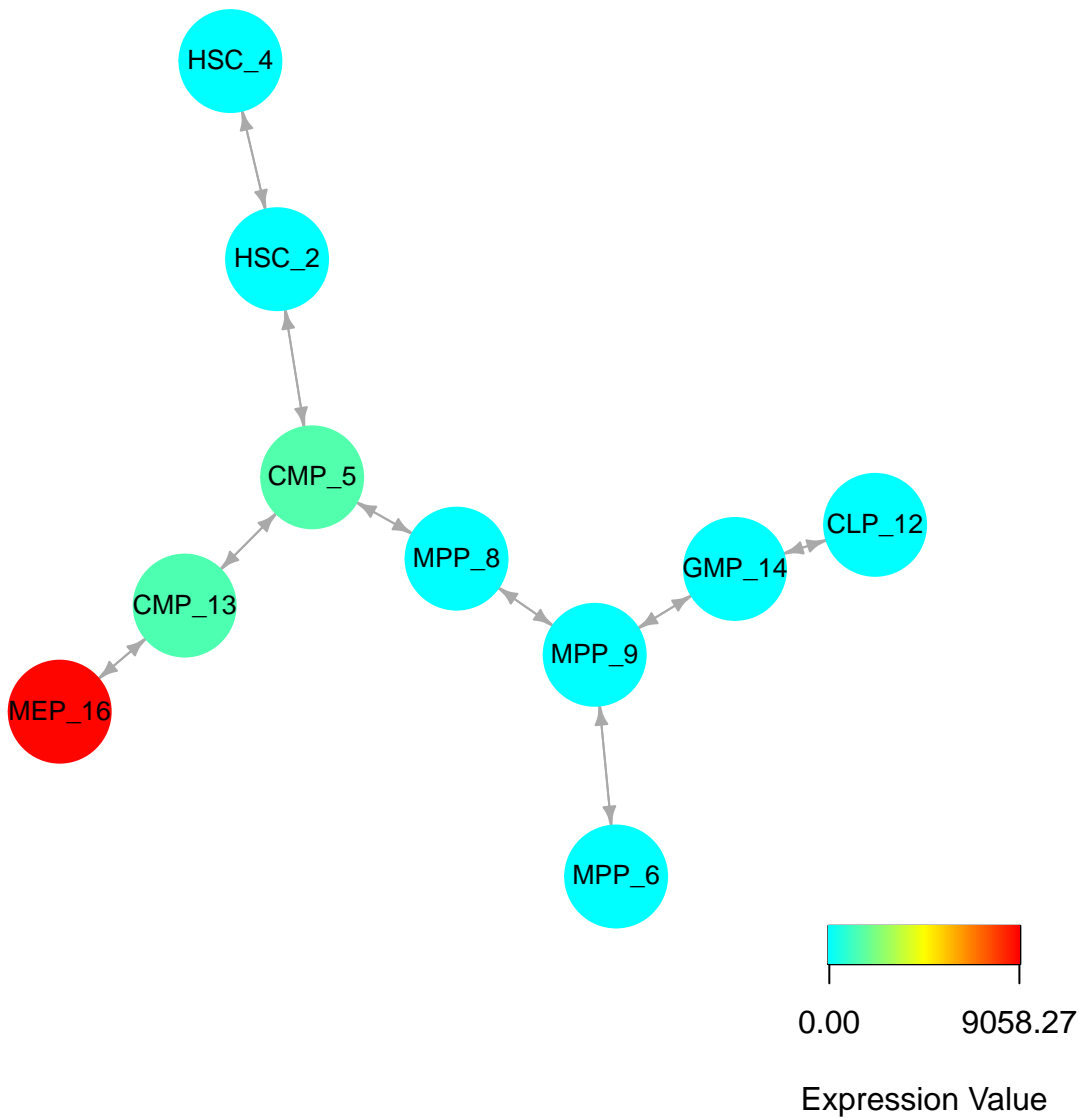

Supplement: Supplementary file 5 — Supplementary Data 2 [file 41467_2019_9670_MOESM5_ESM.zip › Sup_data2/Guo_2013/mpath/Marker_Gata1.pdf]

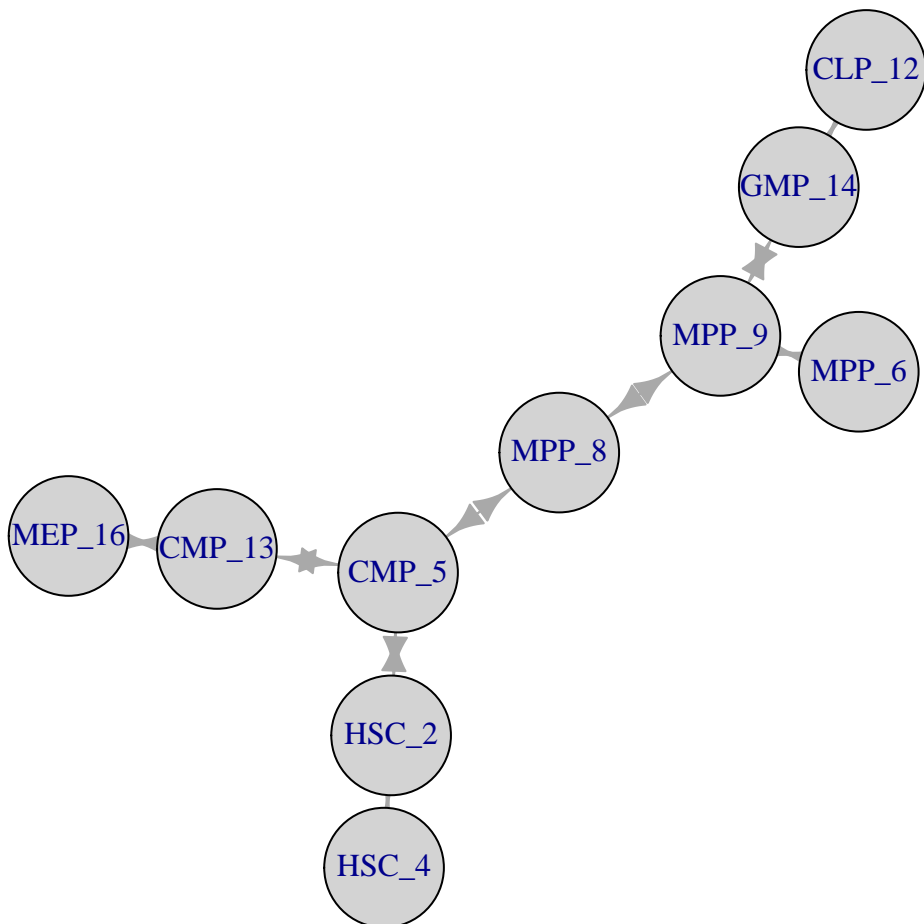

Supplement: Supplementary file 5 — Supplementary Data 2 [file 41467_2019_9670_MOESM5_ESM.zip › Sup_data2/Guo_2013/mpath/qPCR_rpkm_state_transition_mst.pdf]

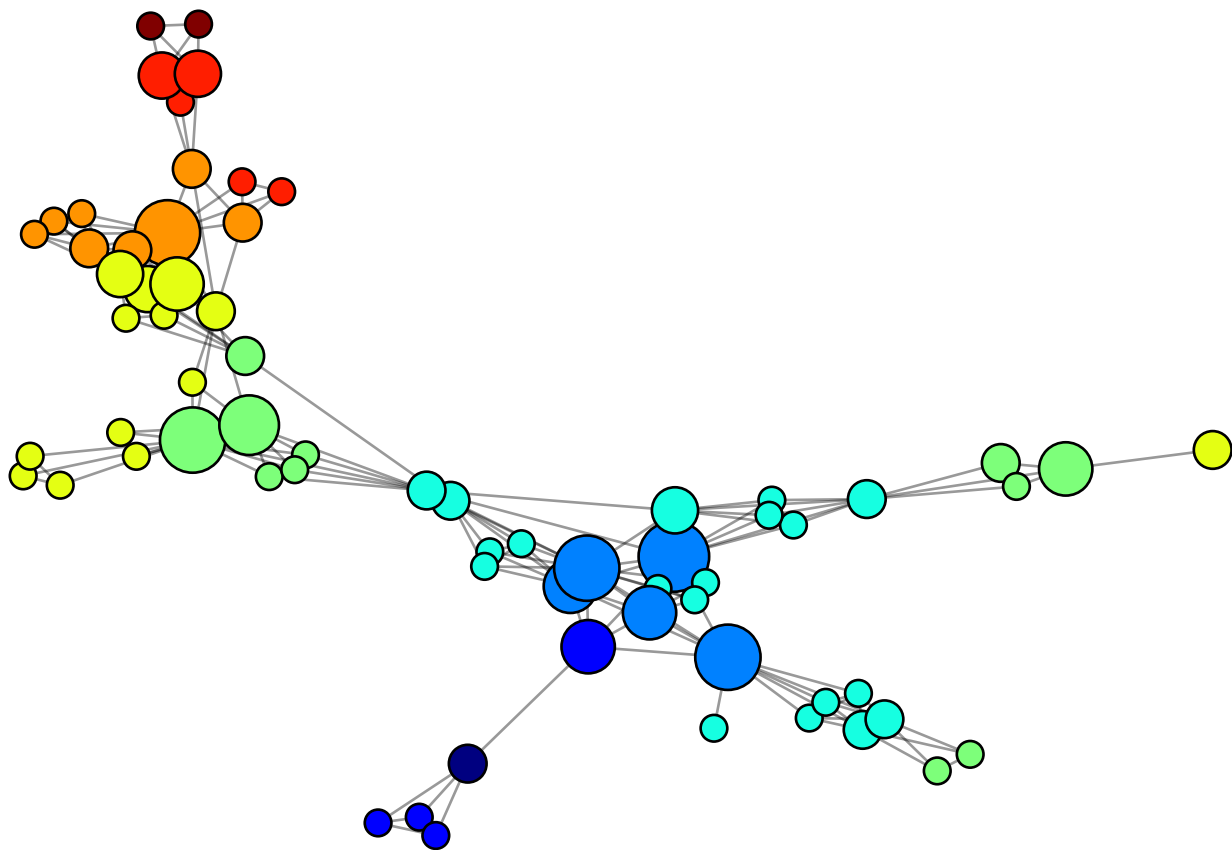

Supplement: Supplementary file 5 — Supplementary Data 2 [file 41467_2019_9670_MOESM5_ESM.zip › Sup_data2/Guo_2013/sctda/dist_root.pdf]

tSNE projection

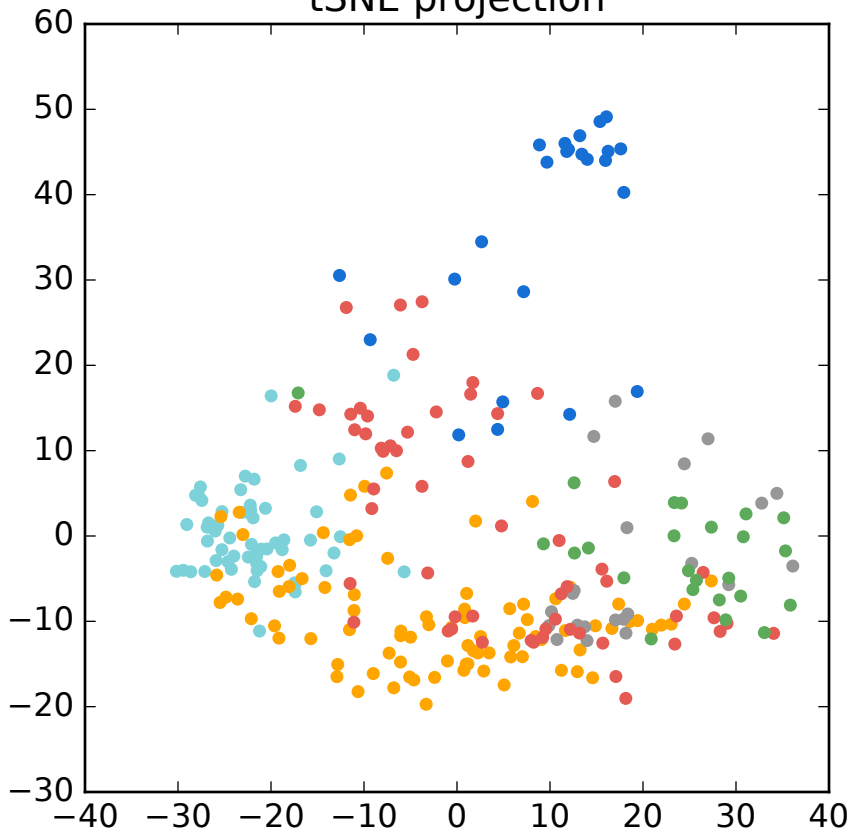

Supplement: Supplementary file 5 — Supplementary Data 2 [file 41467_2019_9670_MOESM5_ESM.zip › Sup_data2/Guo_2013/sctda/tSNE_by_labels.pdf]

State

|   |   |   |   |
|---|---|---|---|
| 1 | 4 | 7 | 9 |
| 2 | 5 | 8 |   |

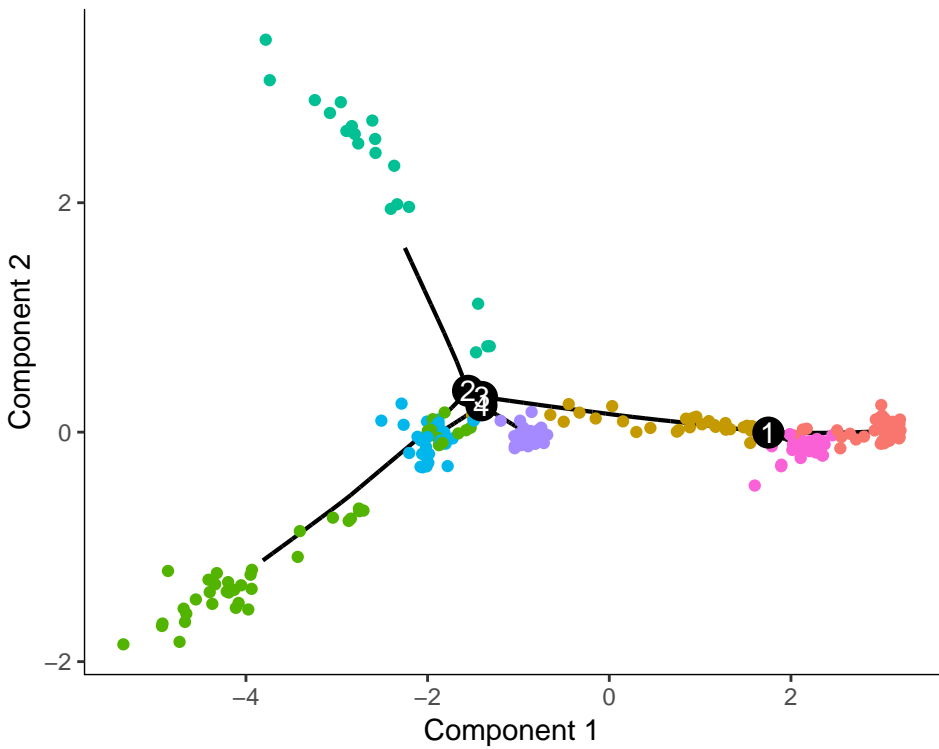

Supplement: Supplementary file 5 — Supplementary Data 2 [file 41467_2019_9670_MOESM5_ESM.zip › Sup_data2/Guo_2013/monocle2/monocle2_trajectory_by_state.pdf]

label

|     |     |     |
|-----|-----|-----|
| CLP | GMP | MEP |
| CMP | HSC | MPP |

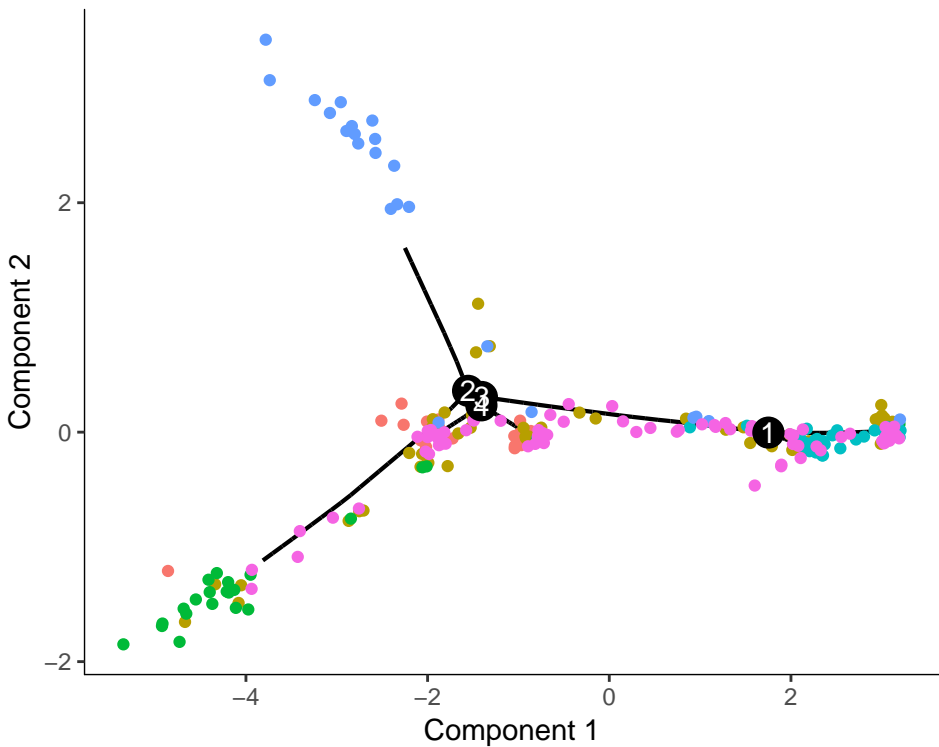

Supplement: Supplementary file 5 — Supplementary Data 2 [file 41467_2019_9670_MOESM5_ESM.zip › Sup_data2/Guo_2013/monocle2/monocle2_trajectory_by_time.pdf]

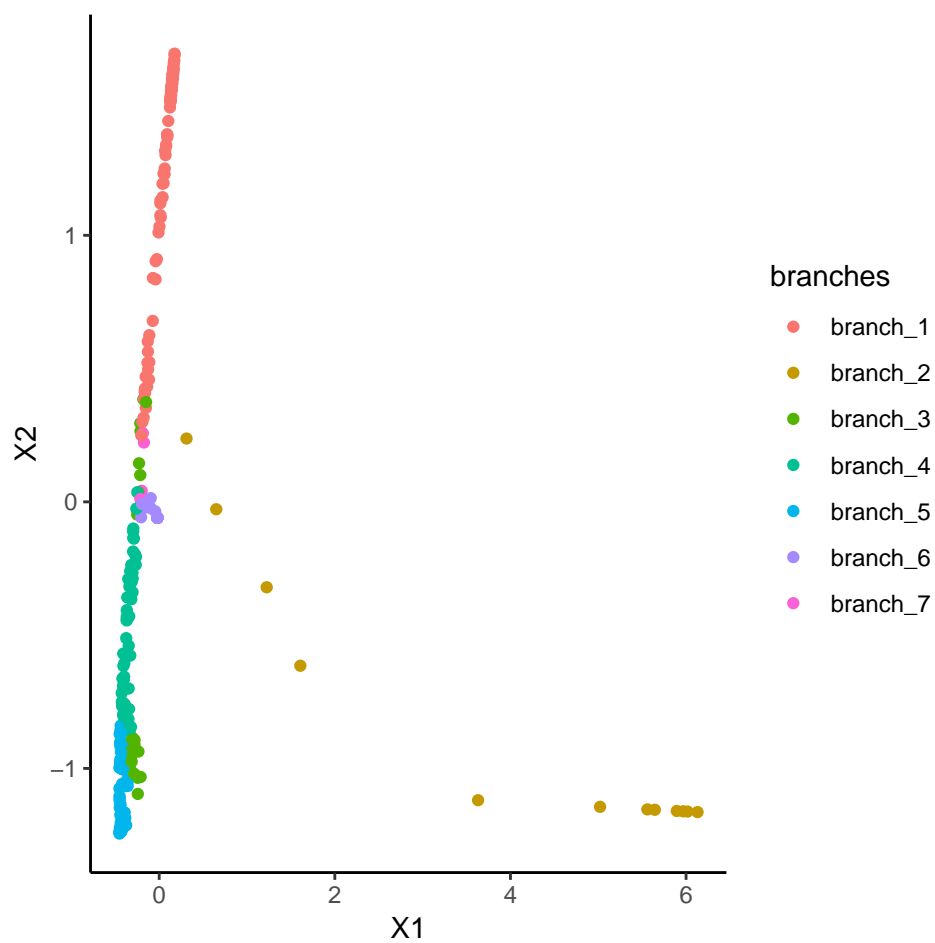

Supplement: Supplementary file 5 — Supplementary Data 2 [file 41467_2019_9670_MOESM5_ESM.zip › Sup_data2/Guo_2013/slicer/slicer_trajectory_by_branch.pdf]

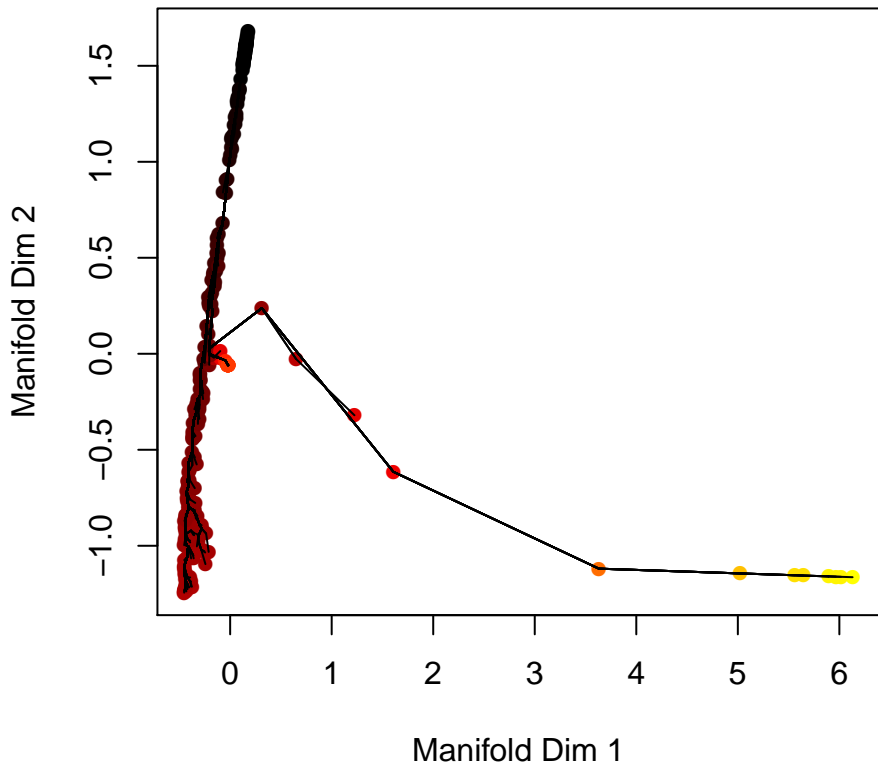

Supplement: Supplementary file 5 — Supplementary Data 2 [file 41467_2019_9670_MOESM5_ESM.zip › Sup_data2/Guo_2013/slicer/slicer_trajectory.pdf]

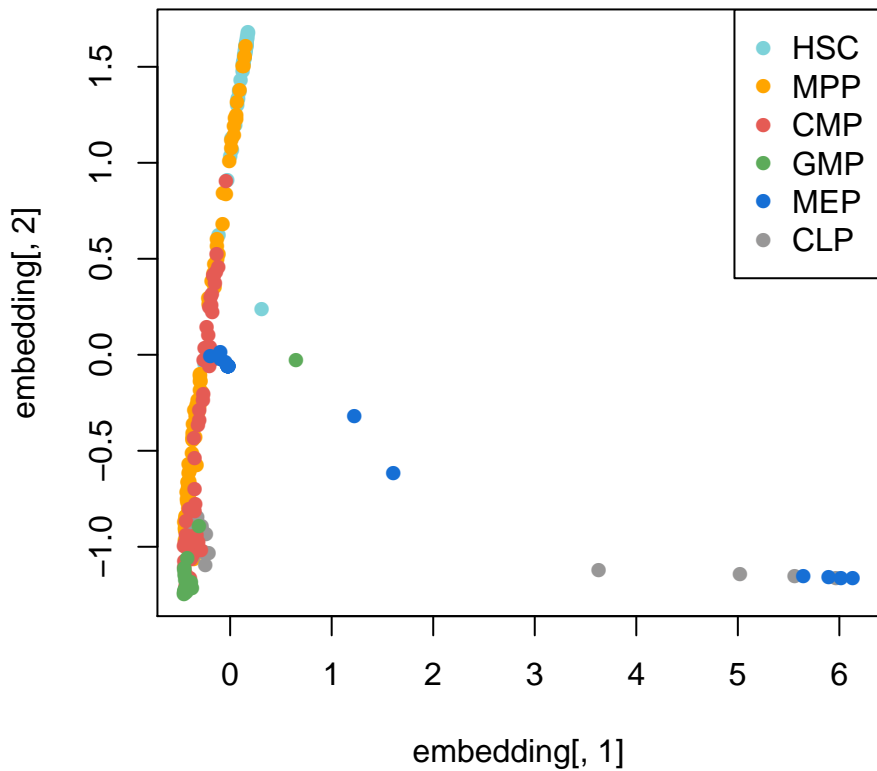

Supplement: Supplementary file 5 — Supplementary Data 2 [file 41467_2019_9670_MOESM5_ESM.zip › Sup_data2/Guo_2013/slicer/slicer_trajectory_by_label.pdf]

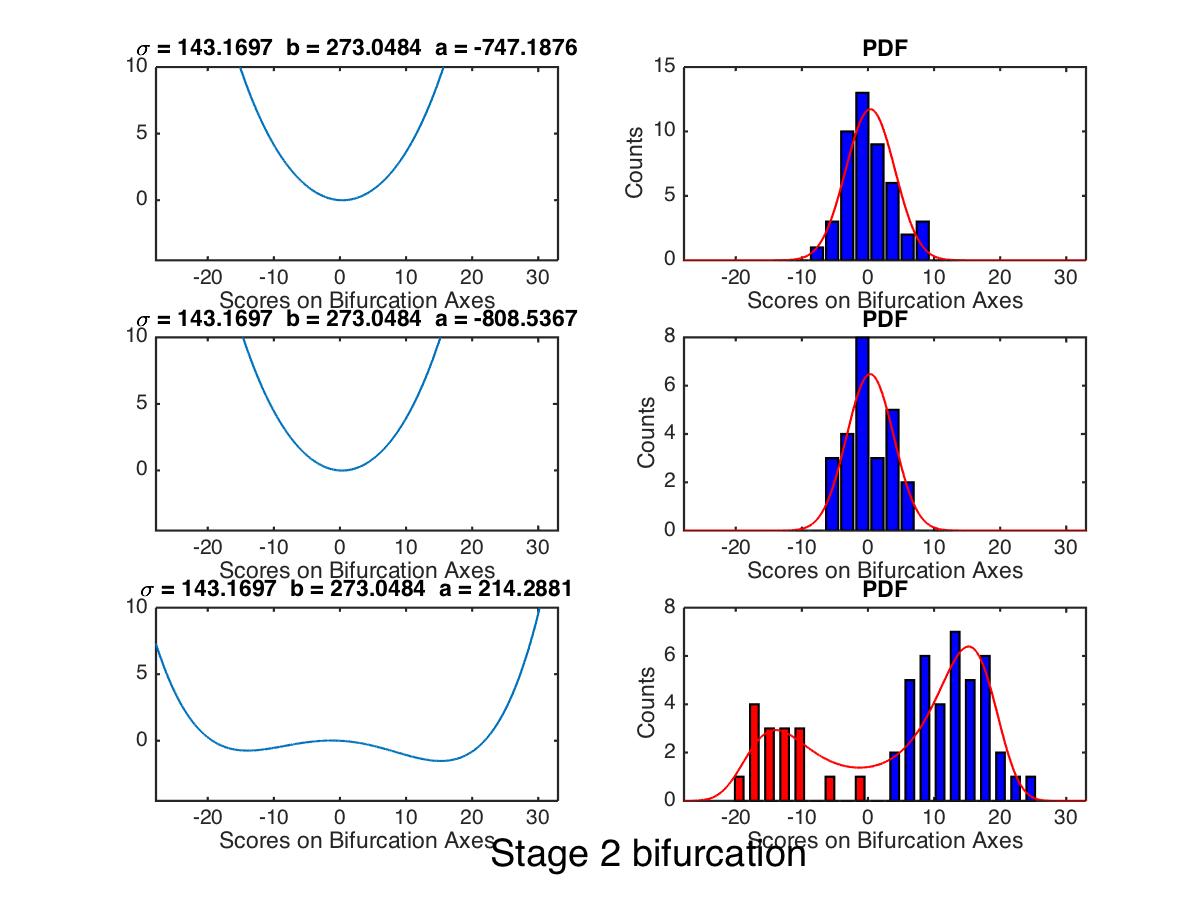

Supplement: Supplementary file 5 — Supplementary Data 2 [file 41467_2019_9670_MOESM5_ESM.zip › Sup_data2/Guo_2013/scuba/Result_run1/figures/results_fit_2.jpg]

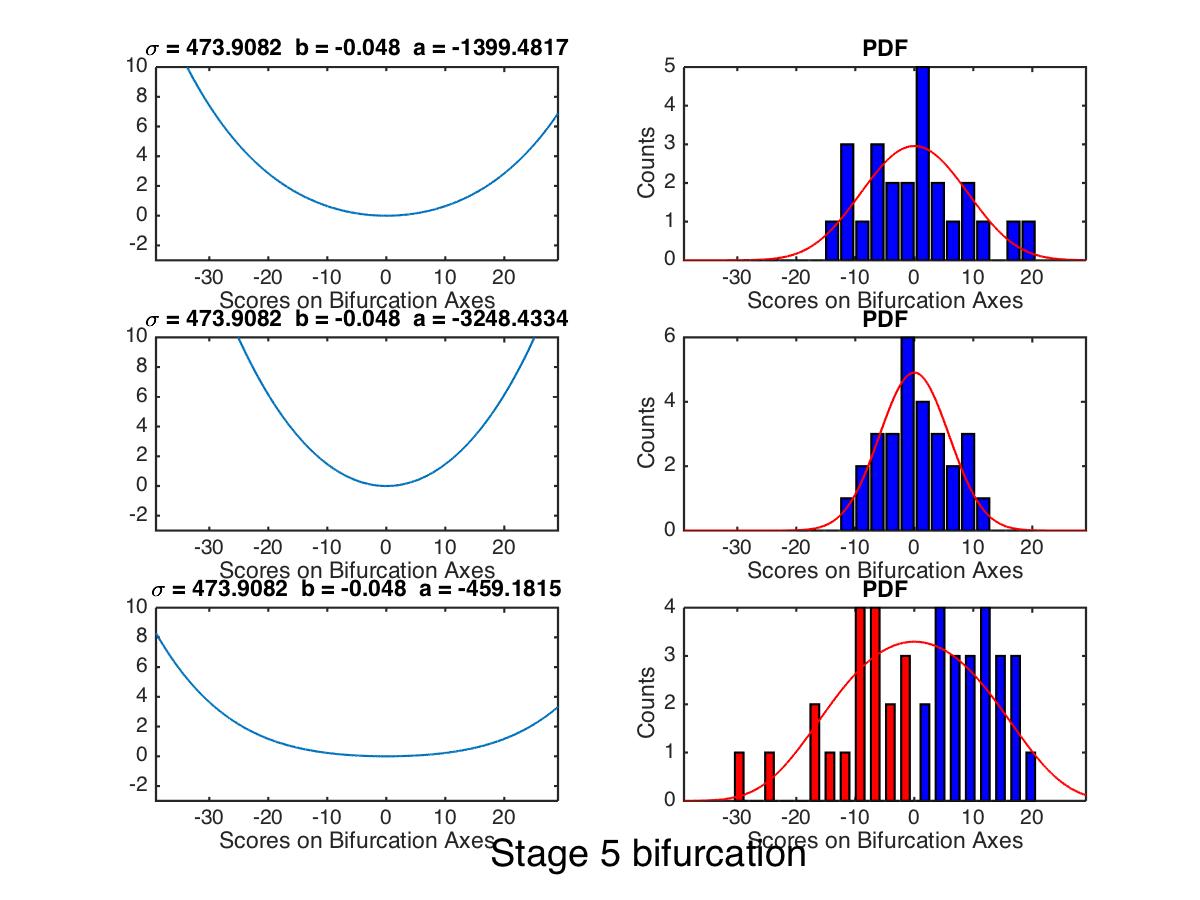

Supplement: Supplementary file 5 — Supplementary Data 2 [file 41467_2019_9670_MOESM5_ESM.zip › Sup_data2/Guo_2013/scuba/Result_run1/figures/results_fit_5.jpg]

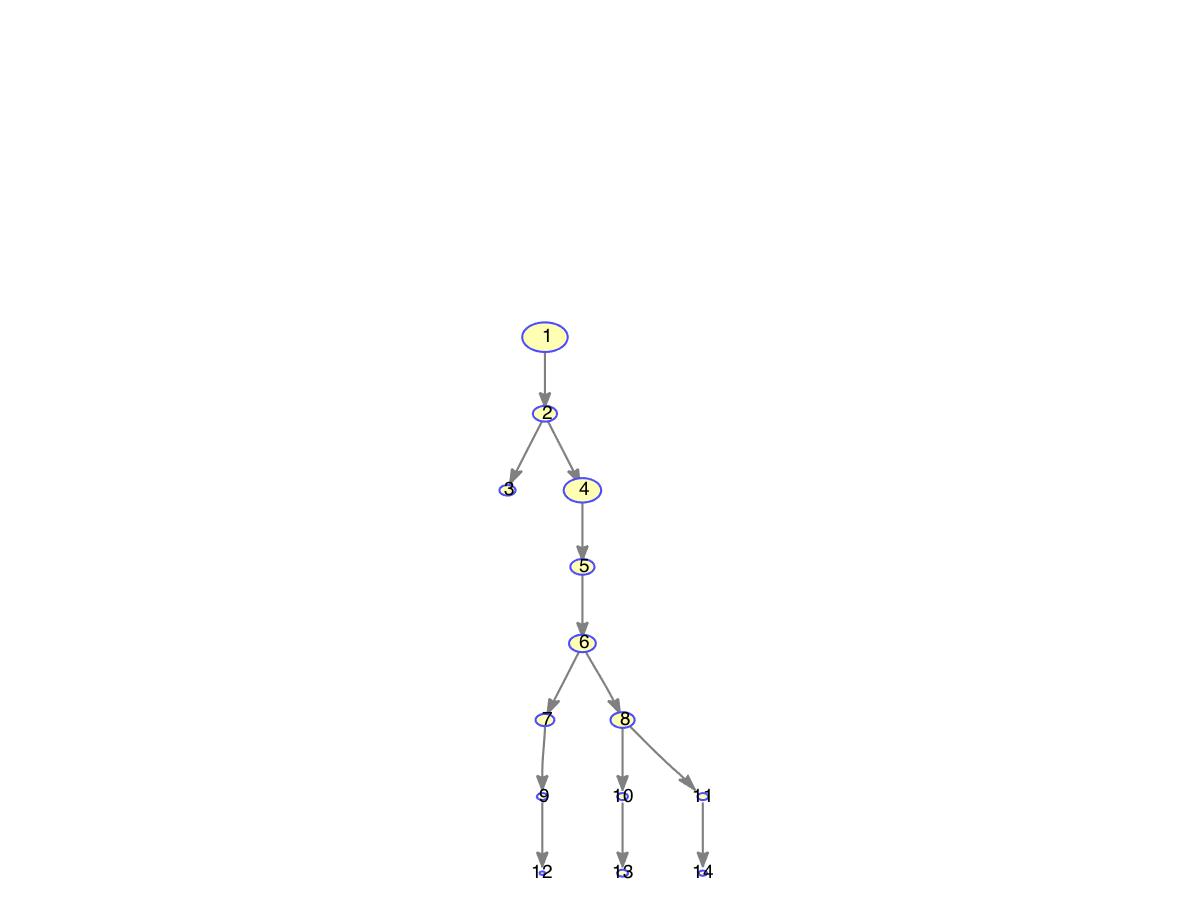

Supplement: Supplementary file 5 — Supplementary Data 2 [file 41467_2019_9670_MOESM5_ESM.zip › Sup_data2/Guo_2013/scuba/Result_run1/figures/tree.jpg]

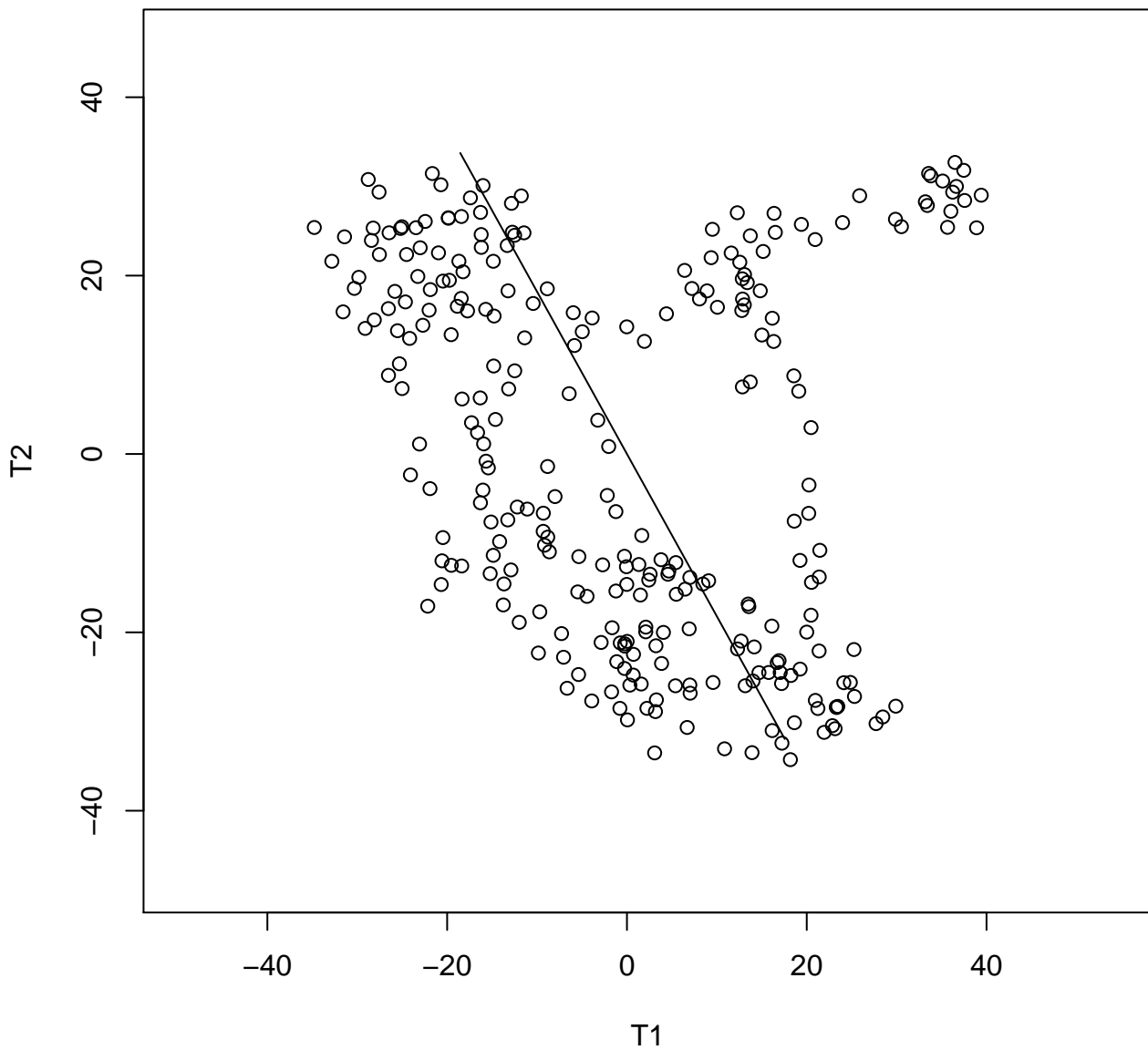

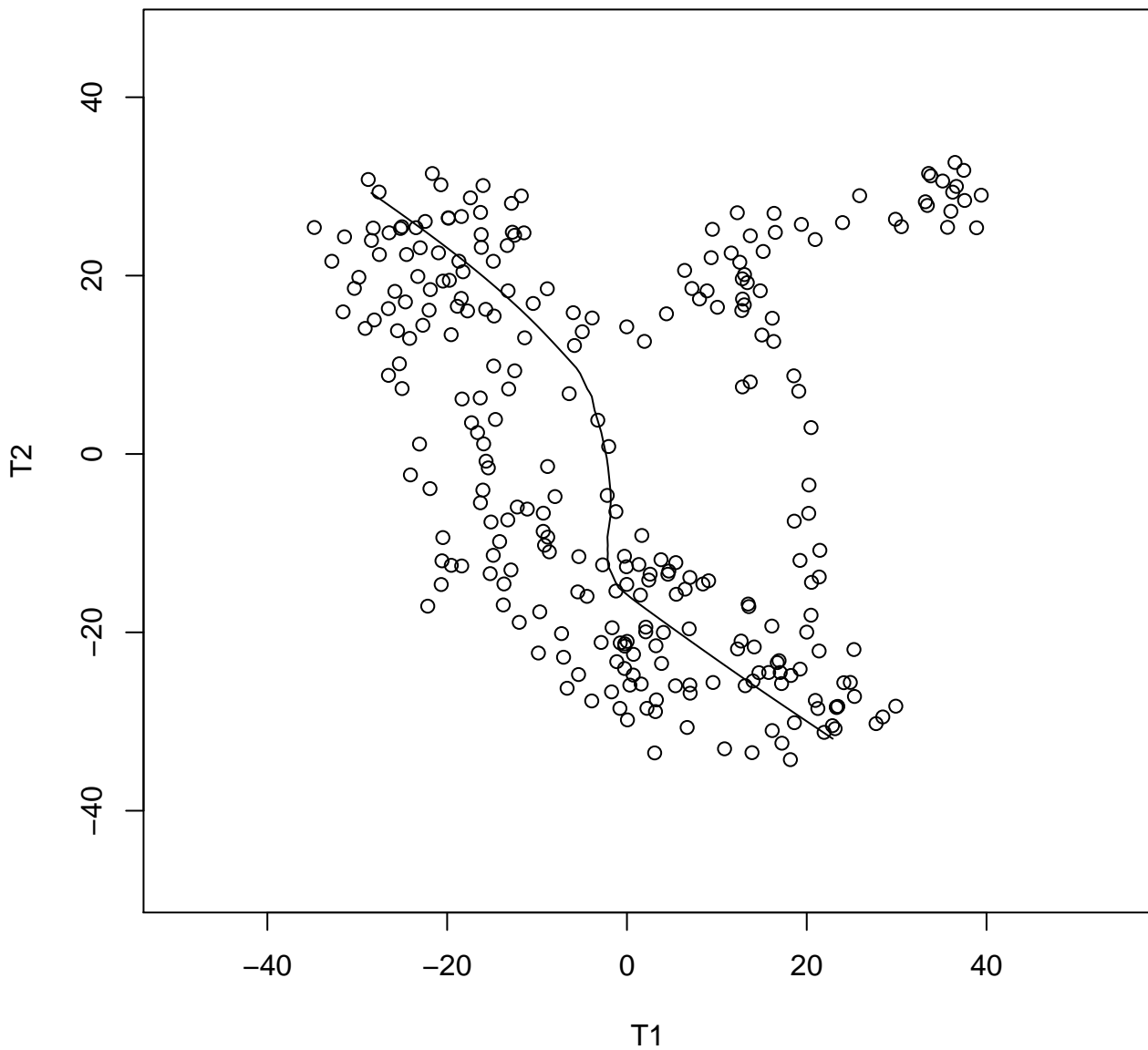

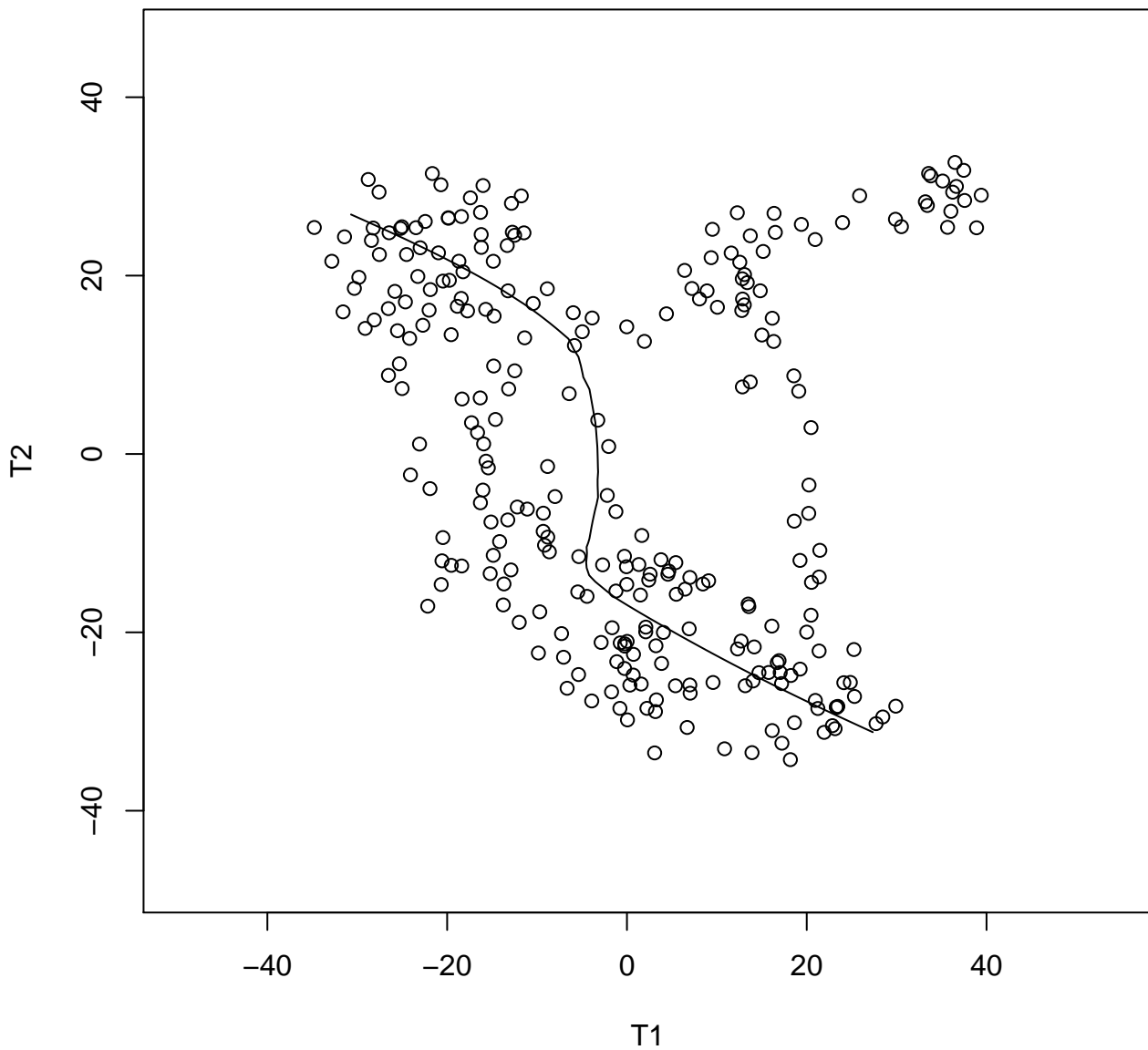

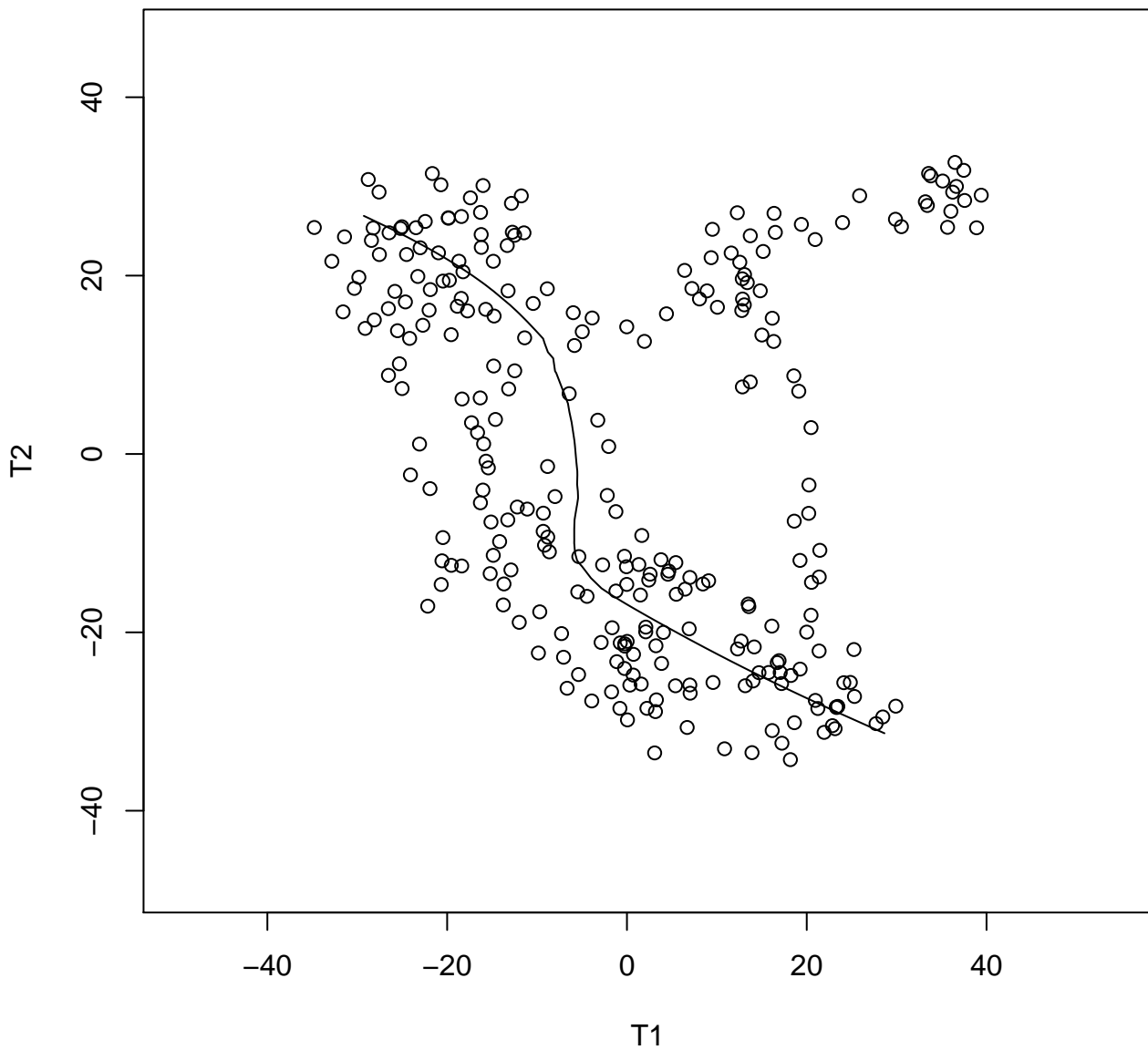

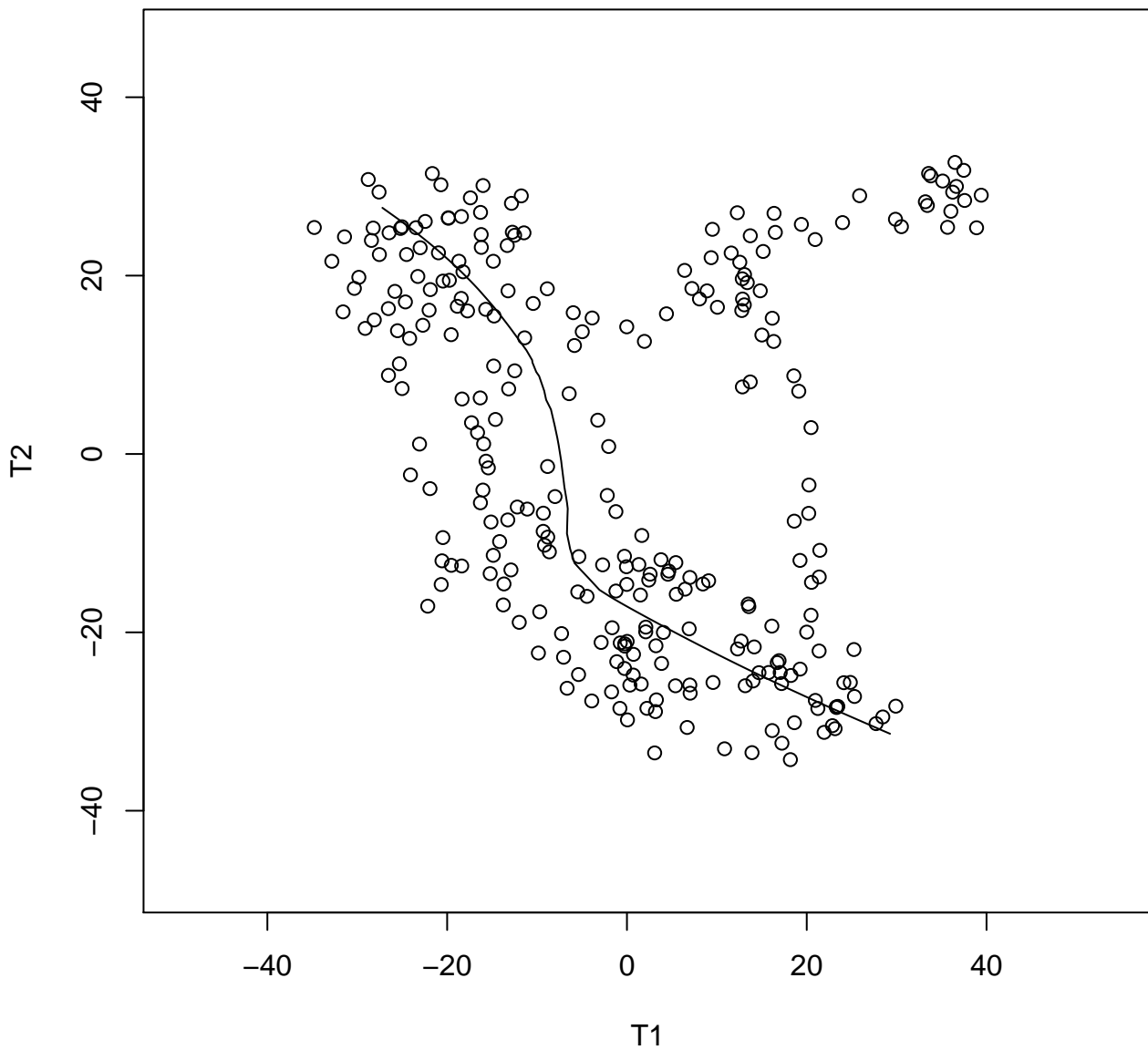

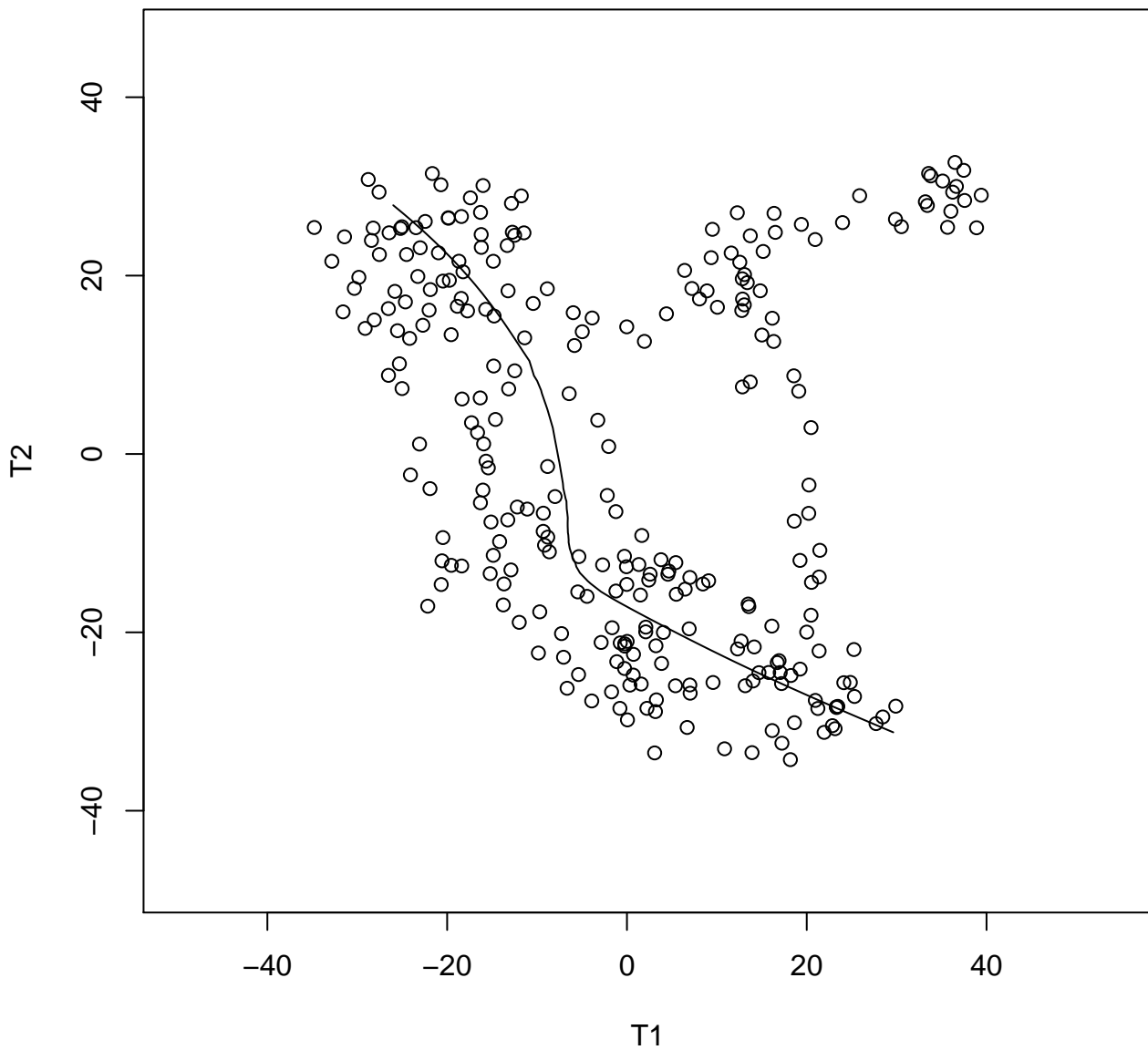

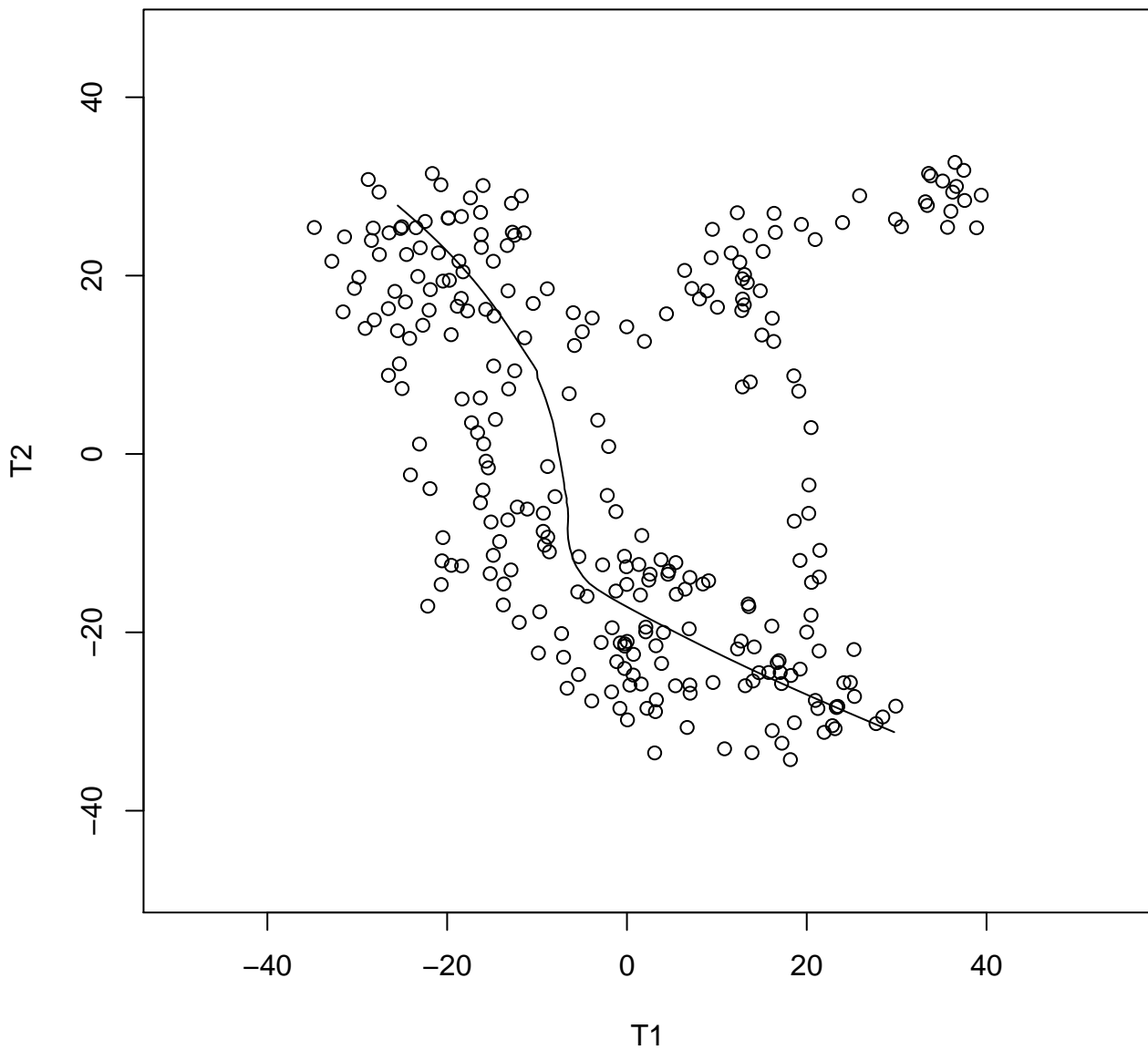

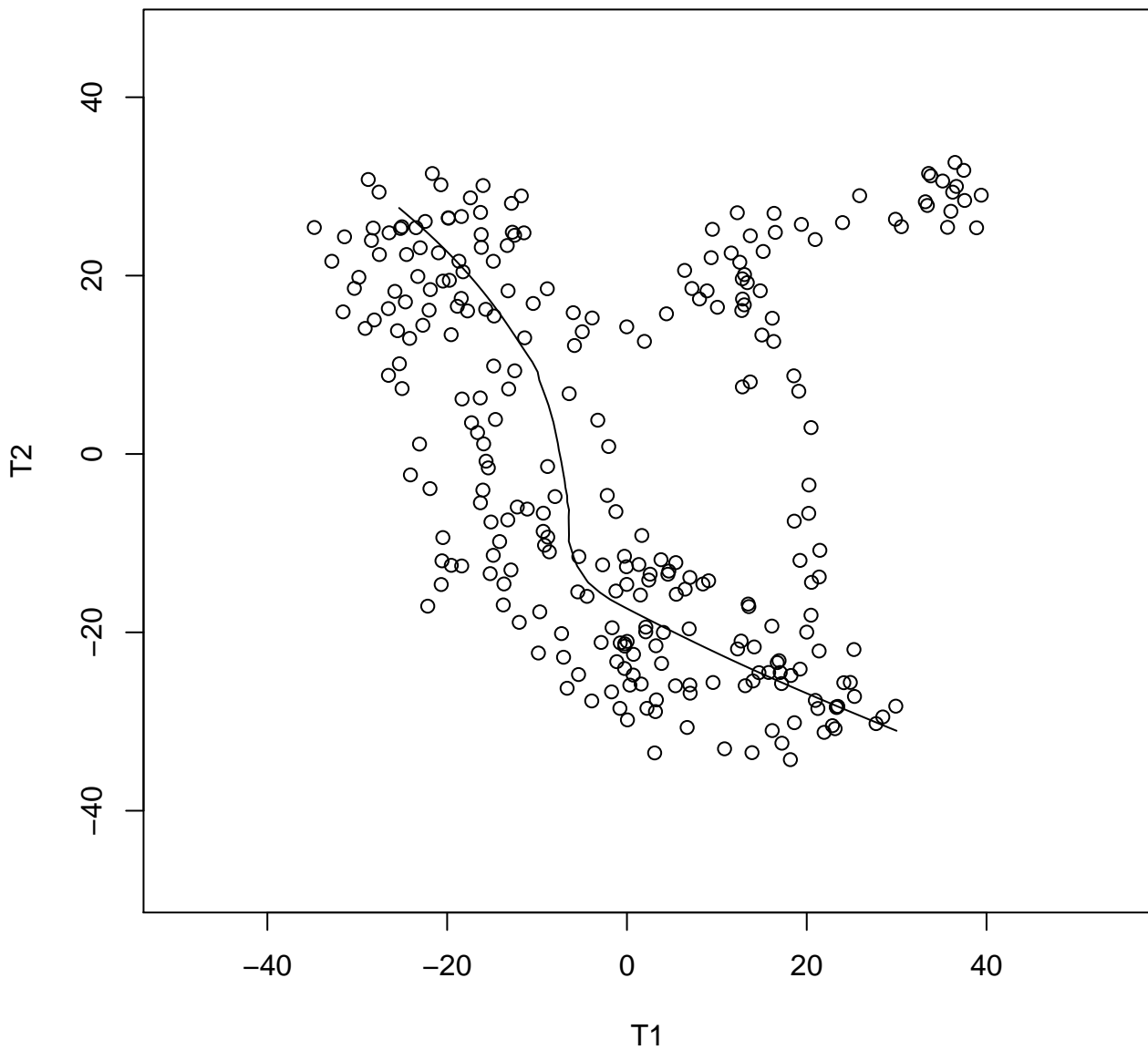

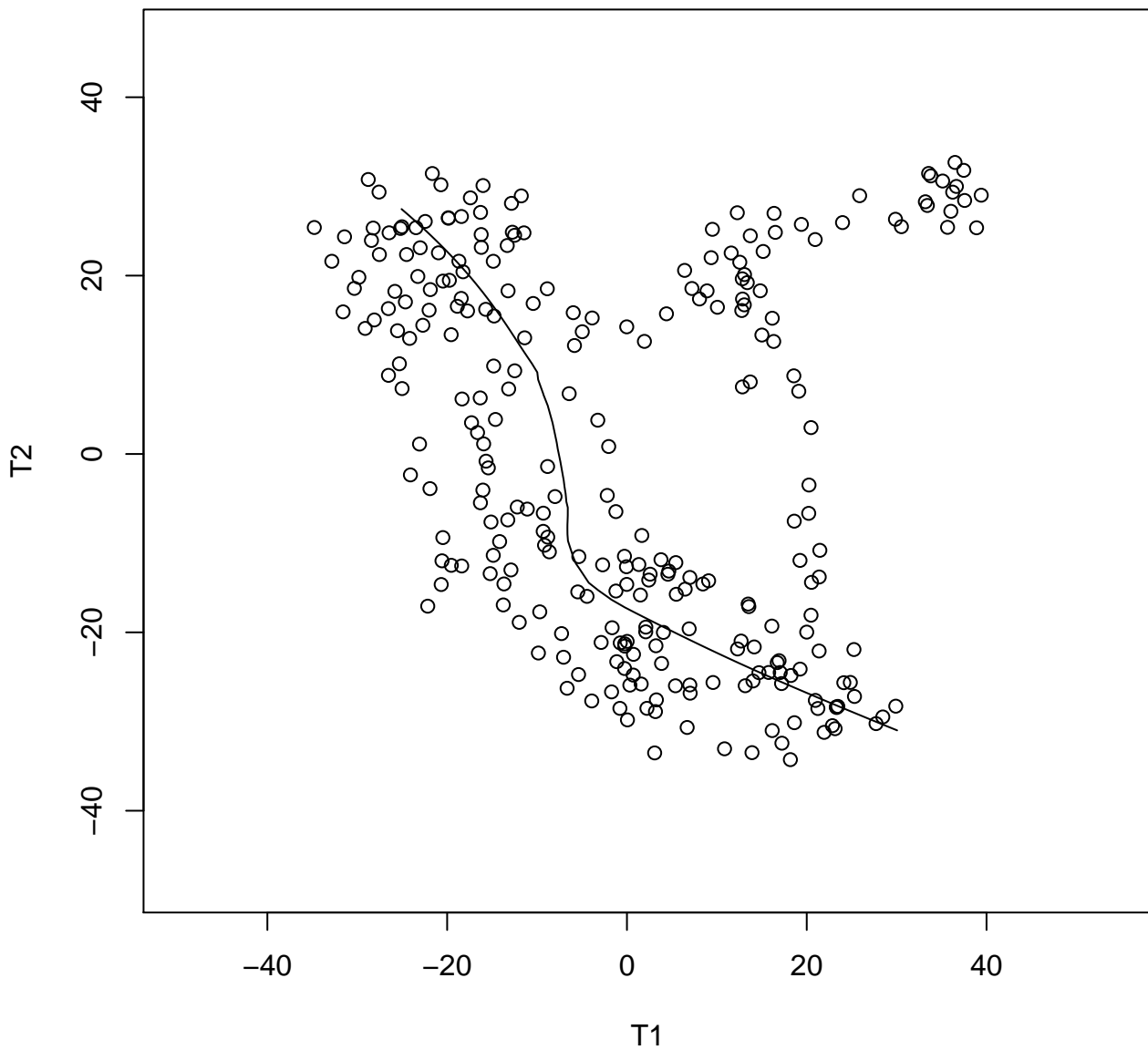

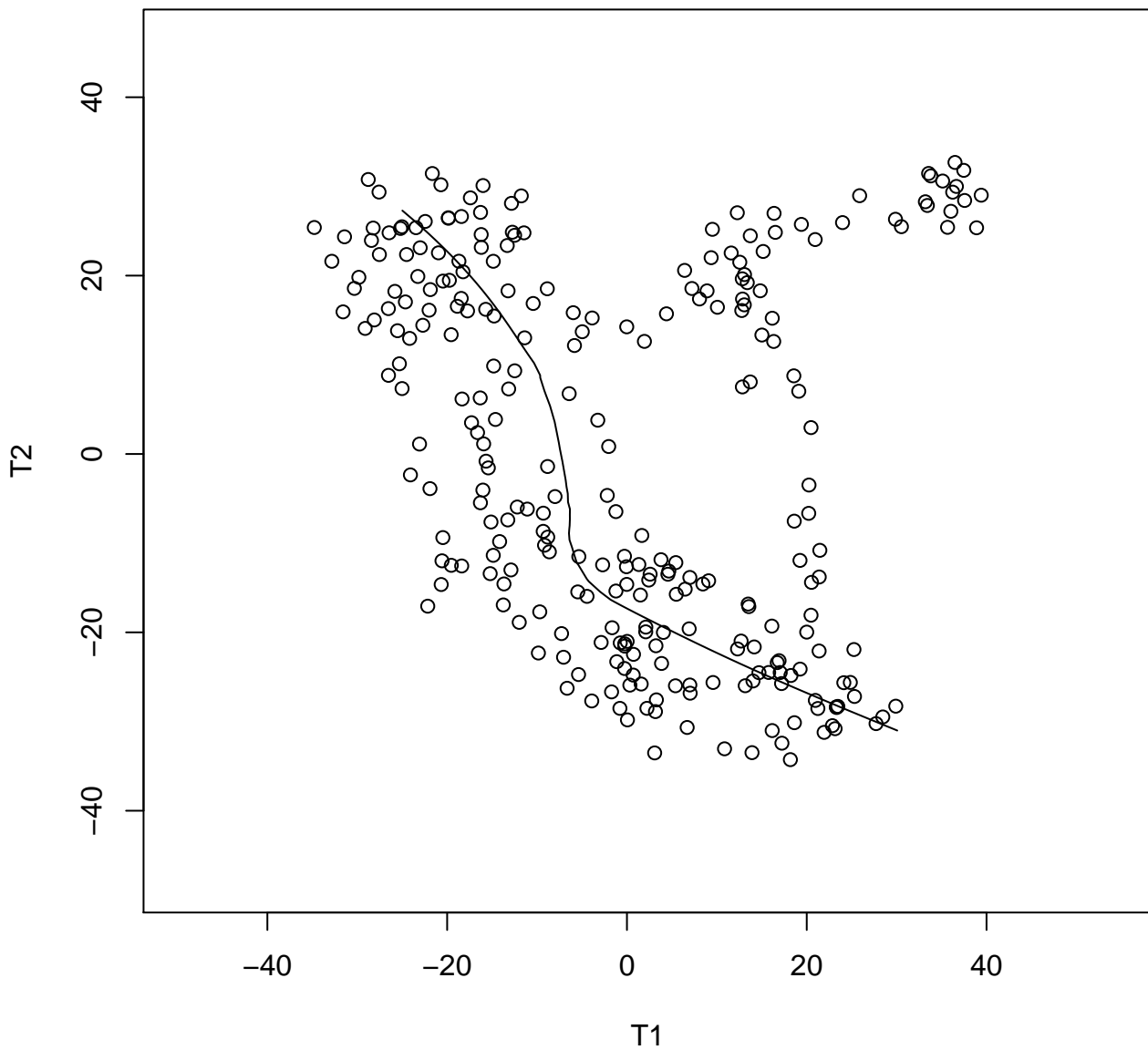

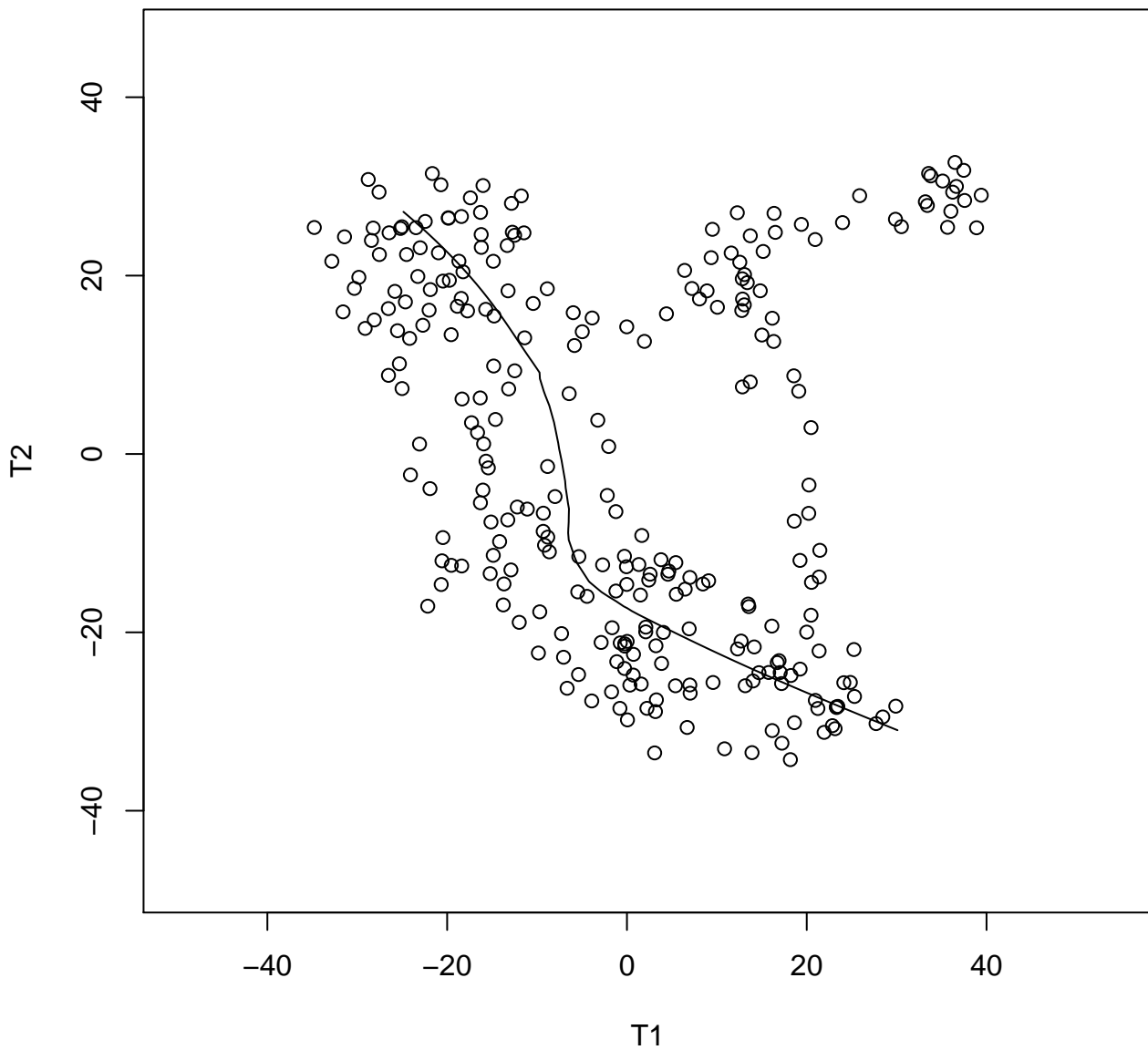

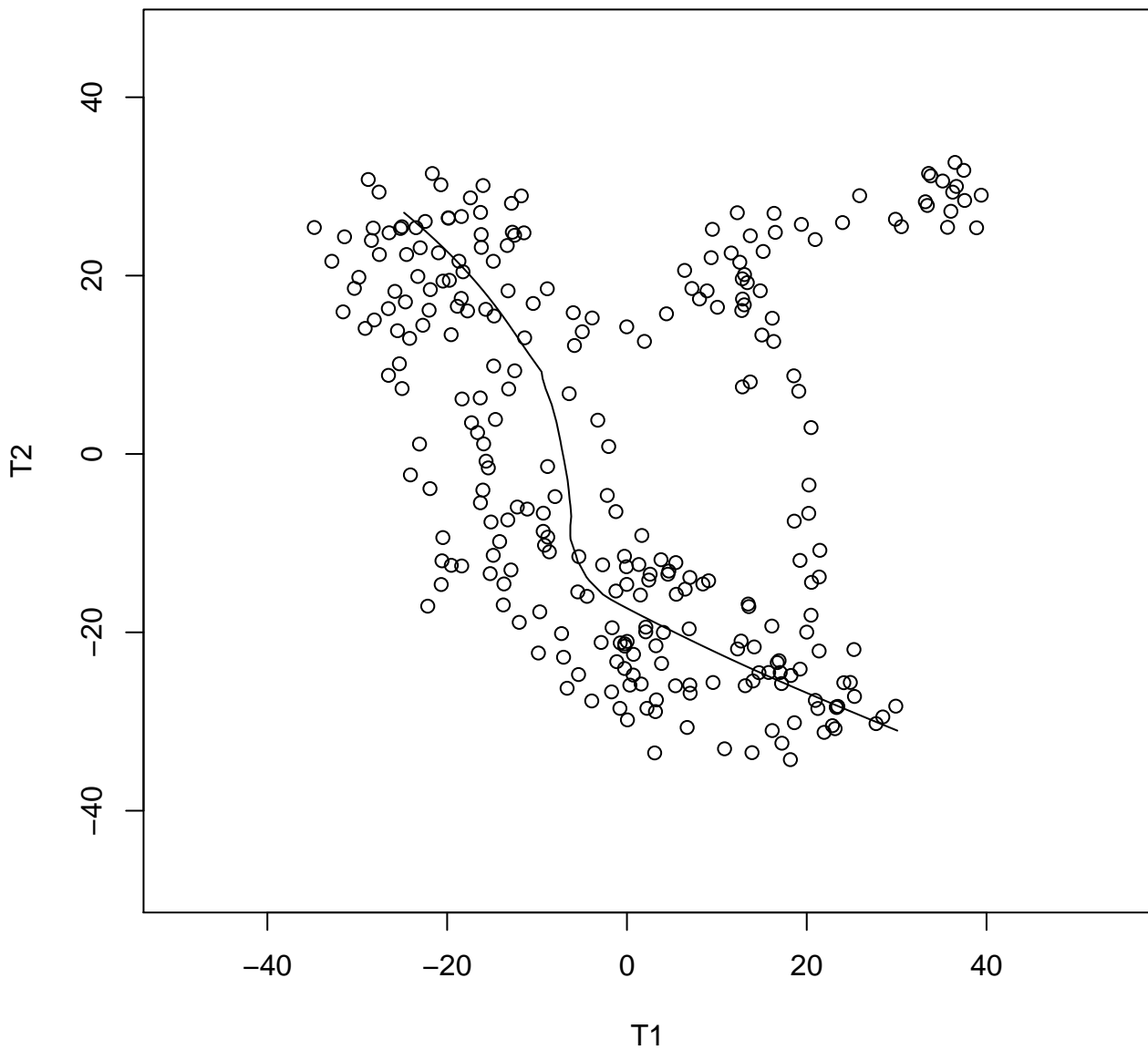

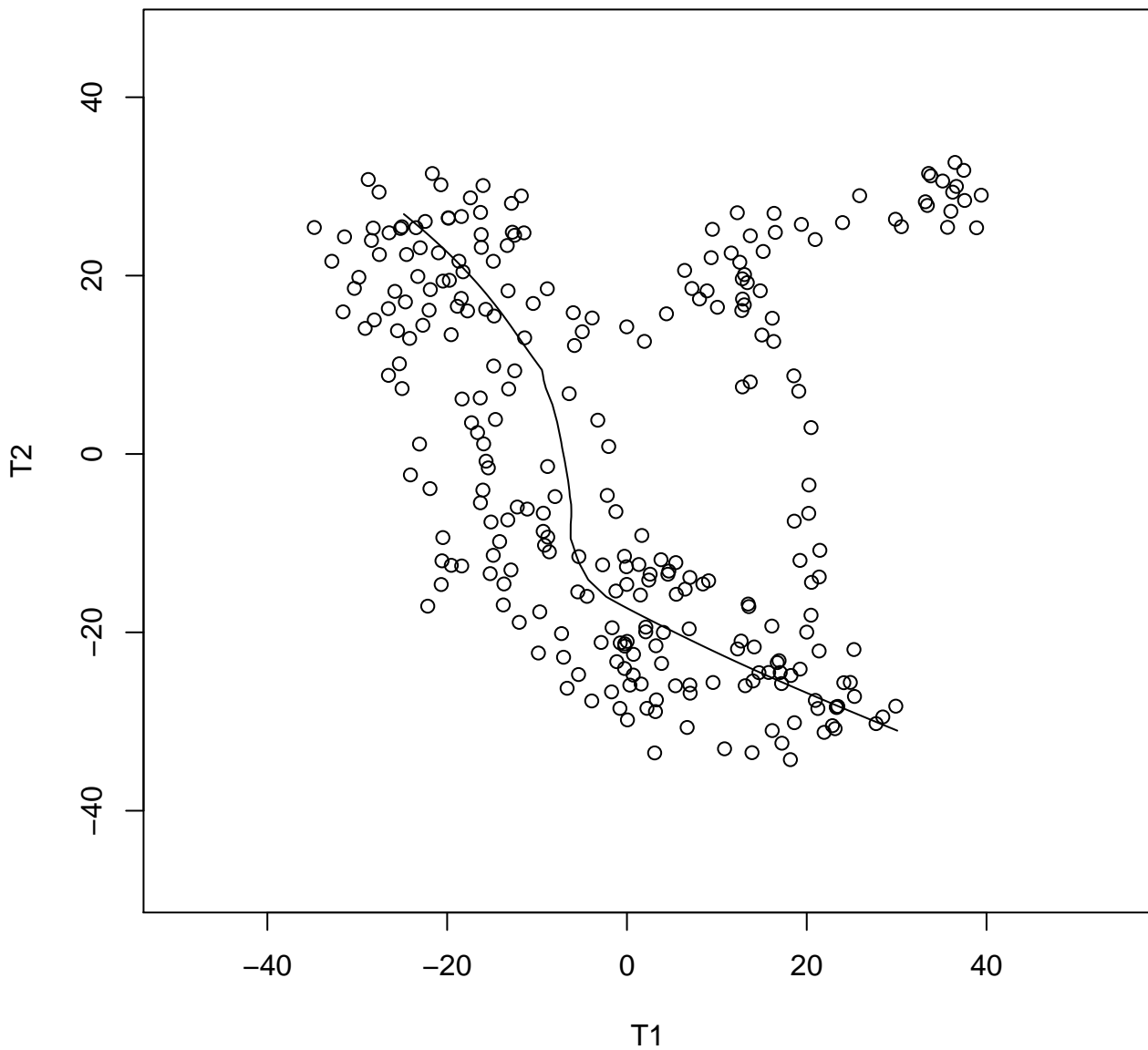

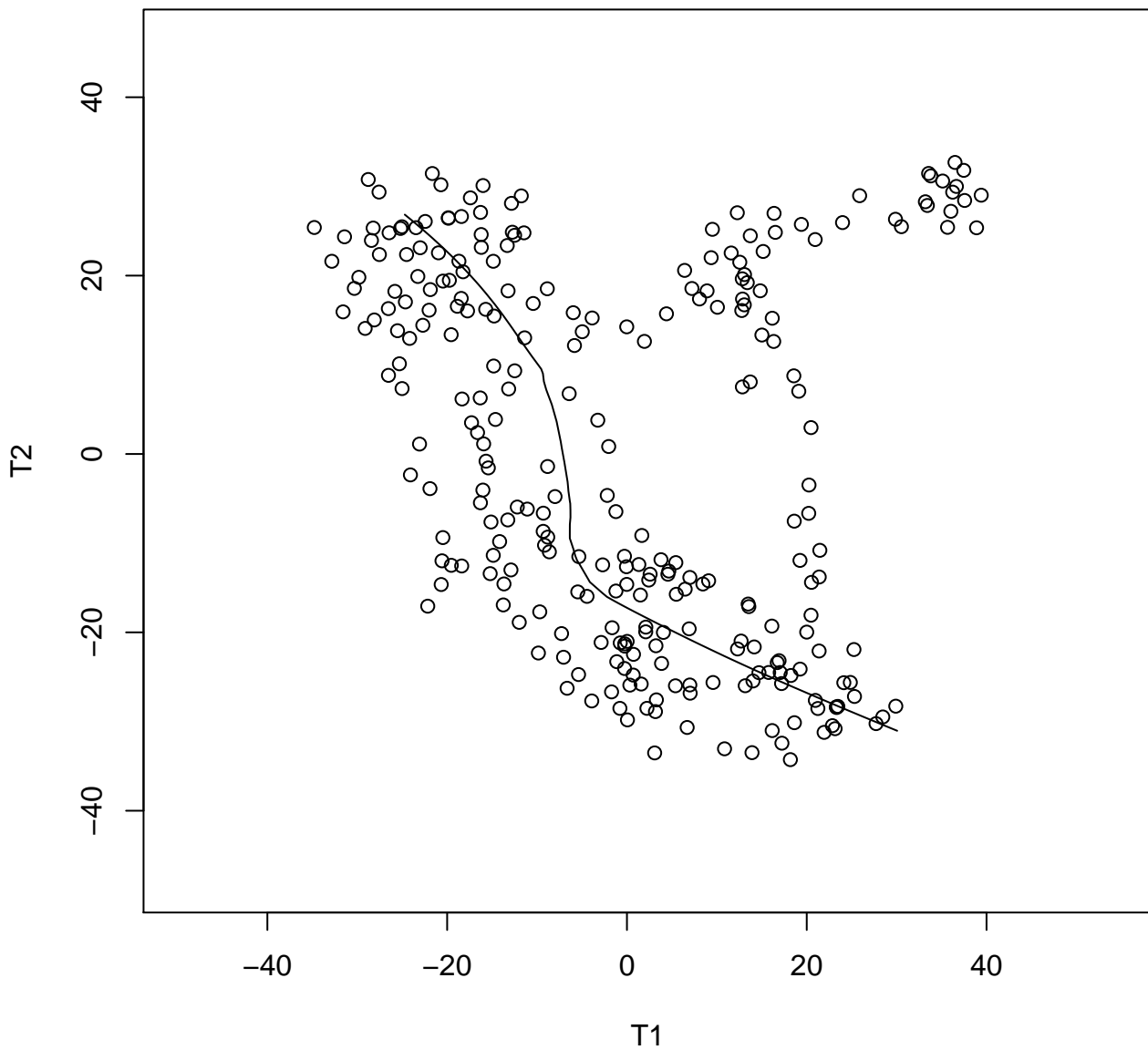

Supplement: Supplementary file 5 — Supplementary Data 2 [file 41467_2019_9670_MOESM5_ESM.zip › Sup_data2/Guo_2013/scuba/Result_run1/Rplots.pdf]

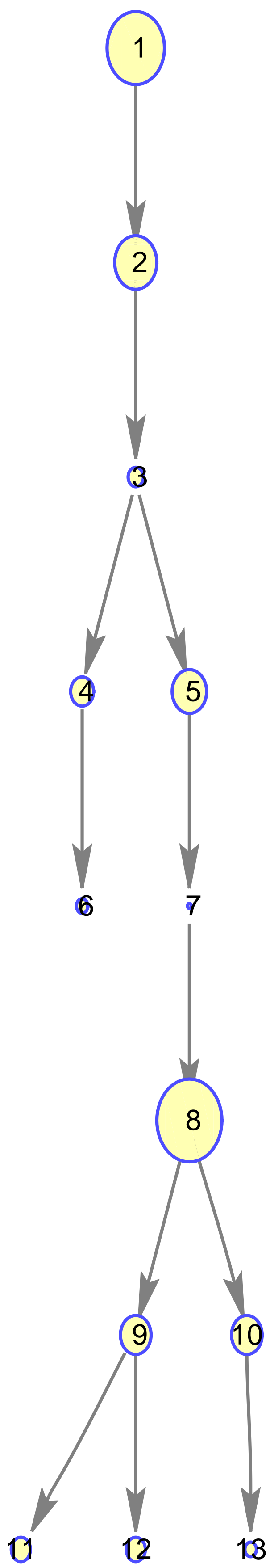

Supplement: Supplementary file 5 — Supplementary Data 2 [file 41467_2019_9670_MOESM5_ESM.zip › Sup_data2/Guo_2013/scuba/sample_data/qPCR/figures/tree.pdf]

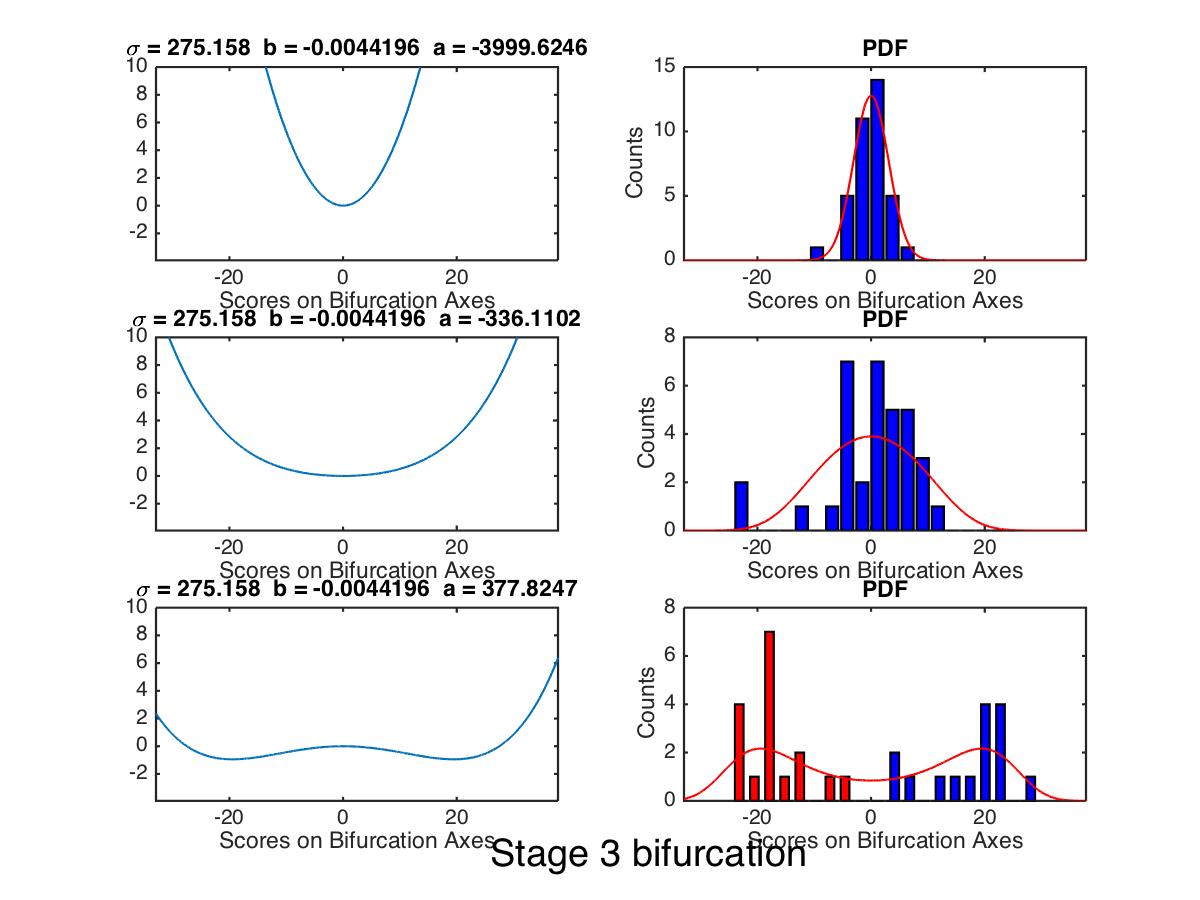

Supplement: Supplementary file 5 — Supplementary Data 2 [file 41467_2019_9670_MOESM5_ESM.zip › Sup_data2/Guo_2013/scuba/Result_run2/figures/results_fit_3.jpg]

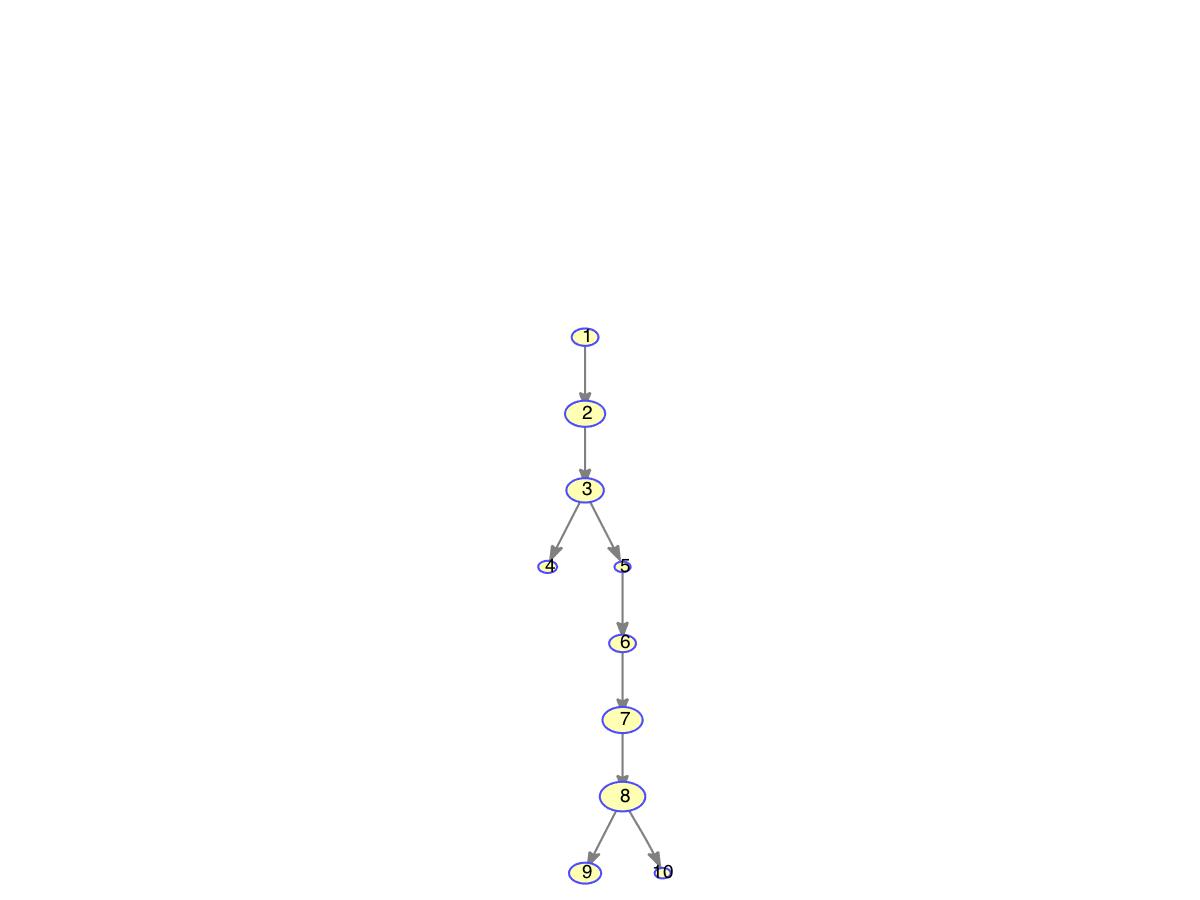

Supplement: Supplementary file 5 — Supplementary Data 2 [file 41467_2019_9670_MOESM5_ESM.zip › Sup_data2/Guo_2013/scuba/Result_run2/figures/tree.jpg]

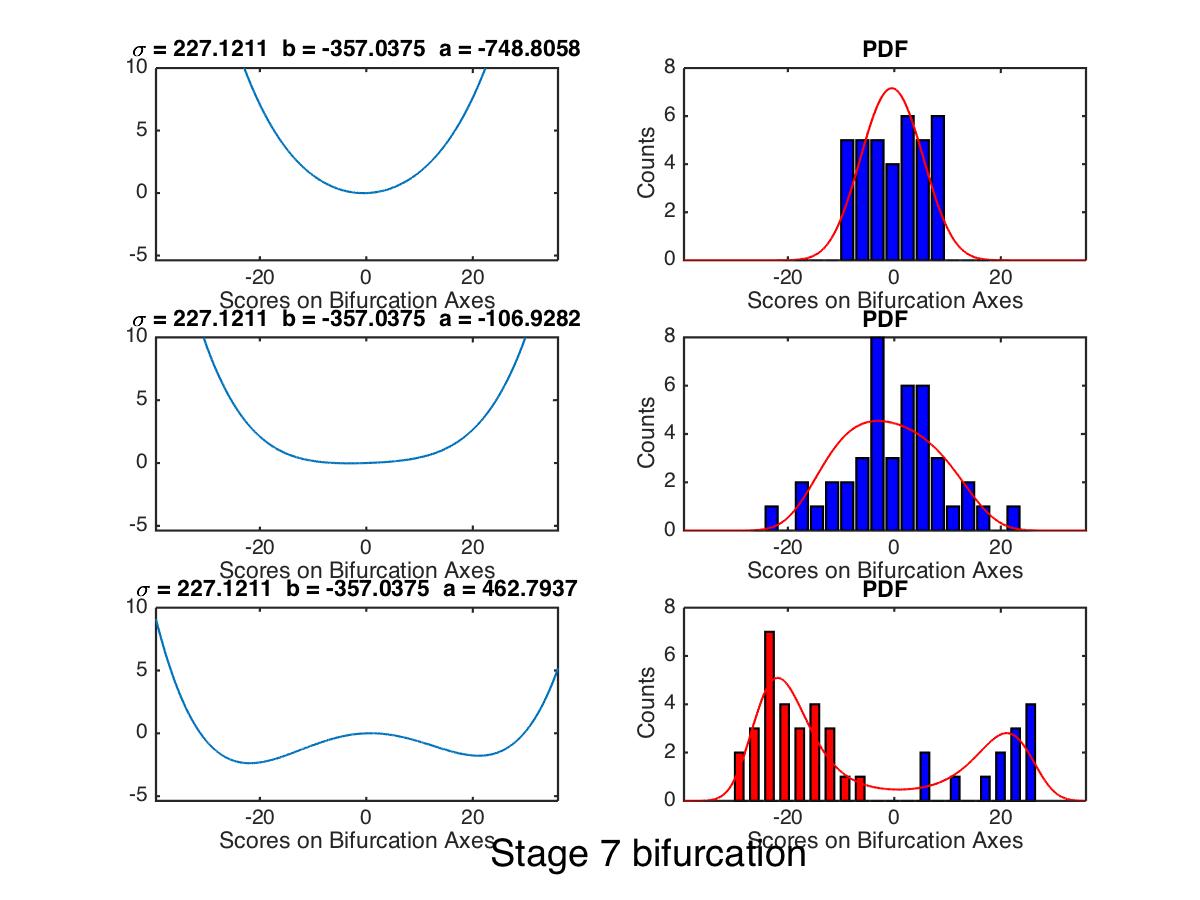

Supplement: Supplementary file 5 — Supplementary Data 2 [file 41467_2019_9670_MOESM5_ESM.zip › Sup_data2/Guo_2013/scuba/Result_run2/figures/results_fit_7.jpg]

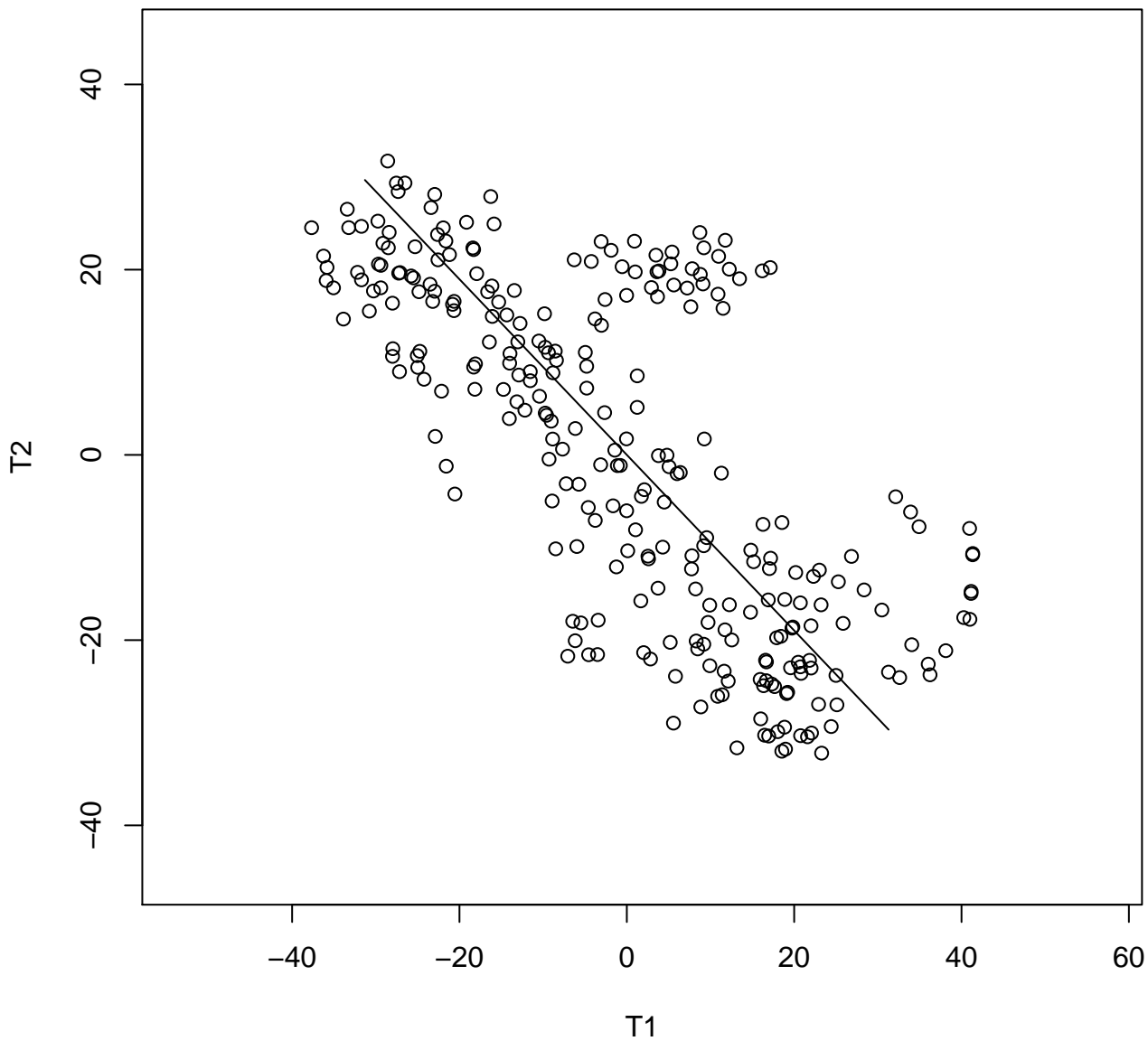

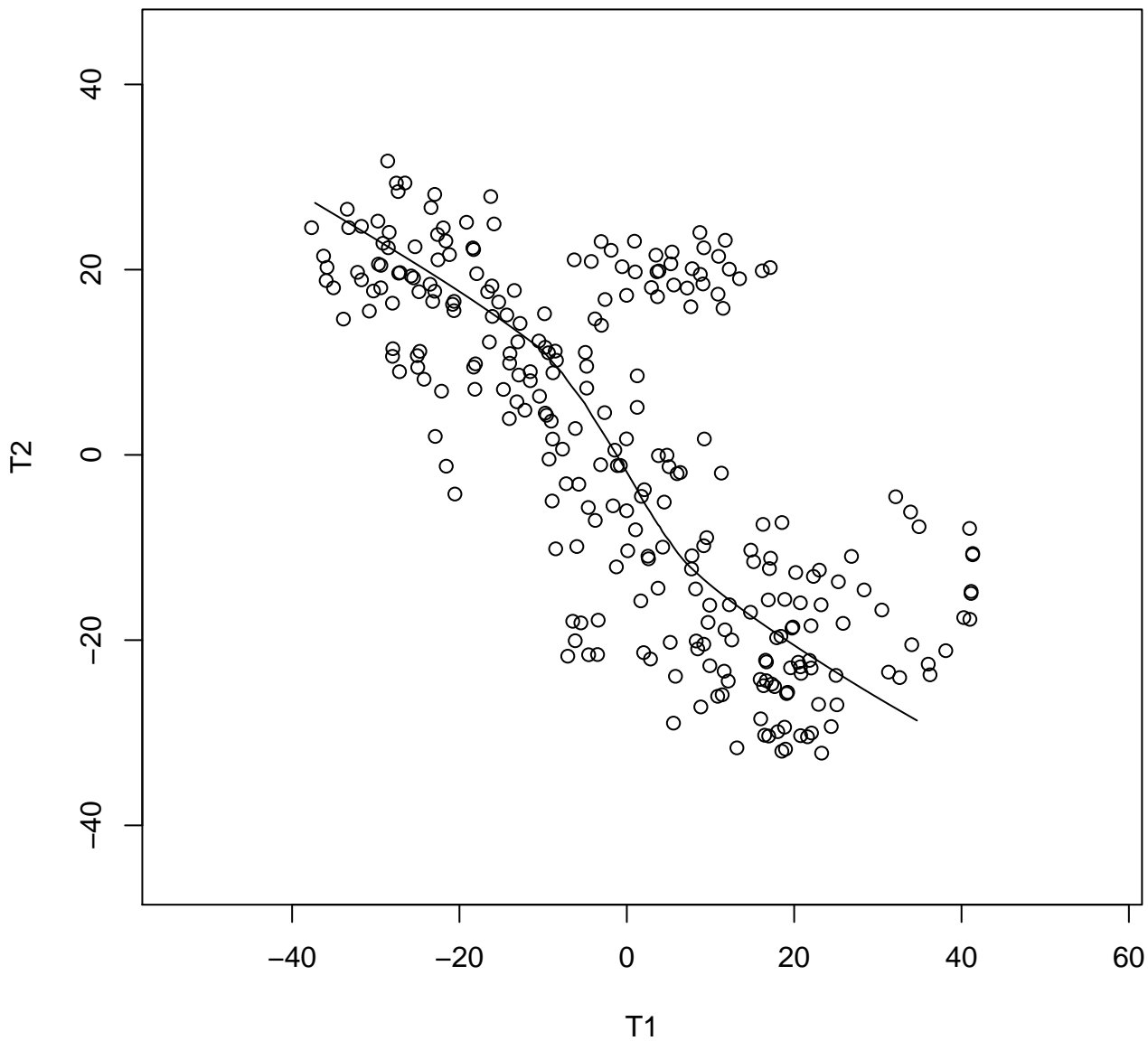

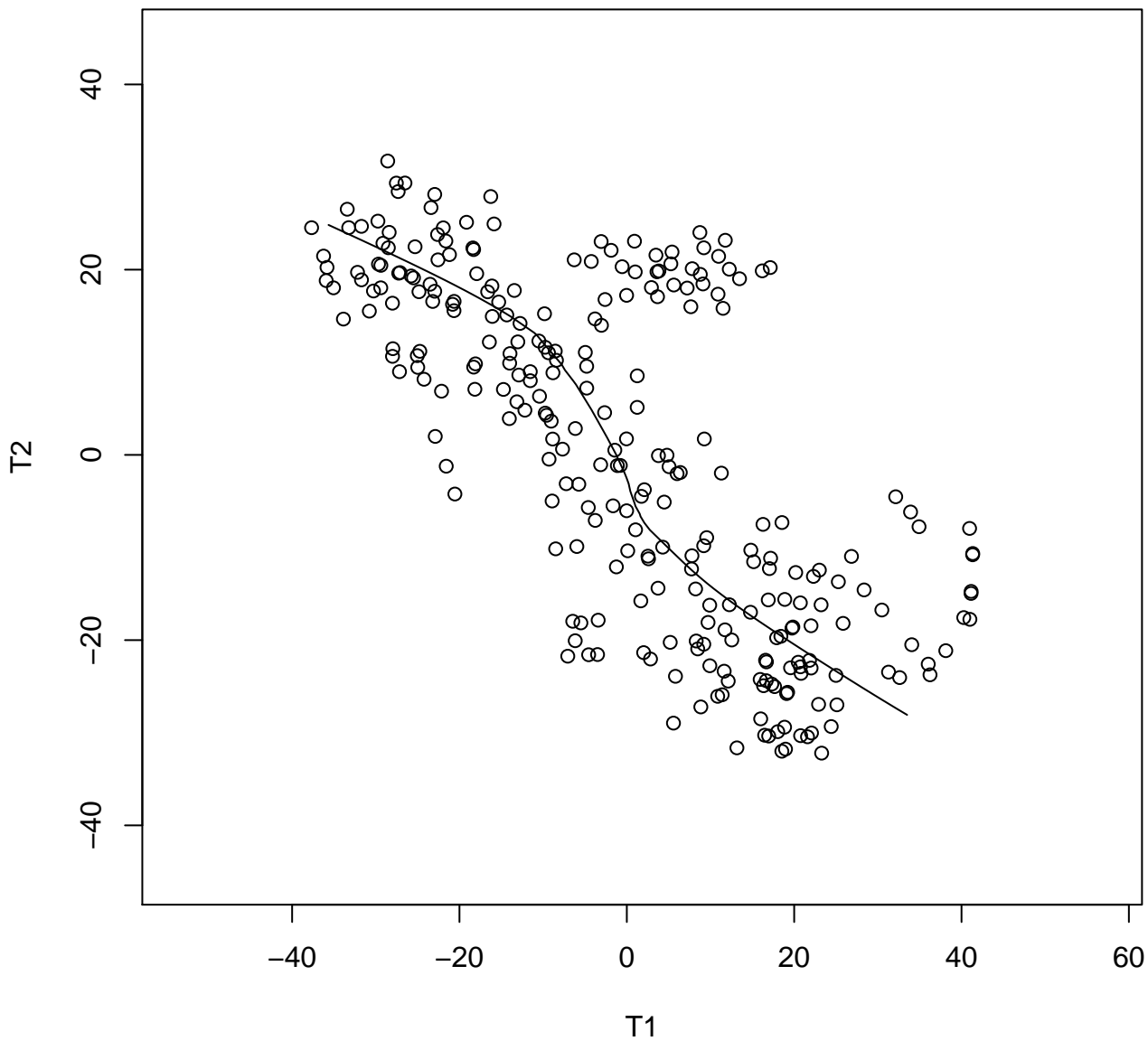

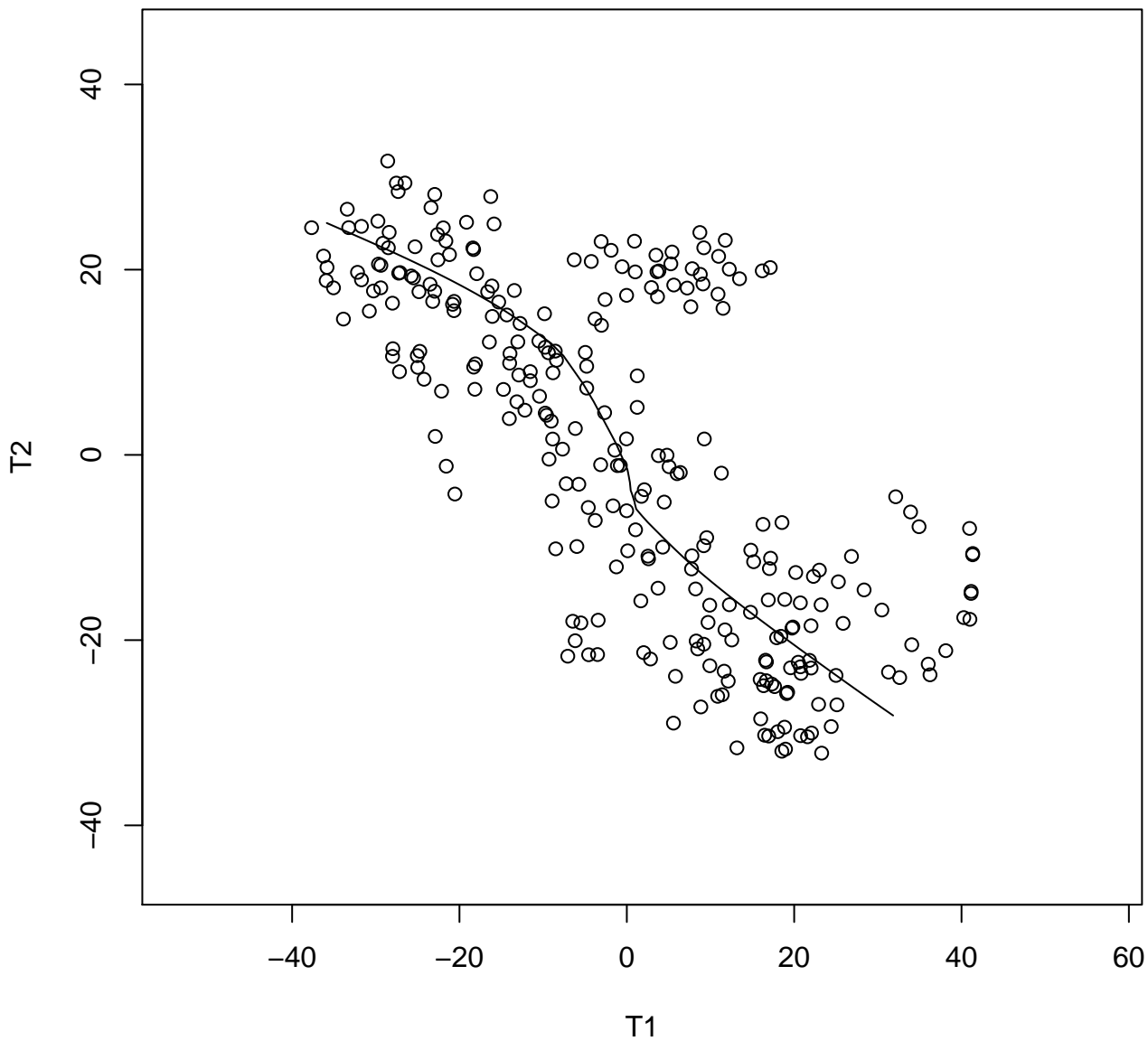

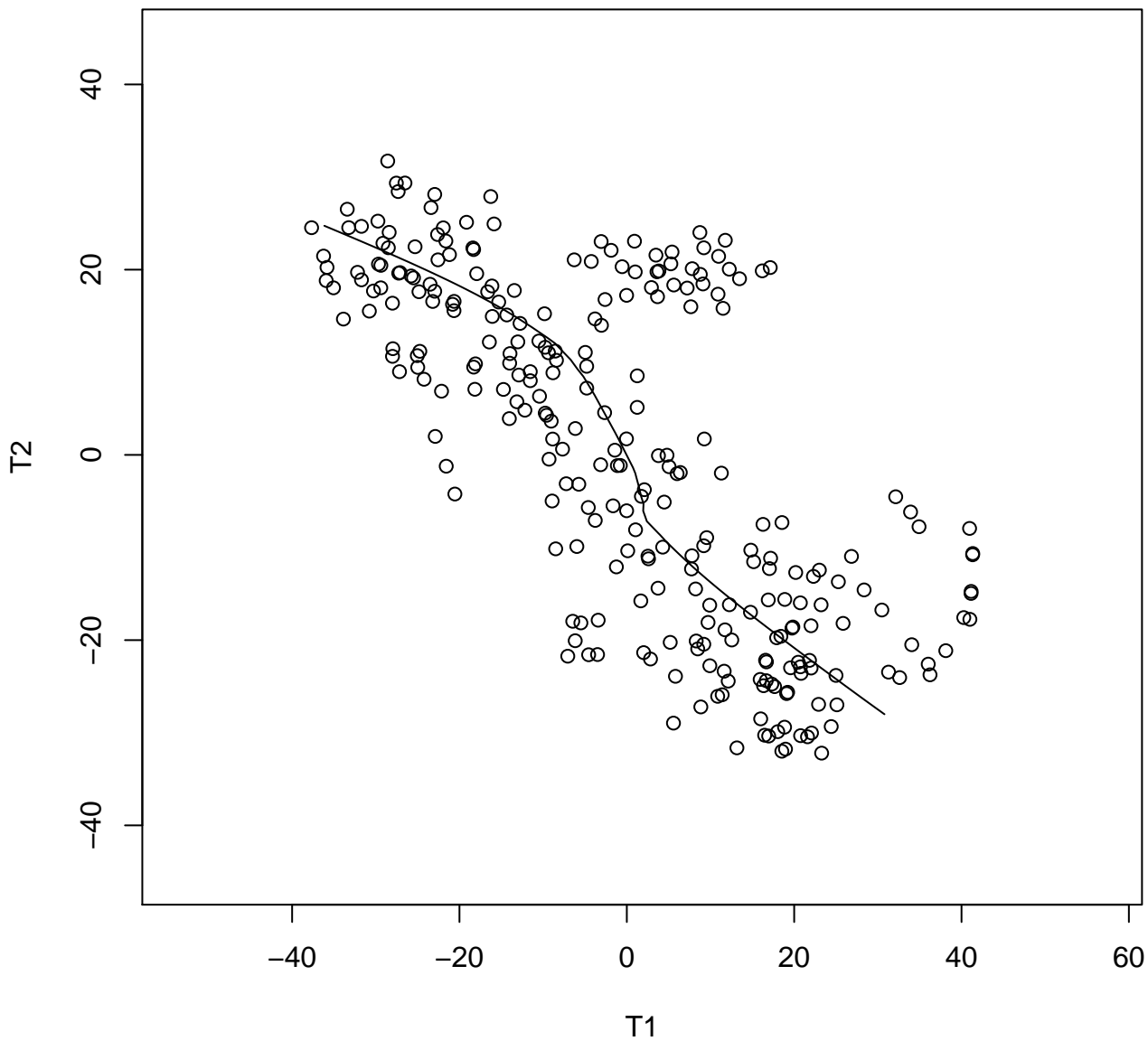

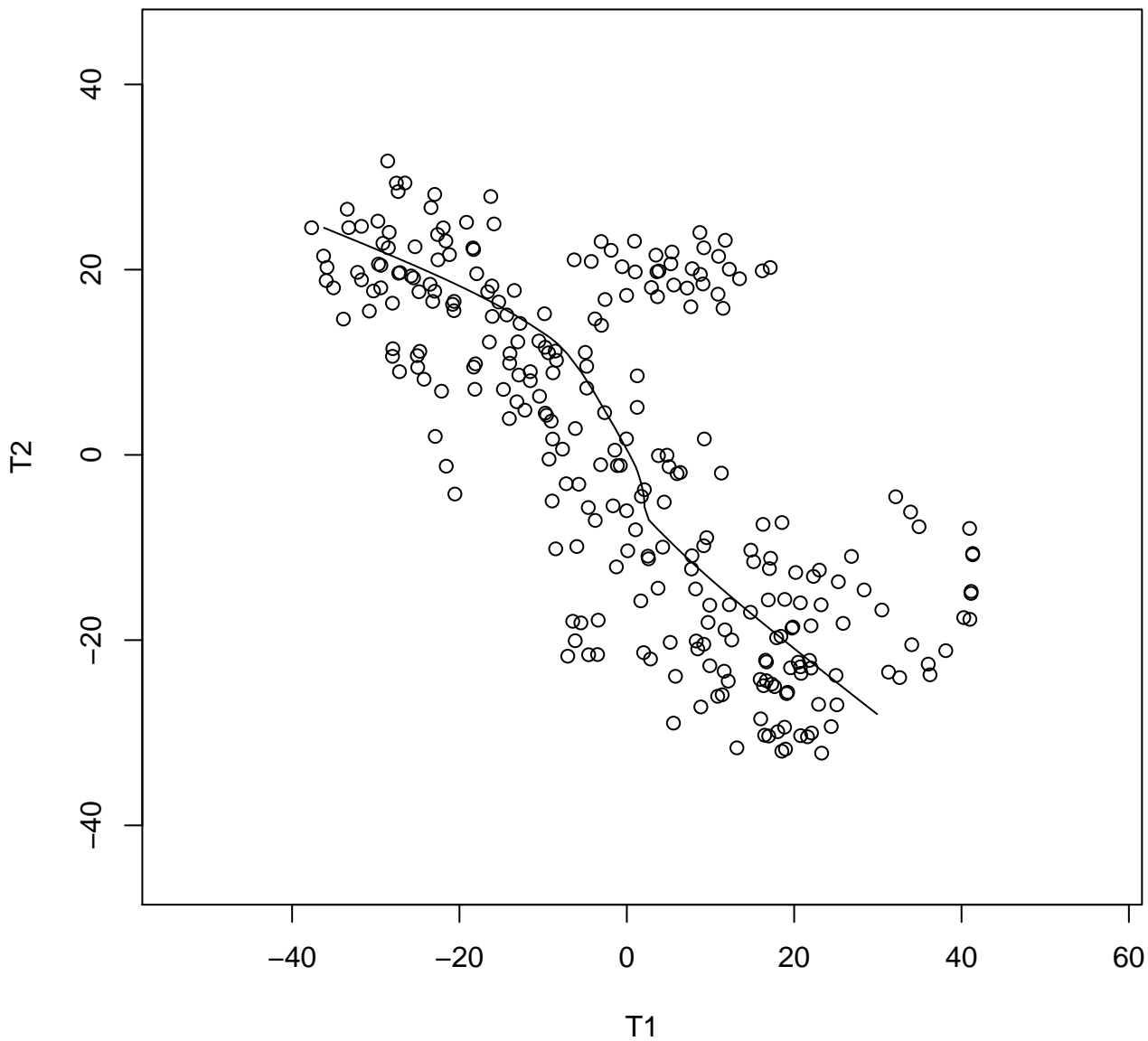

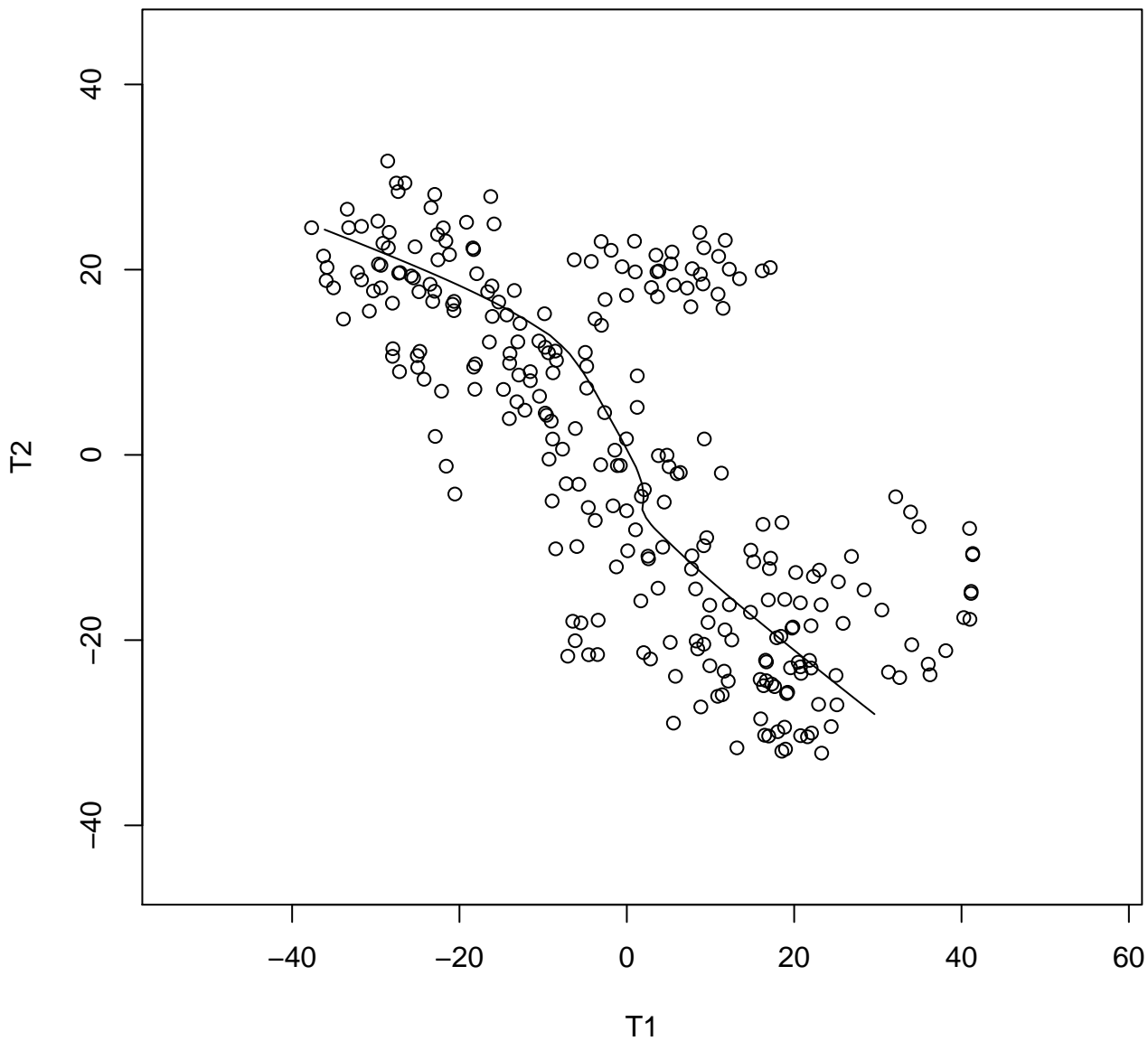

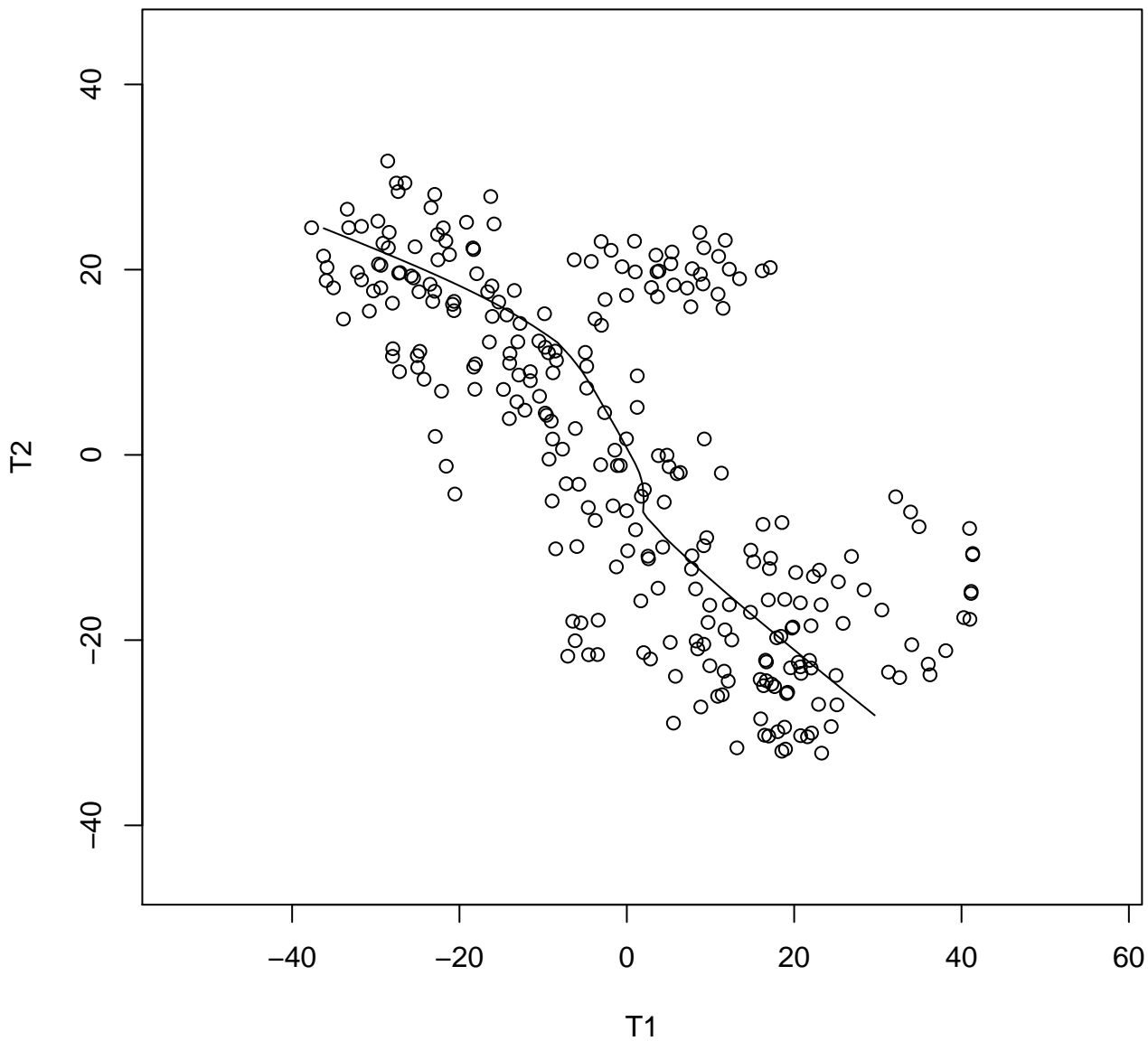

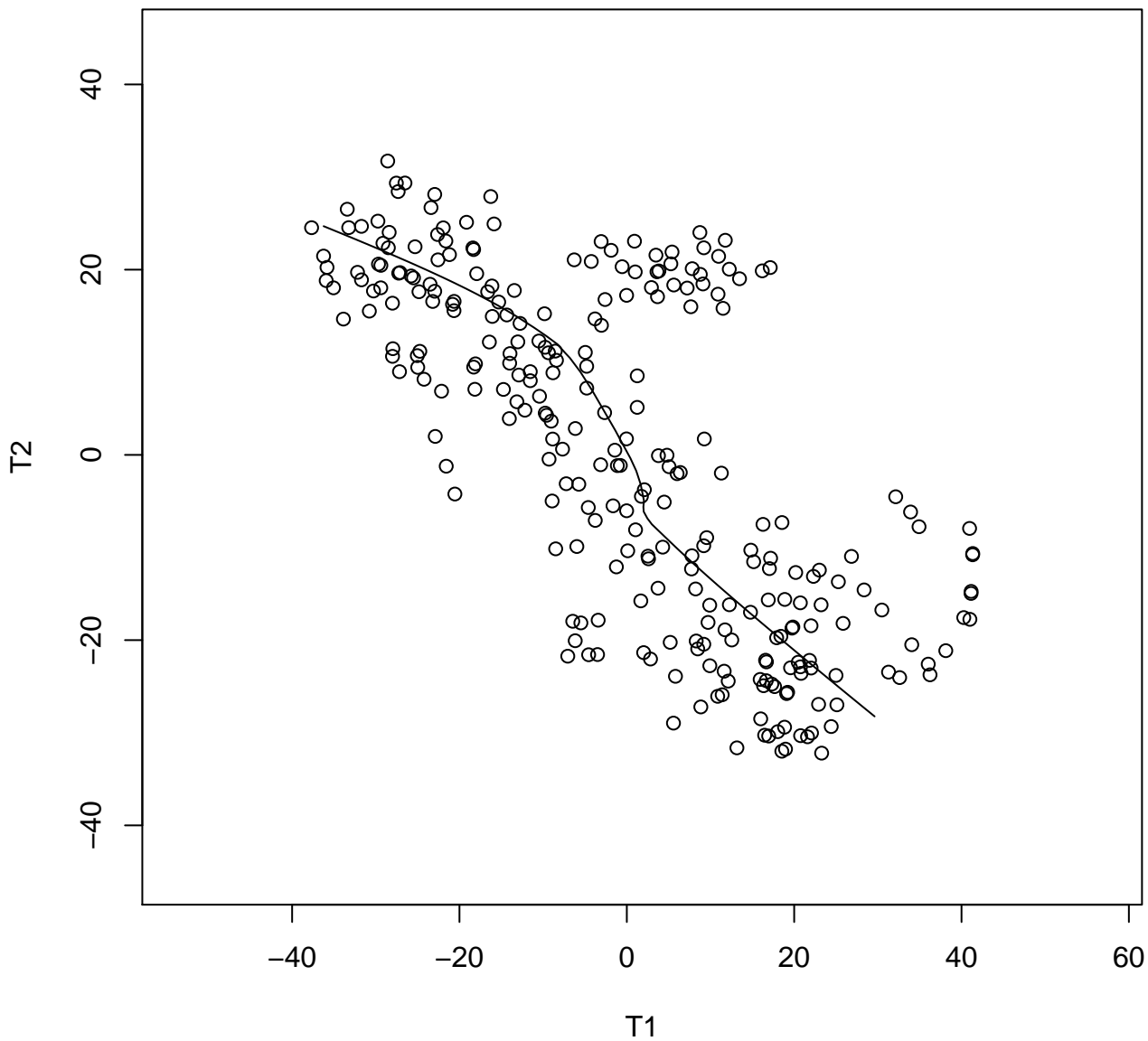

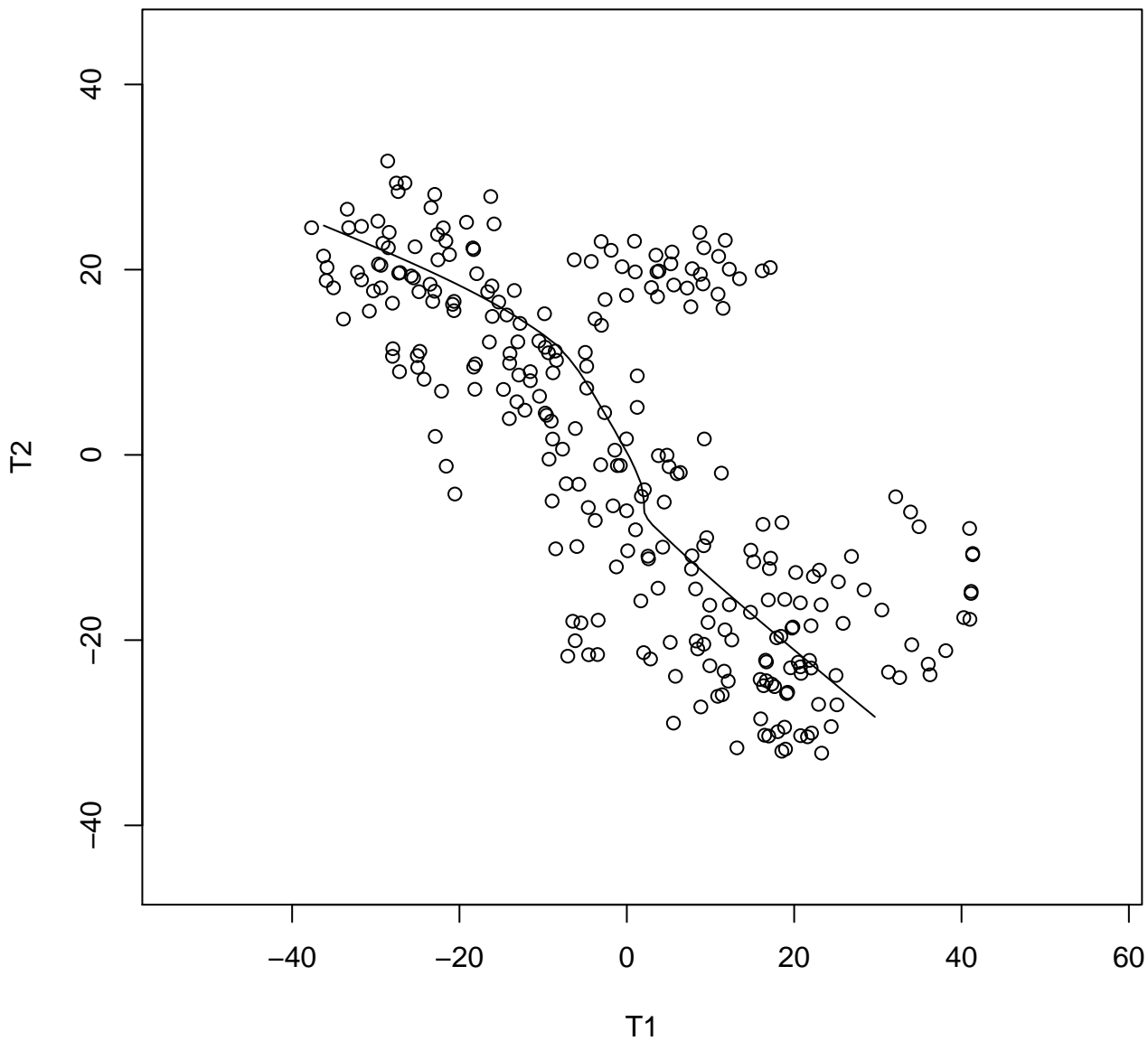

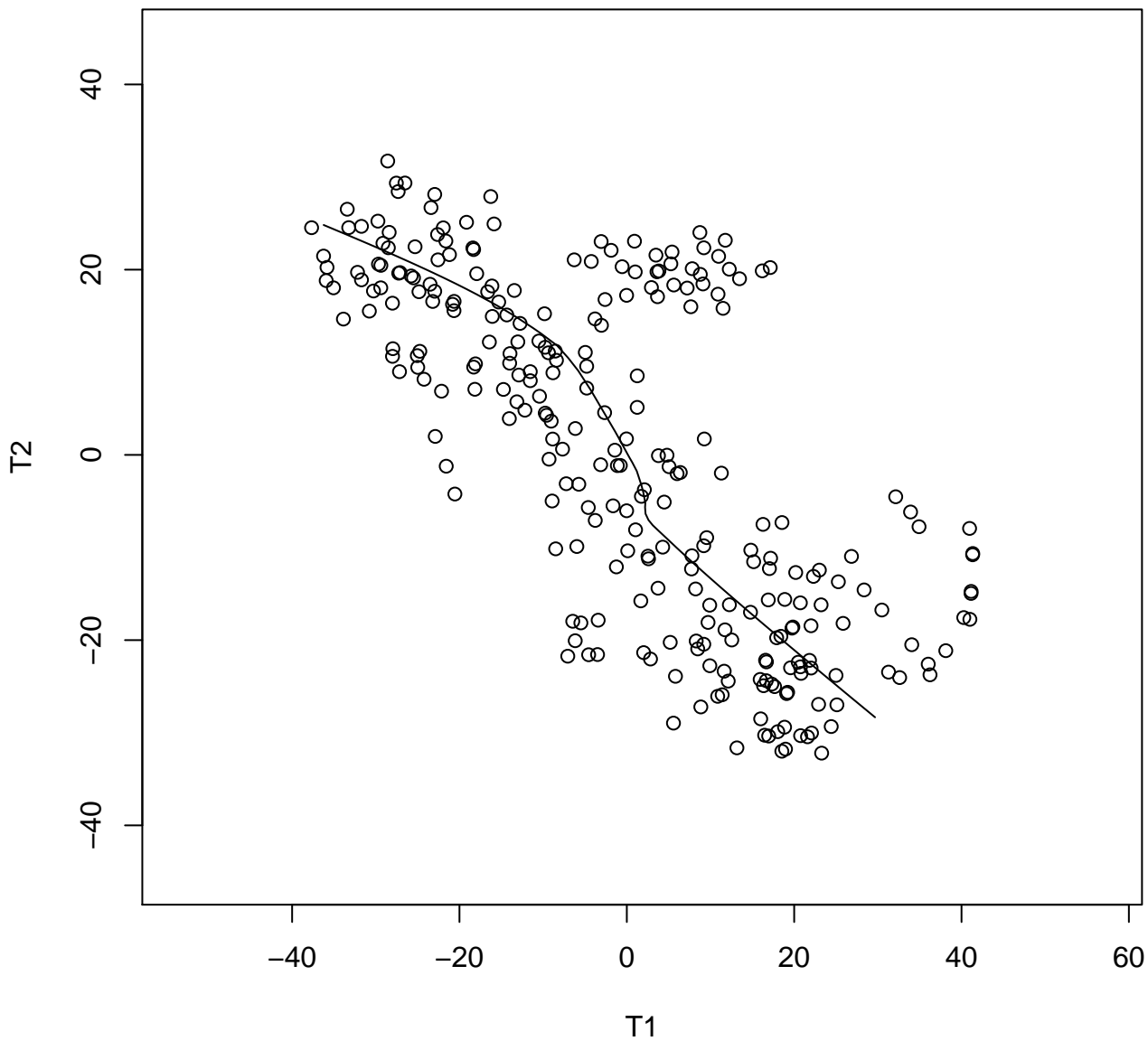

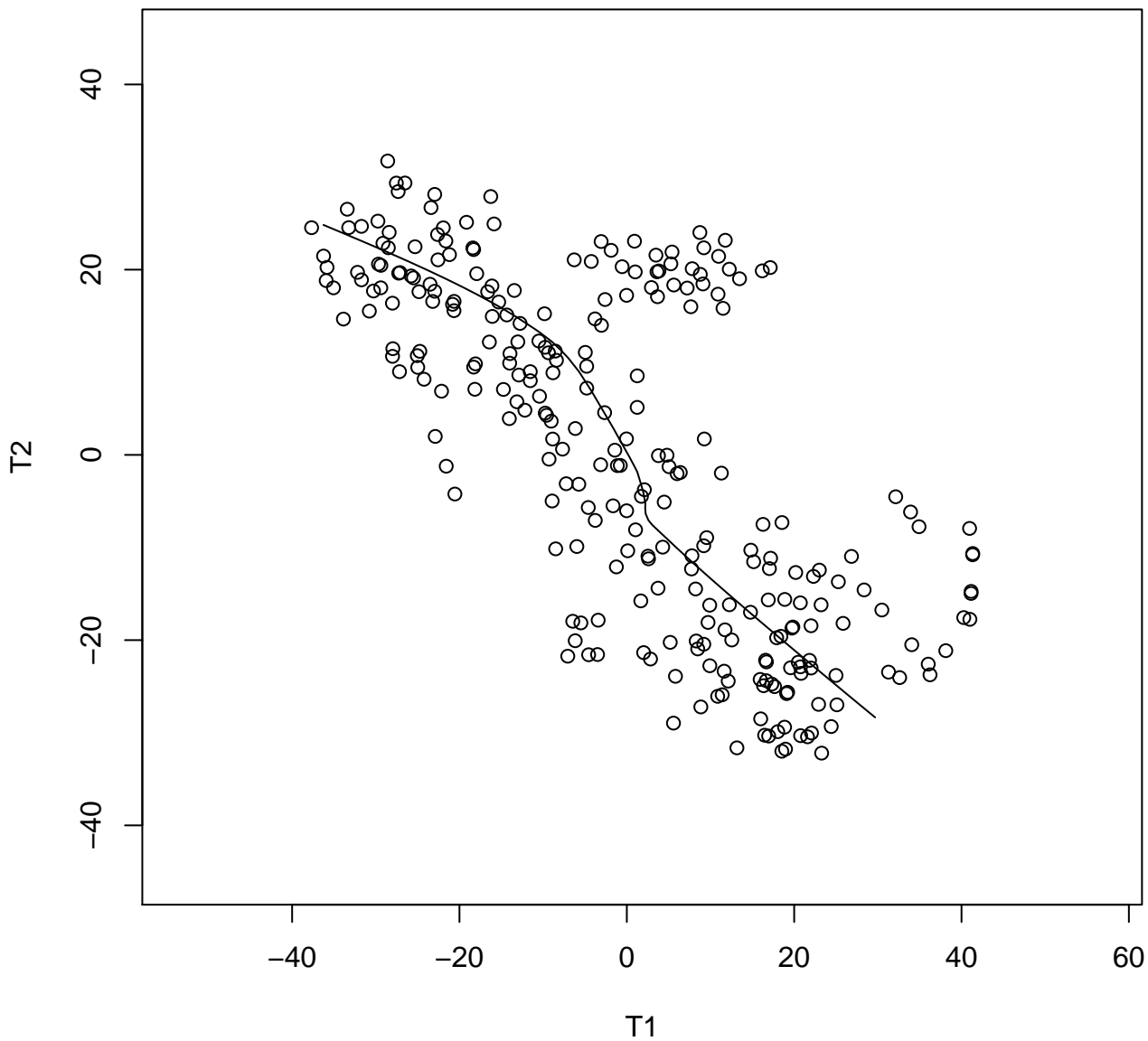

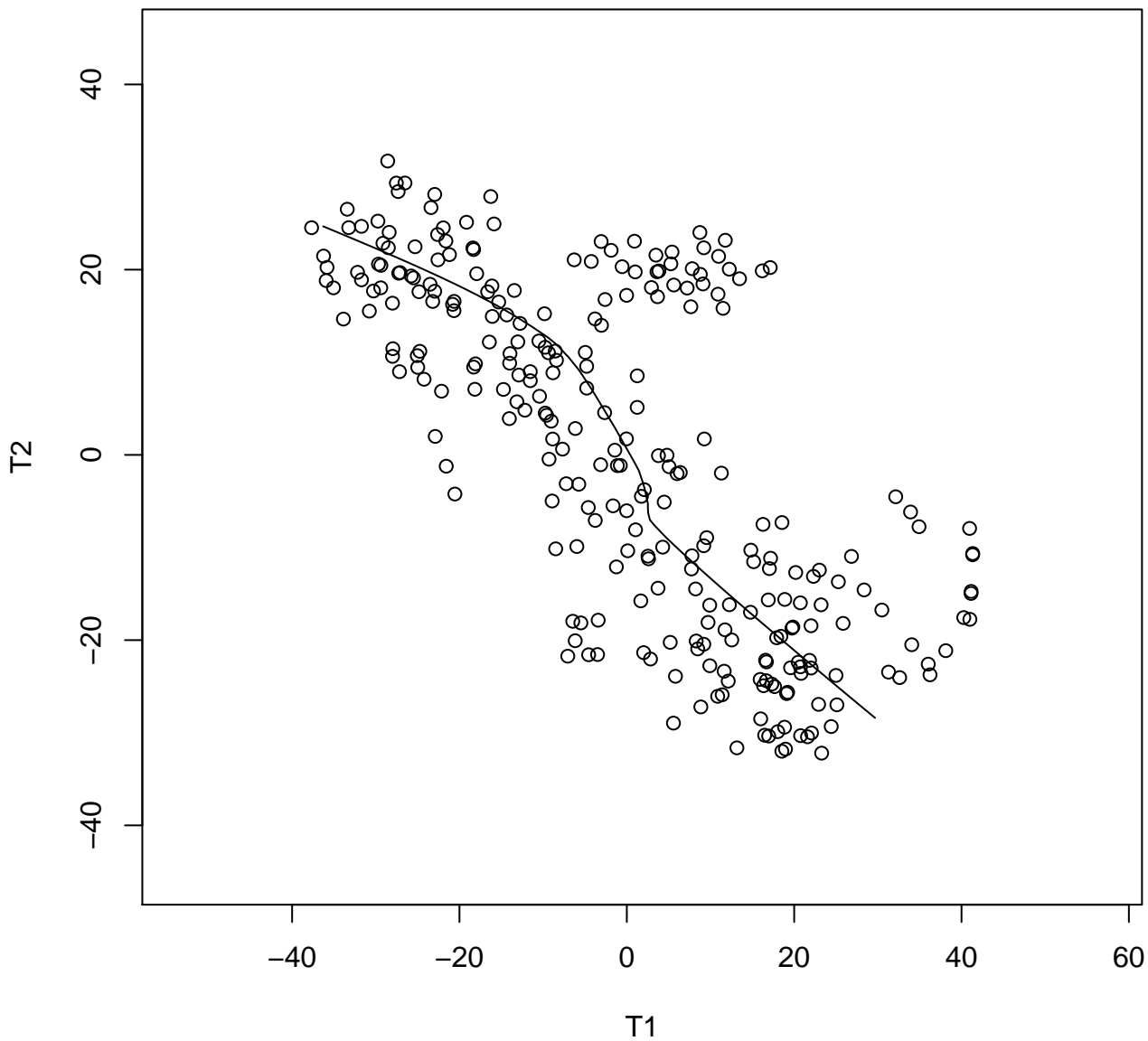

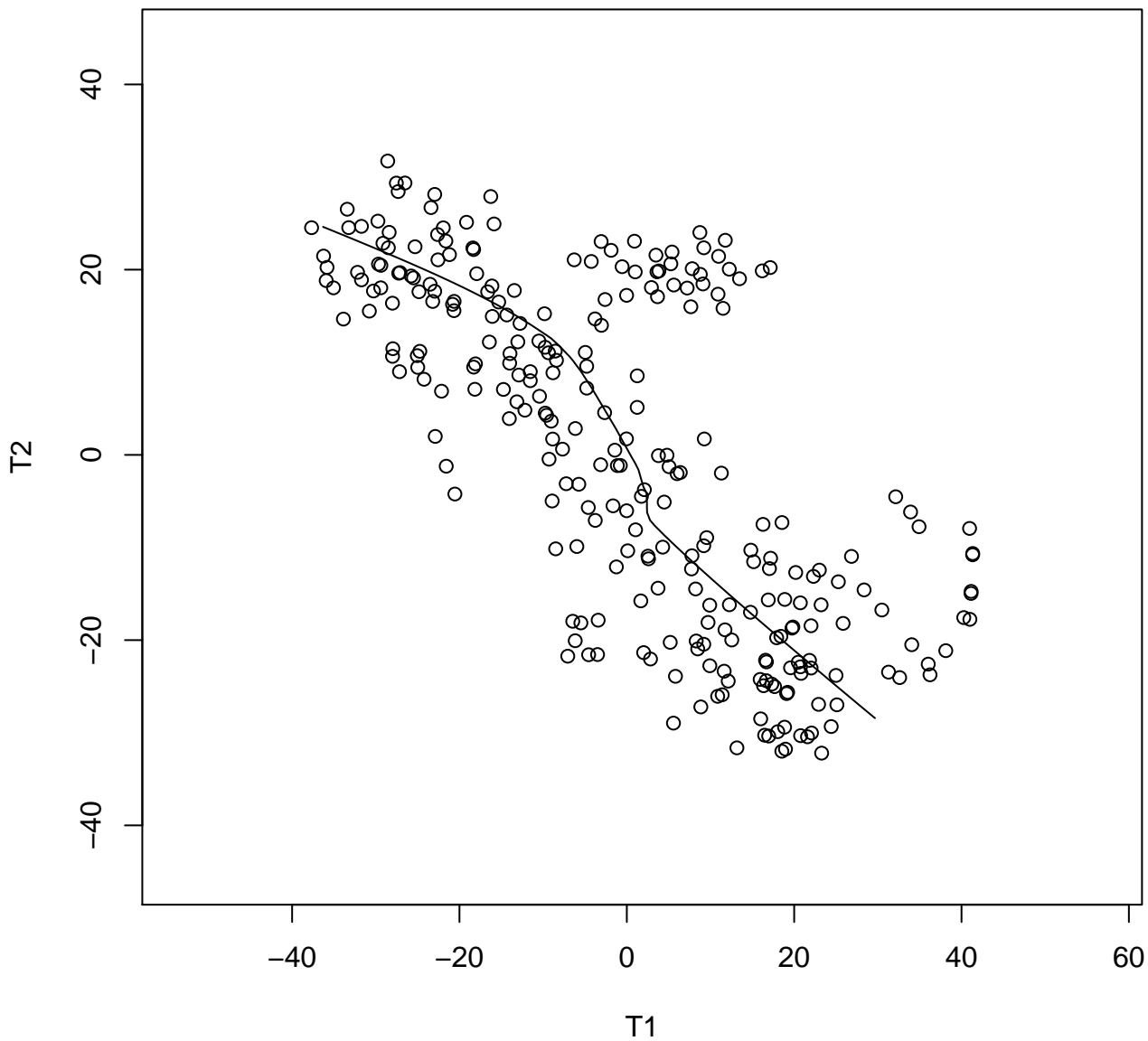

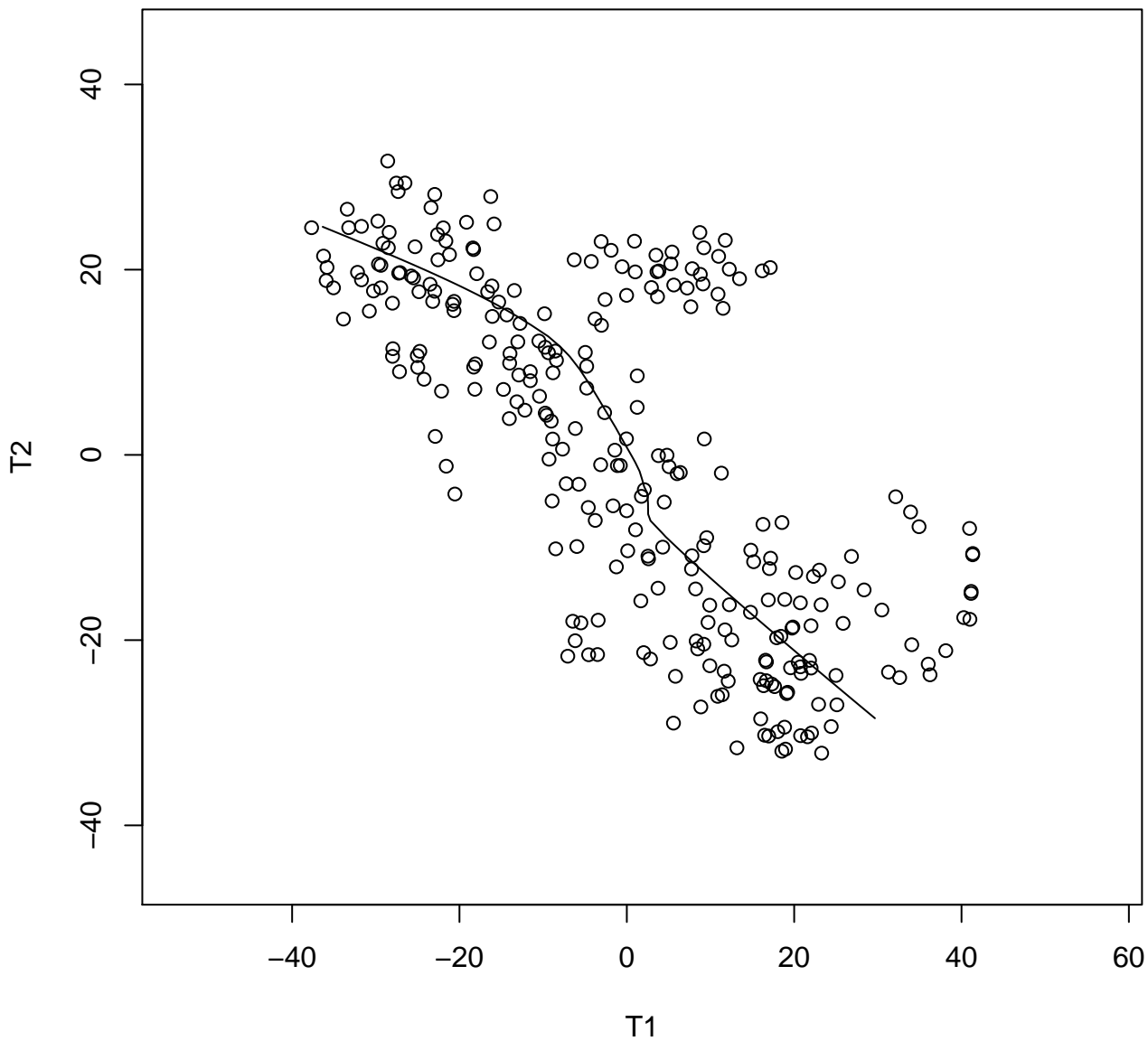

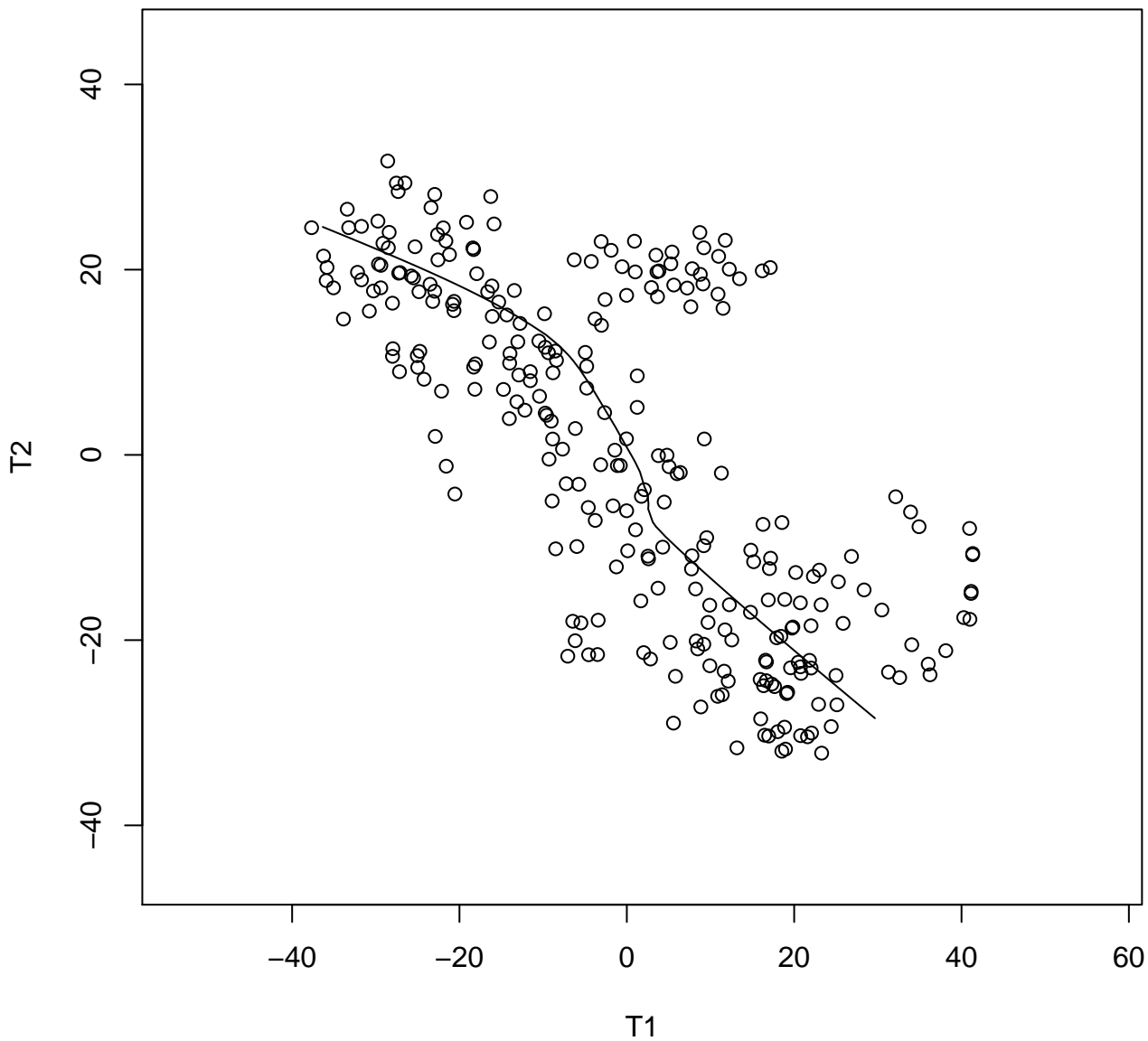

Supplement: Supplementary file 5 — Supplementary Data 2 [file 41467_2019_9670_MOESM5_ESM.zip › Sup_data2/Guo_2013/scuba/Result_run2/Rplots.pdf]

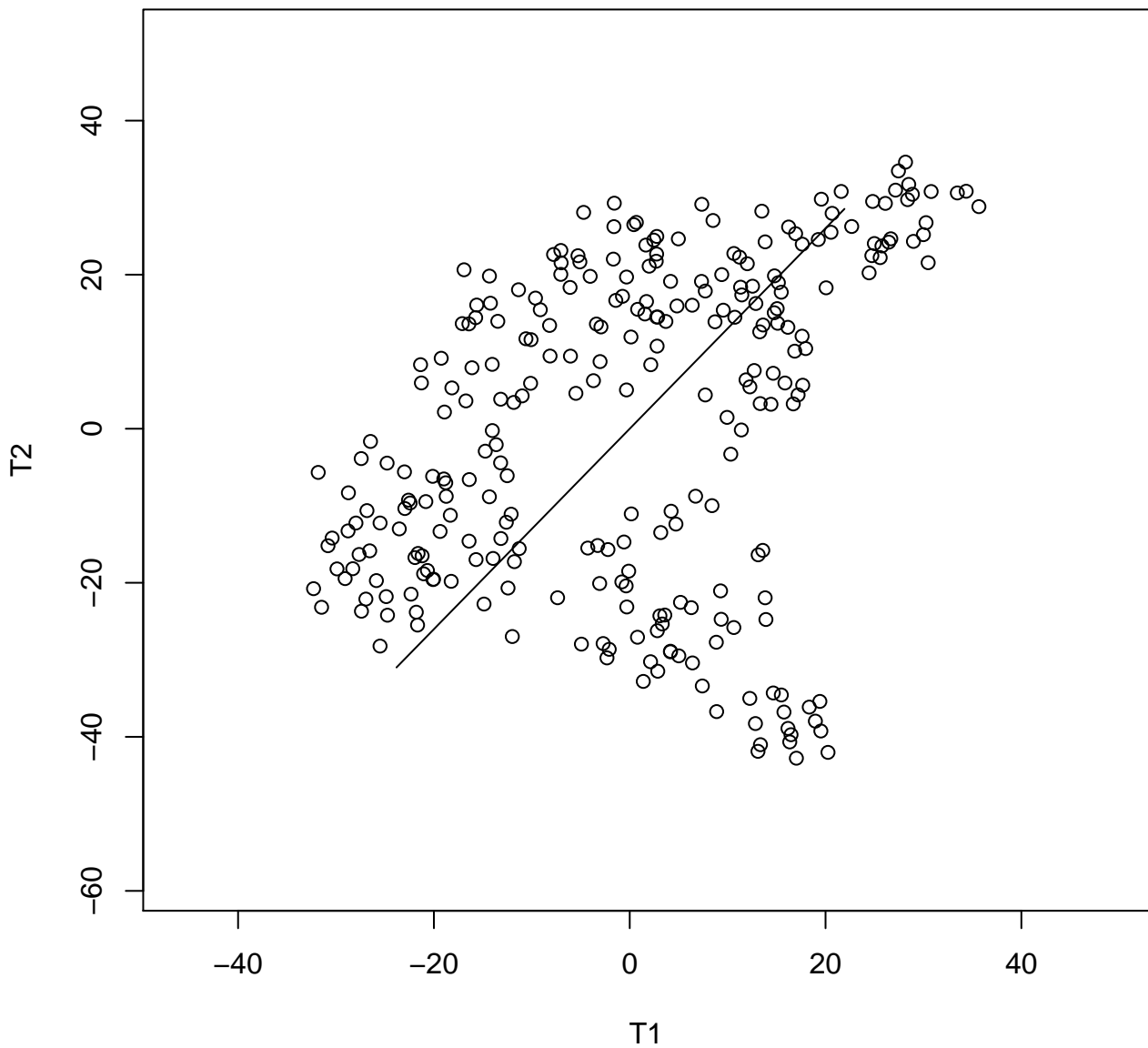

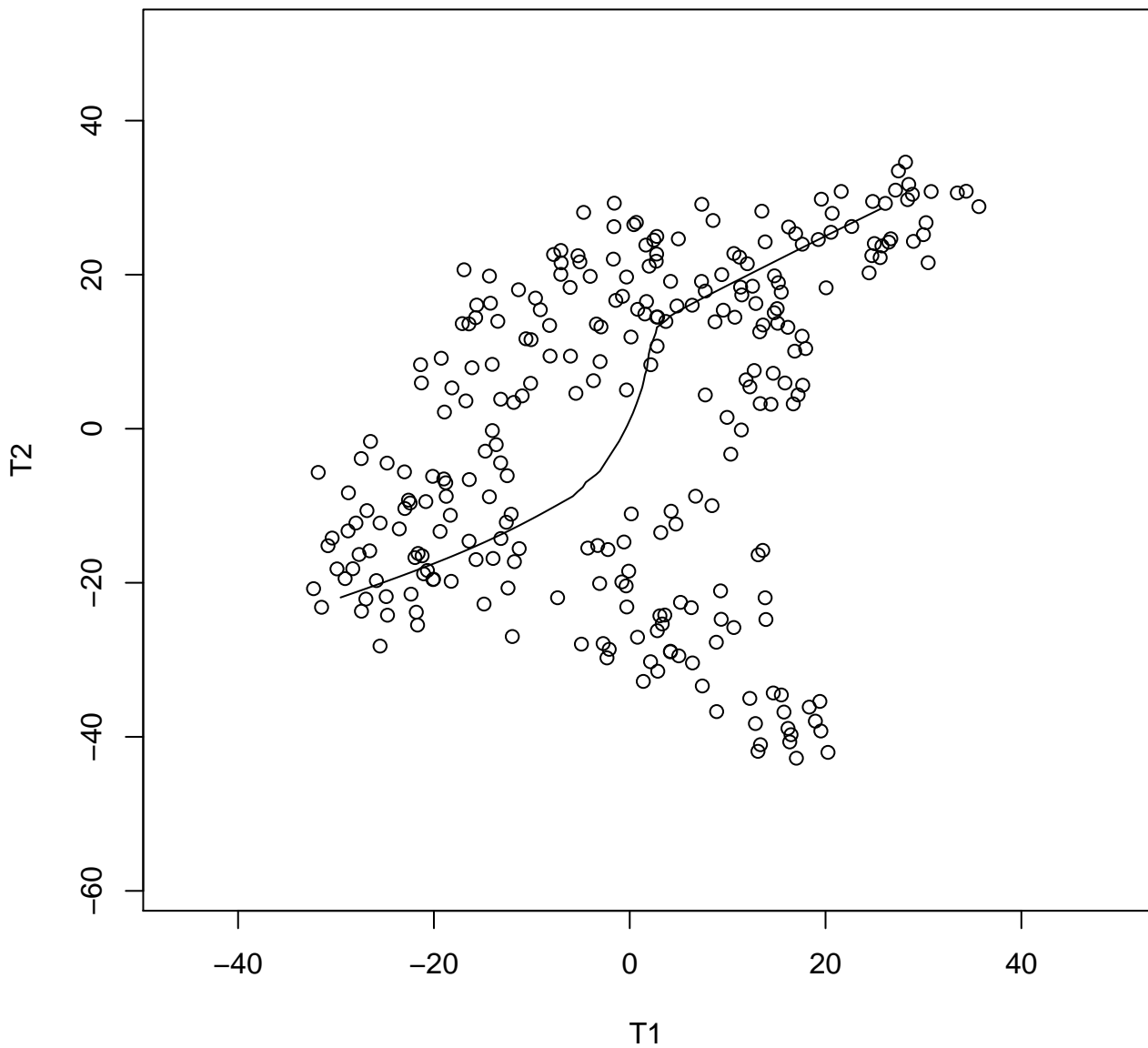

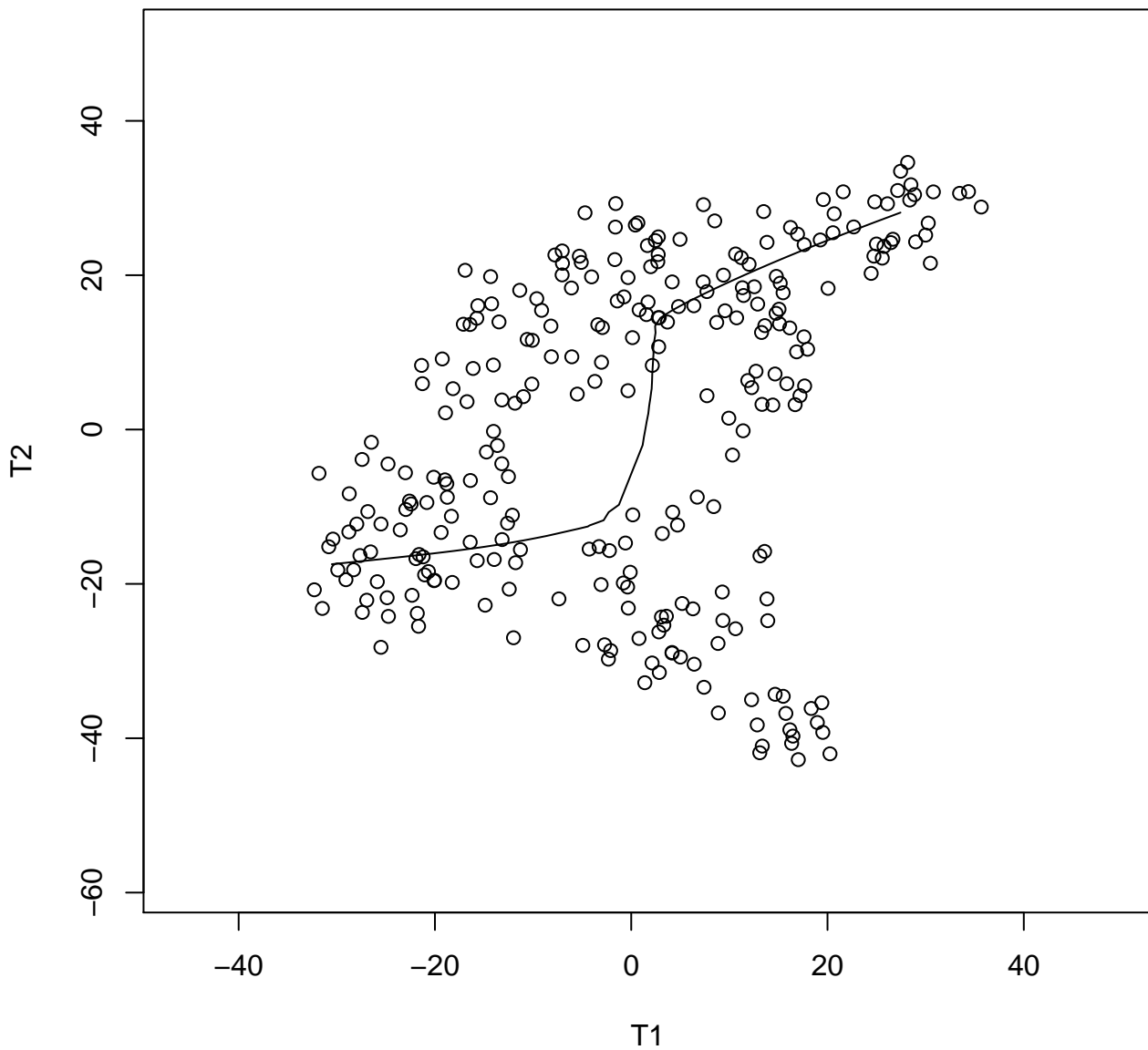

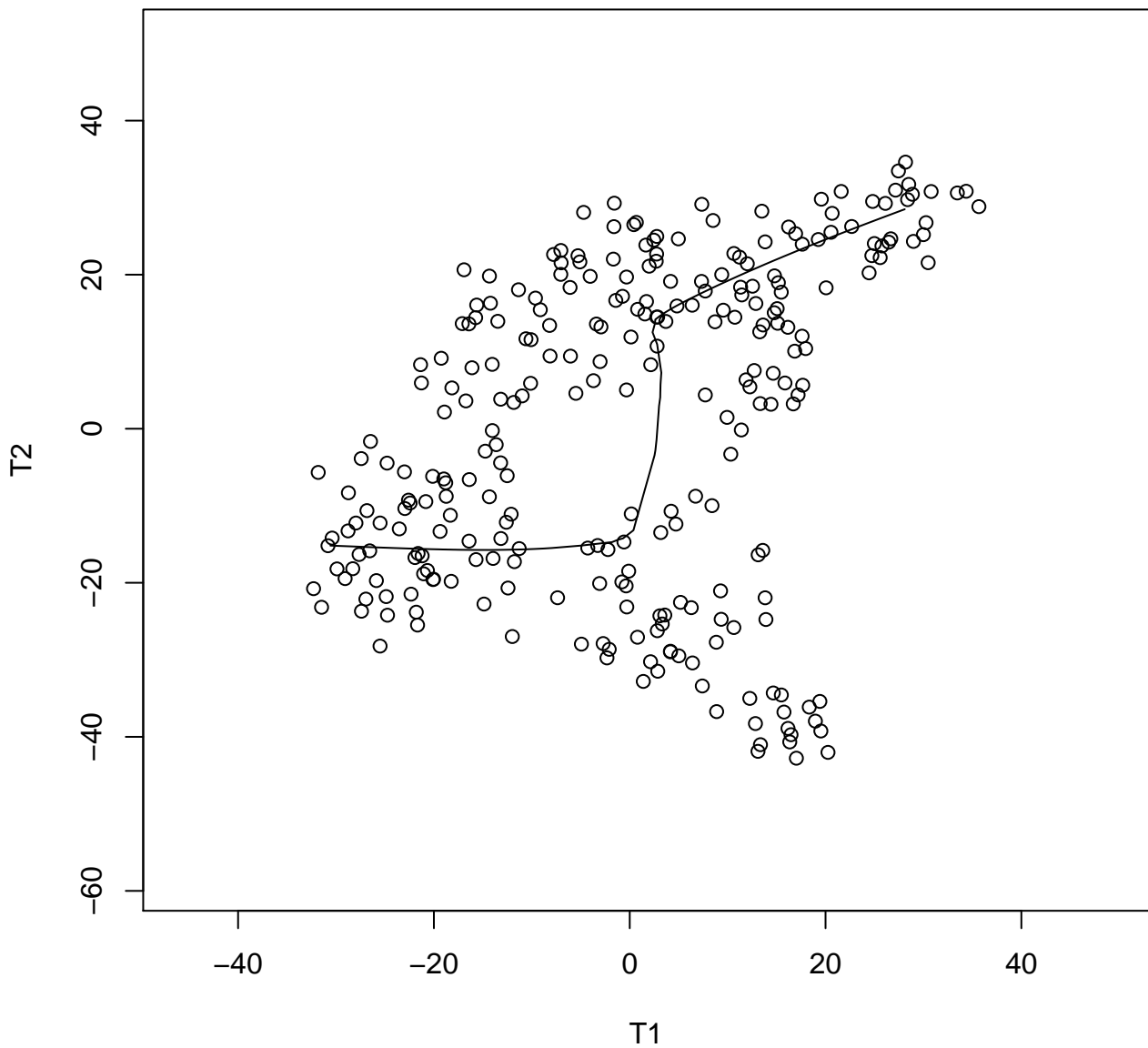

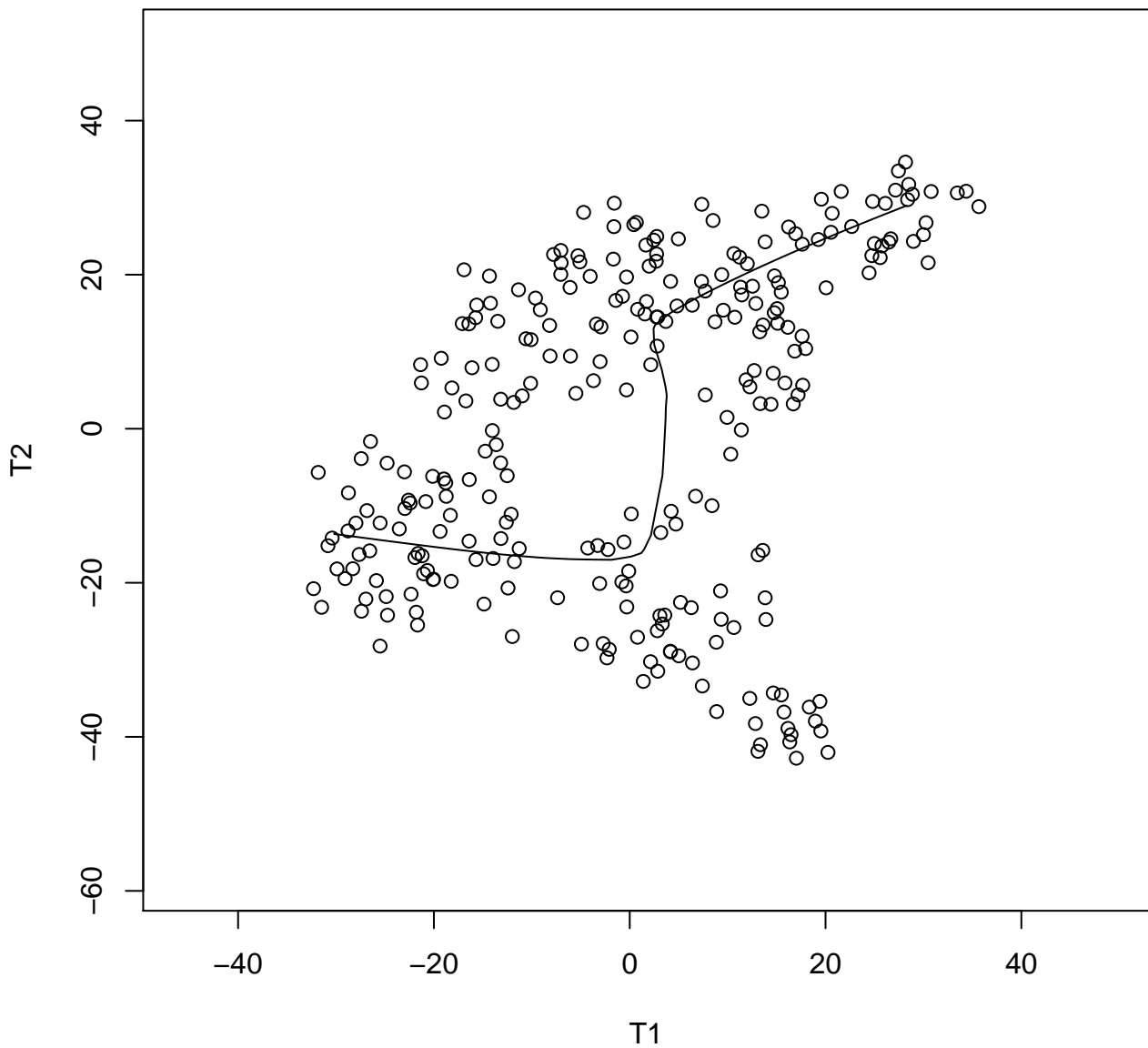

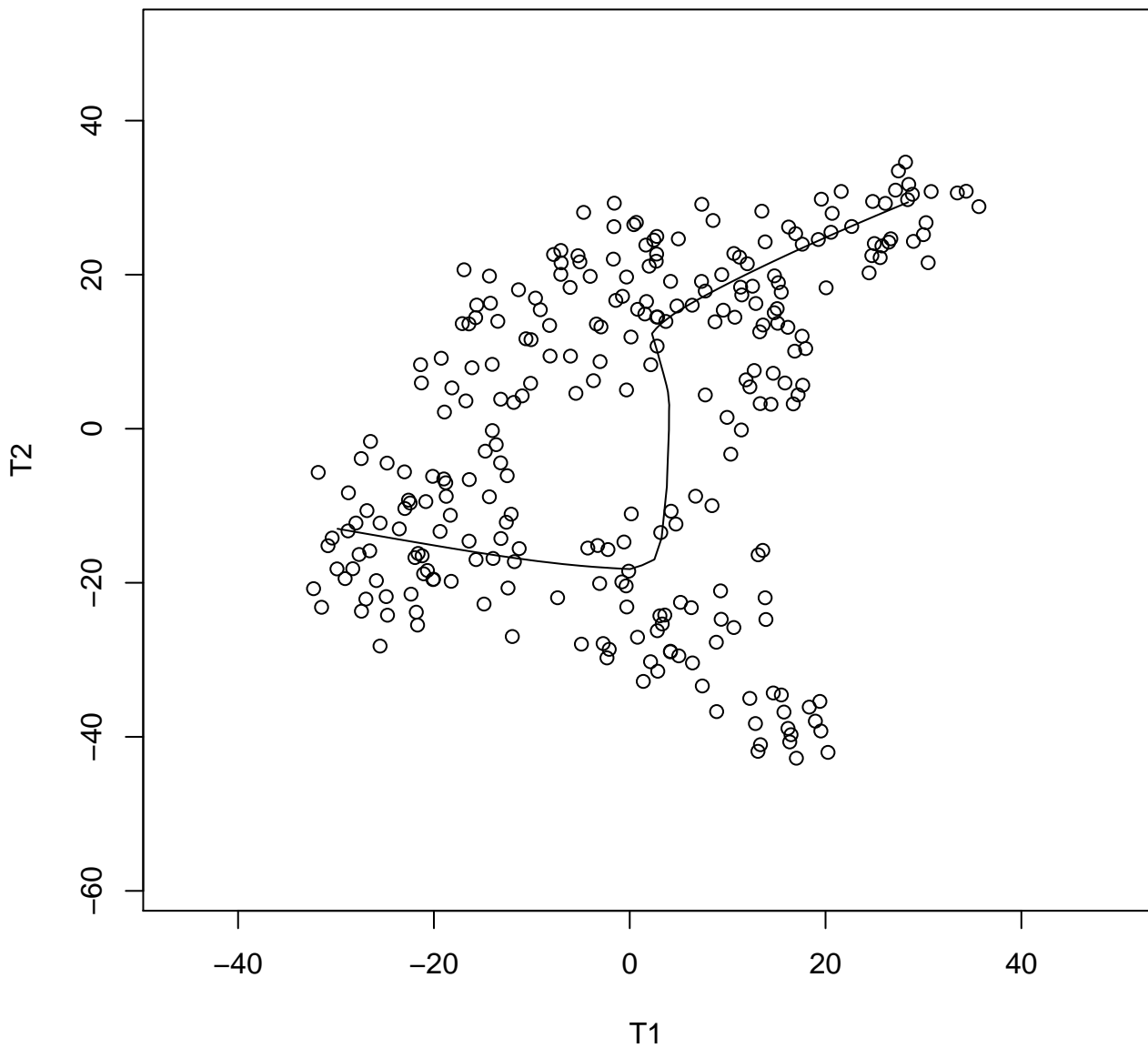

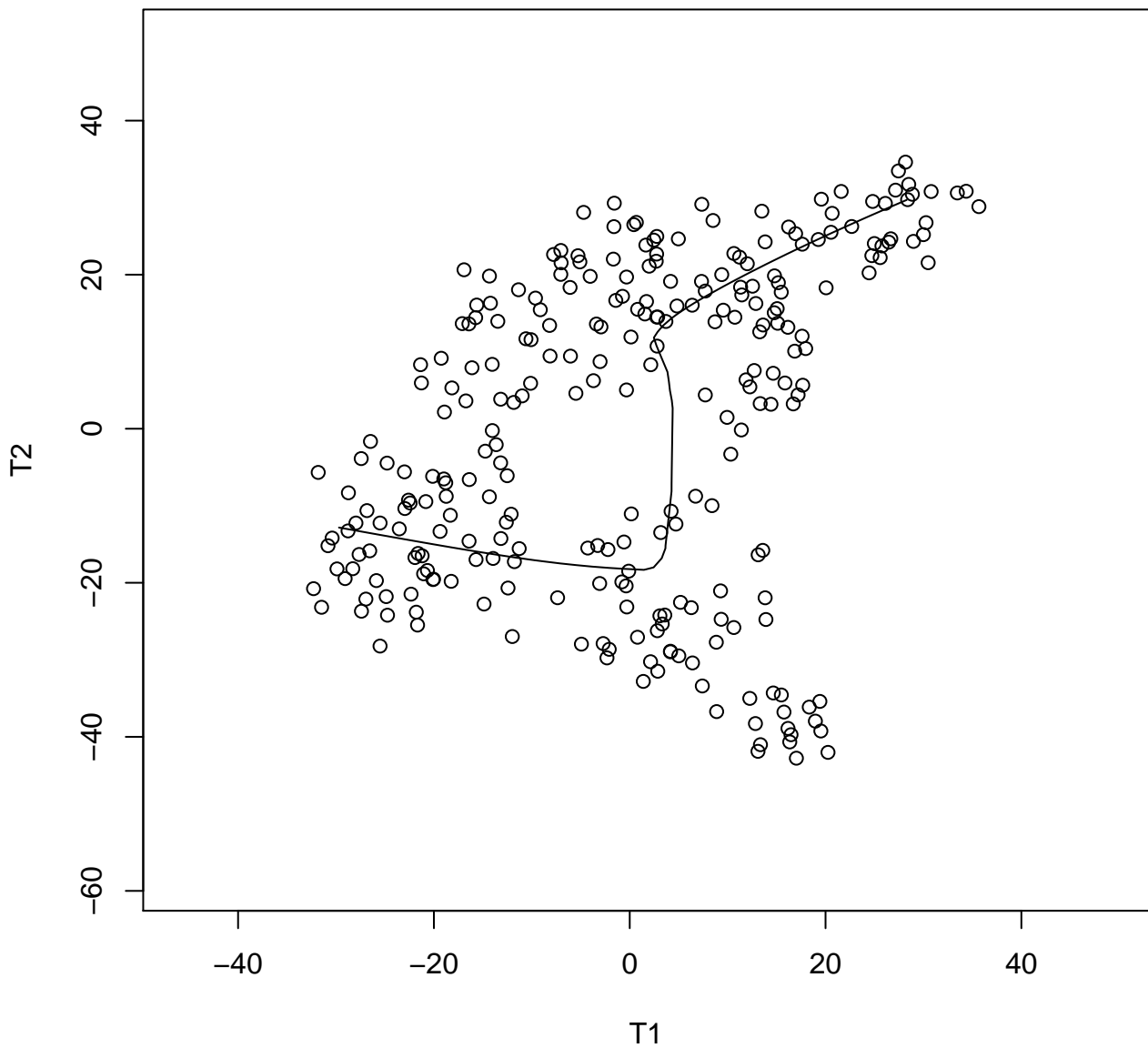

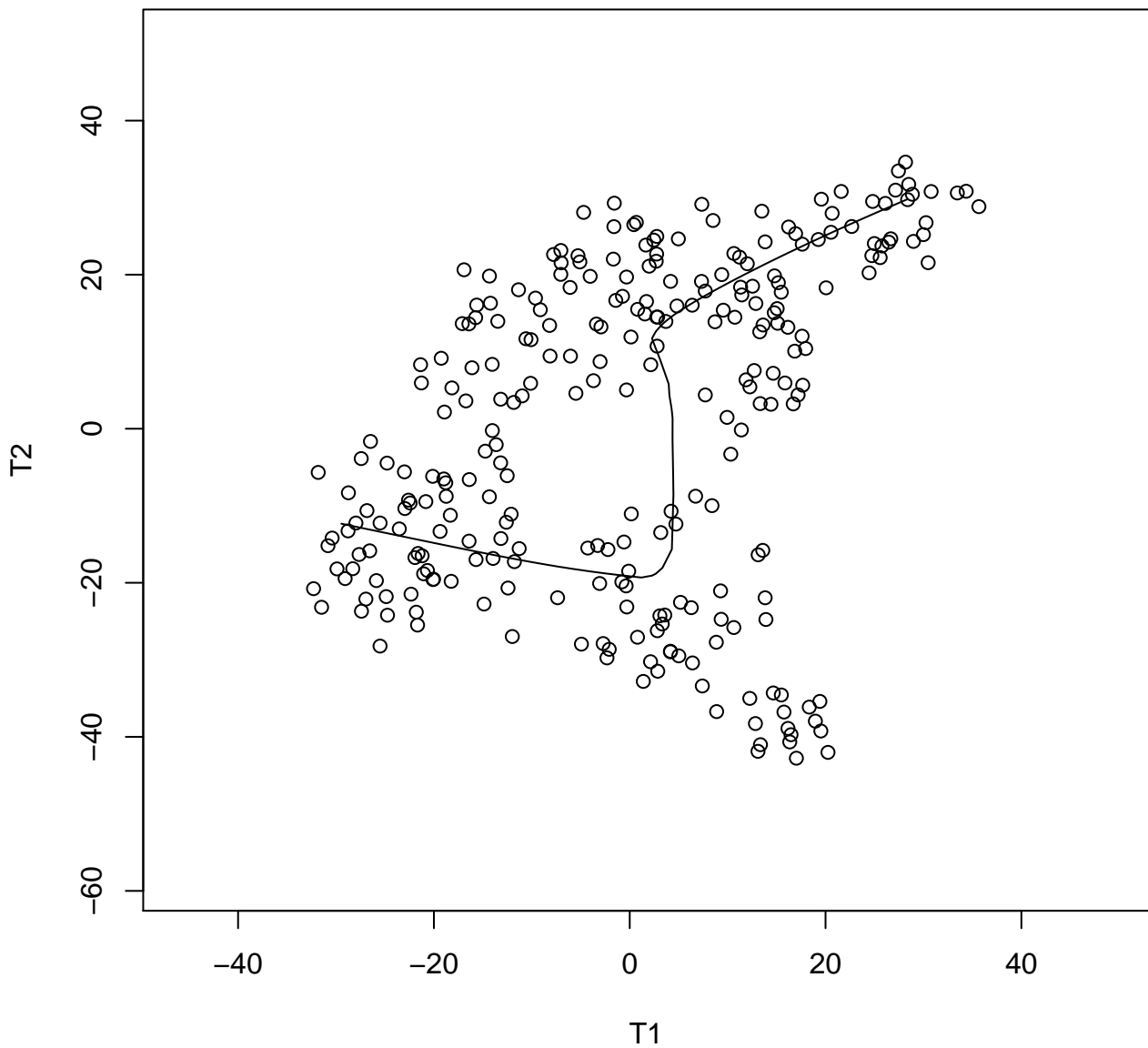

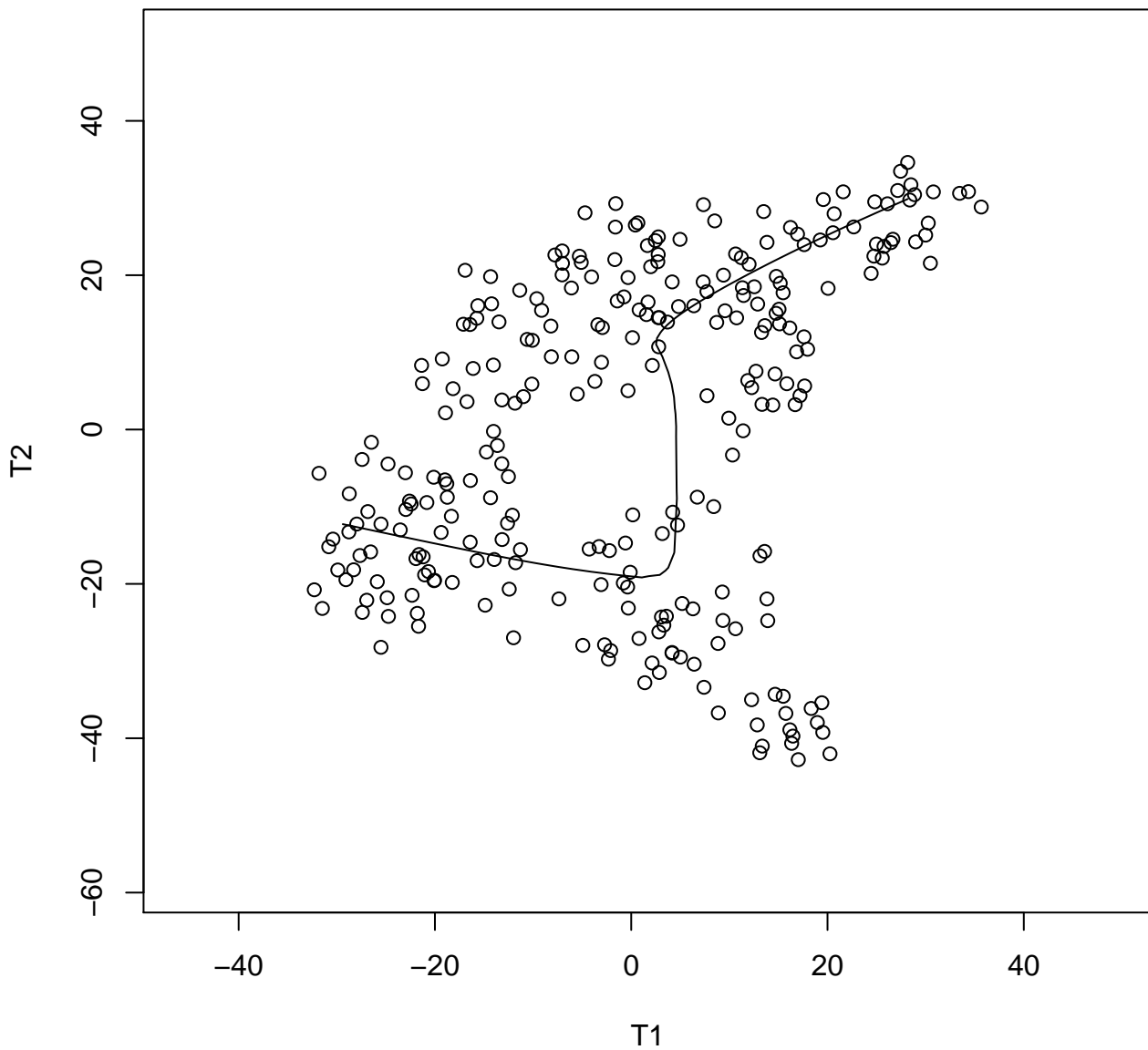

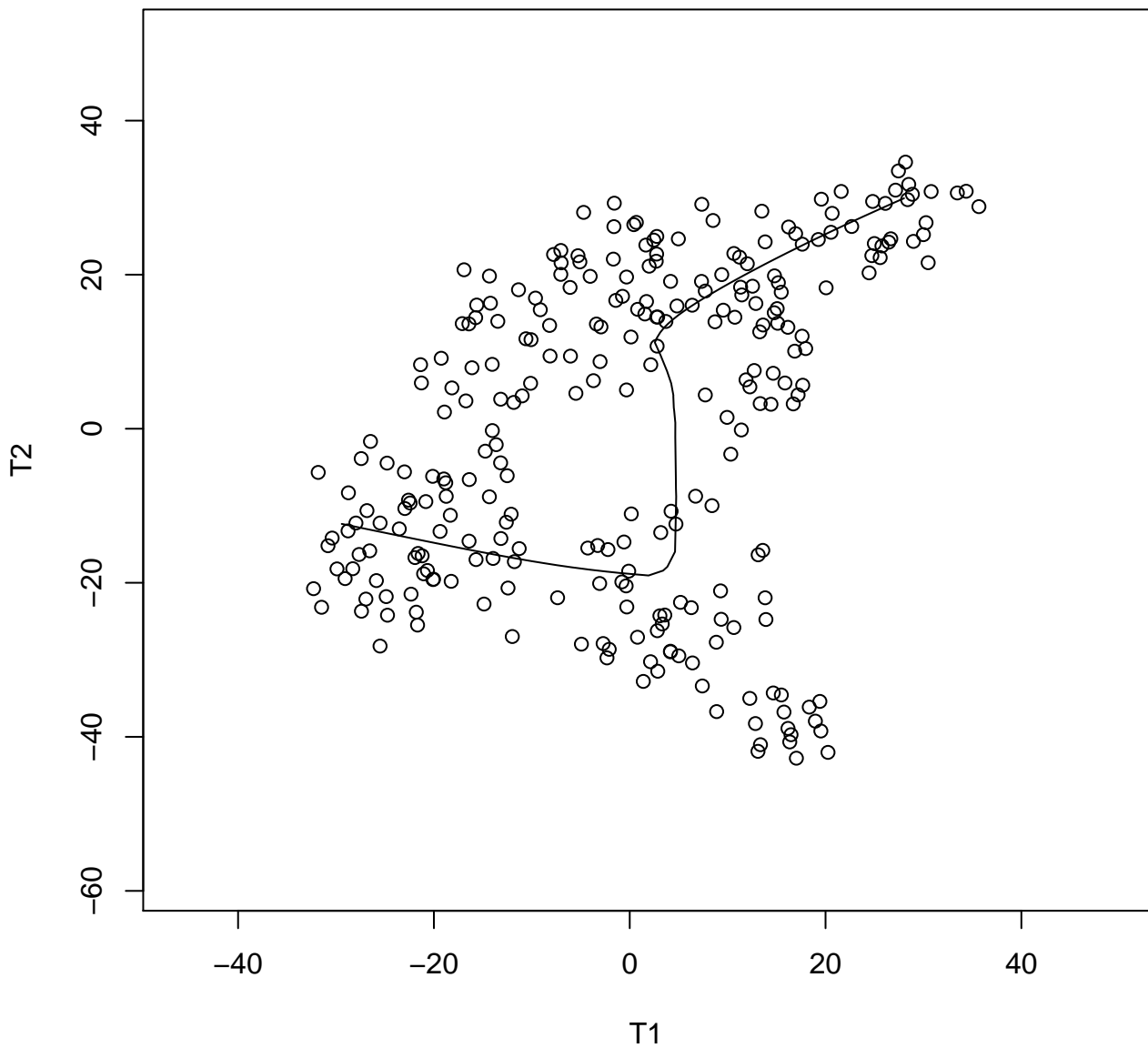

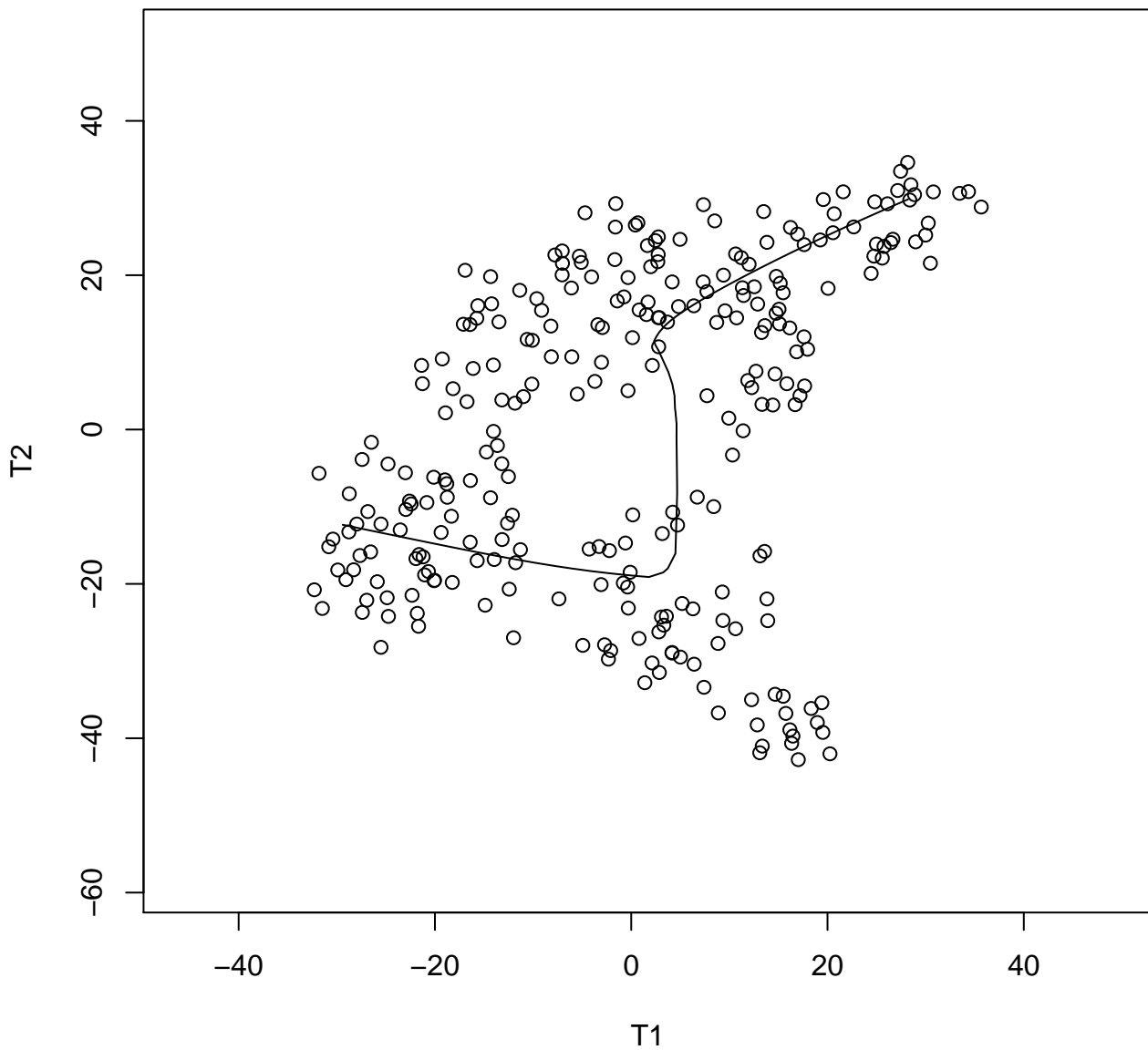

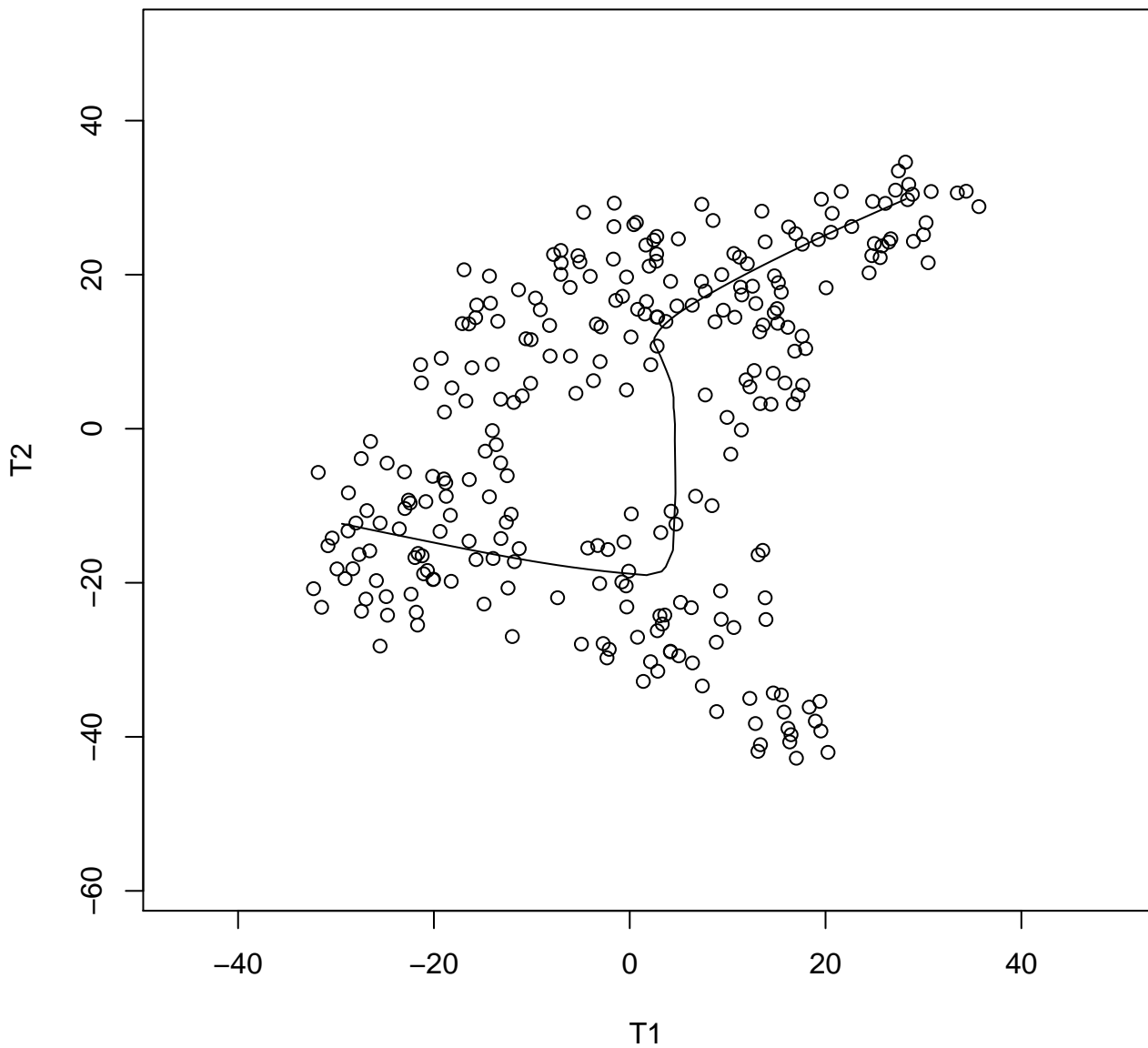

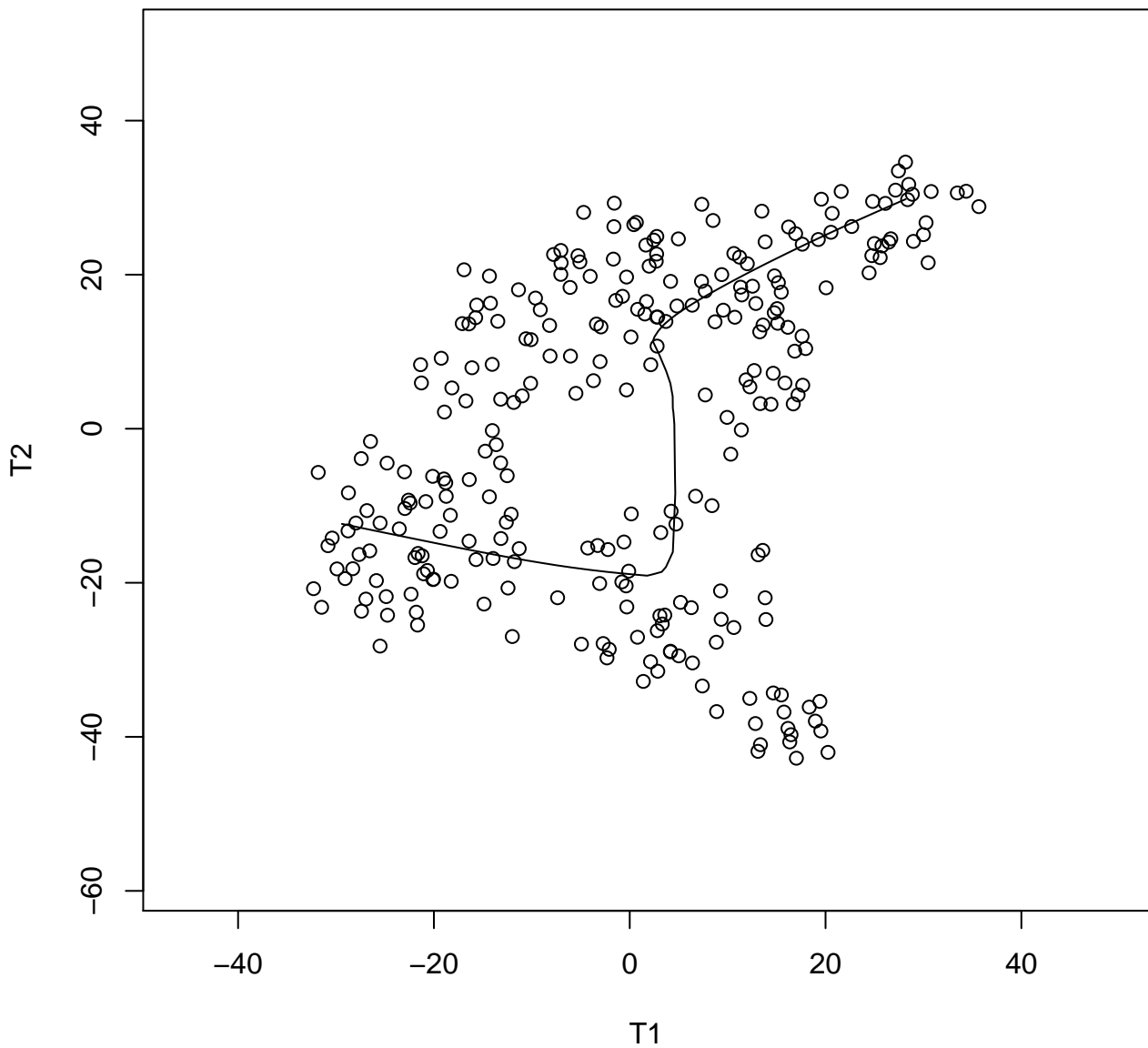

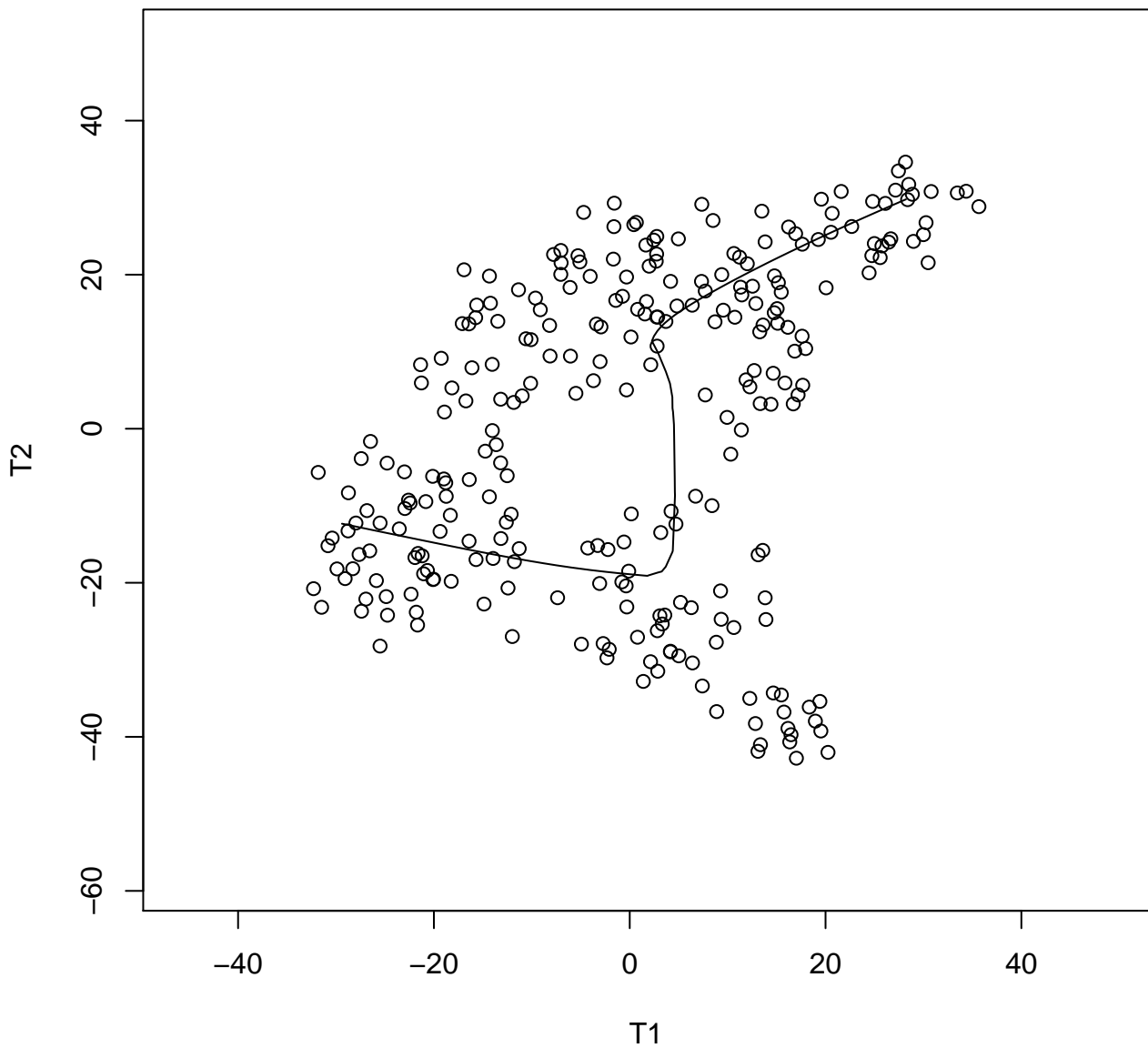

Supplement: Supplementary file 5 — Supplementary Data 2 [file 41467_2019_9670_MOESM5_ESM.zip › Sup_data2/Guo_2013/scuba/Rplots.pdf]

phate 2

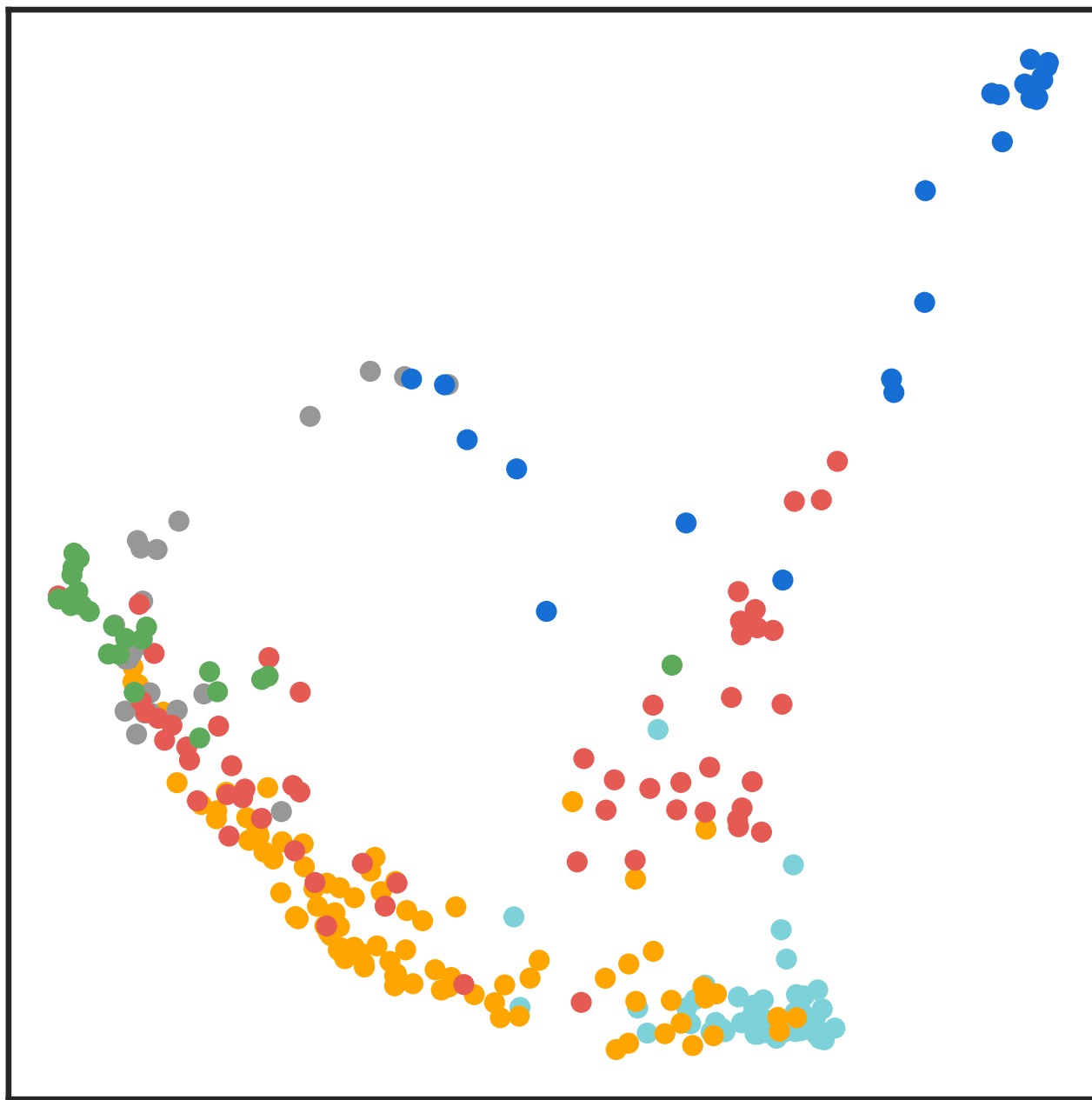

phate 1

Supplement: Supplementary file 5 — Supplementary Data 2 [file 41467_2019_9670_MOESM5_ESM.zip › Sup_data2/Guo_2013/phate/phate_guo2013.pdf]

dpt pseudotime

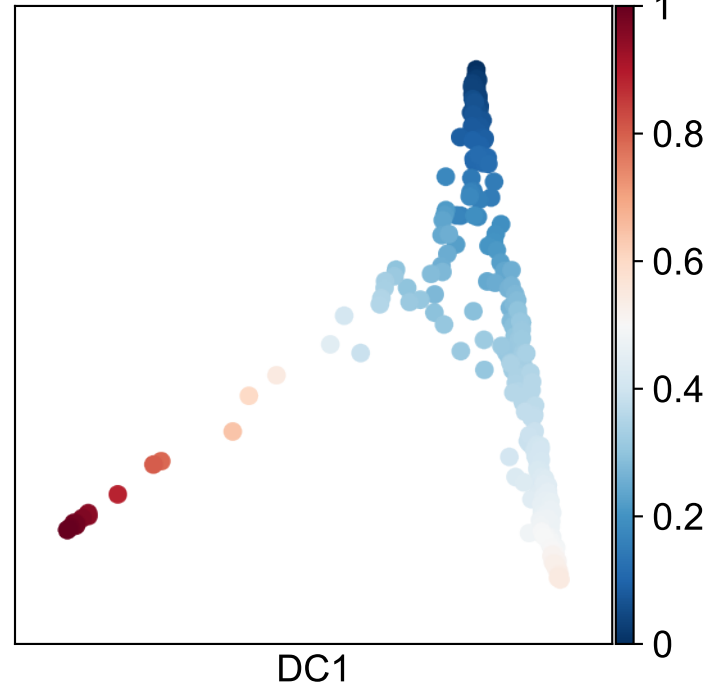

dpt groups

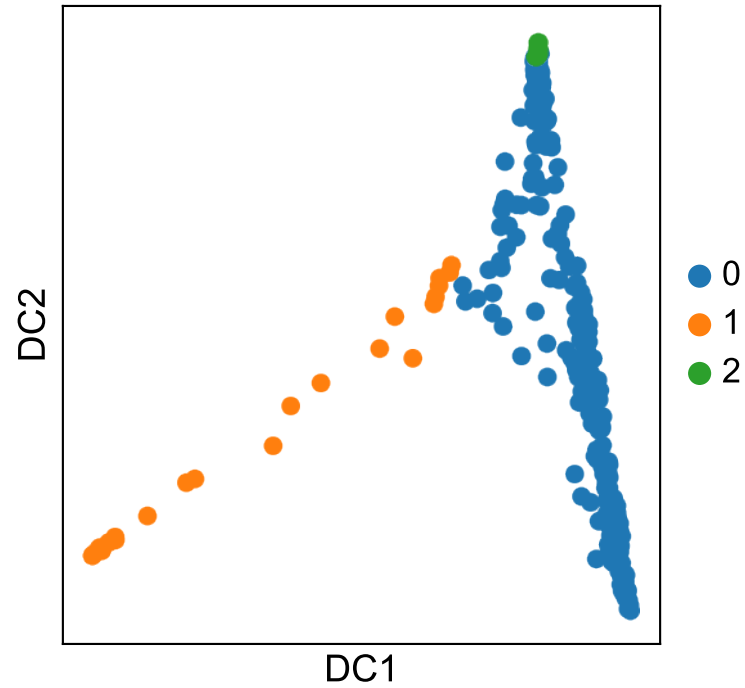

label

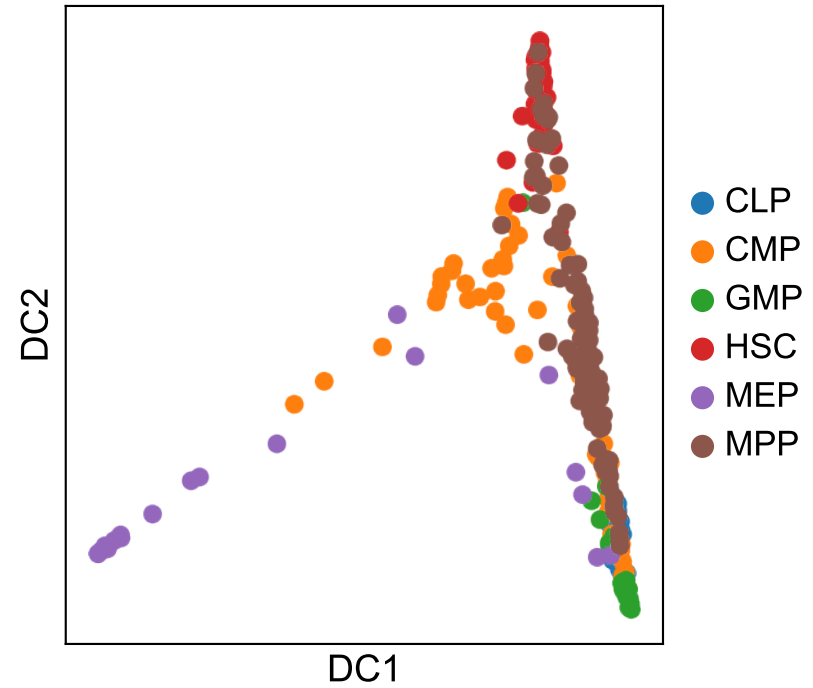

Supplement: Supplementary file 5 — Supplementary Data 2 [file 41467_2019_9670_MOESM5_ESM.zip › Sup_data2/Guo_2013/dpt/figures/diffmap_components12.pdf]

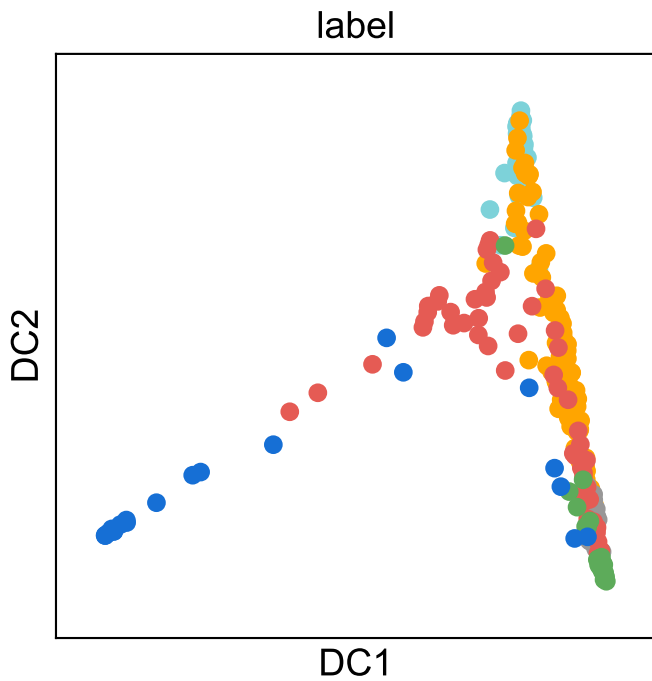

Supplement: Supplementary file 5 — Supplementary Data 2 [file 41467_2019_9670_MOESM5_ESM.zip › Sup_data2/Guo_2013/dpt/dpt_guo2013_labels.pdf]

# CD55

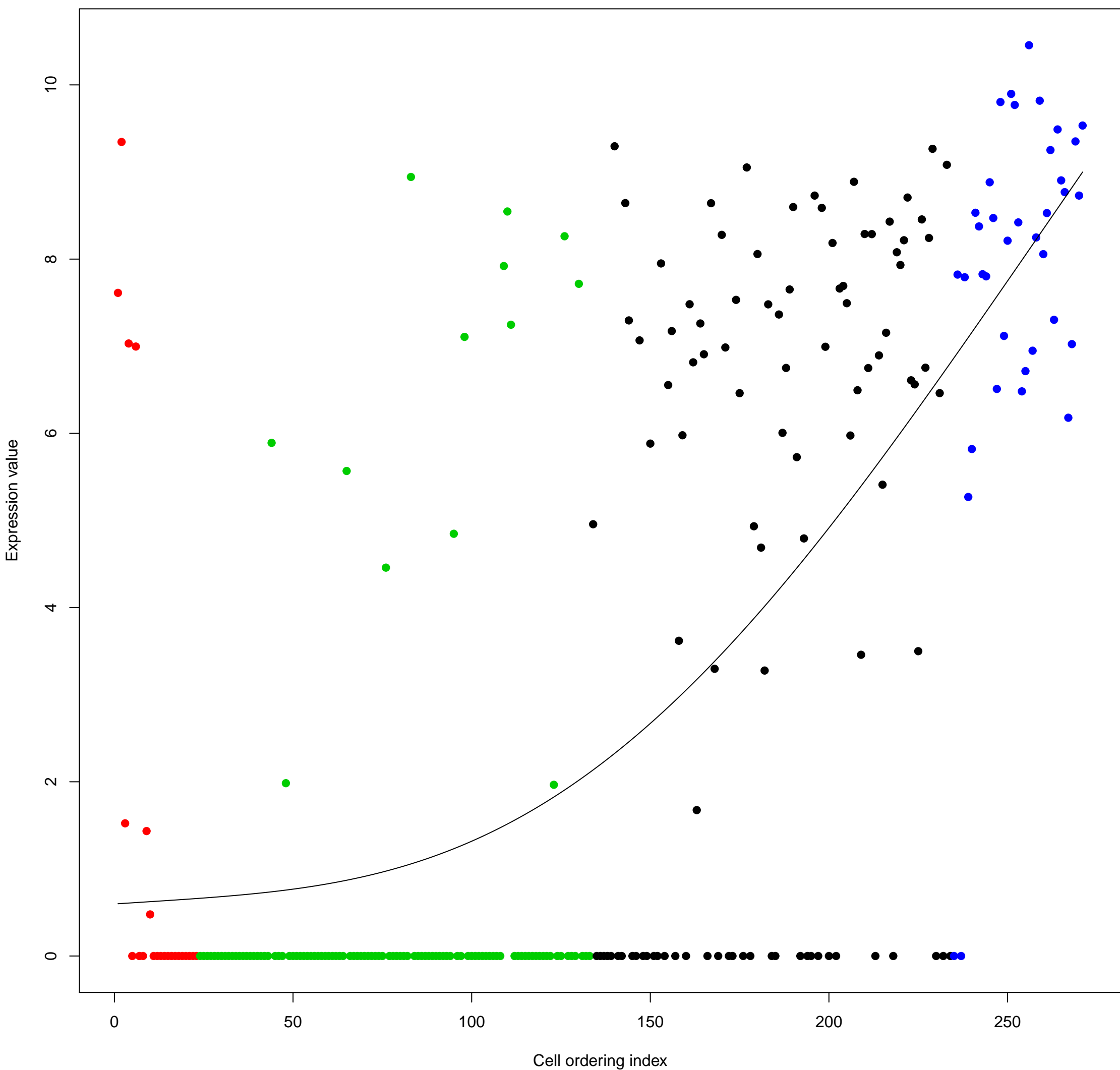

Supplement: Supplementary file 5 — Supplementary Data 2 [file 41467_2019_9670_MOESM5_ESM.zip › Sup_data2/Guo_2013/tscan/CD55_single_cell.pdf]

State 1 2 3 4

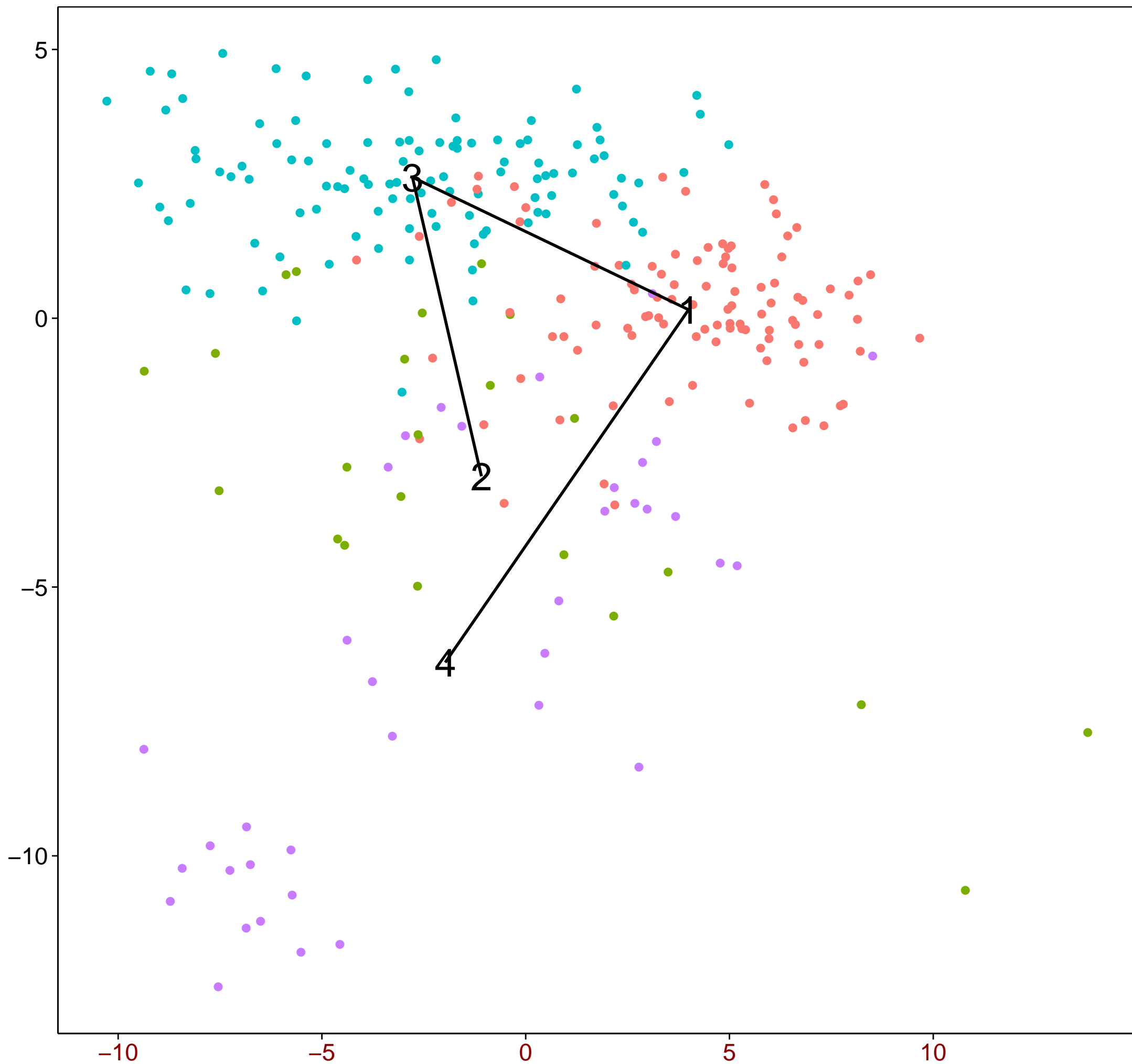

Supplement: Supplementary file 5 — Supplementary Data 2 [file 41467_2019_9670_MOESM5_ESM.zip › Sup_data2/Guo_2013/tscan/Cell_ordering.pdf]

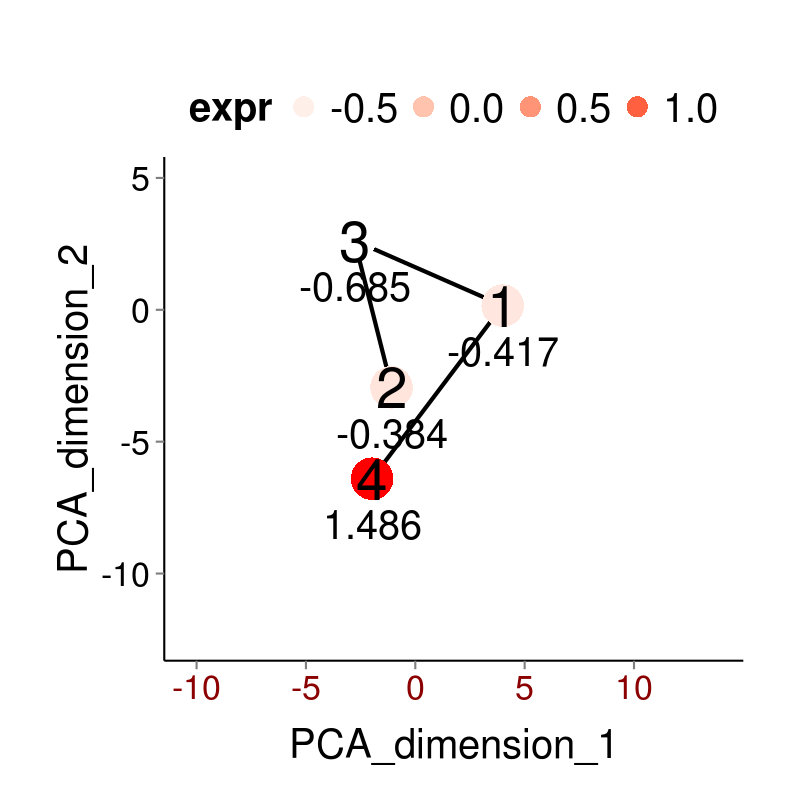

Supplement: Supplementary file 5 — Supplementary Data 2 [file 41467_2019_9670_MOESM5_ESM.zip › Sup_data2/Guo_2013/tscan/Gata1.png]

Gata1

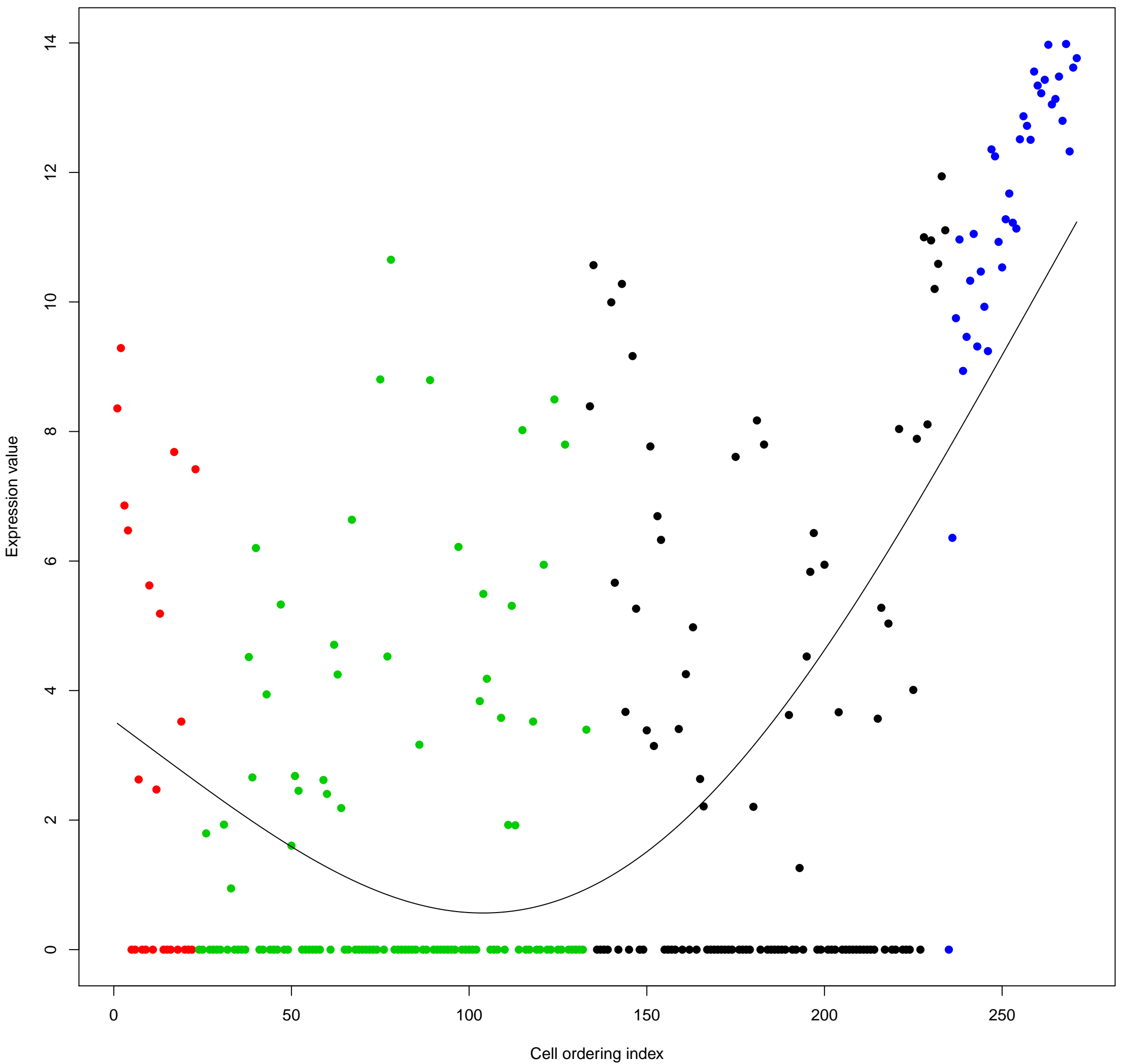

Supplement: Supplementary file 5 — Supplementary Data 2 [file 41467_2019_9670_MOESM5_ESM.zip › Sup_data2/Guo_2013/tscan/Gata1_single_cell.pdf]

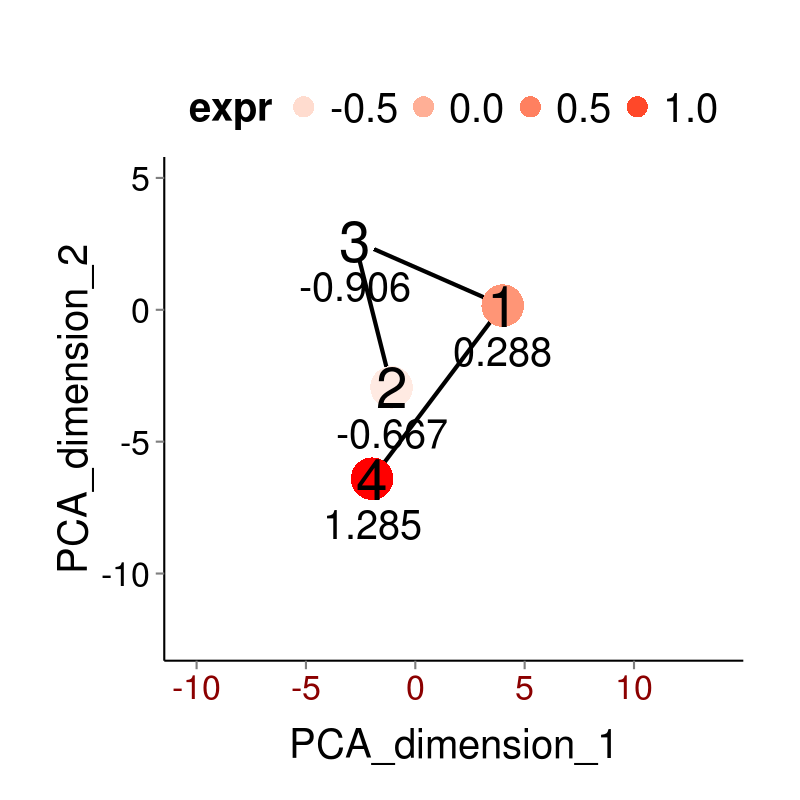

Supplement: Supplementary file 5 — Supplementary Data 2 [file 41467_2019_9670_MOESM5_ESM.zip › Sup_data2/Guo_2013/tscan/CD55.png]

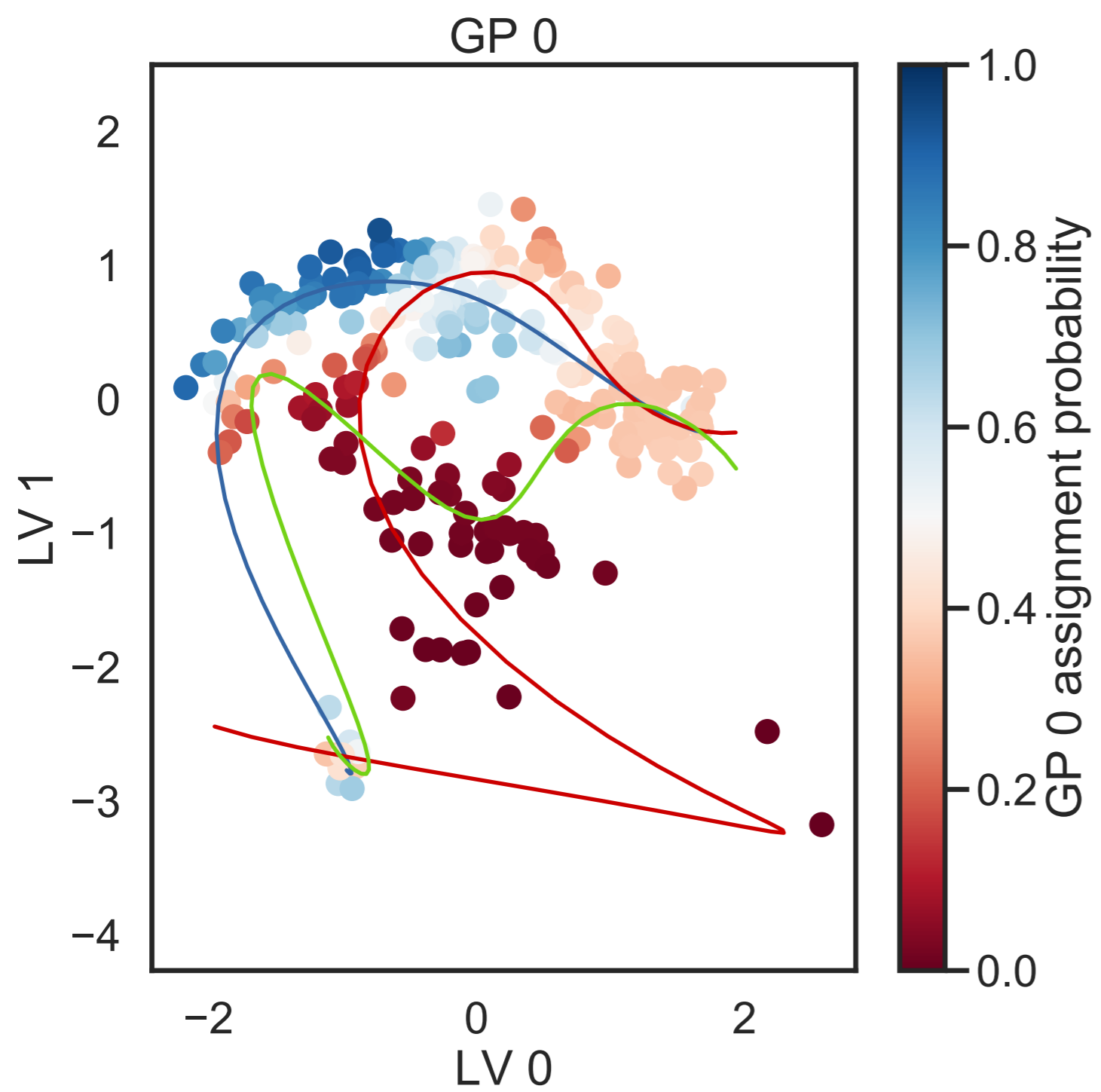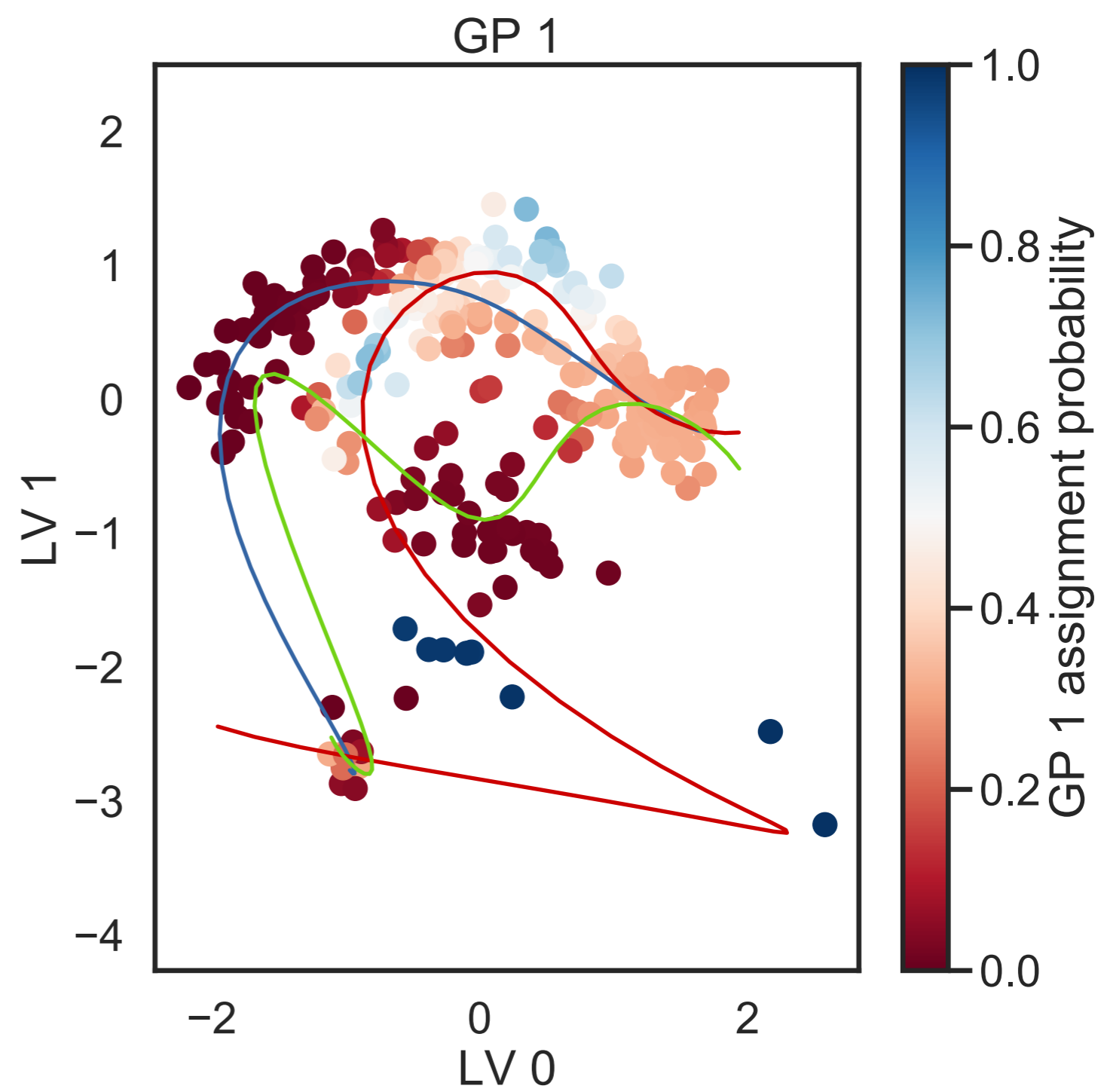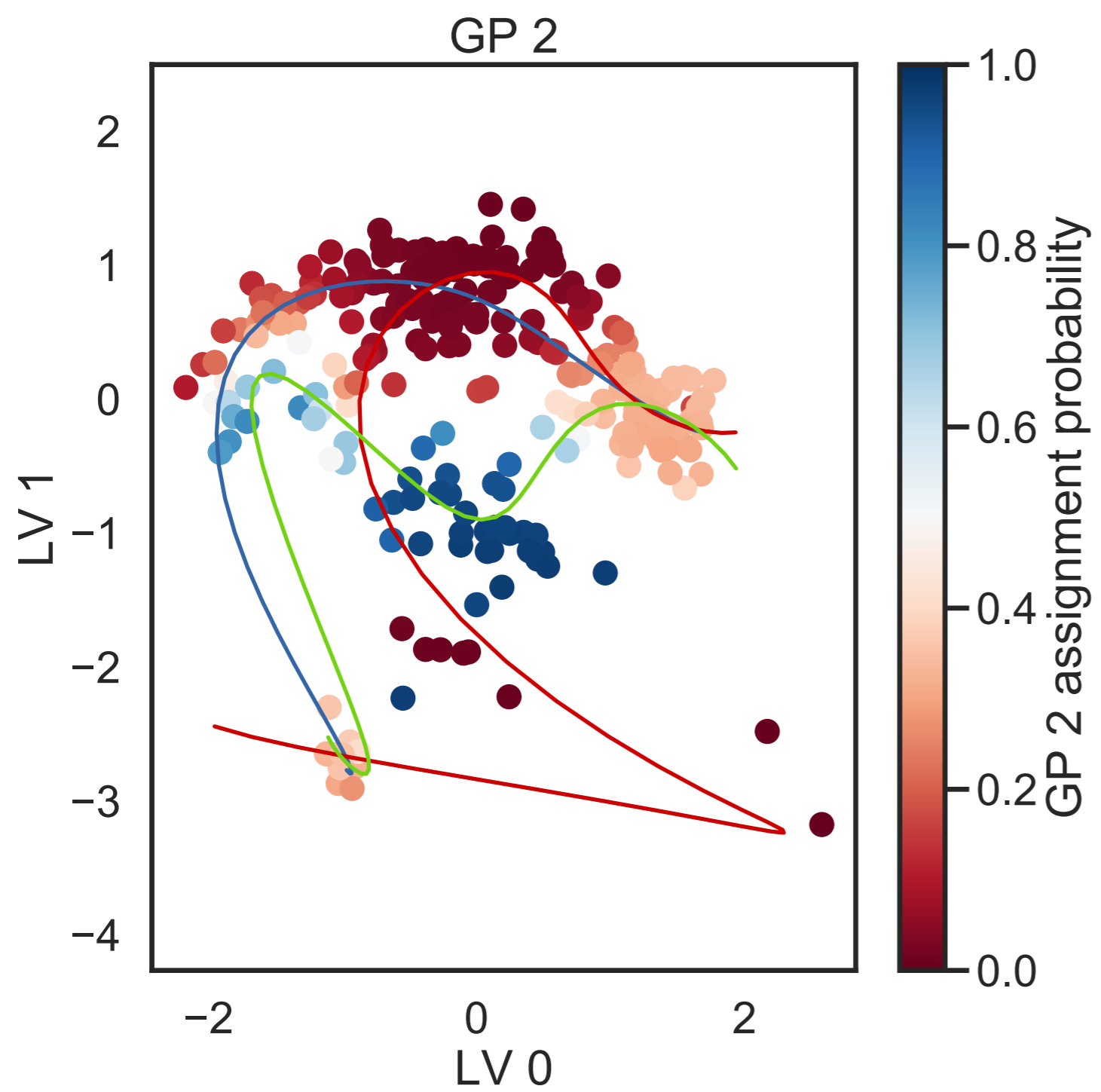

Supplement: Supplementary file 5 — Supplementary Data 2 [file 41467_2019_9670_MOESM5_ESM.zip › Sup_data2/Guo_2013/gpfates/gpfates_guo2013_fig2.pdf]

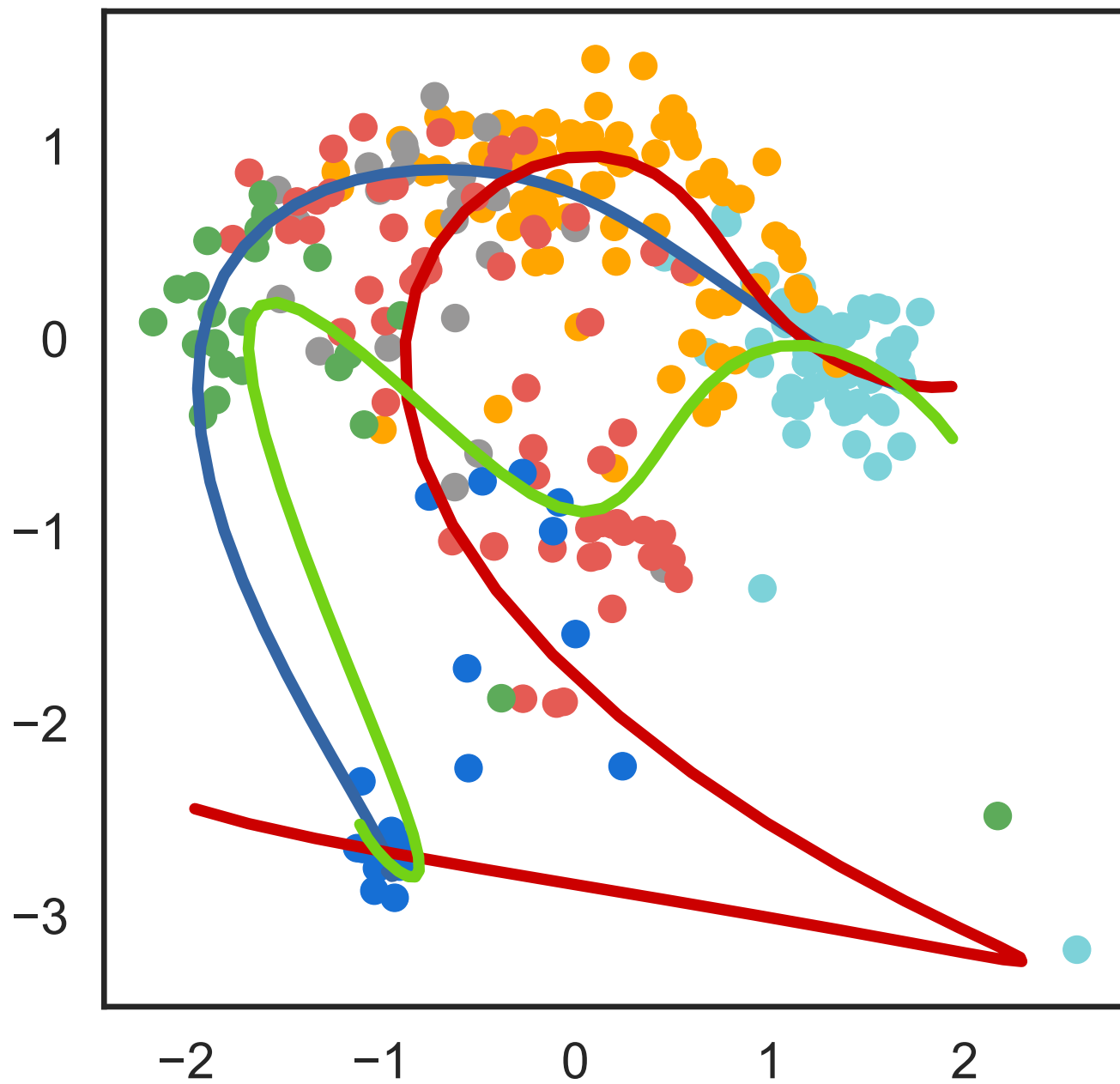

Supplement: Supplementary file 5 — Supplementary Data 2 [file 41467_2019_9670_MOESM5_ESM.zip › Sup_data2/Guo_2013/gpfates/gpfates_guo2013.pdf]

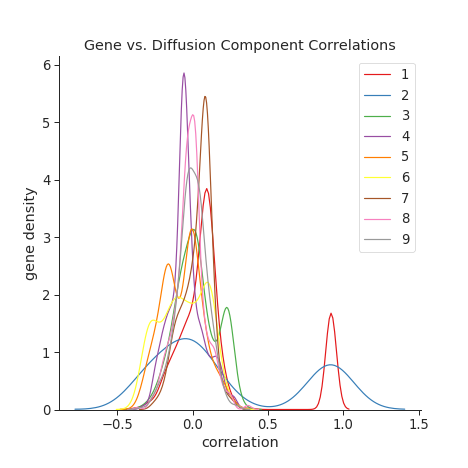

Supplement: Supplementary file 5 — Supplementary Data 2 [file 41467_2019_9670_MOESM5_ESM.zip › Sup_data2/Synthetic/wishbone/gene_component_correlations.png]

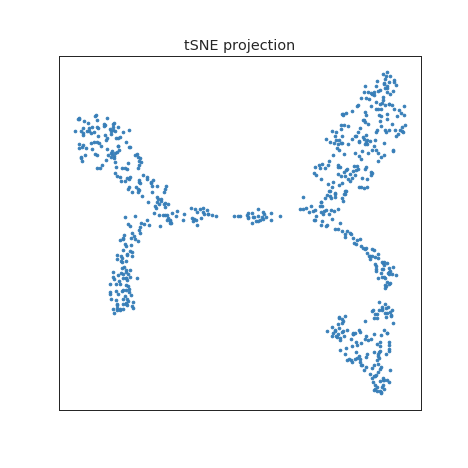

Supplement: Supplementary file 5 — Supplementary Data 2 [file 41467_2019_9670_MOESM5_ESM.zip › Sup_data2/Synthetic/wishbone/tSNE.png]

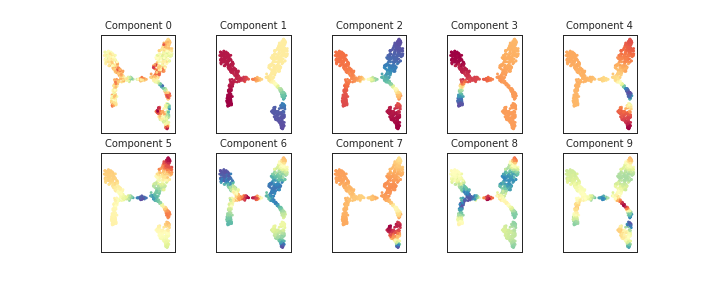

Supplement: Supplementary file 5 — Supplementary Data 2 [file 41467_2019_9670_MOESM5_ESM.zip › Sup_data2/Synthetic/wishbone/diffusion_components.png]

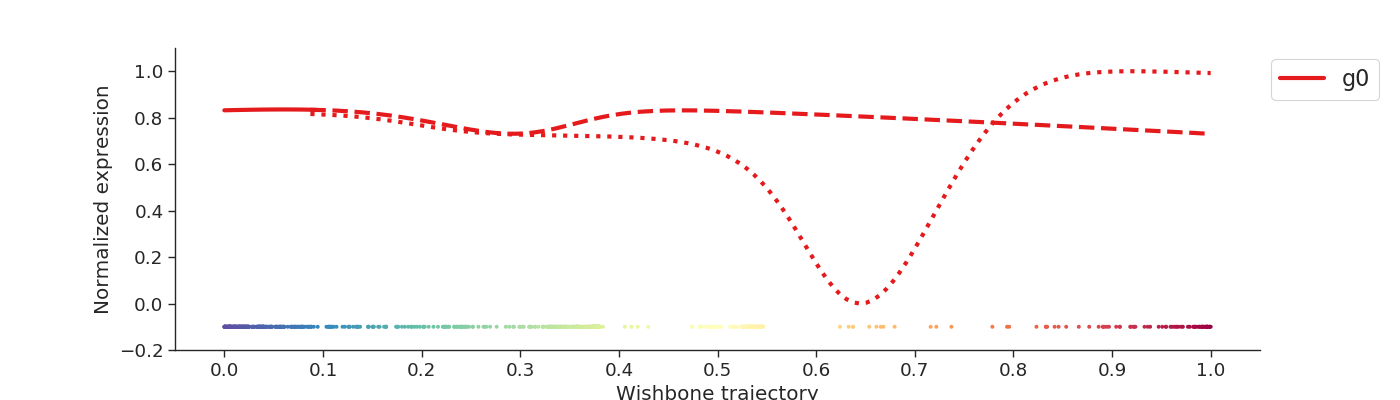

Supplement: Supplementary file 5 — Supplementary Data 2 [file 41467_2019_9670_MOESM5_ESM.zip › Sup_data2/Synthetic/wishbone/marker_trajectory_g0.png]

Wishbone trajectory

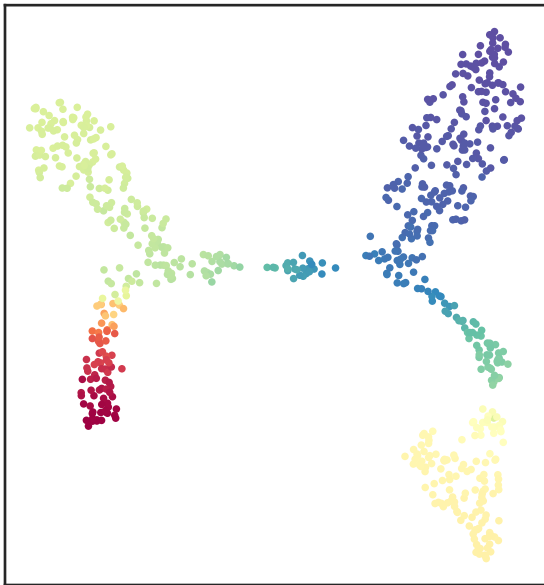

Branch associations

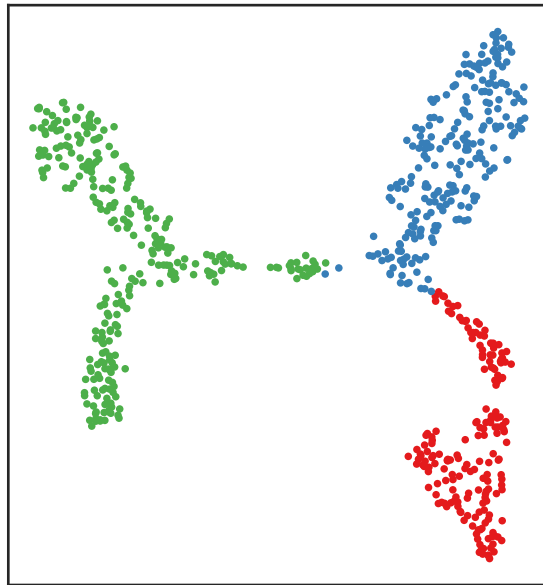

Supplement: Supplementary file 5 — Supplementary Data 2 [file 41467_2019_9670_MOESM5_ESM.zip › Sup_data2/Synthetic/wishbone/wishbone_on_tsne.pdf]

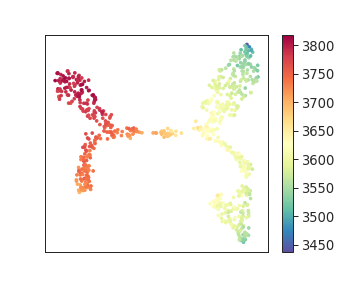

Supplement: Supplementary file 5 — Supplementary Data 2 [file 41467_2019_9670_MOESM5_ESM.zip › Sup_data2/Synthetic/wishbone/tSNE_by_cell_sizes.png]

tSNE projection

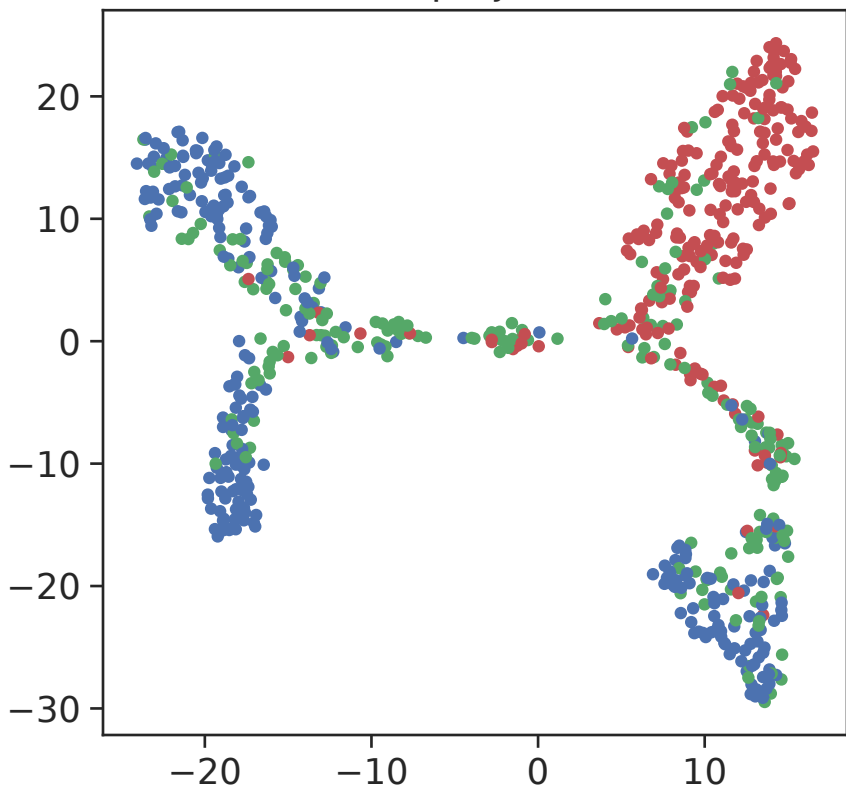

Supplement: Supplementary file 5 — Supplementary Data 2 [file 41467_2019_9670_MOESM5_ESM.zip › Sup_data2/Synthetic/wishbone/tSNE_by_labels.pdf]

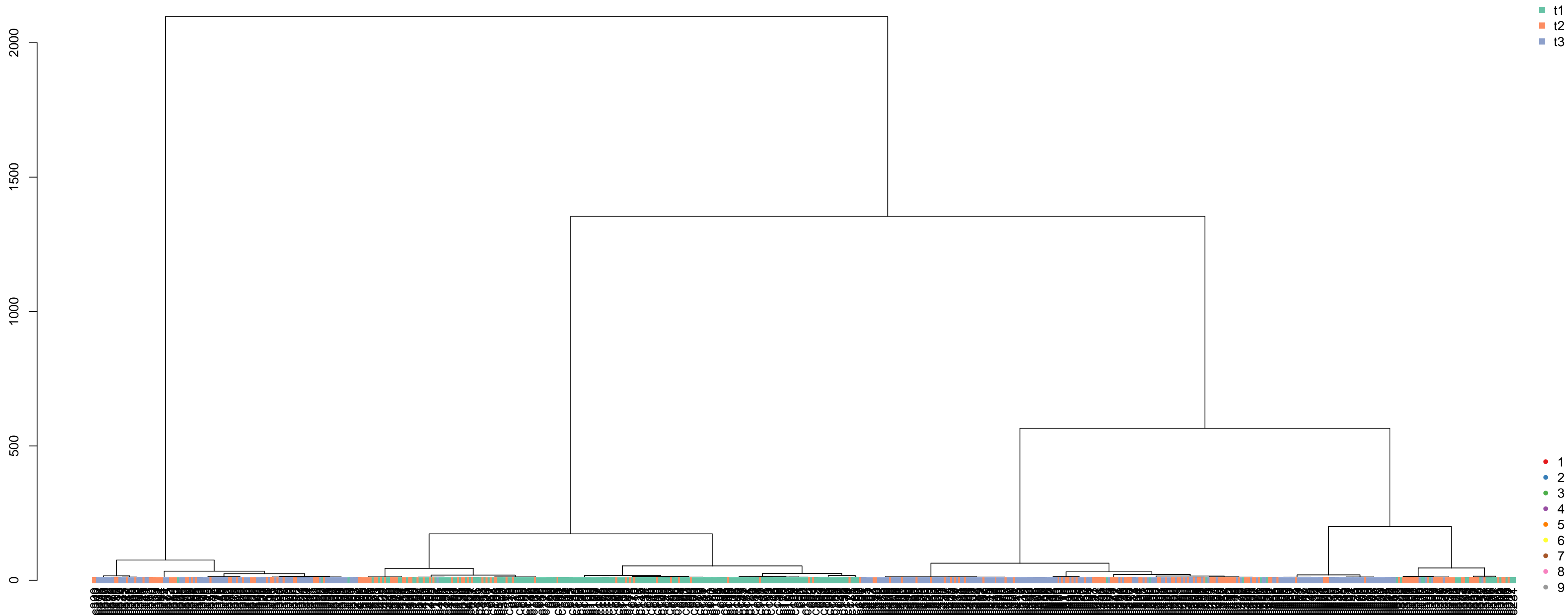

Supplement: Supplementary file 5 — Supplementary Data 2 [file 41467_2019_9670_MOESM5_ESM.zip › Sup_data2/Synthetic/mpath/simulation_rpkm_hc.pdf]

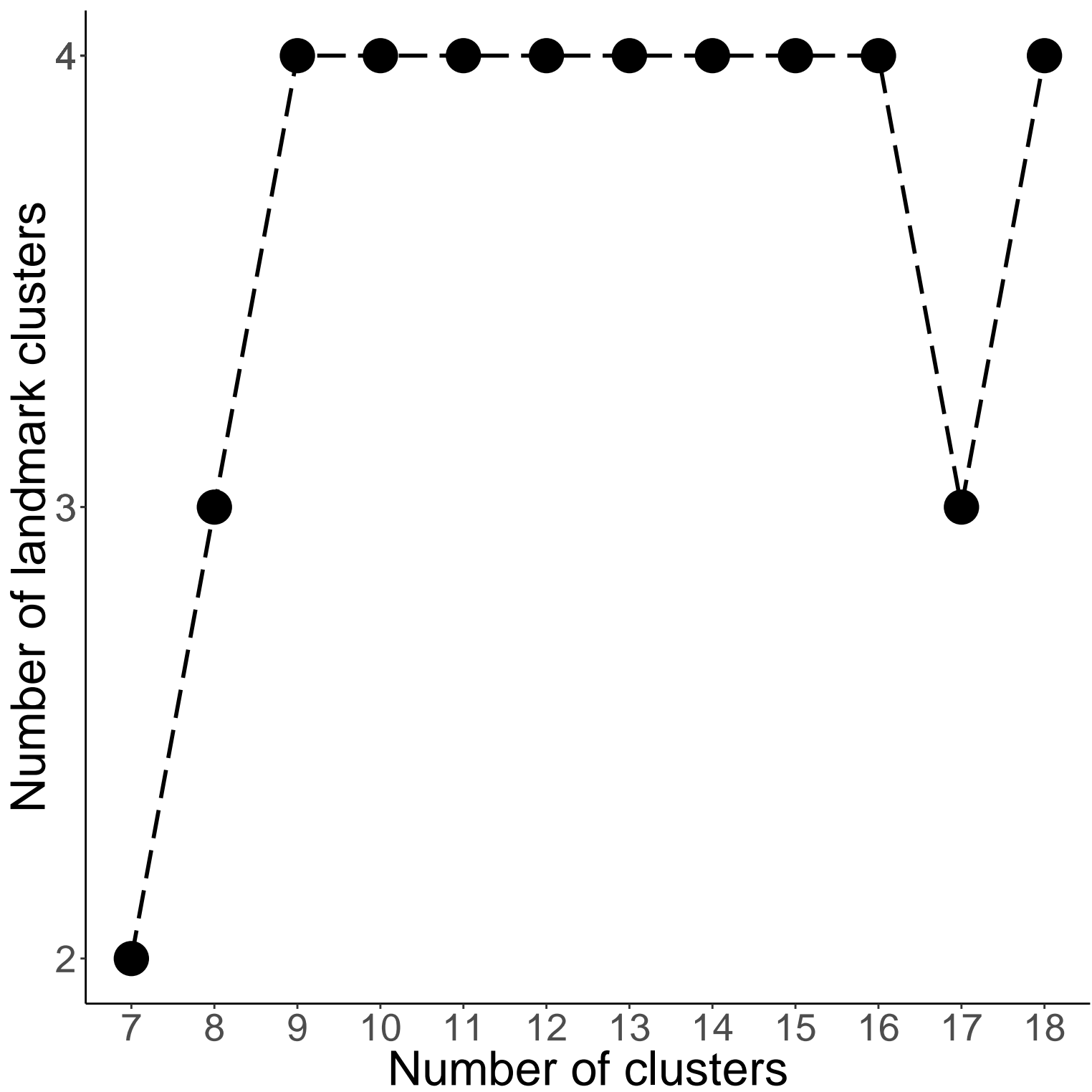

Supplement: Supplementary file 5 — Supplementary Data 2 [file 41467_2019_9670_MOESM5_ESM.zip › Sup_data2/Synthetic/mpath/simulation_rpkm_ncluster_vs_nlm.pdf]

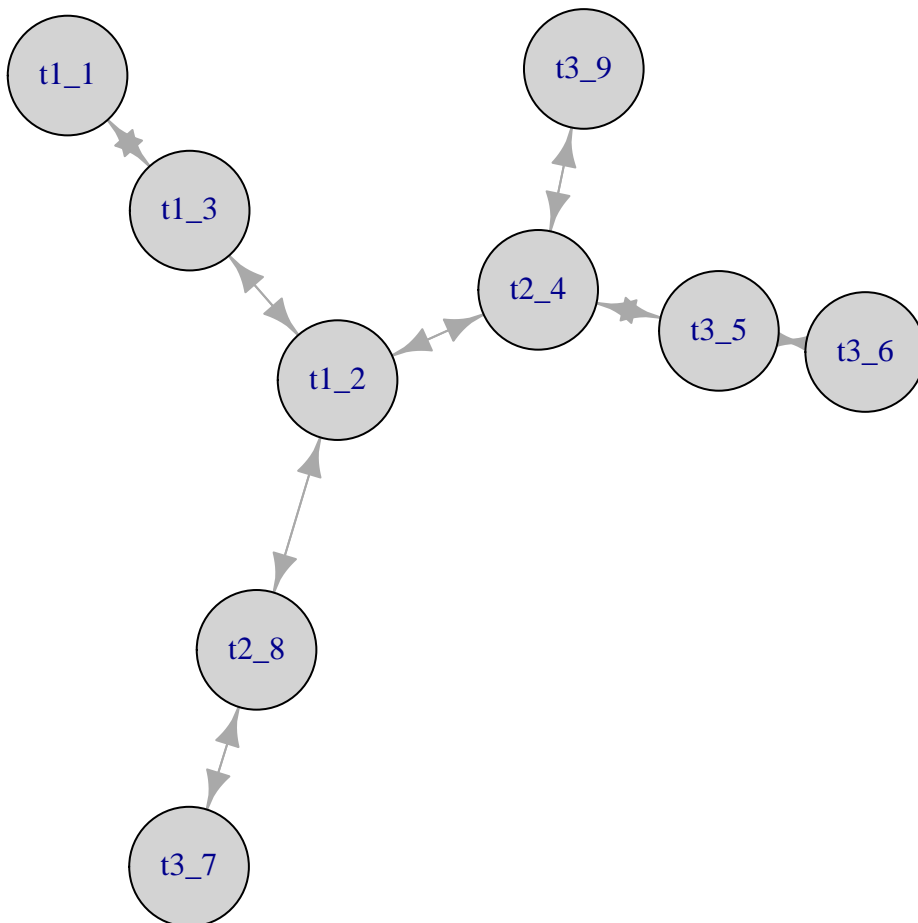

Supplement: Supplementary file 5 — Supplementary Data 2 [file 41467_2019_9670_MOESM5_ESM.zip › Sup_data2/Synthetic/mpath/simulation_rpkm_state_transition_mst.pdf]

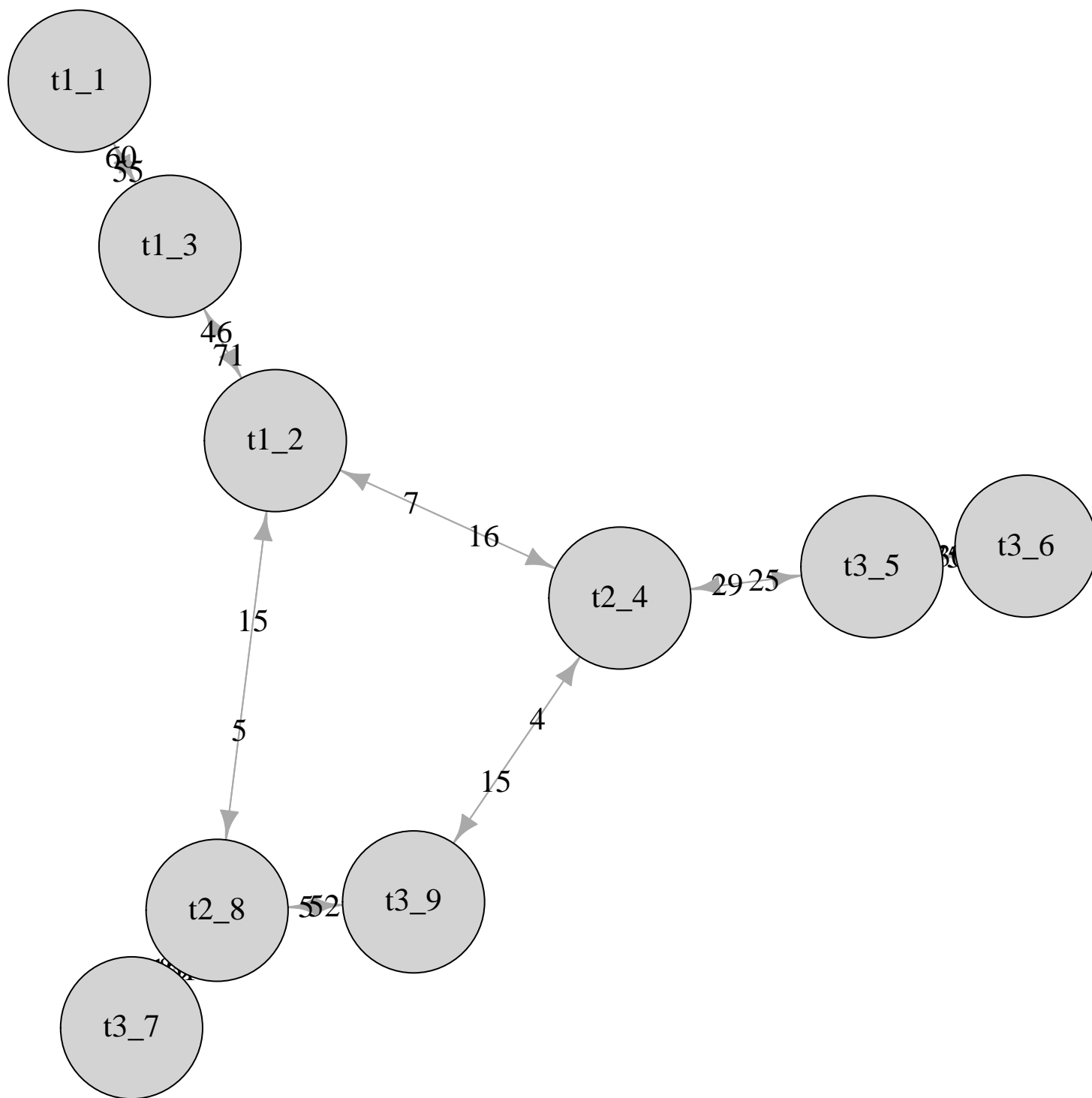

Supplement: Supplementary file 5 — Supplementary Data 2 [file 41467_2019_9670_MOESM5_ESM.zip › Sup_data2/Synthetic/mpath/simulation_rpkm_state_transition.pdf]

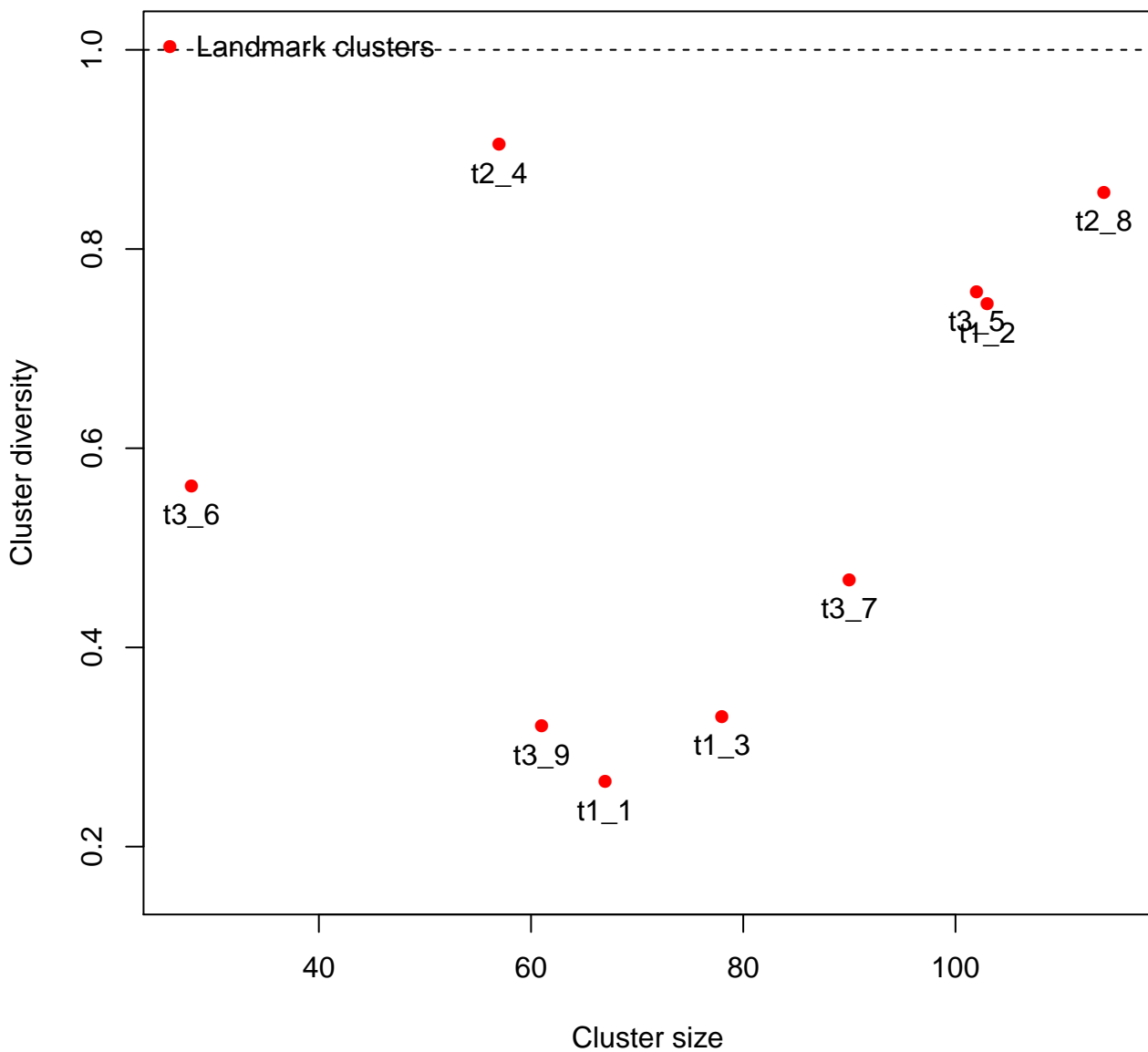

Supplement: Supplementary file 5 — Supplementary Data 2 [file 41467_2019_9670_MOESM5_ESM.zip › Sup_data2/Synthetic/mpath/simulation_rpkm_landmark_cluster.pdf]

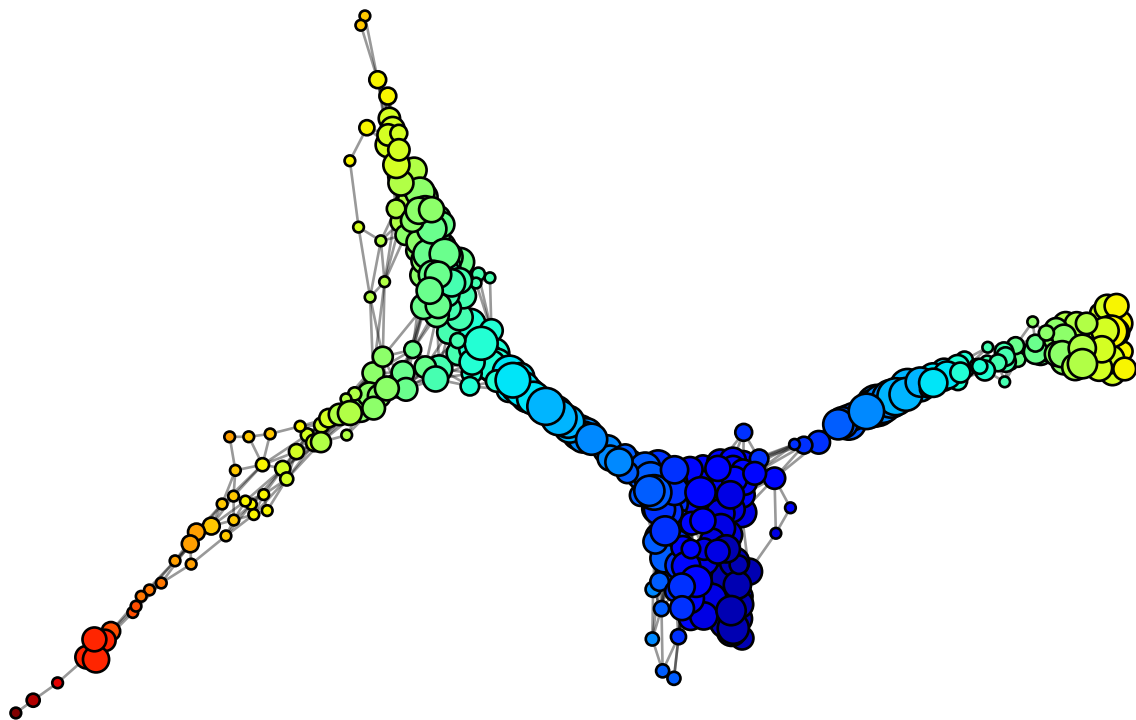

Supplement: Supplementary file 5 — Supplementary Data 2 [file 41467_2019_9670_MOESM5_ESM.zip › Sup_data2/Synthetic/sctda/dist_root.pdf]

label    t1    t2    t3

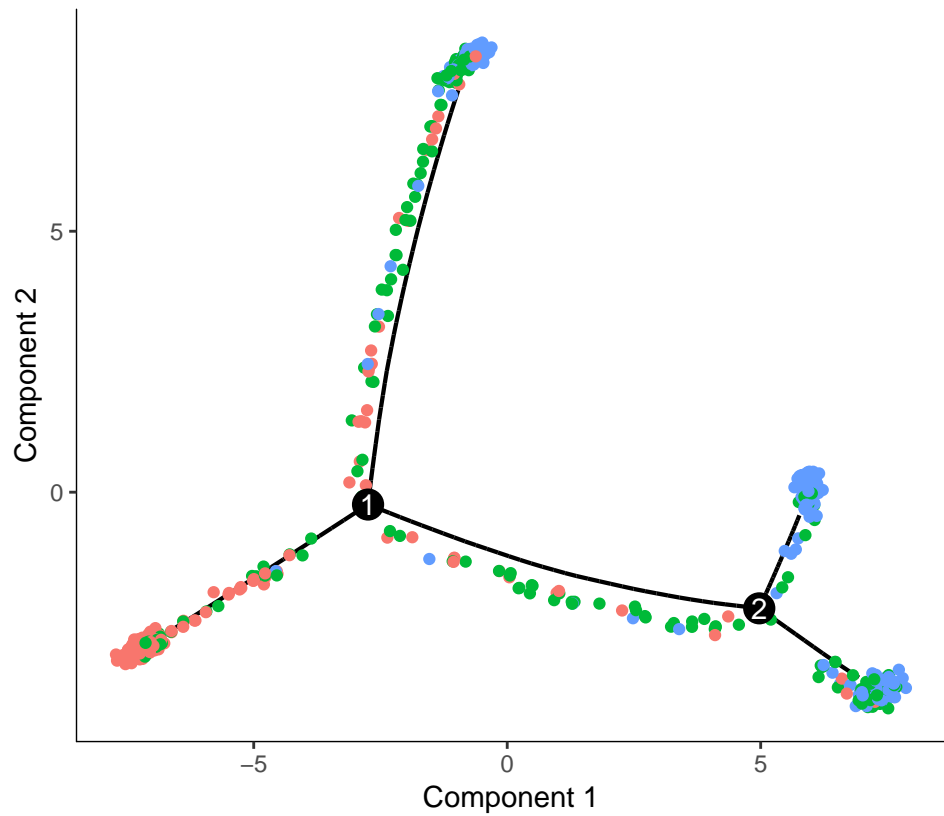

Supplement: Supplementary file 5 — Supplementary Data 2 [file 41467_2019_9670_MOESM5_ESM.zip › Sup_data2/Synthetic/monocle2/monocle2_trajectory_by_label.pdf]

Pseudotime

0 5 10 15

Component 2

Component 1

1

2

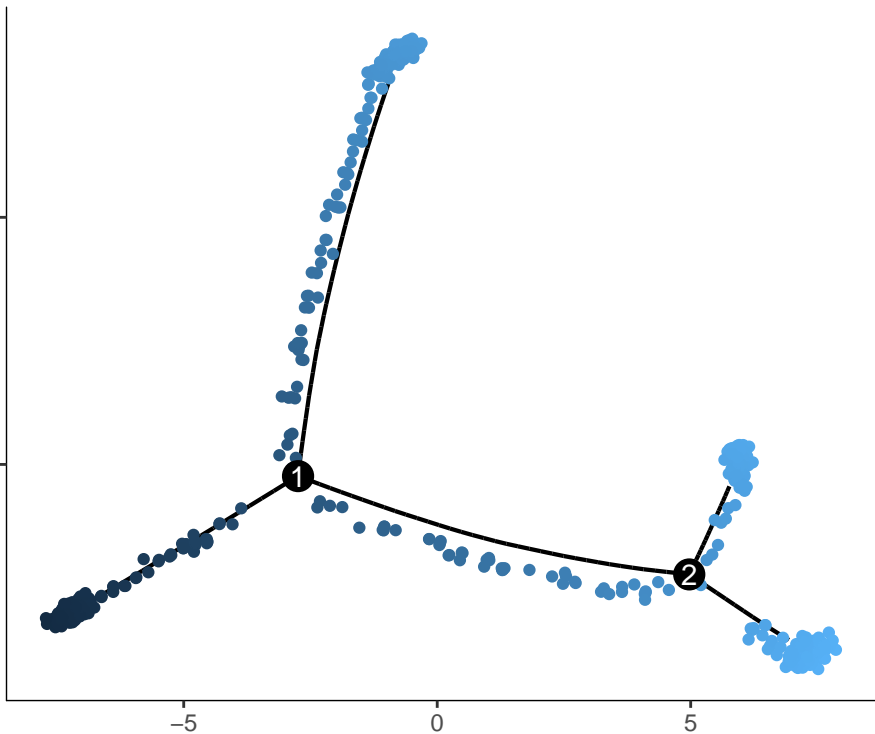

Supplement: Supplementary file 5 — Supplementary Data 2 [file 41467_2019_9670_MOESM5_ESM.zip › Sup_data2/Synthetic/monocle2/monocle2_trajectory_pseudotime.pdf]

State    1    2    3    4    5

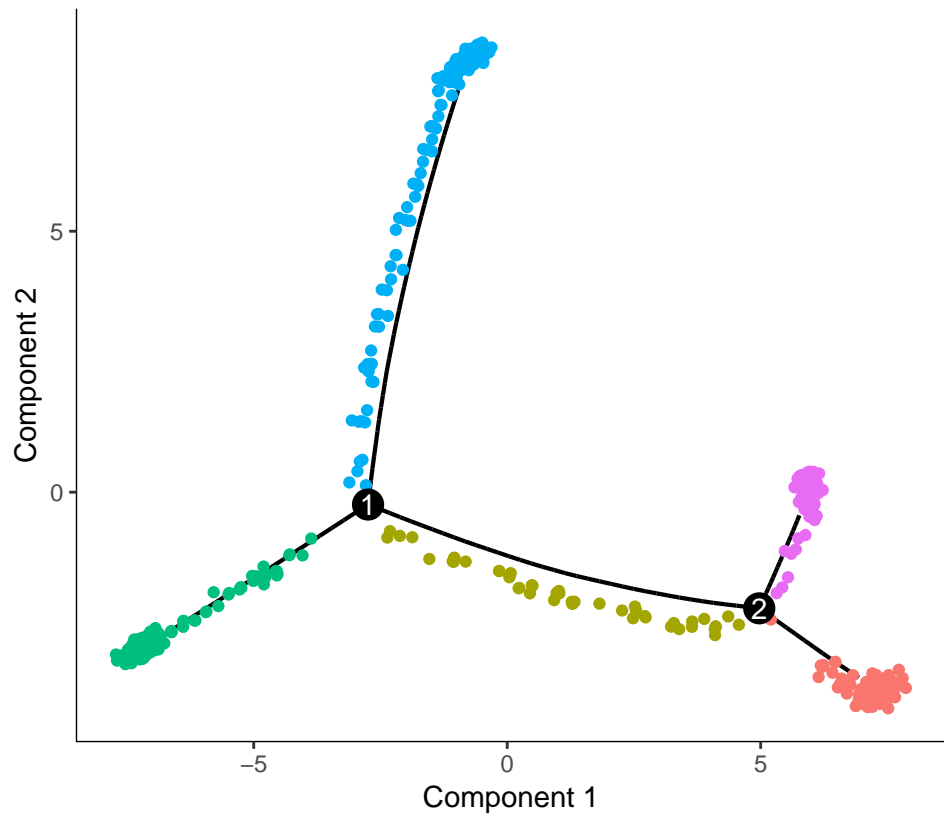

Supplement: Supplementary file 5 — Supplementary Data 2 [file 41467_2019_9670_MOESM5_ESM.zip › Sup_data2/Synthetic/monocle2/monocle2_trajectory_by_state.pdf]

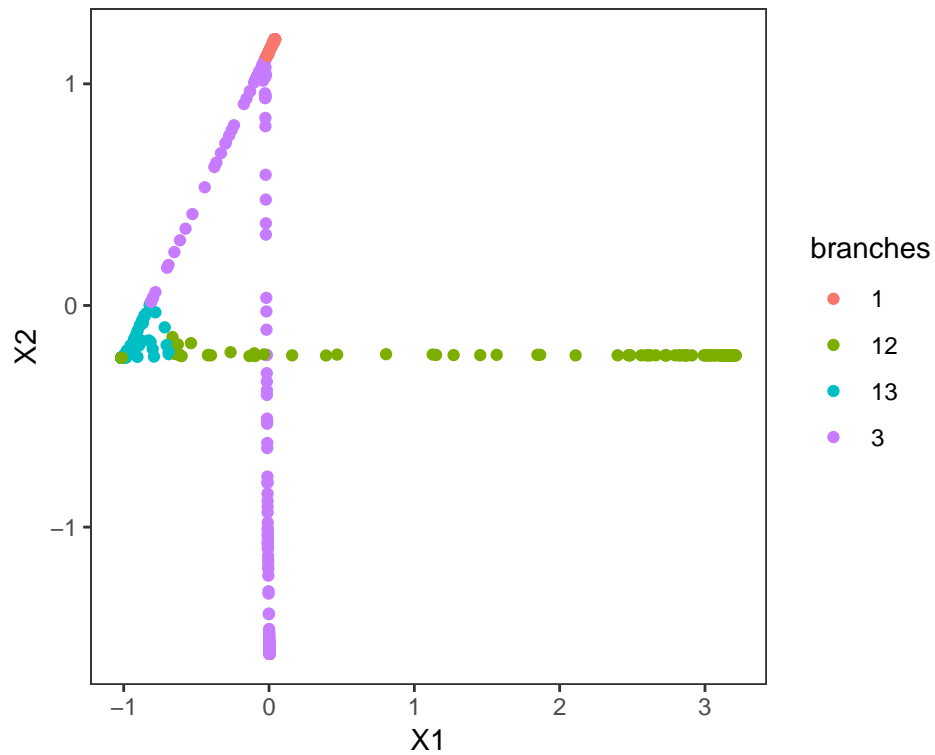

Supplement: Supplementary file 5 — Supplementary Data 2 [file 41467_2019_9670_MOESM5_ESM.zip › Sup_data2/Synthetic/slicer/slicer_trajectory_by_branch.pdf]

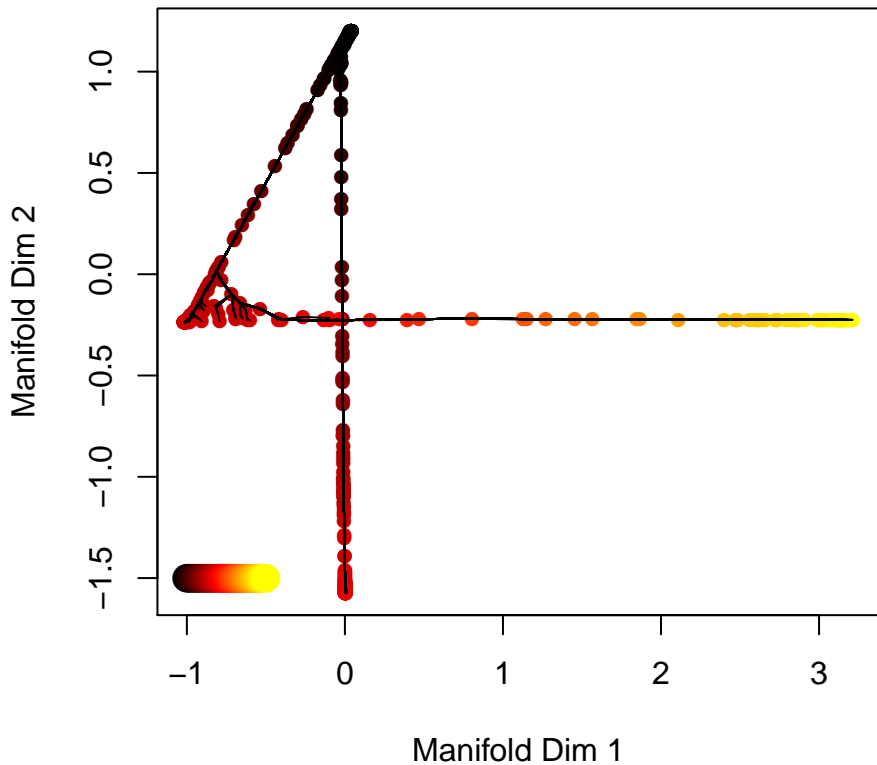

Supplement: Supplementary file 5 — Supplementary Data 2 [file 41467_2019_9670_MOESM5_ESM.zip › Sup_data2/Synthetic/slicer/slicer_trajectory.pdf]

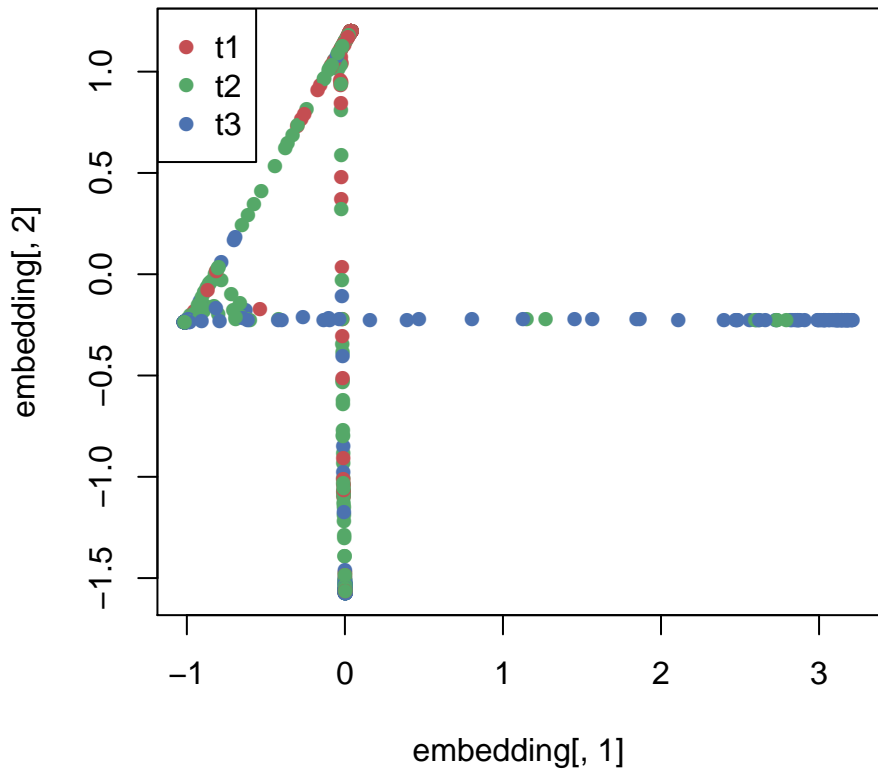

Supplement: Supplementary file 5 — Supplementary Data 2 [file 41467_2019_9670_MOESM5_ESM.zip › Sup_data2/Synthetic/slicer/slicer_trajectory_by_label.pdf]

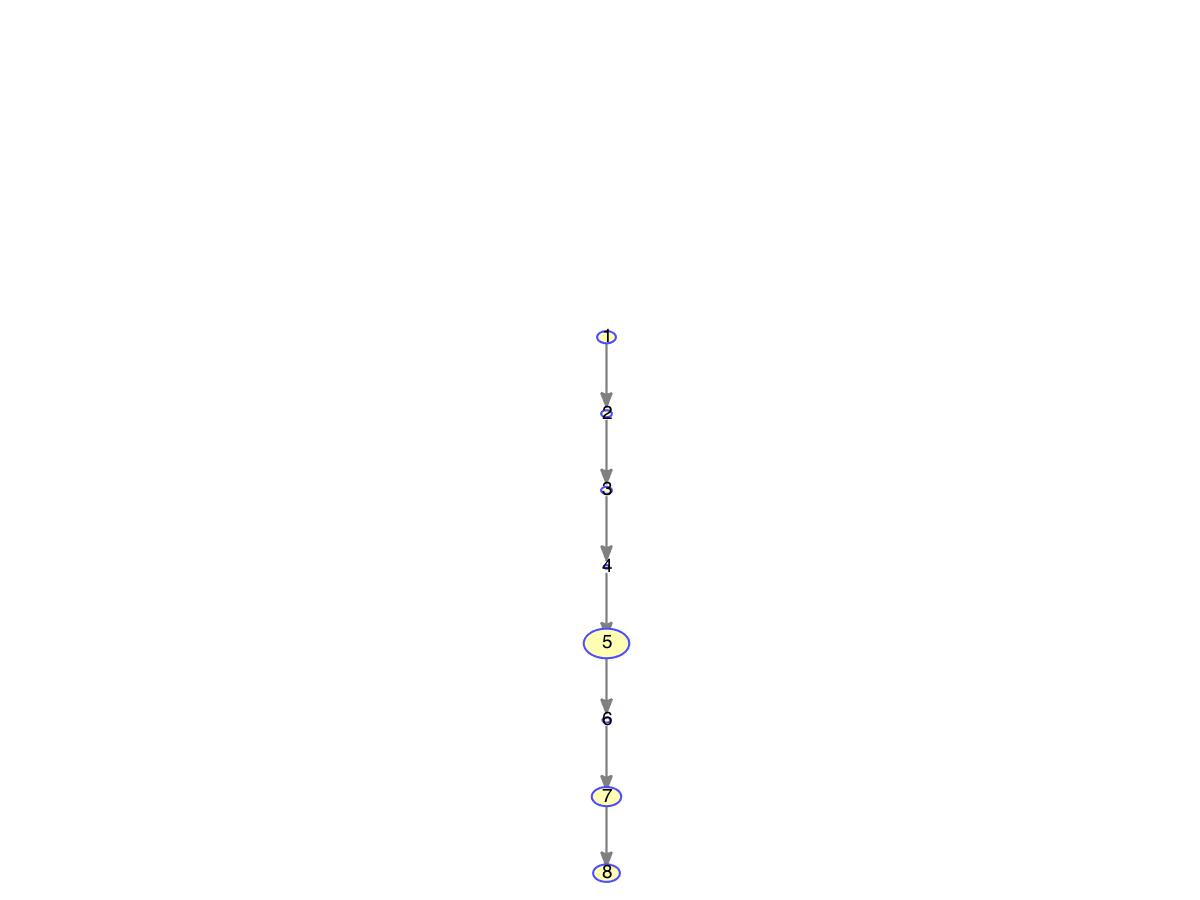

Supplement: Supplementary file 5 — Supplementary Data 2 [file 41467_2019_9670_MOESM5_ESM.zip › Sup_data2/Synthetic/scuba/Result_run1/figures/tree.jpg]

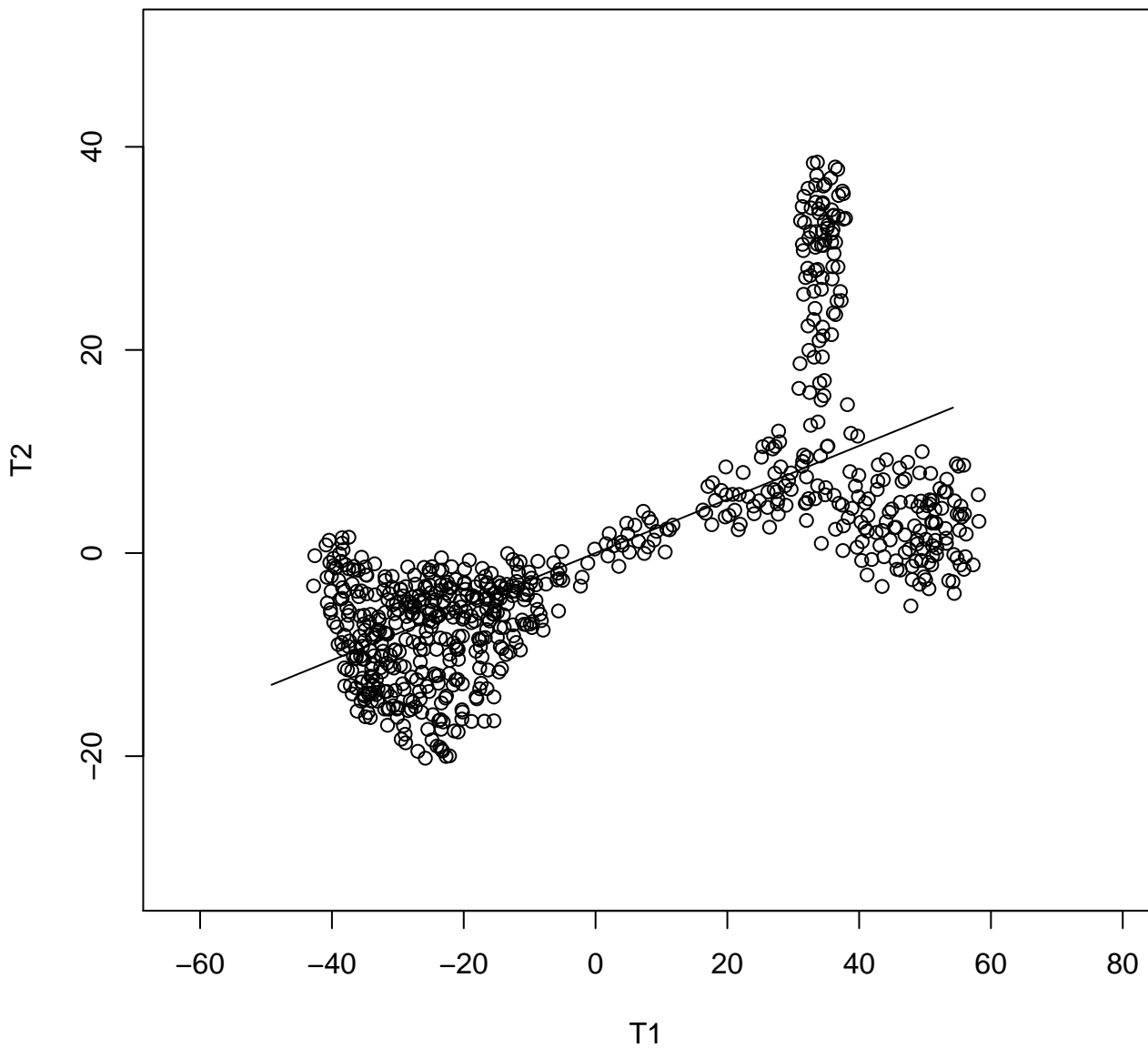

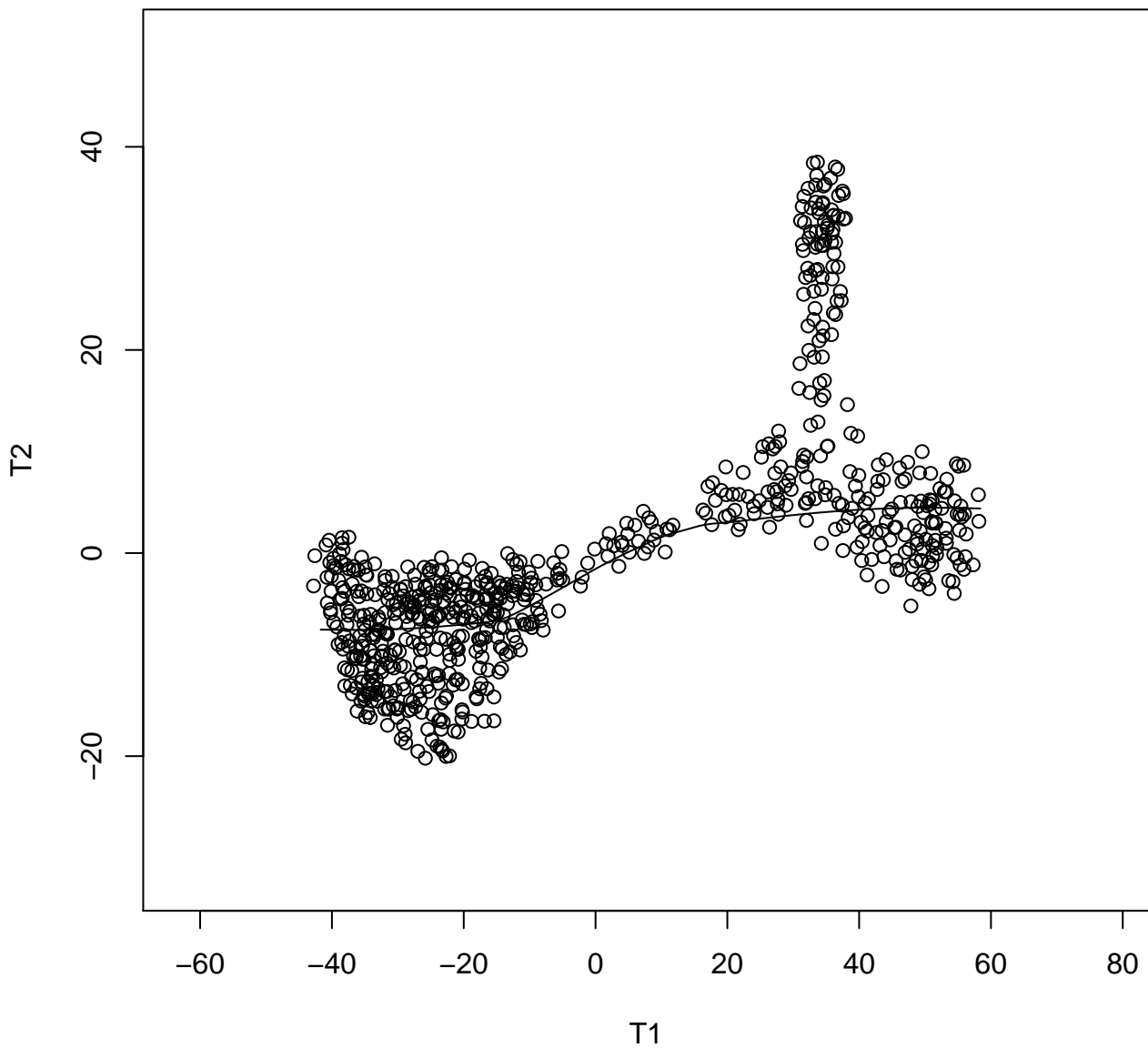

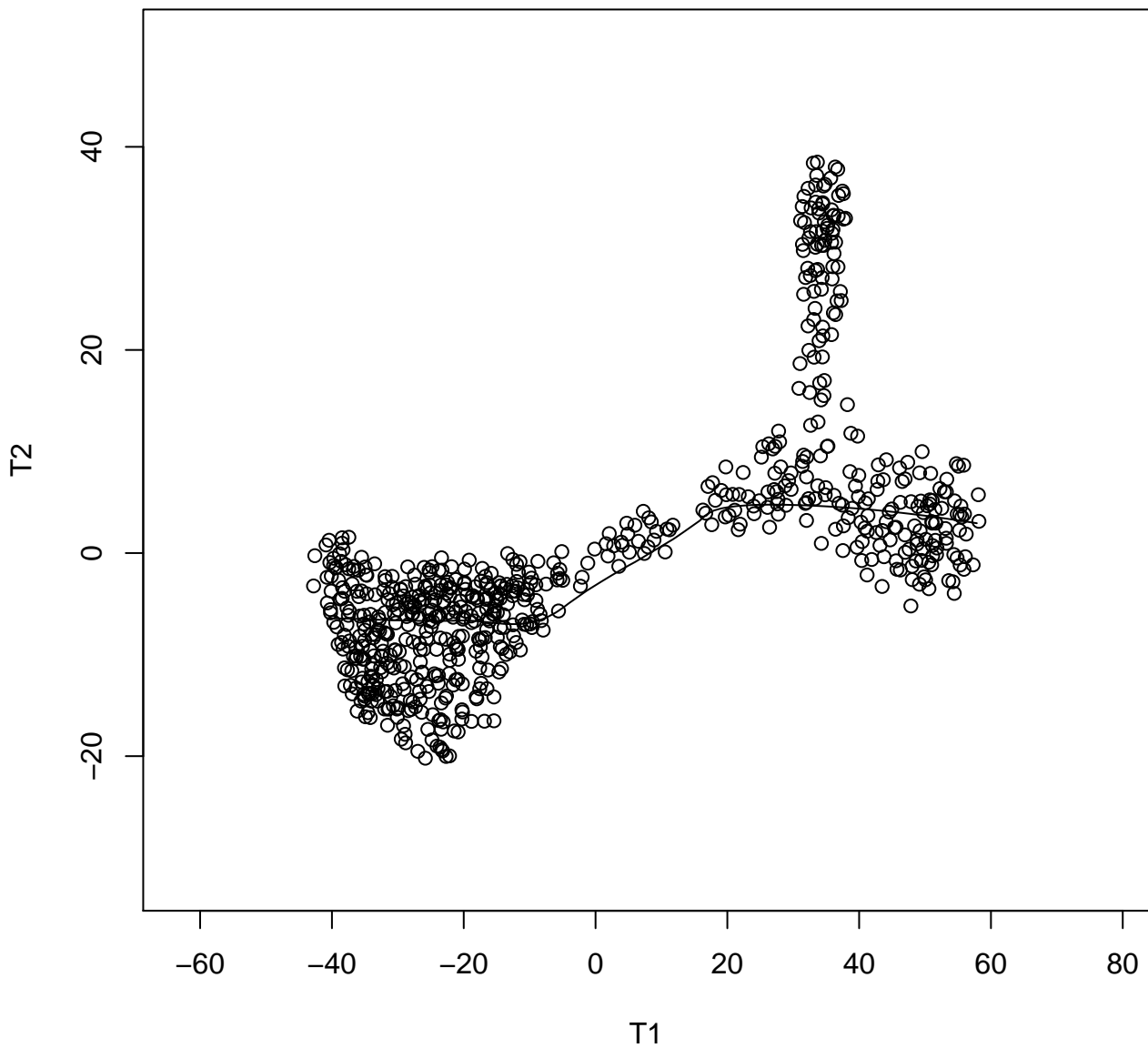

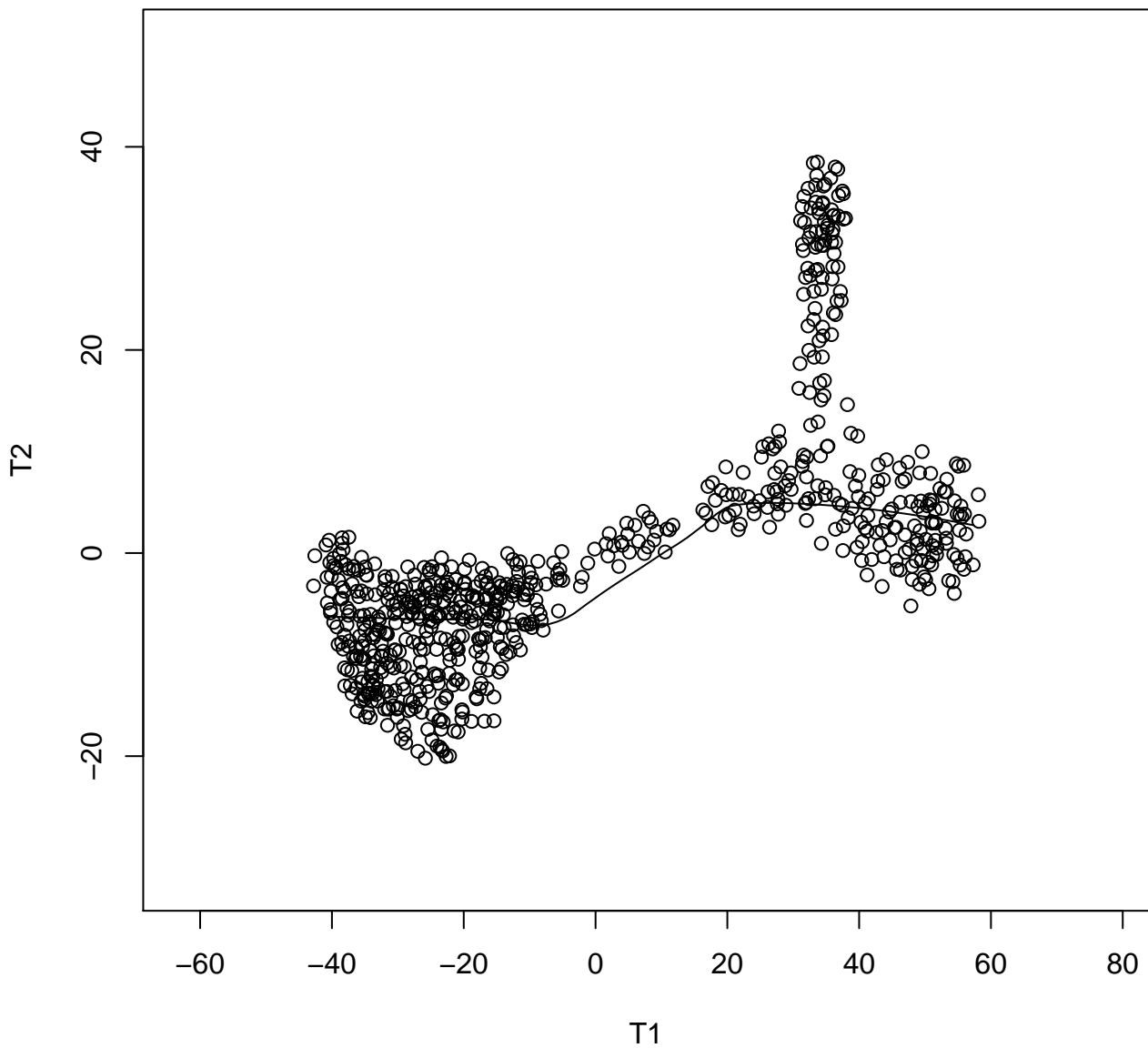

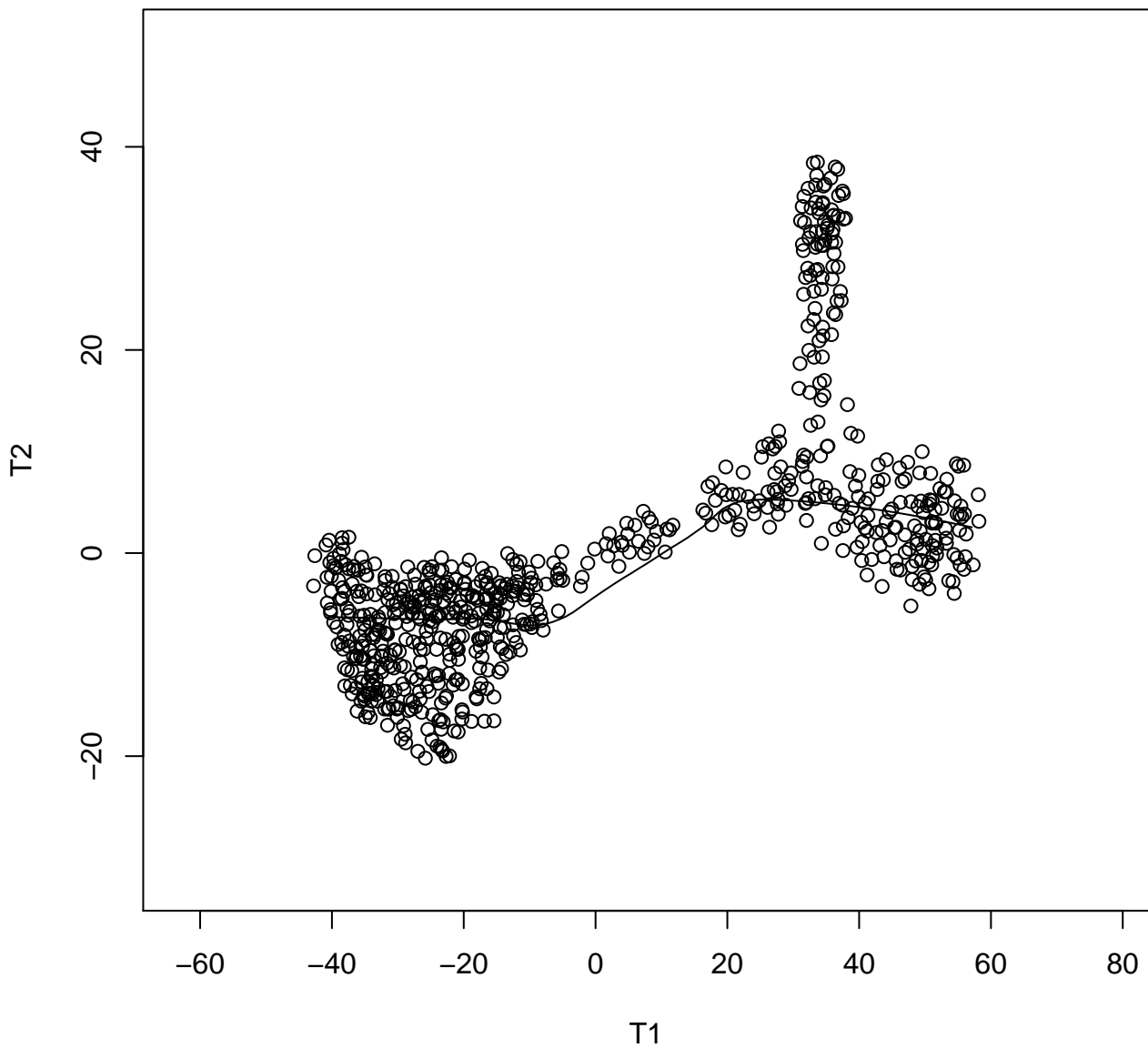

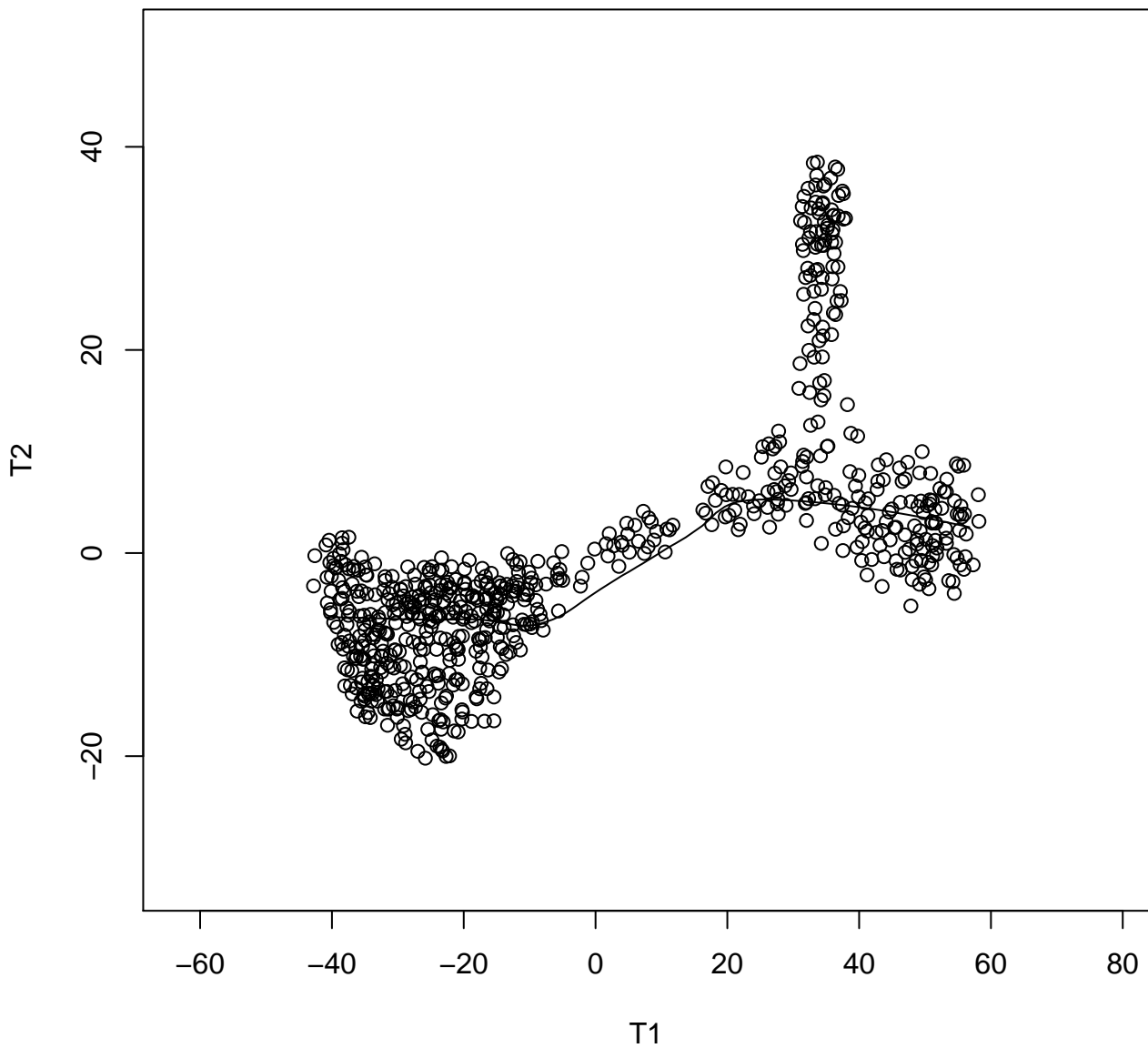

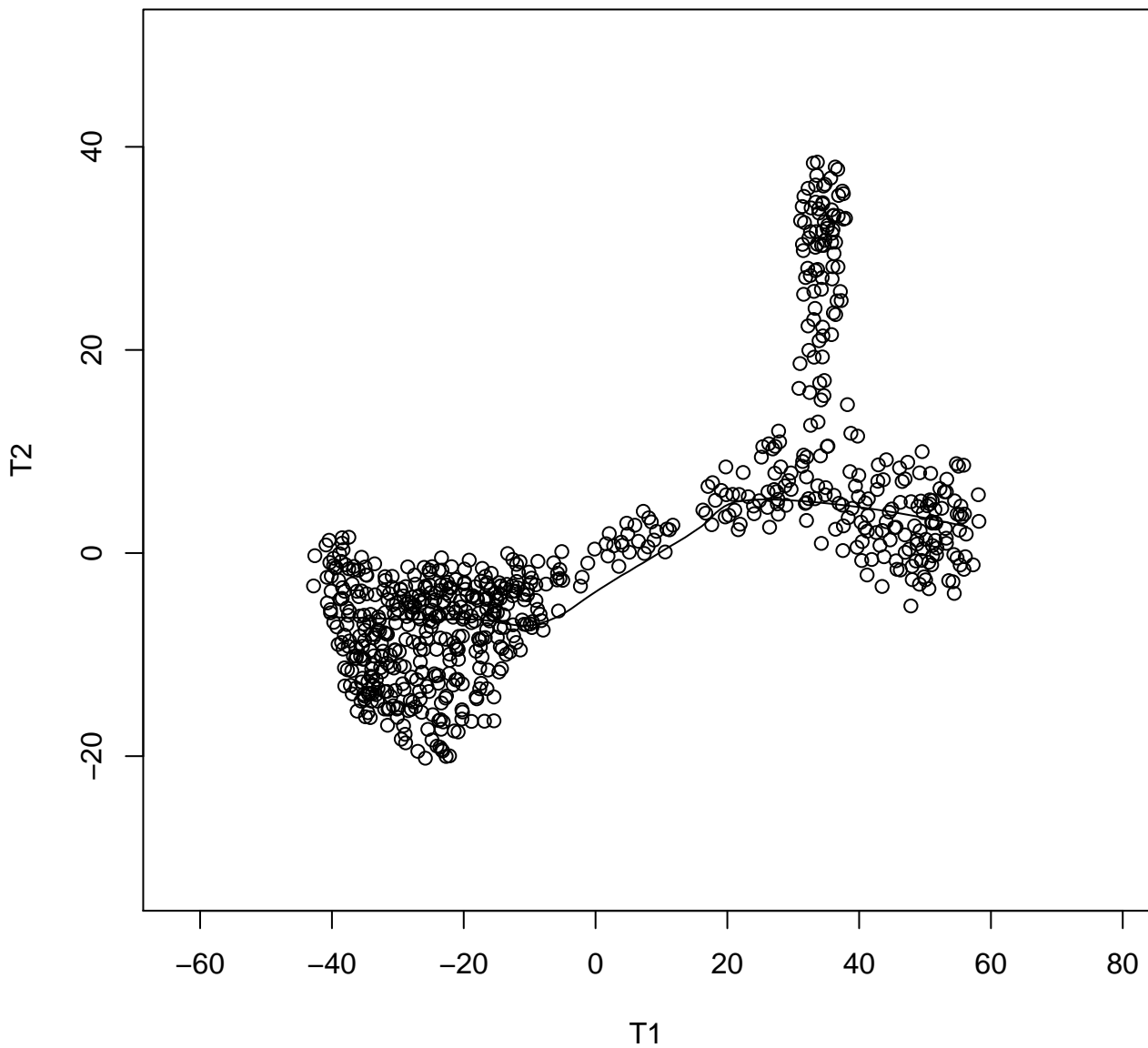

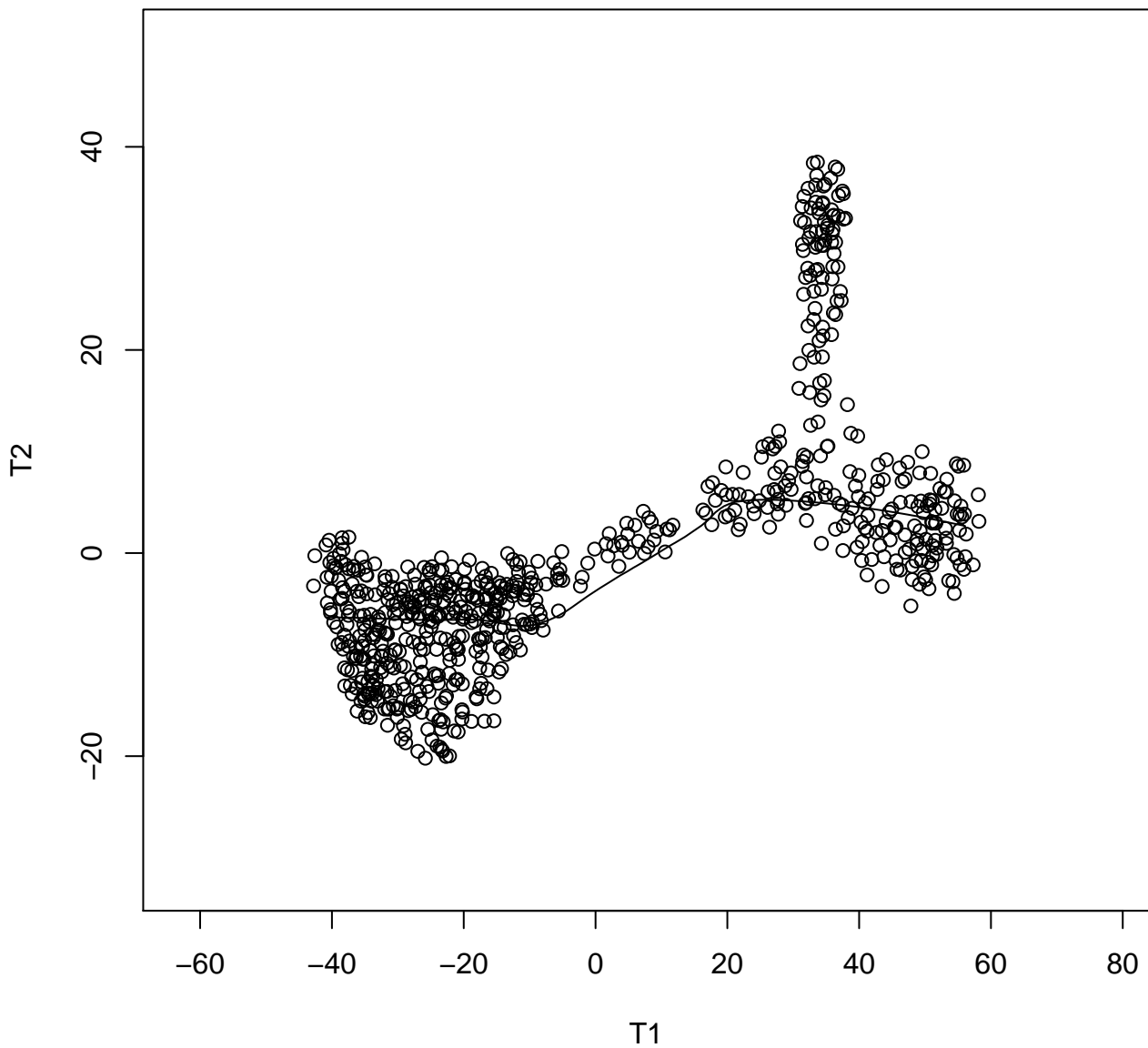

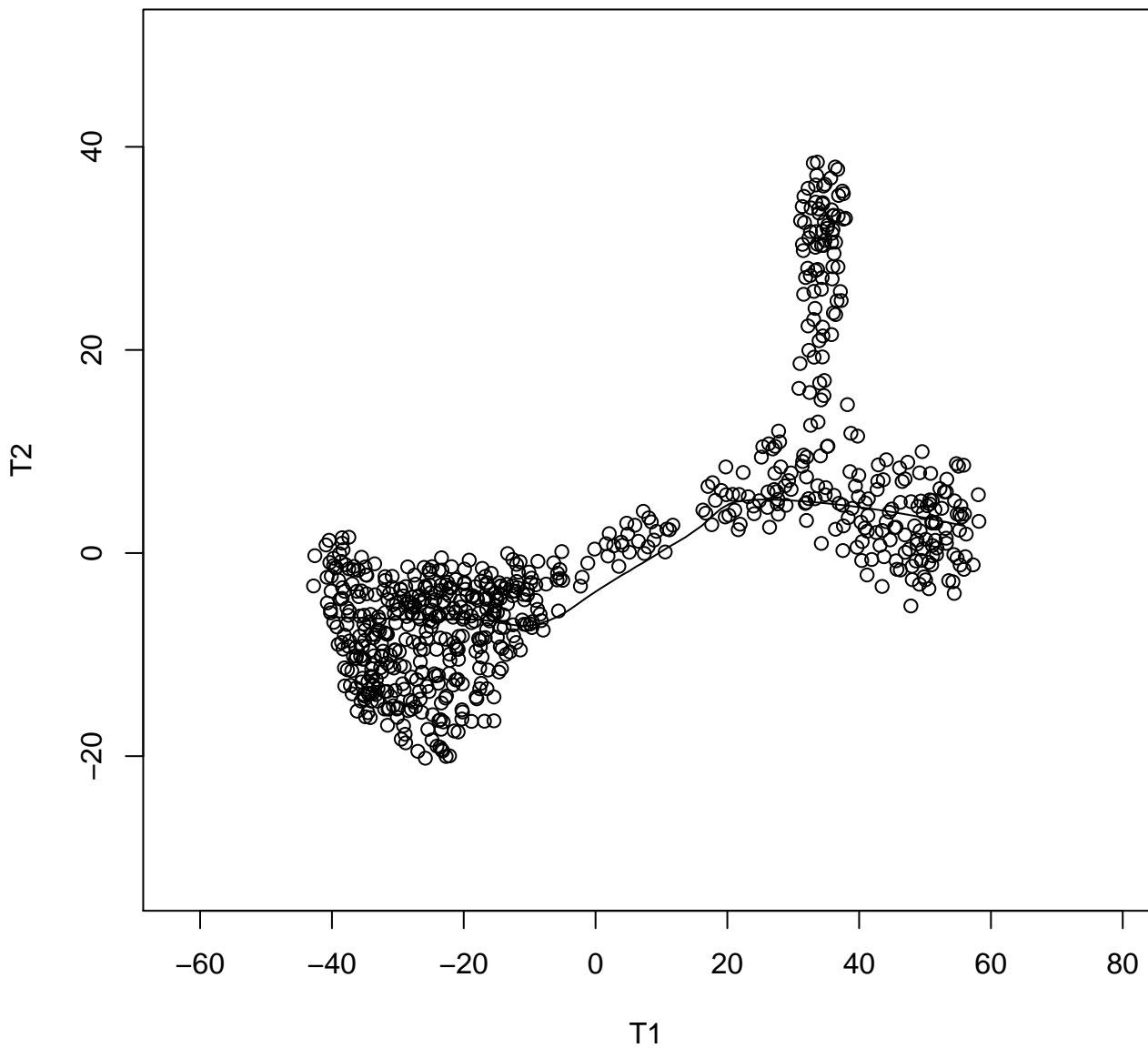

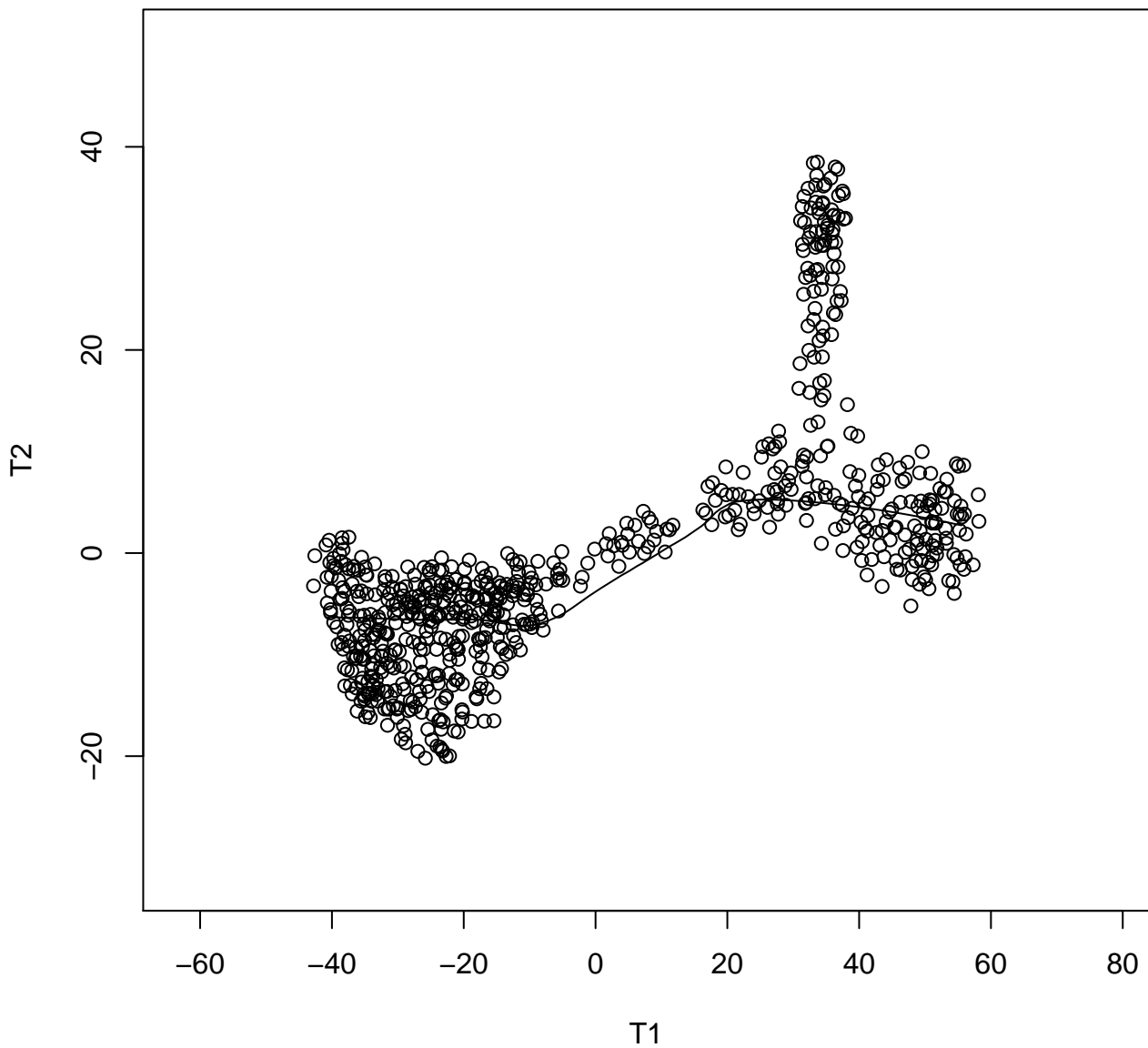

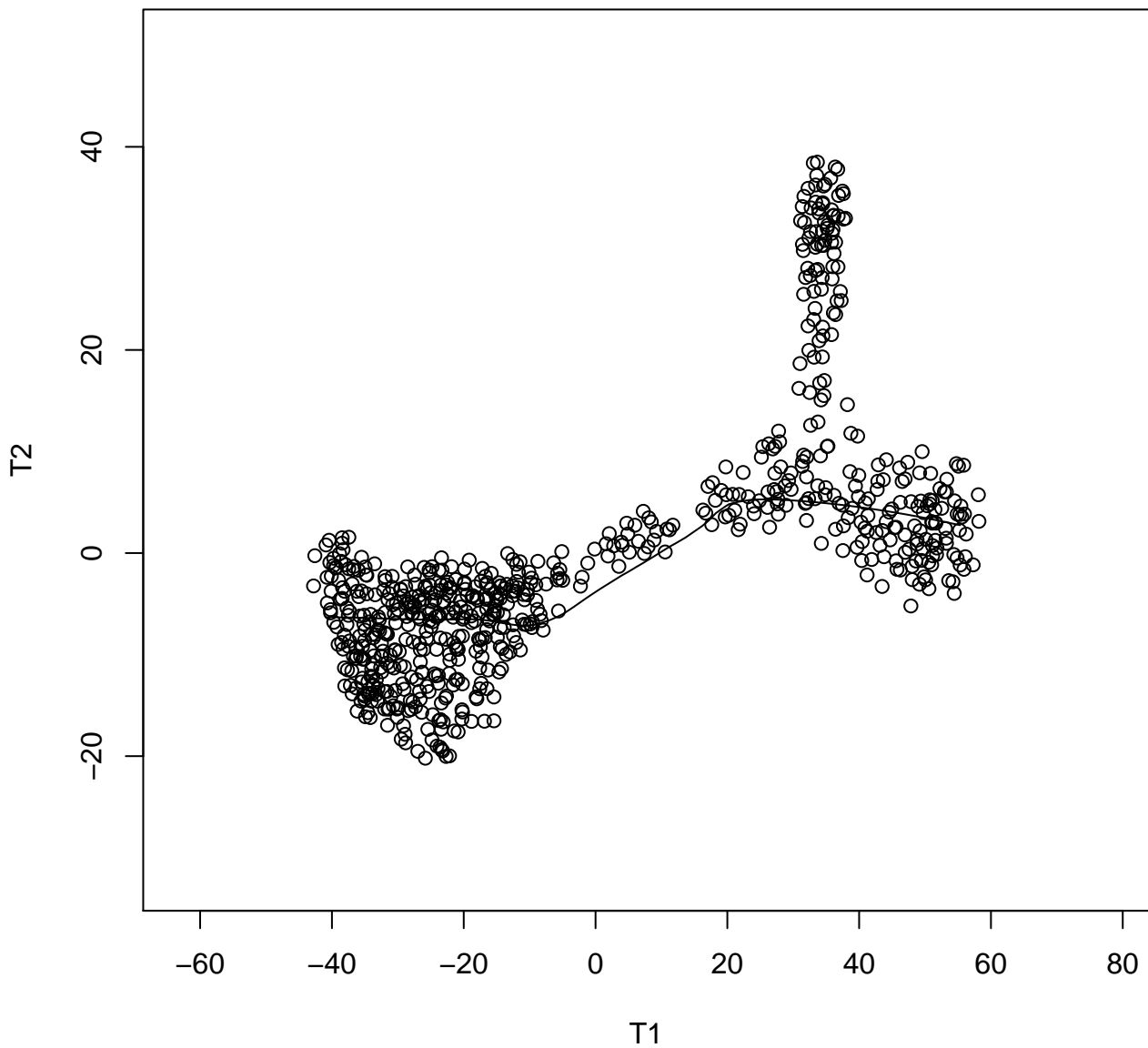

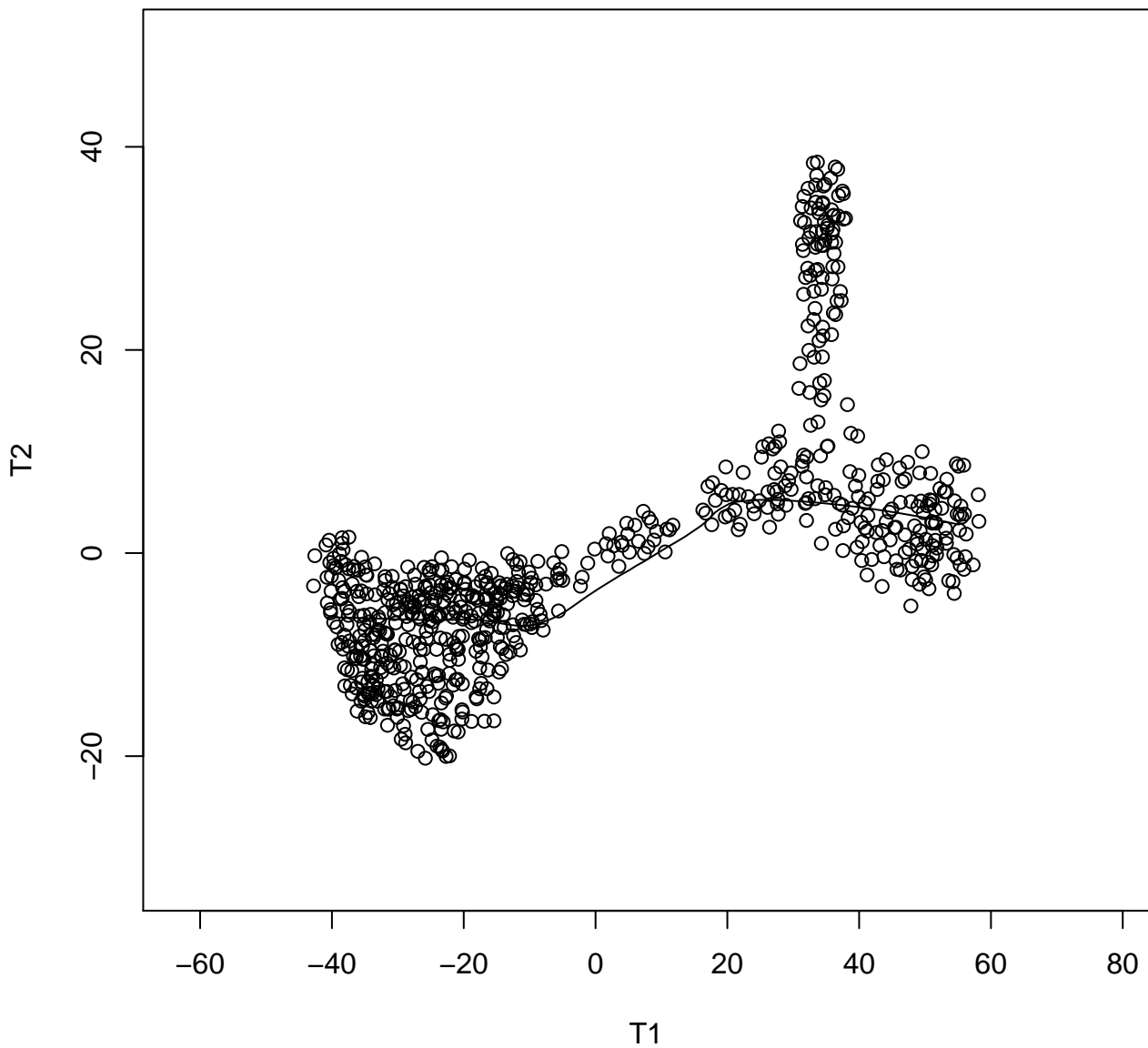

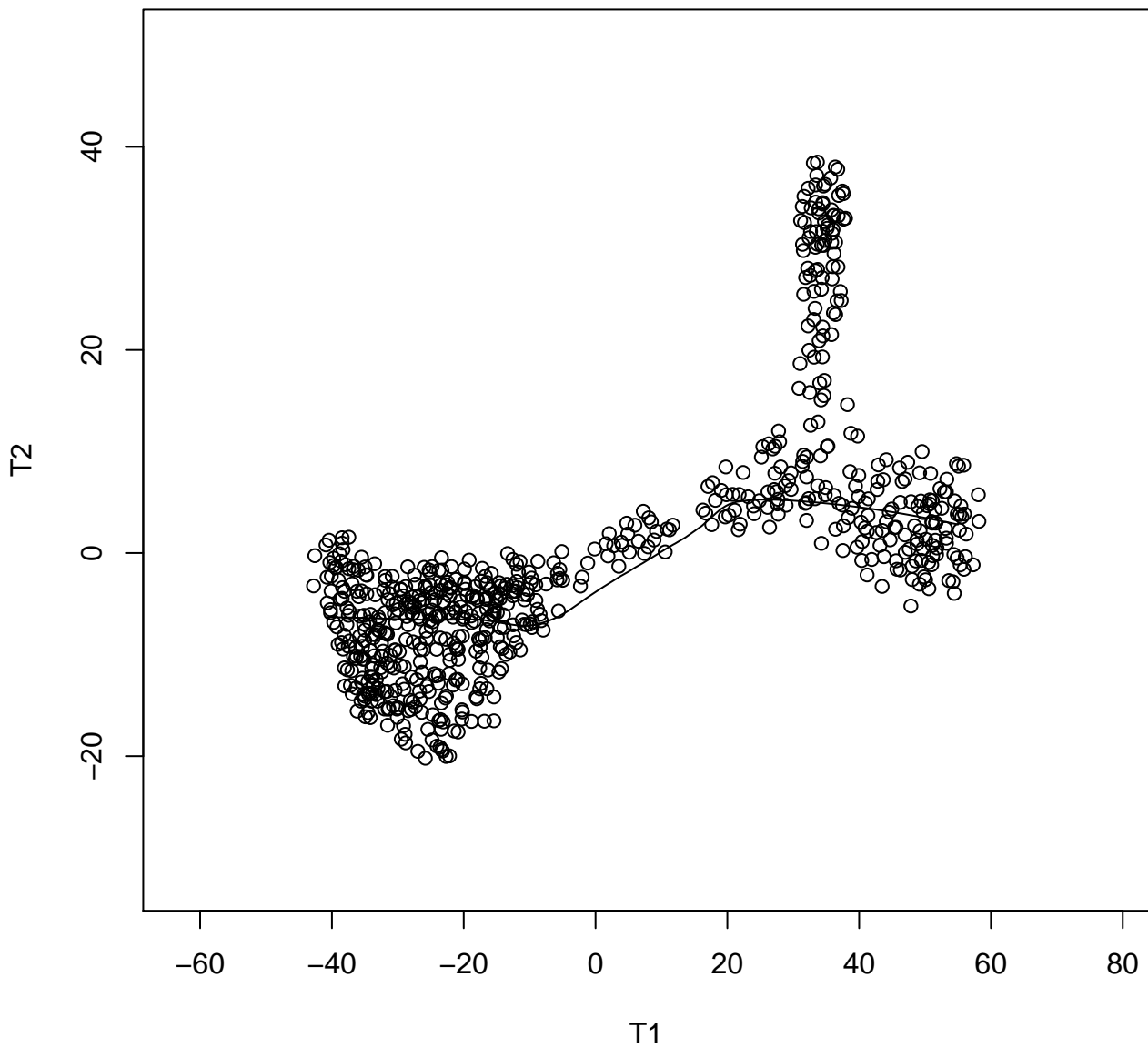

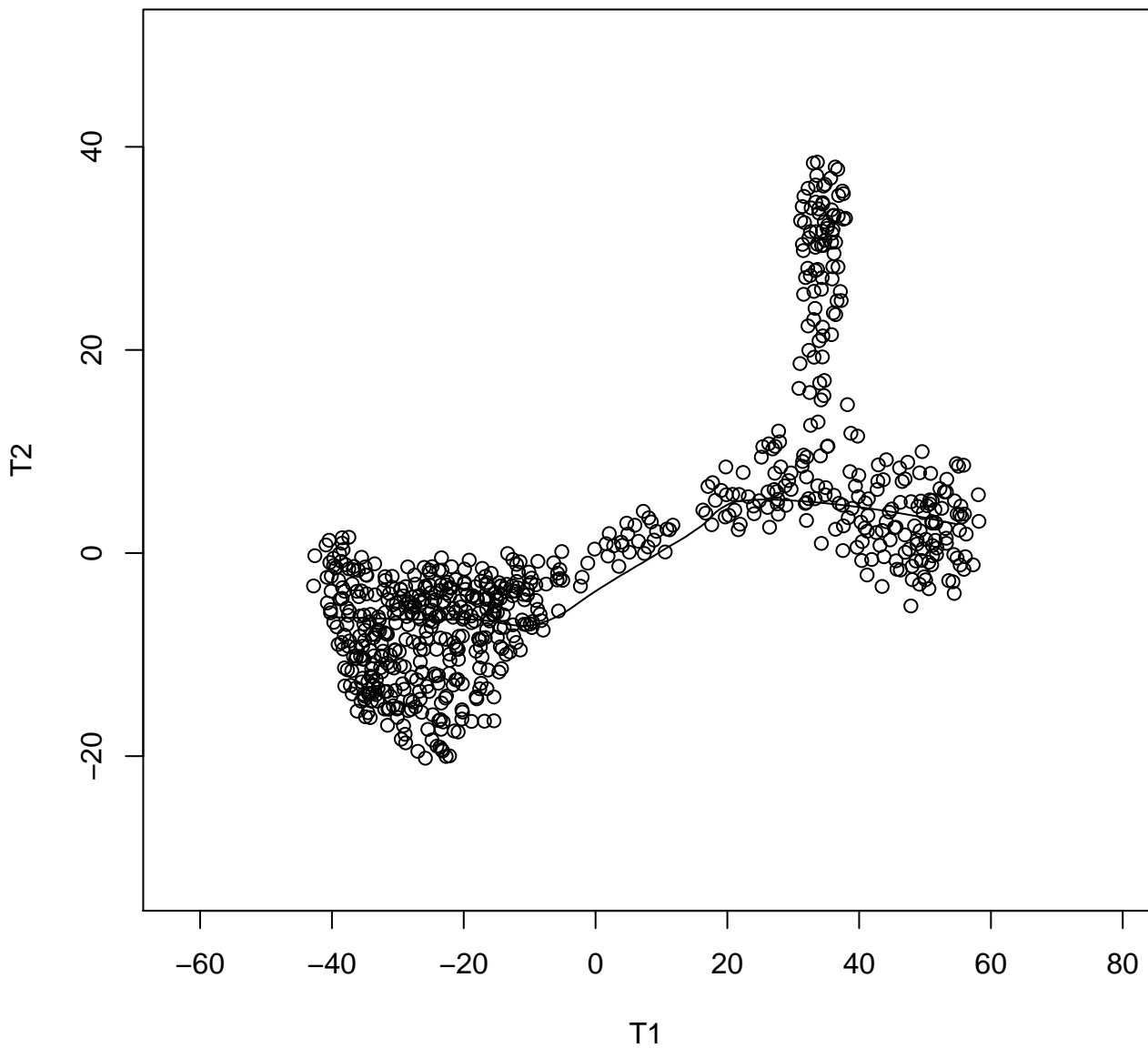

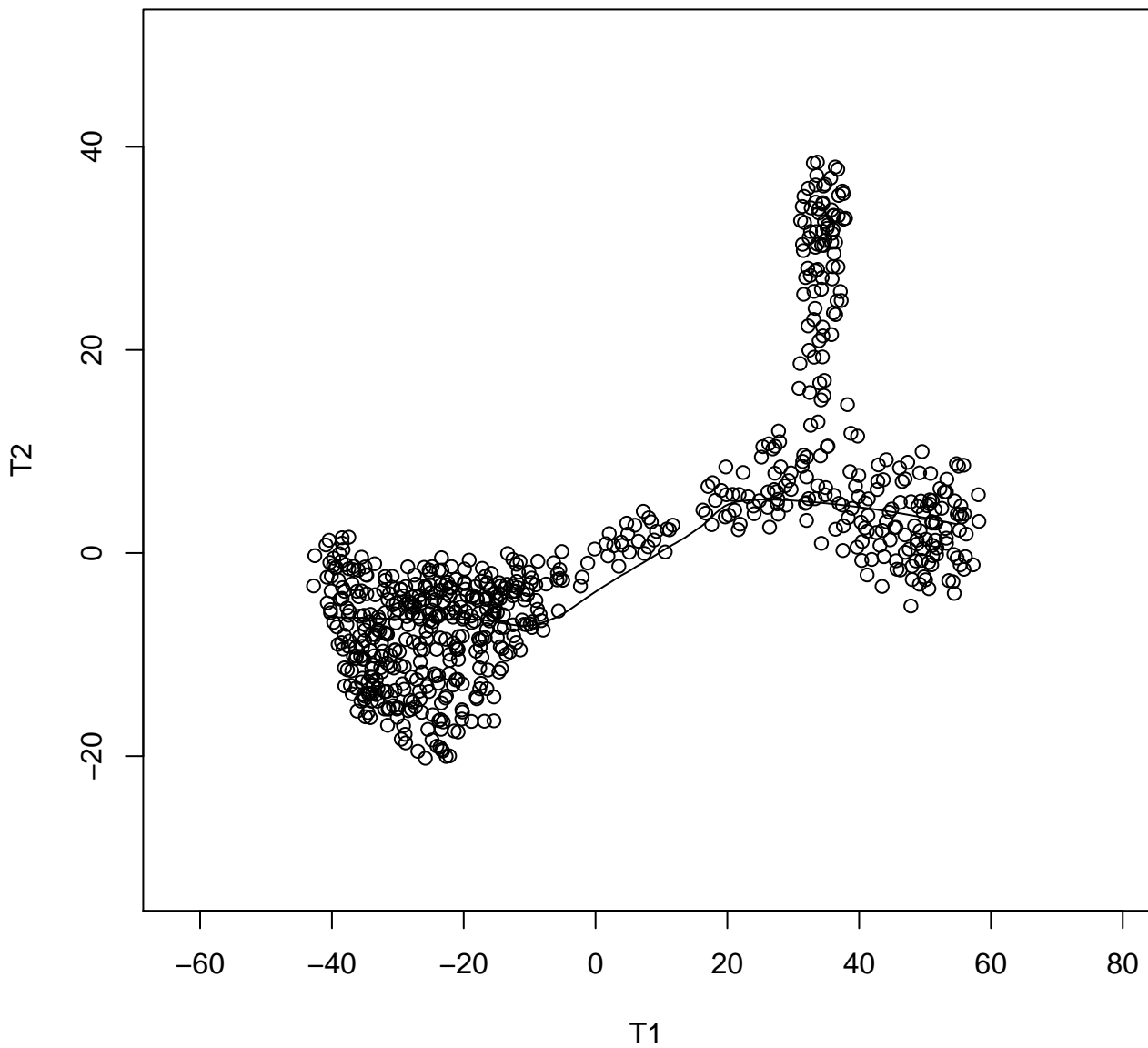

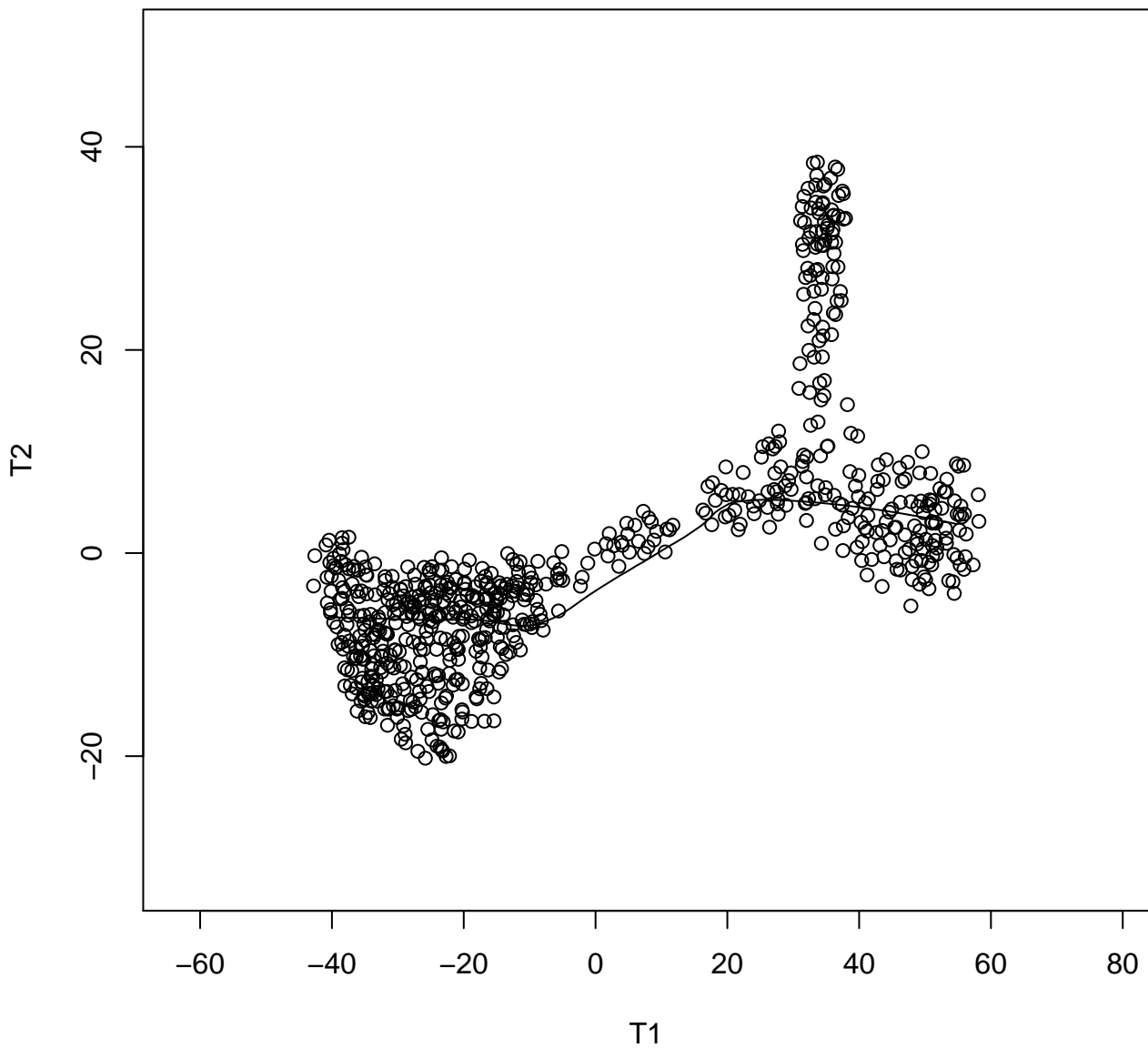

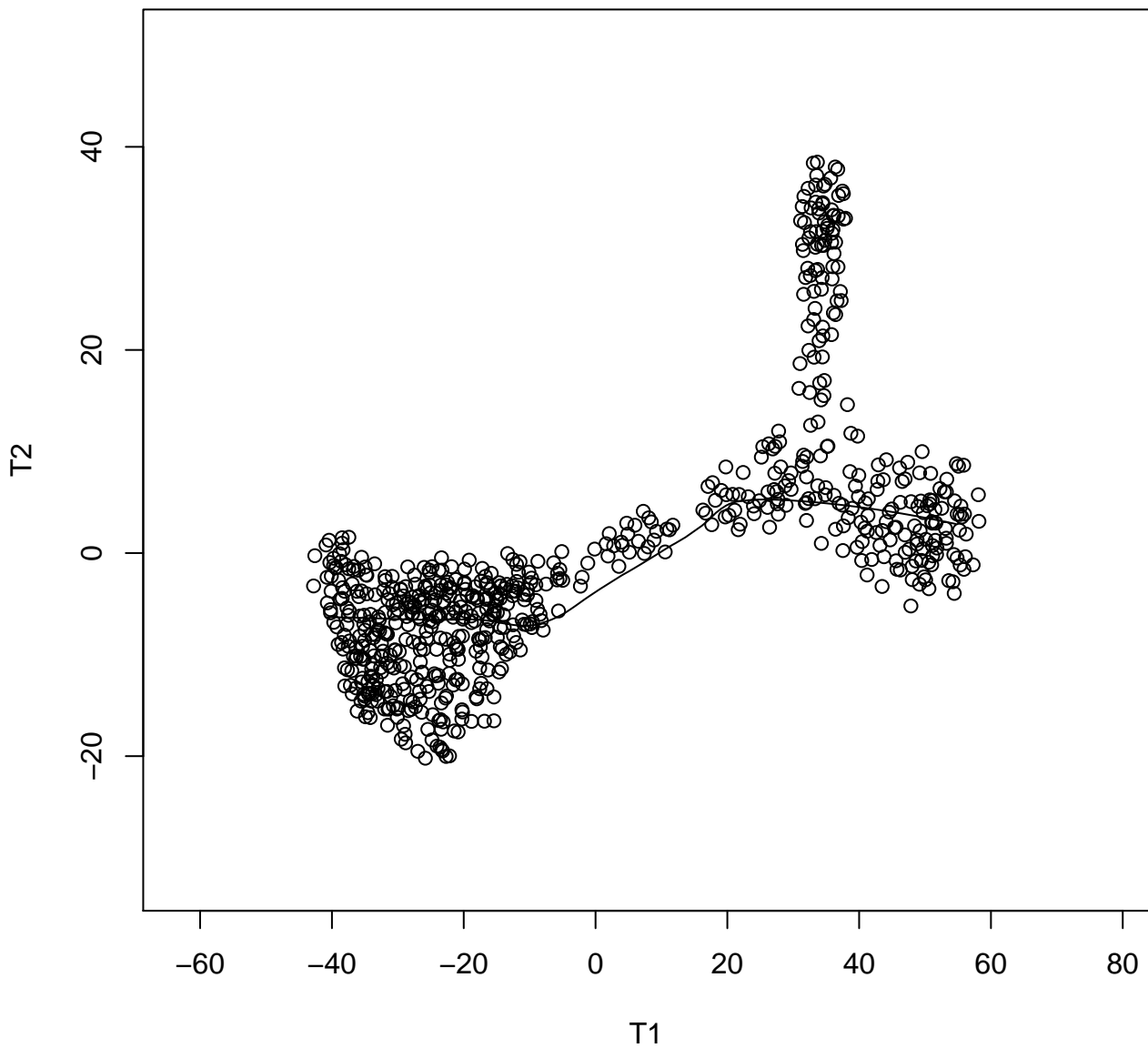

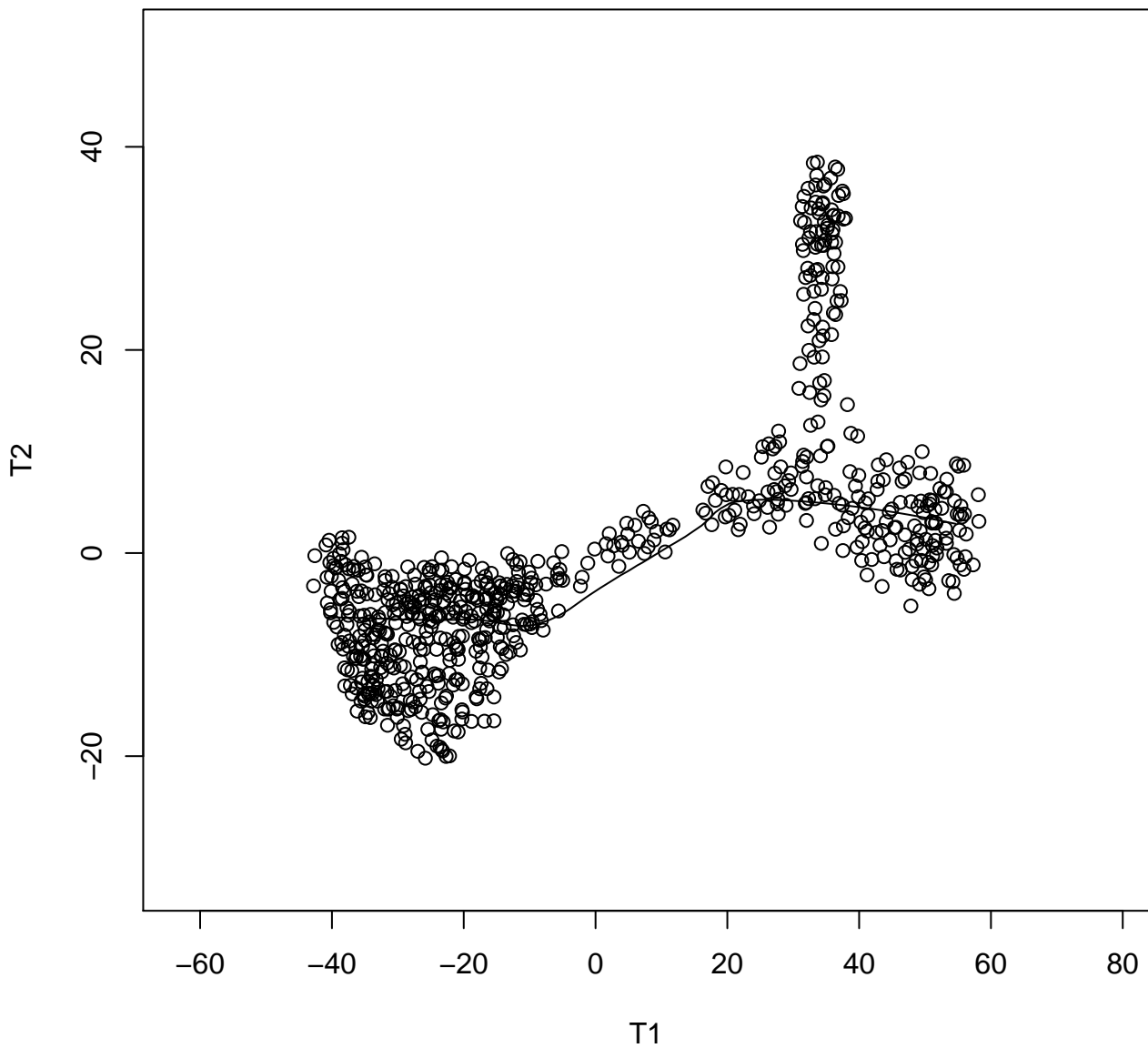

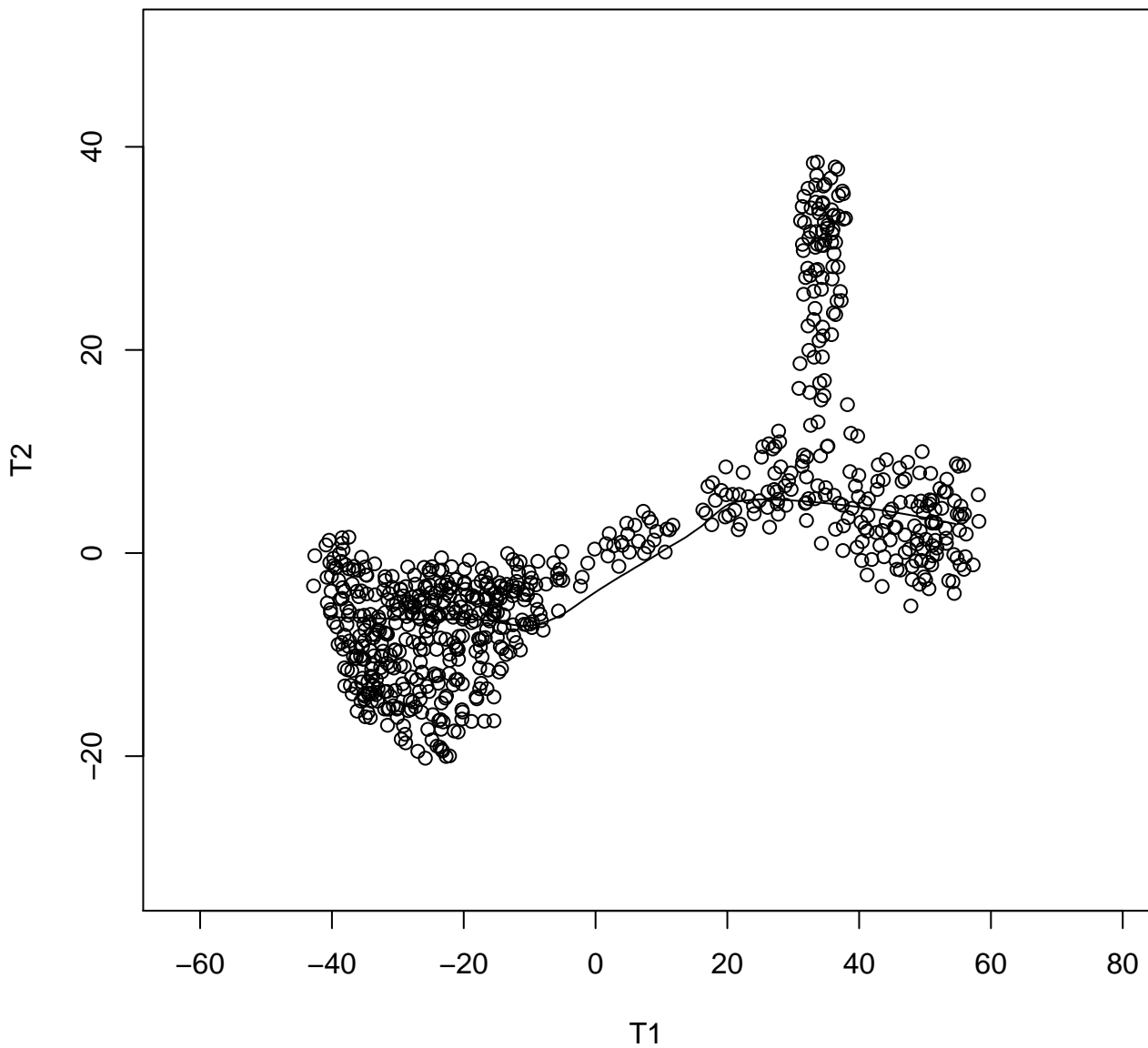

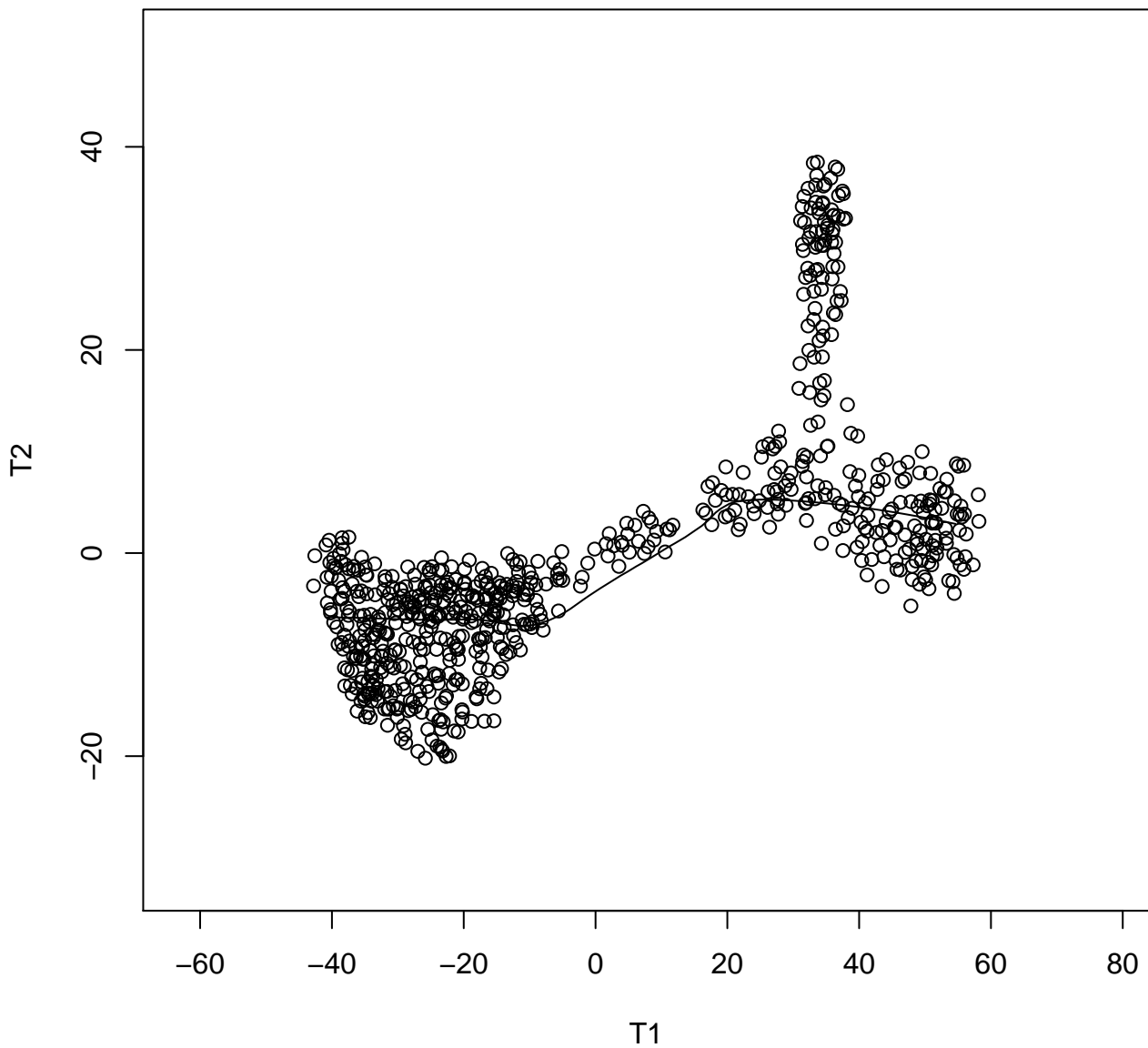

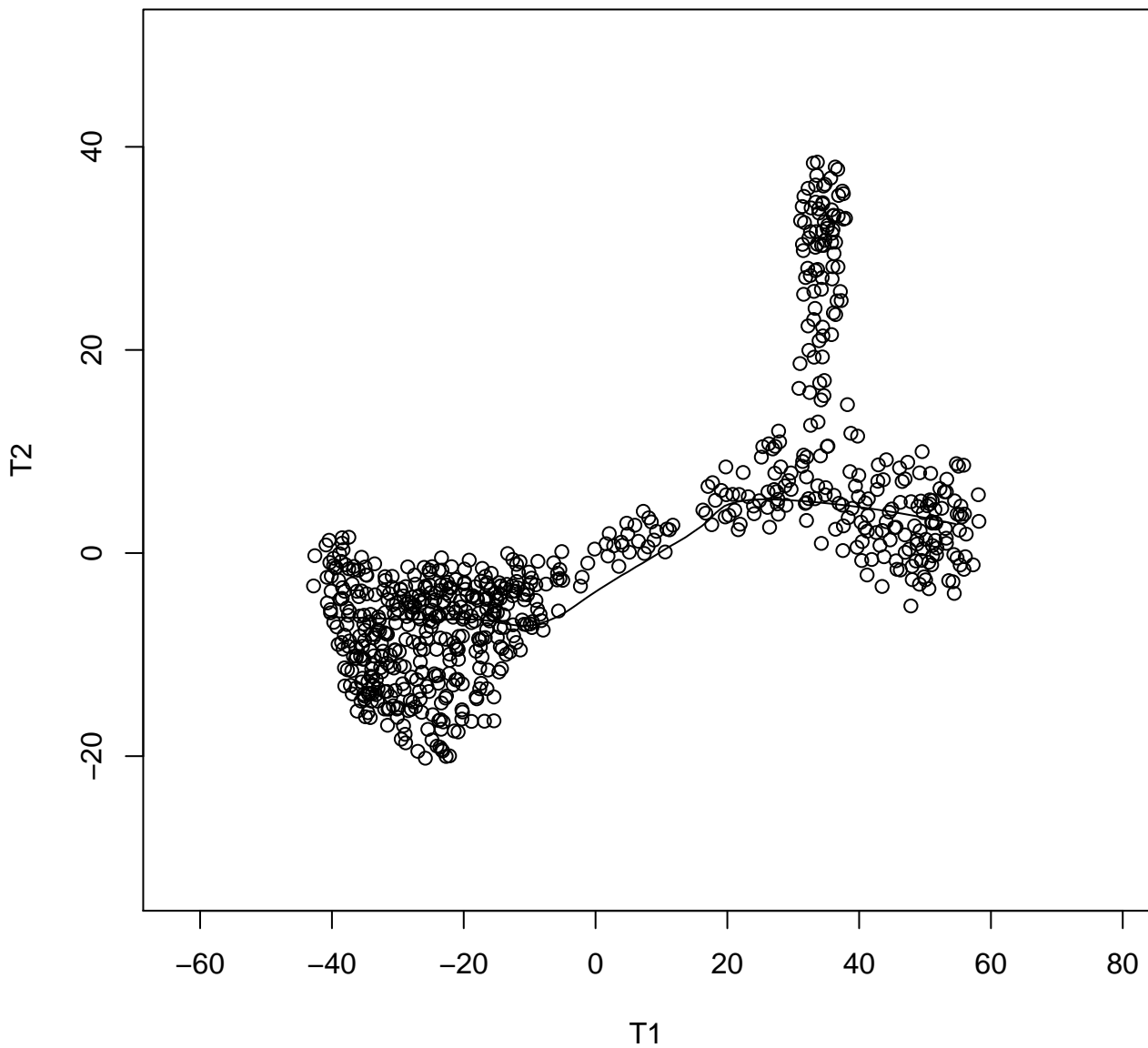

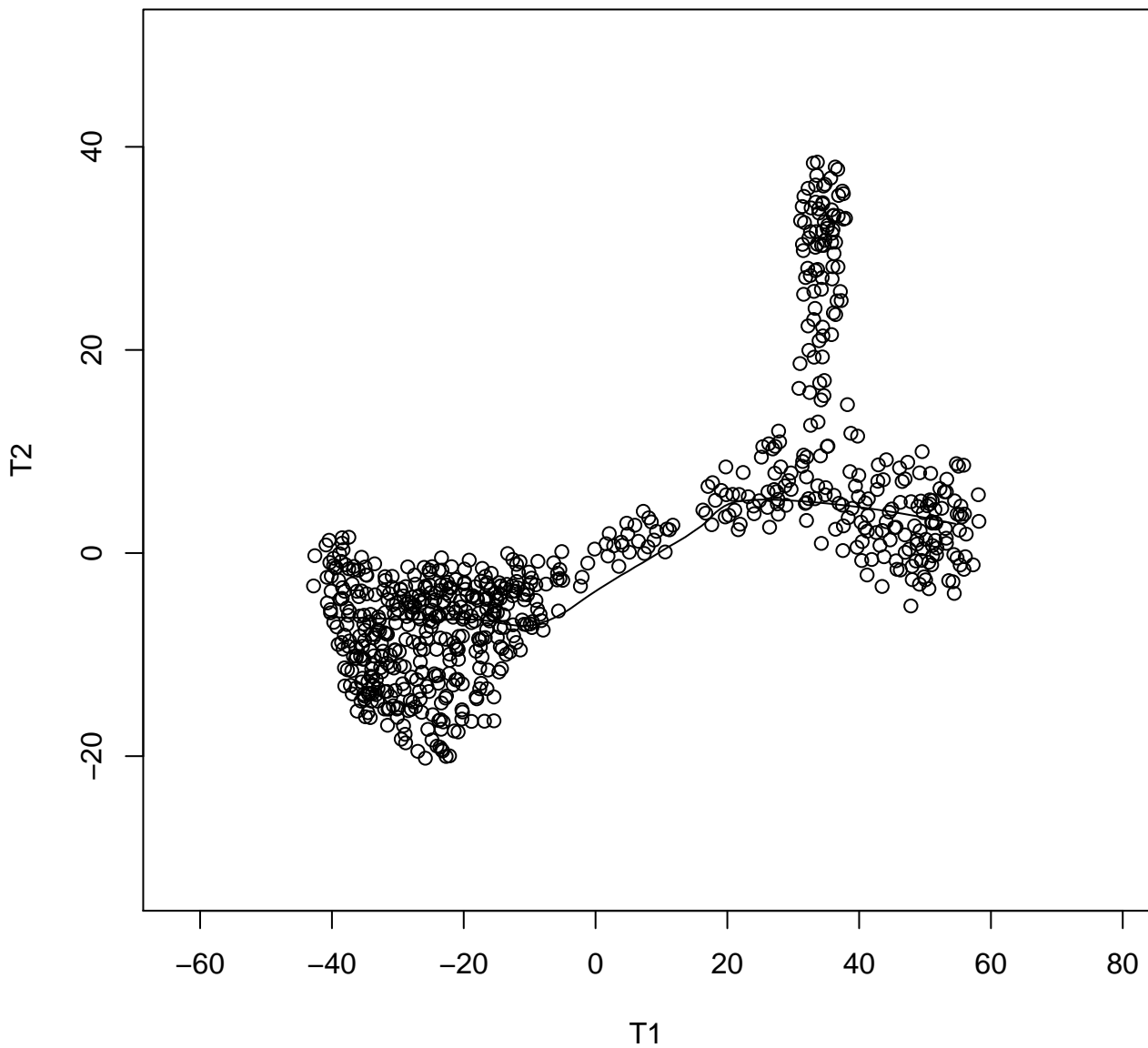

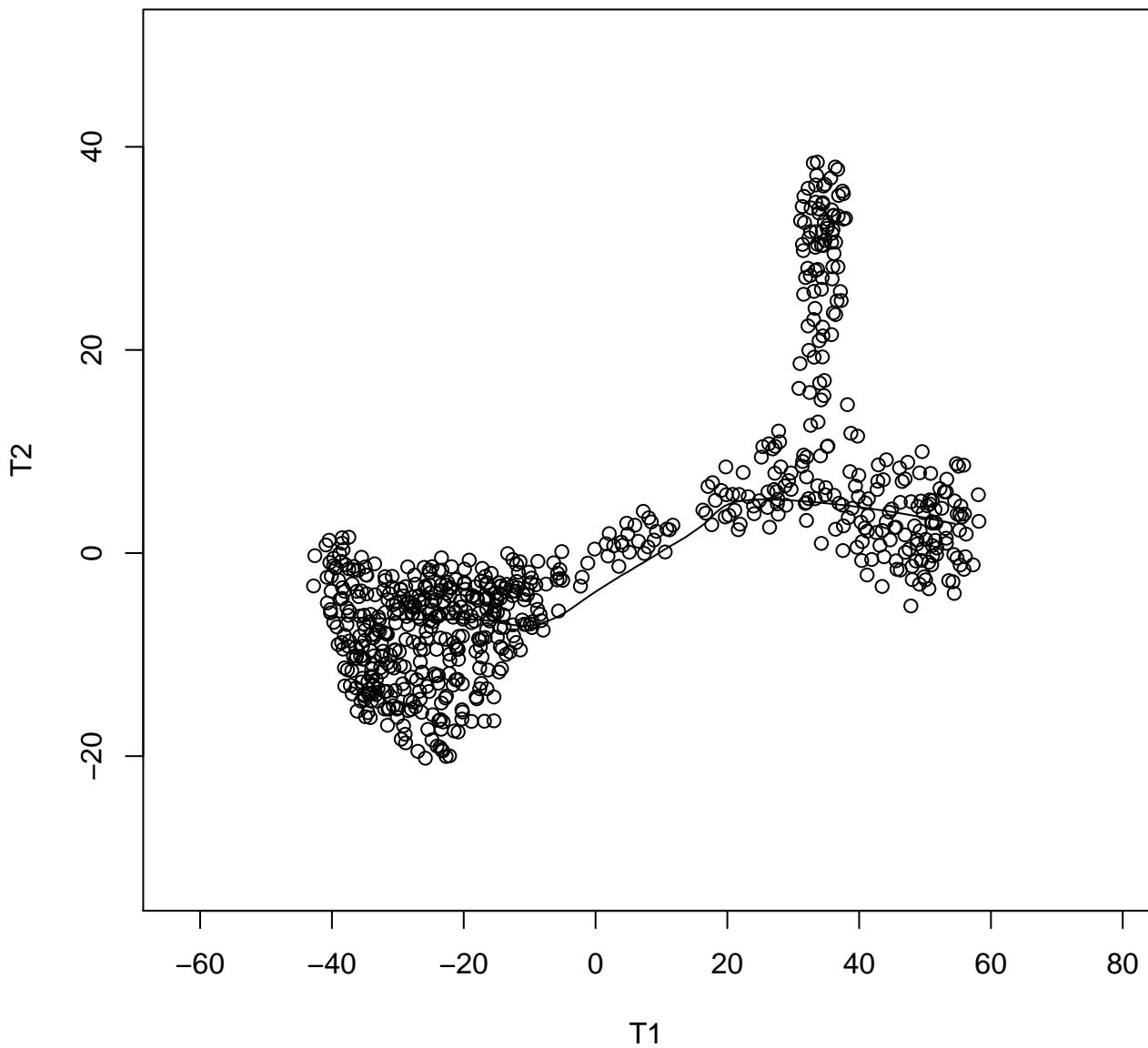

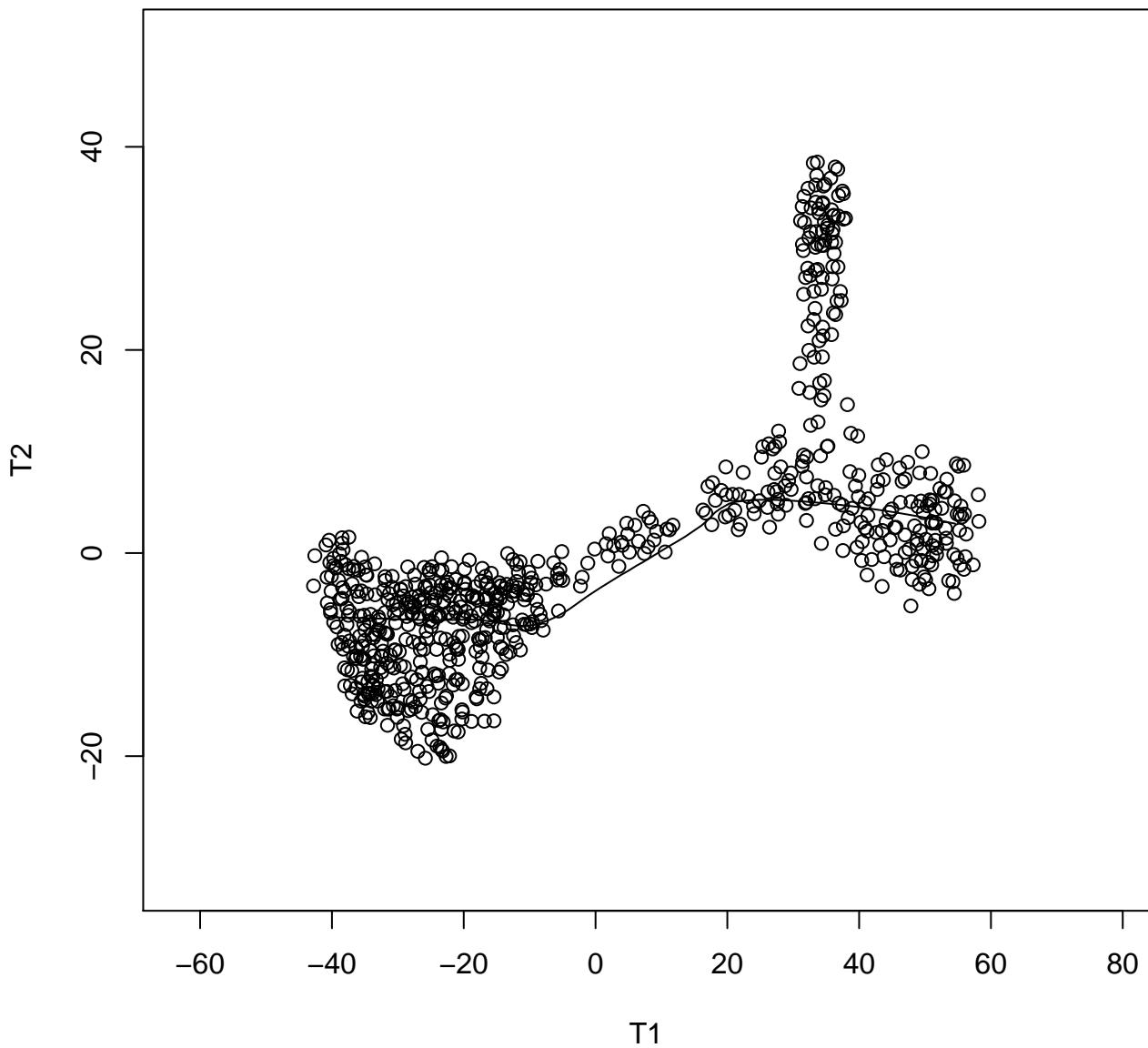

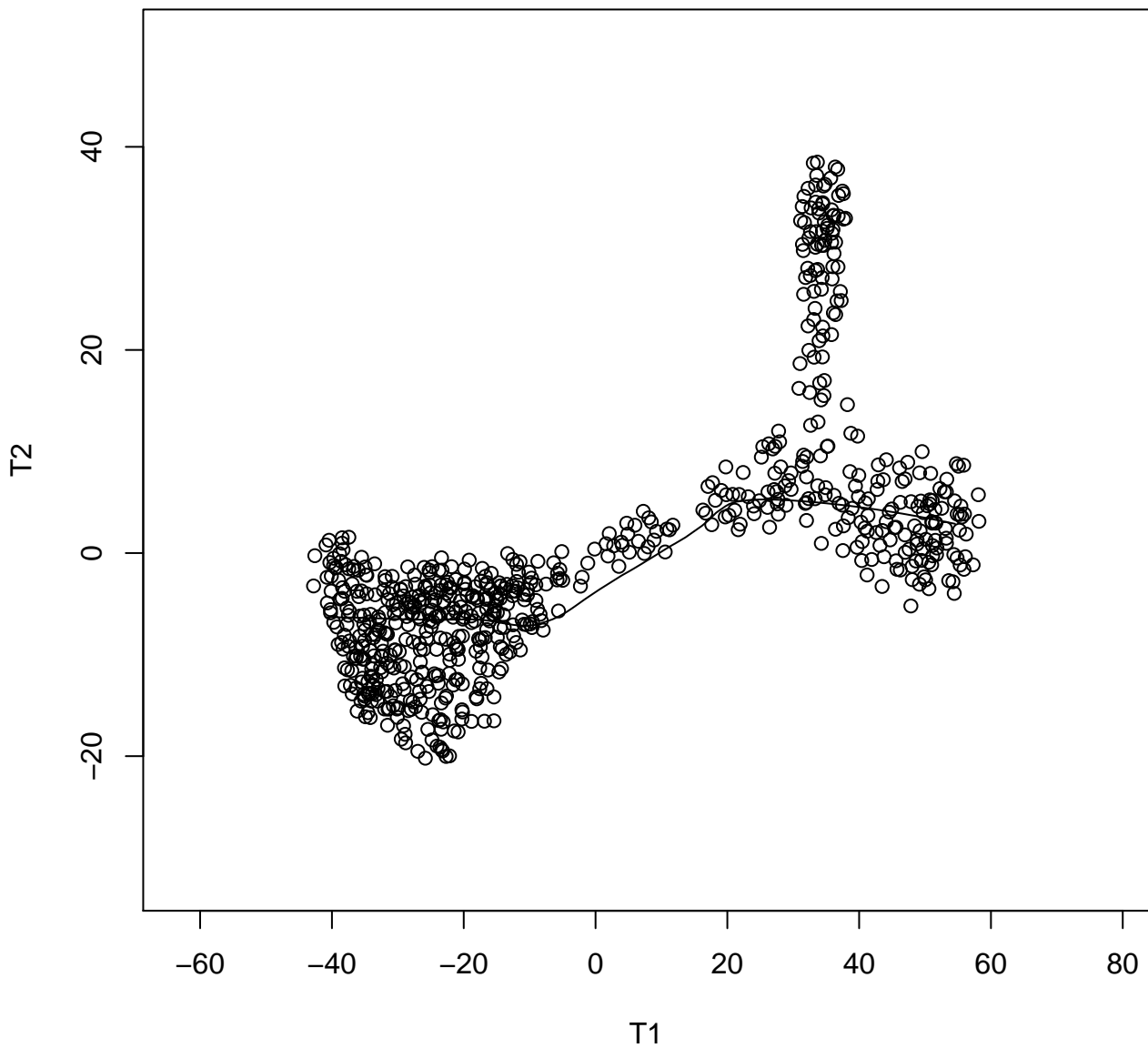

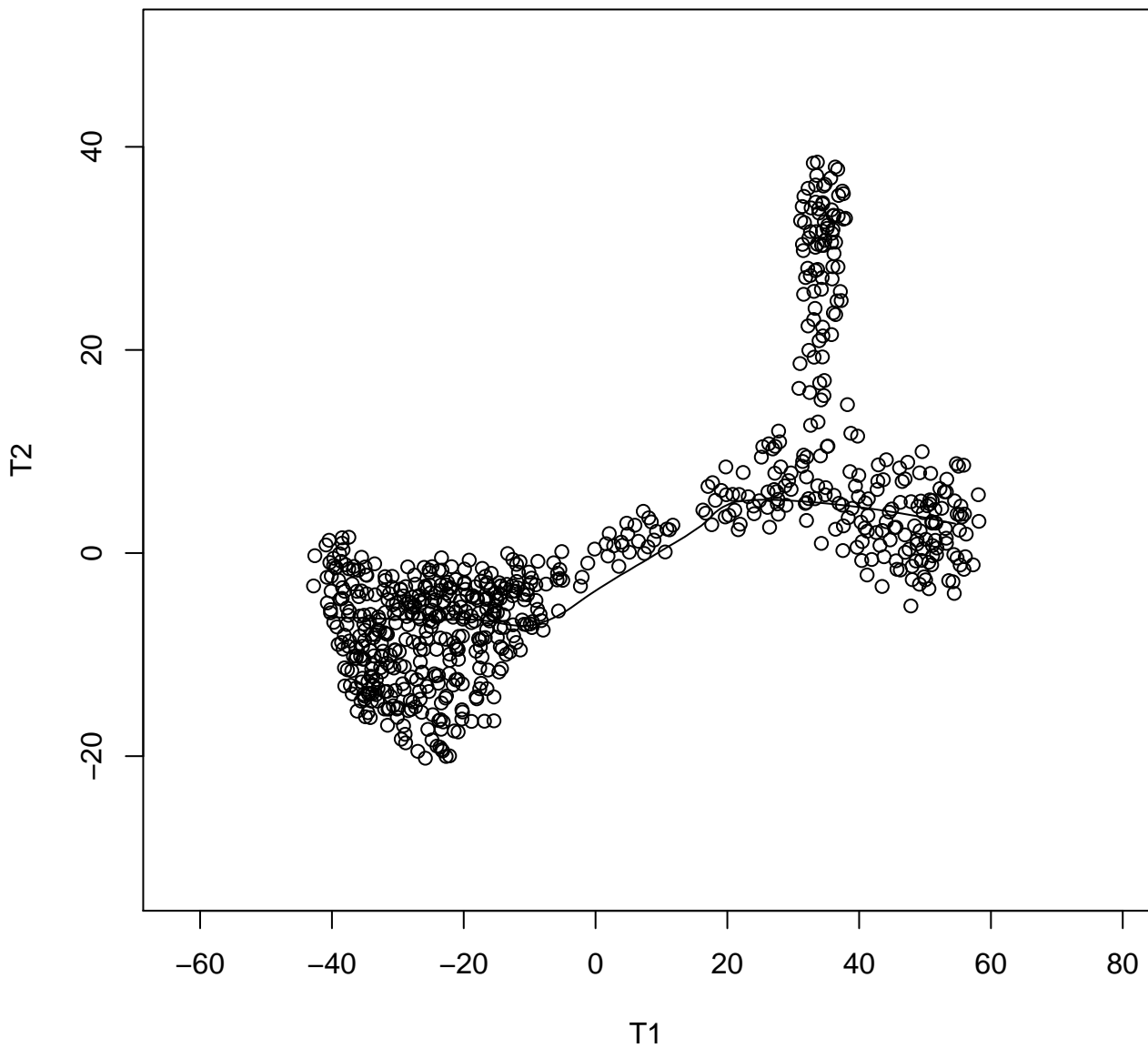

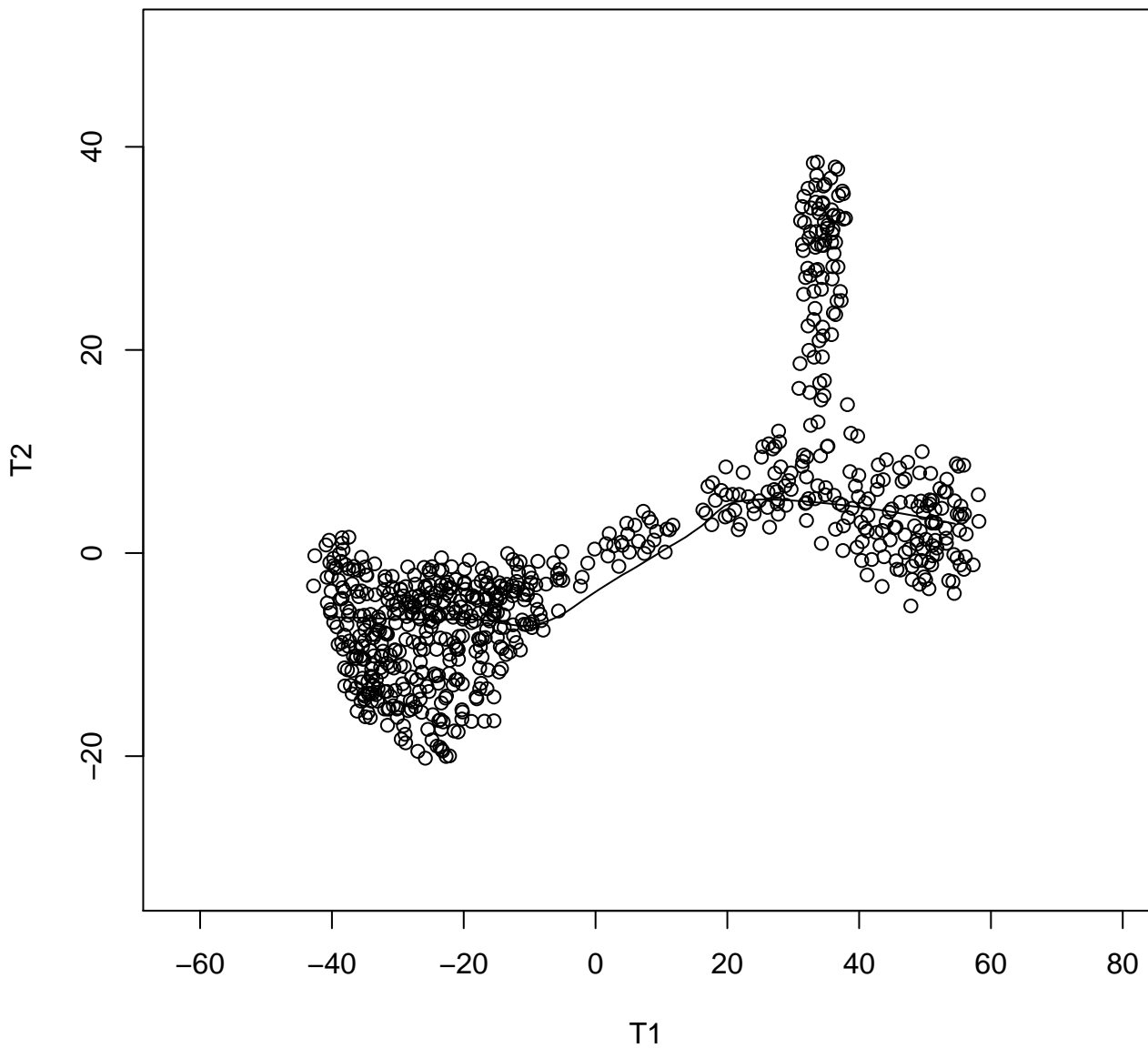

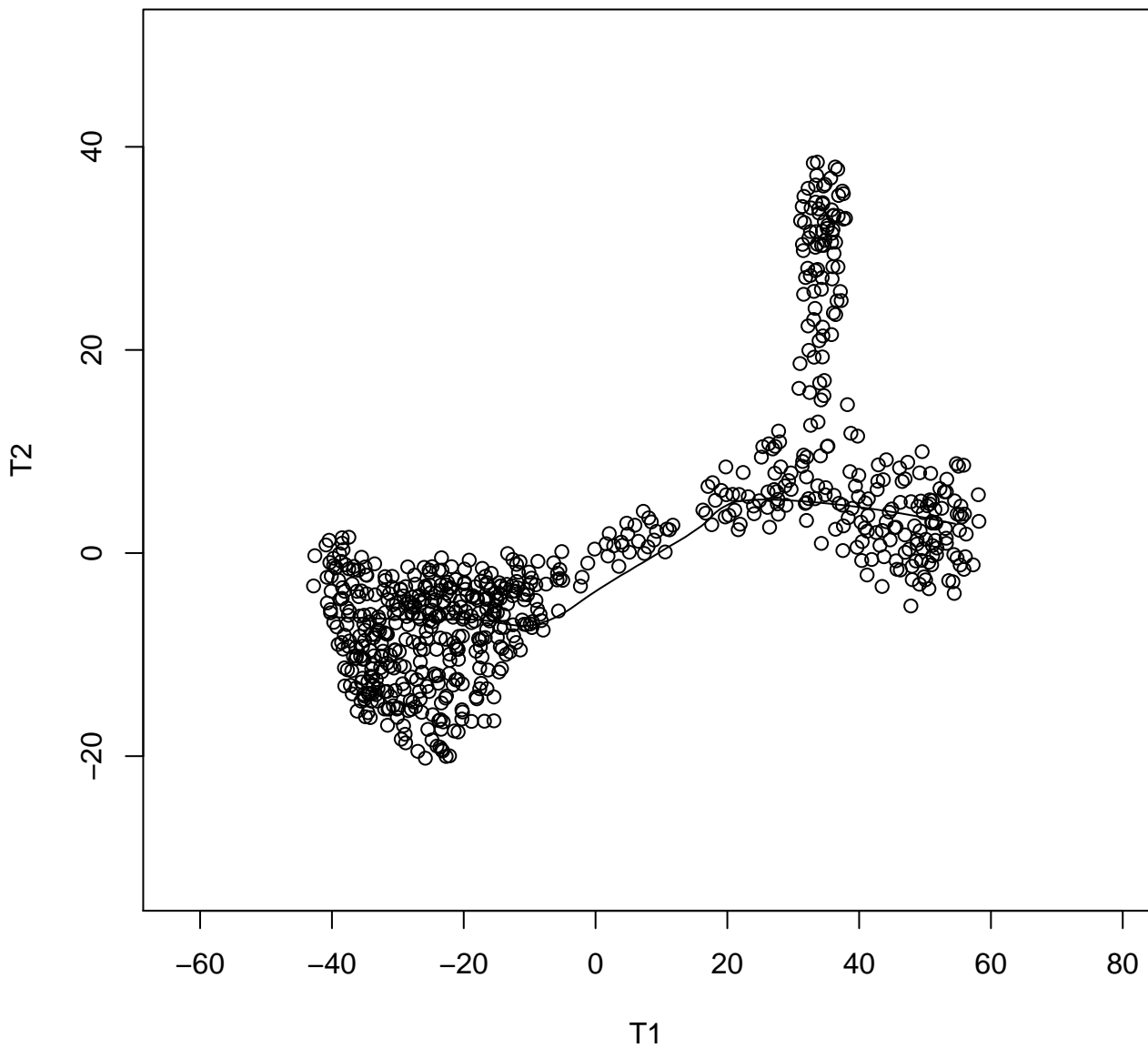

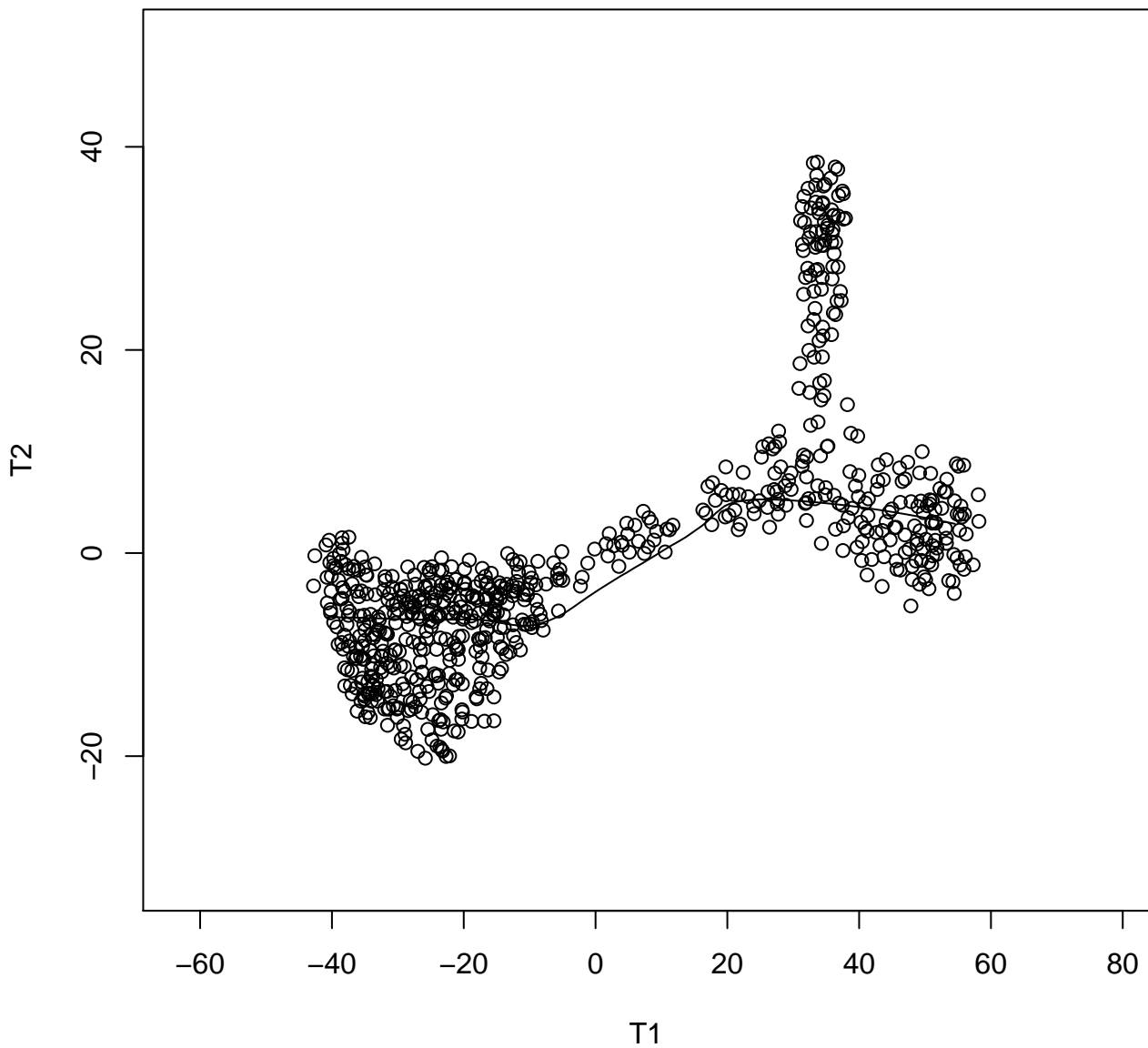

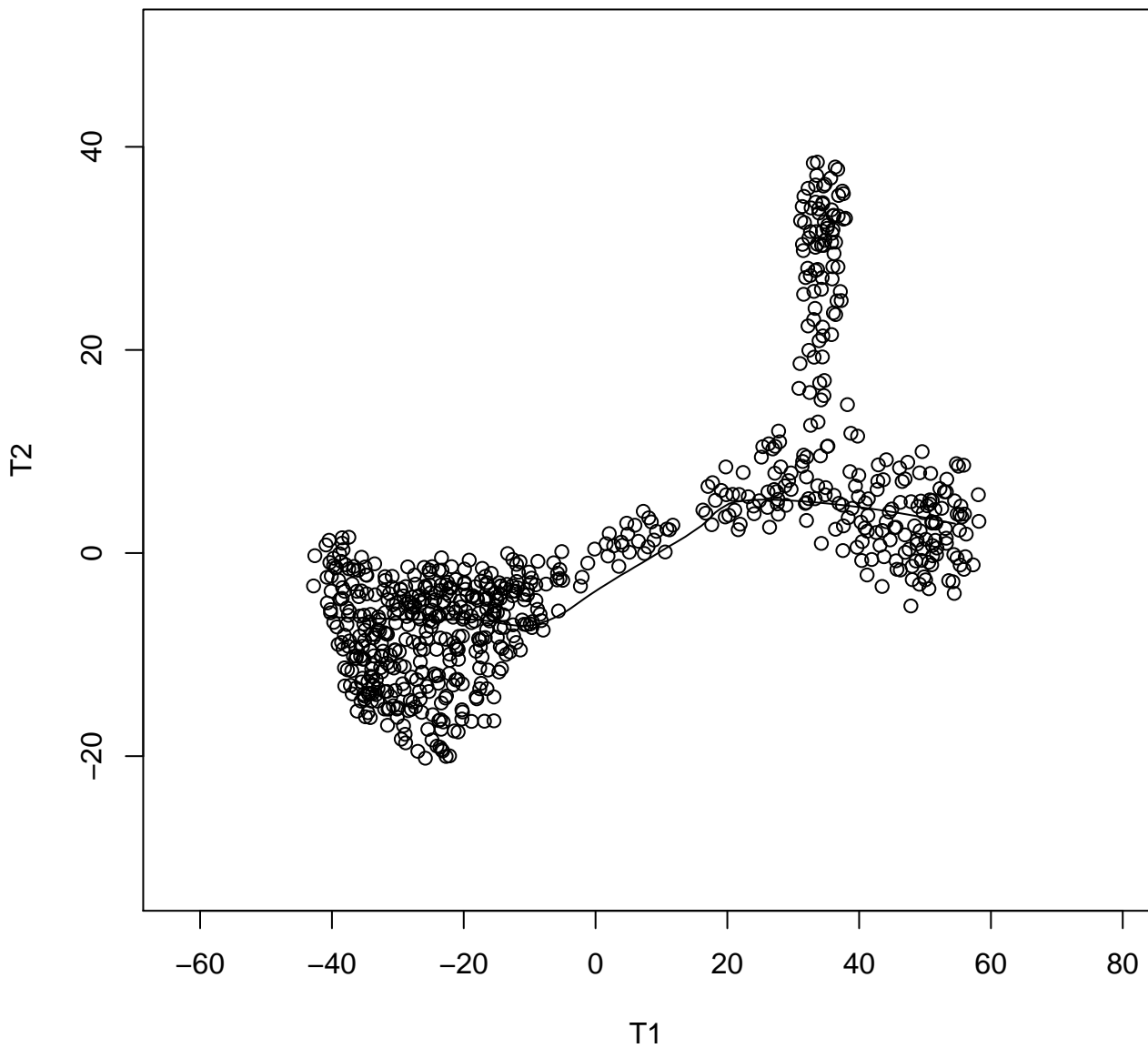

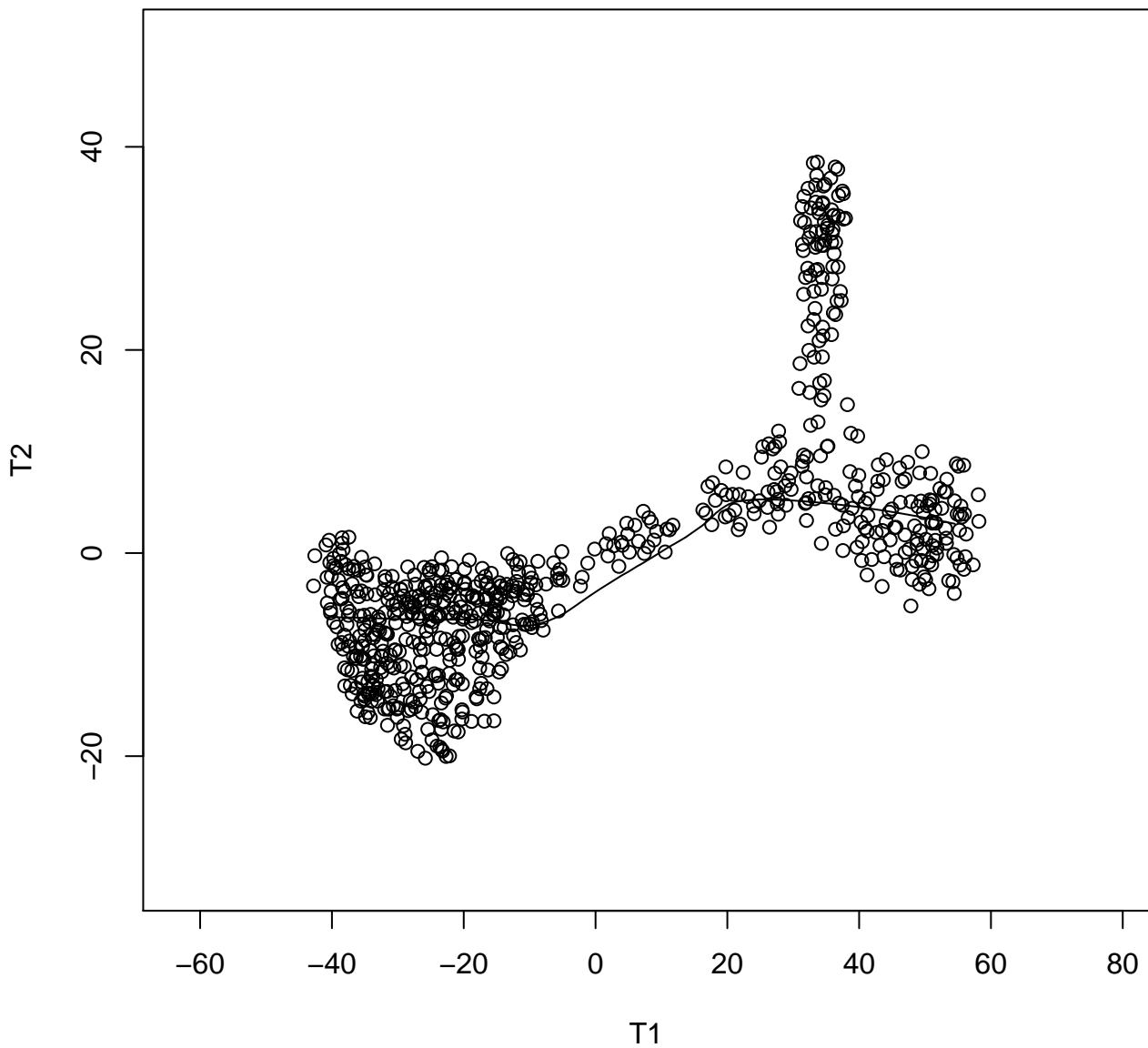

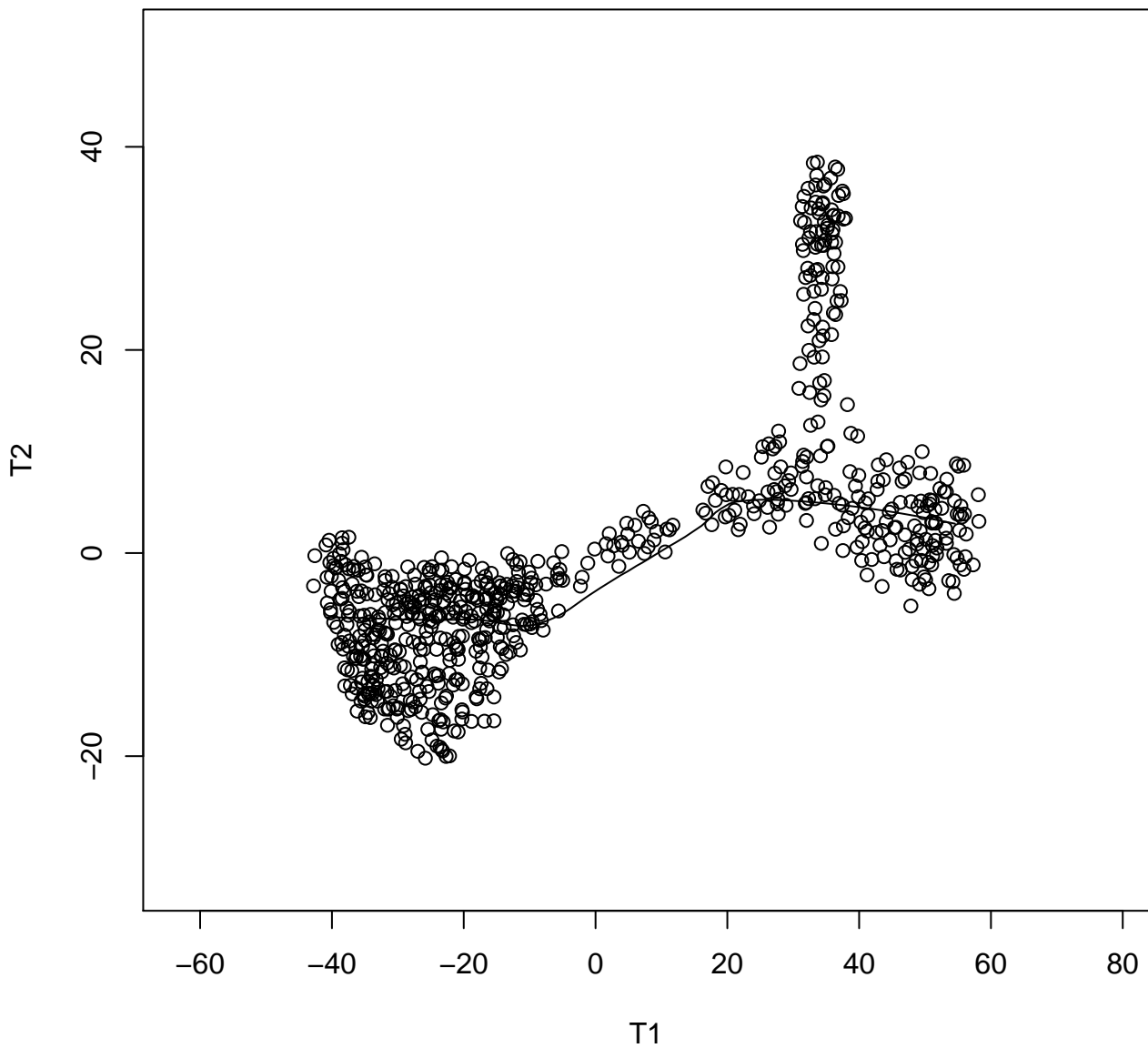

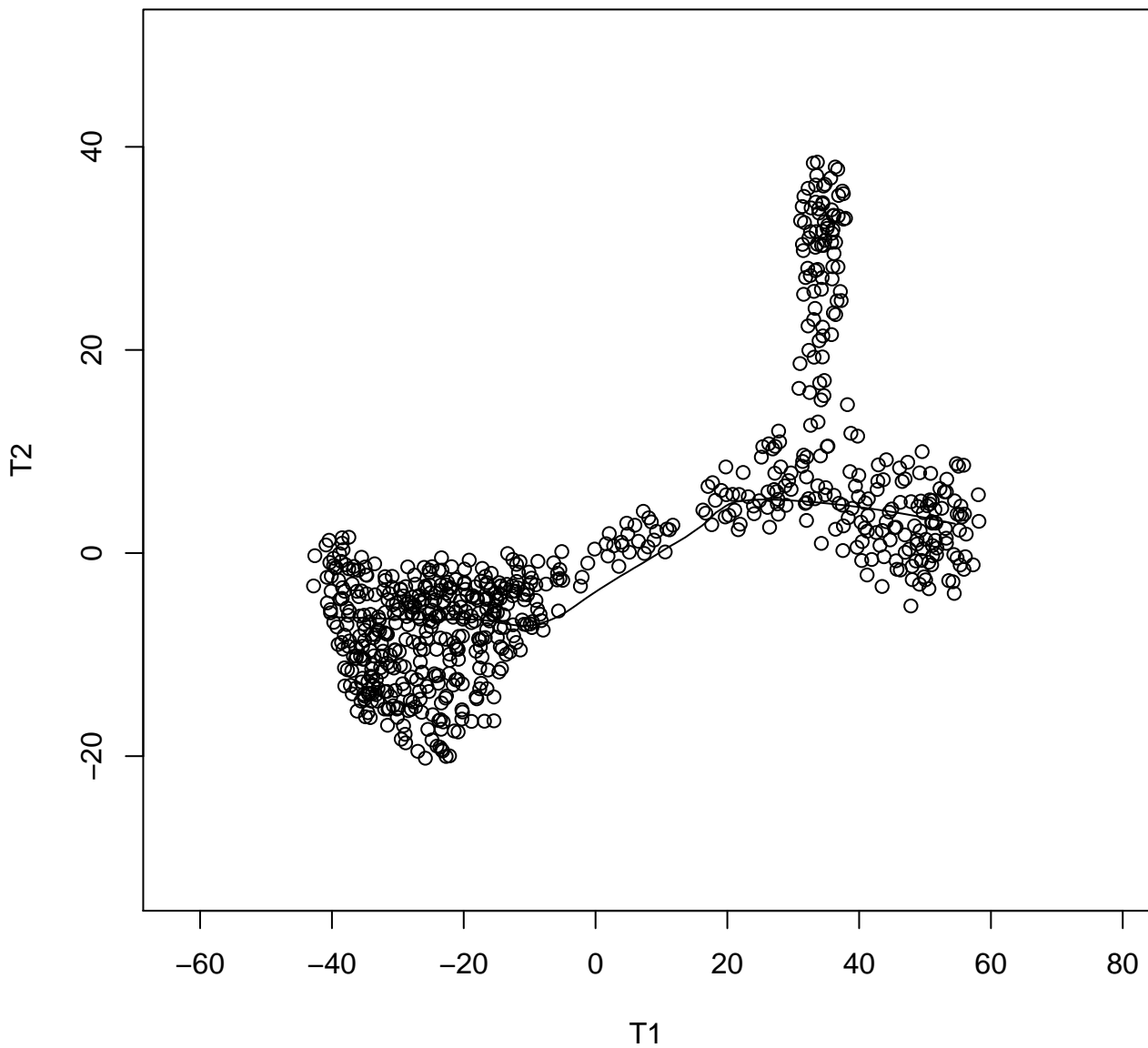

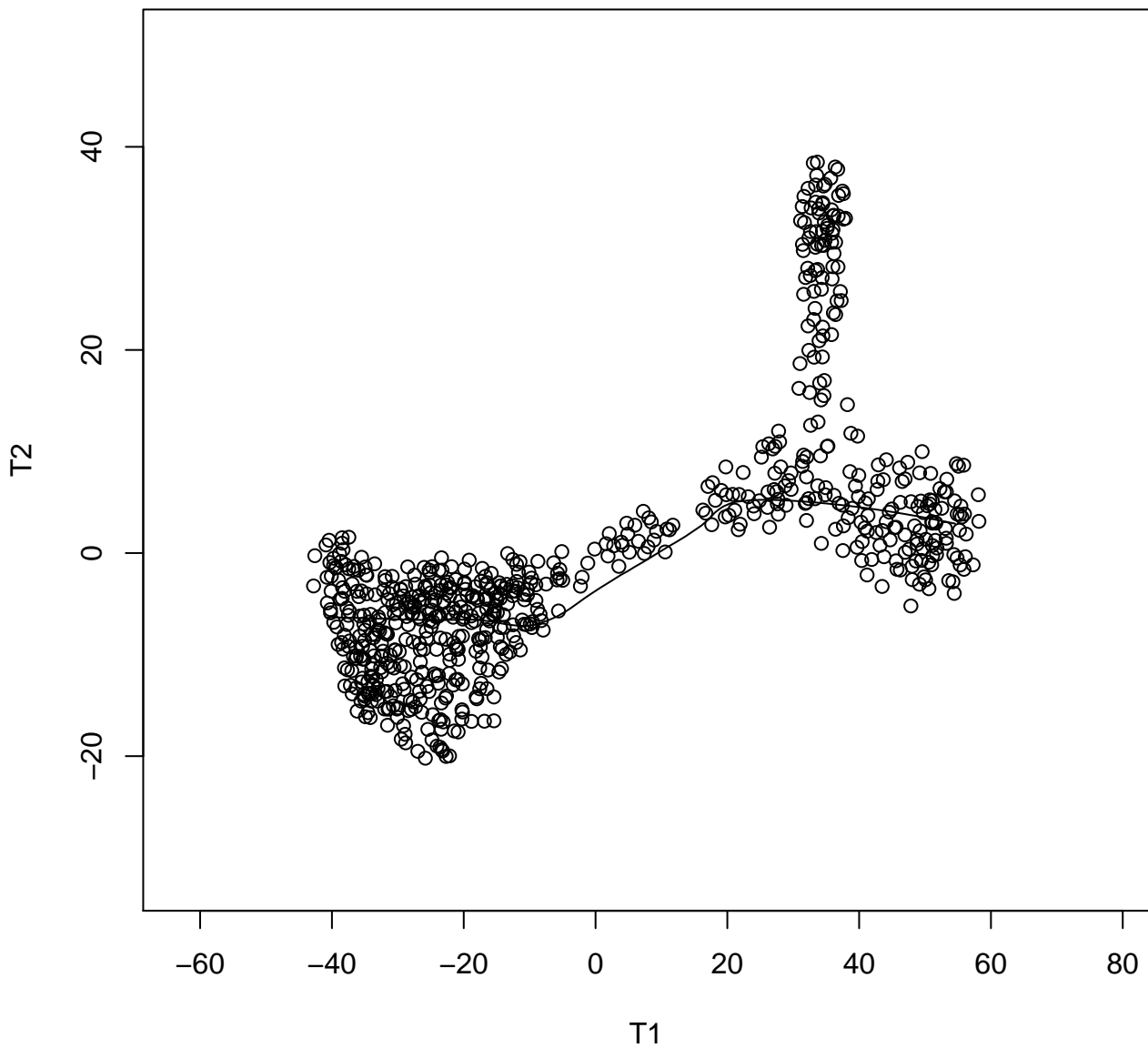

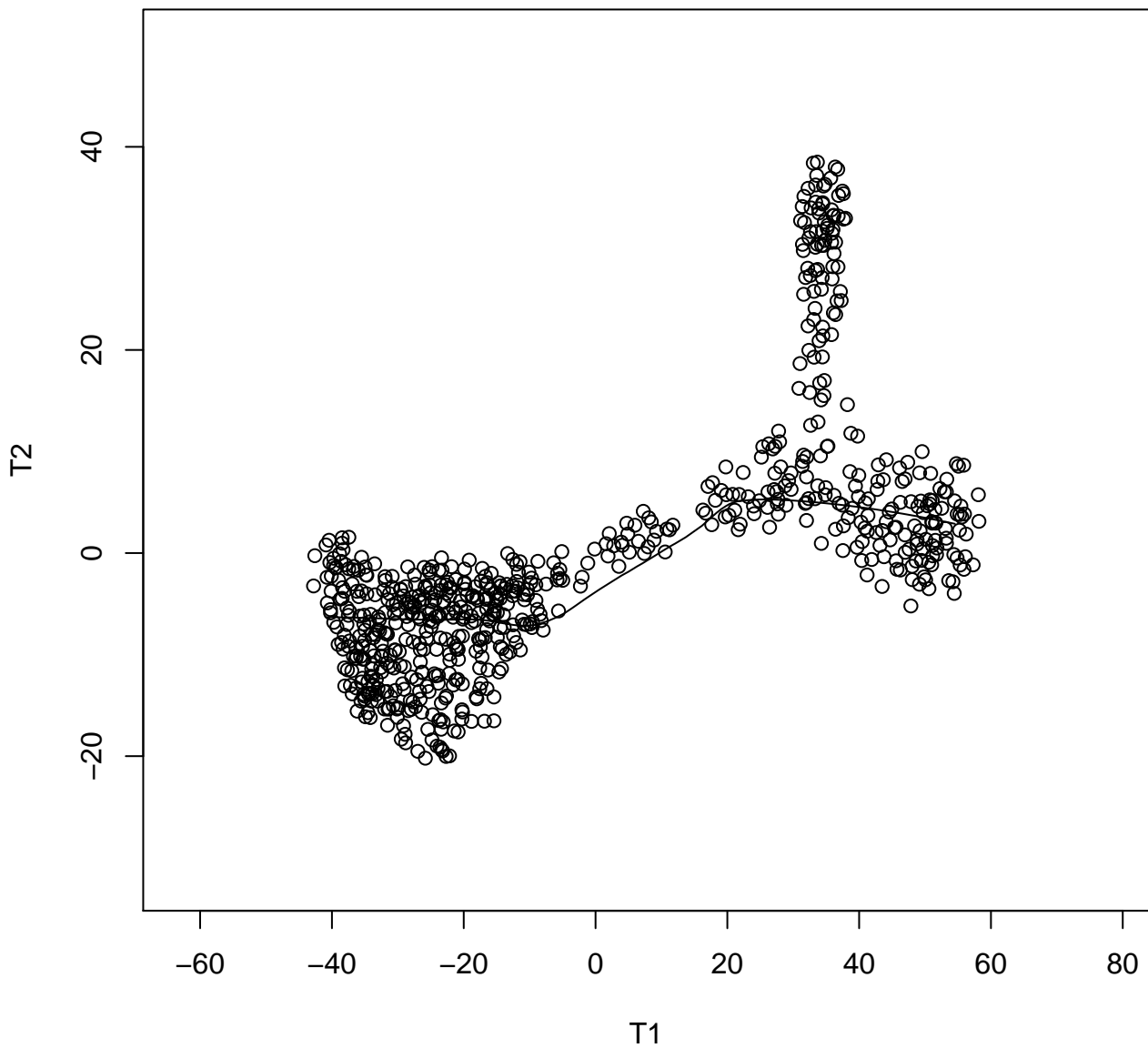

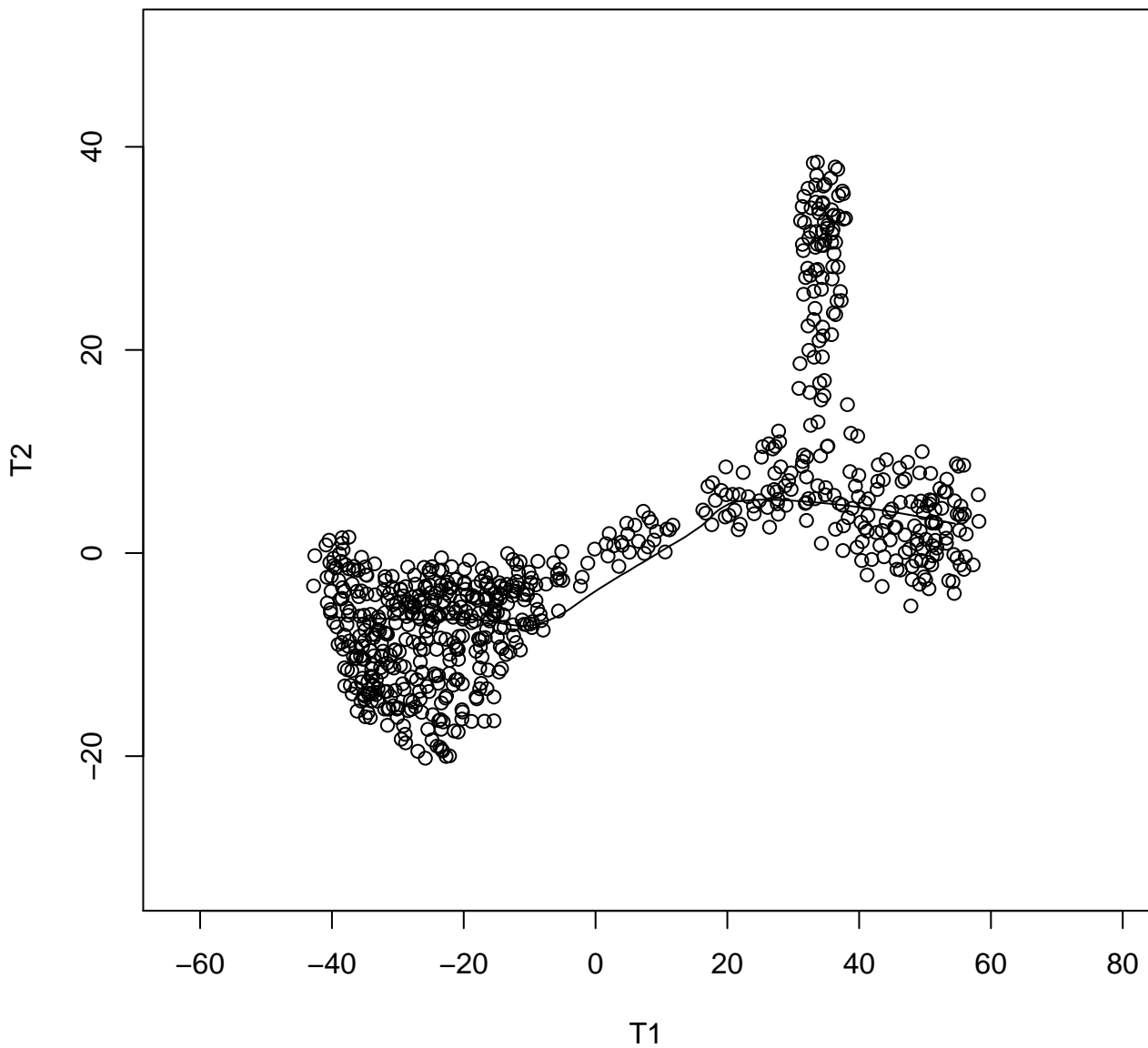

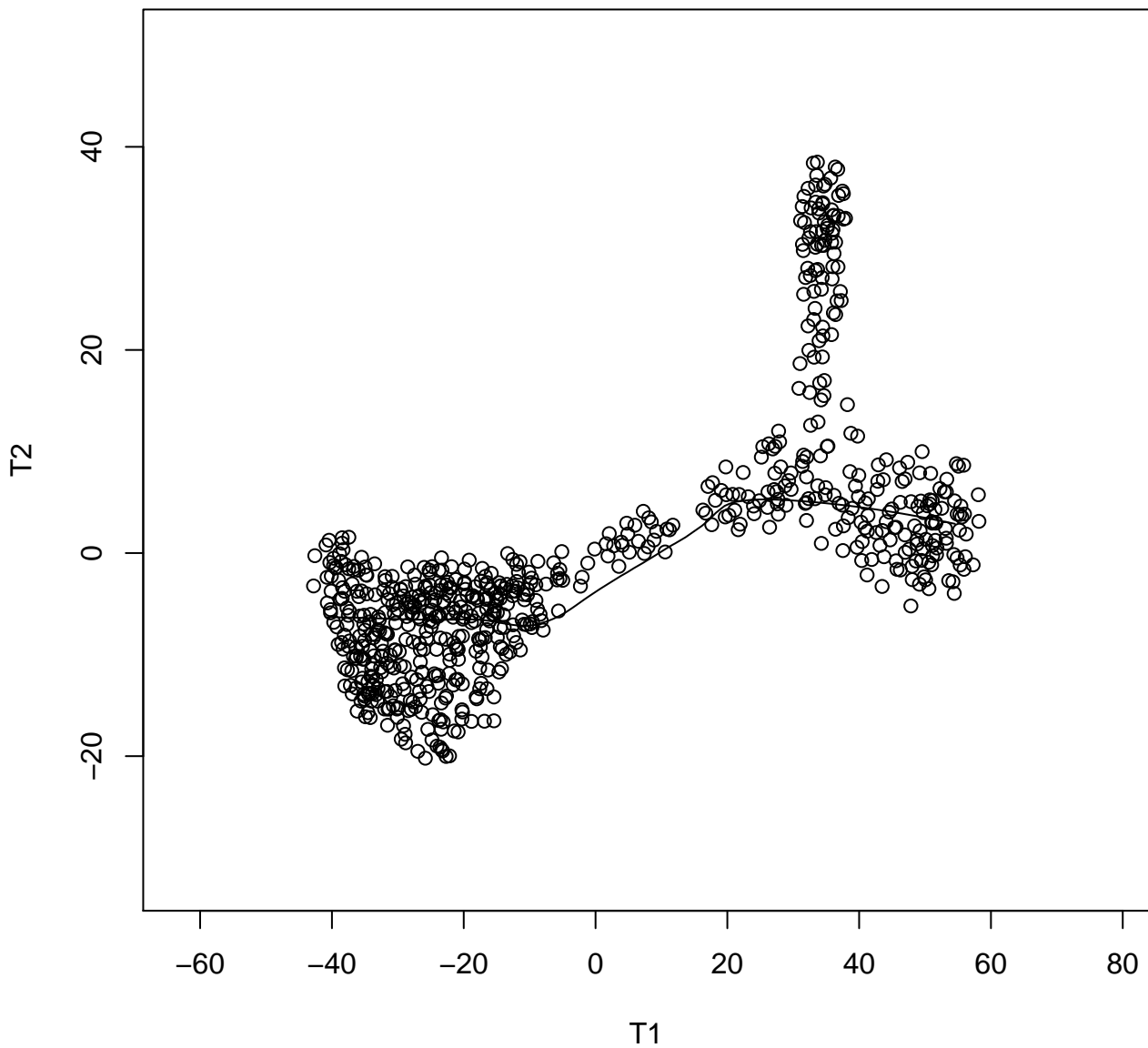

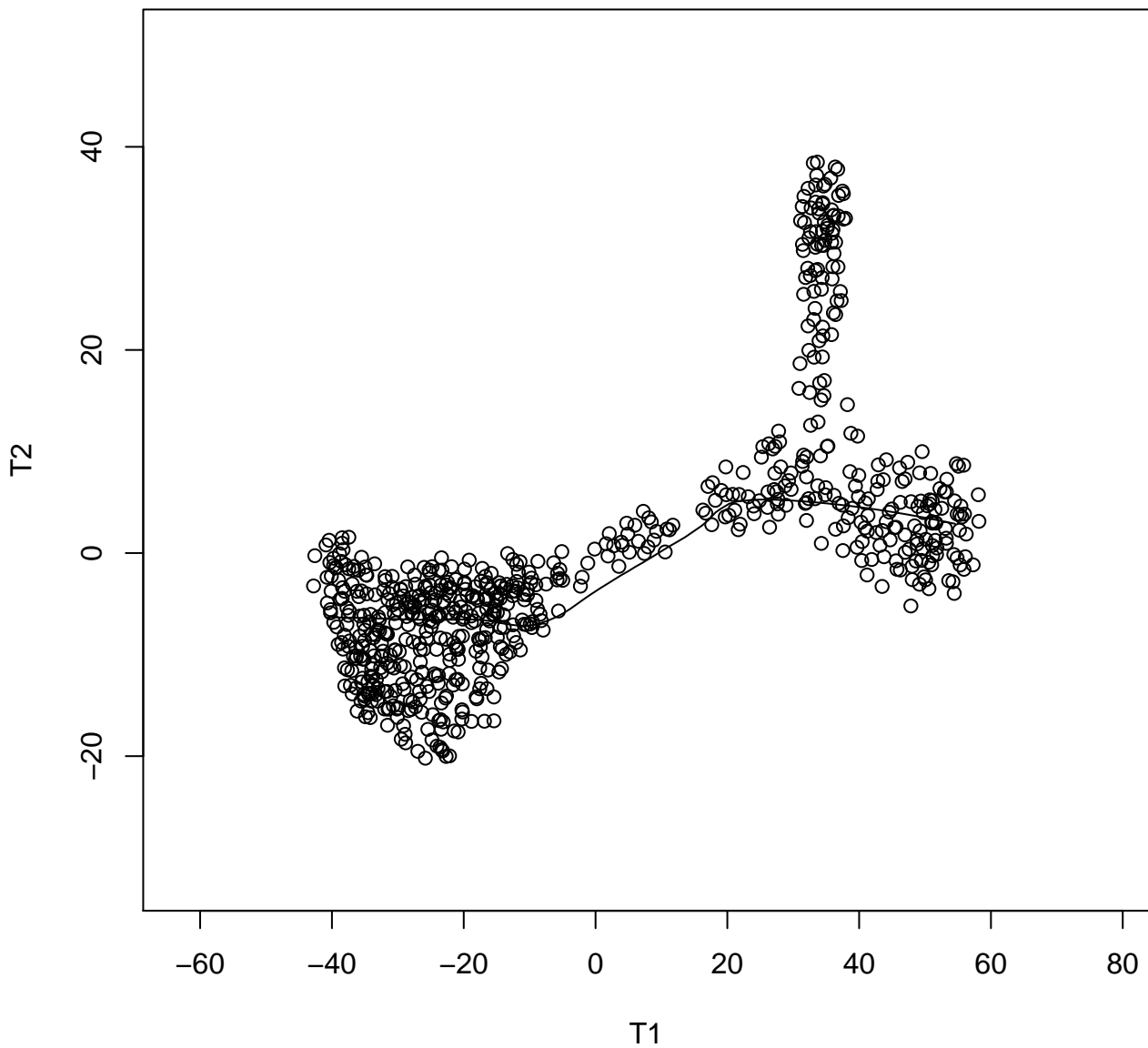

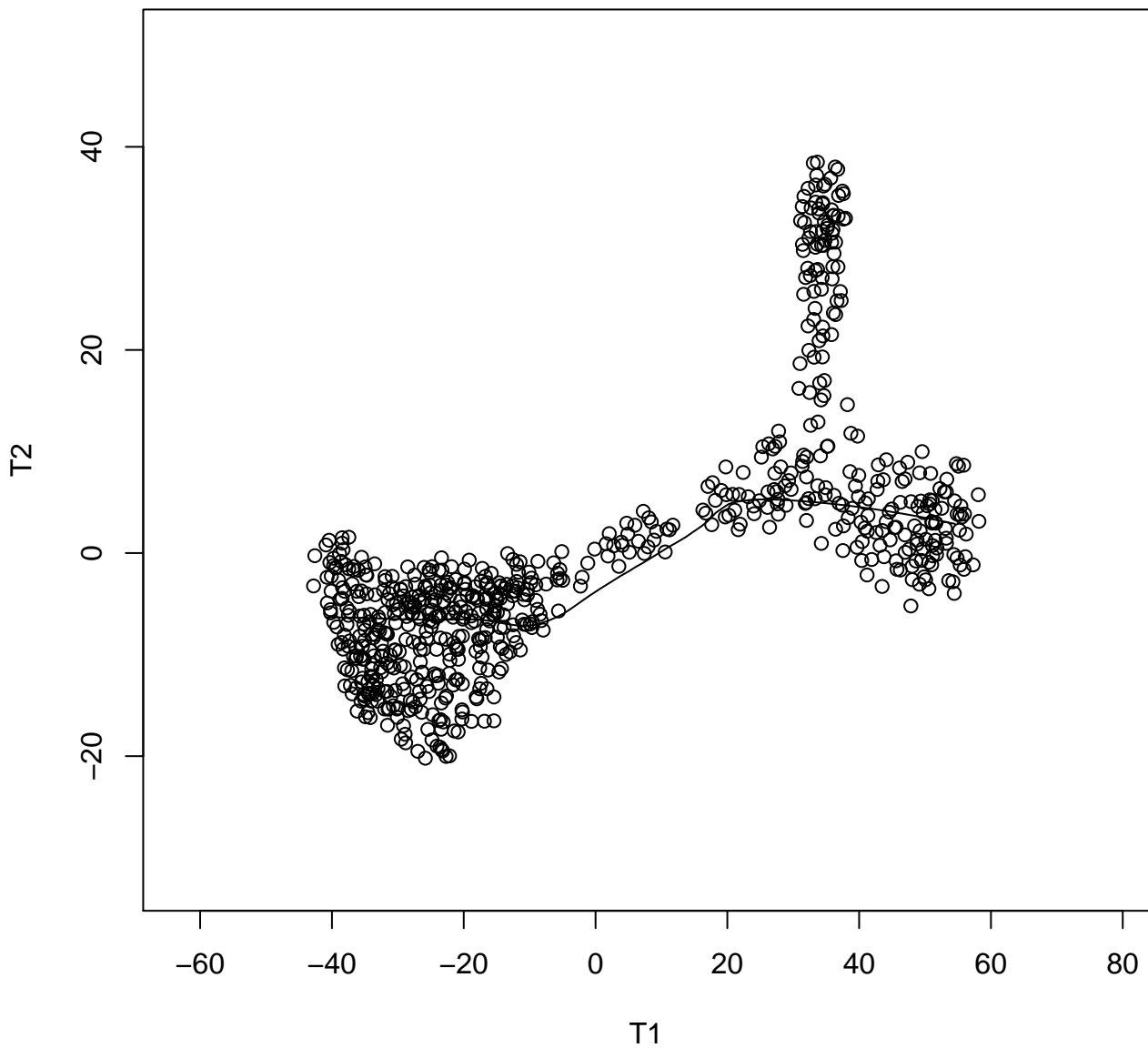

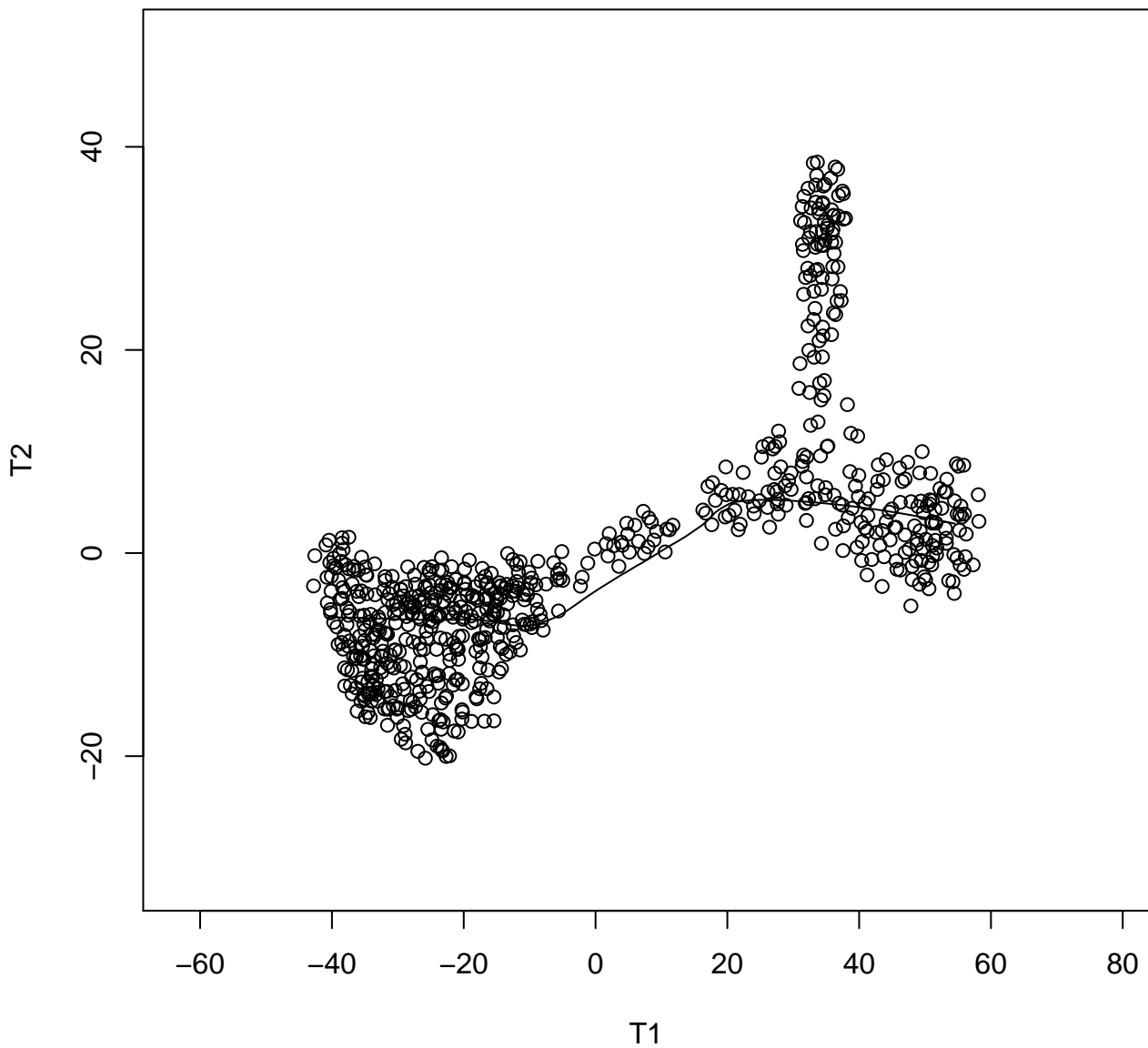

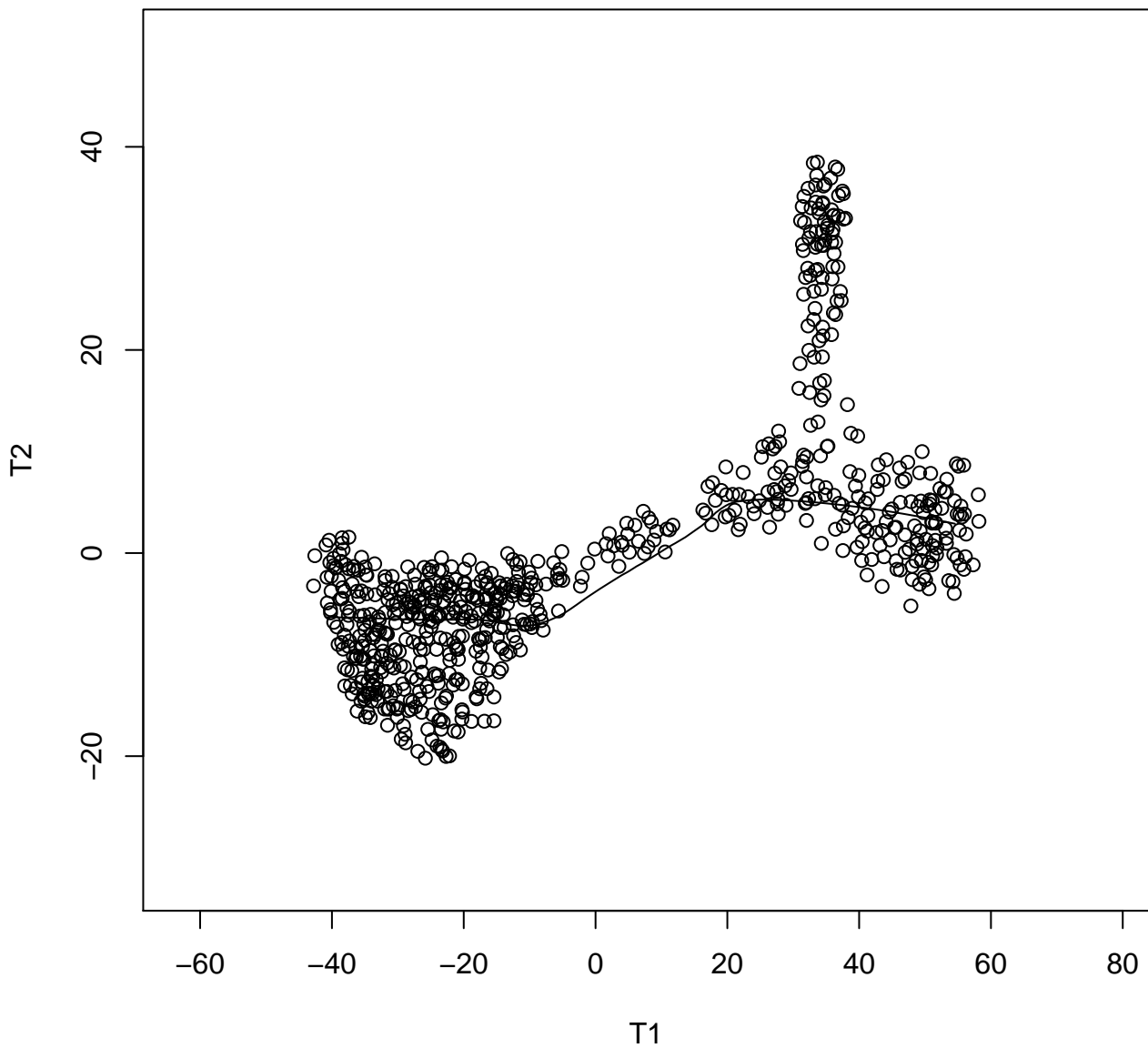

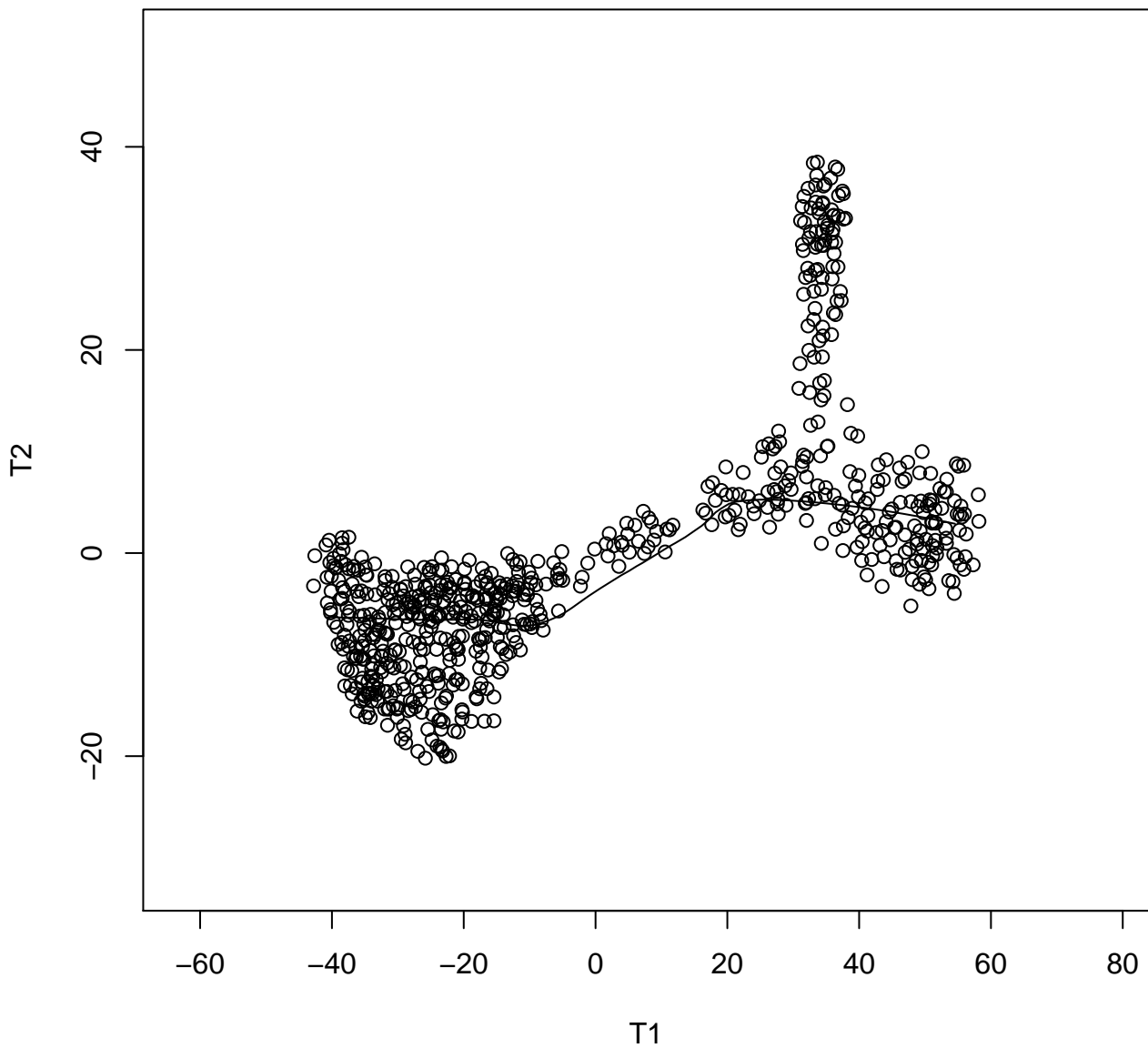

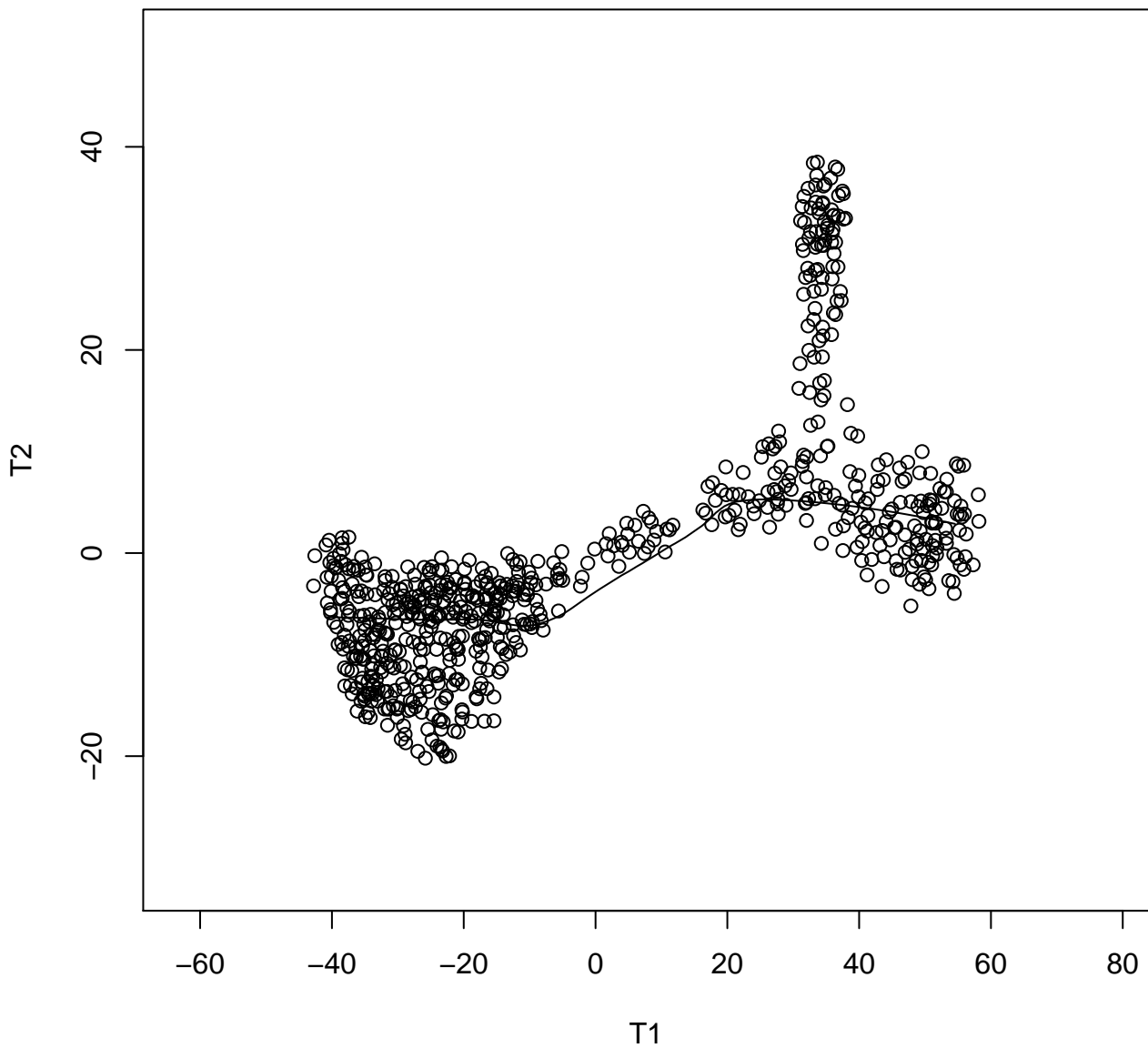

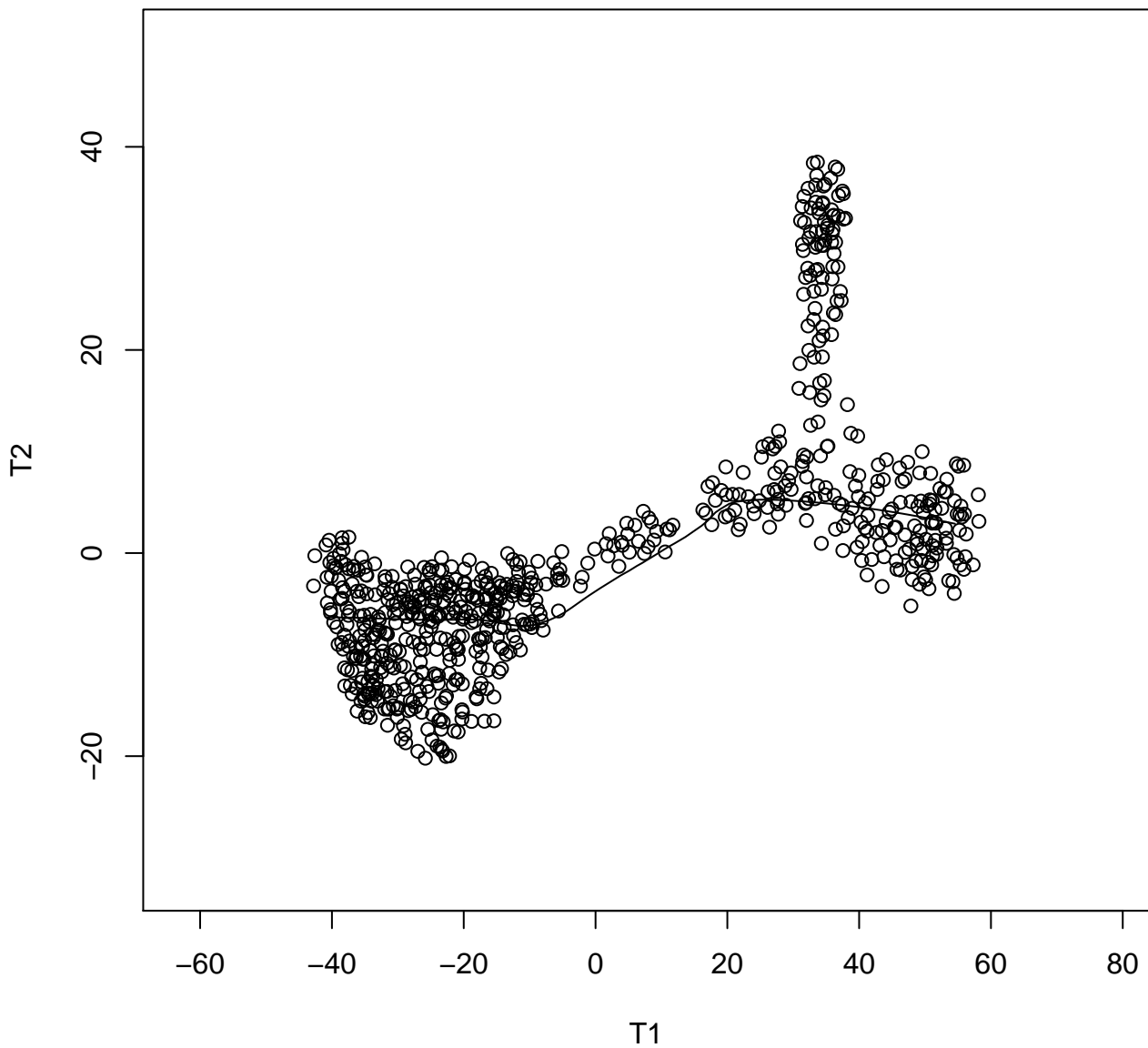

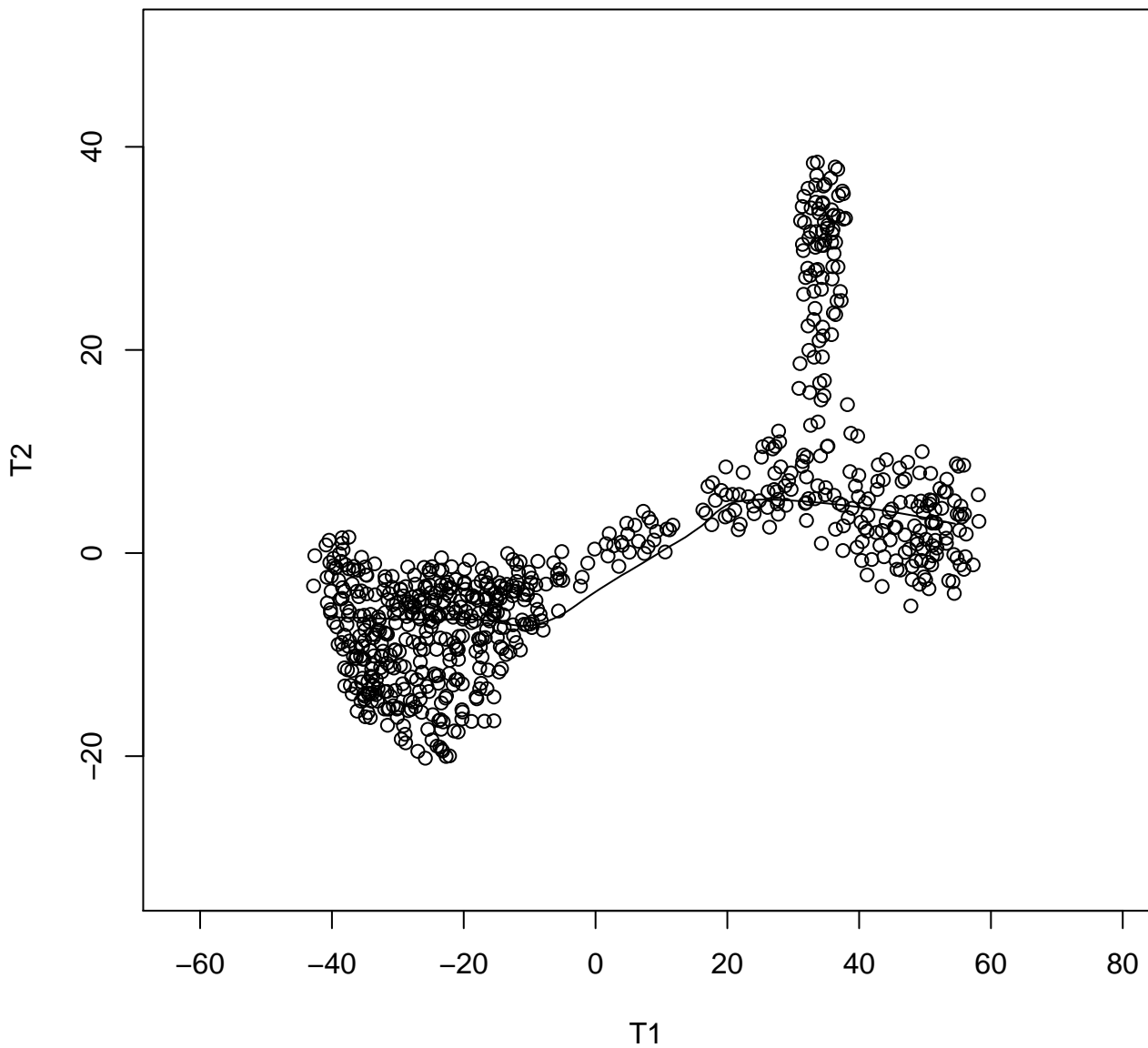

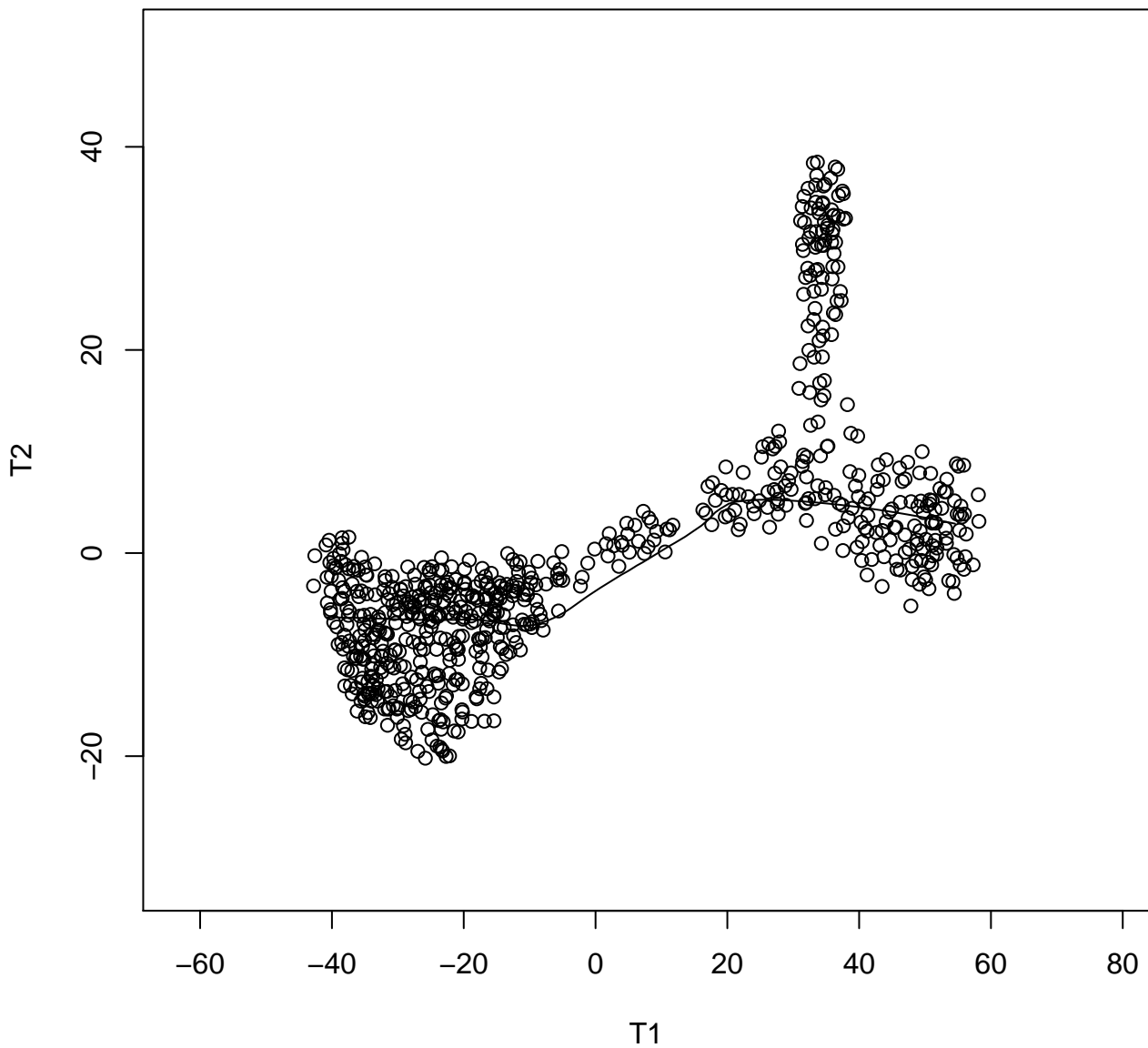

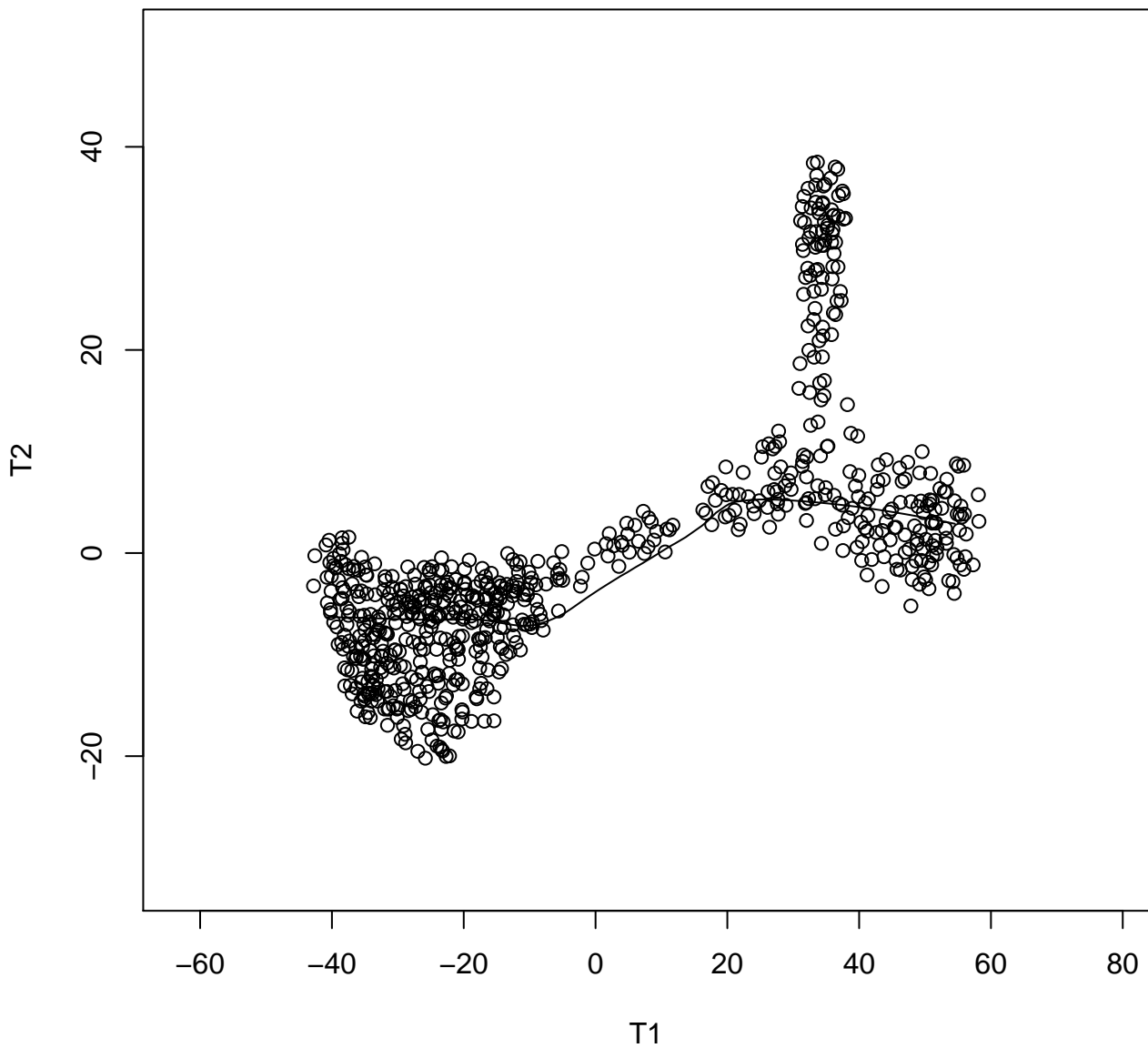

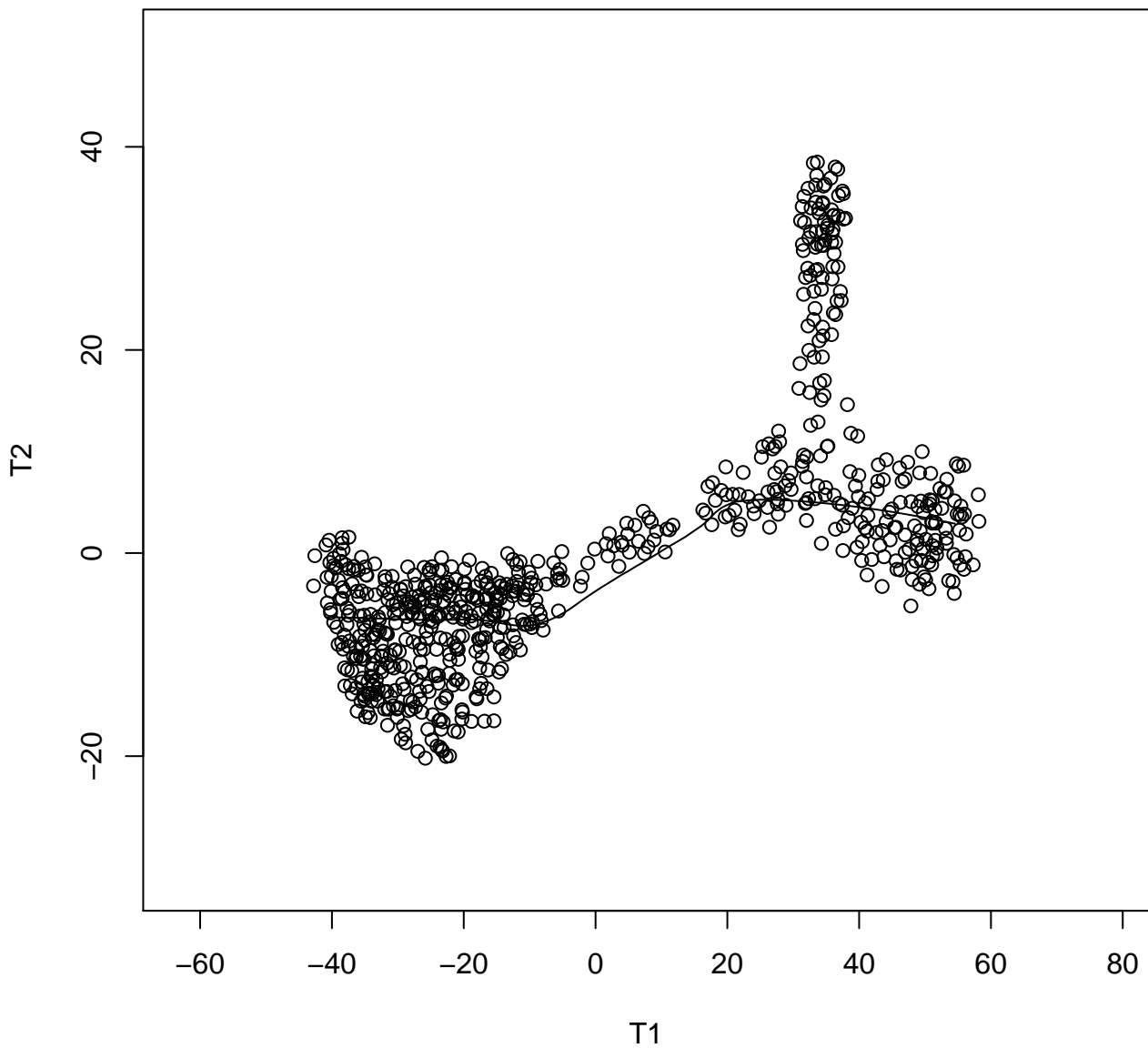

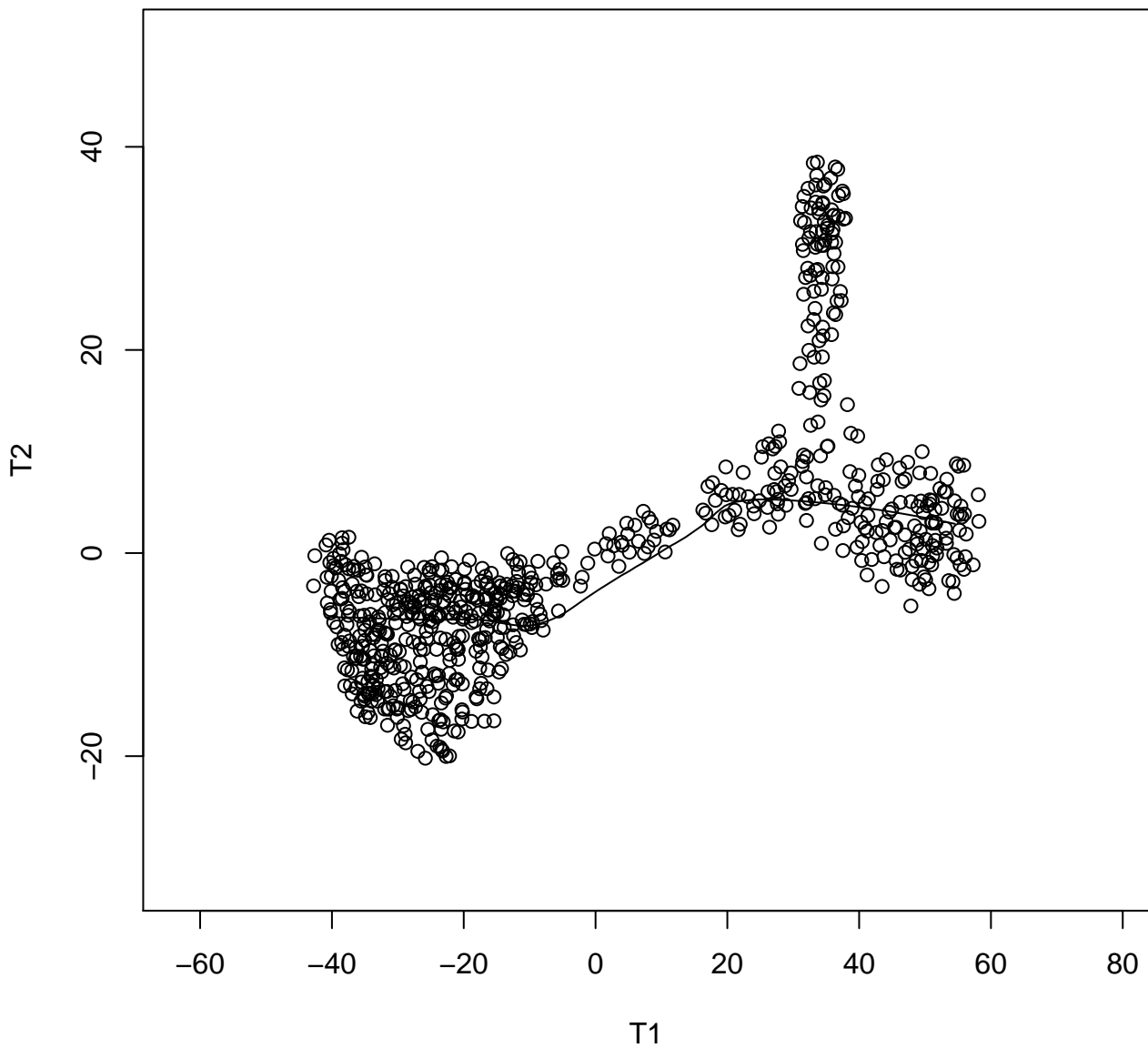

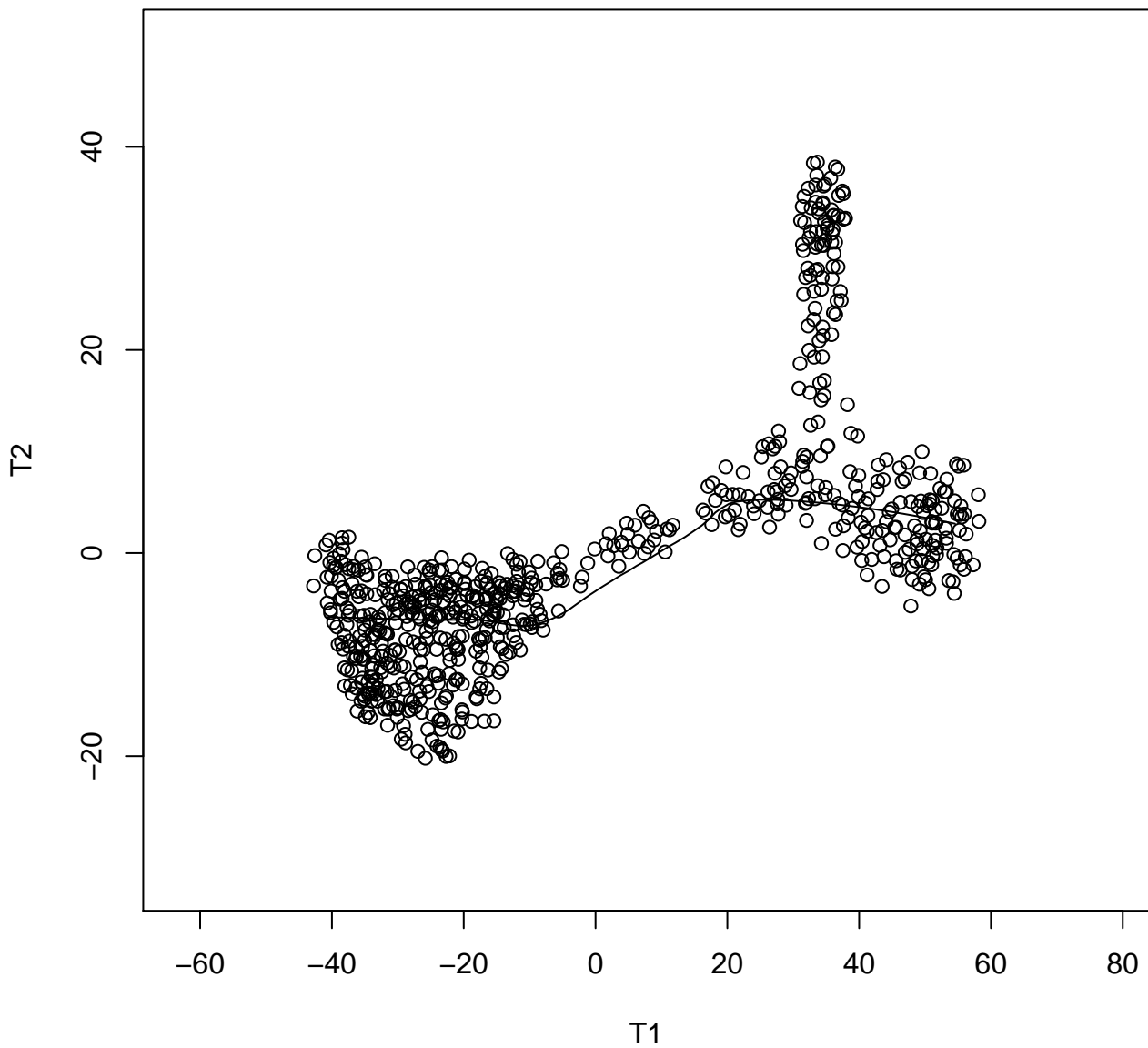

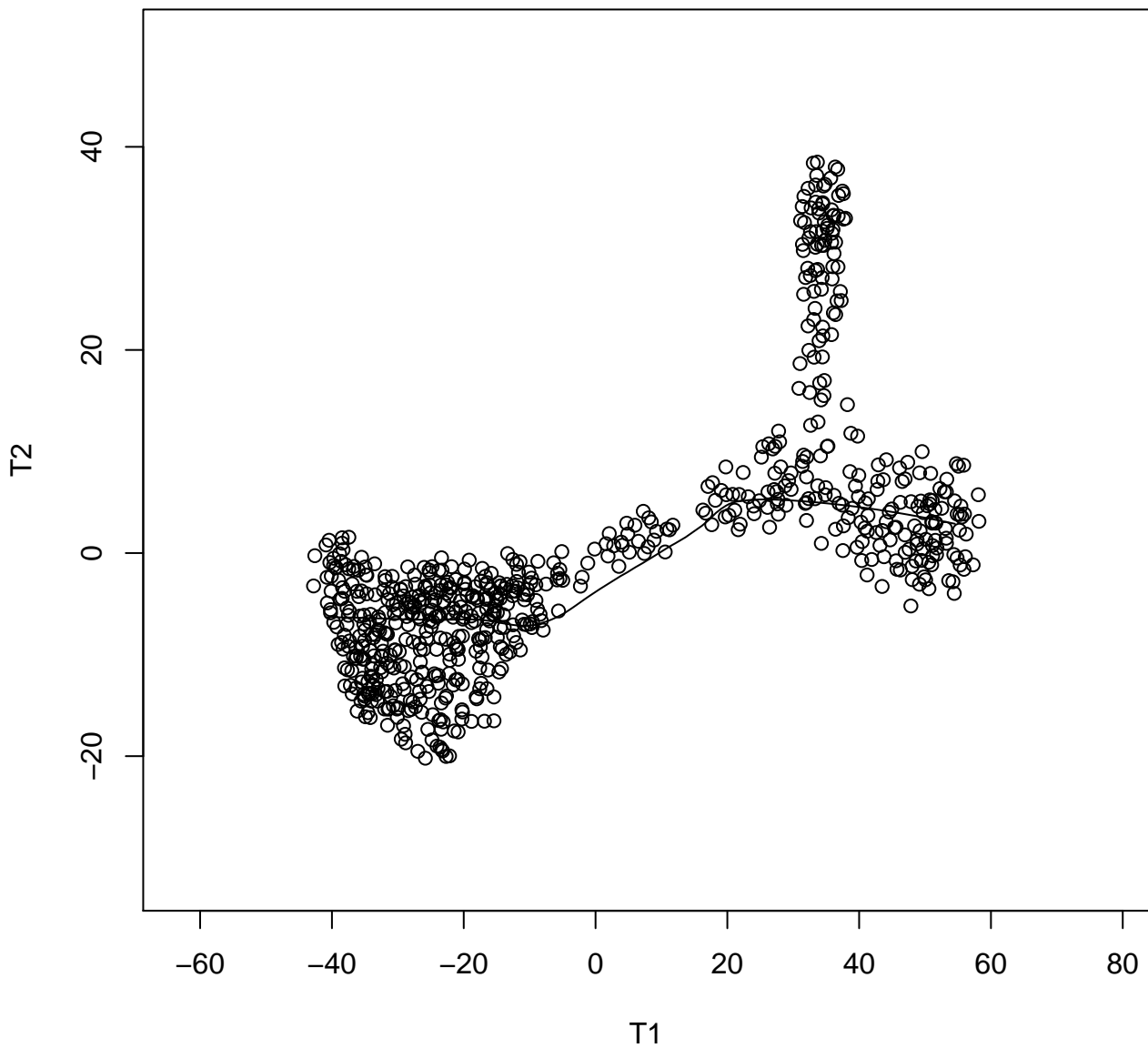

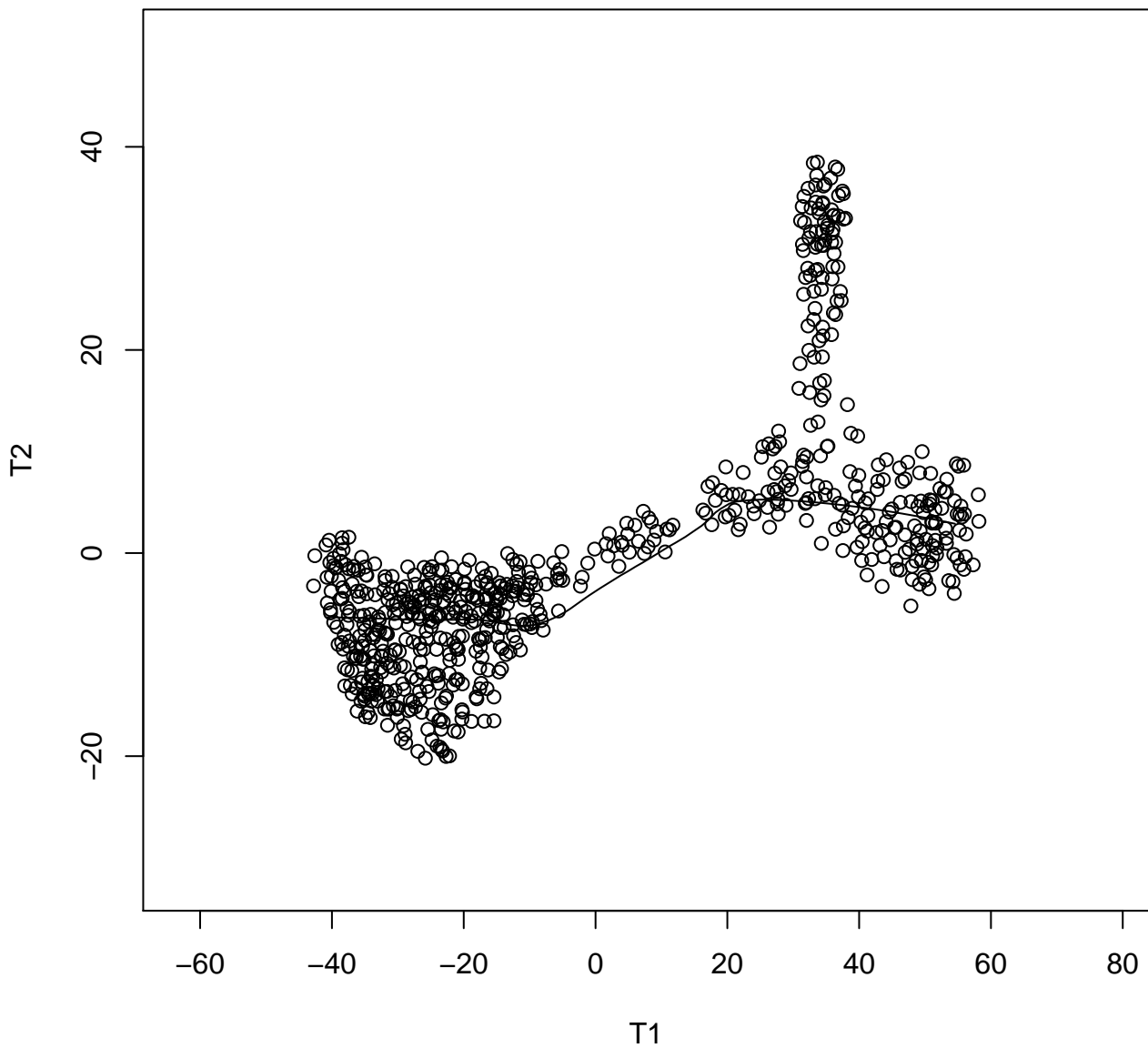

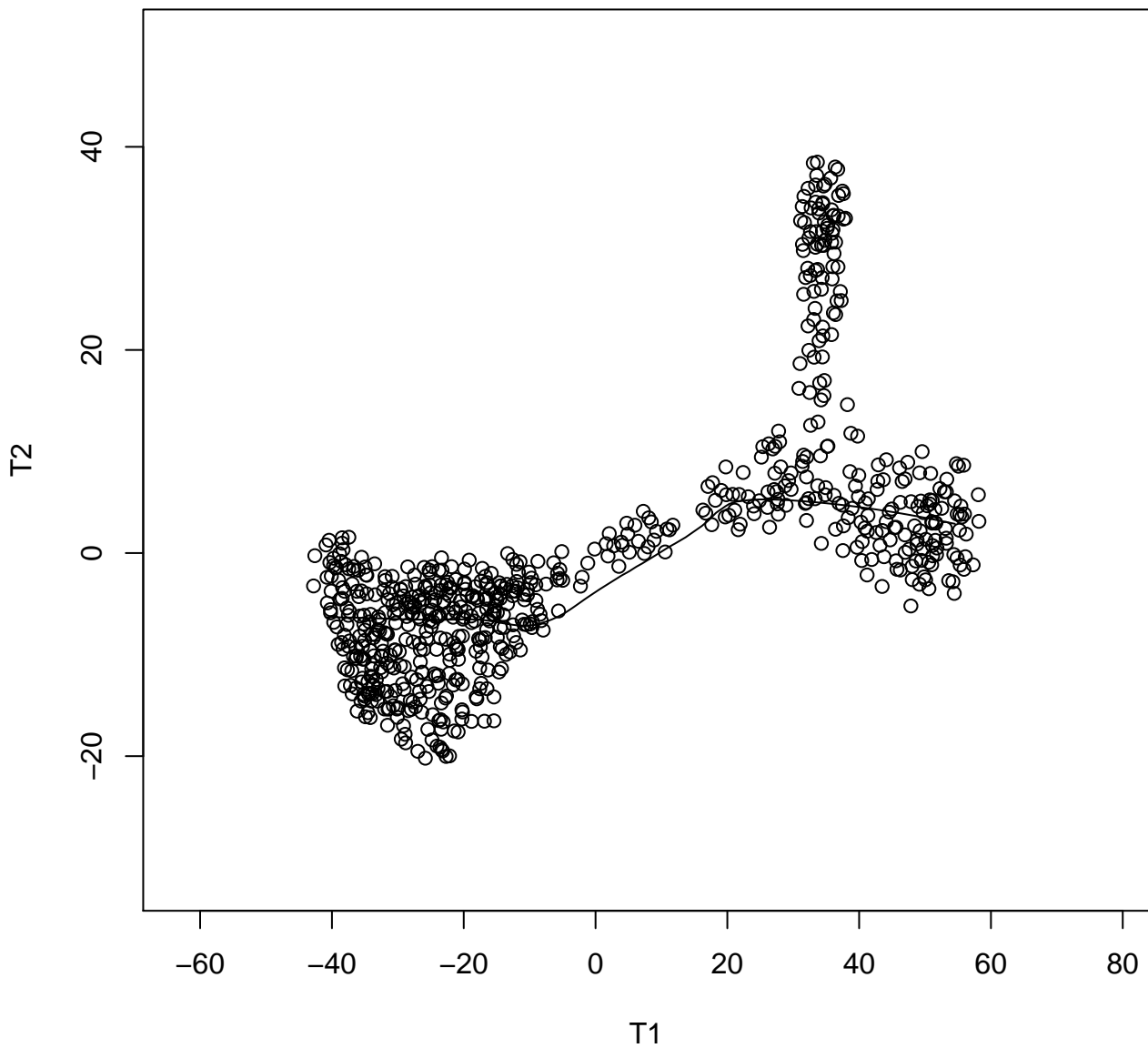

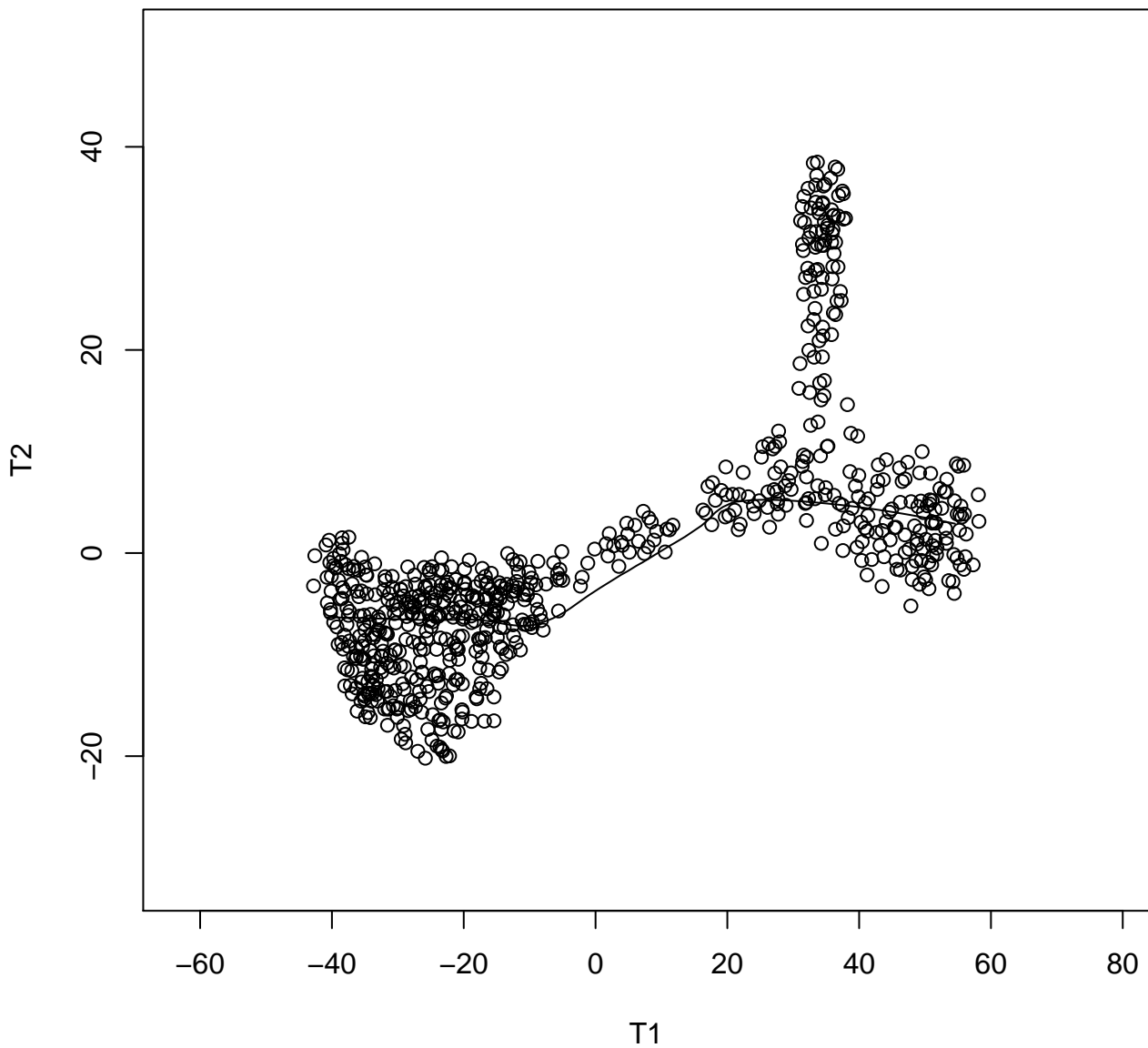

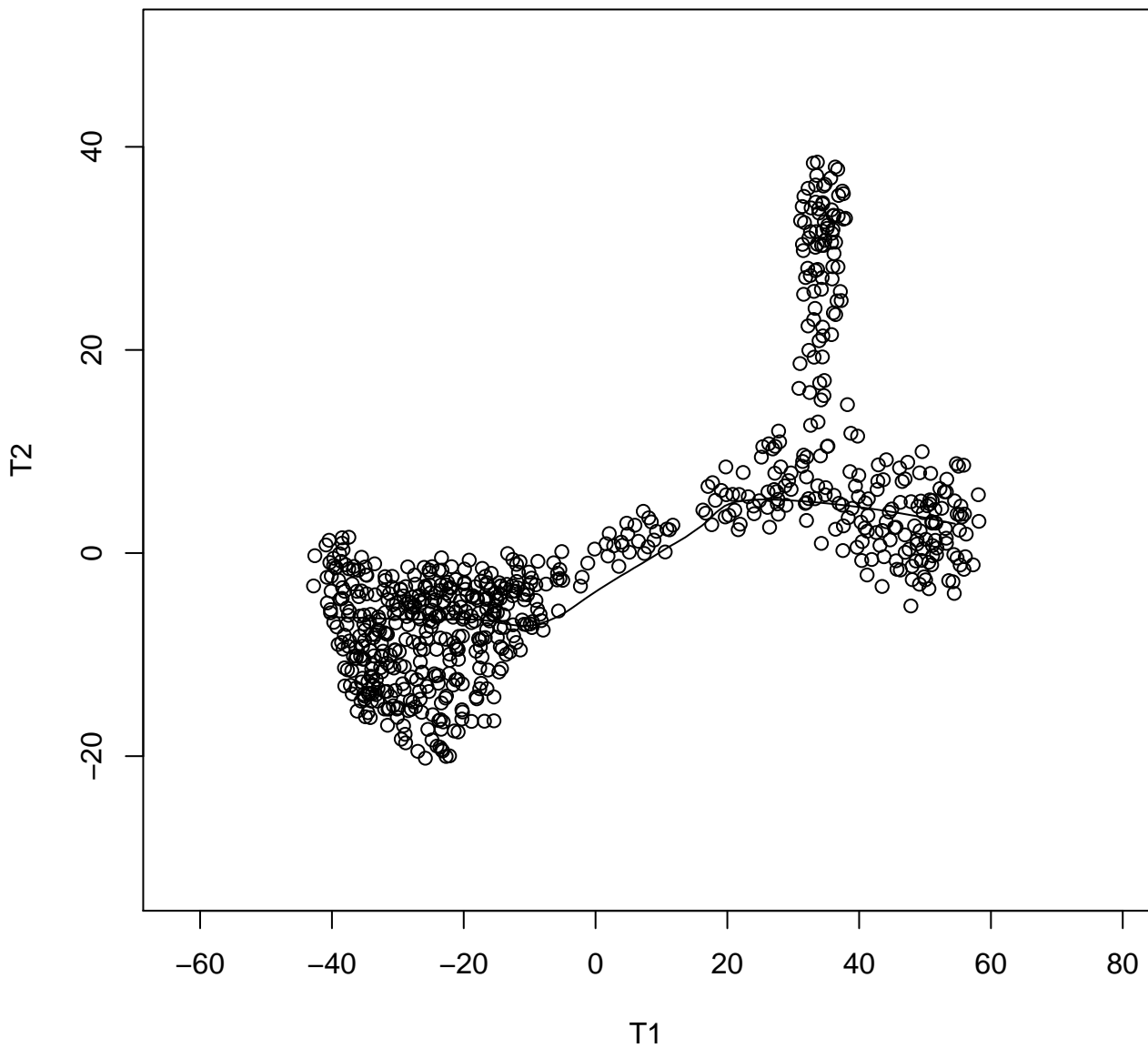

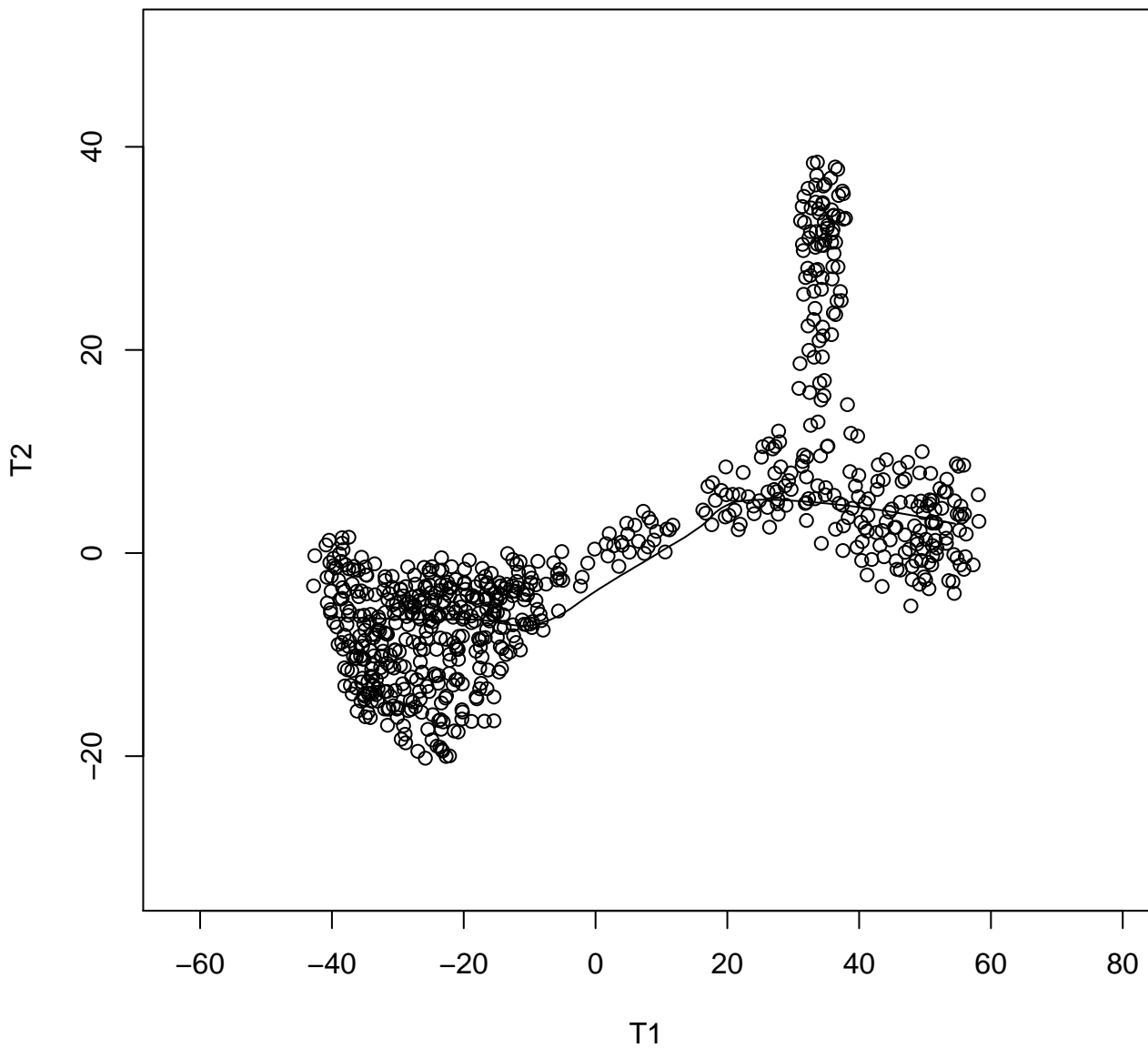

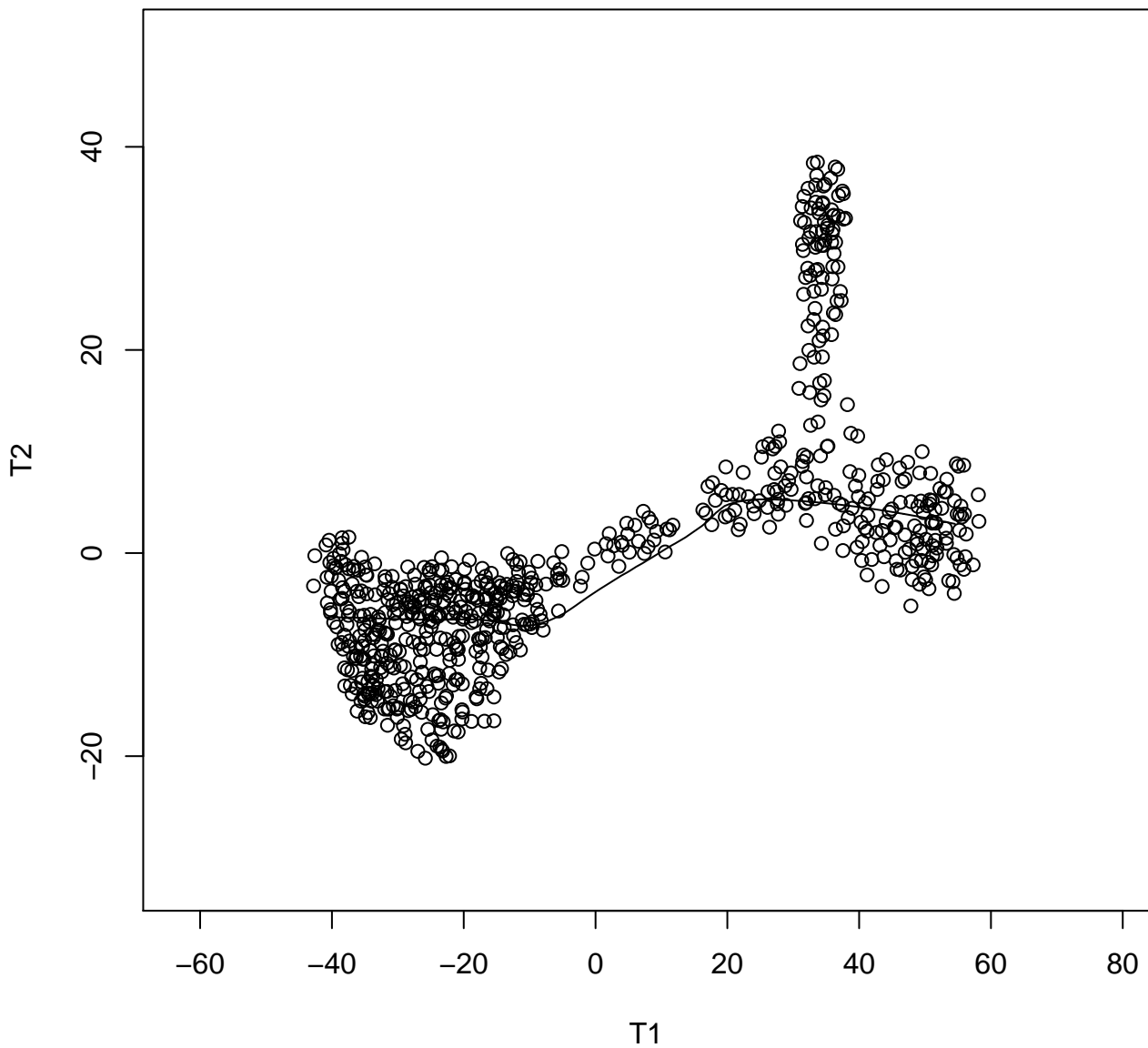

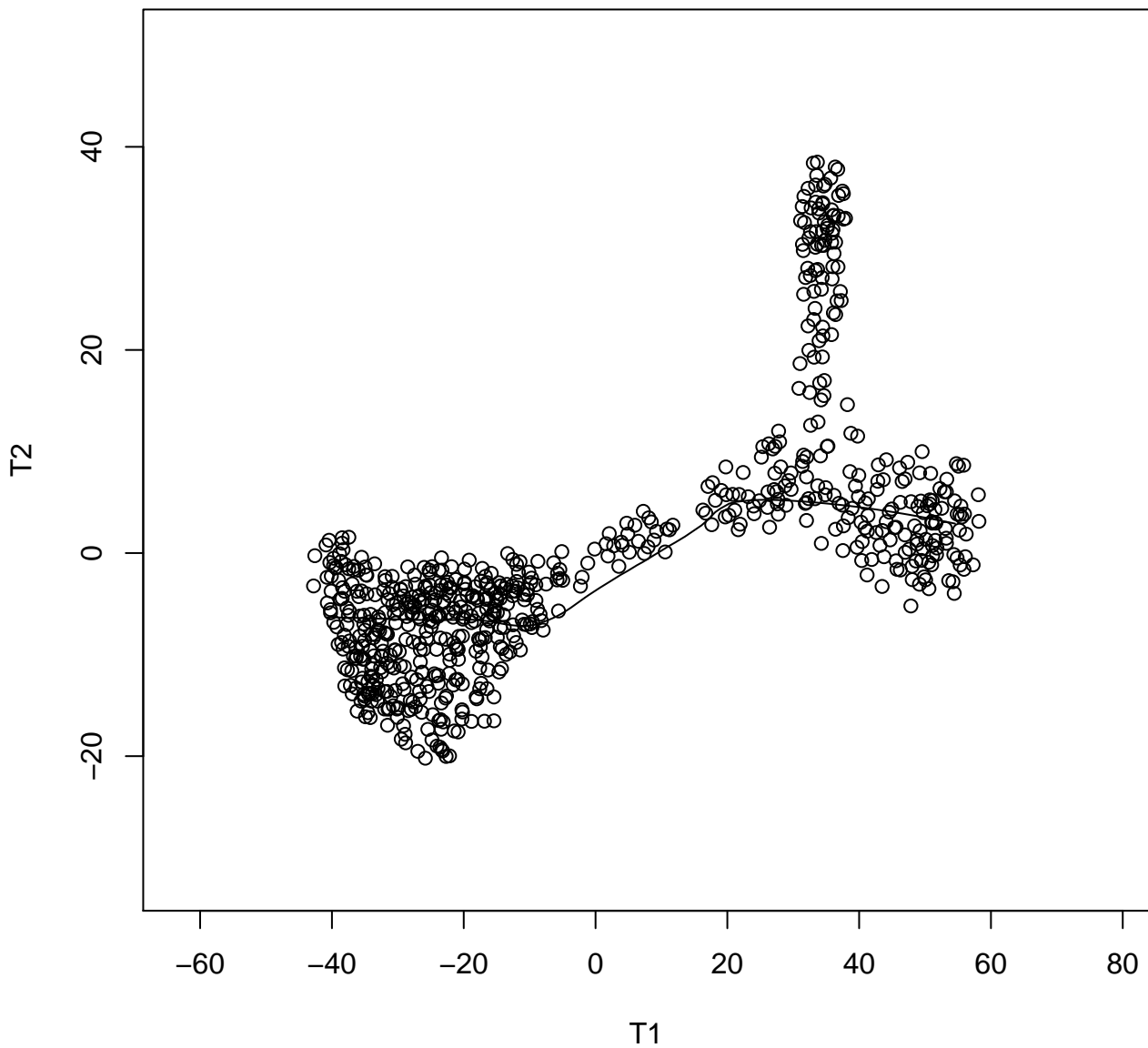

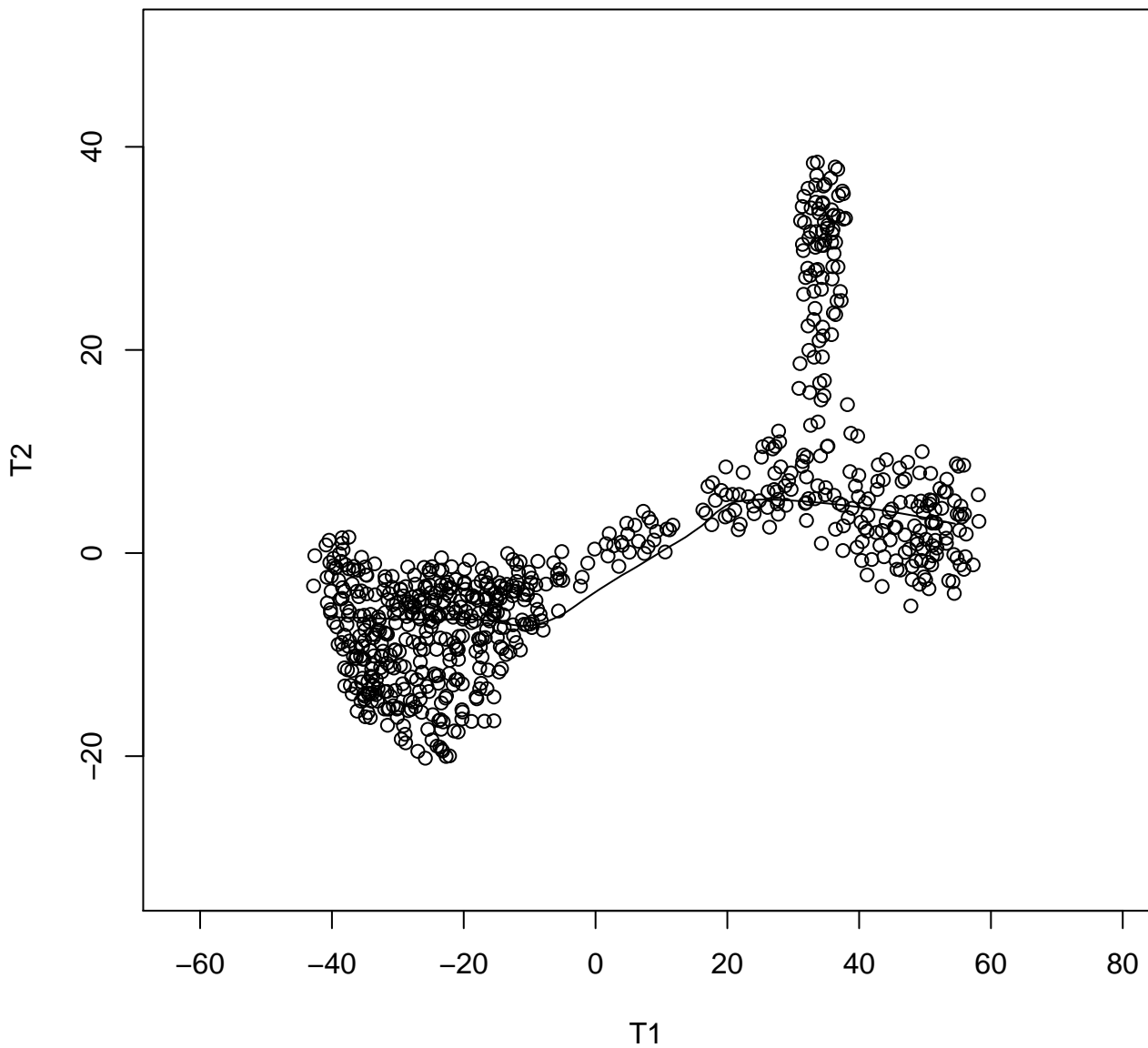

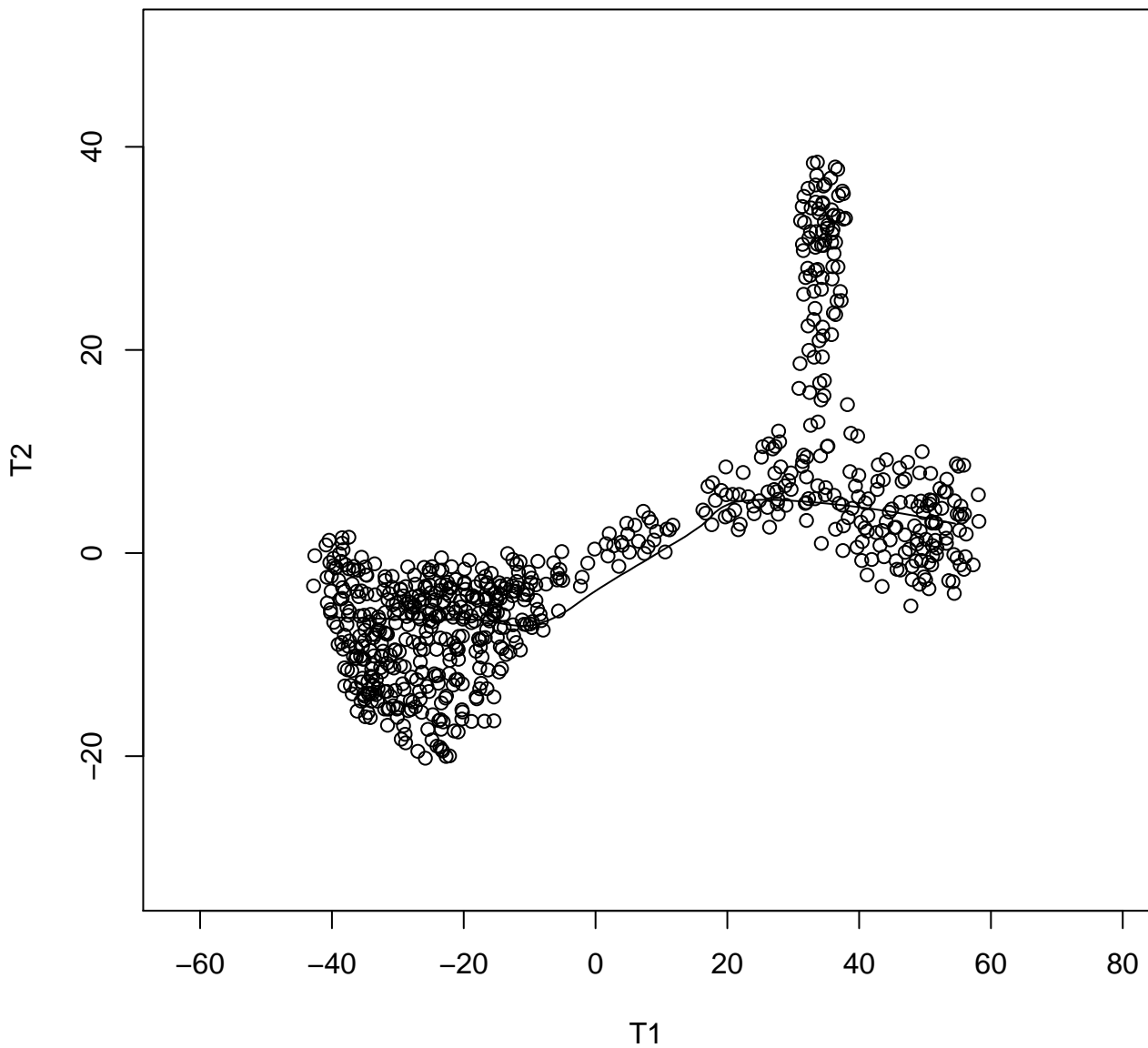

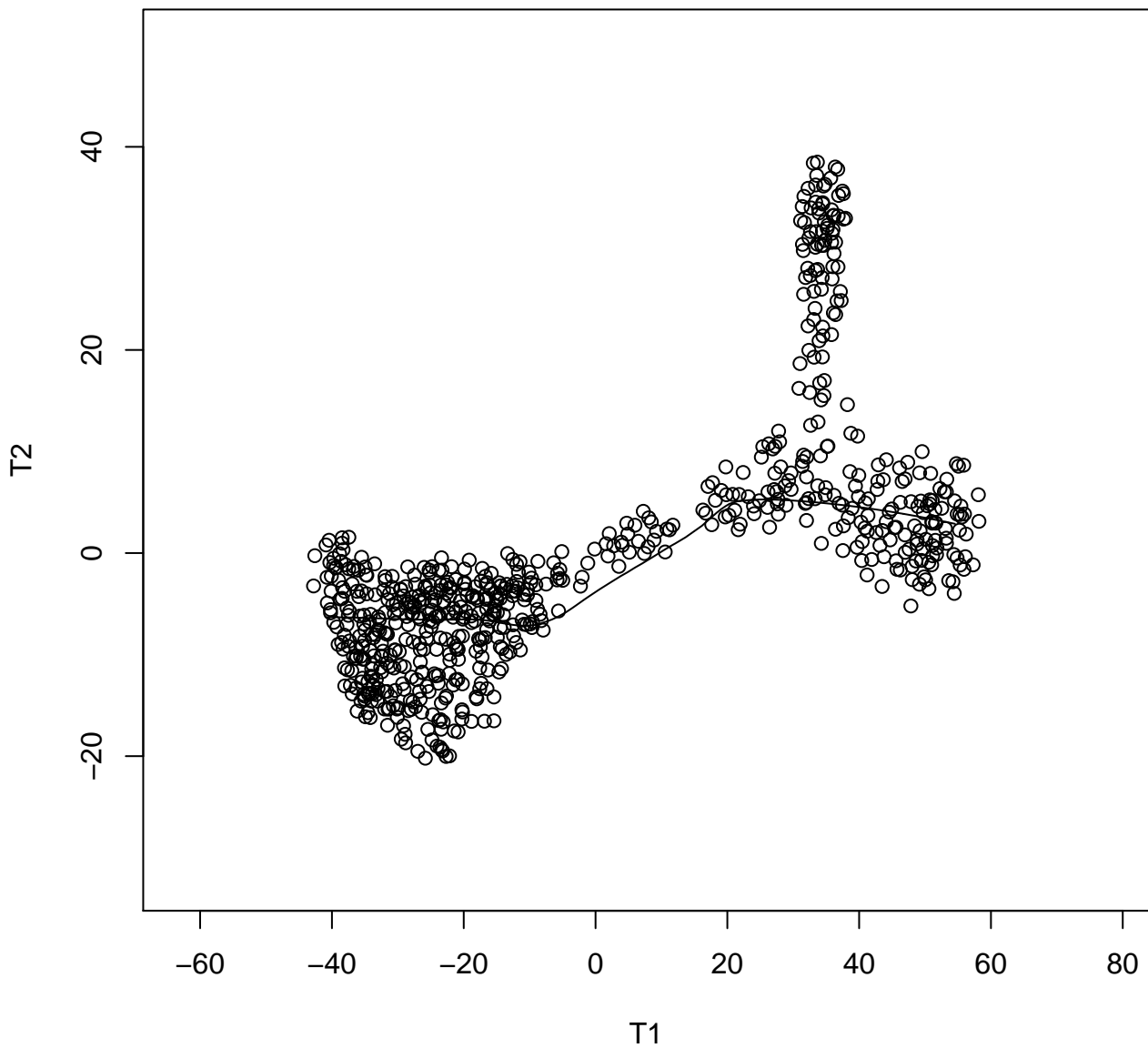

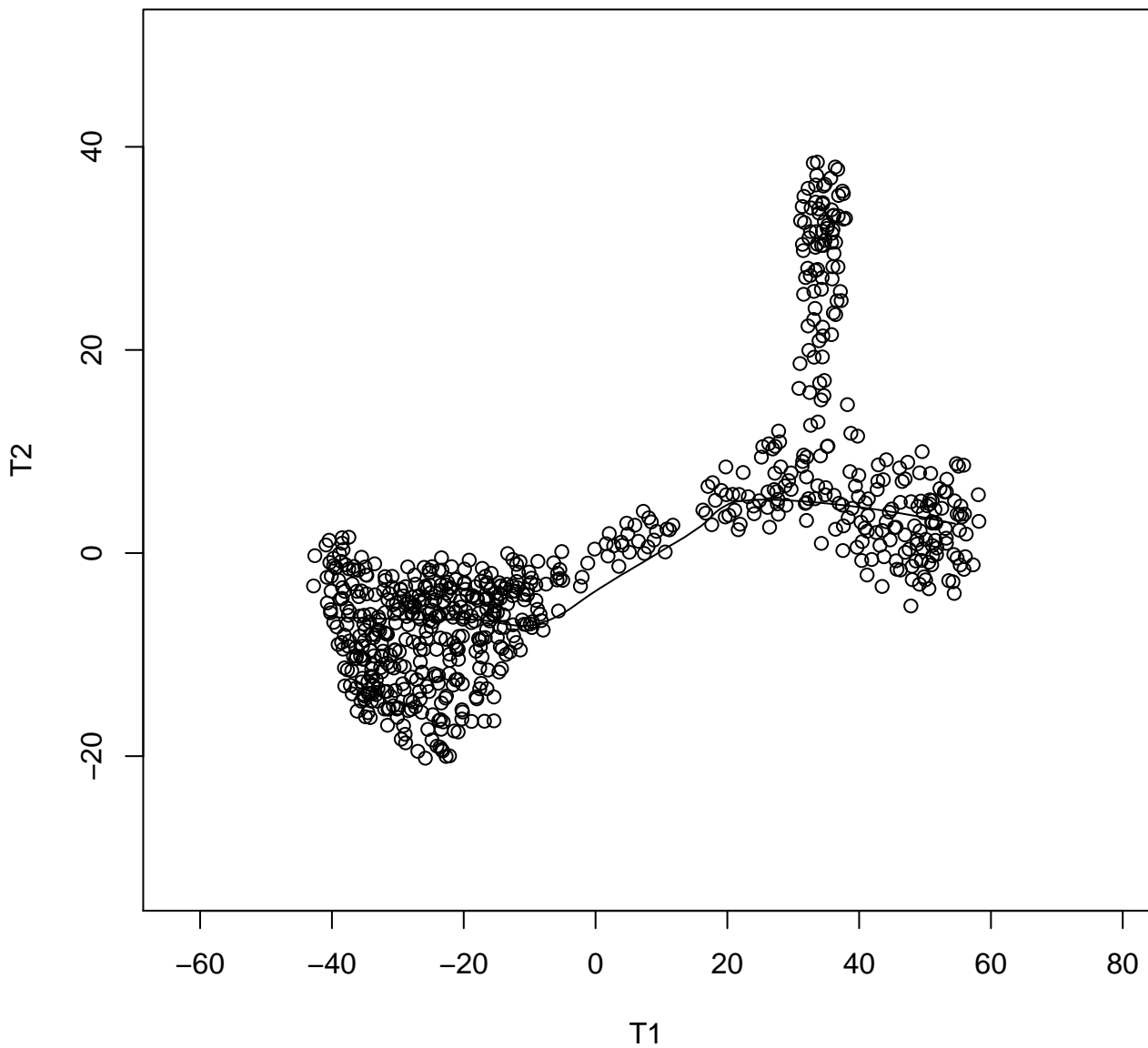

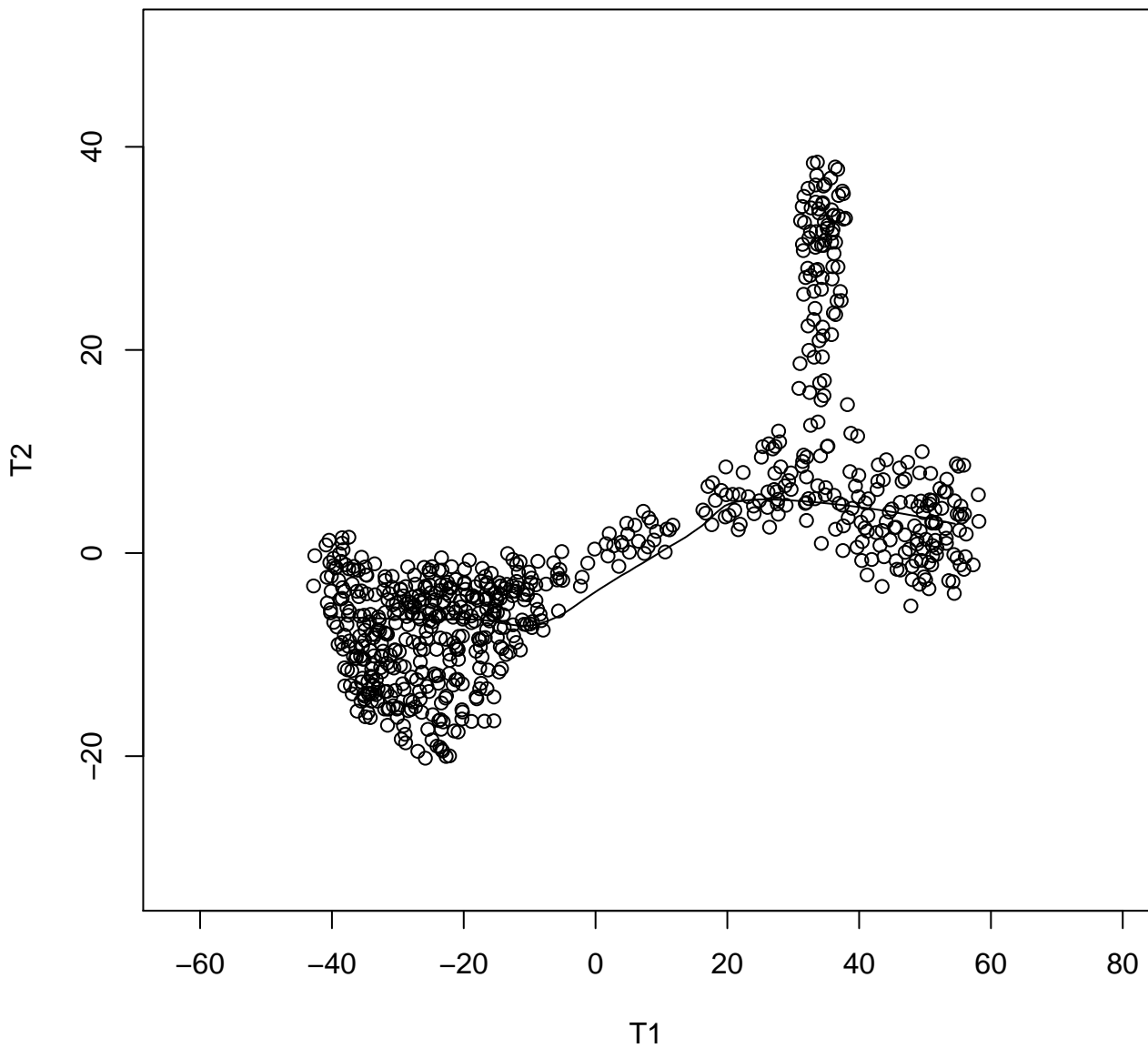

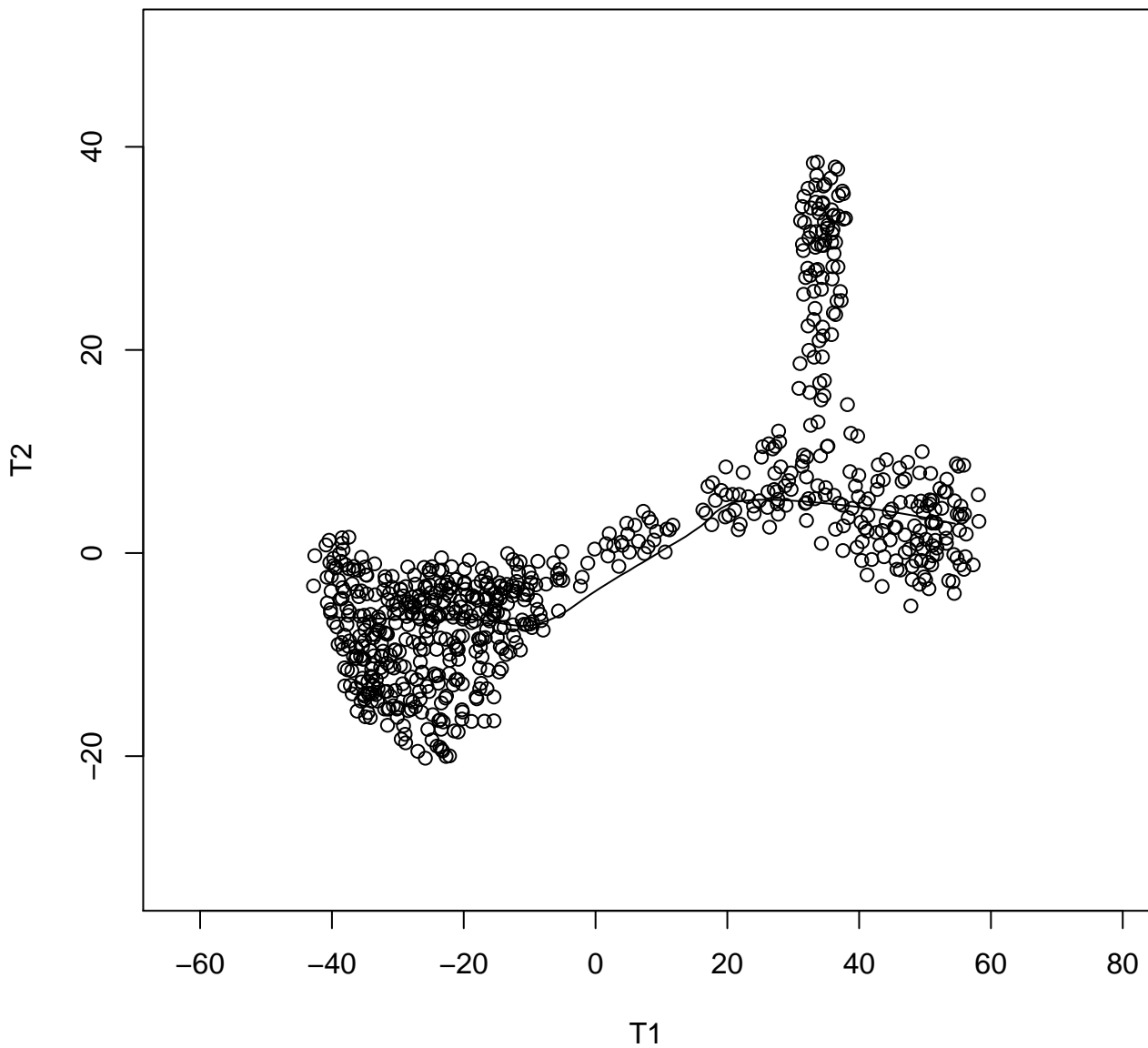

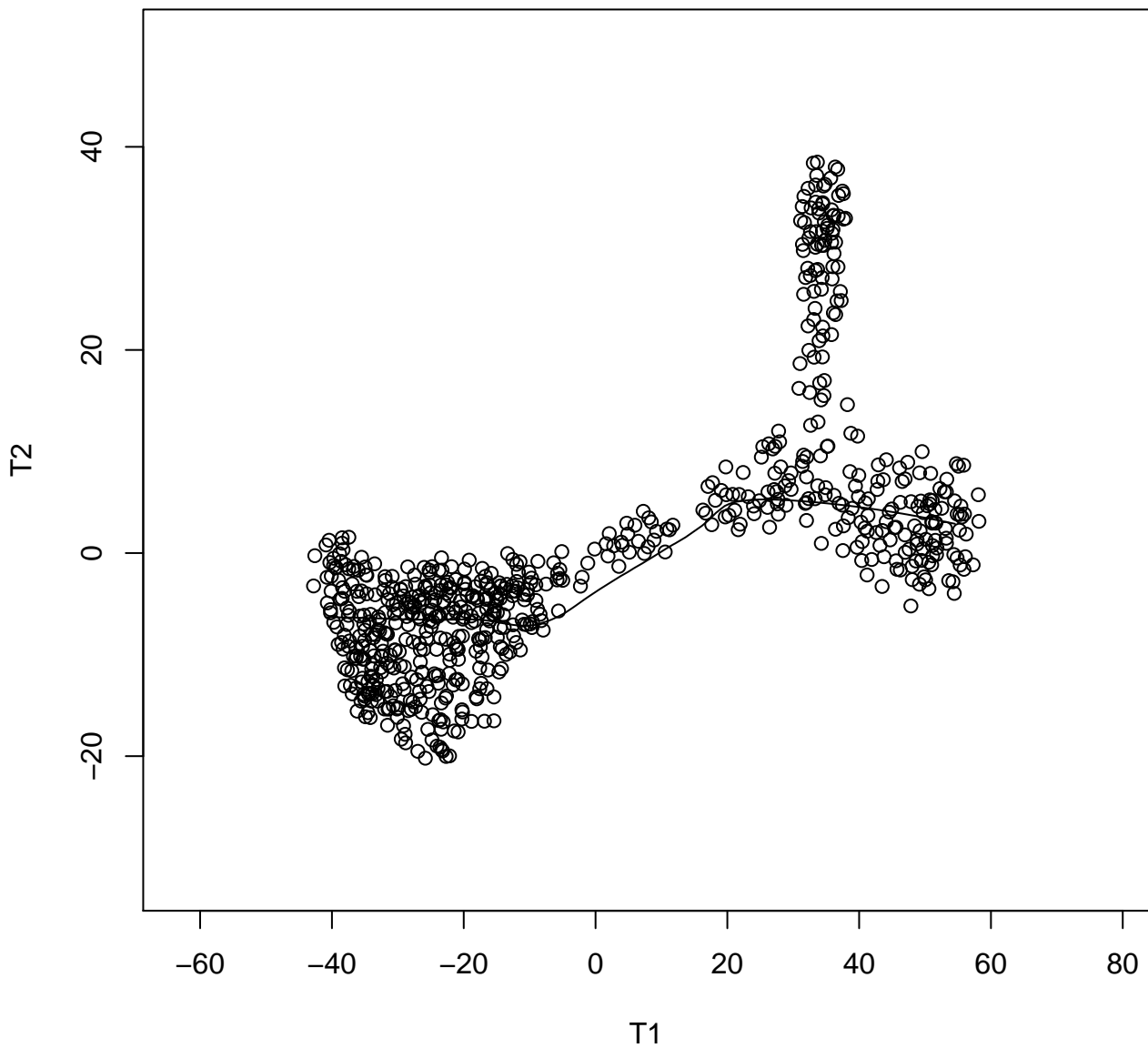

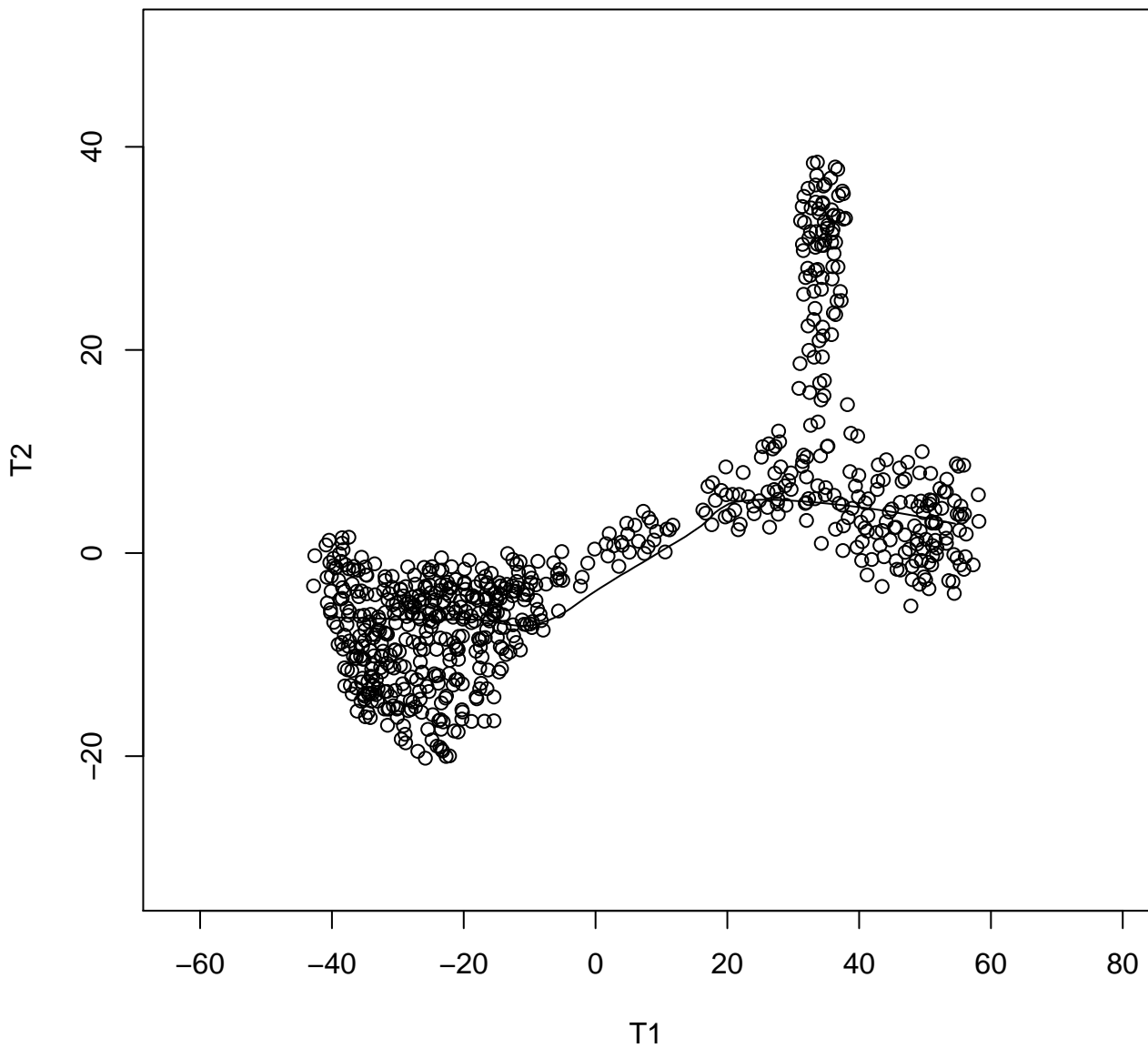

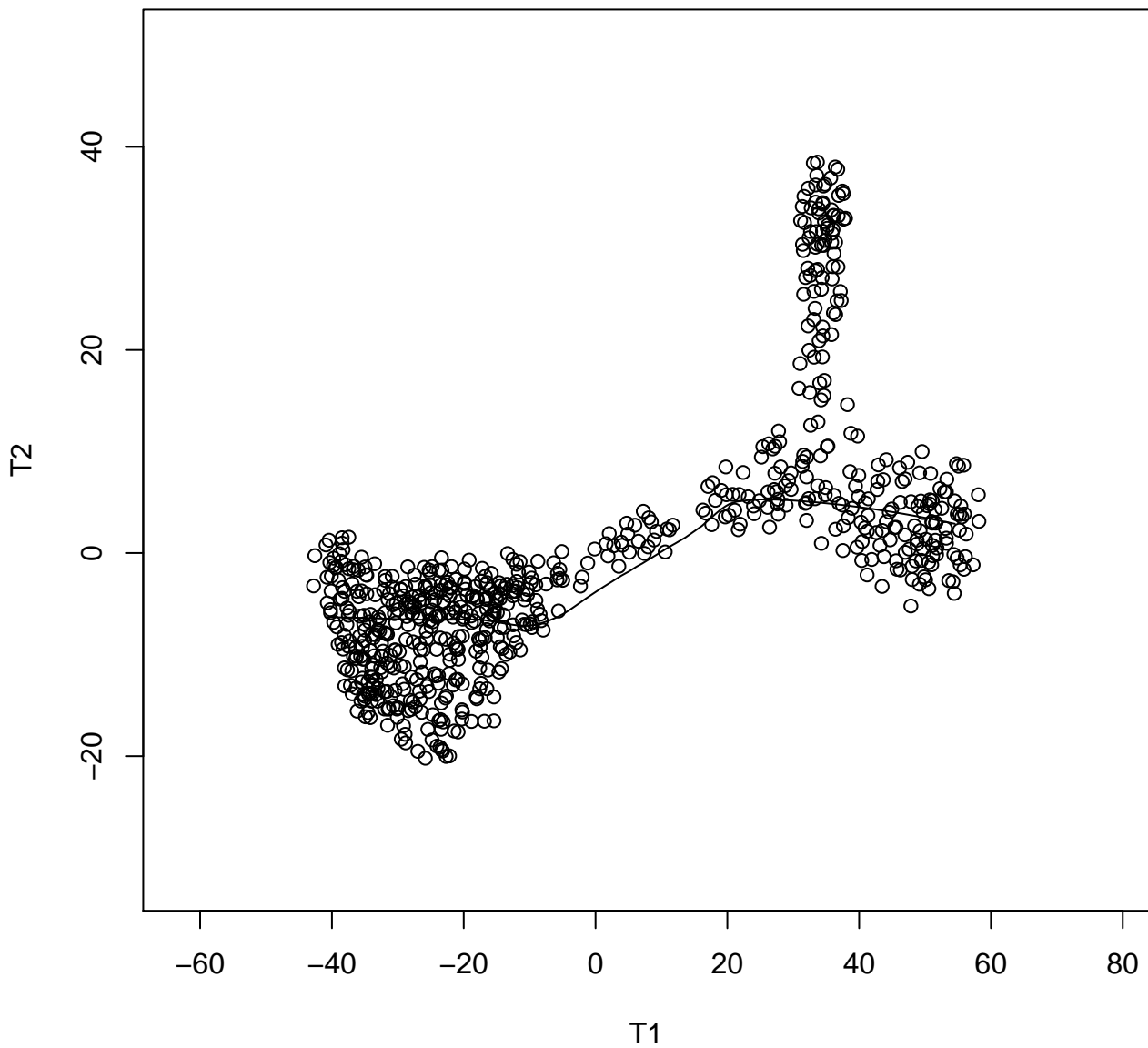

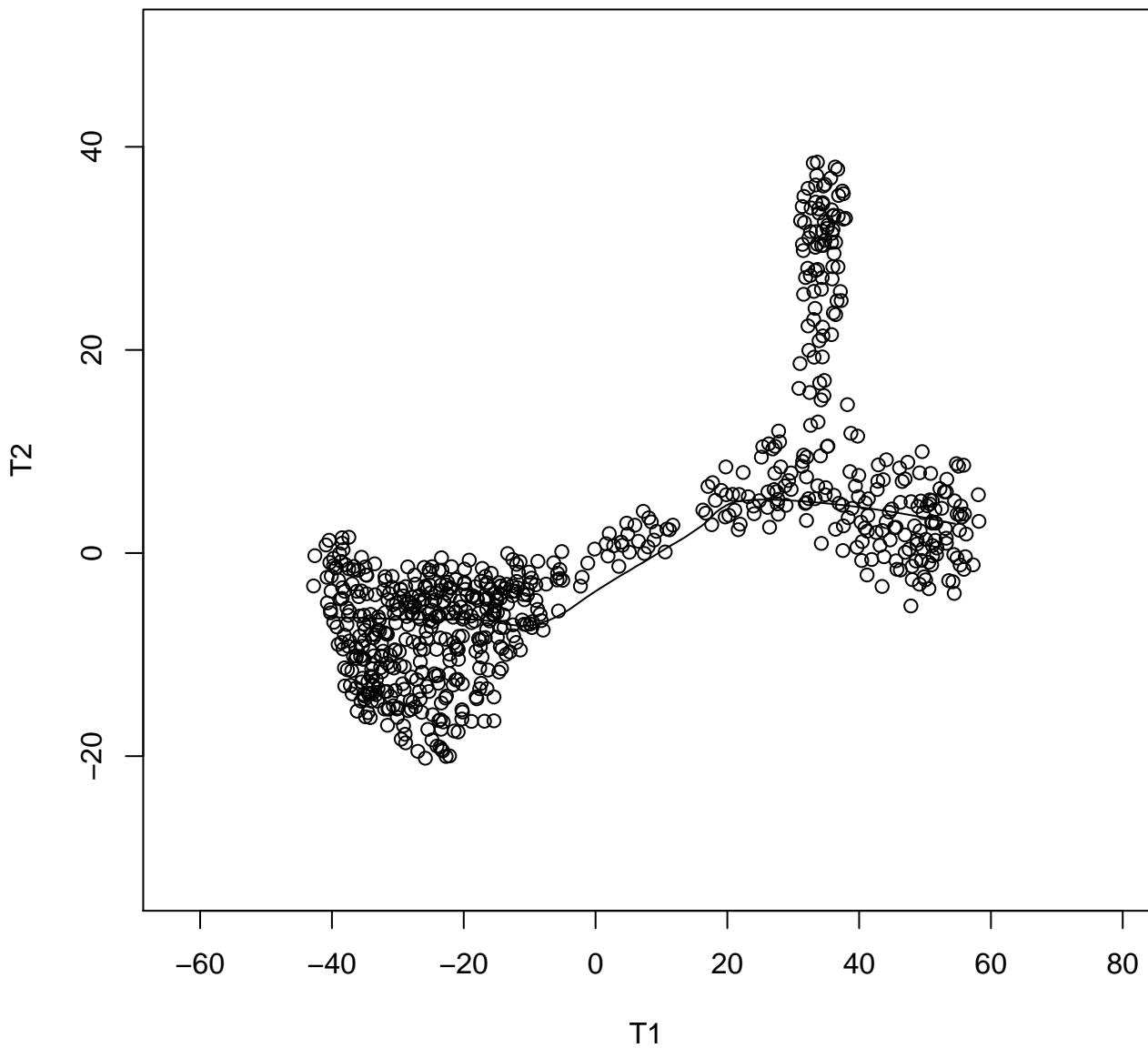

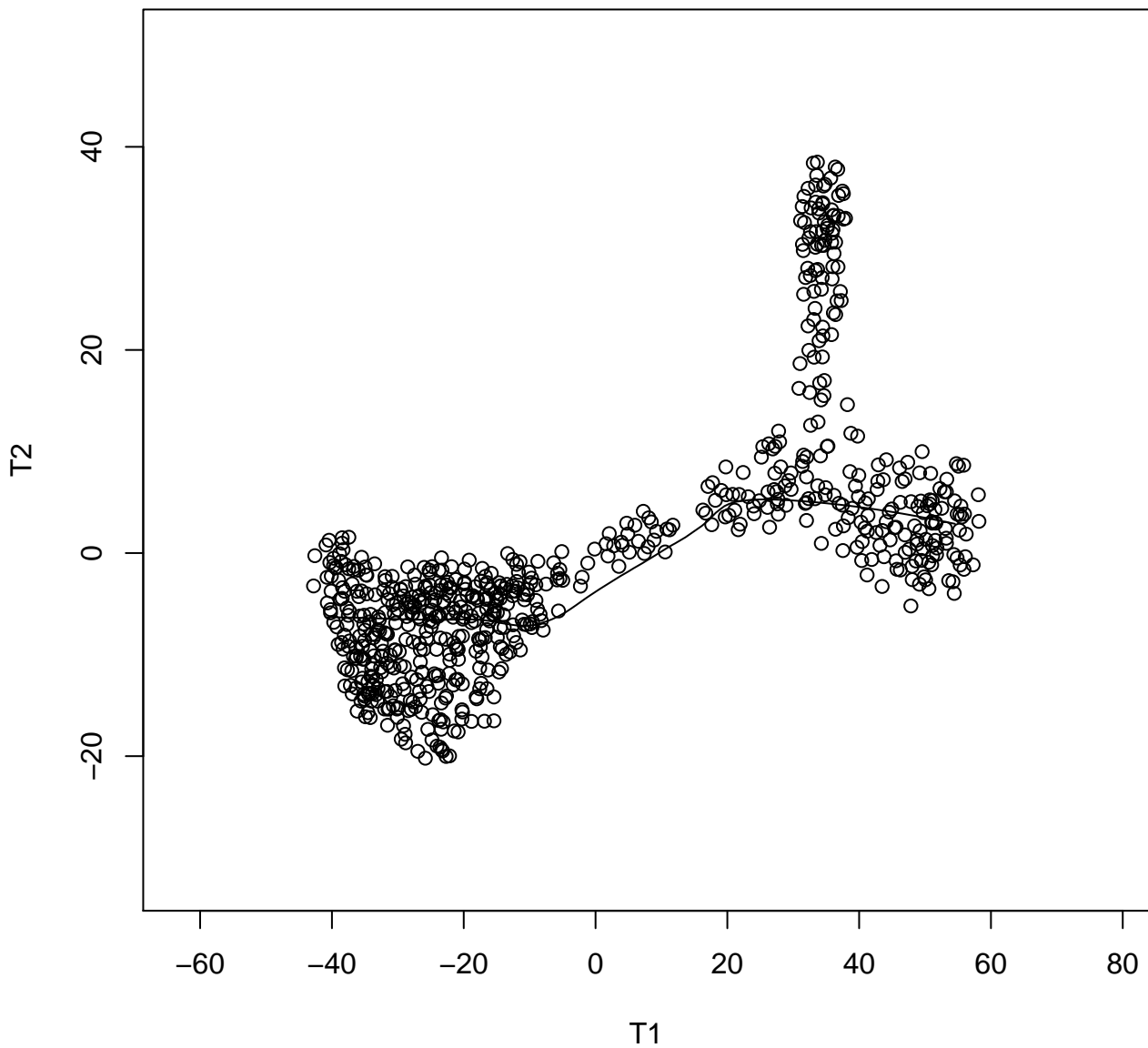

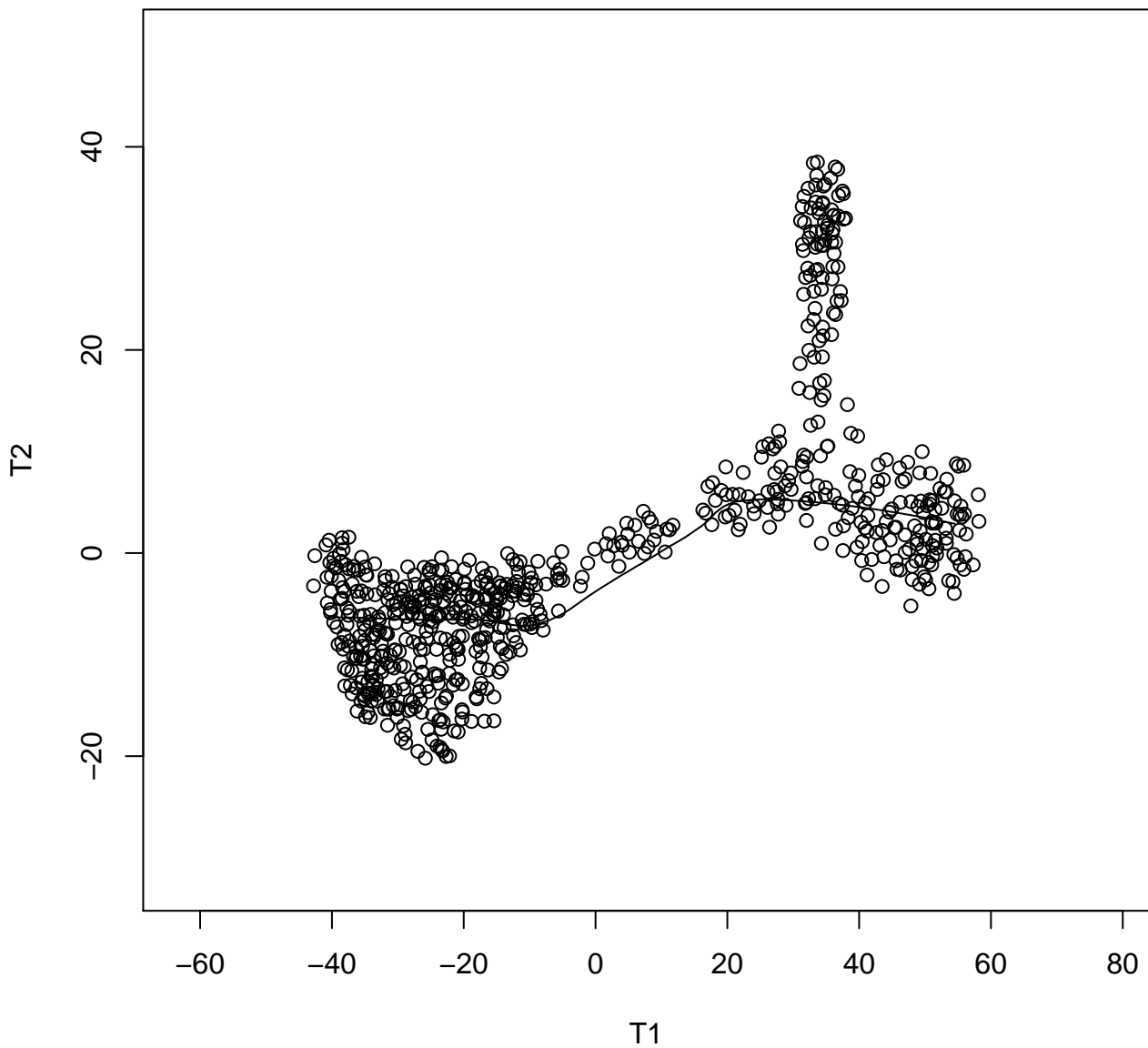

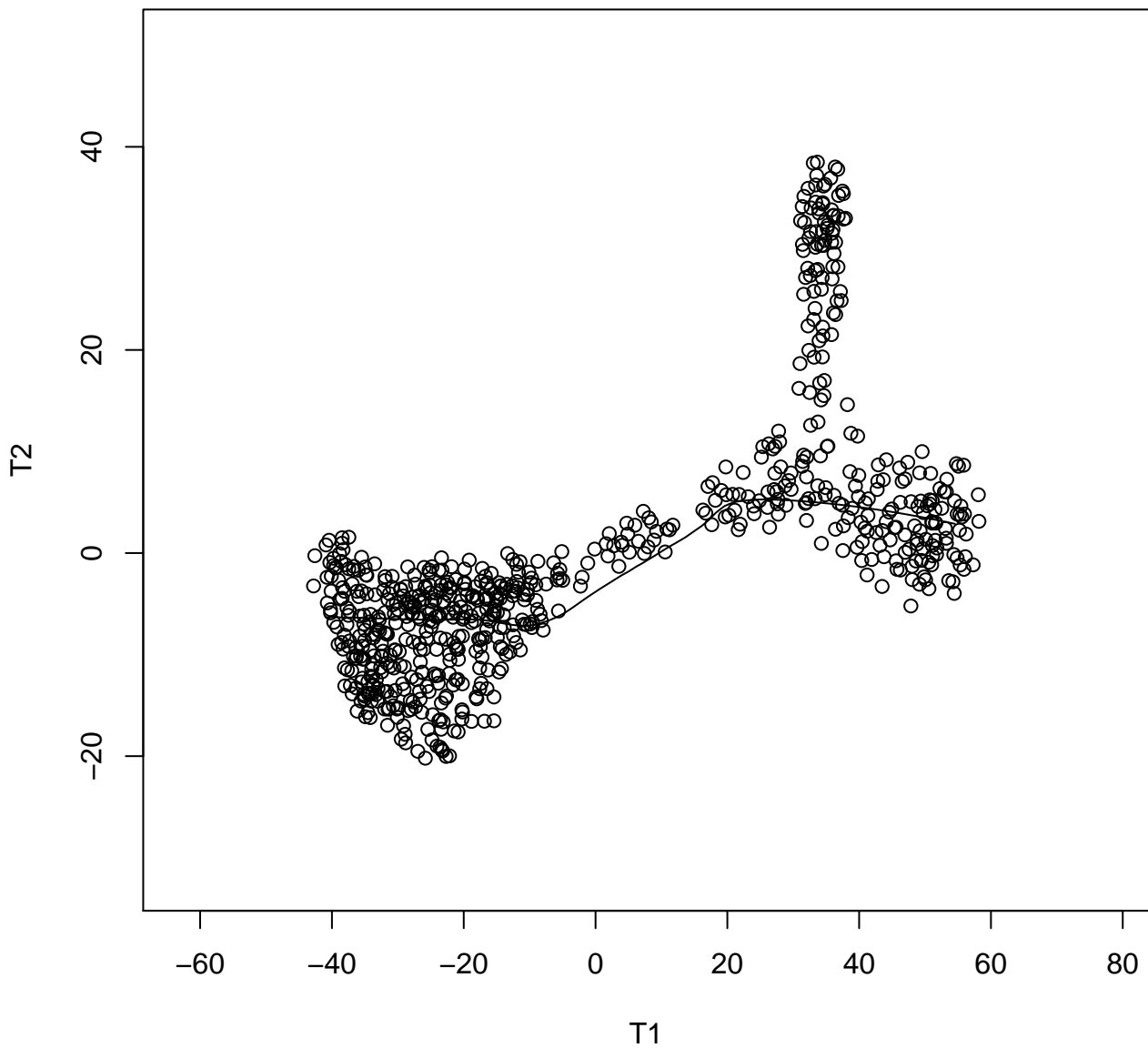

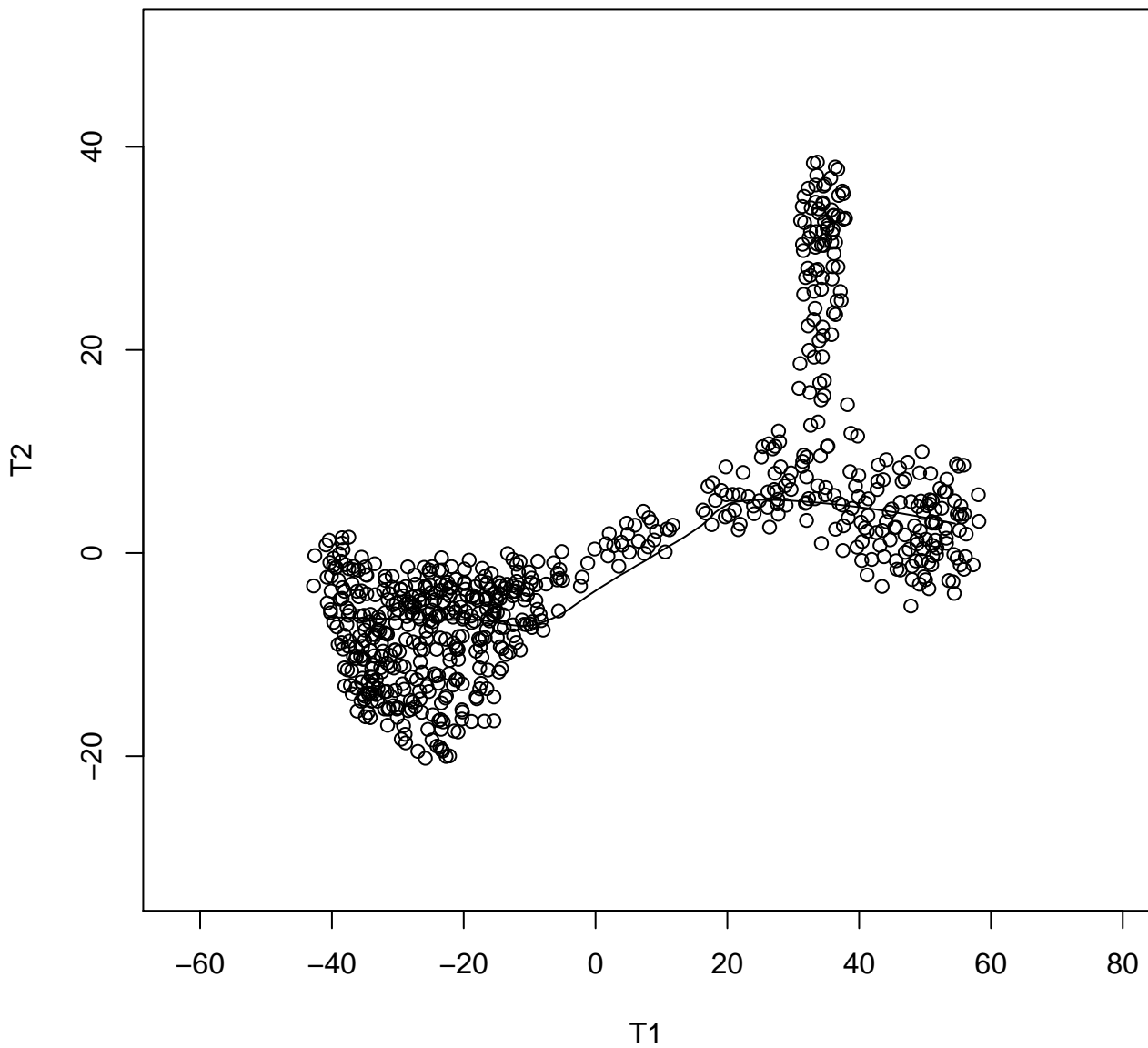

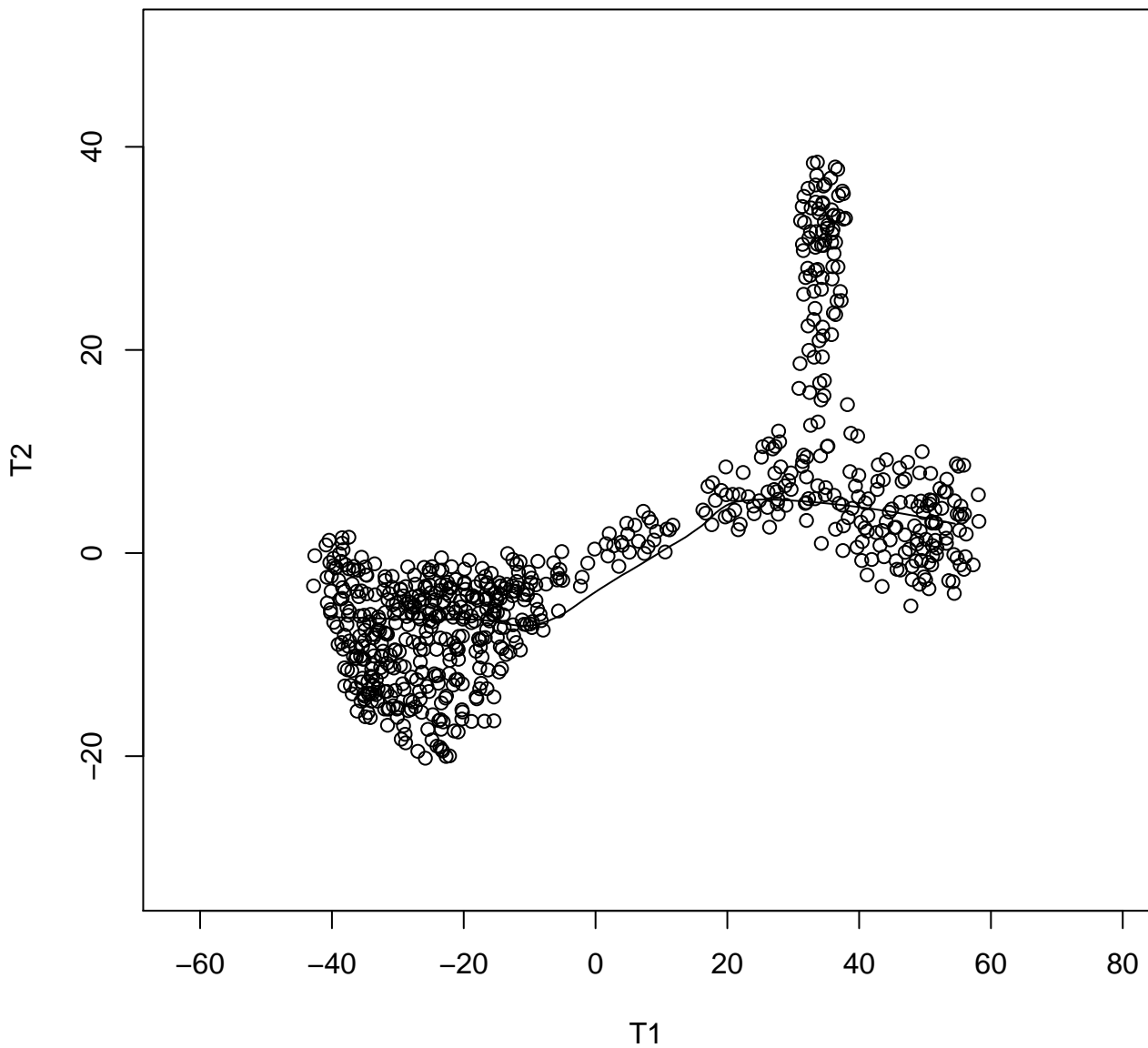

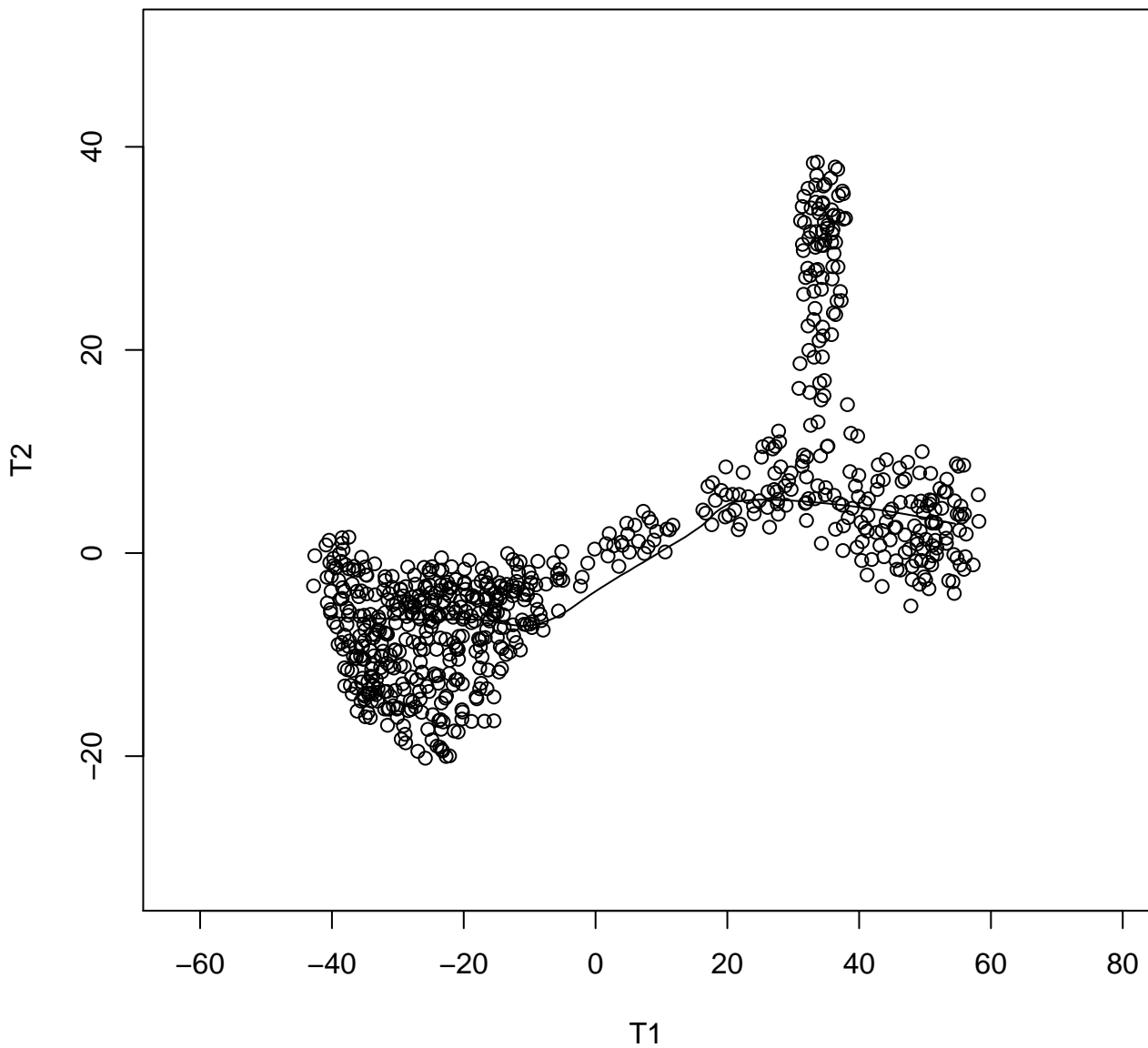

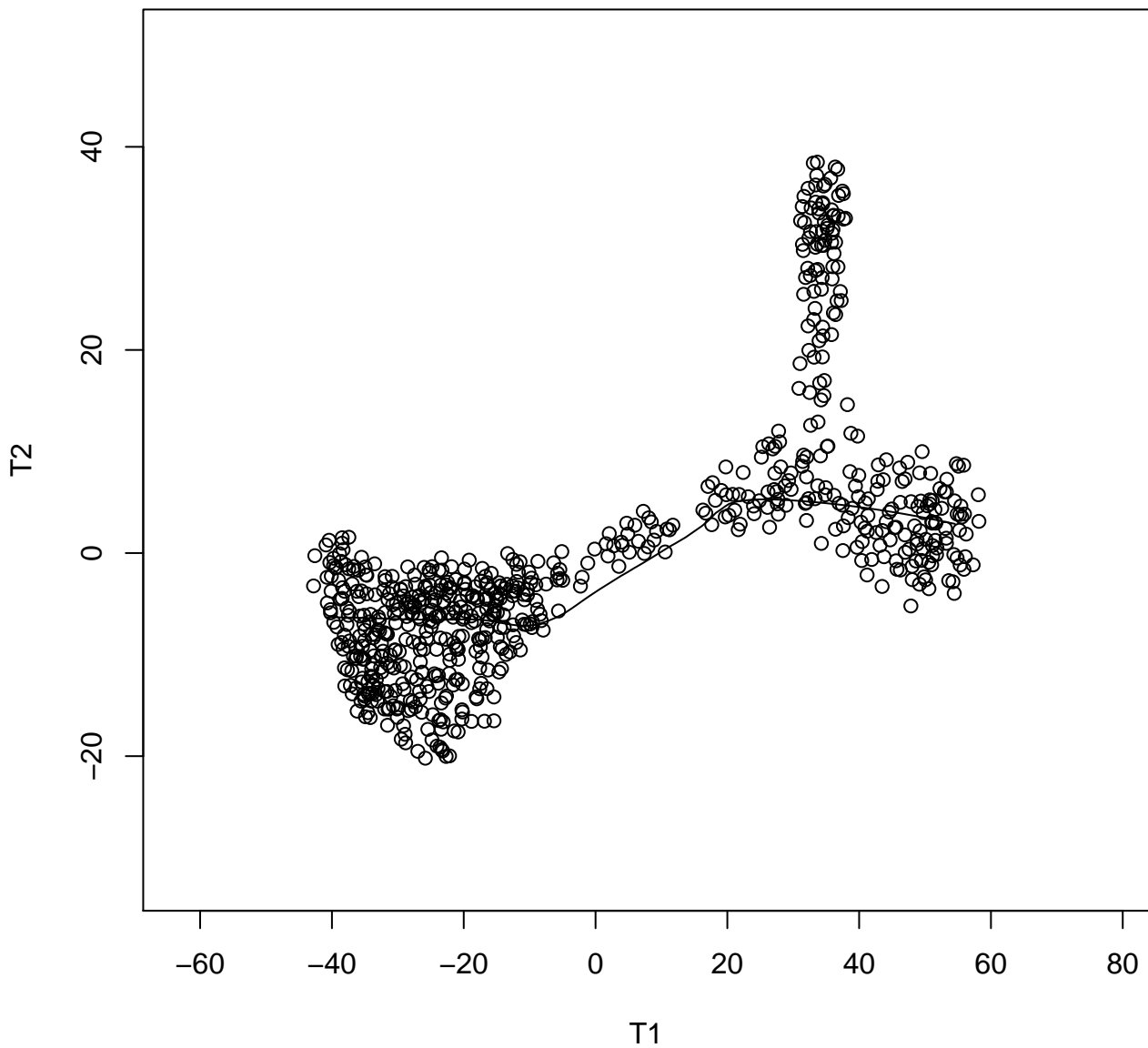

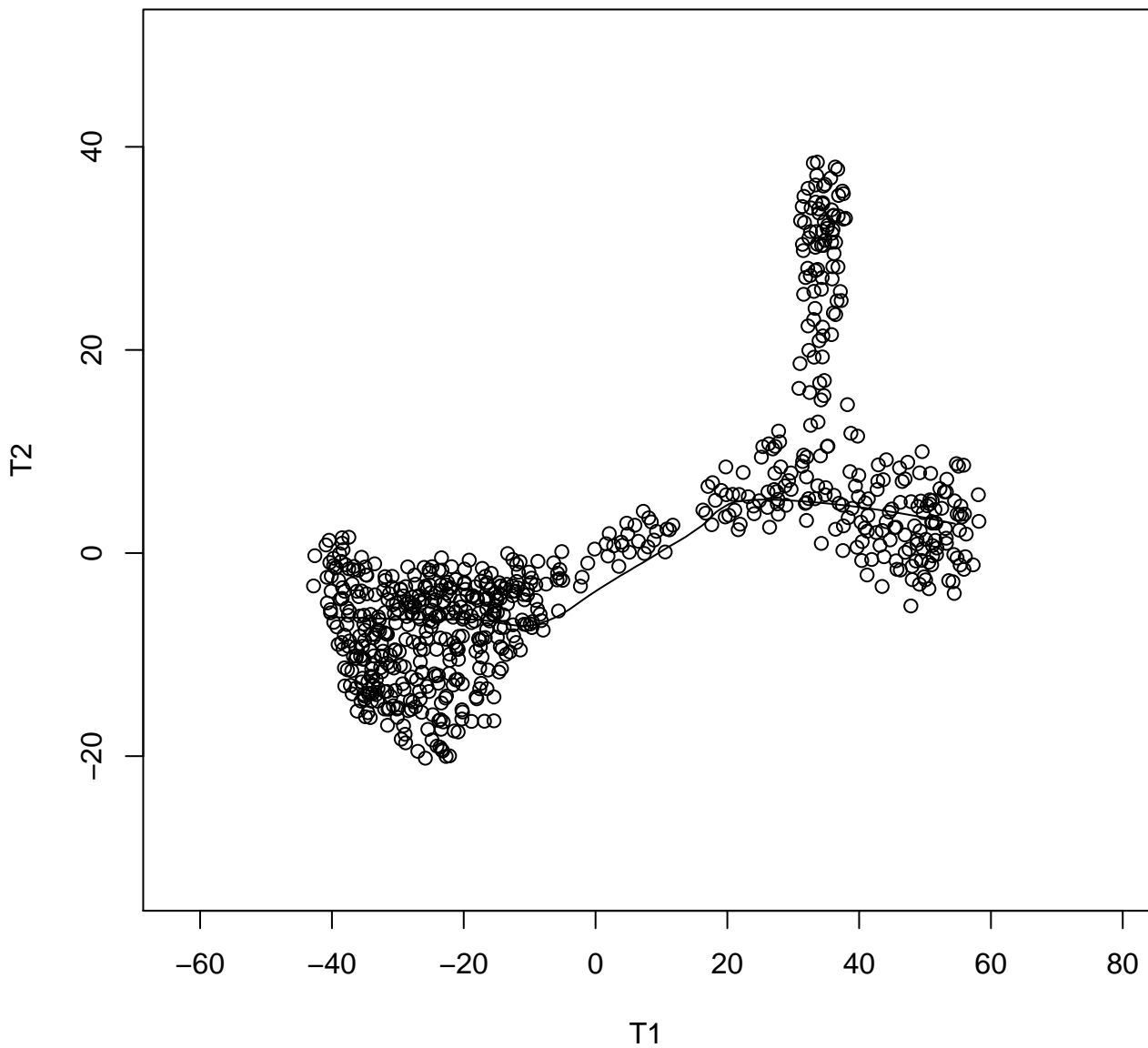

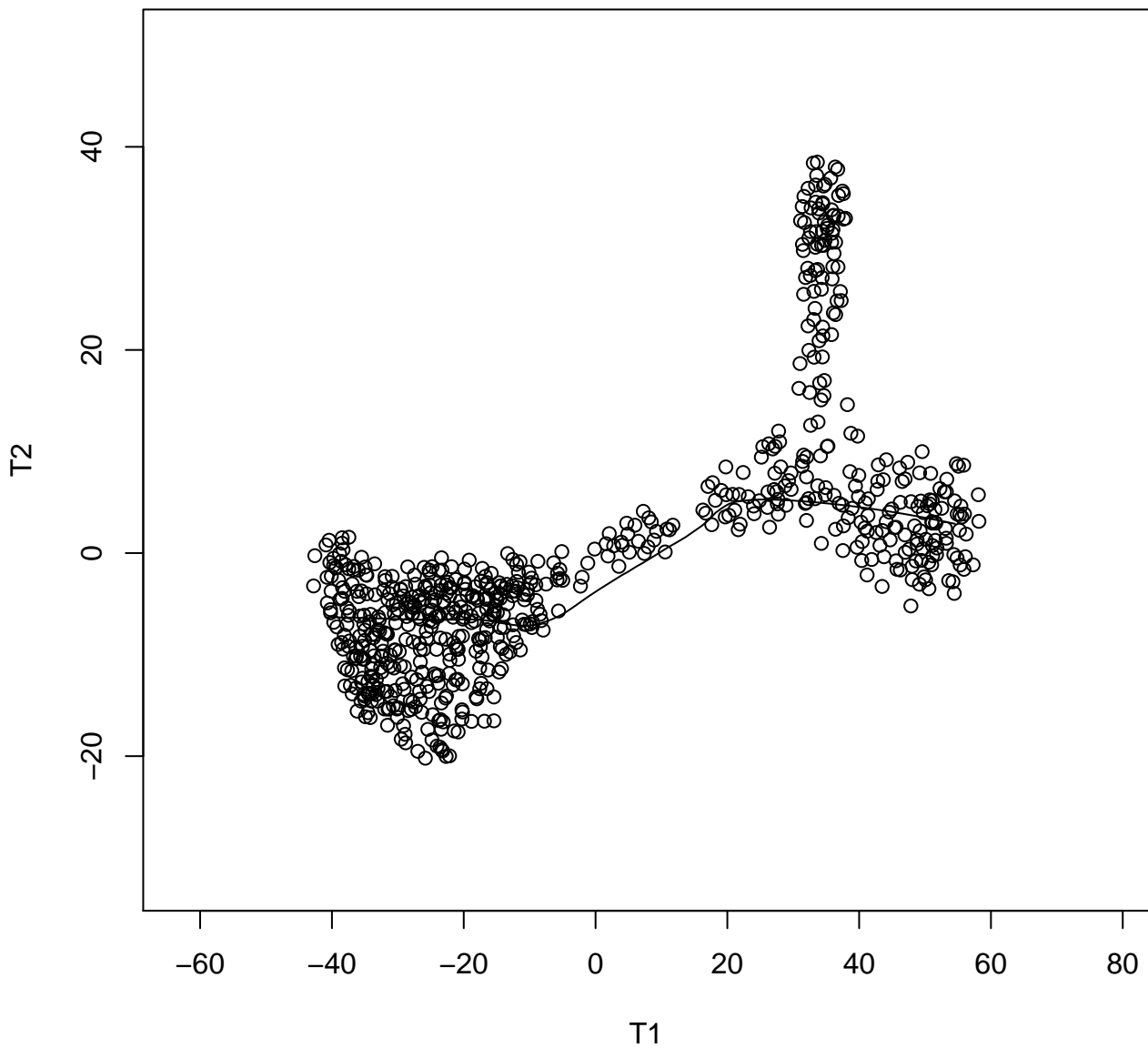

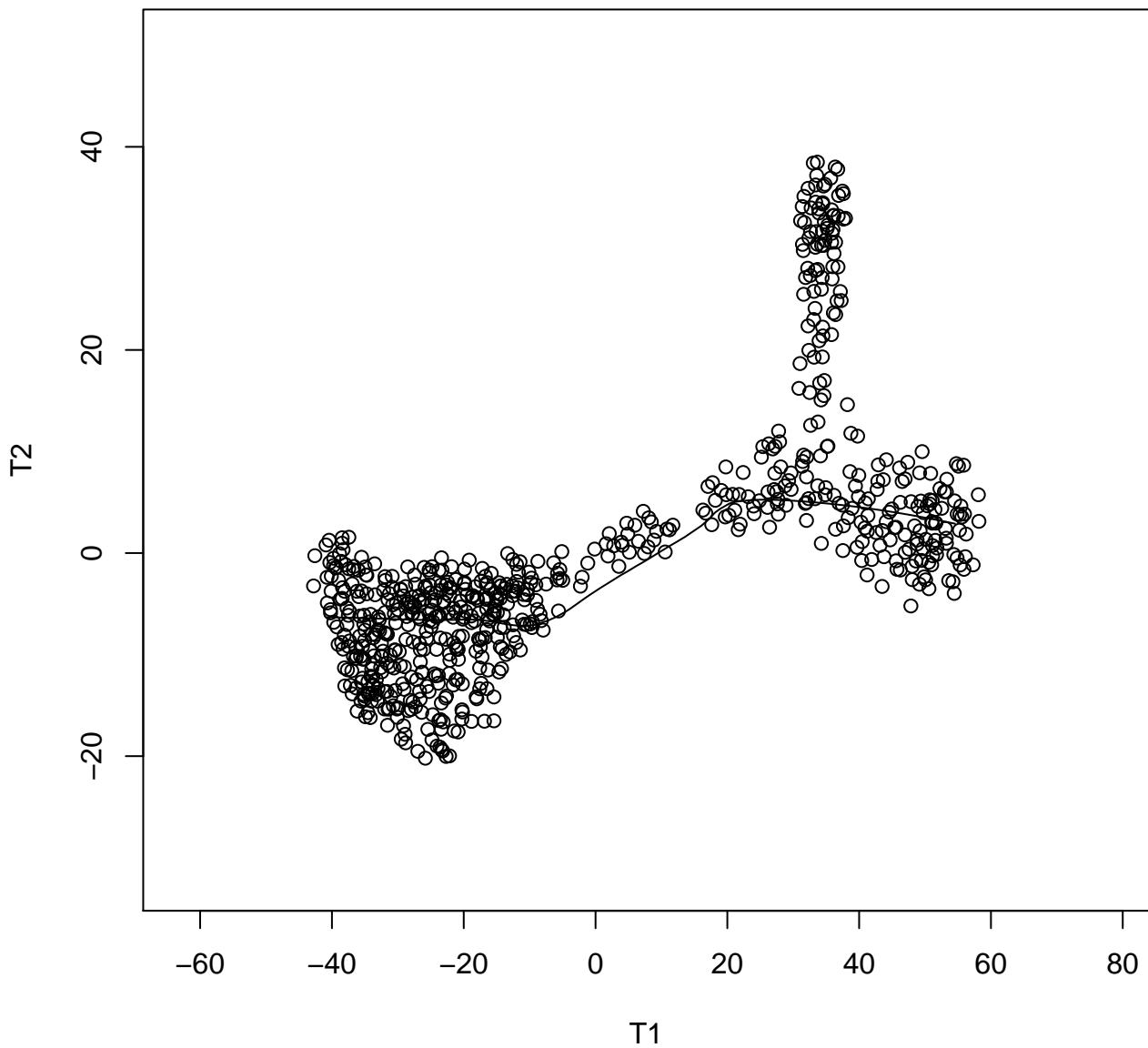

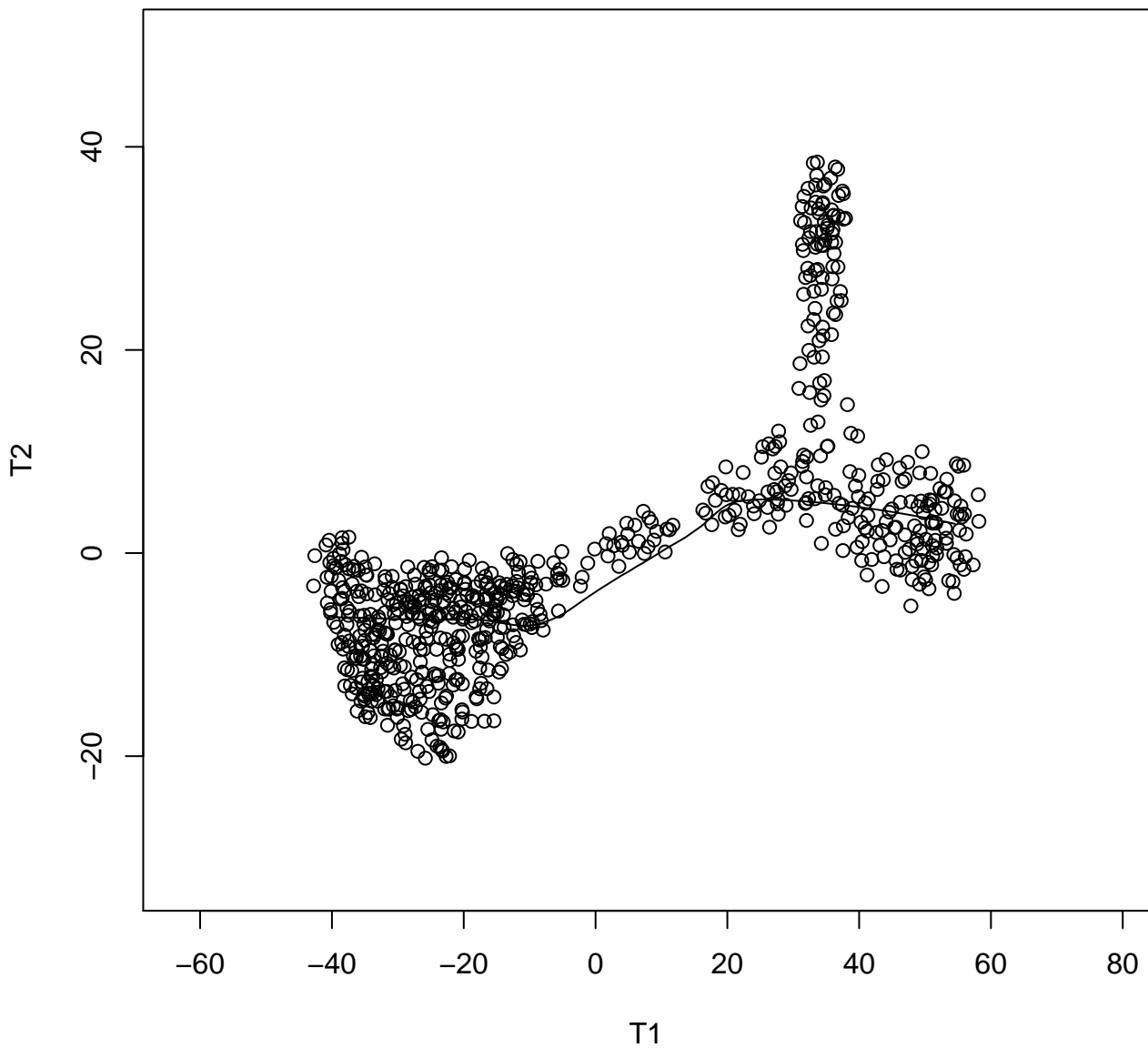

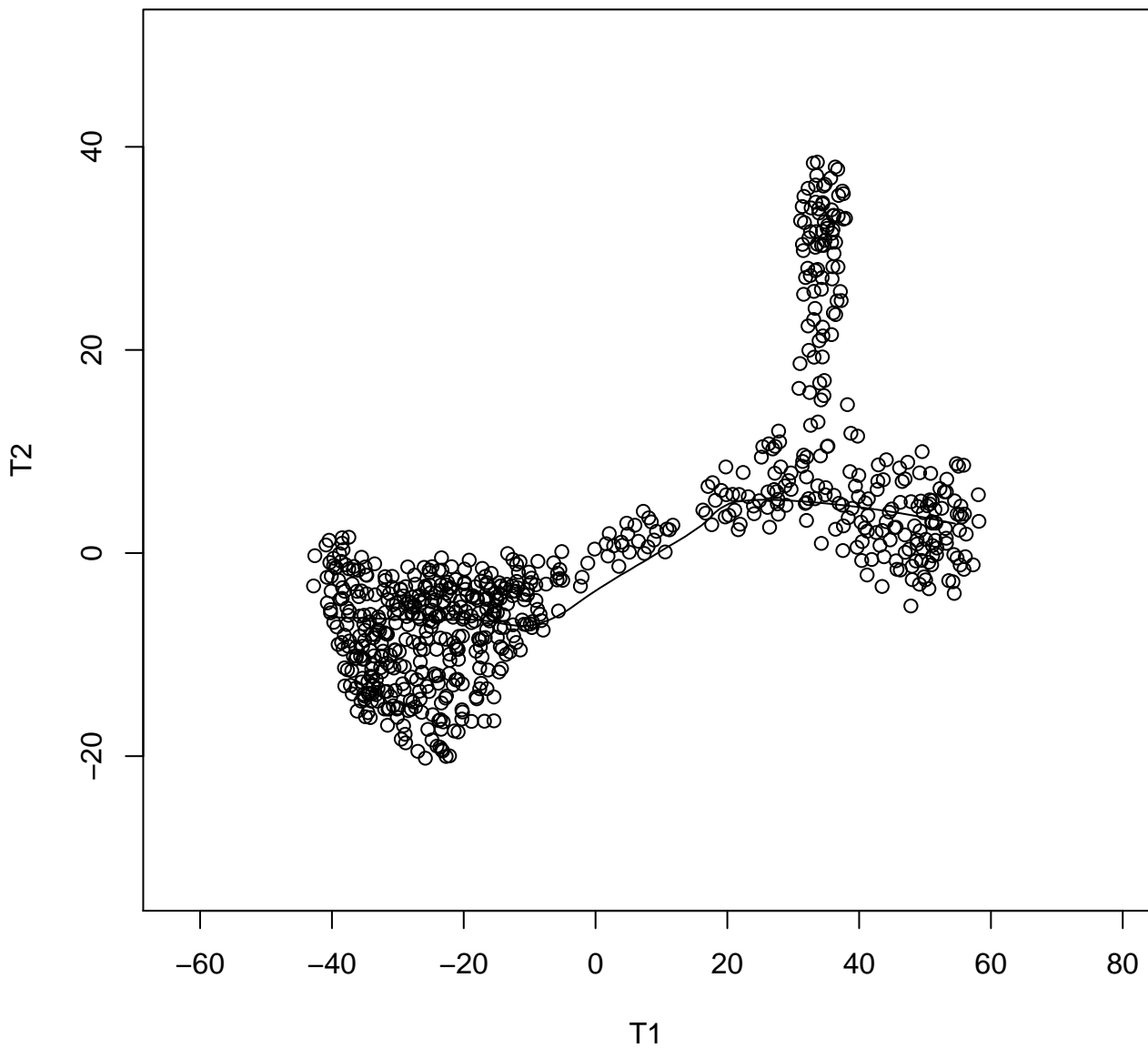

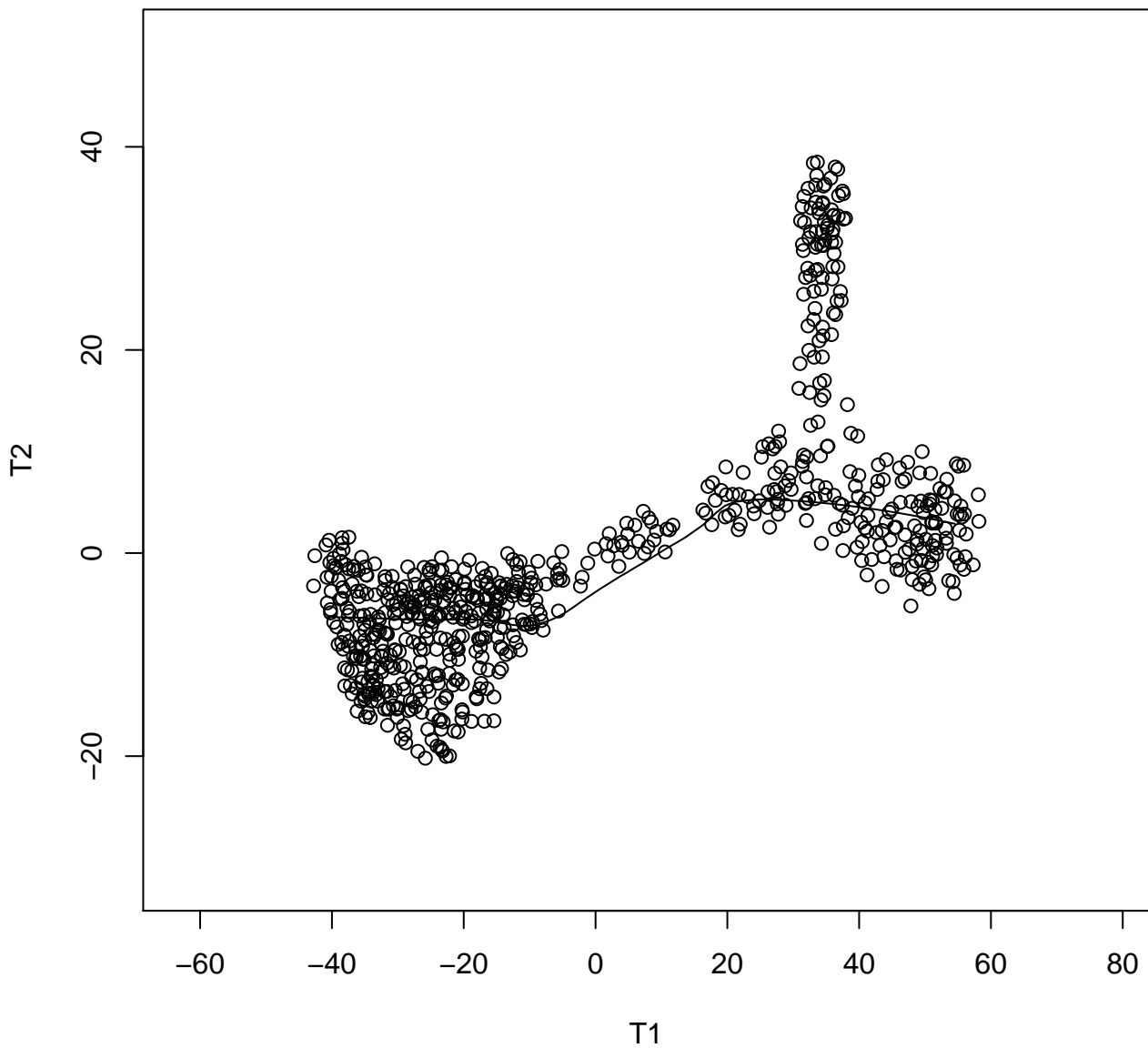

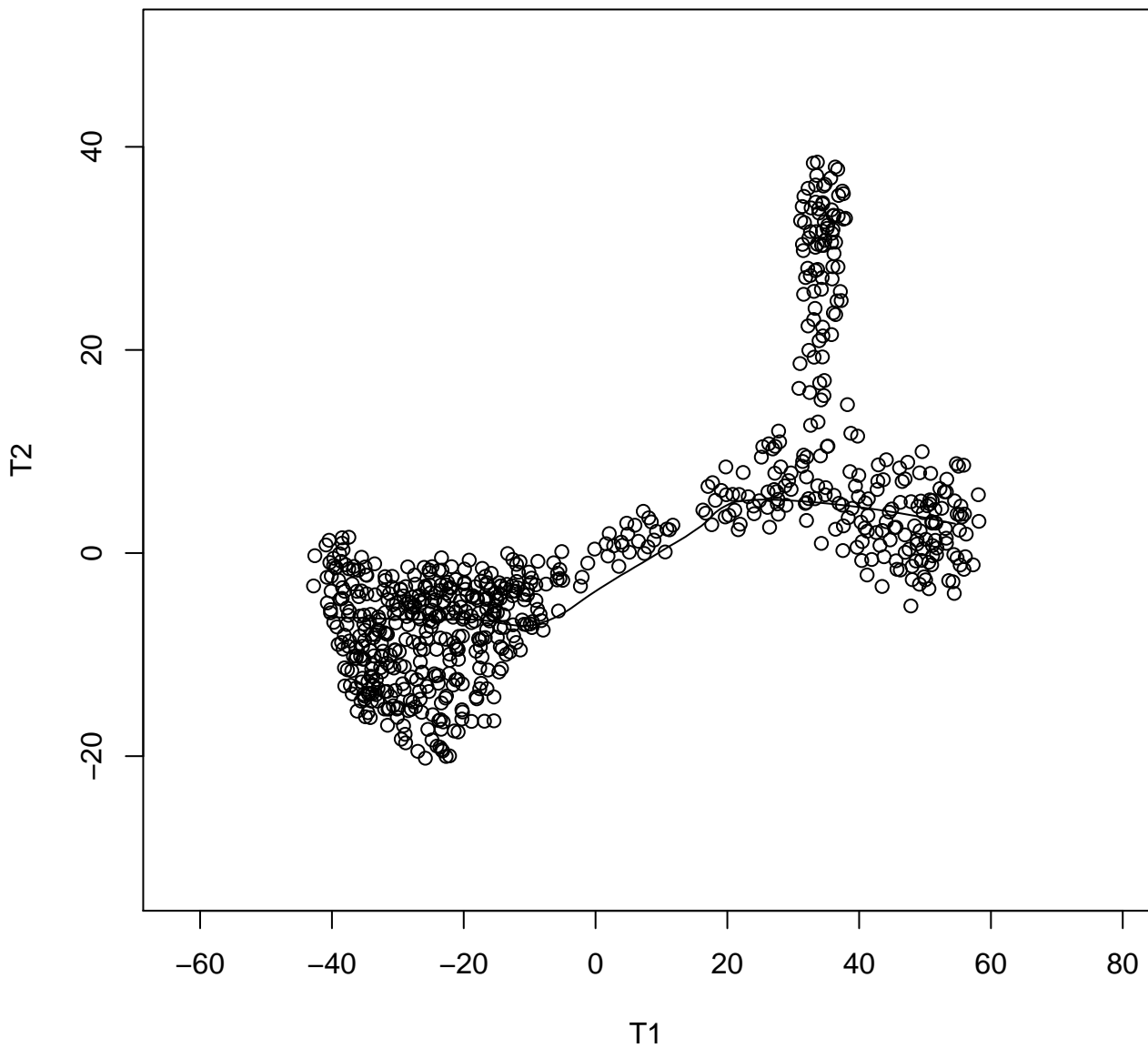

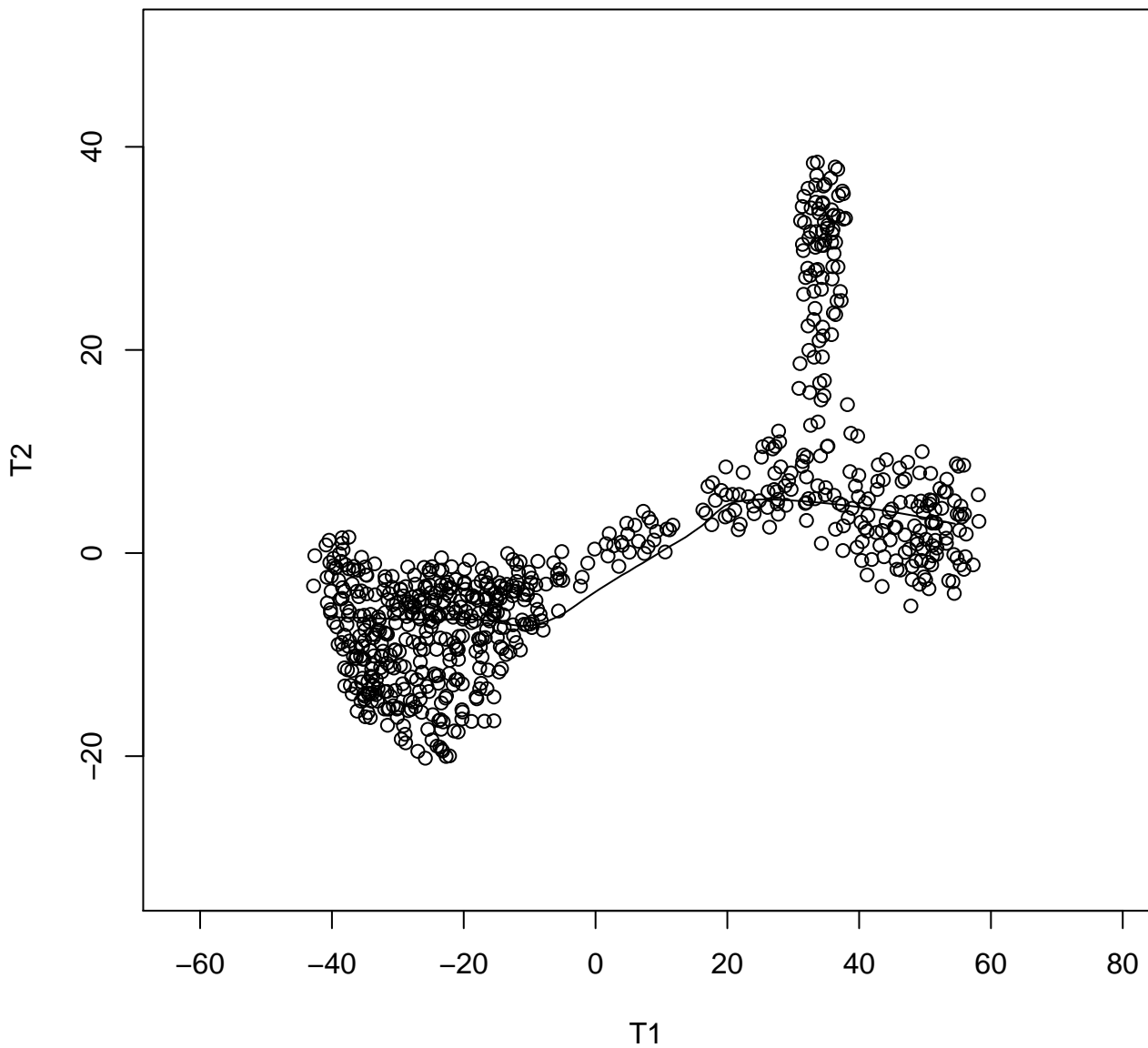

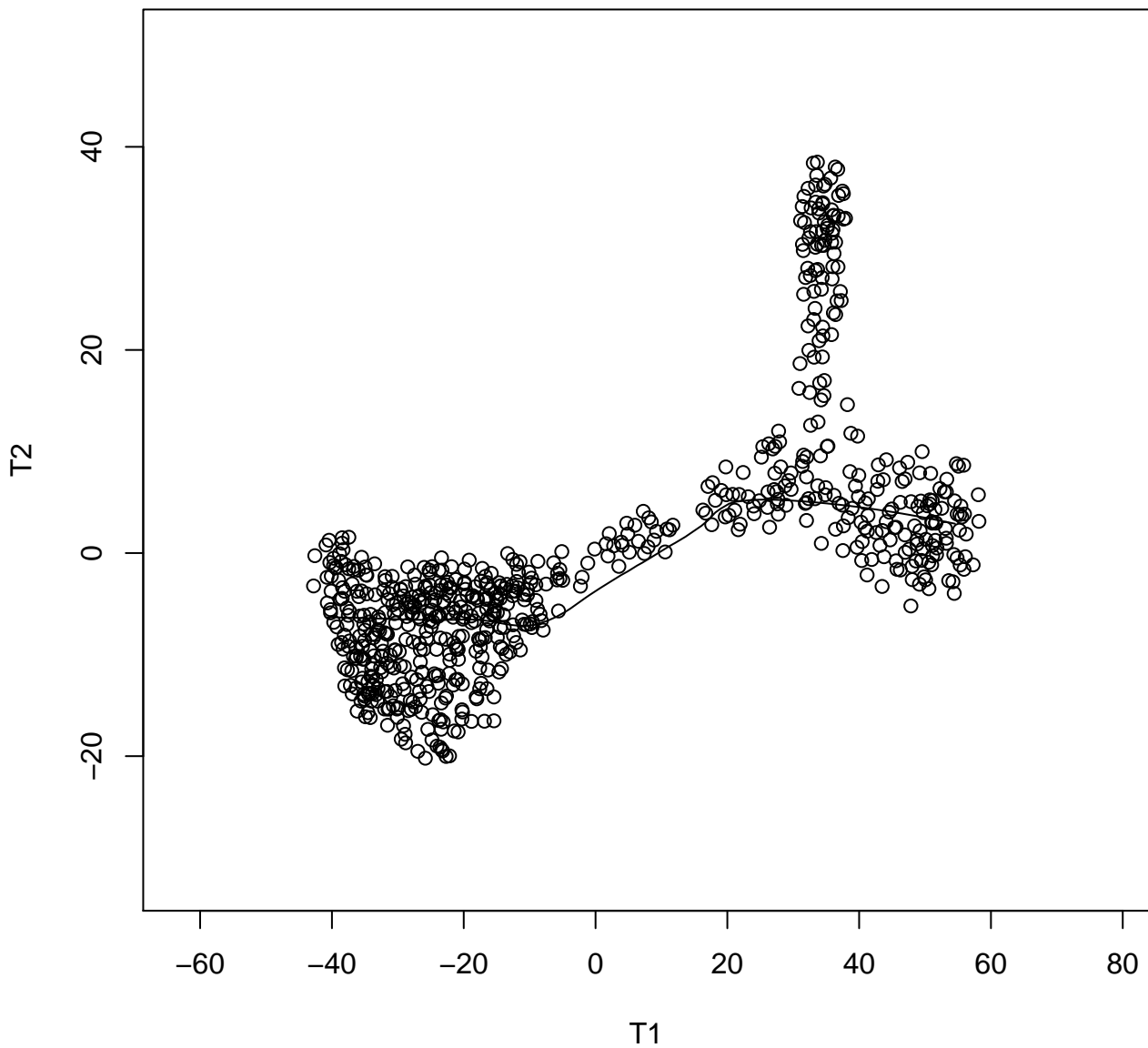

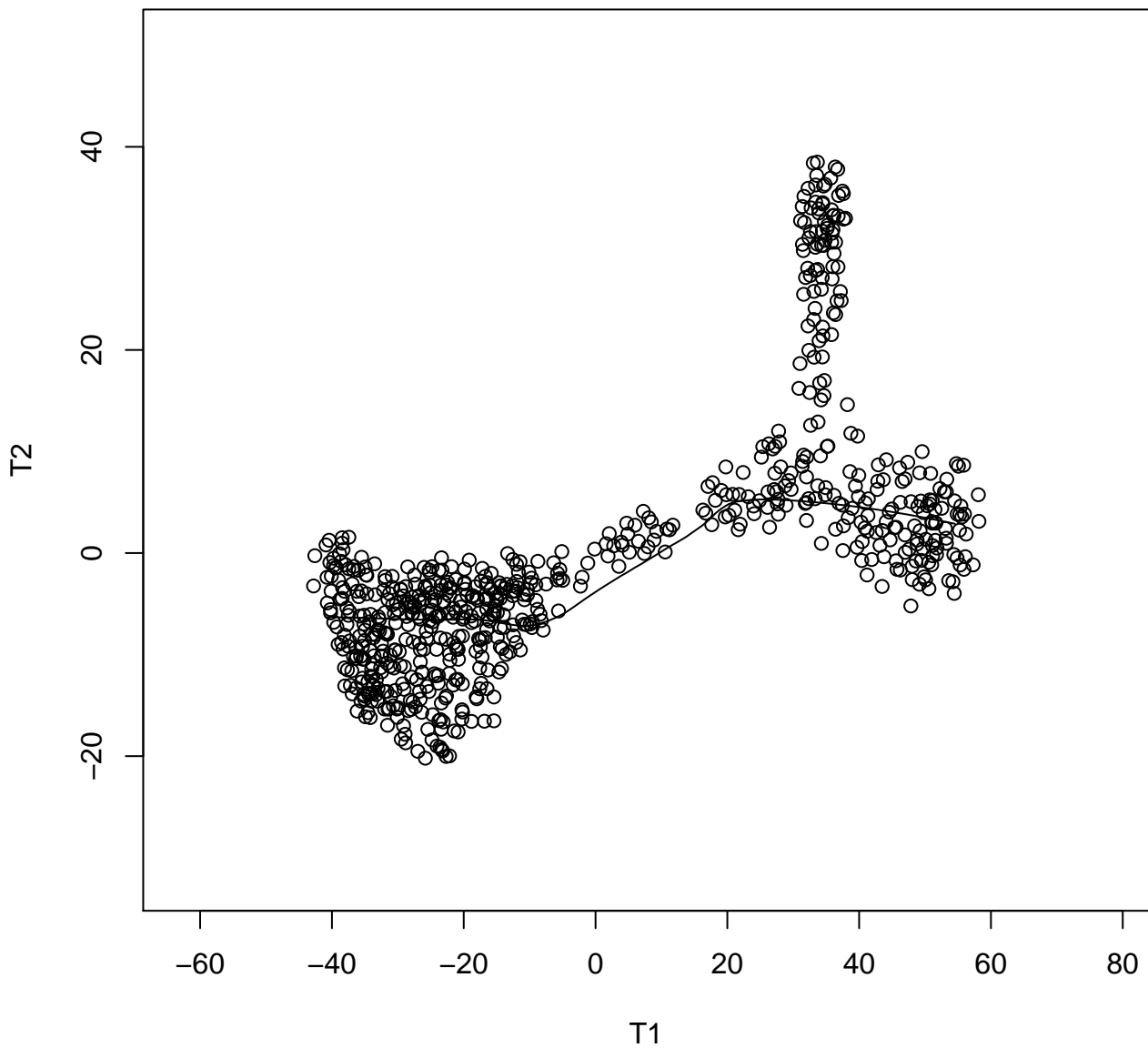

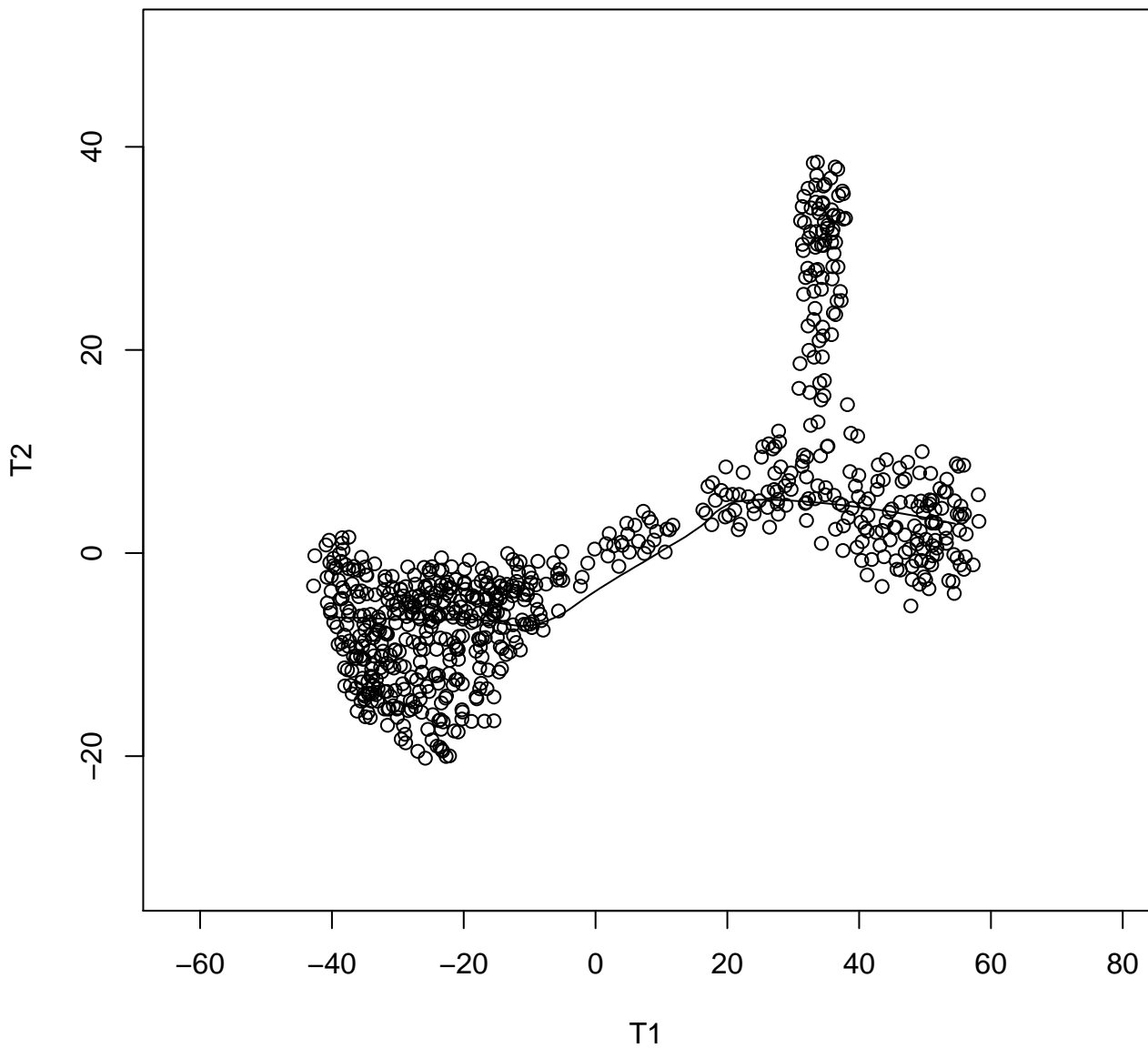

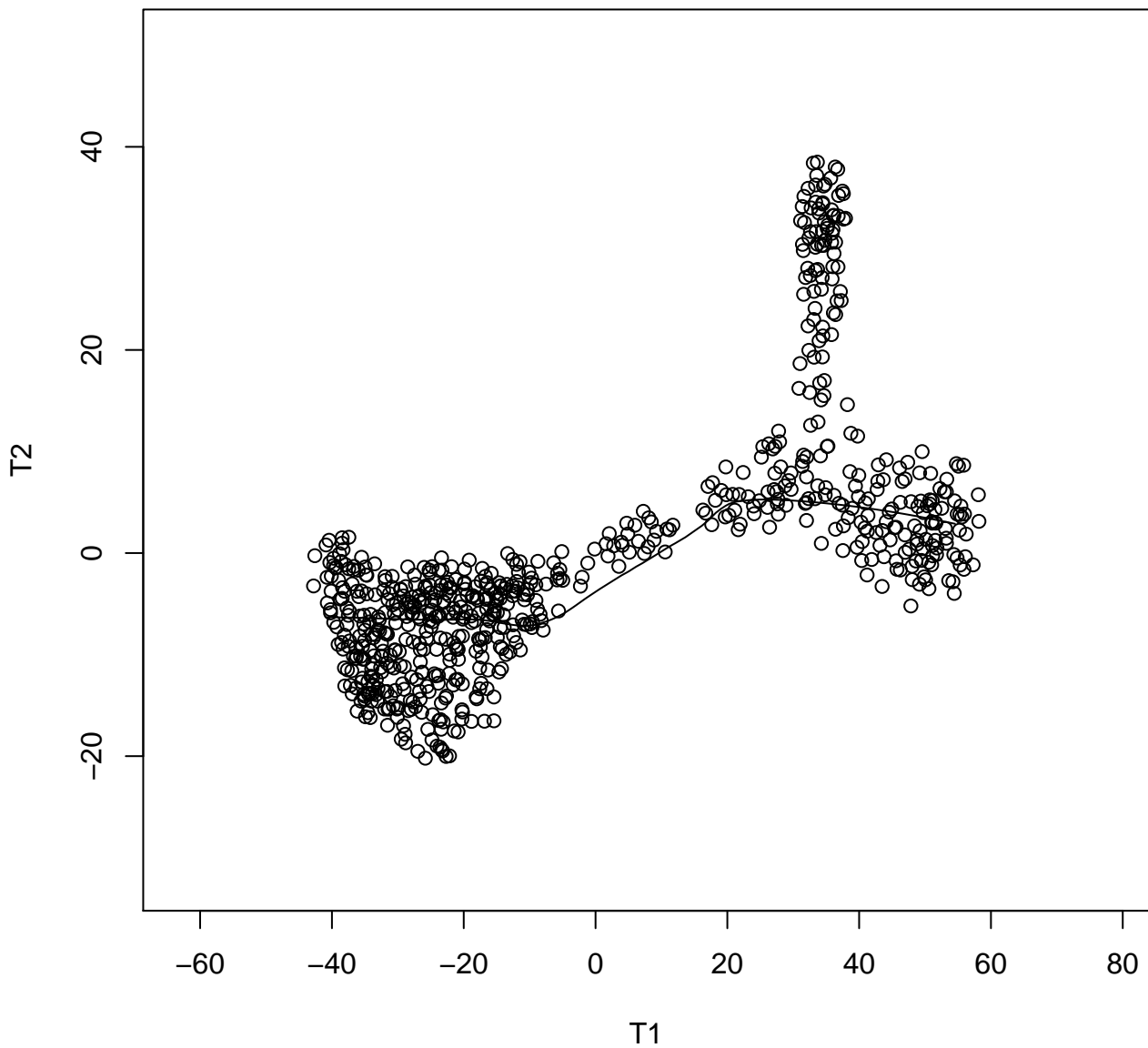

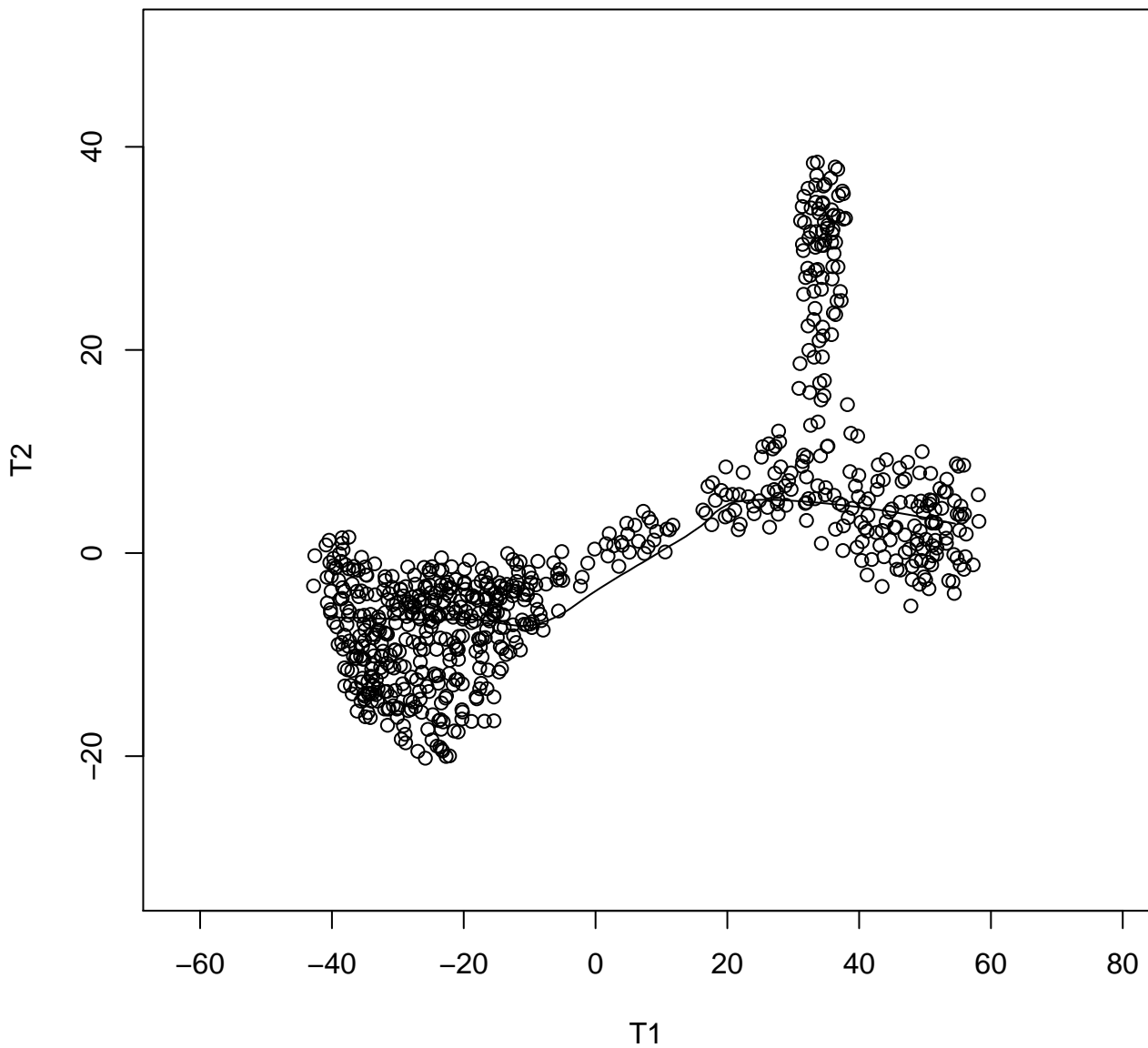

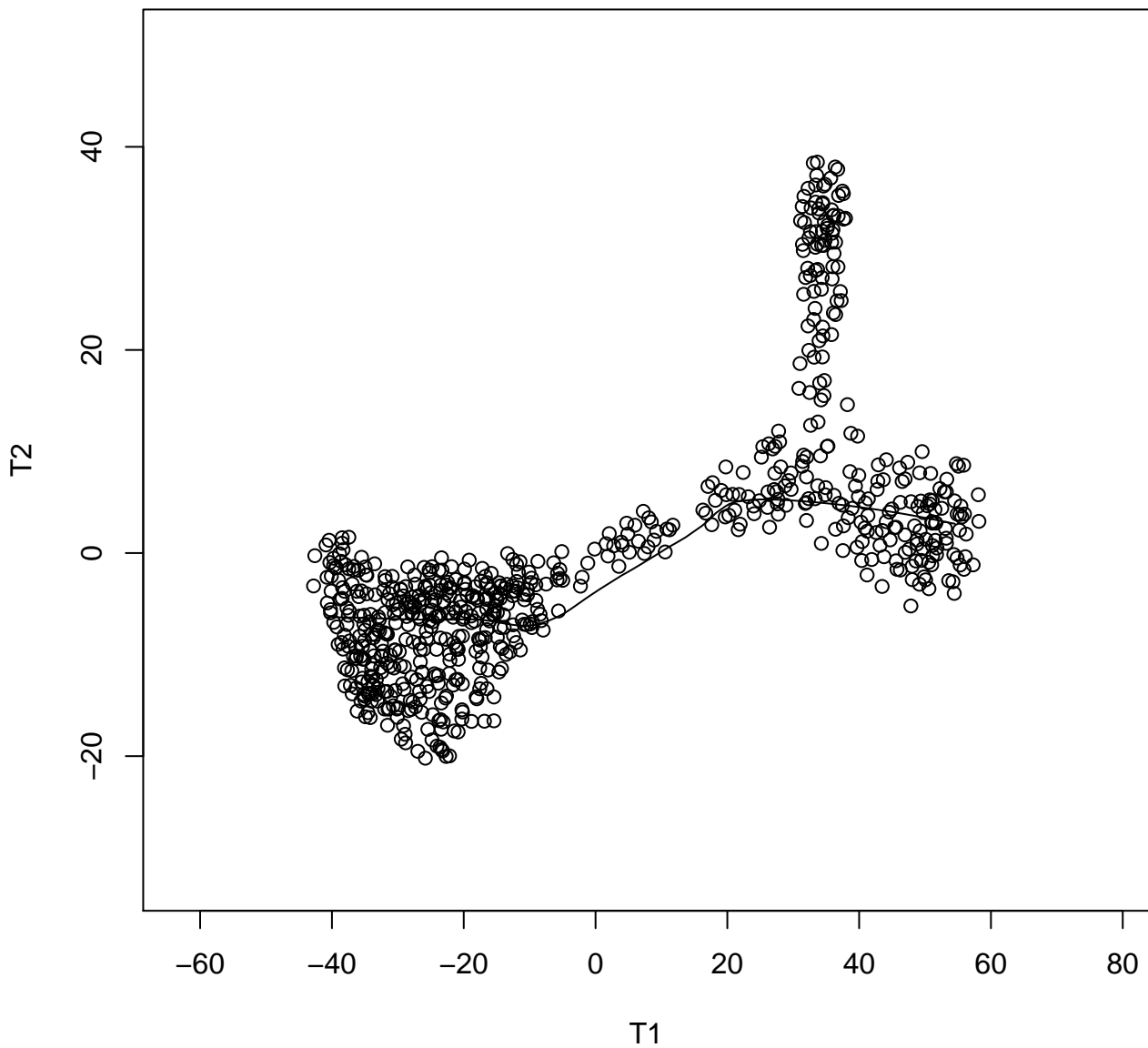

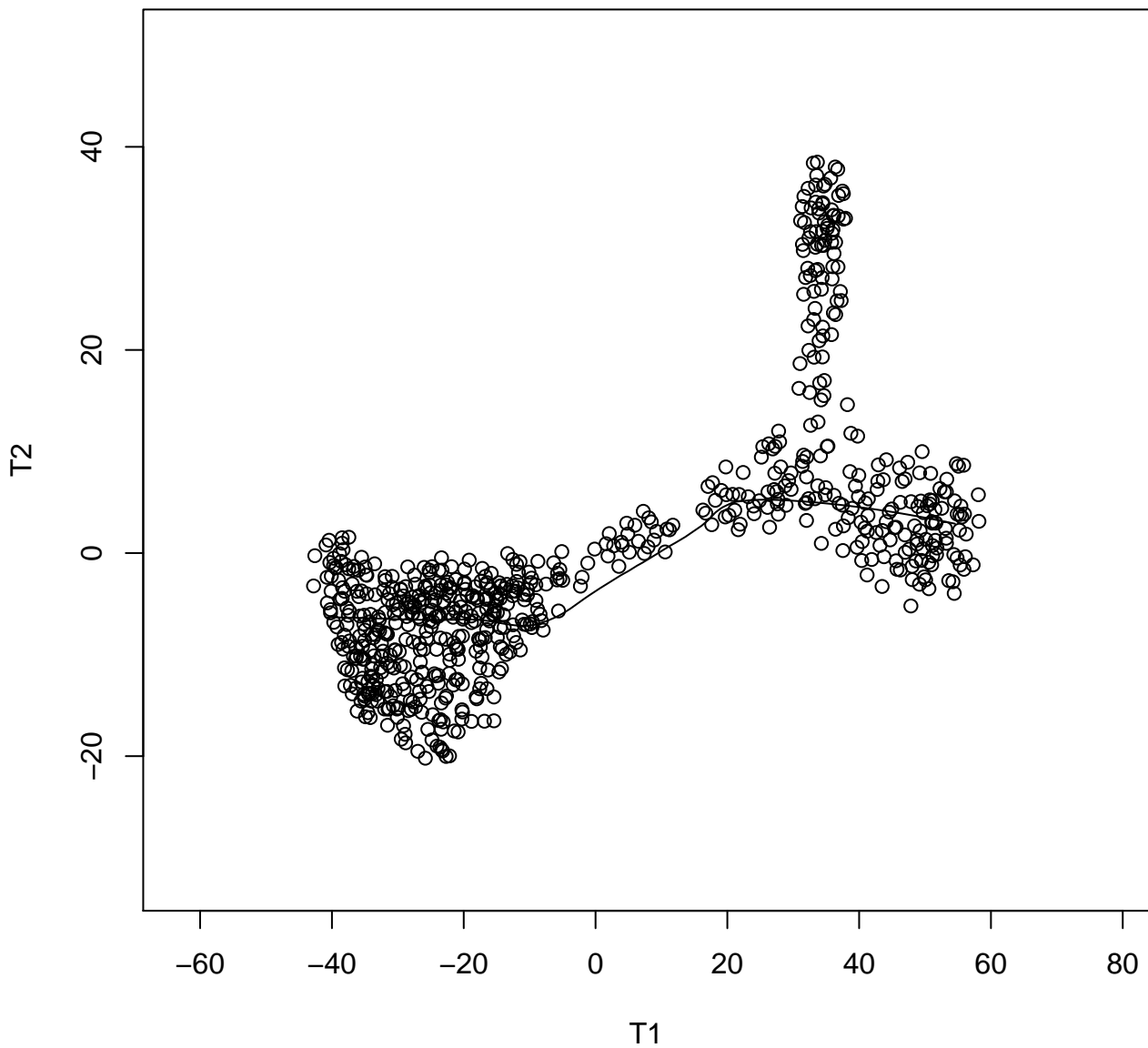

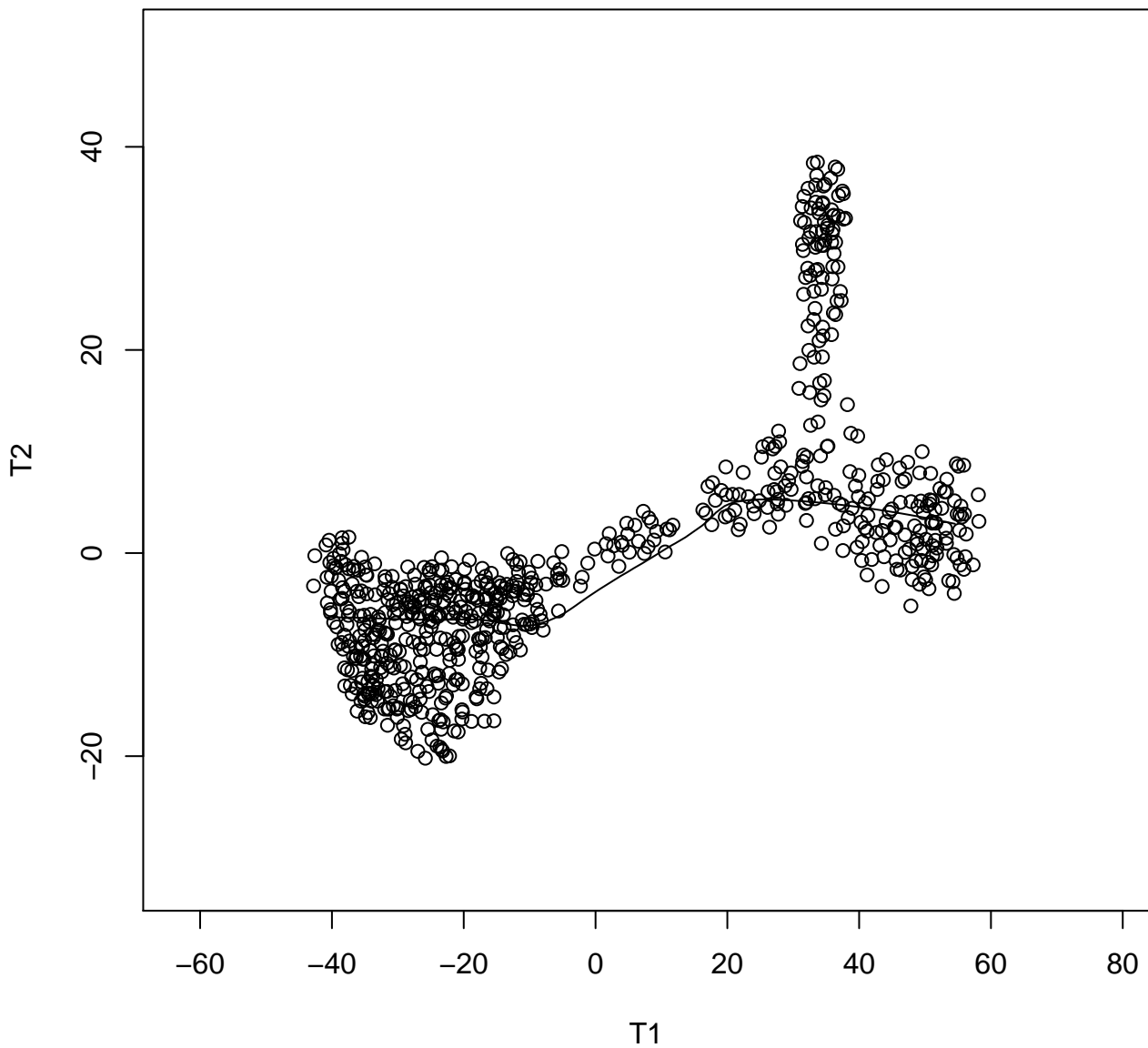

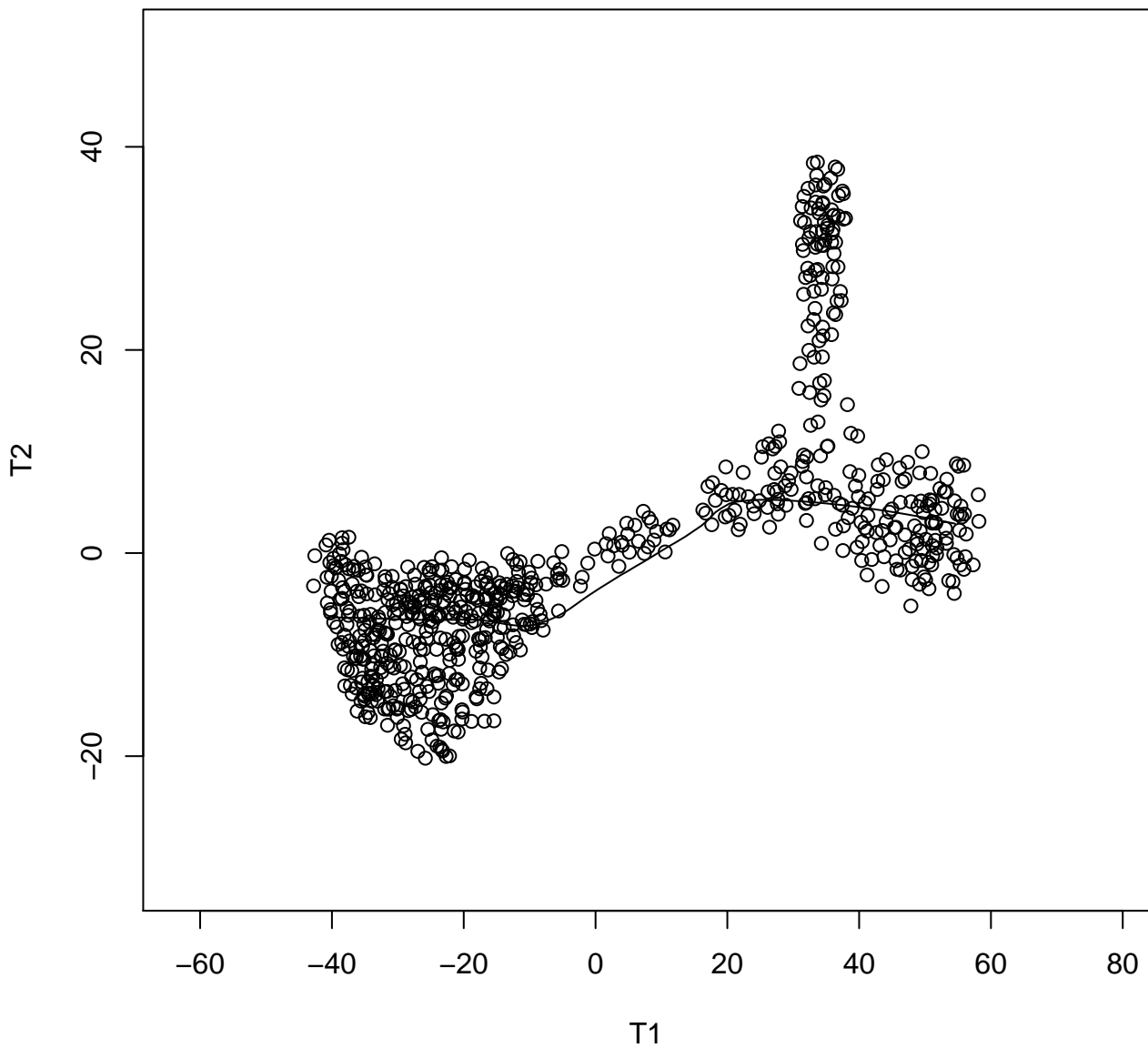

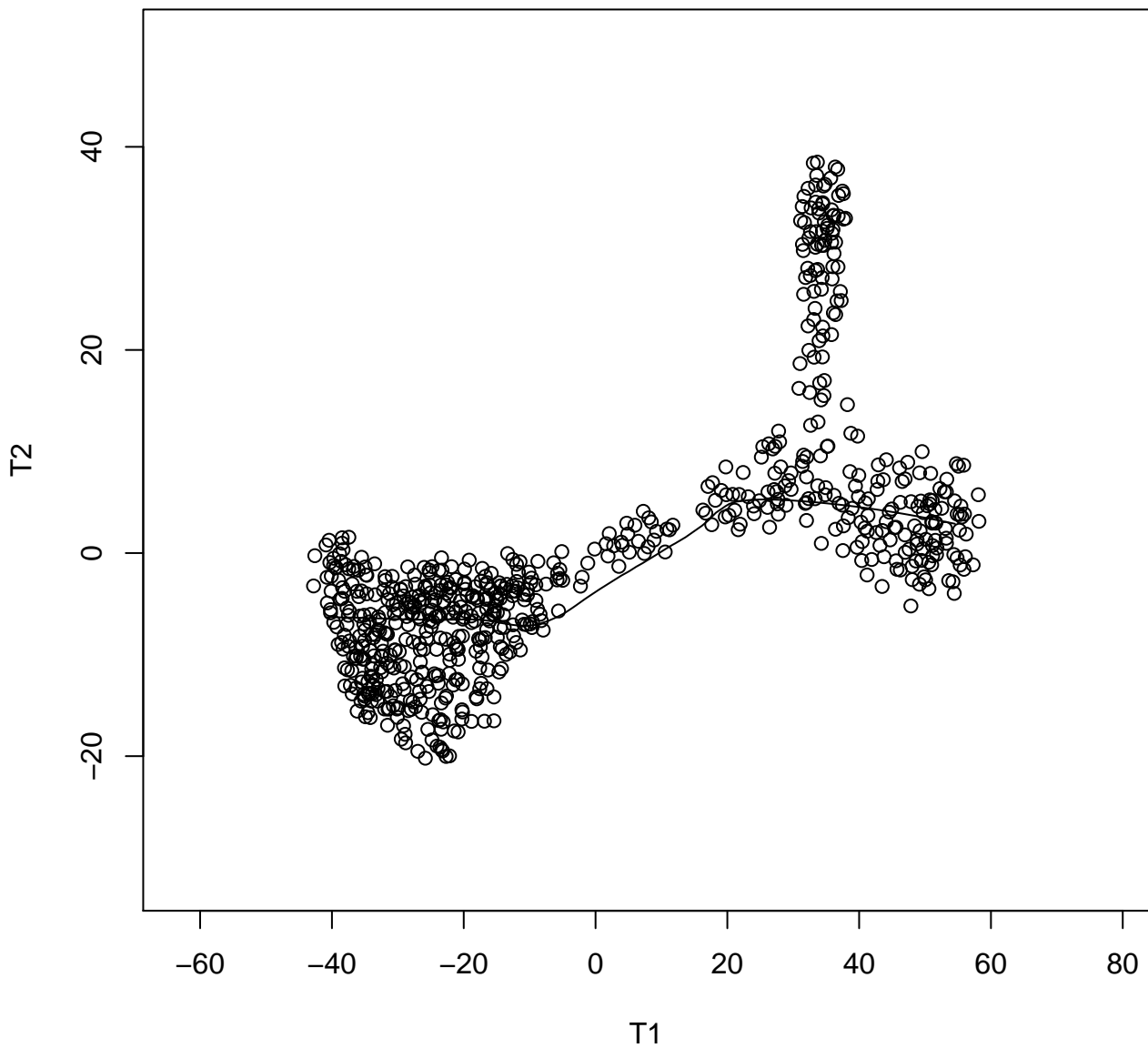

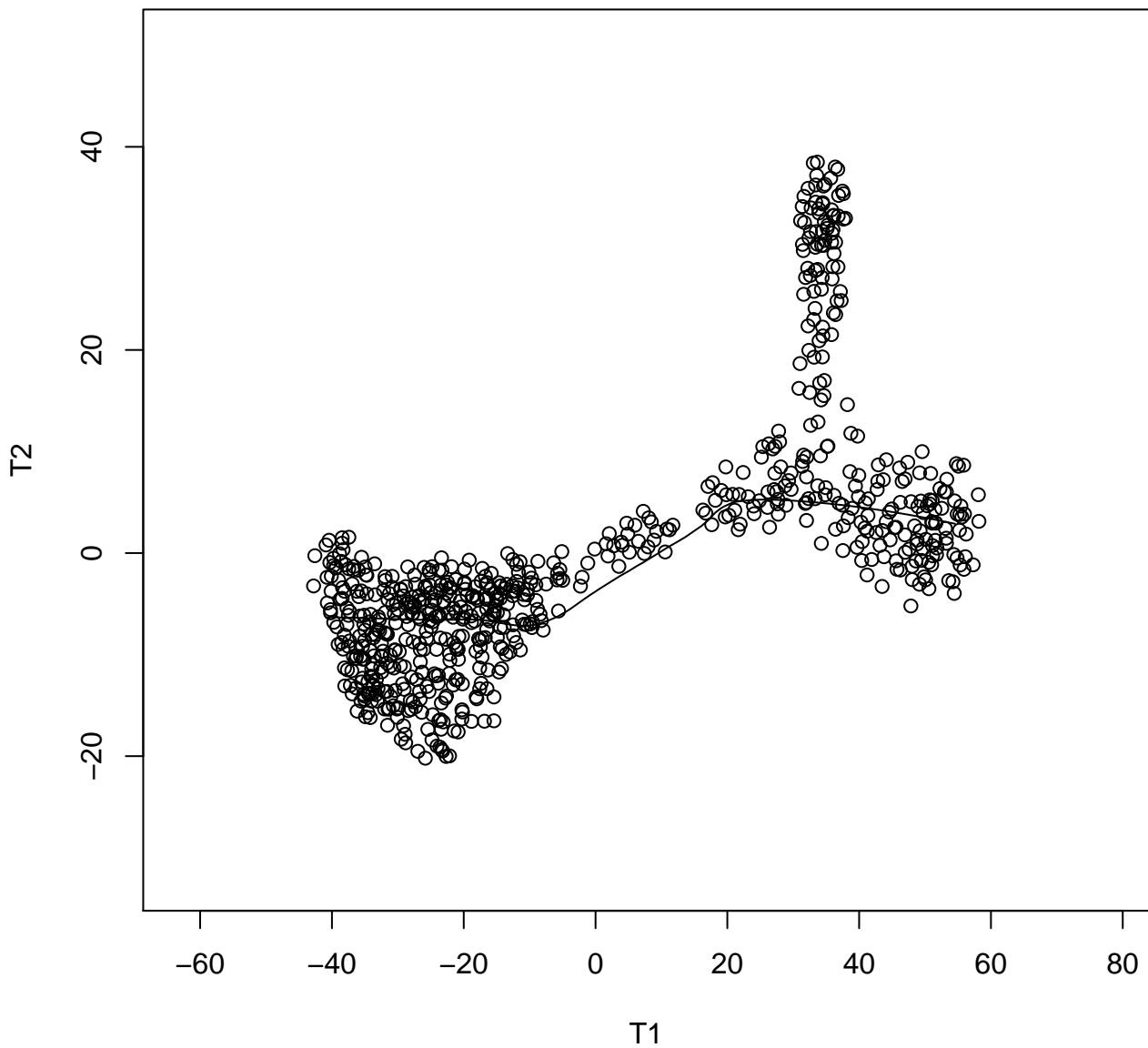

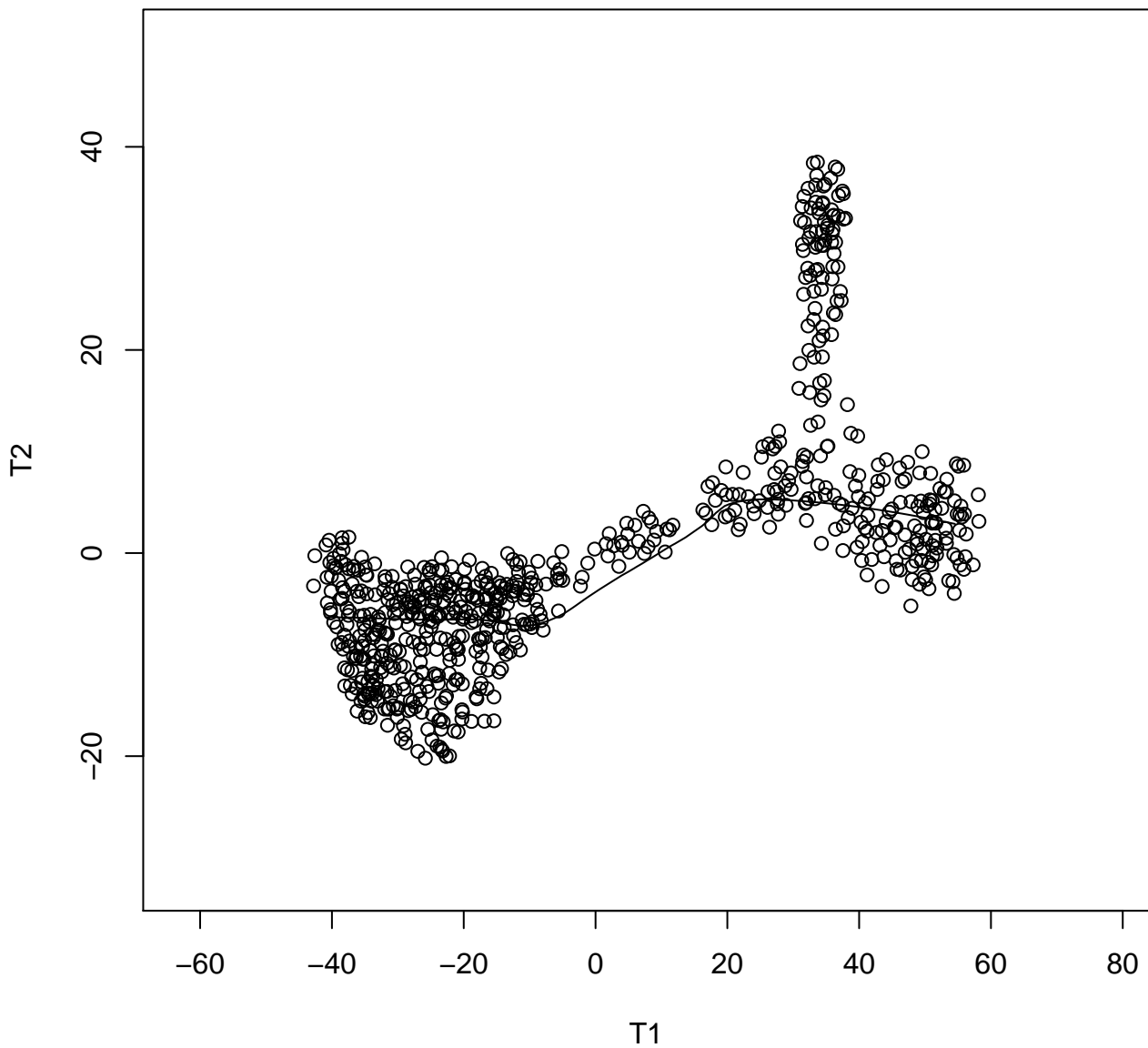

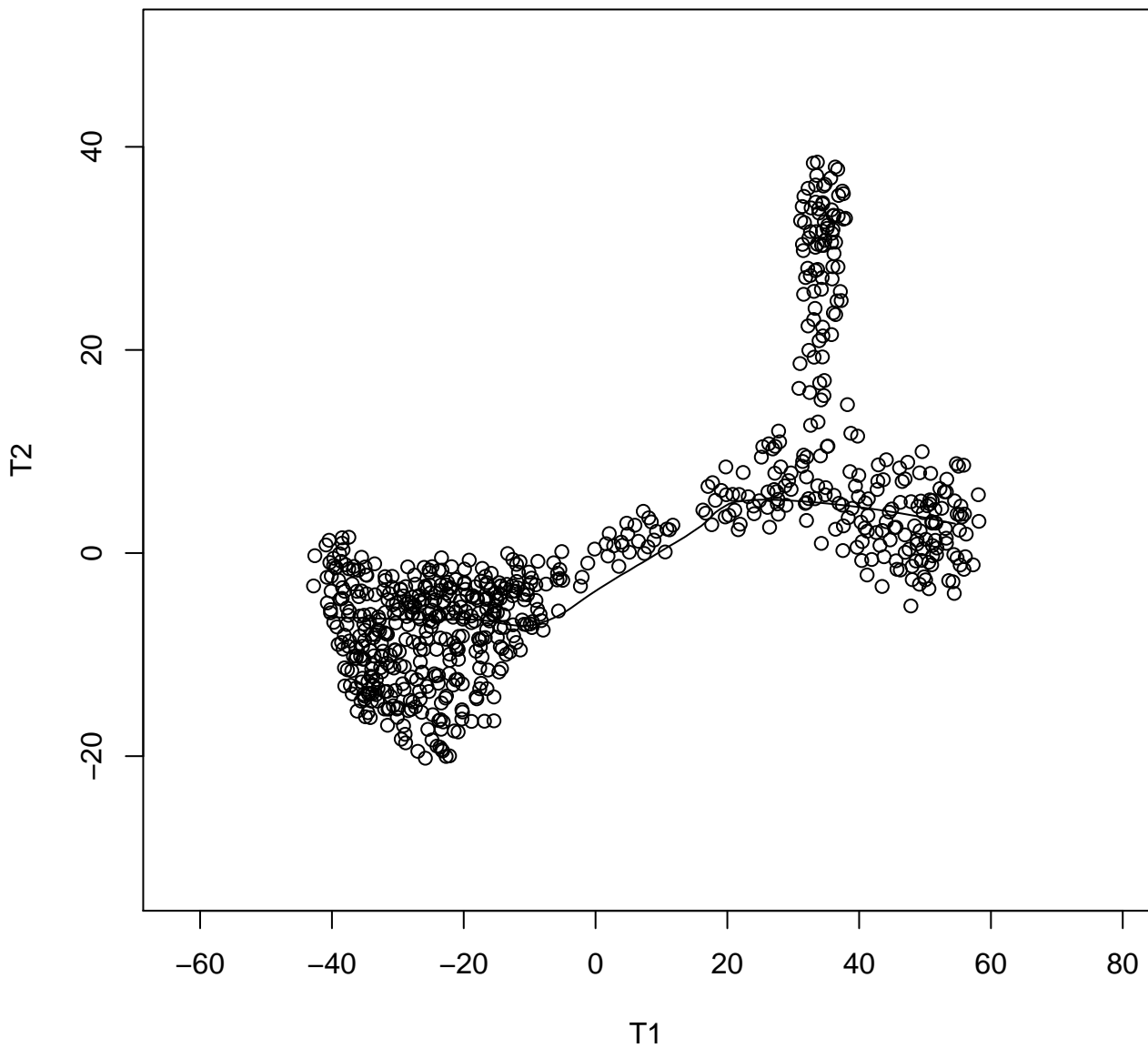

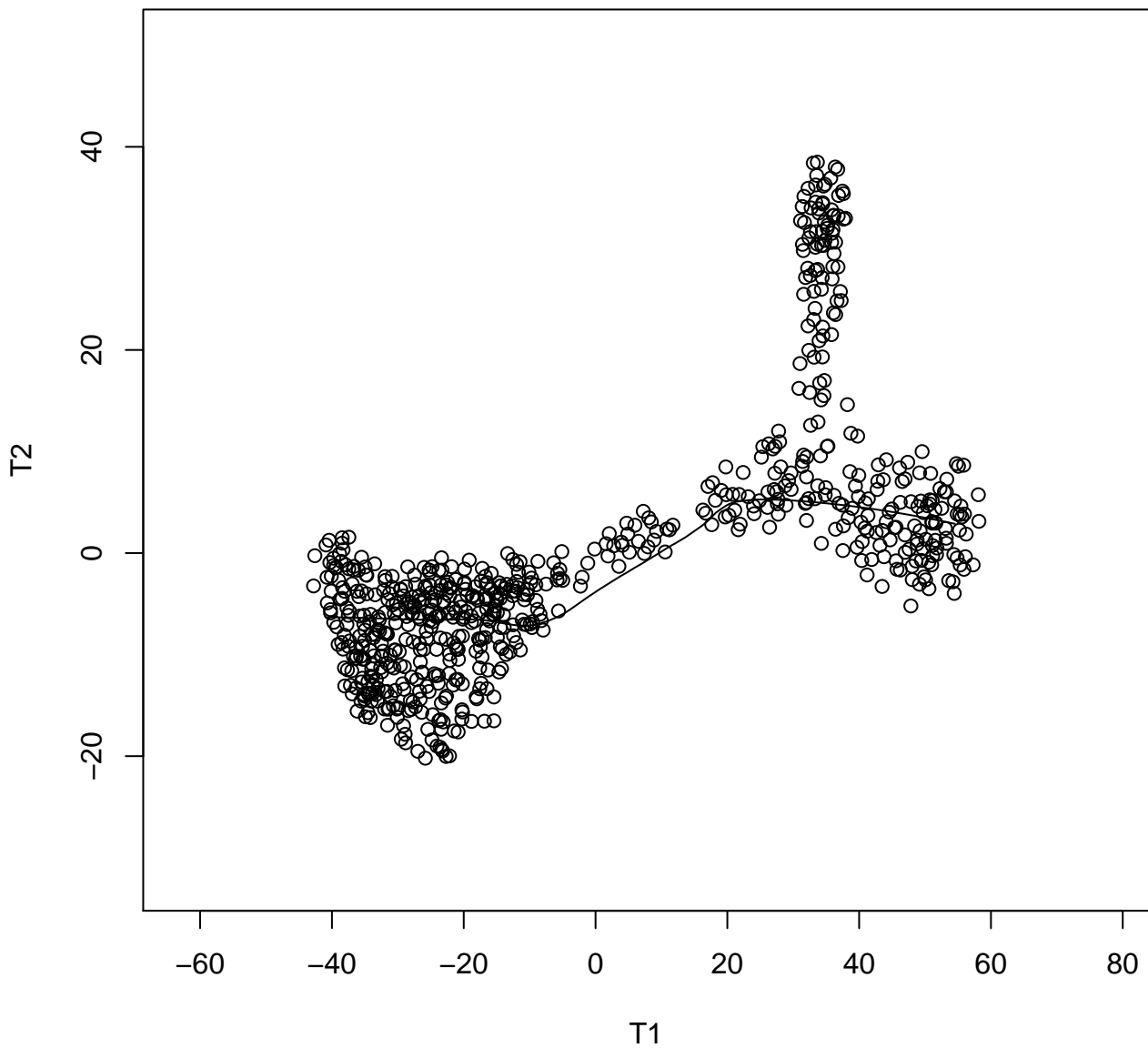

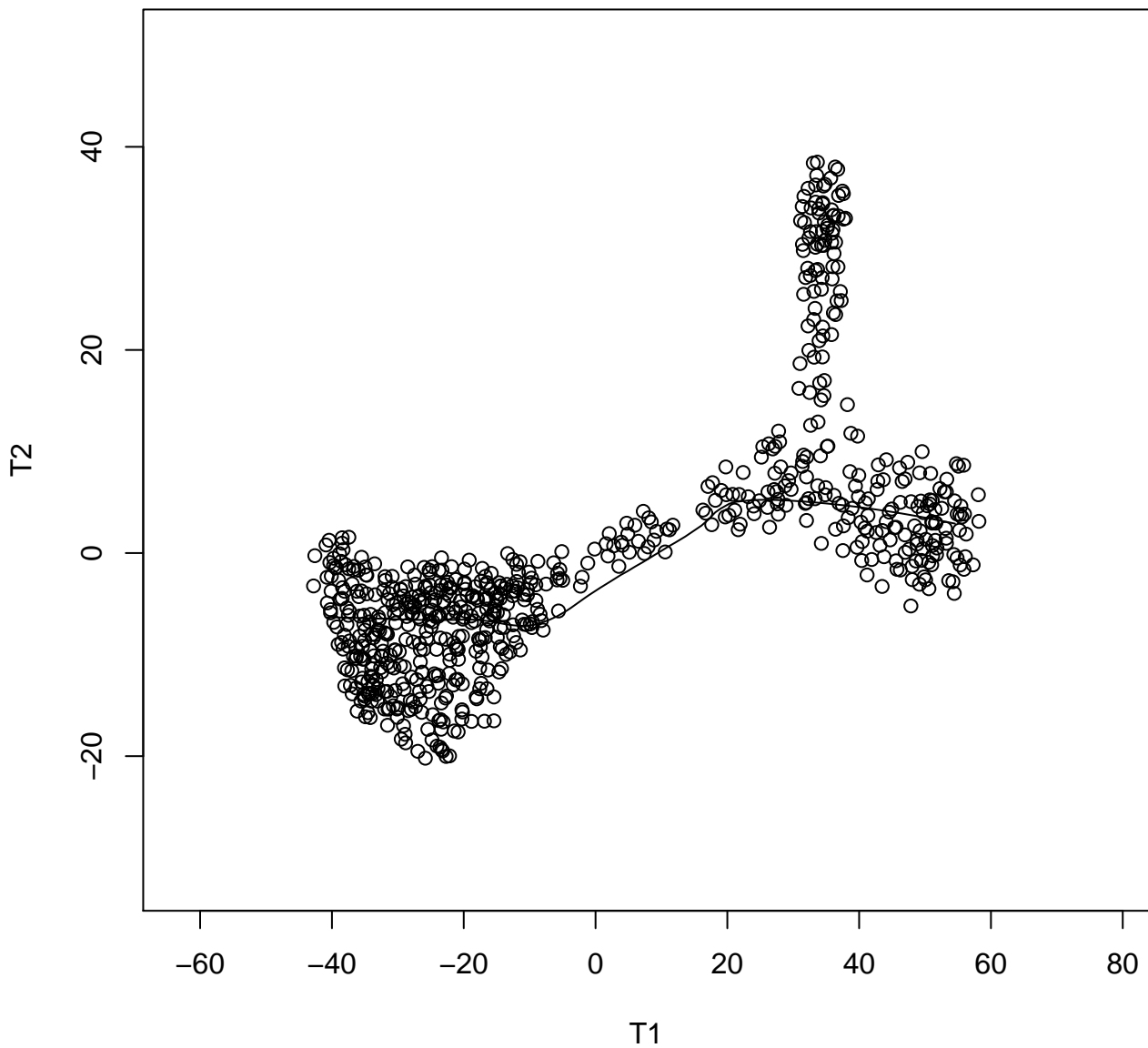

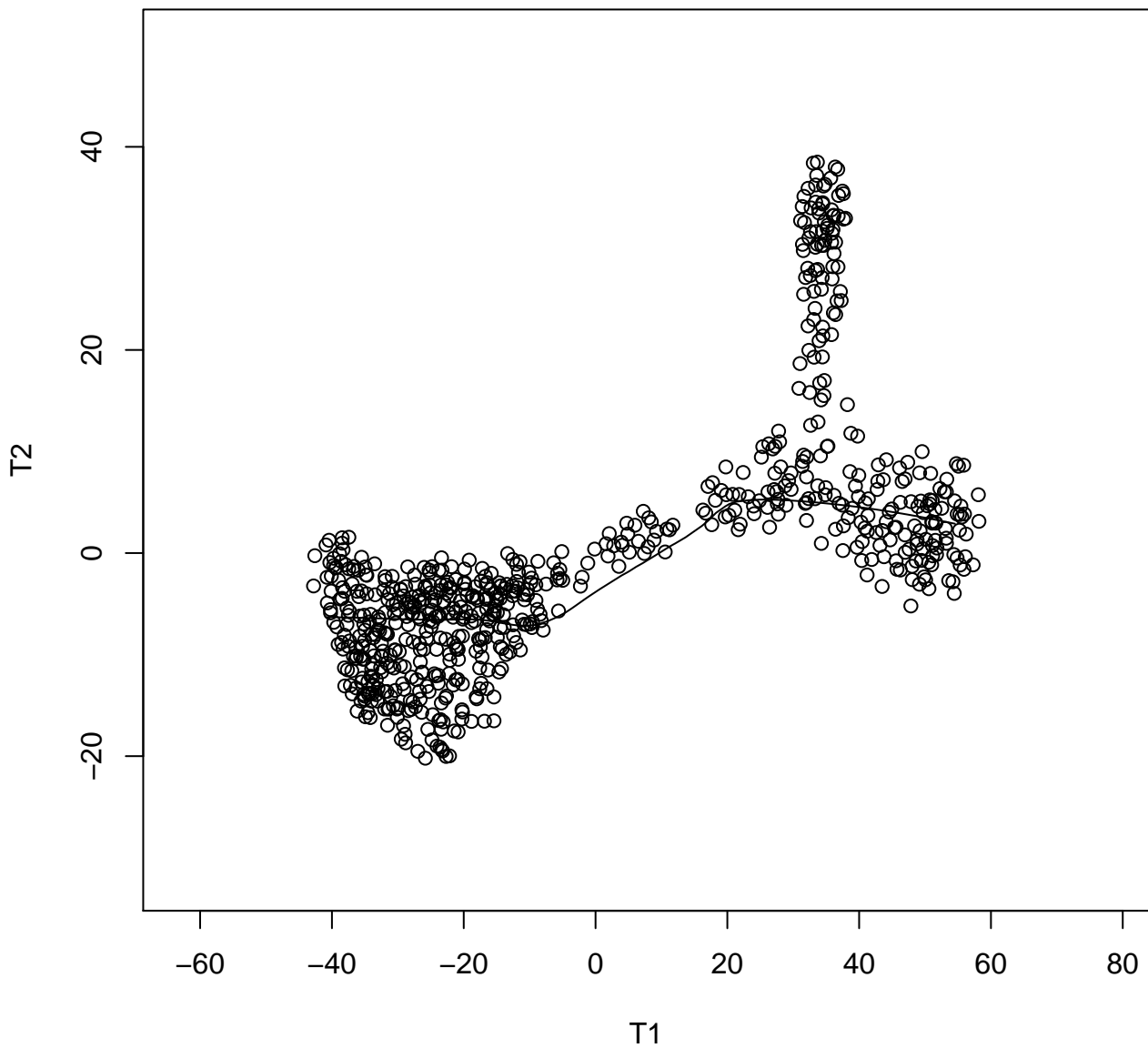

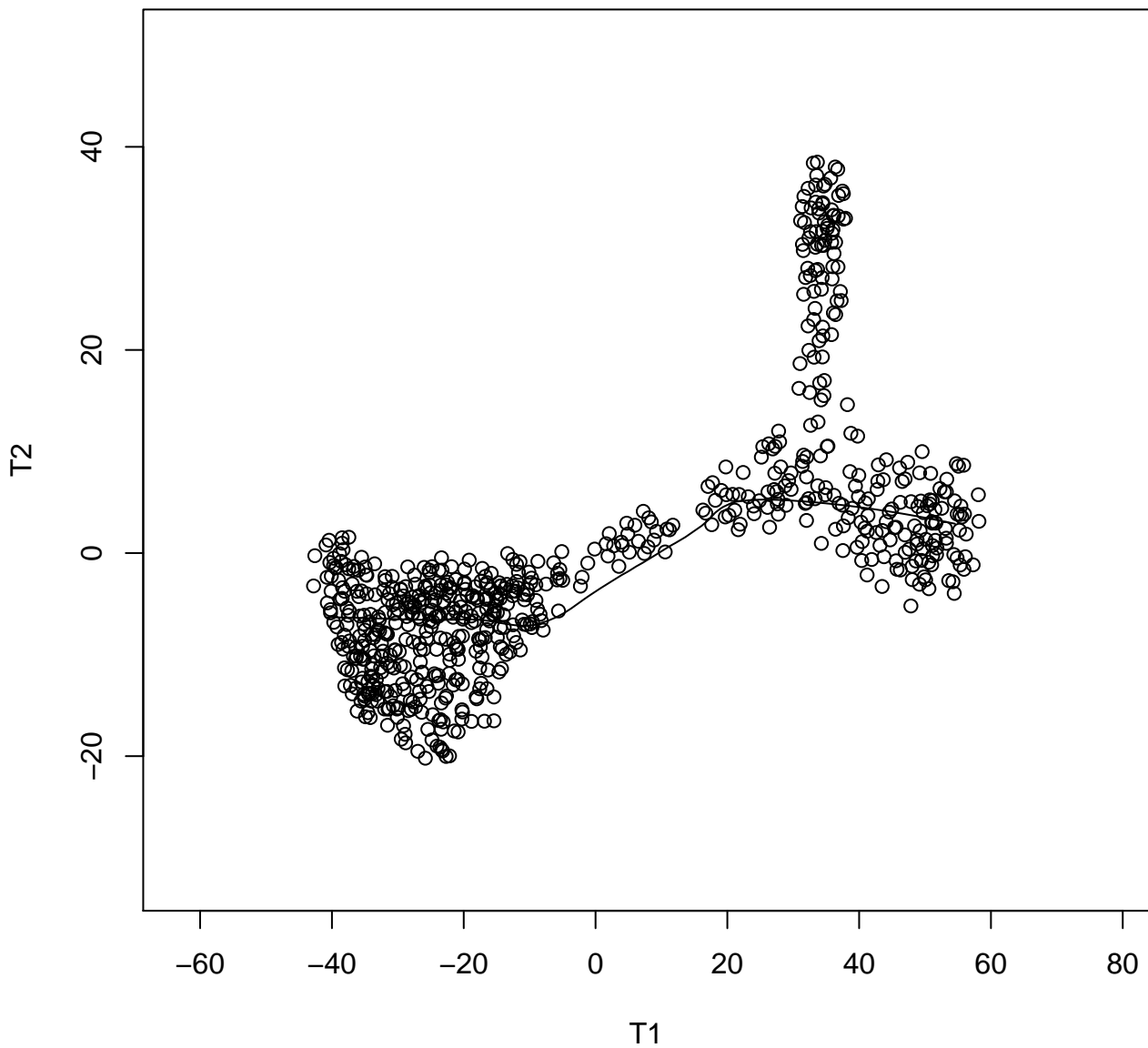

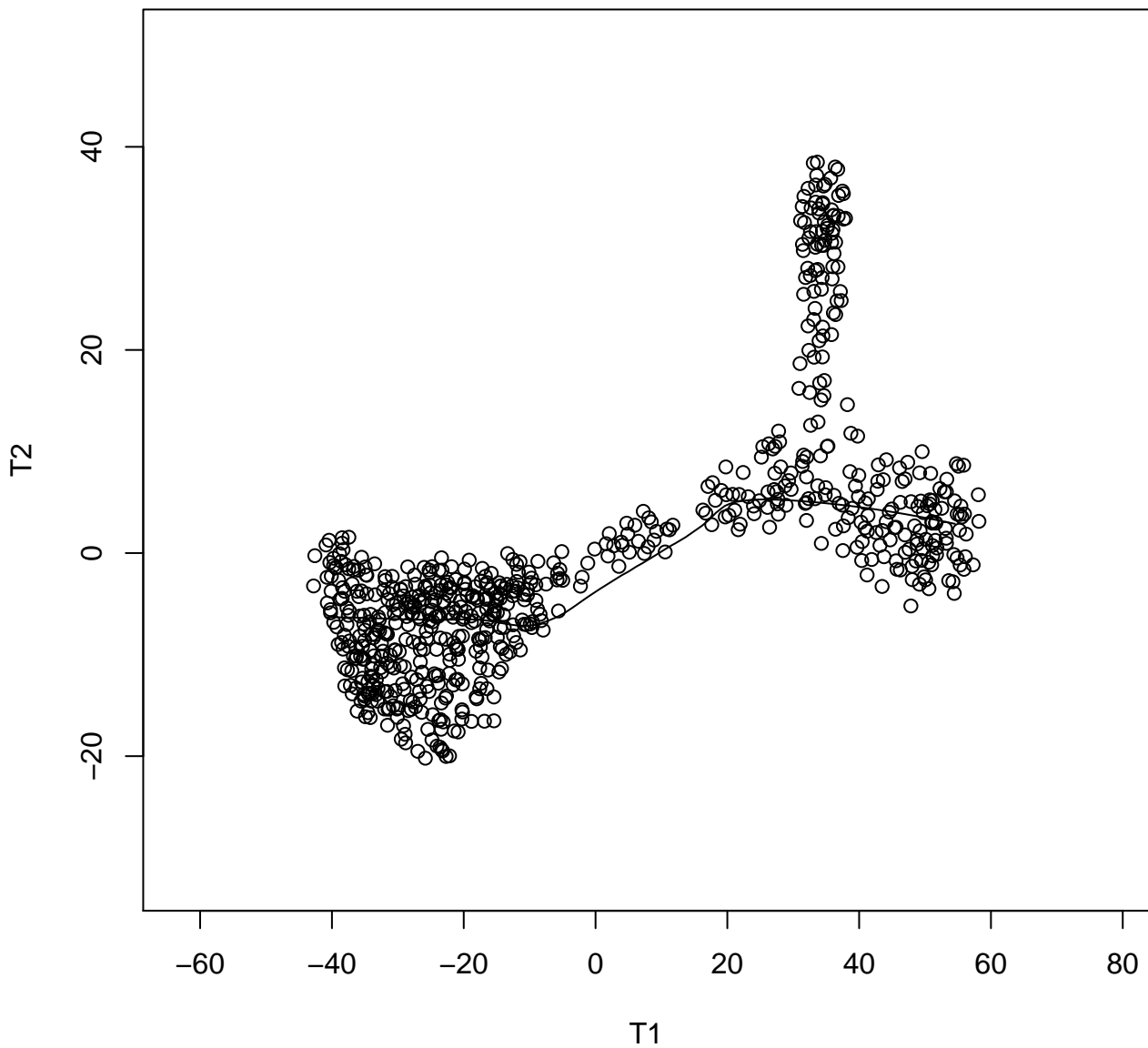

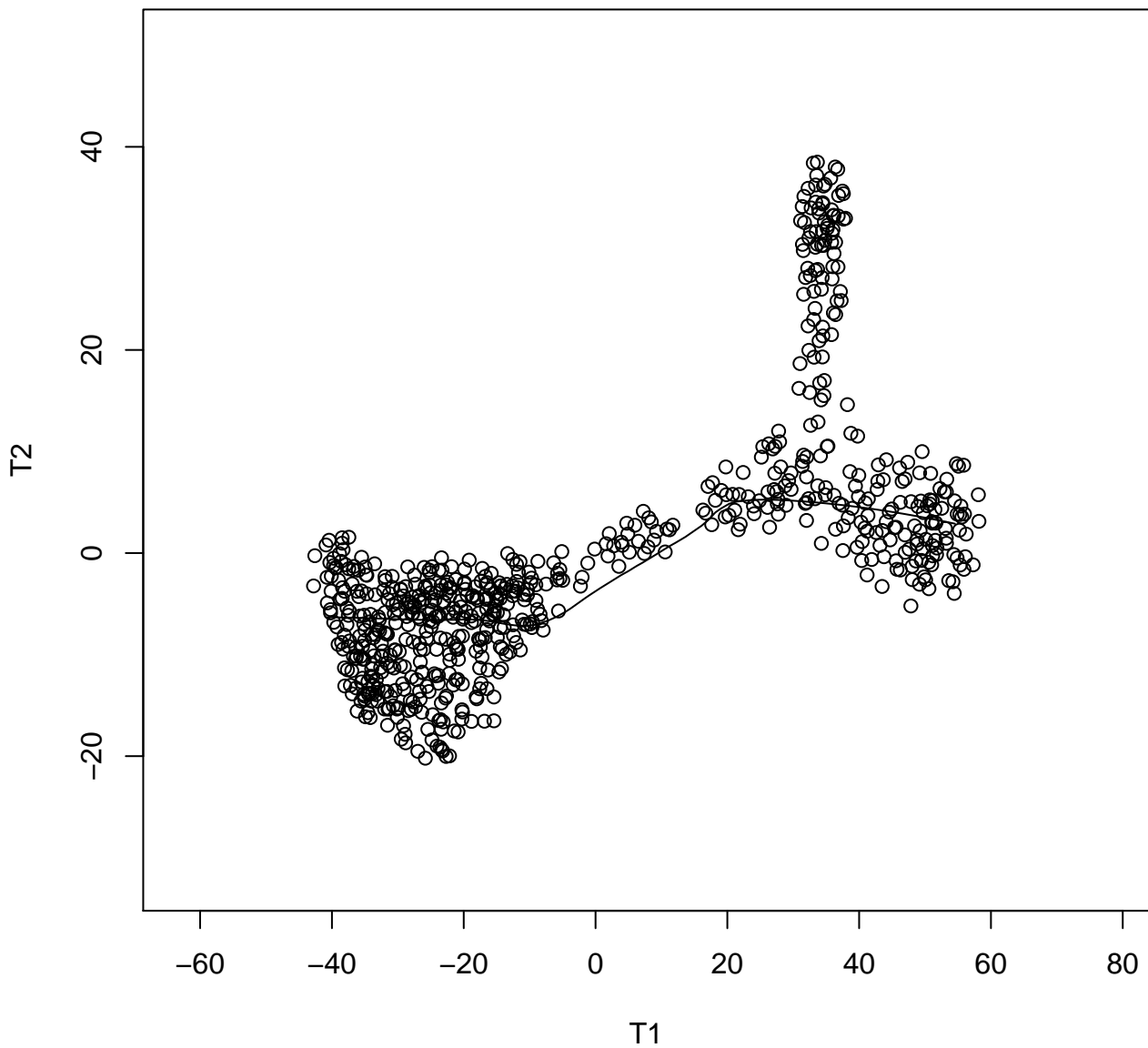

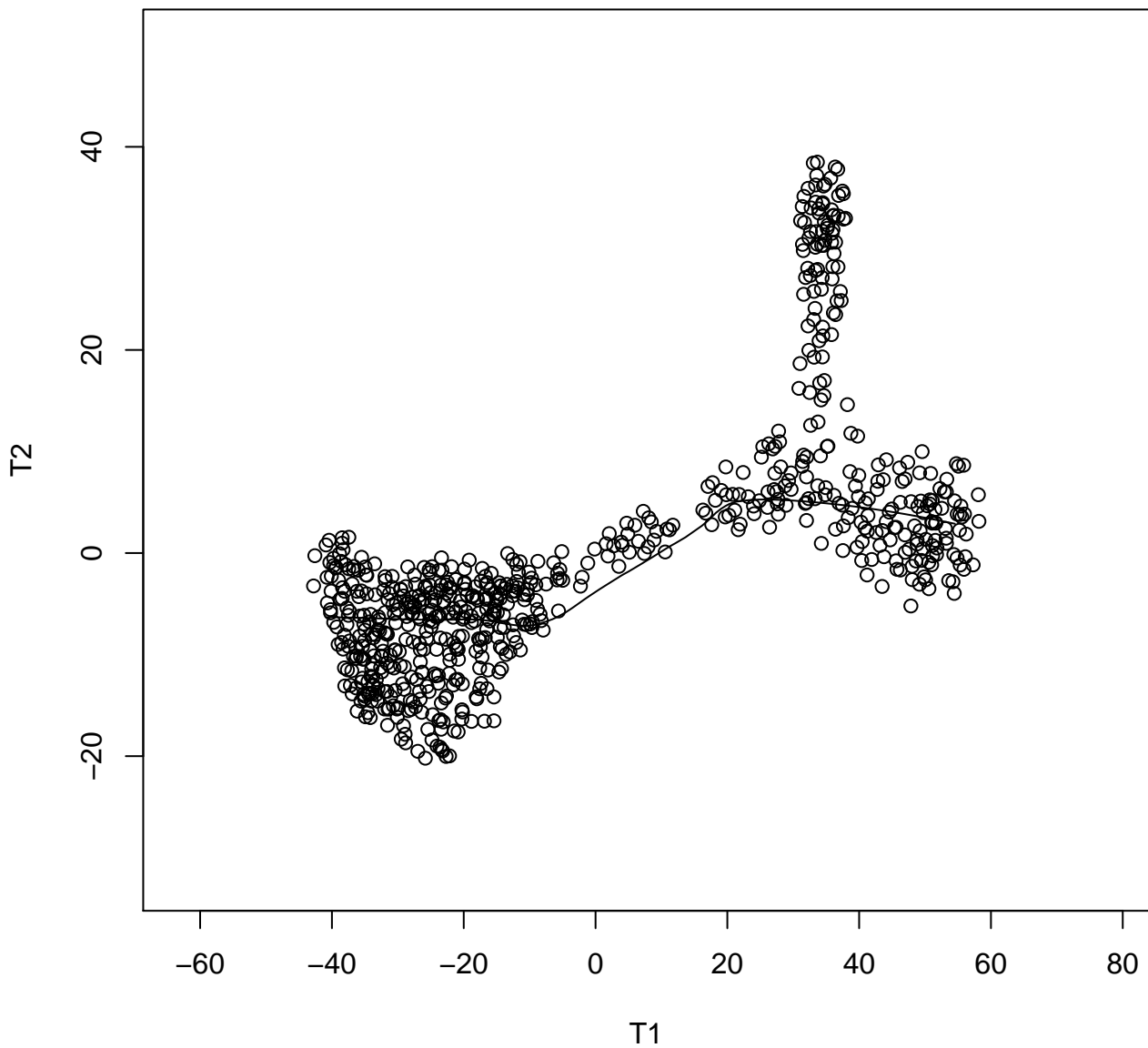

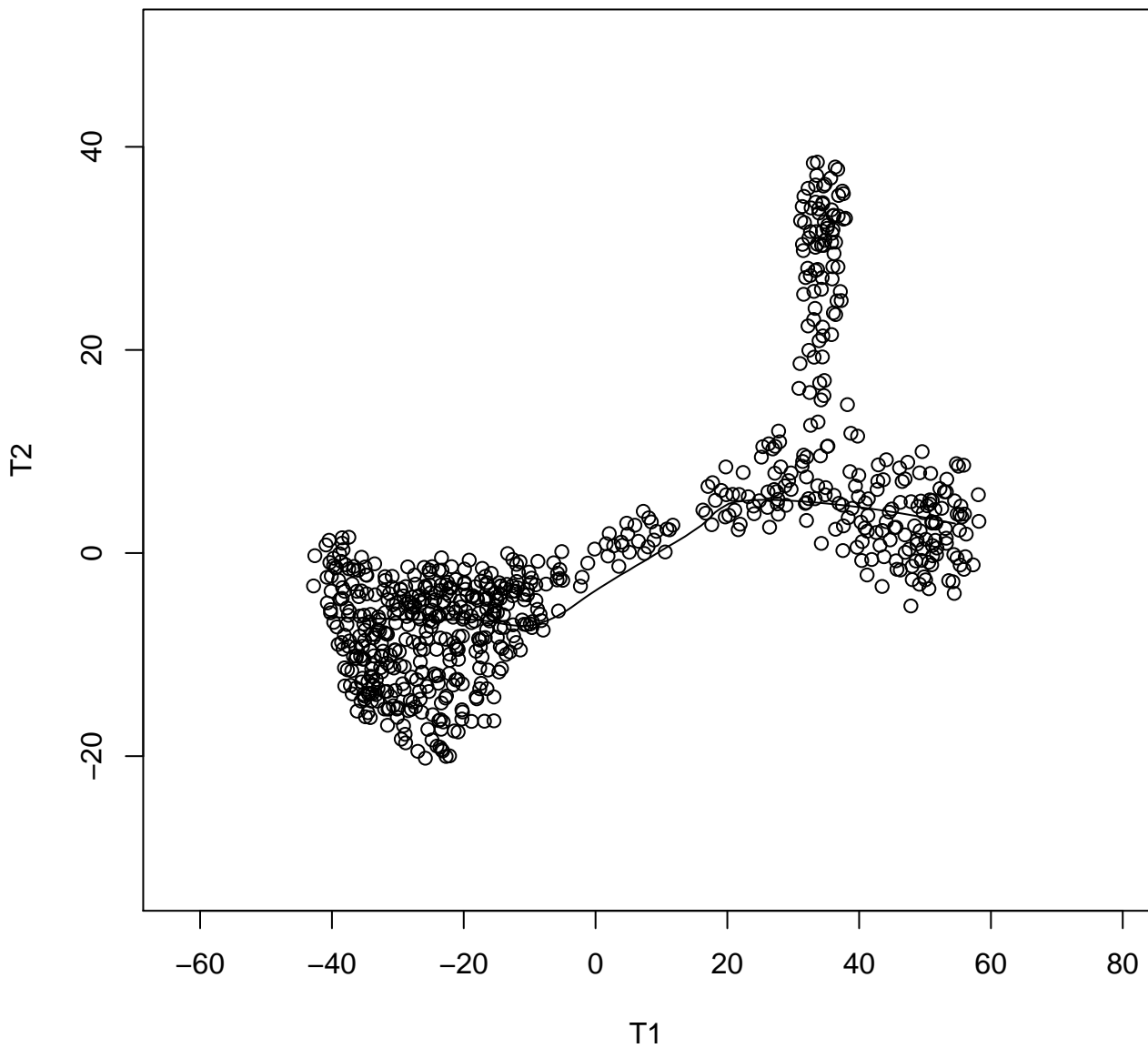

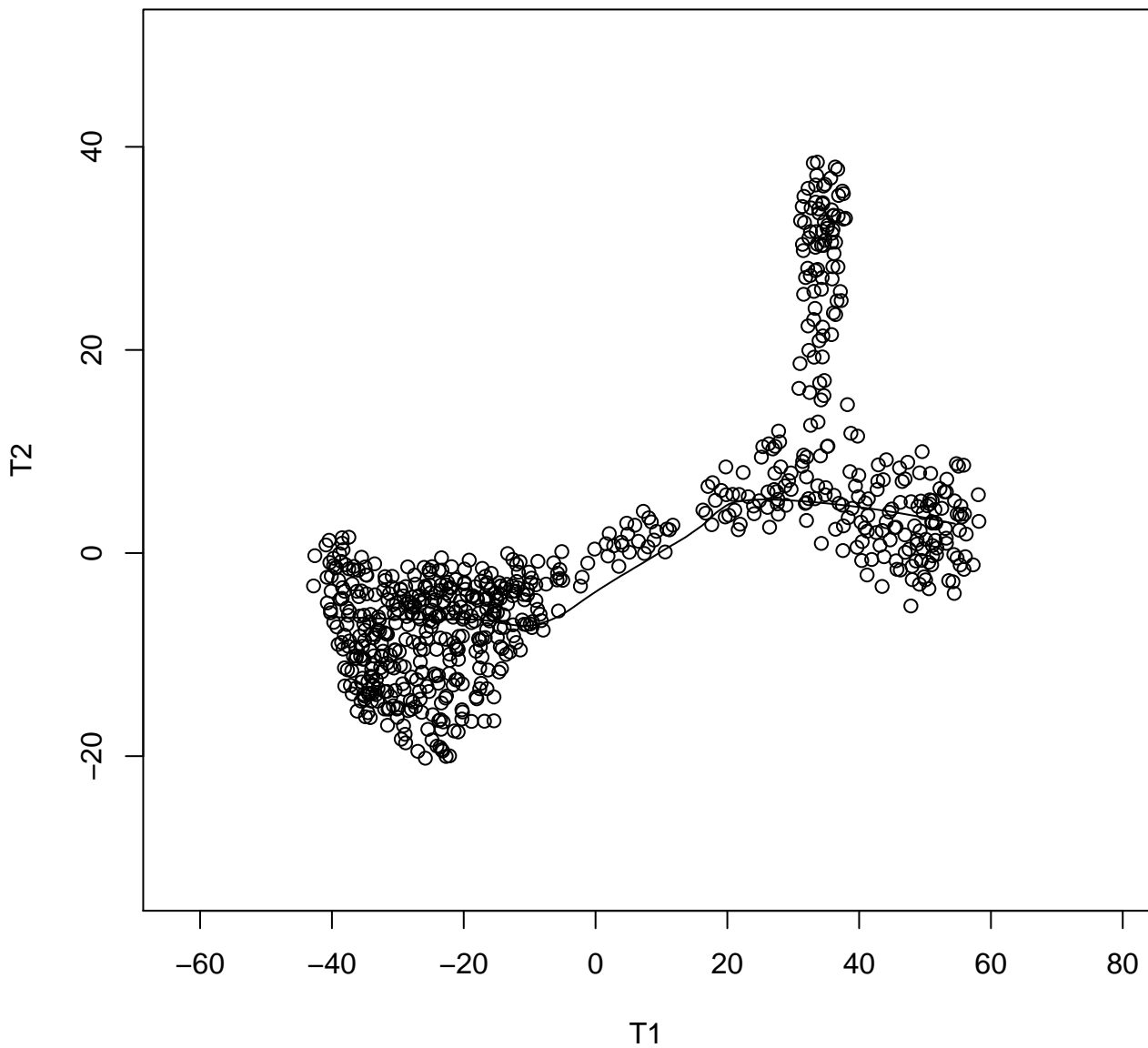

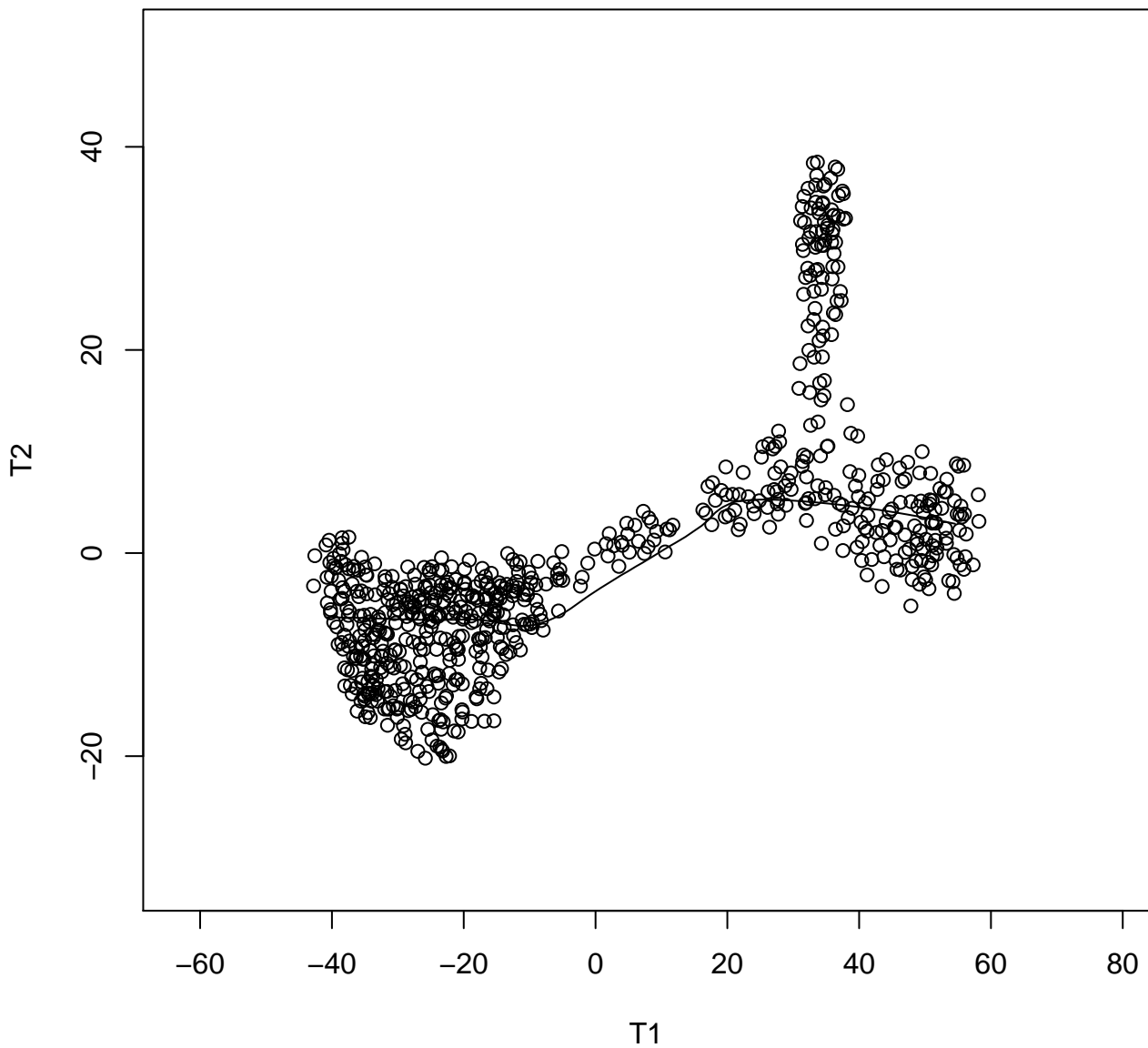

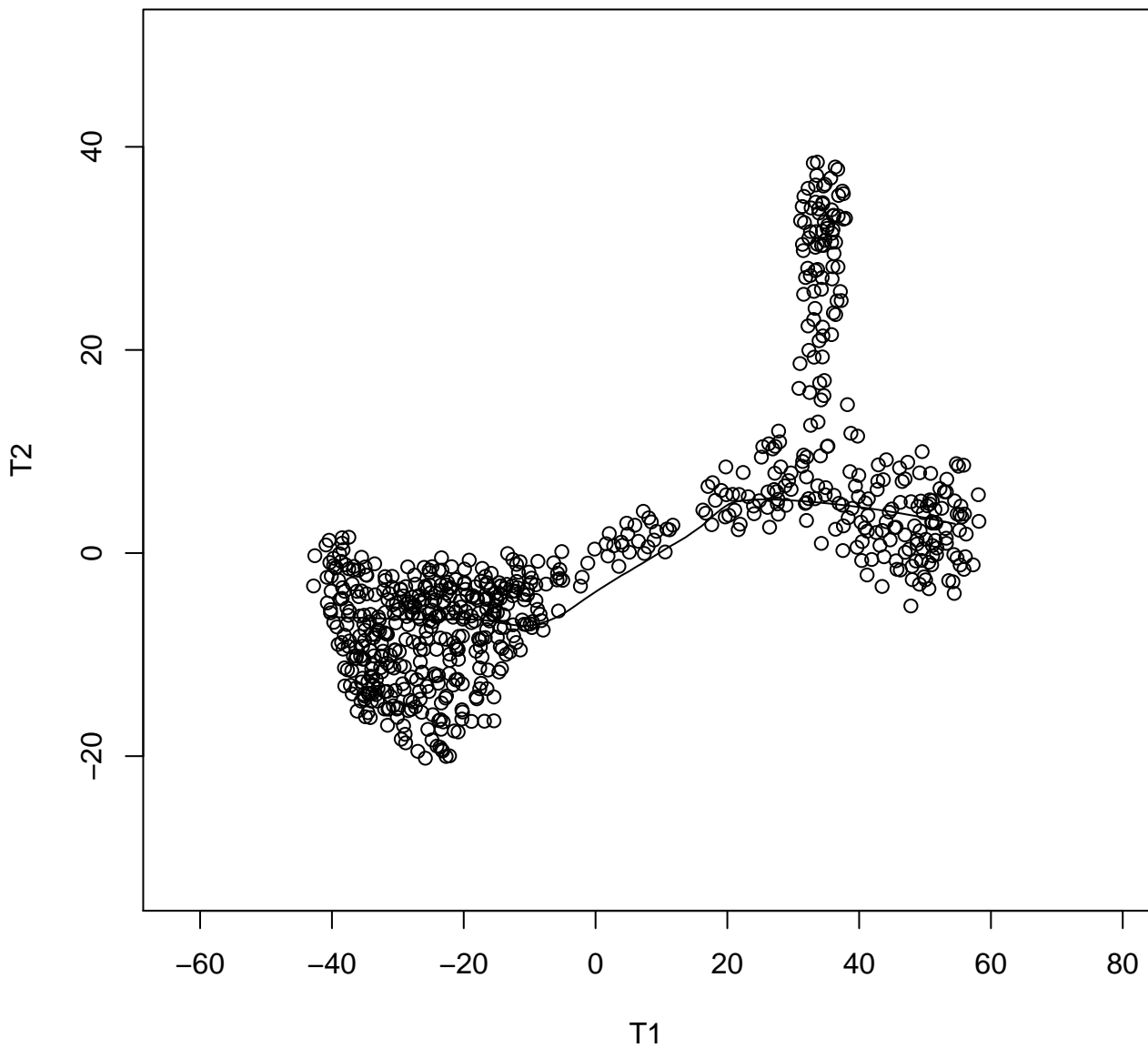

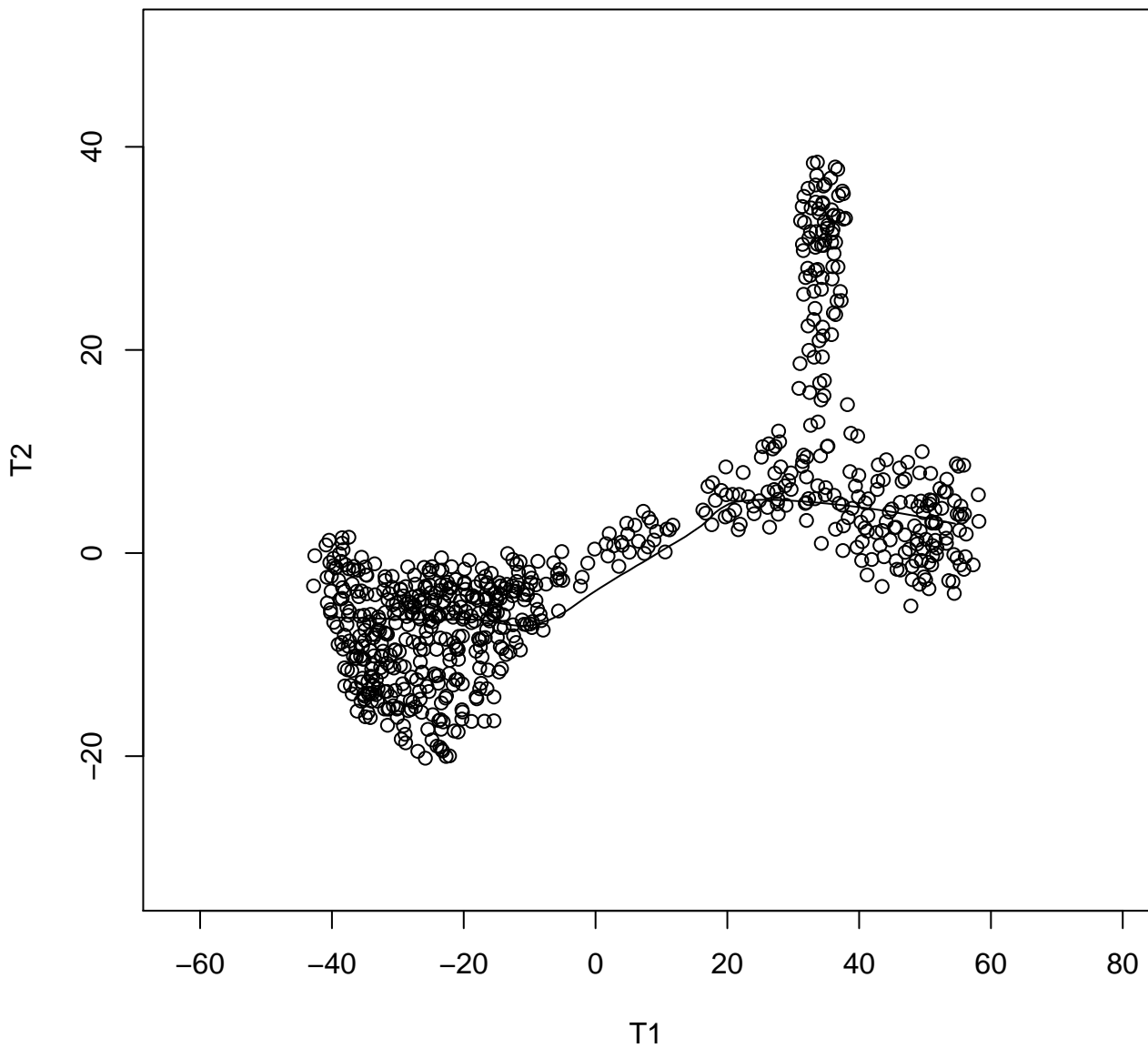

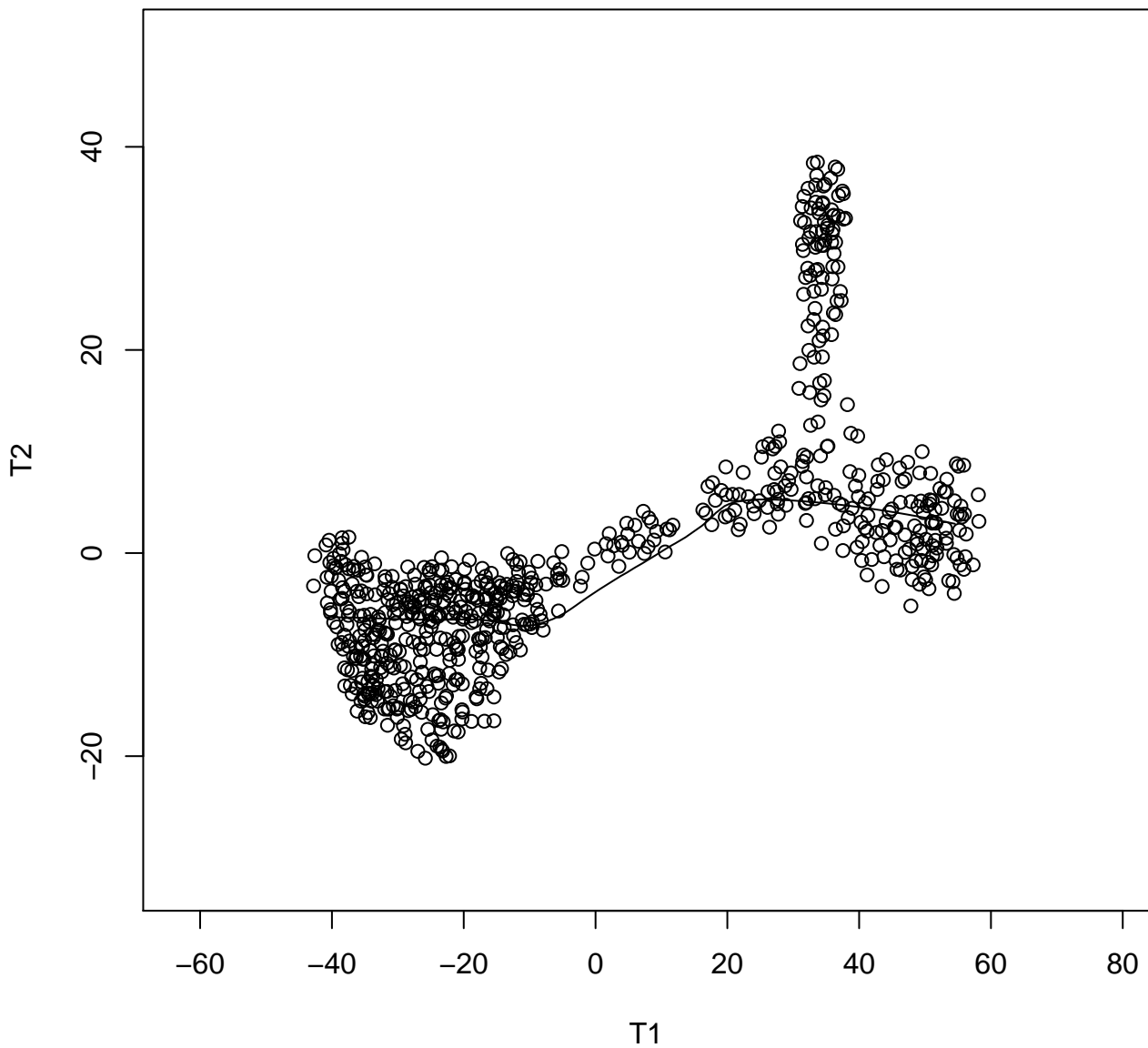

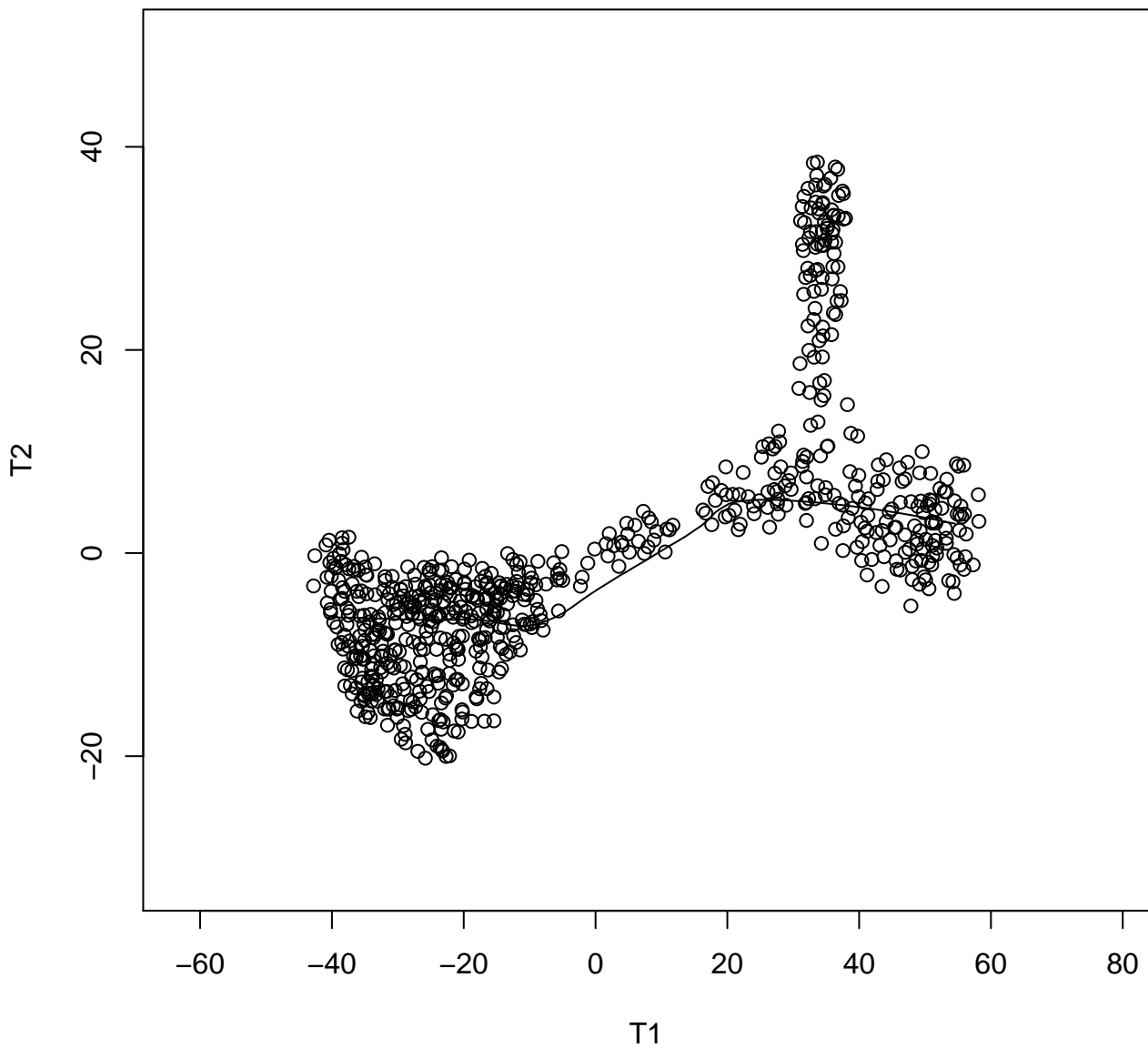

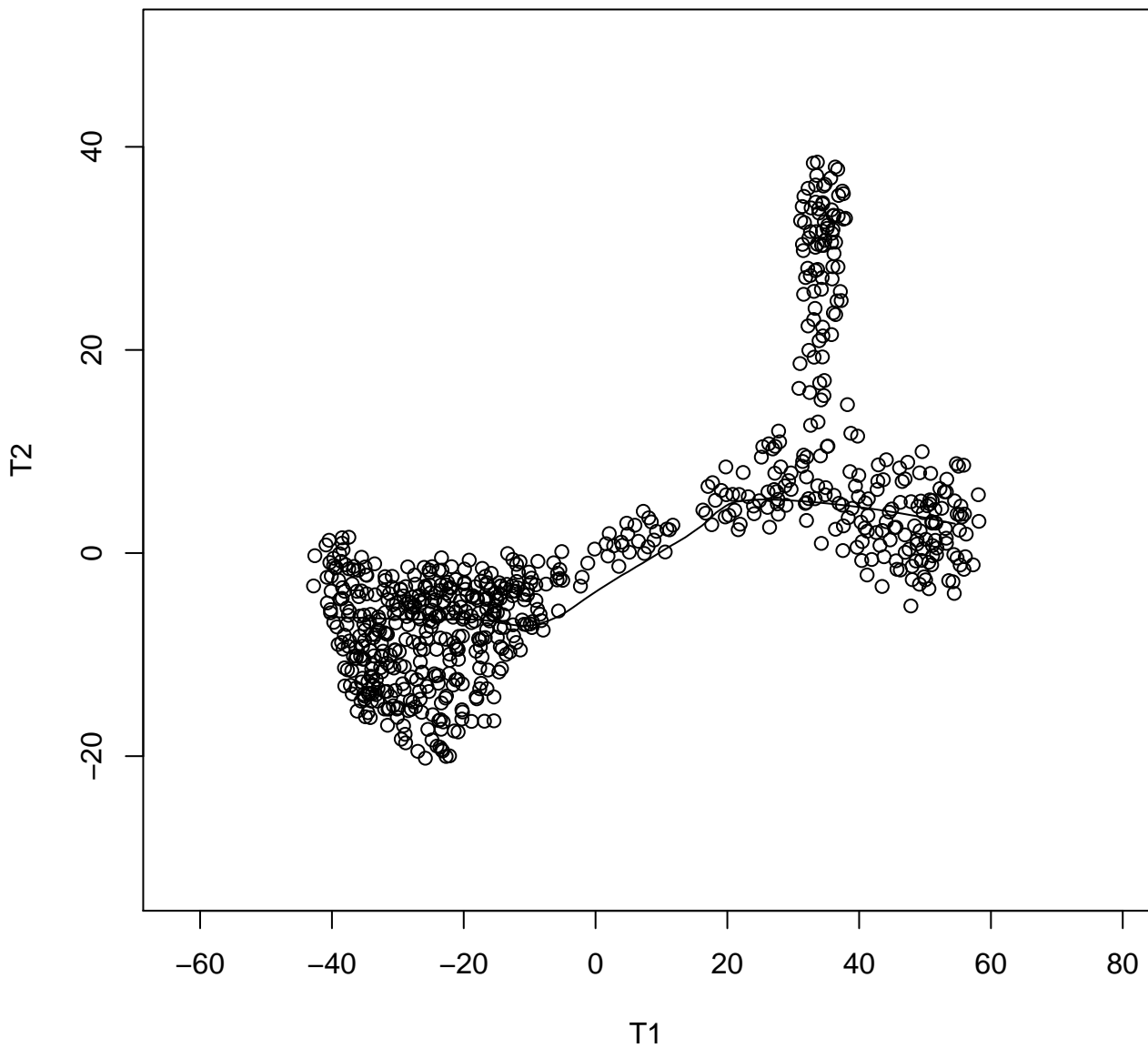

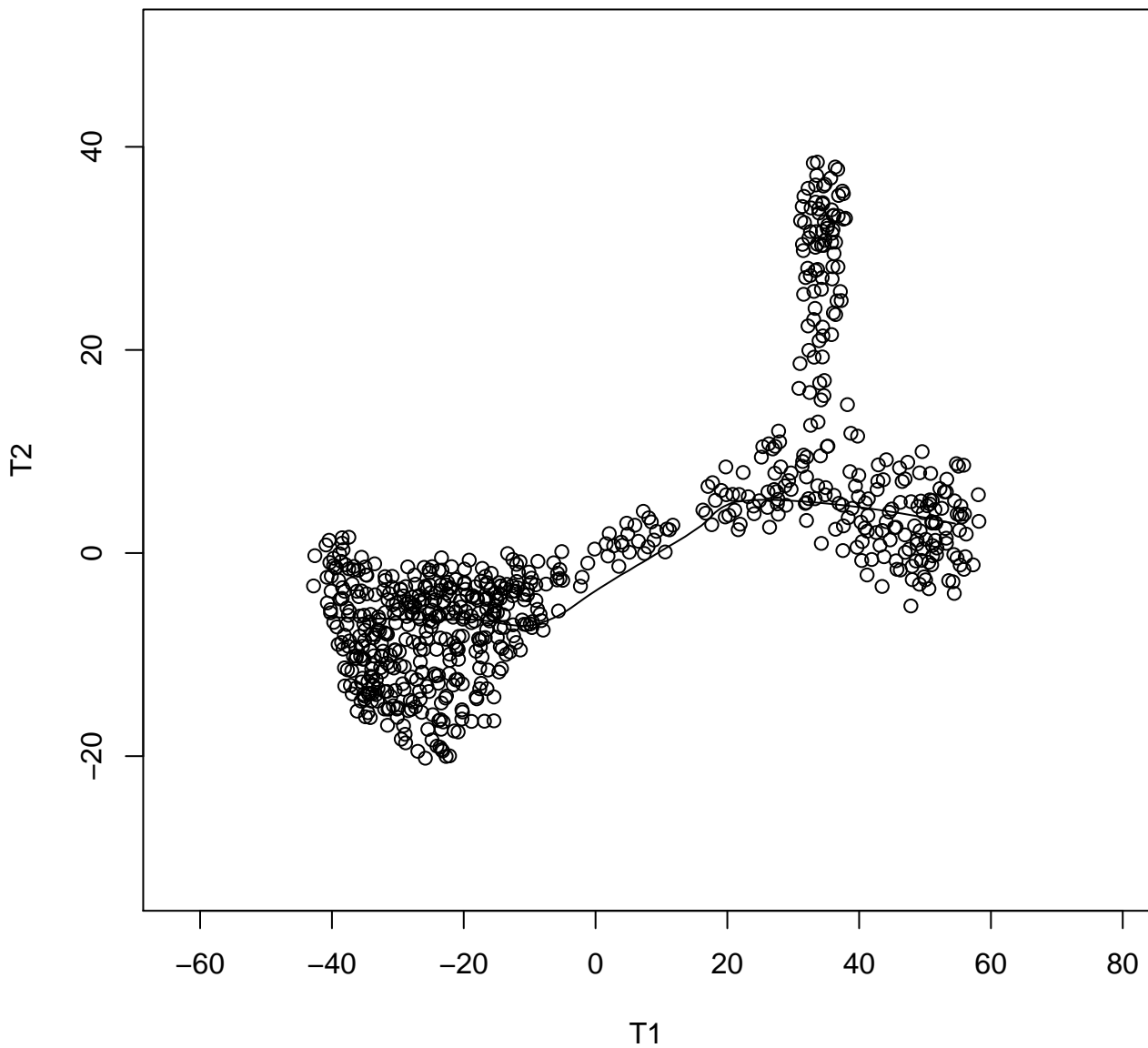

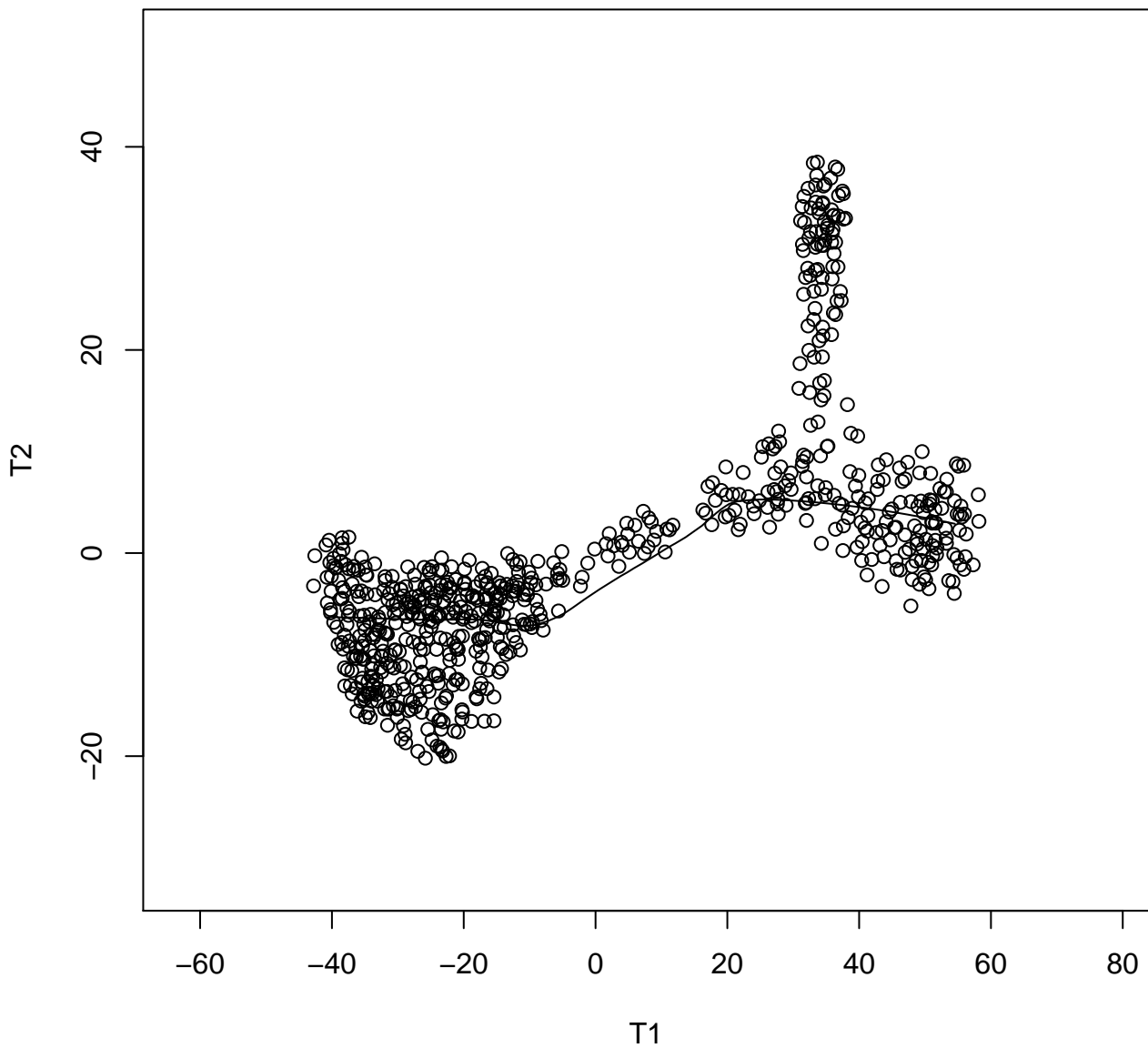

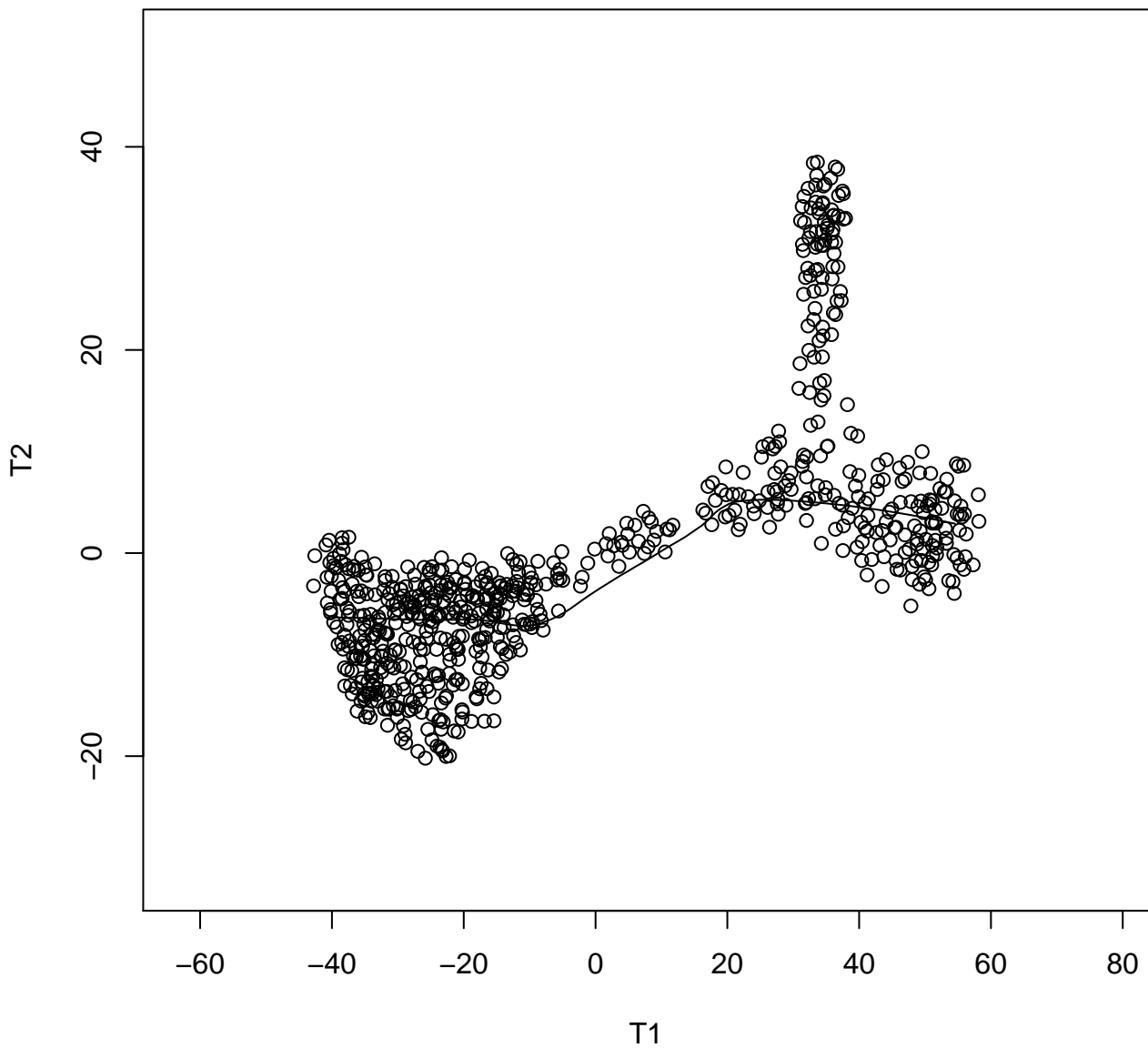

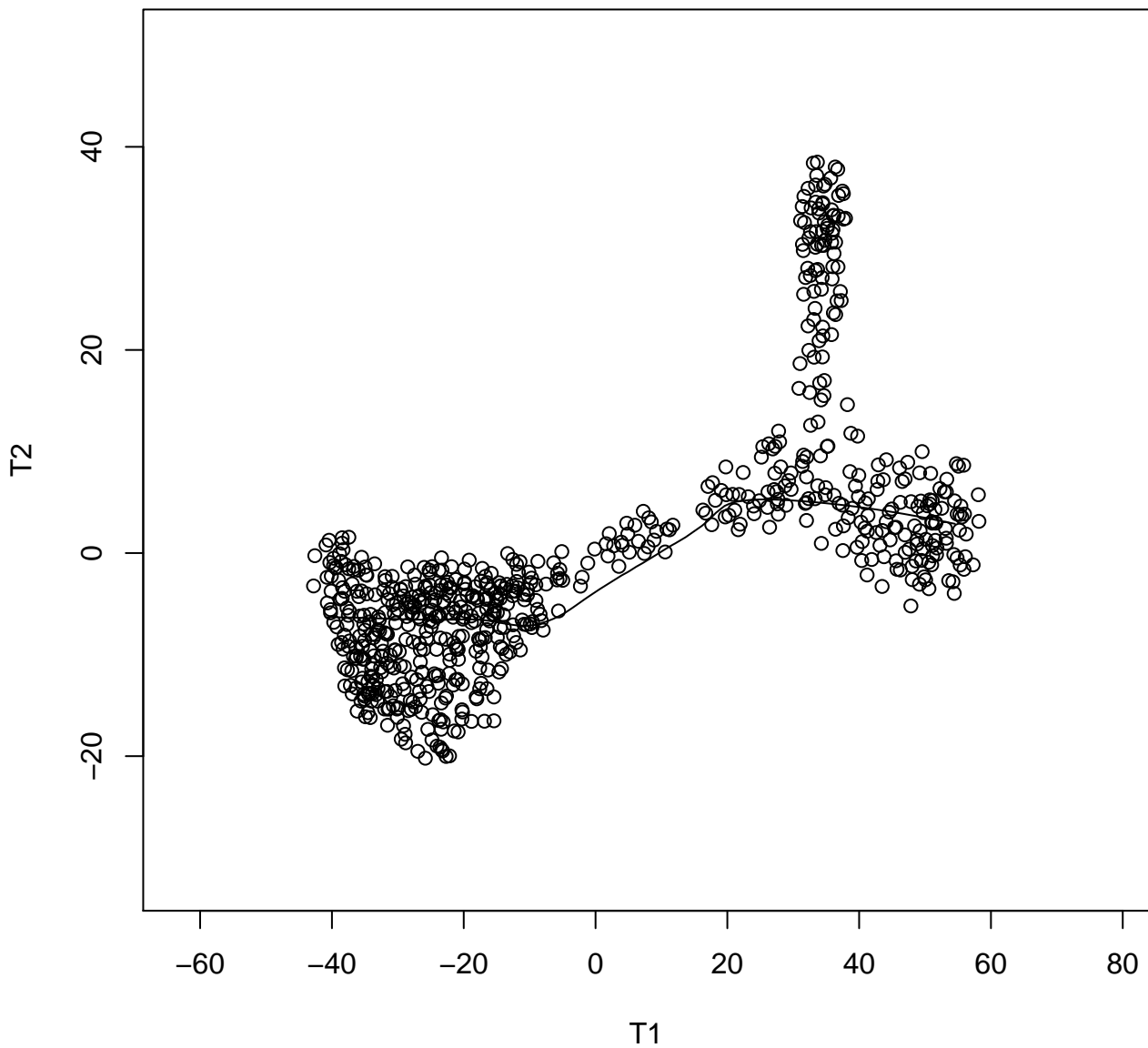

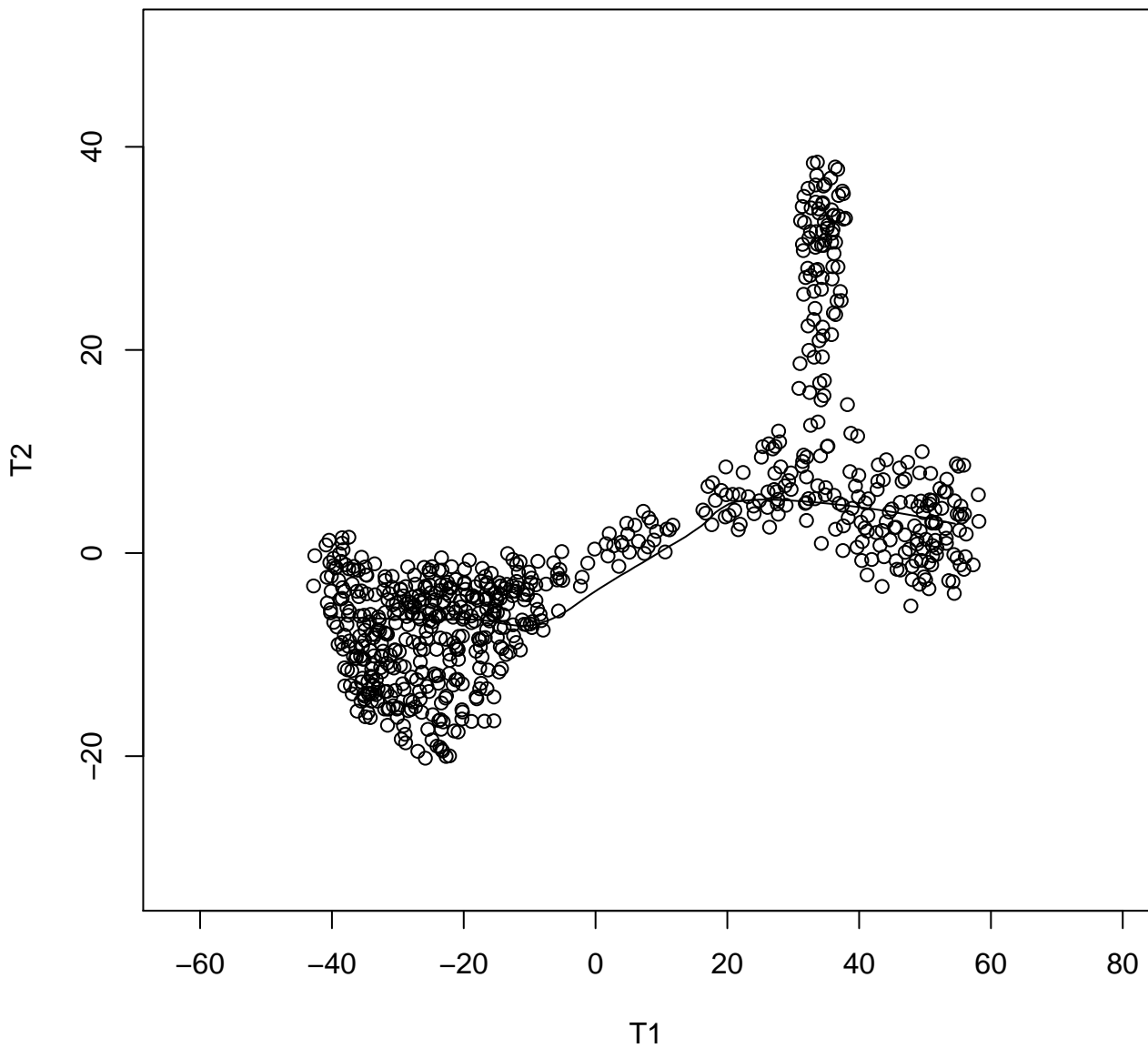

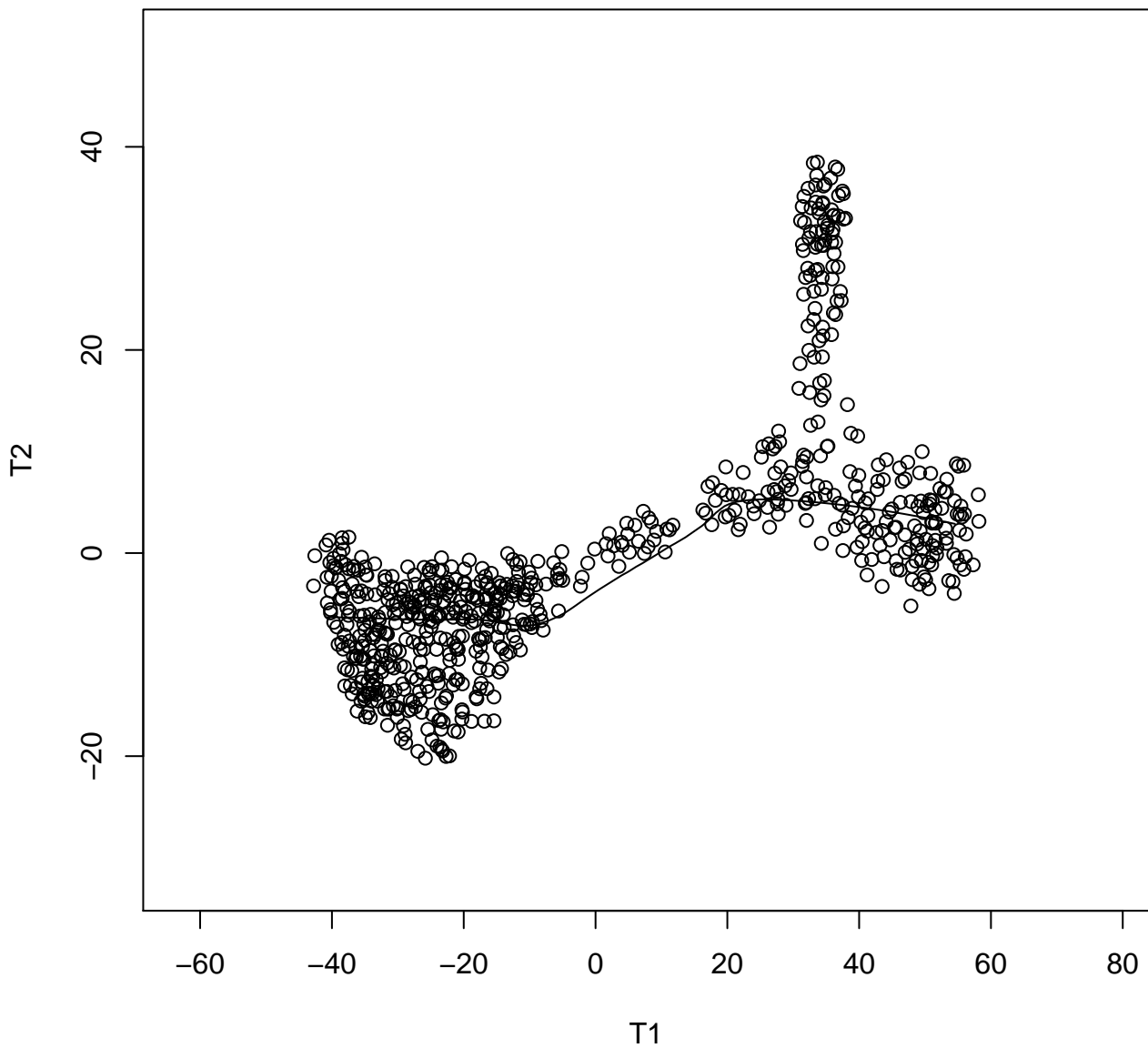

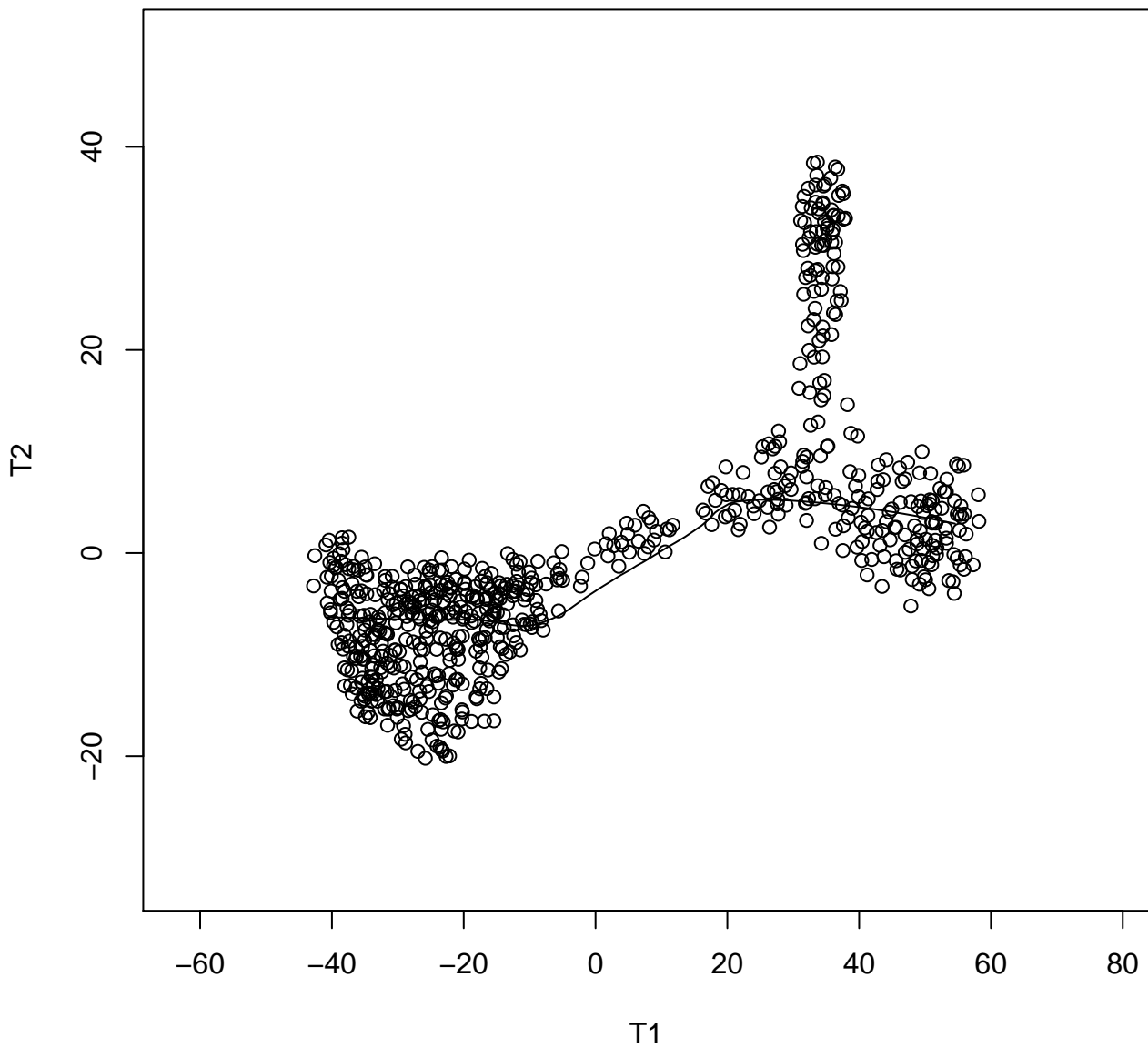

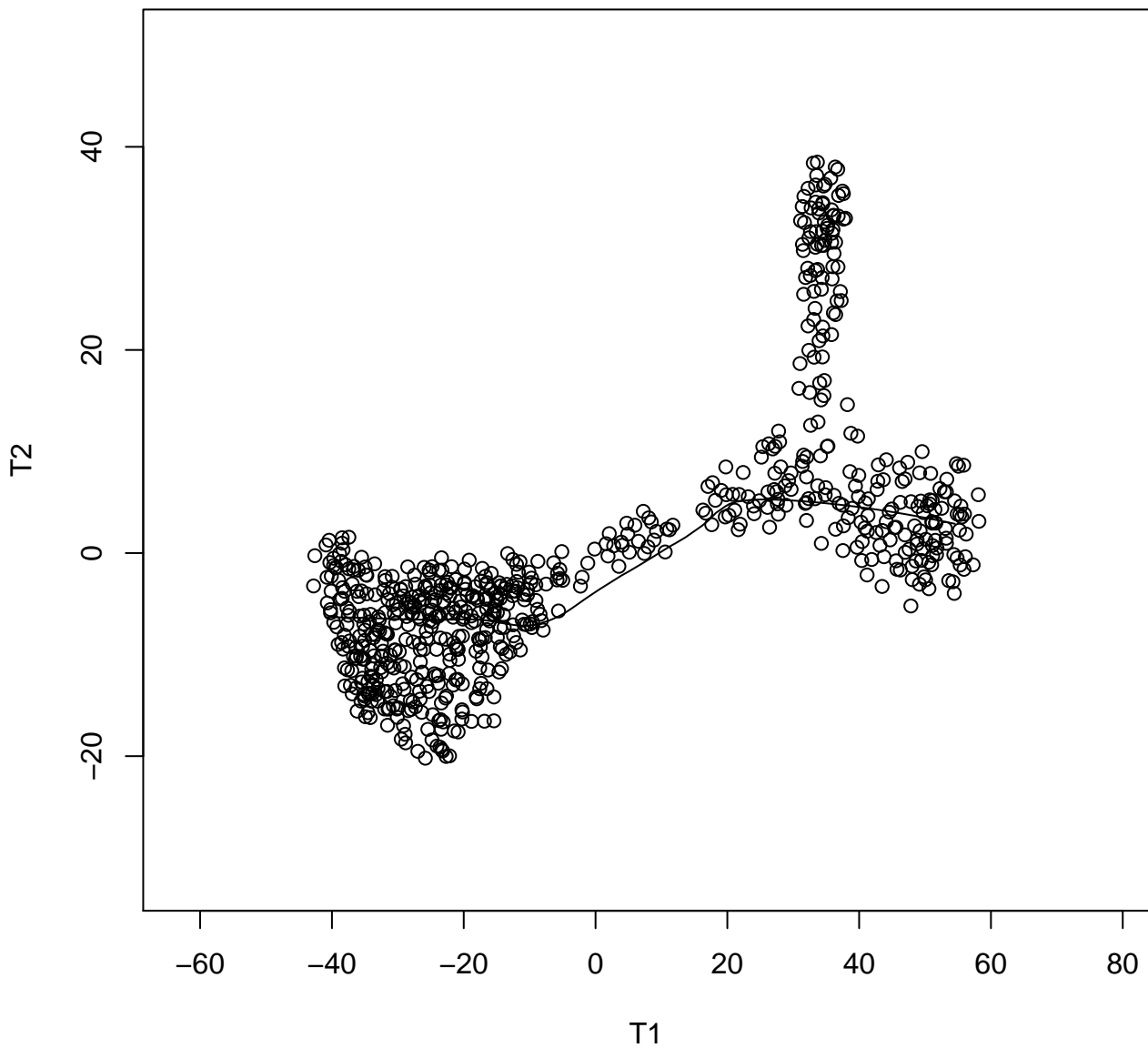

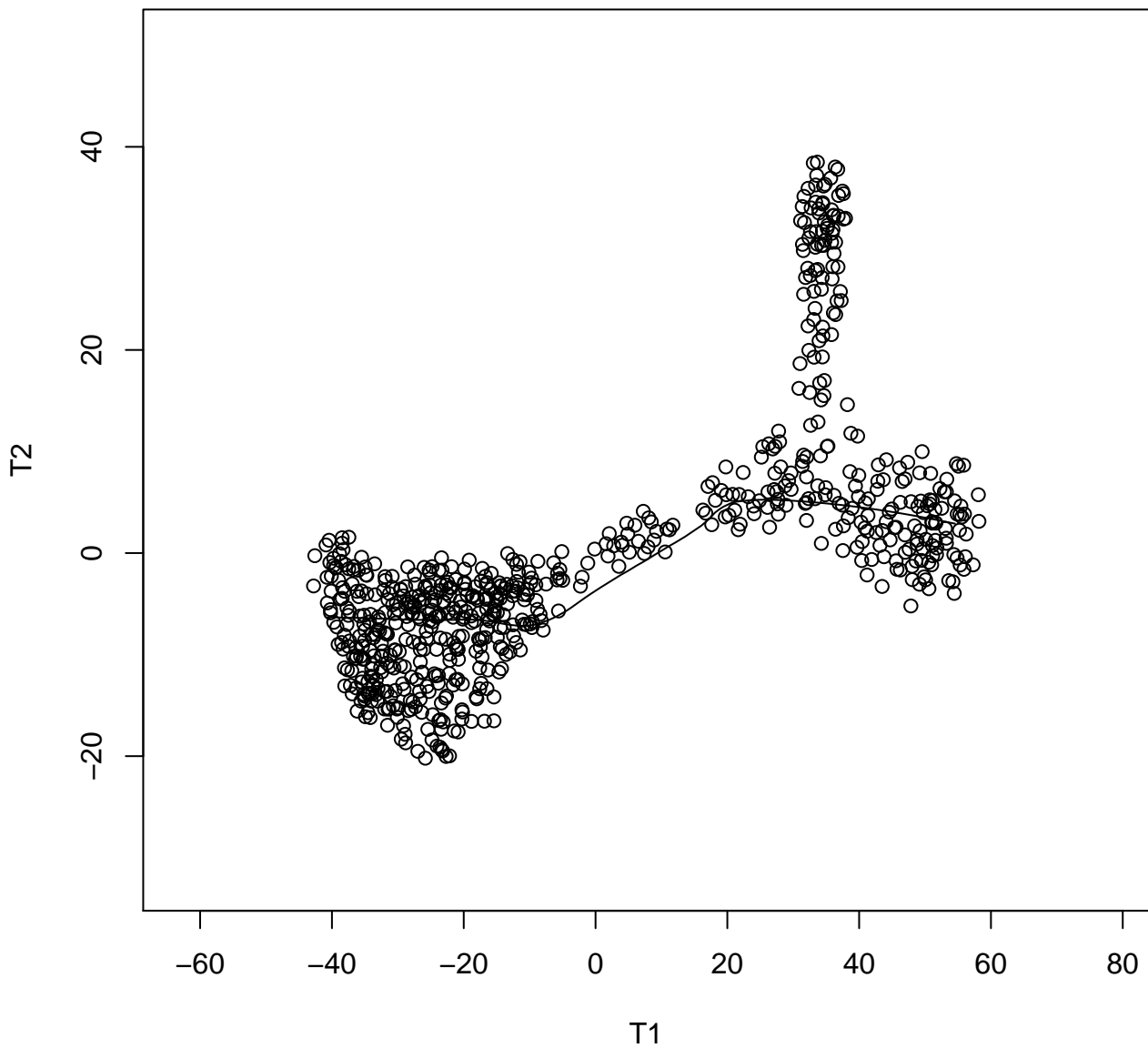

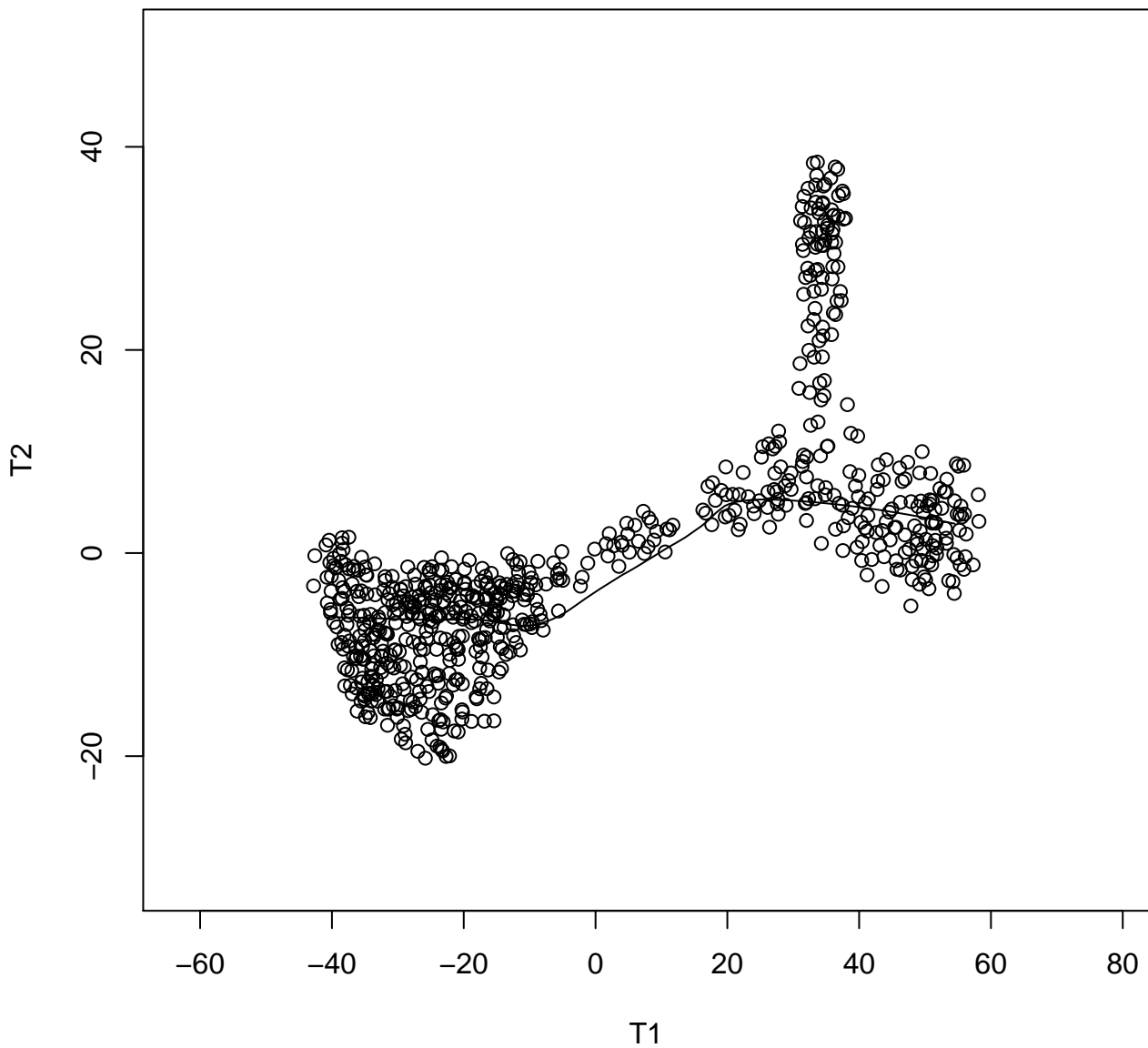

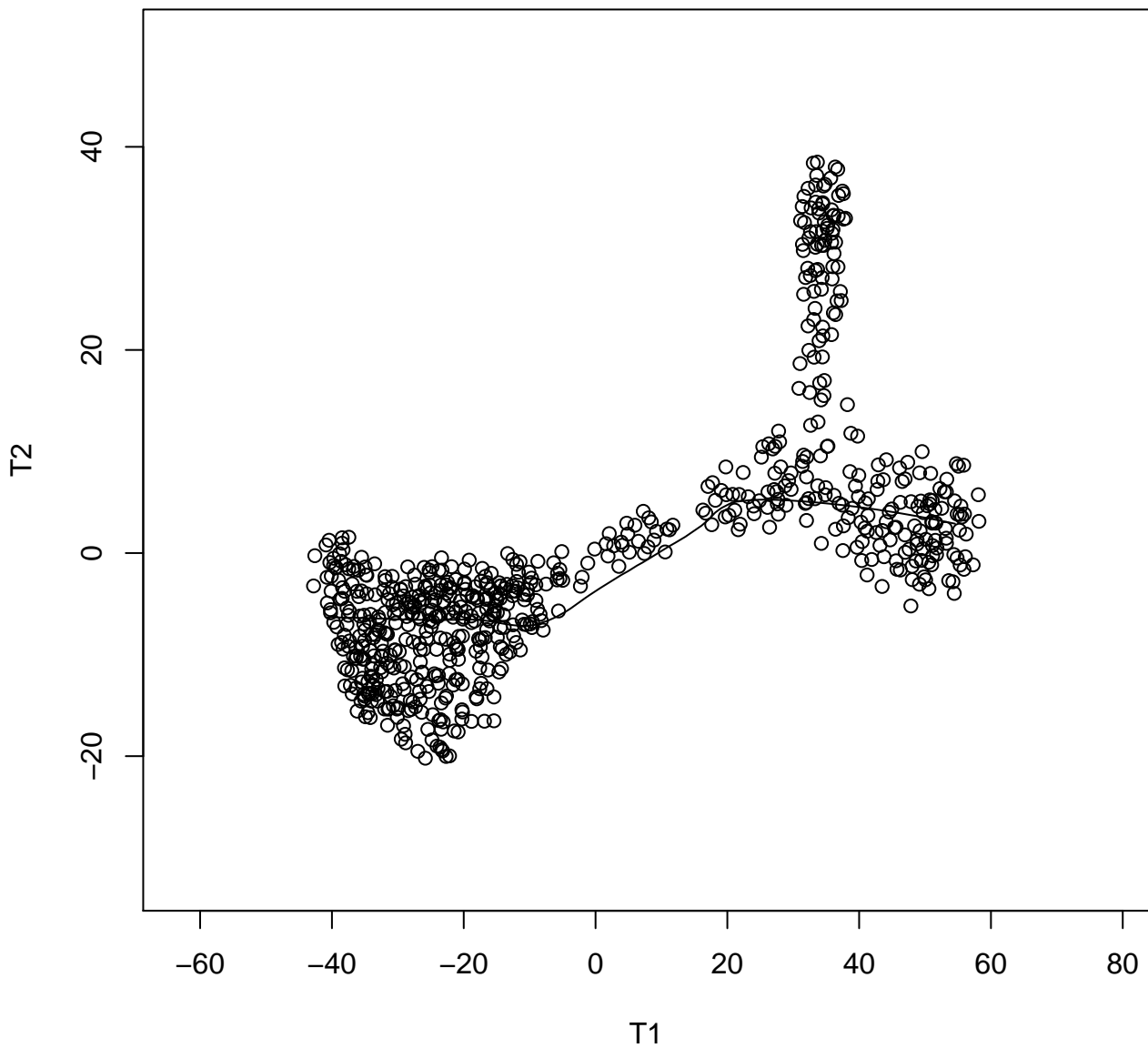

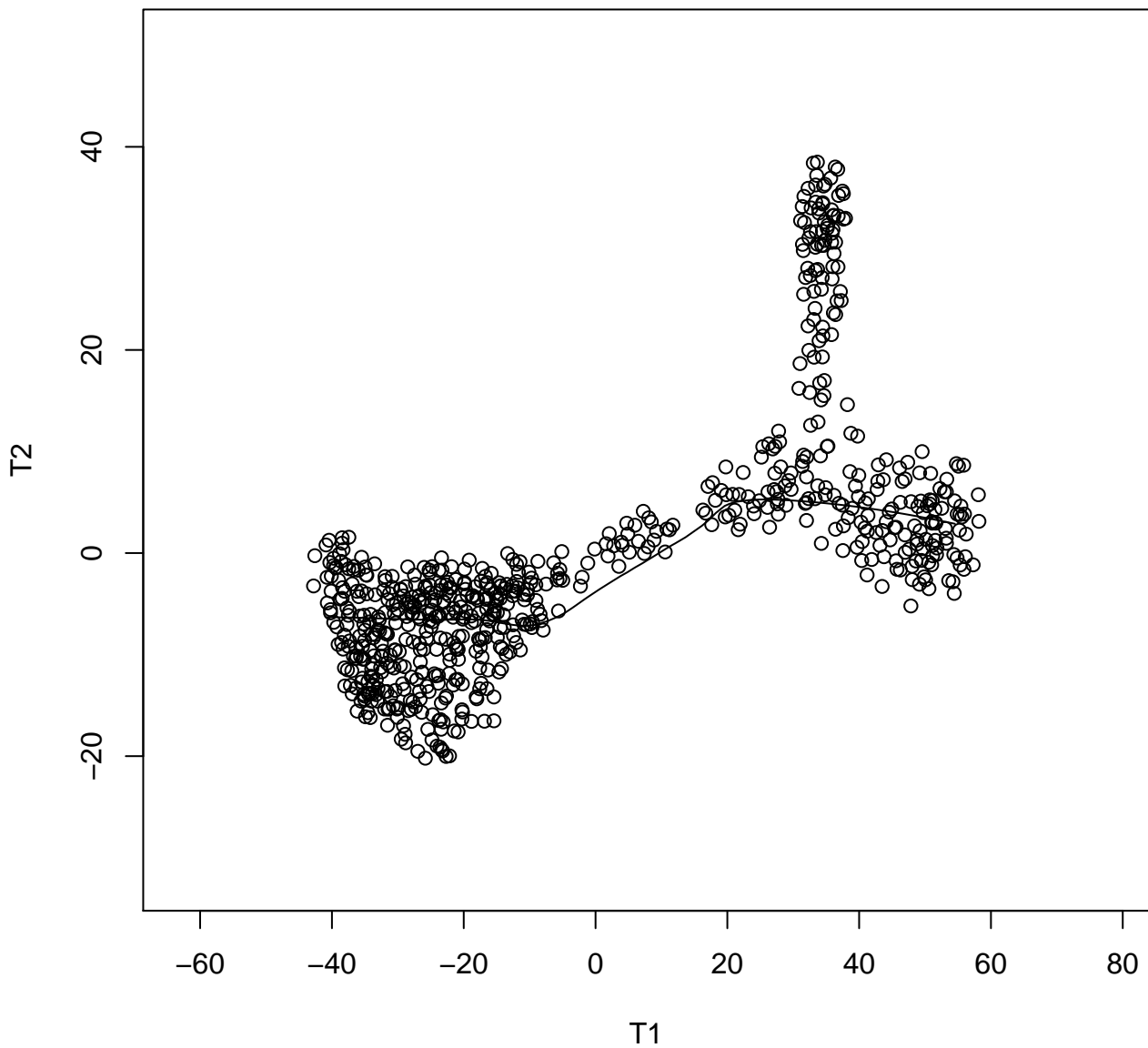

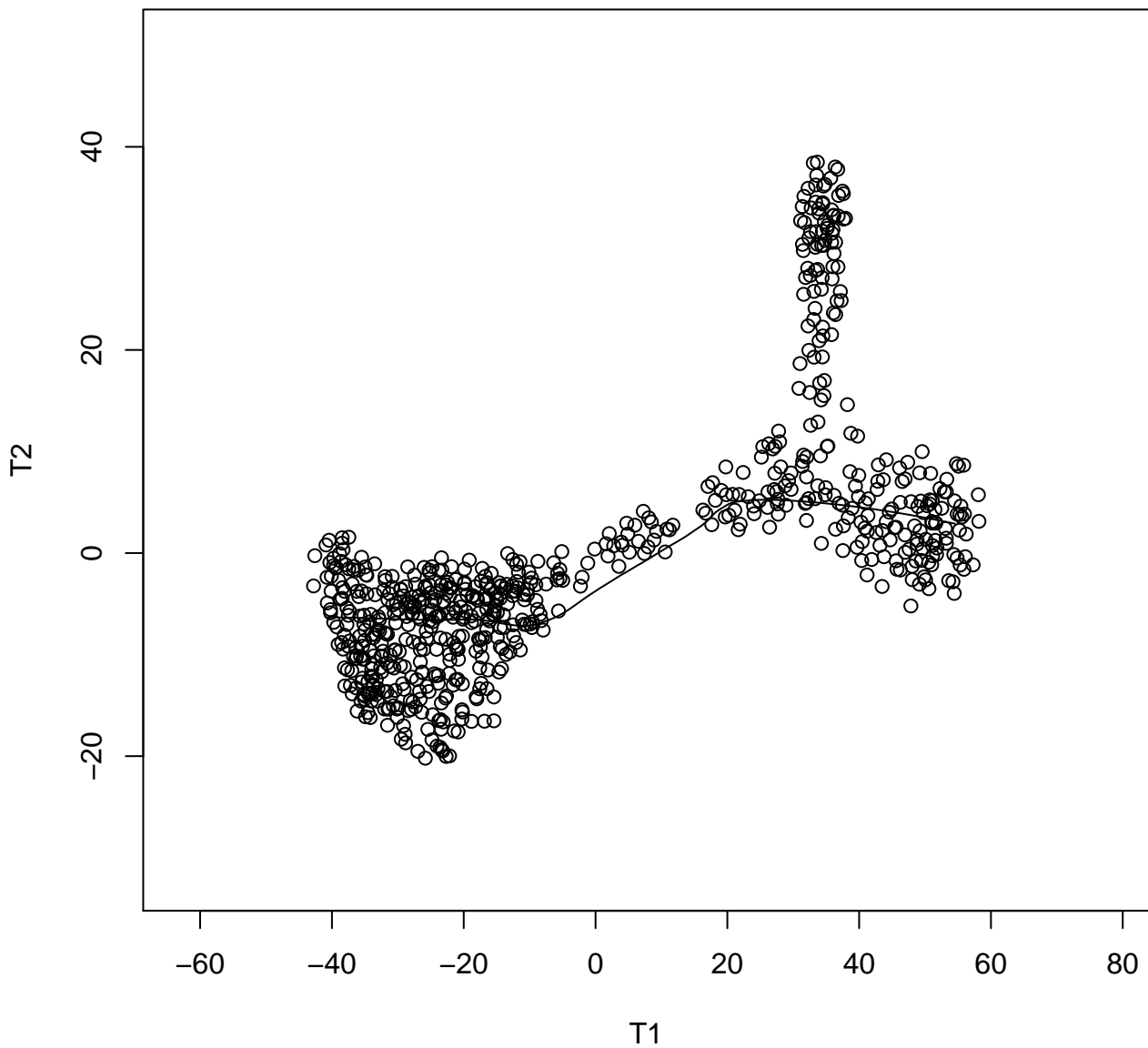

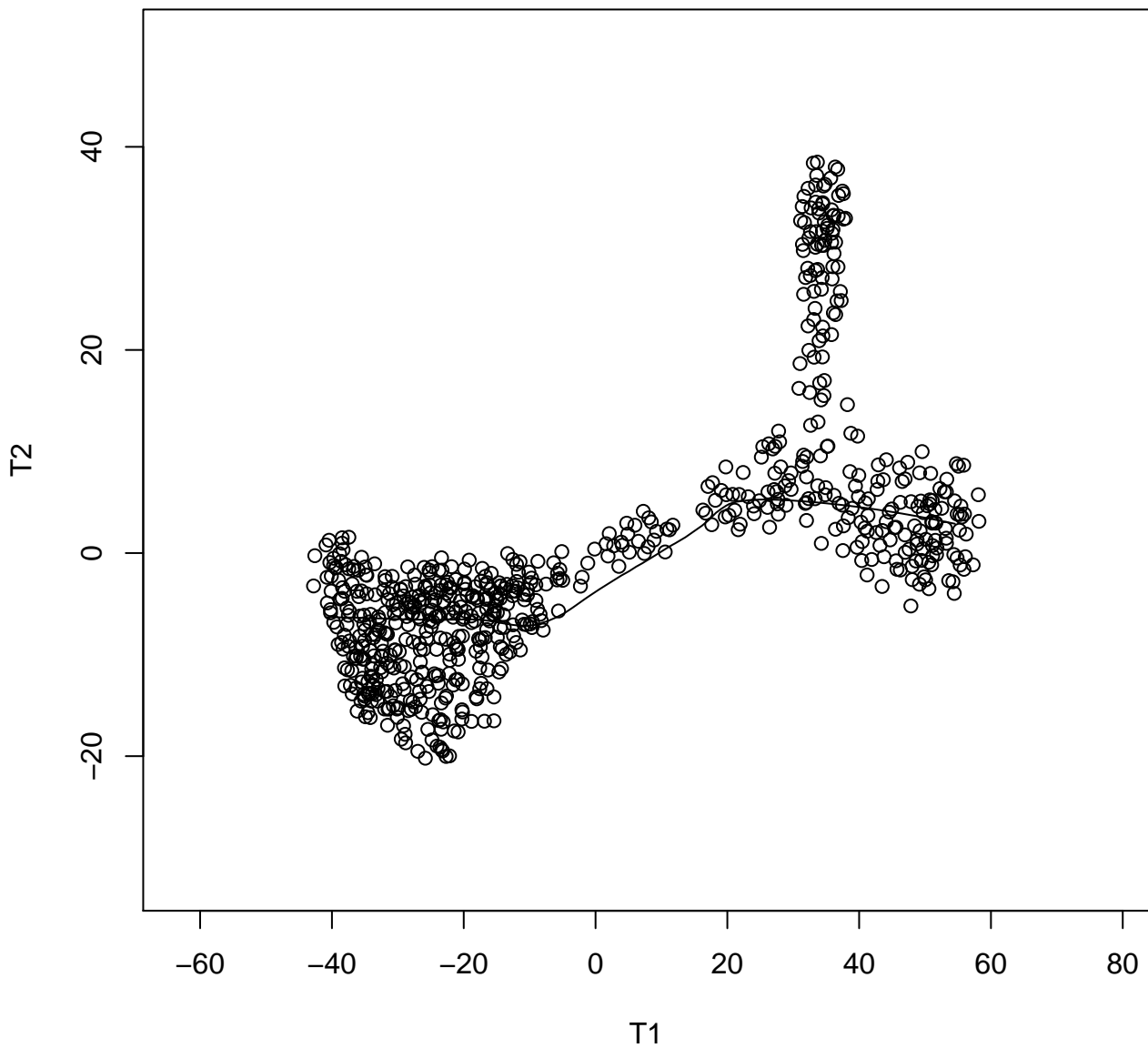

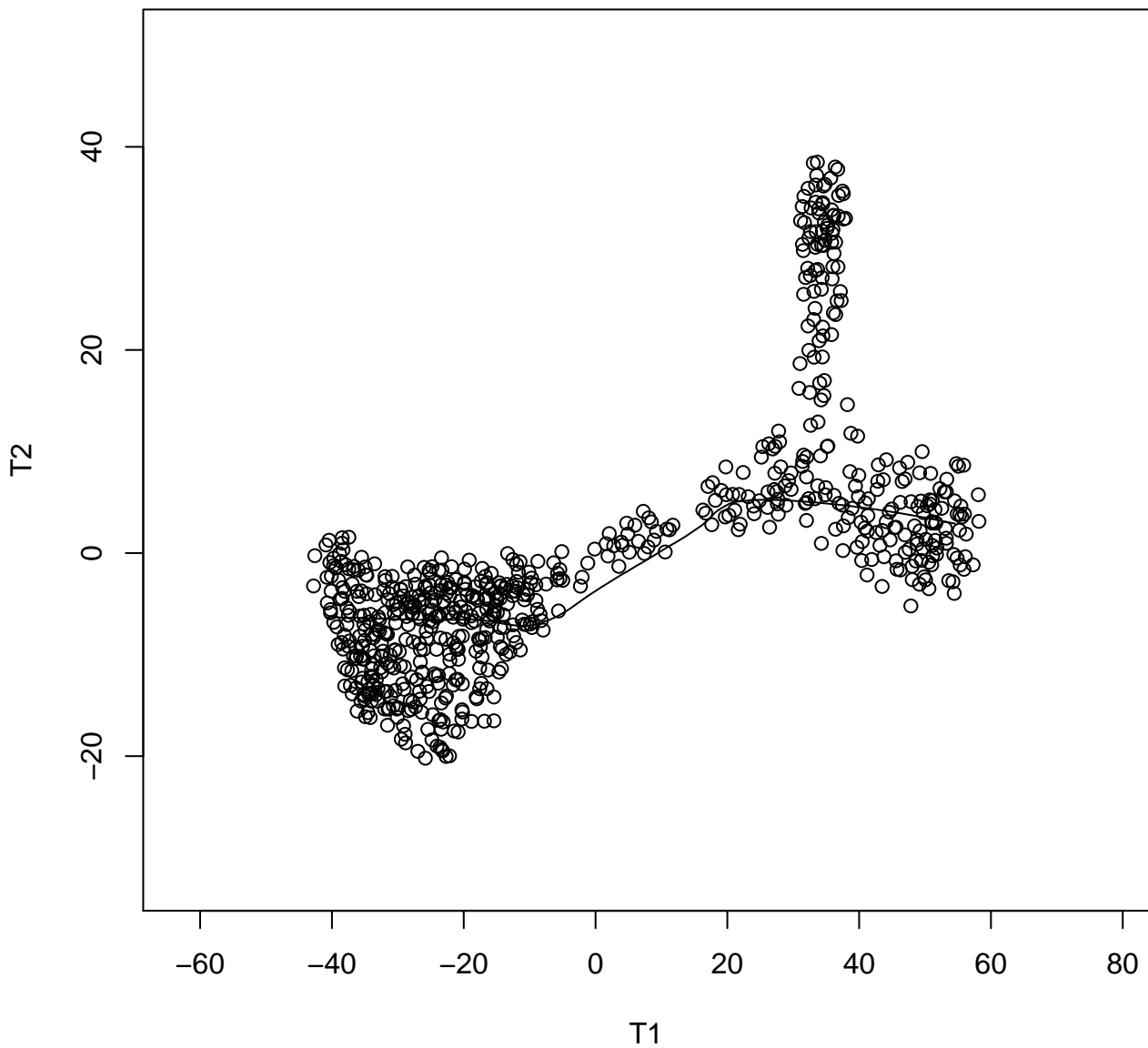

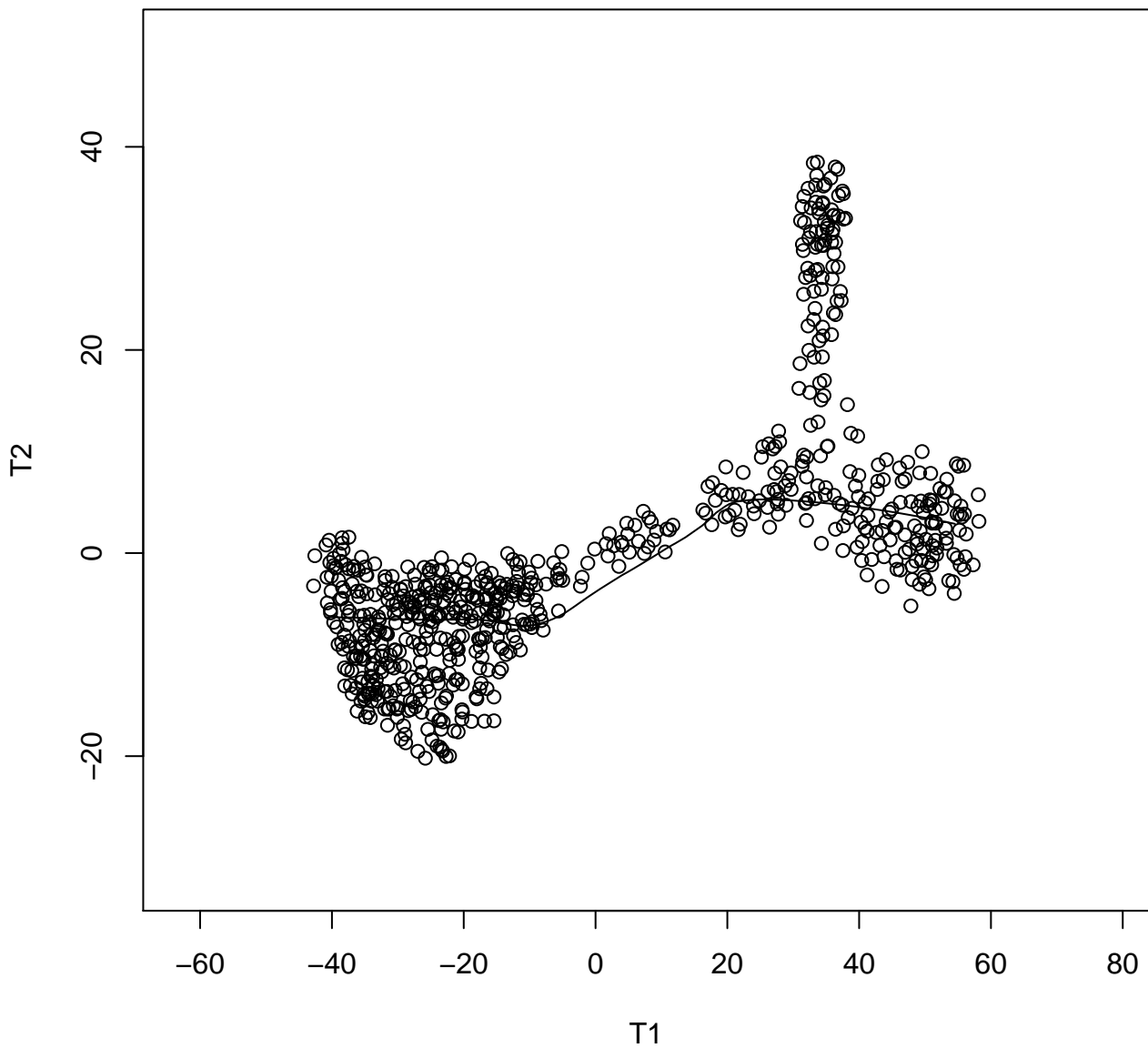

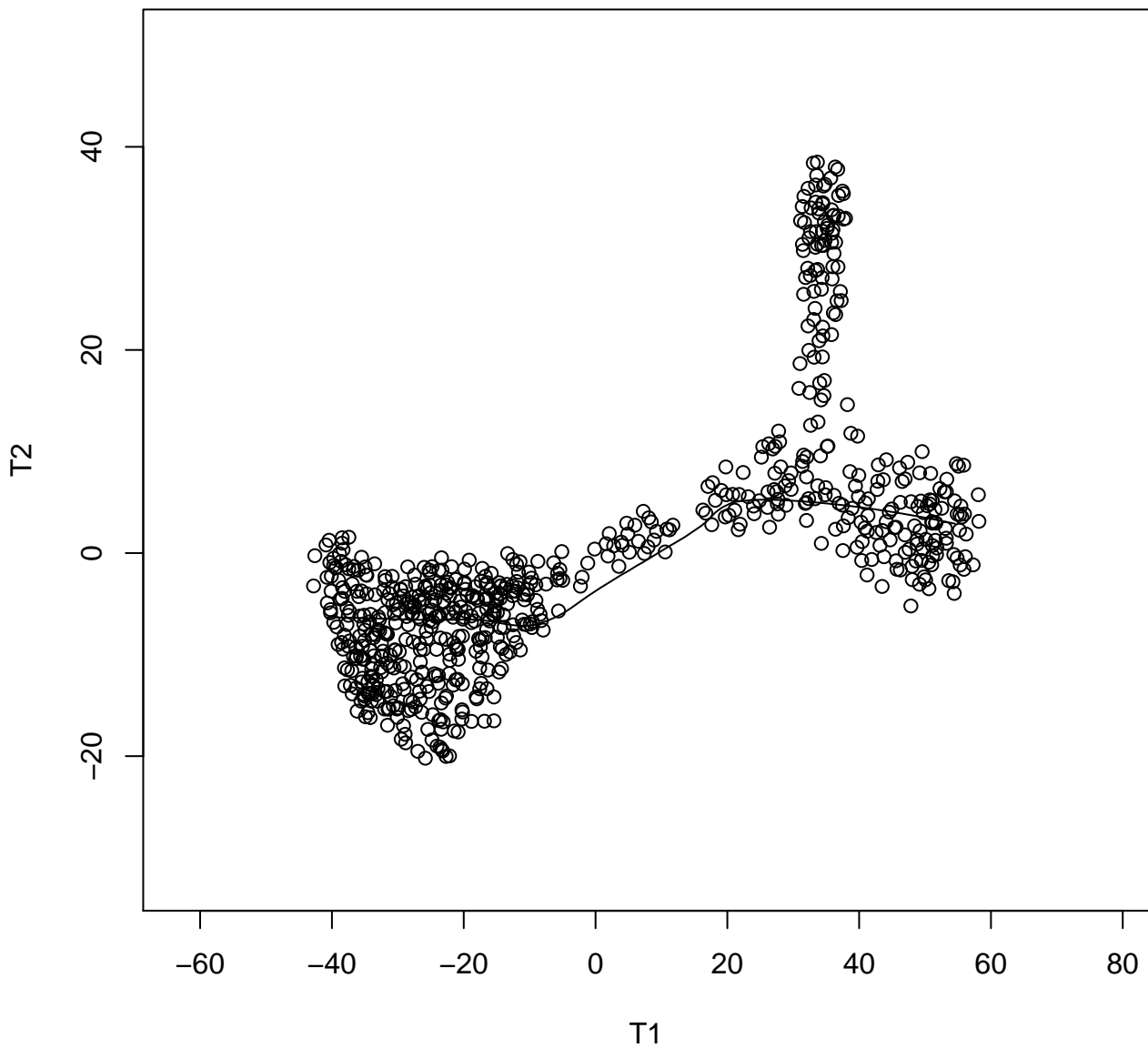

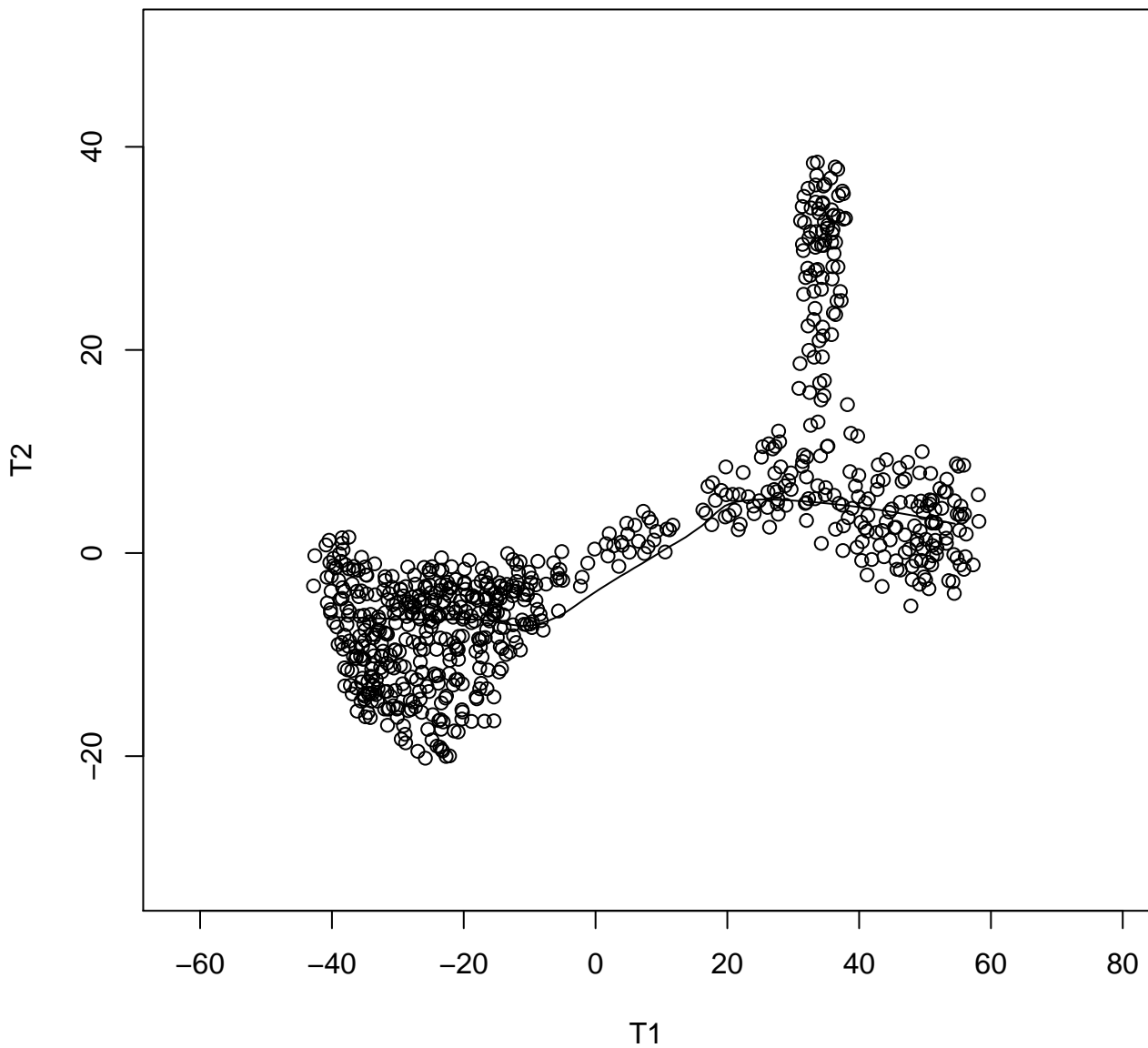

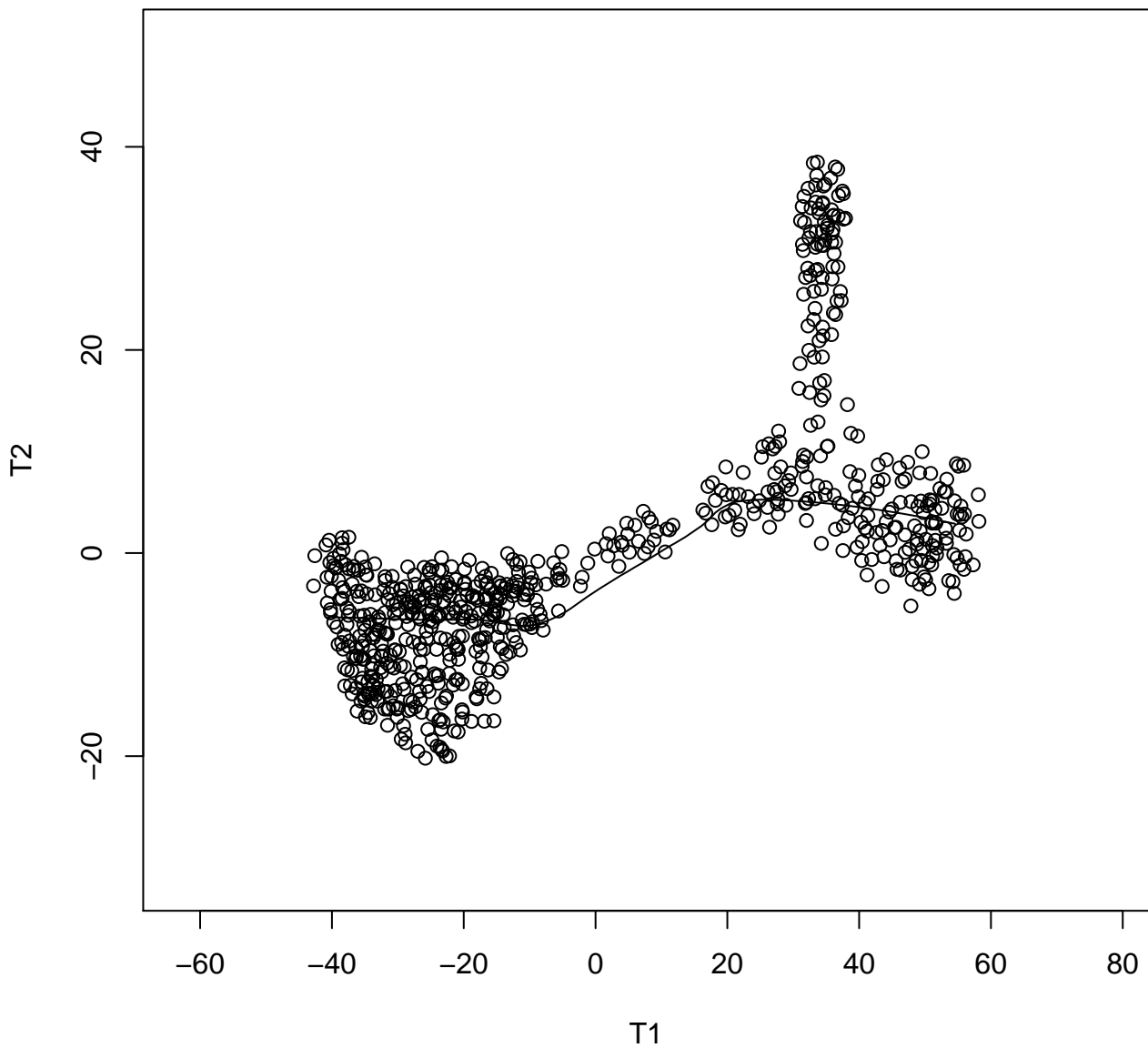

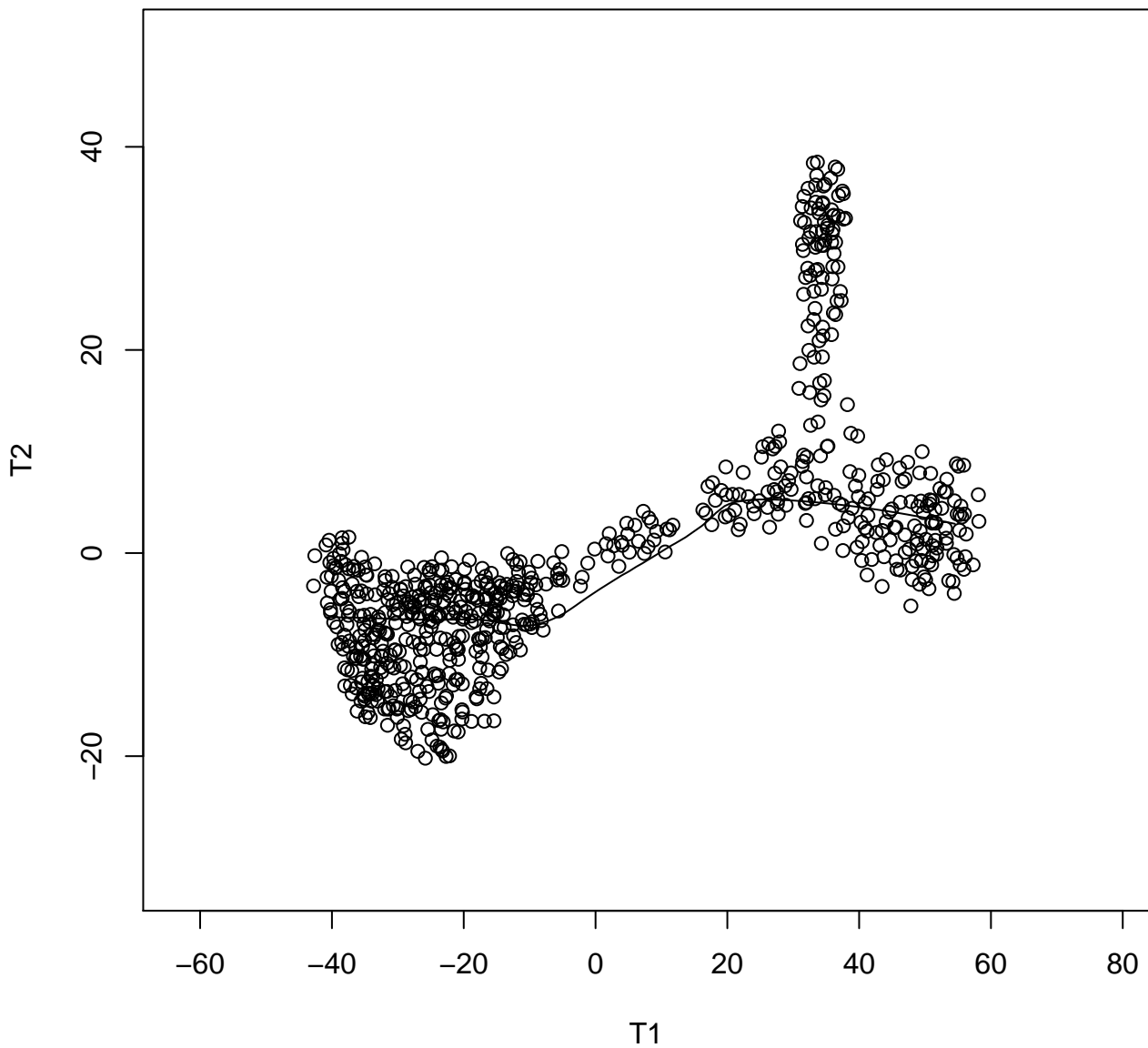

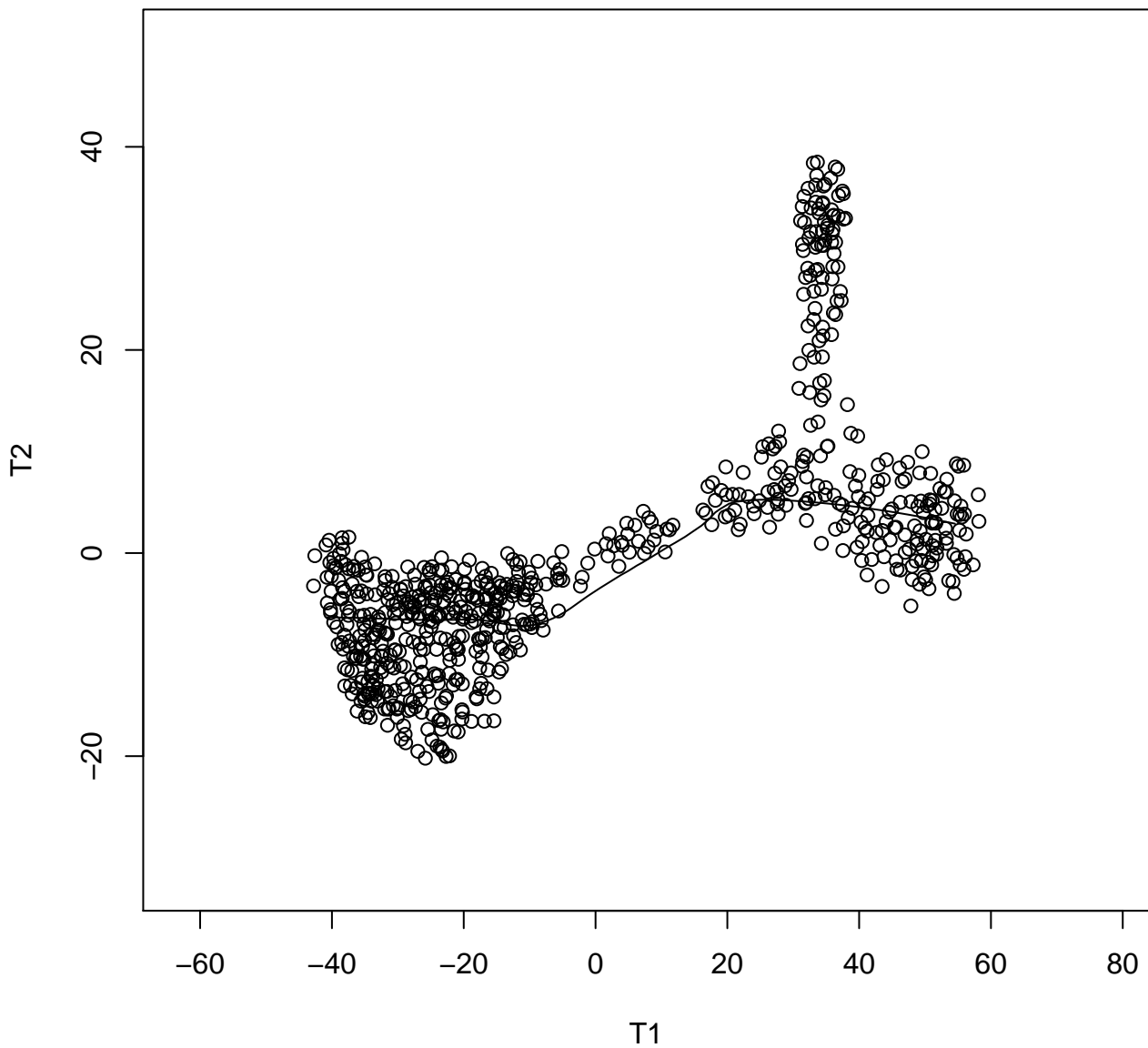

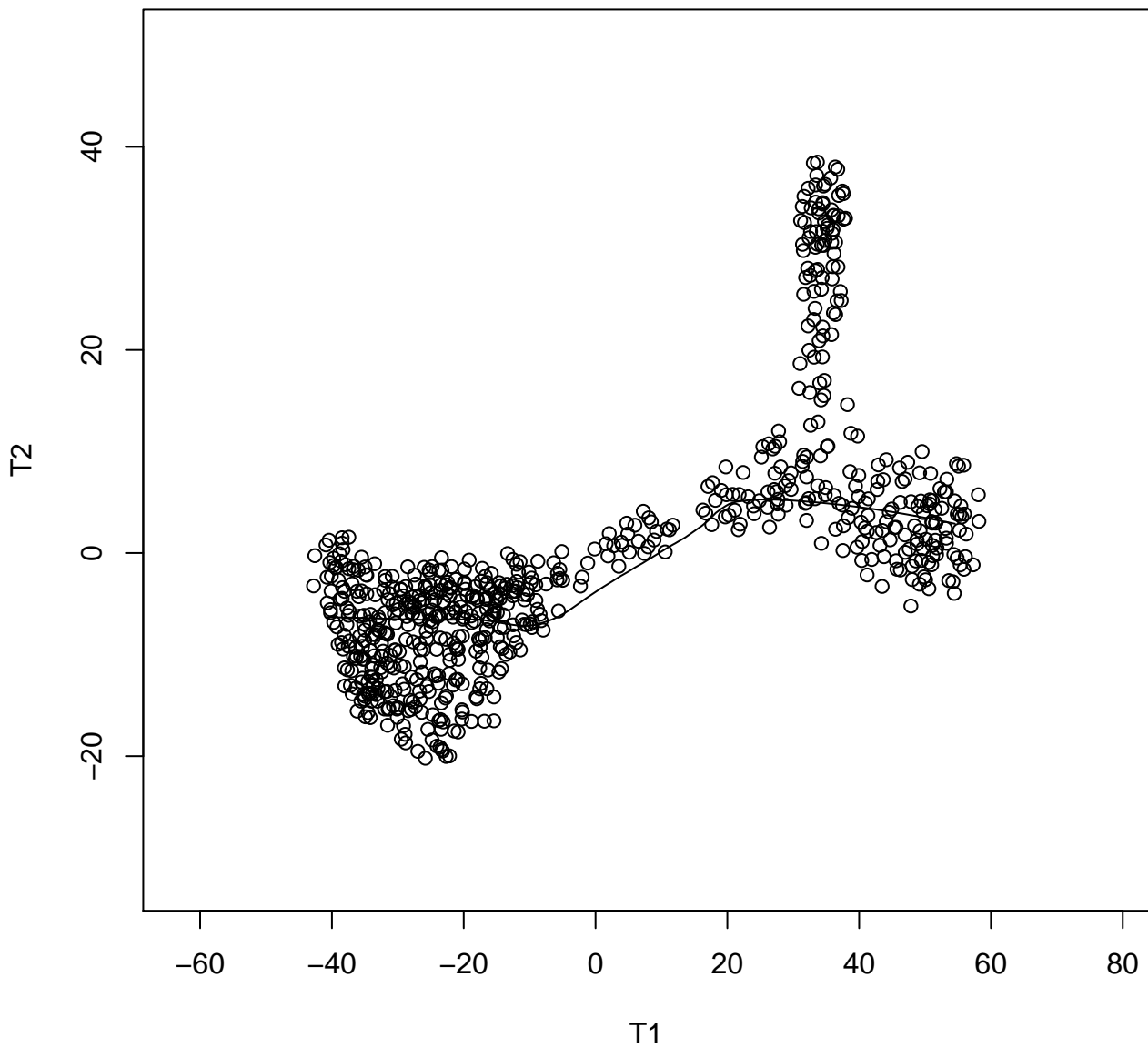

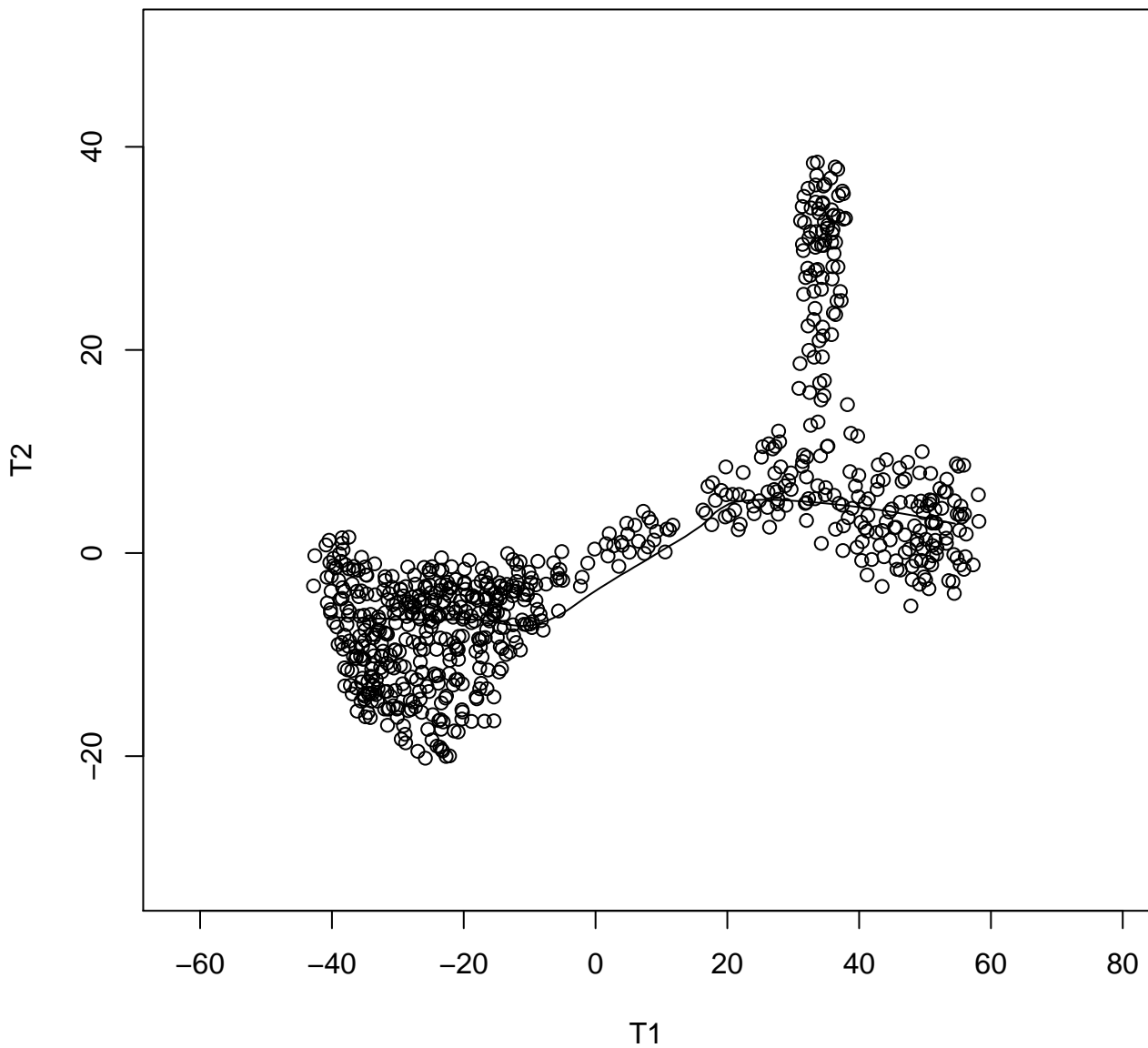

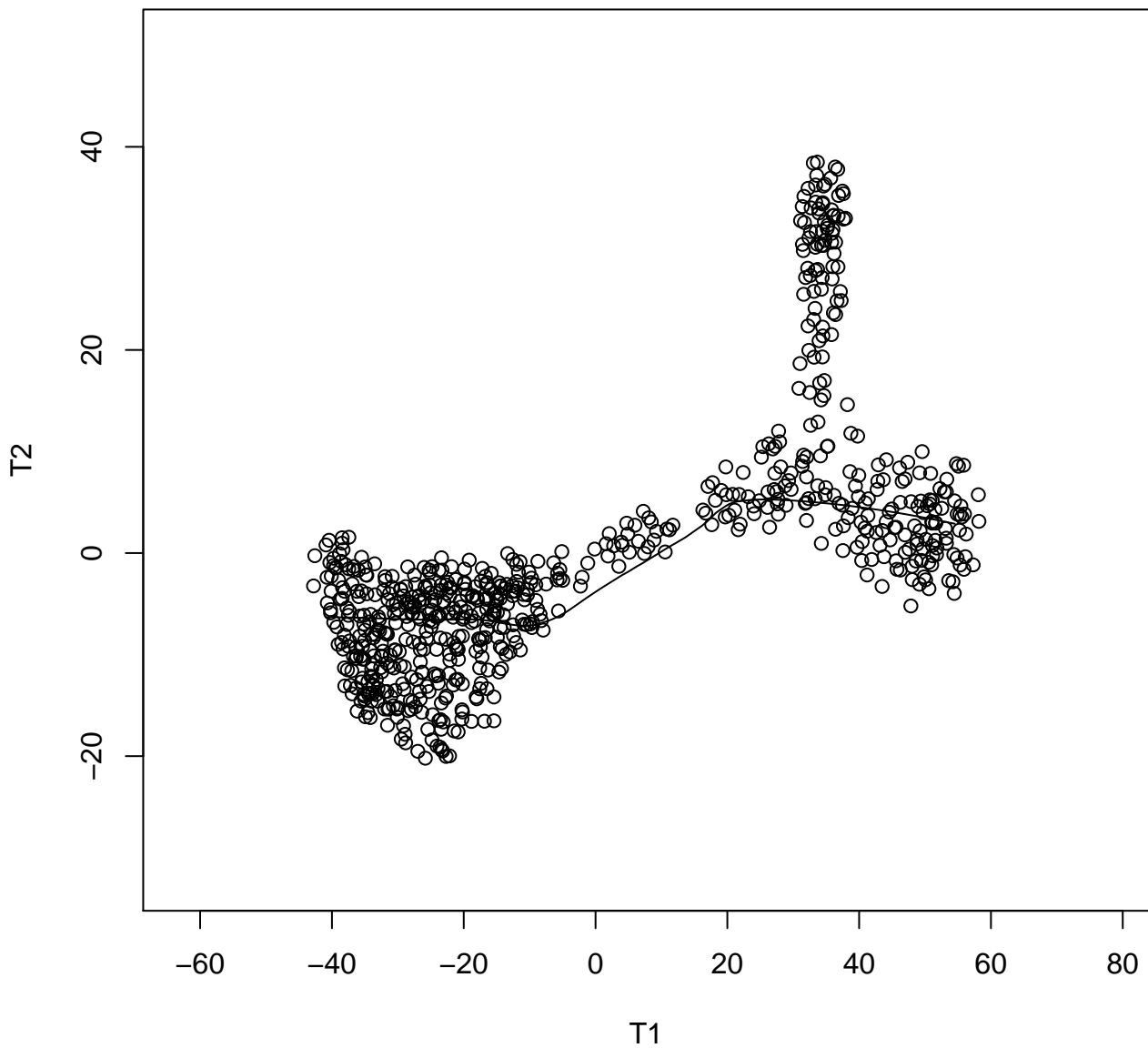

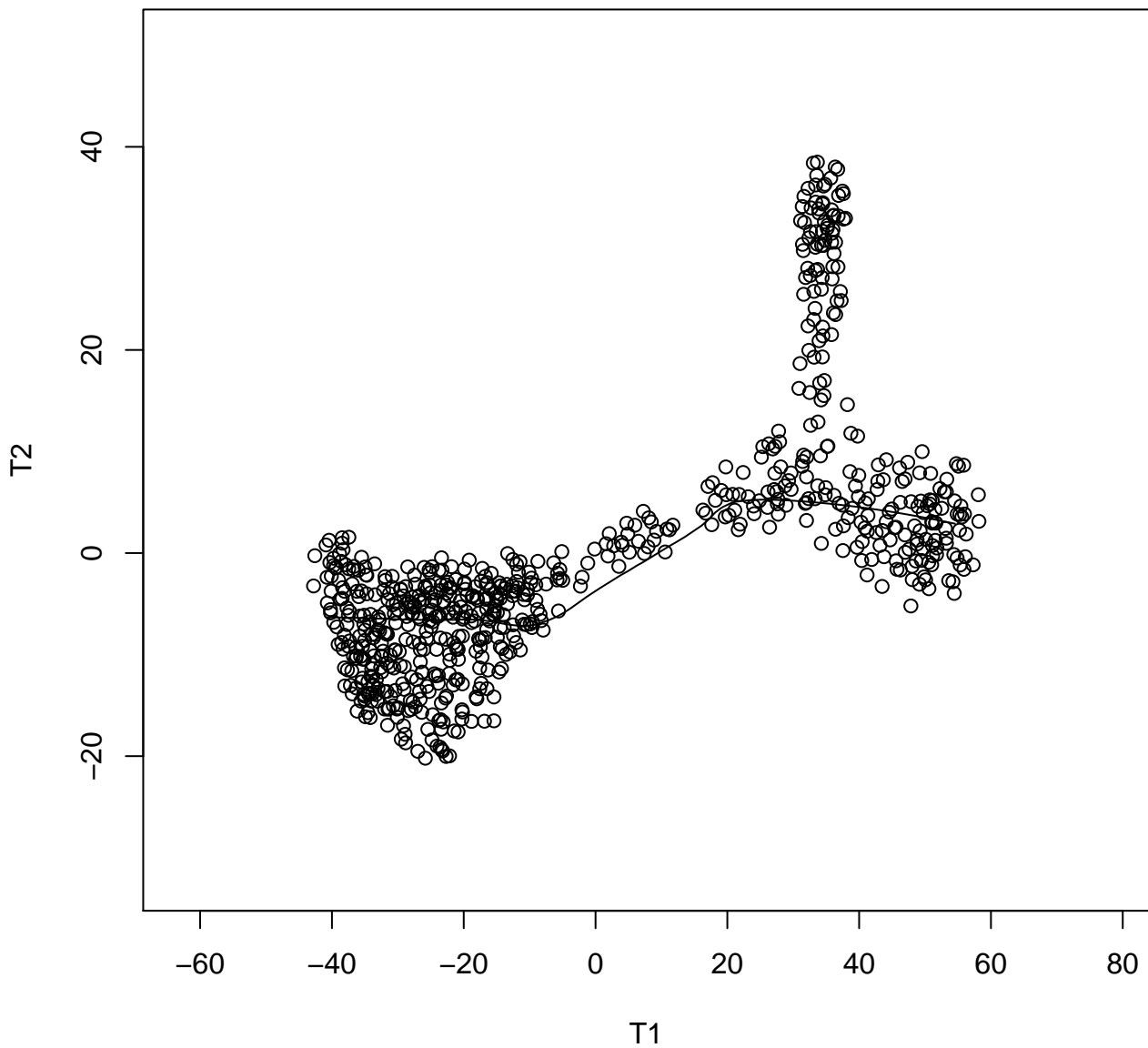

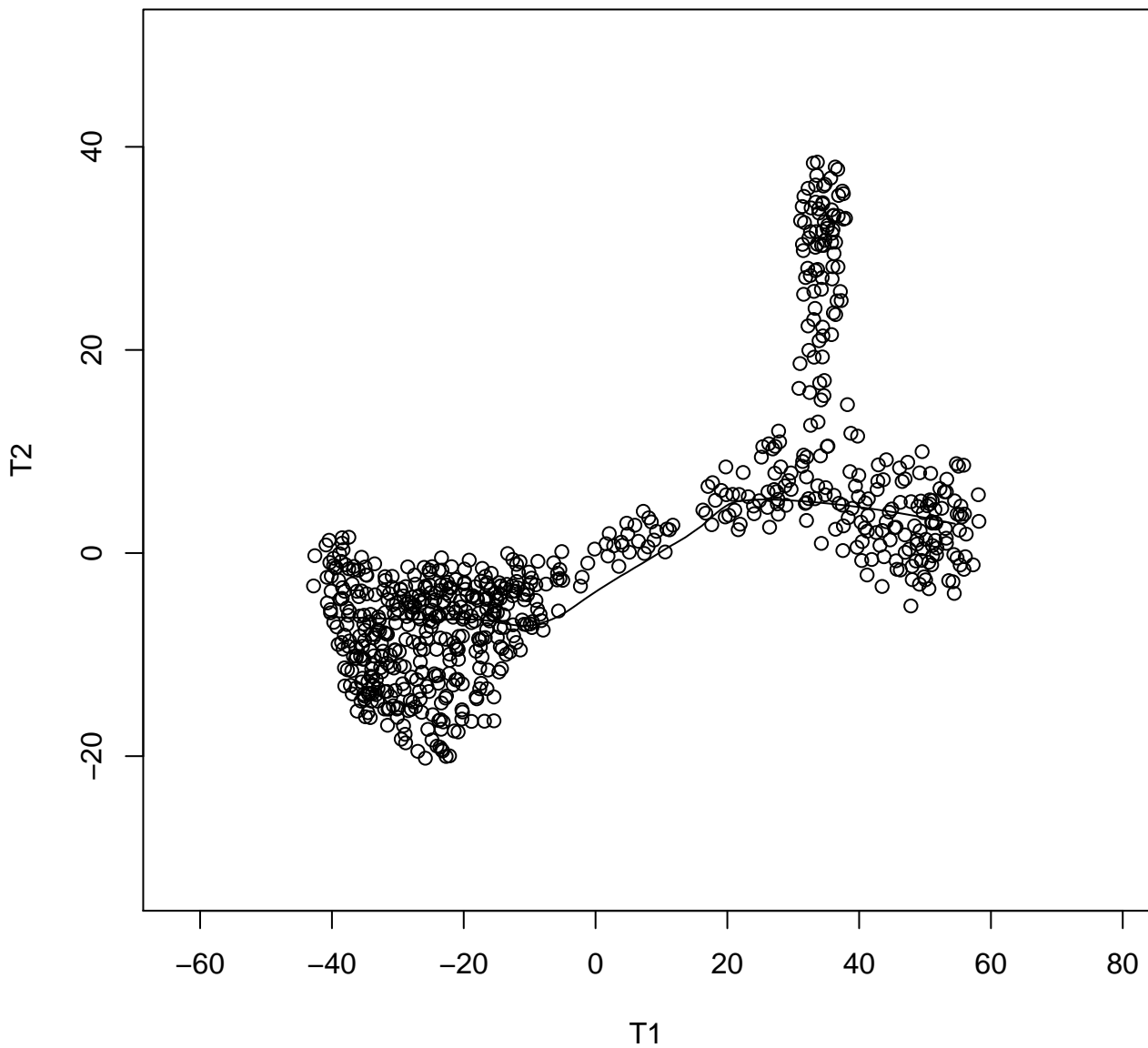

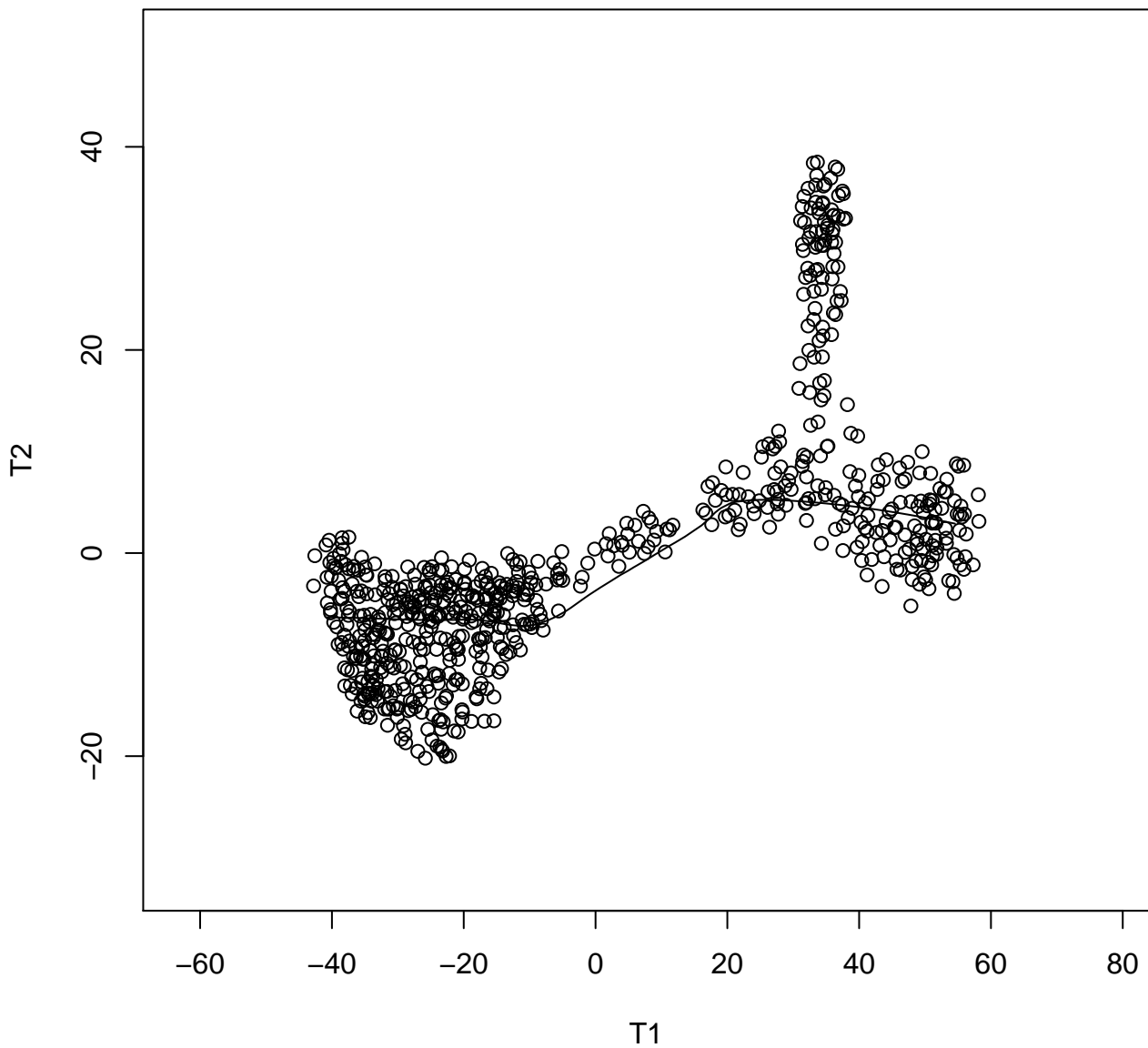

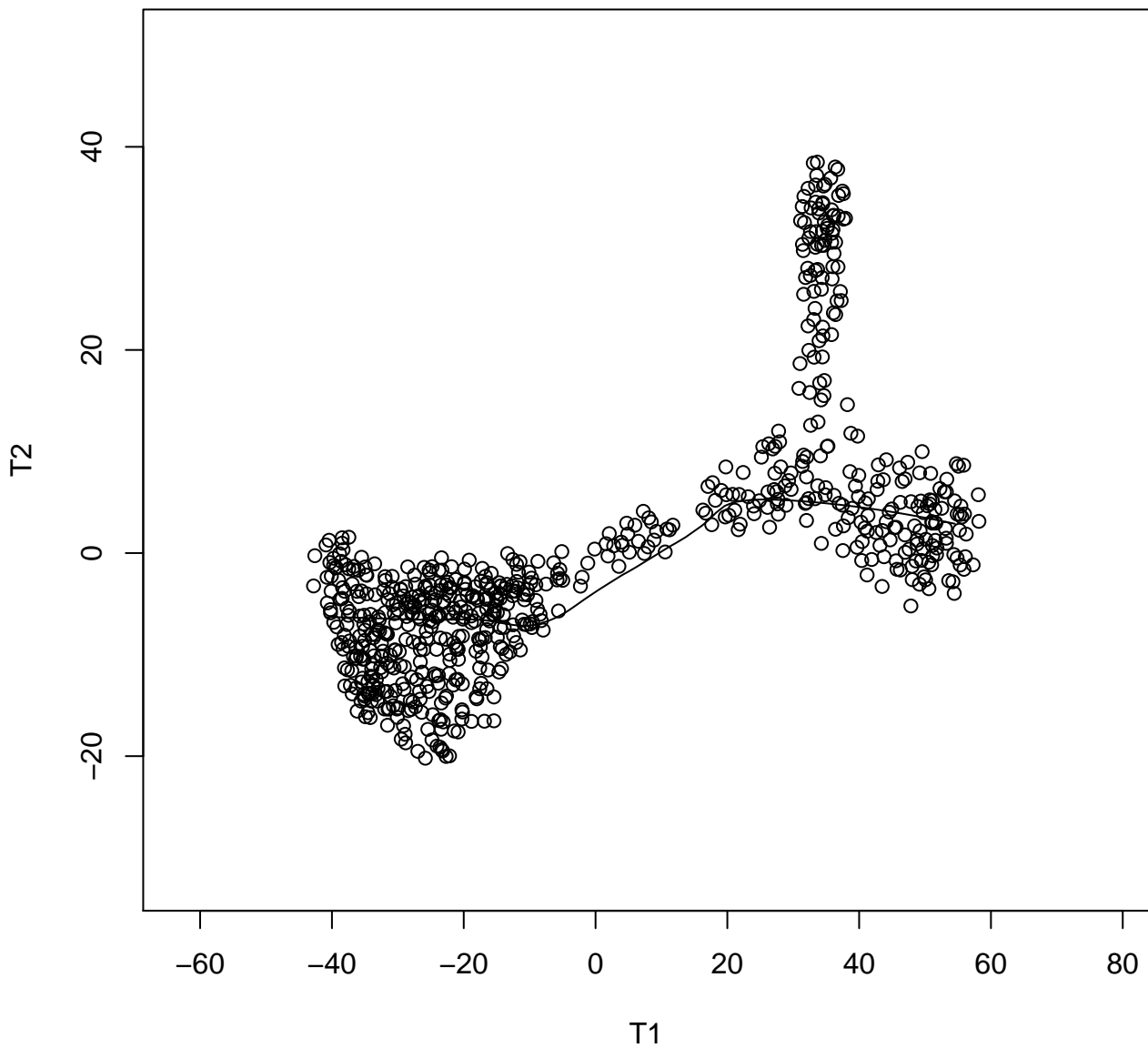

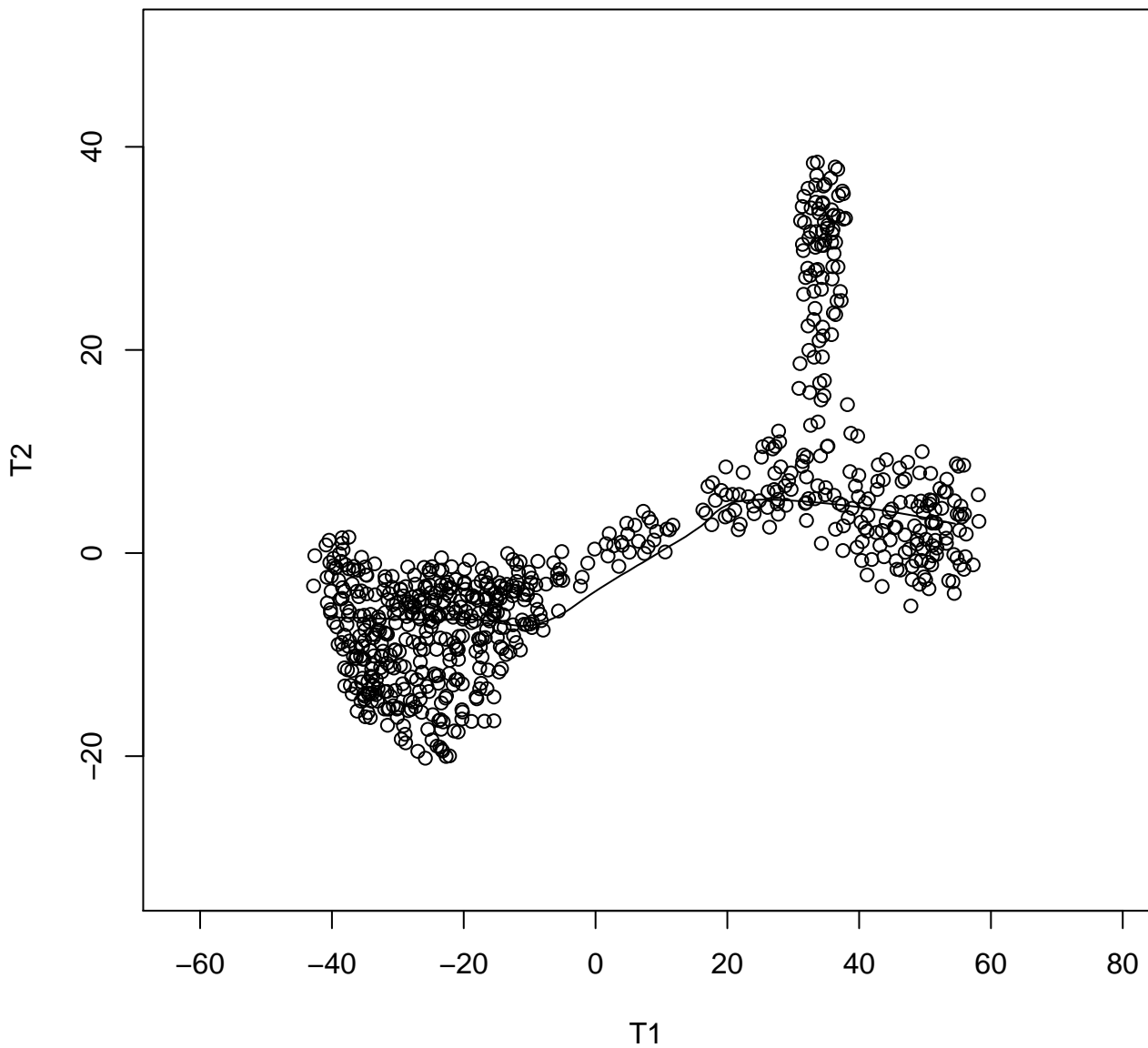

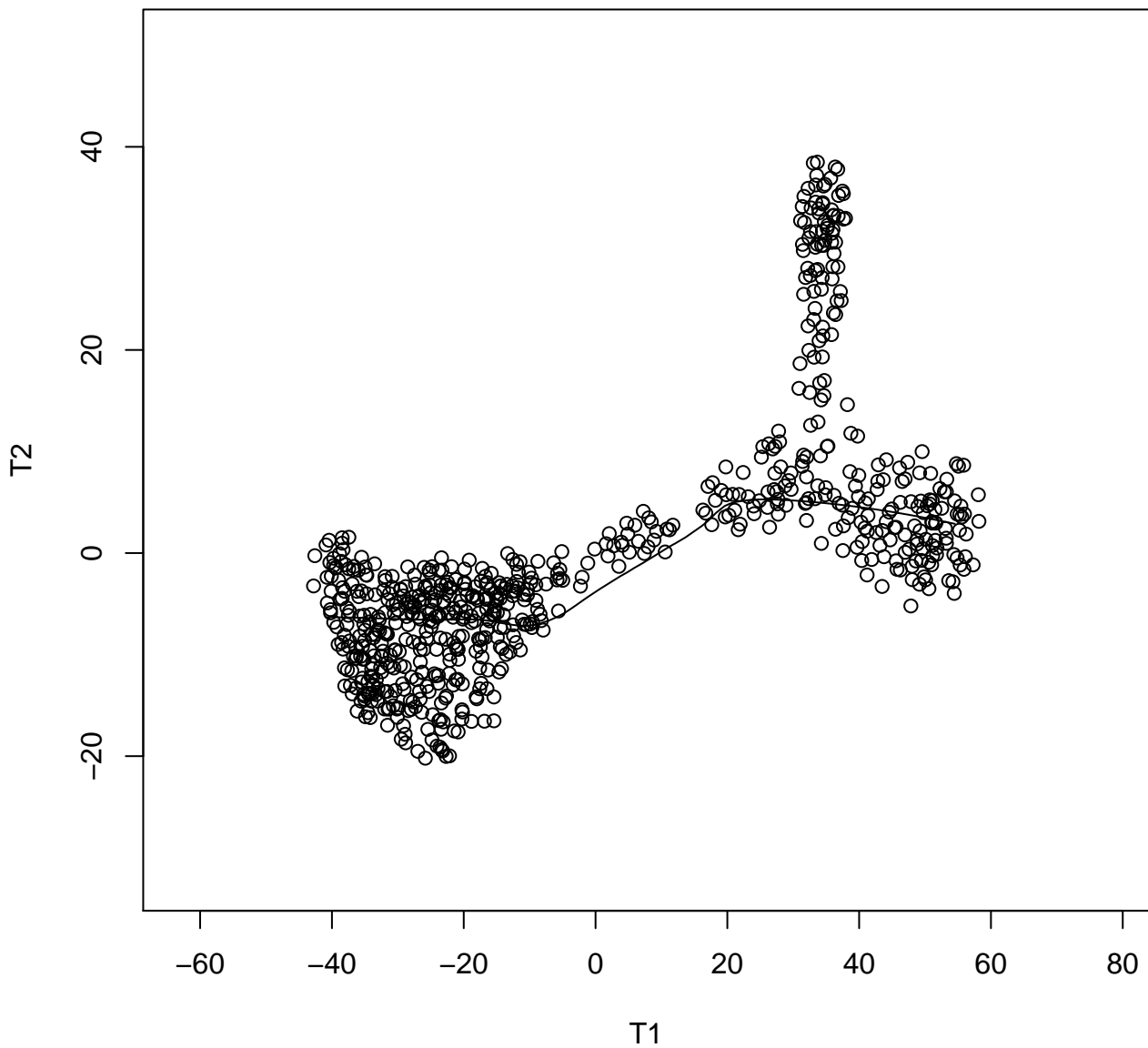

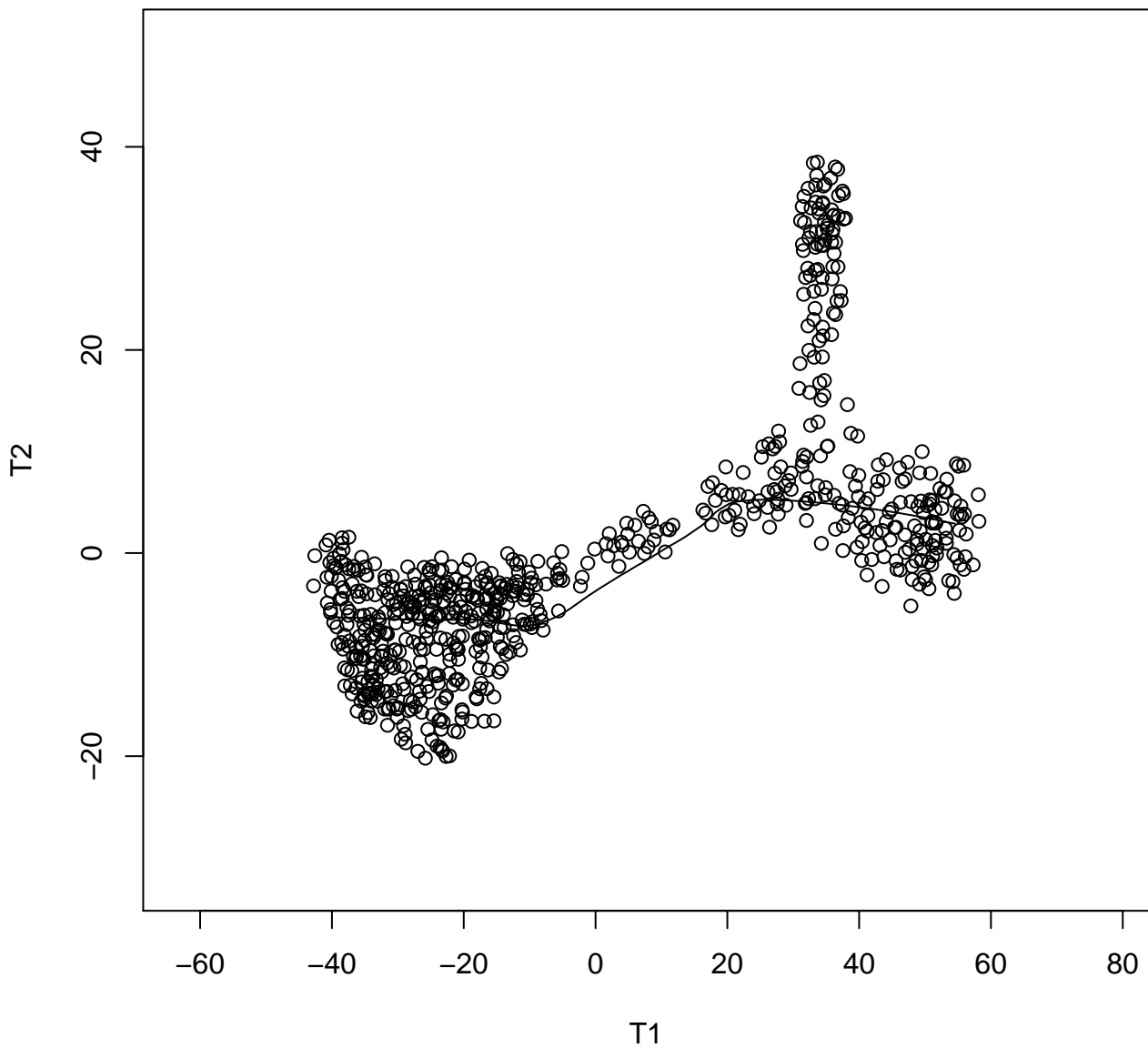

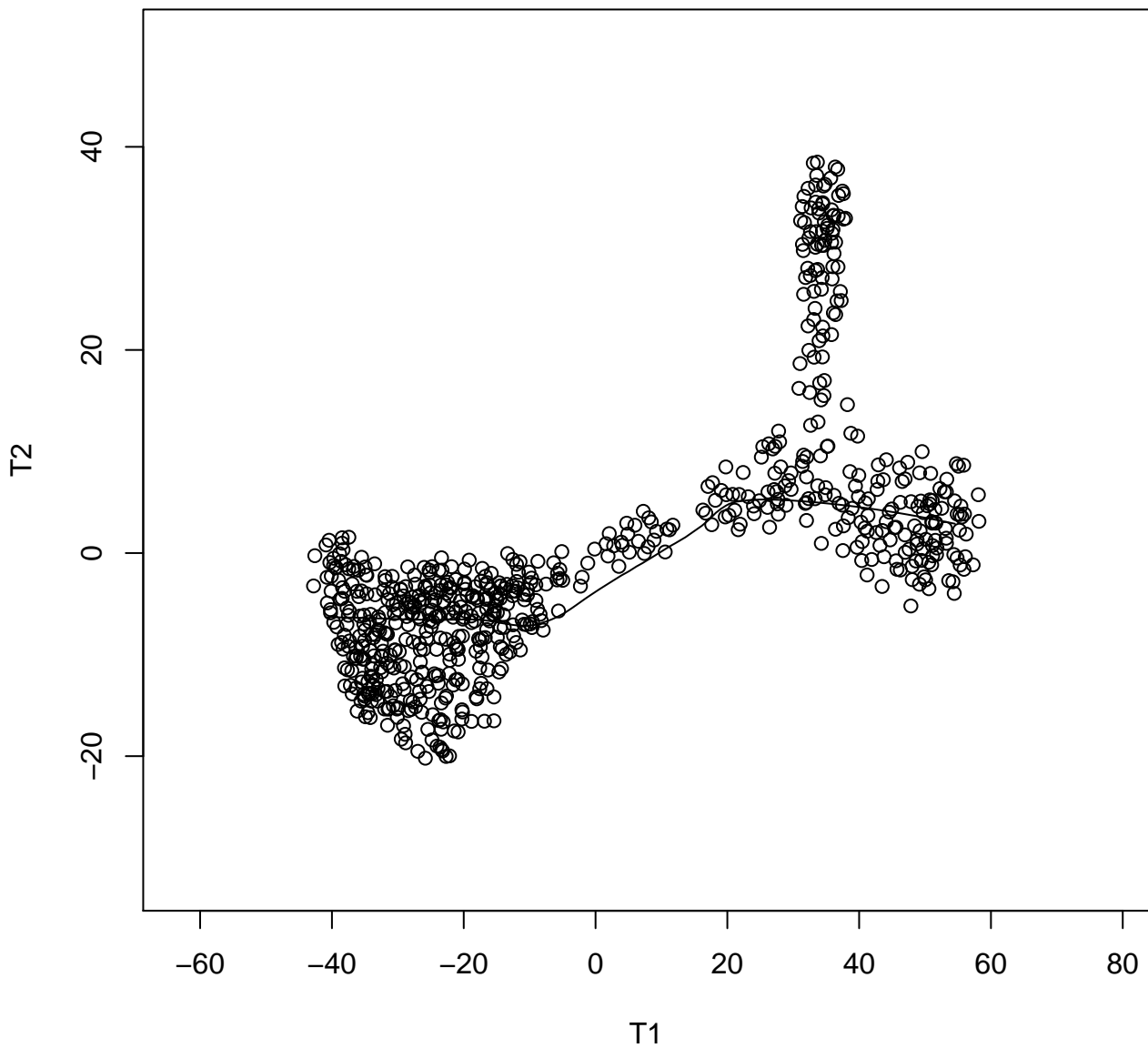

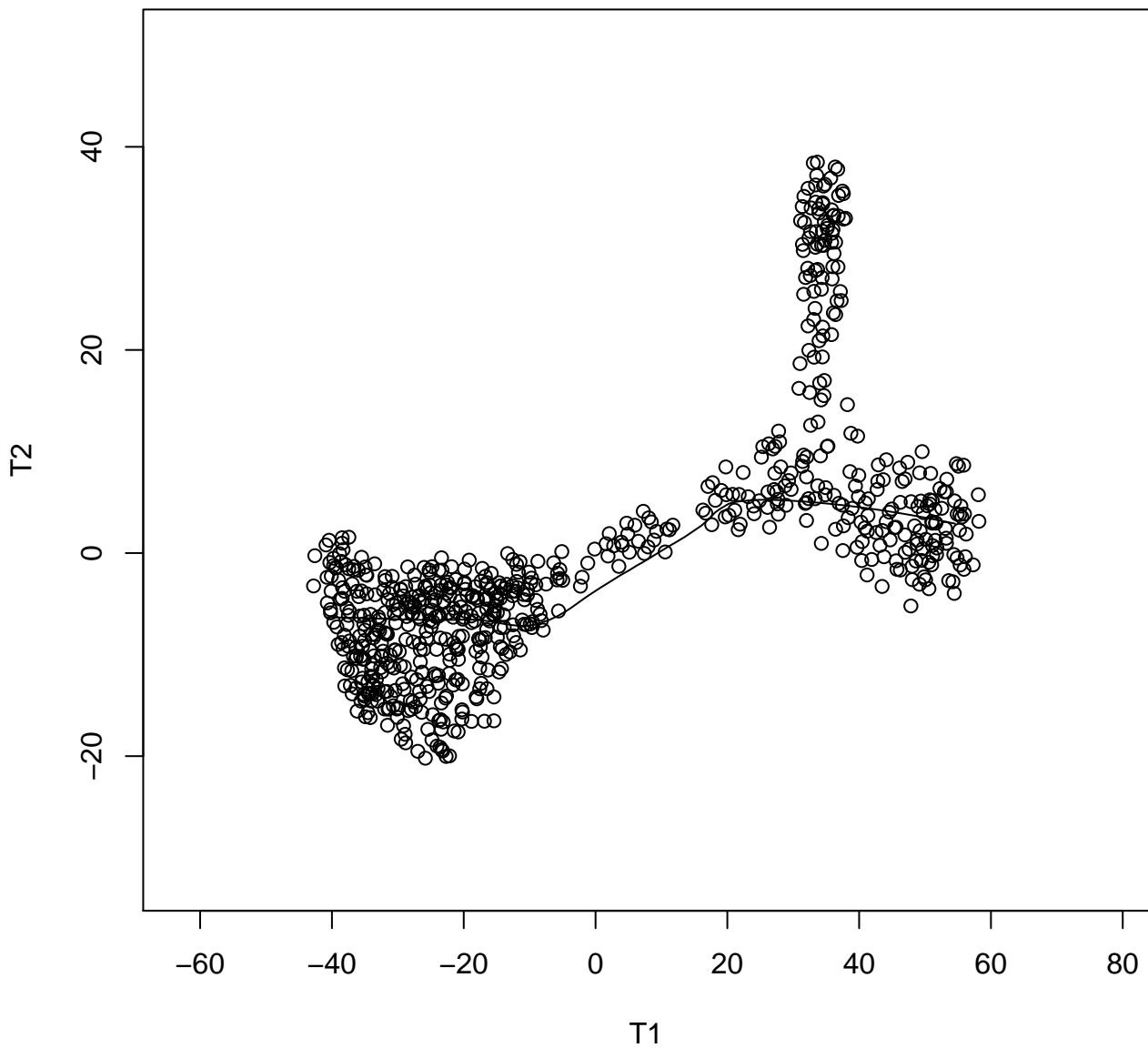

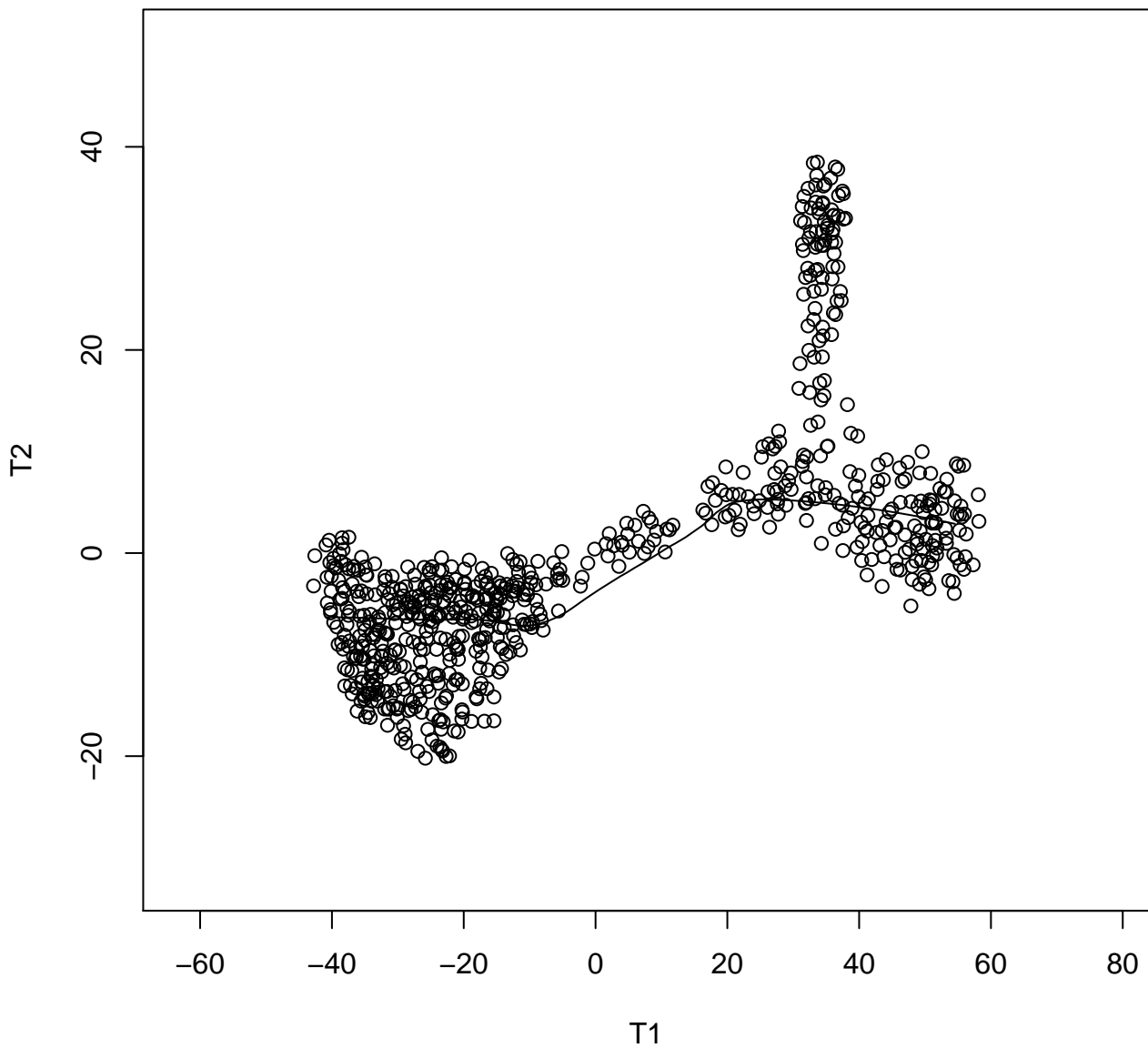

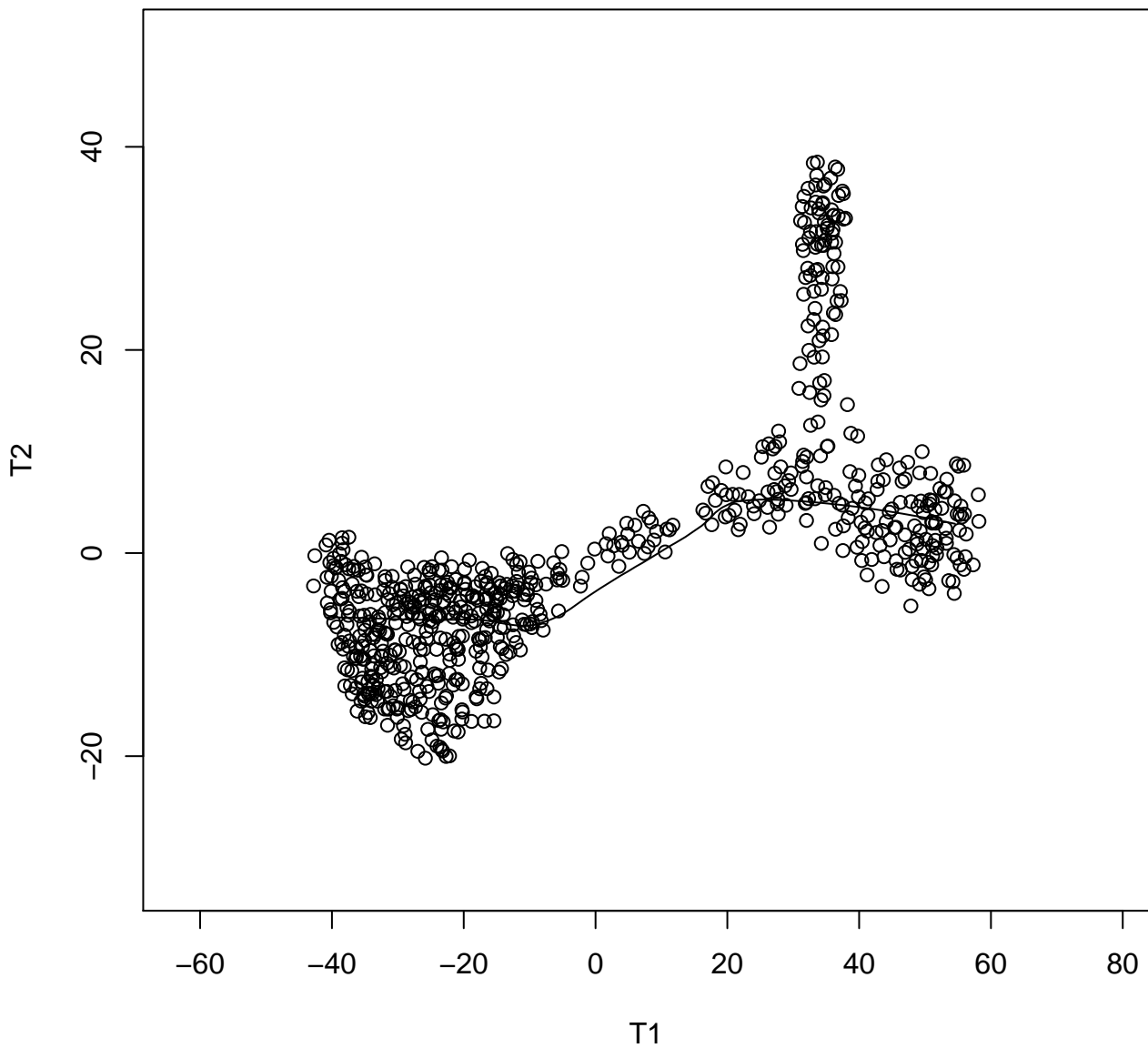

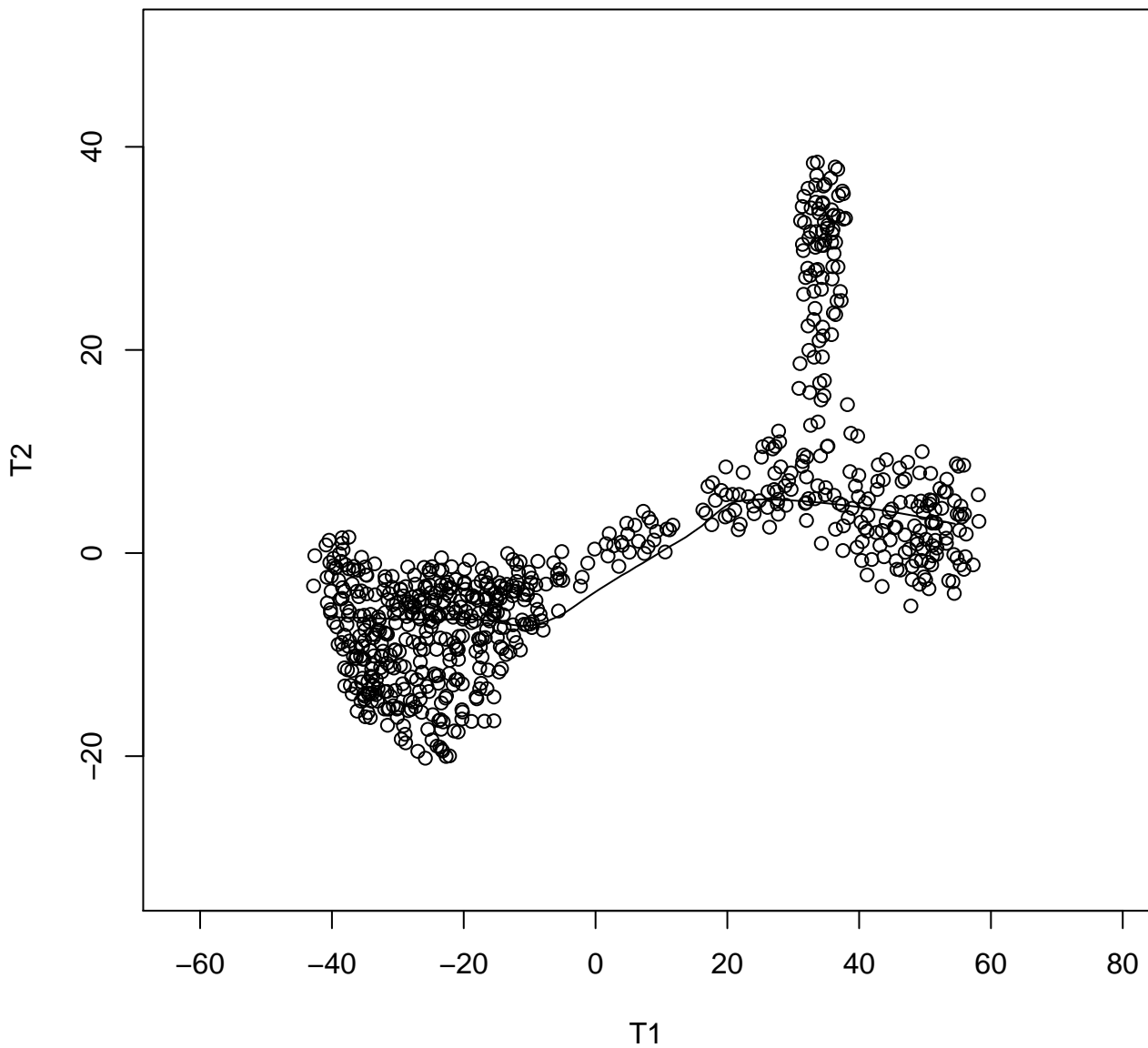

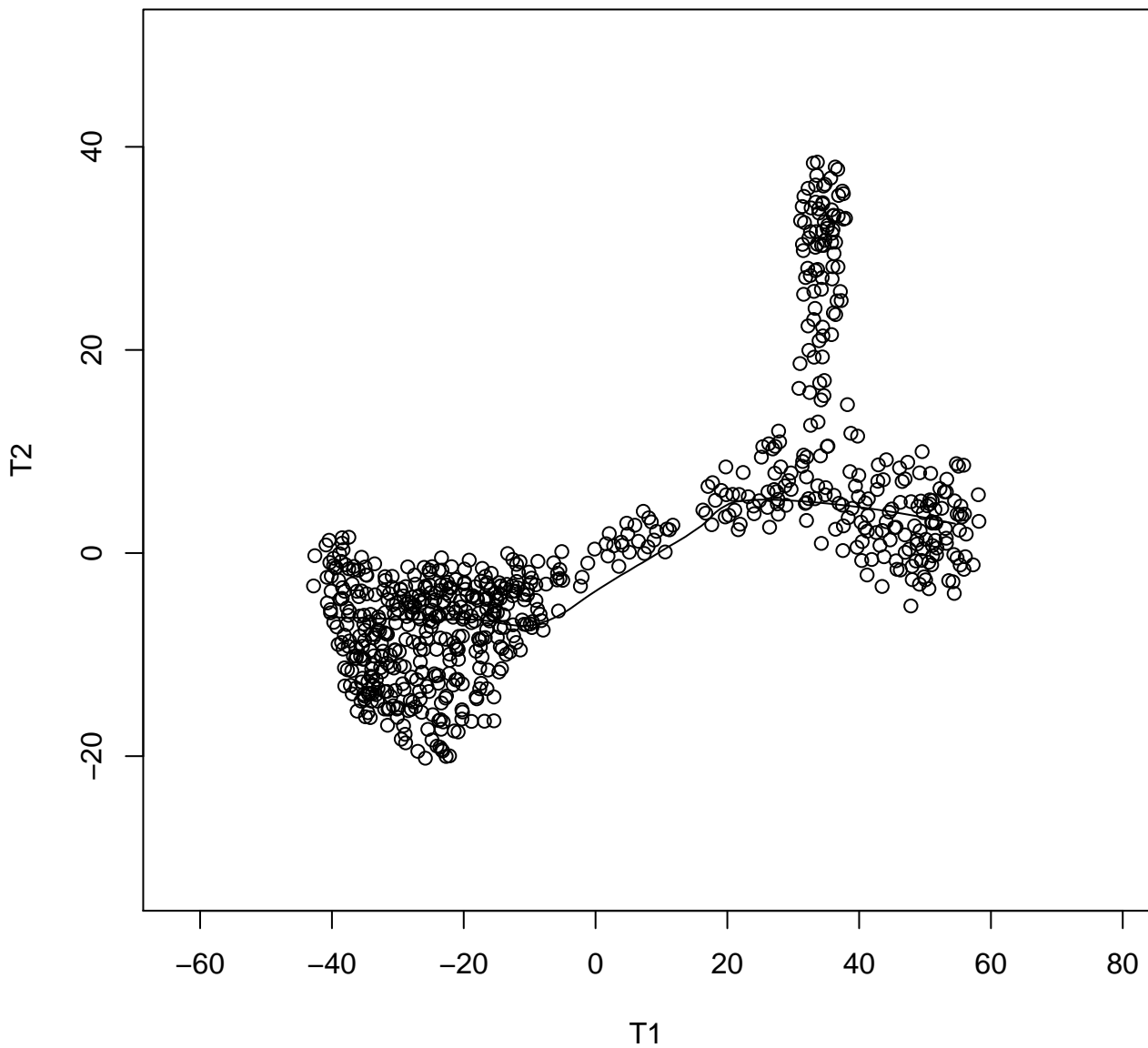

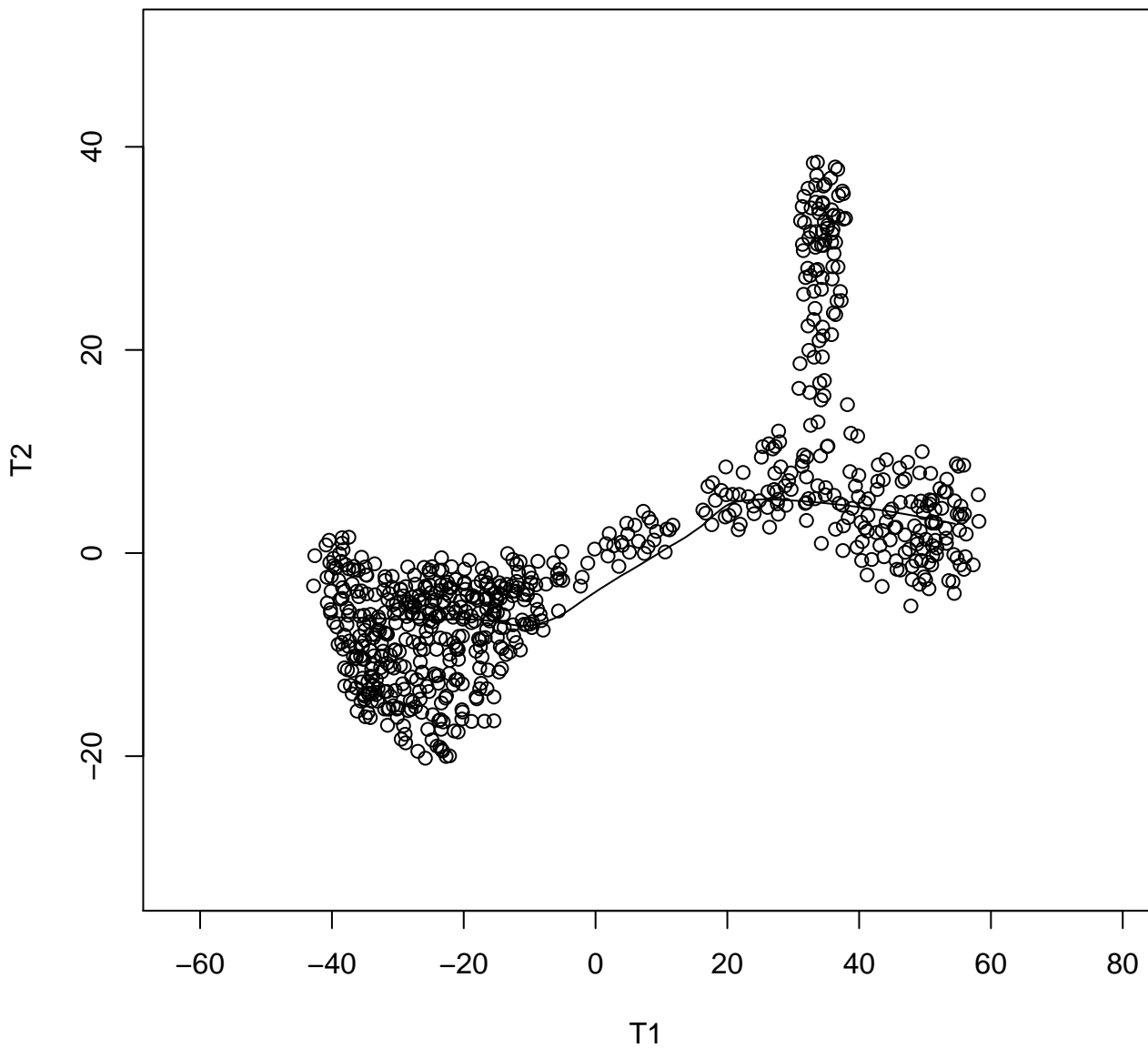

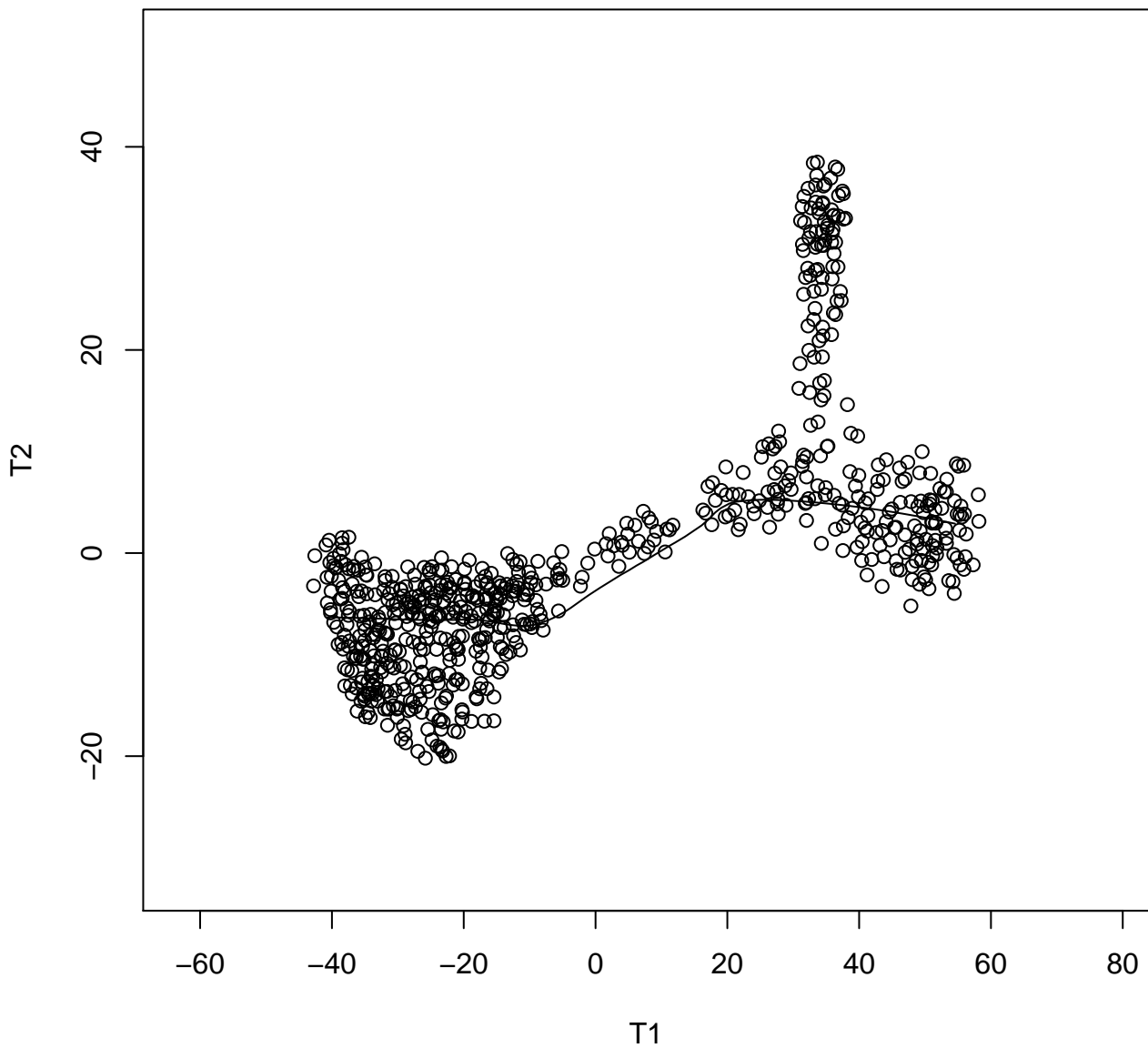

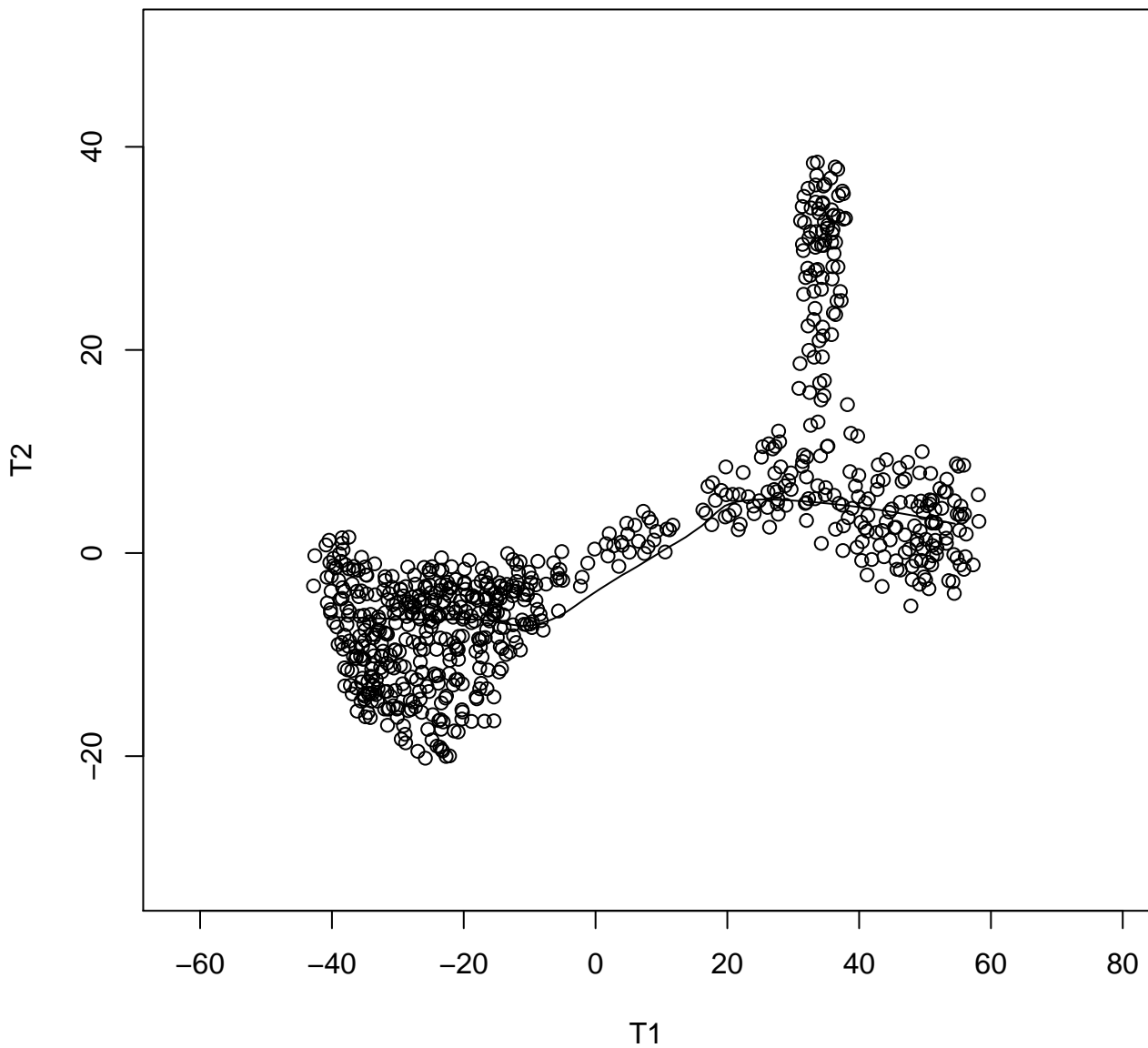

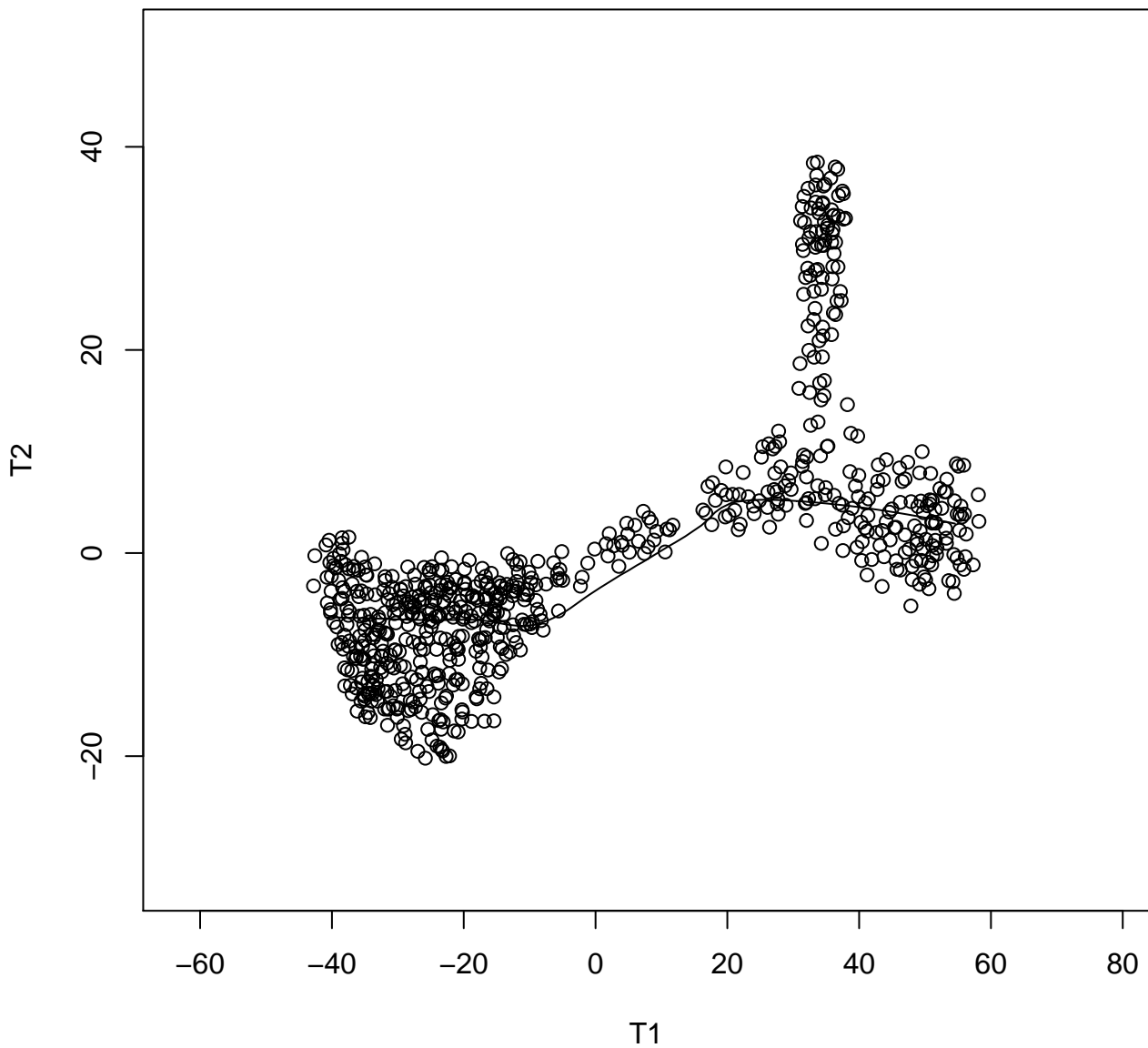

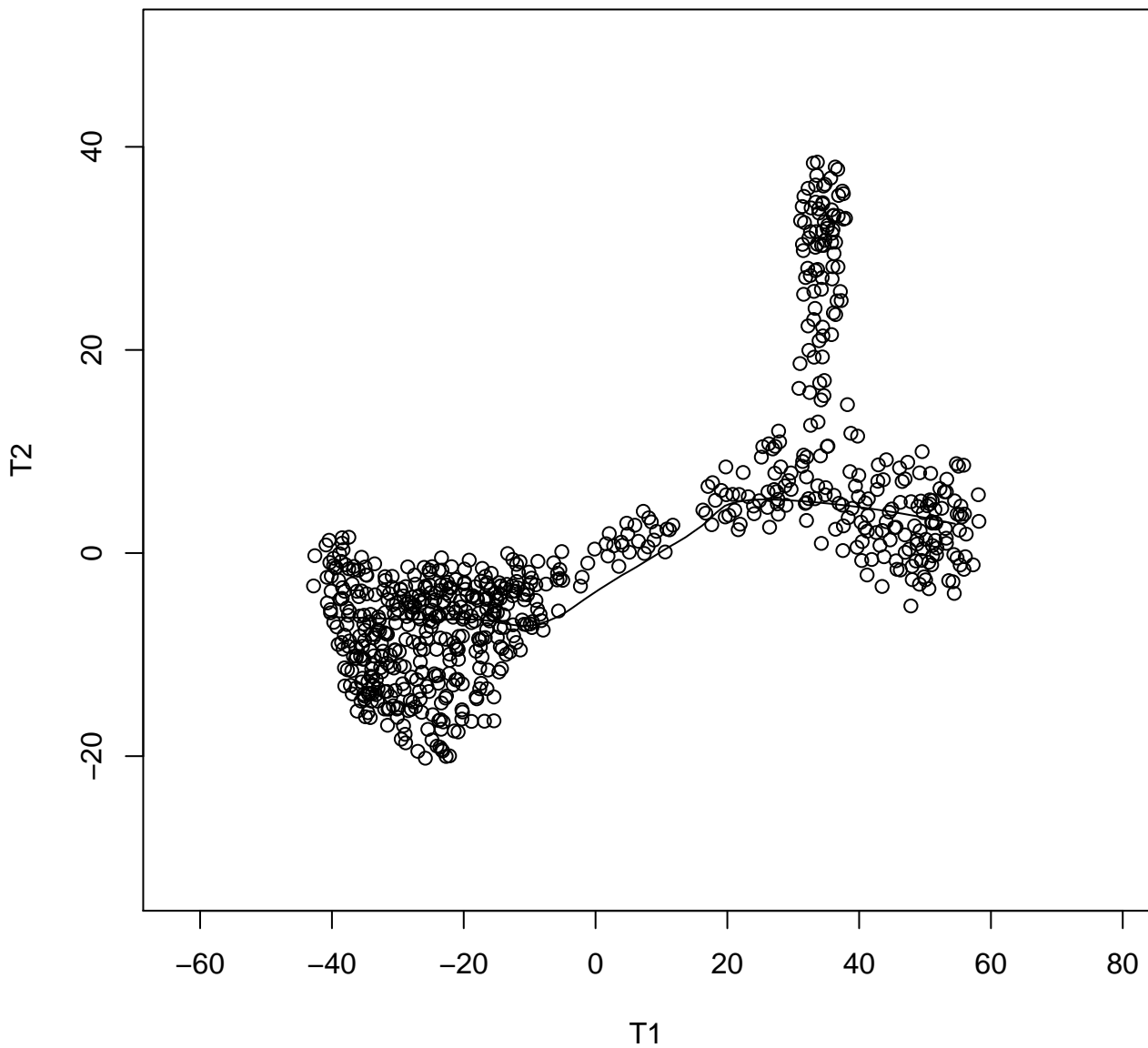

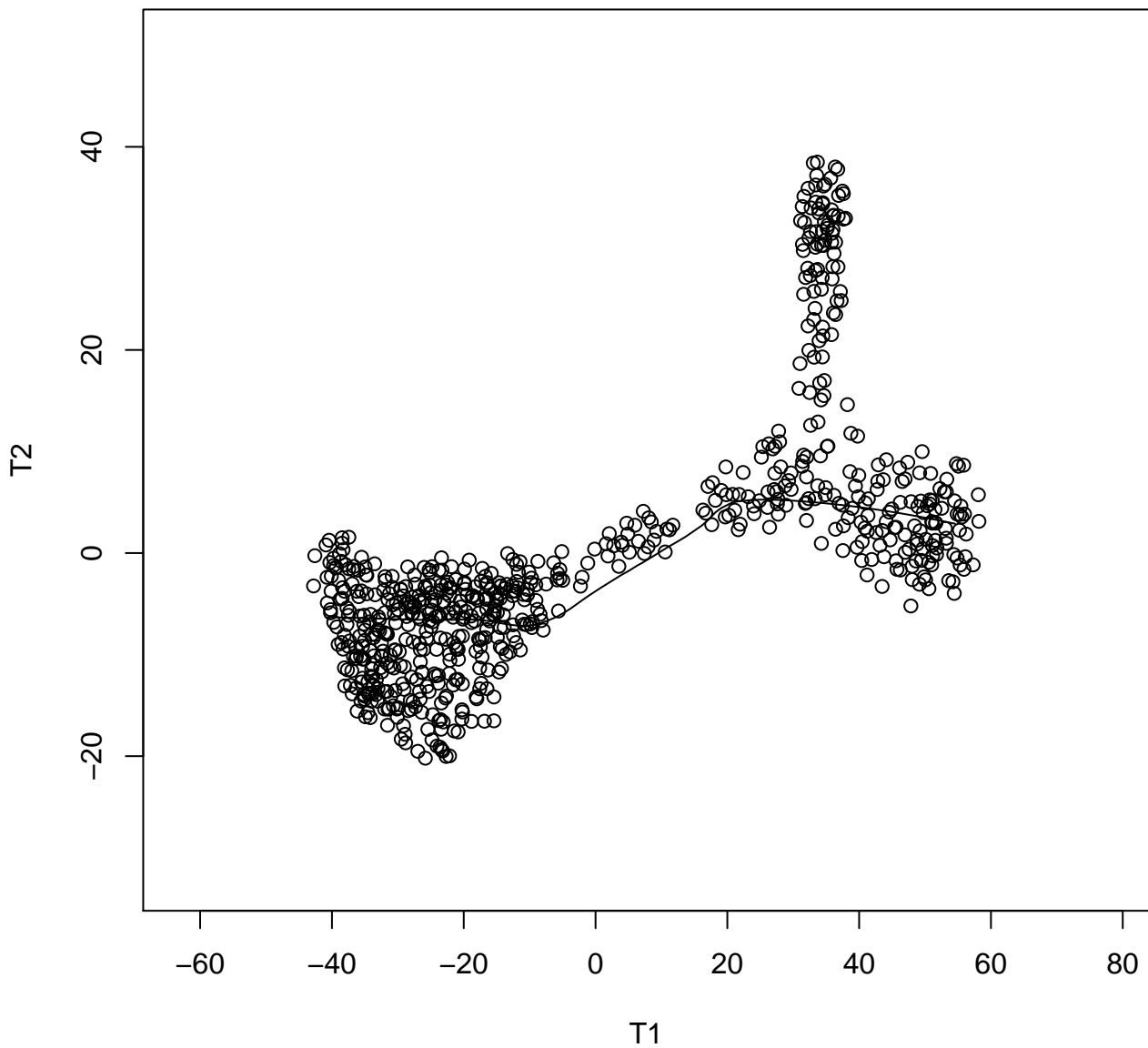

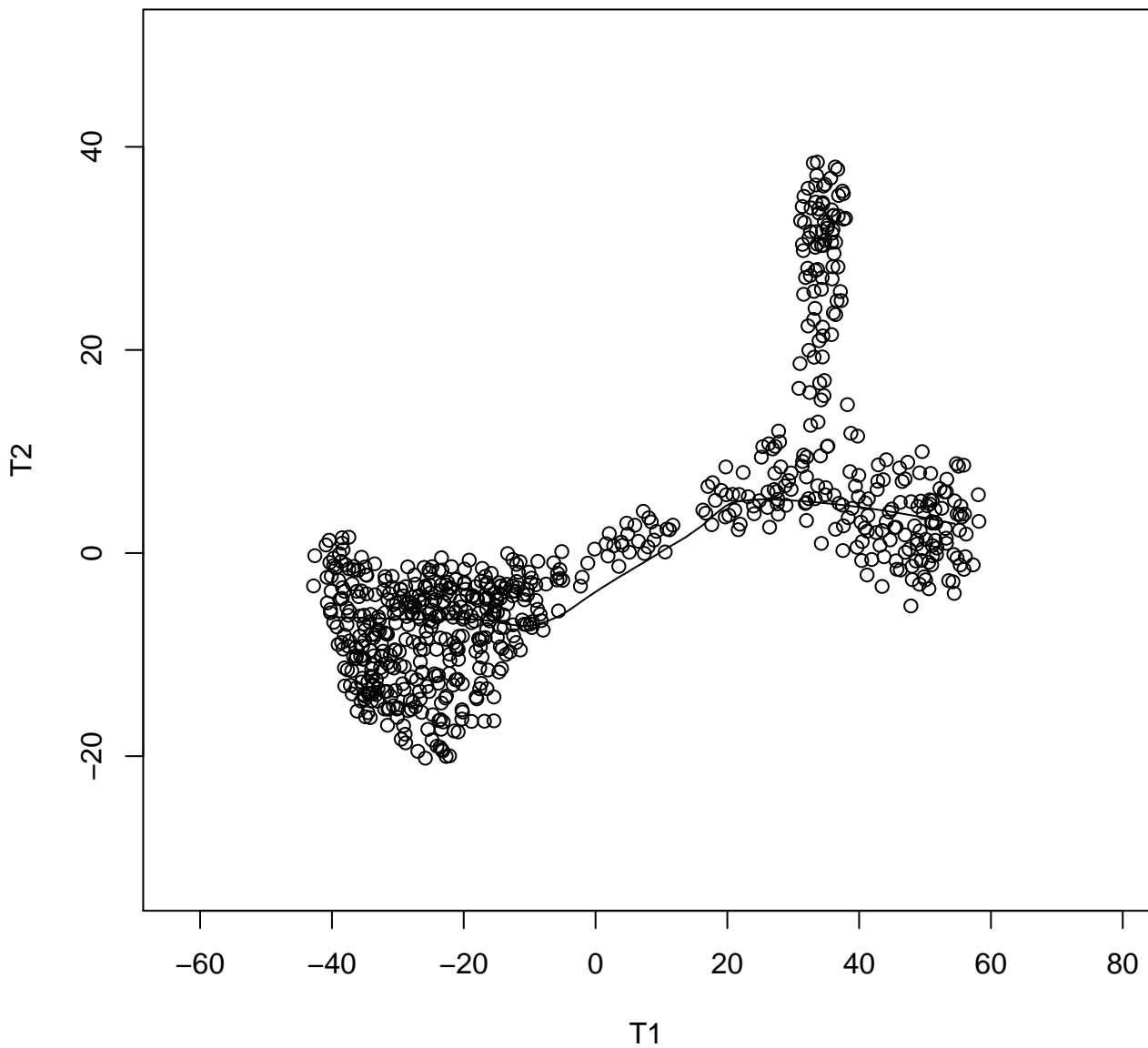

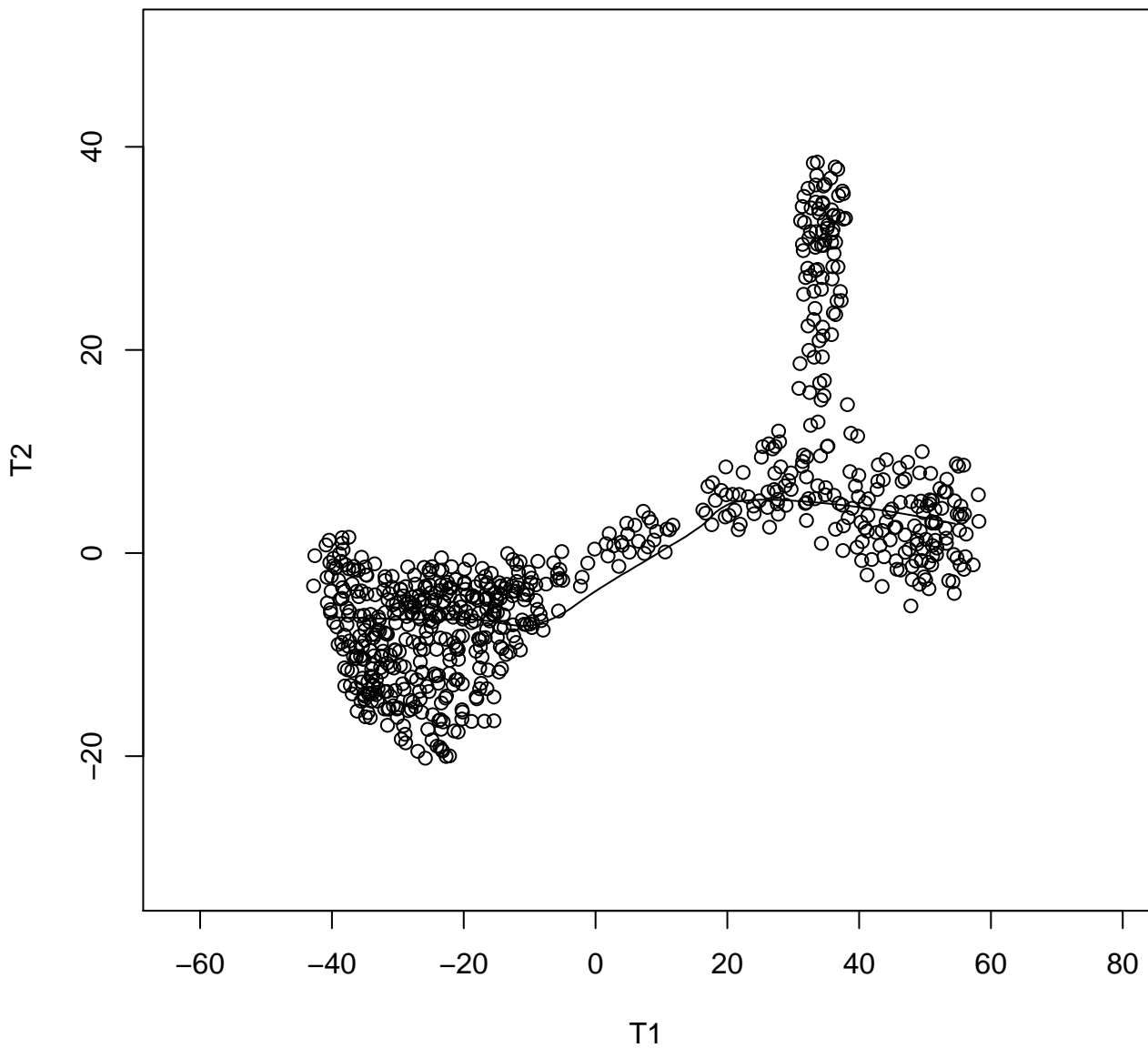

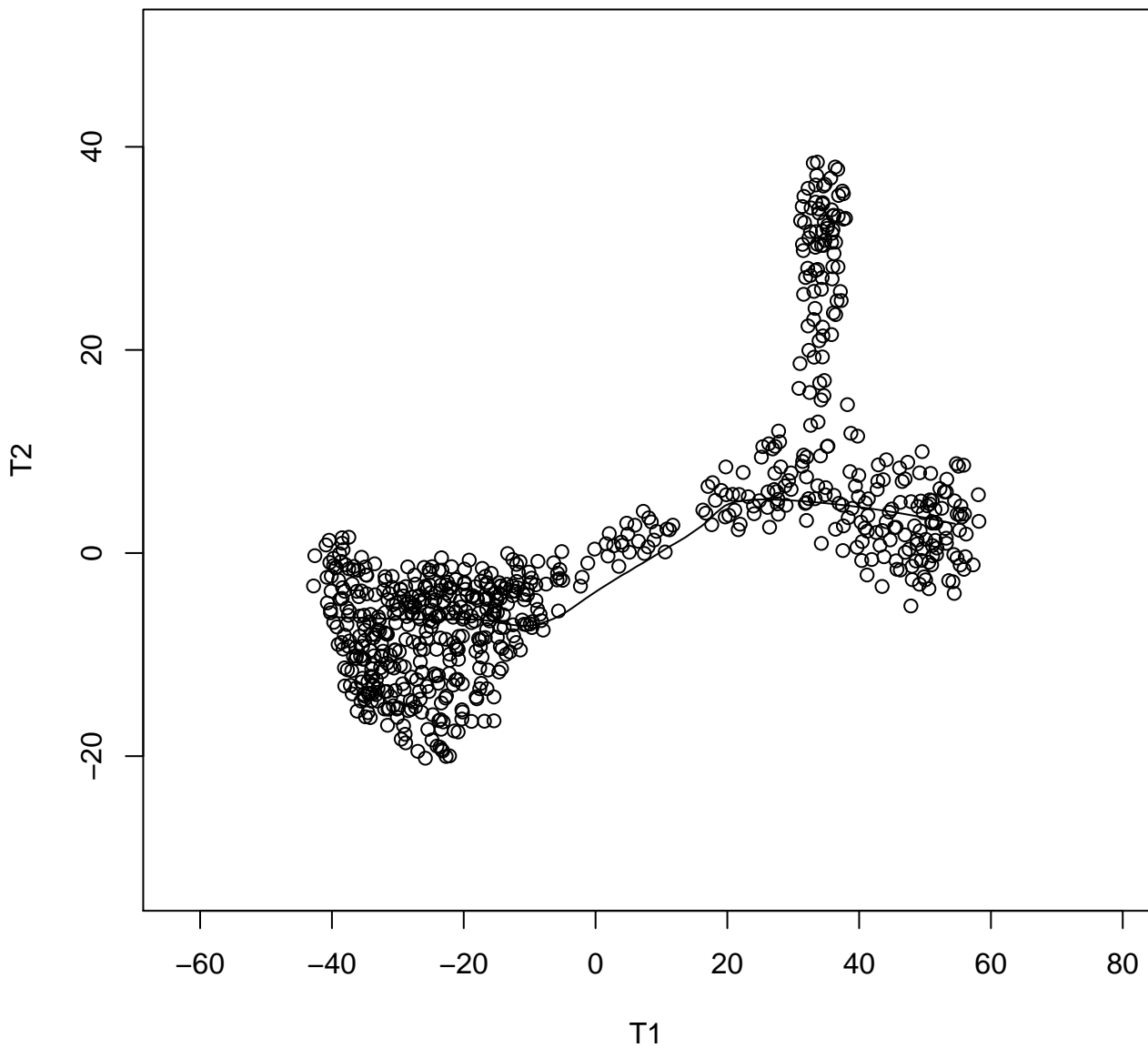

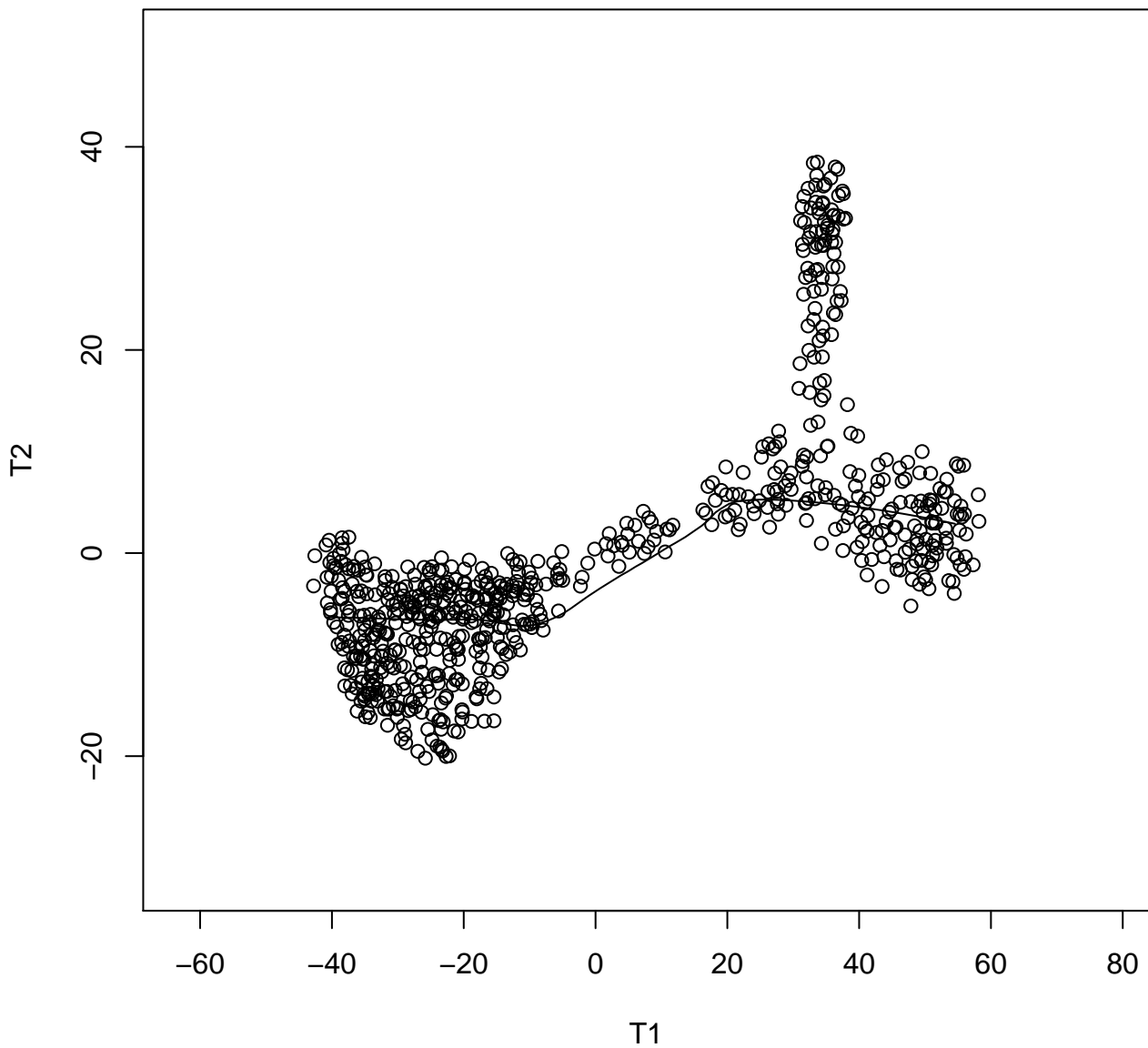

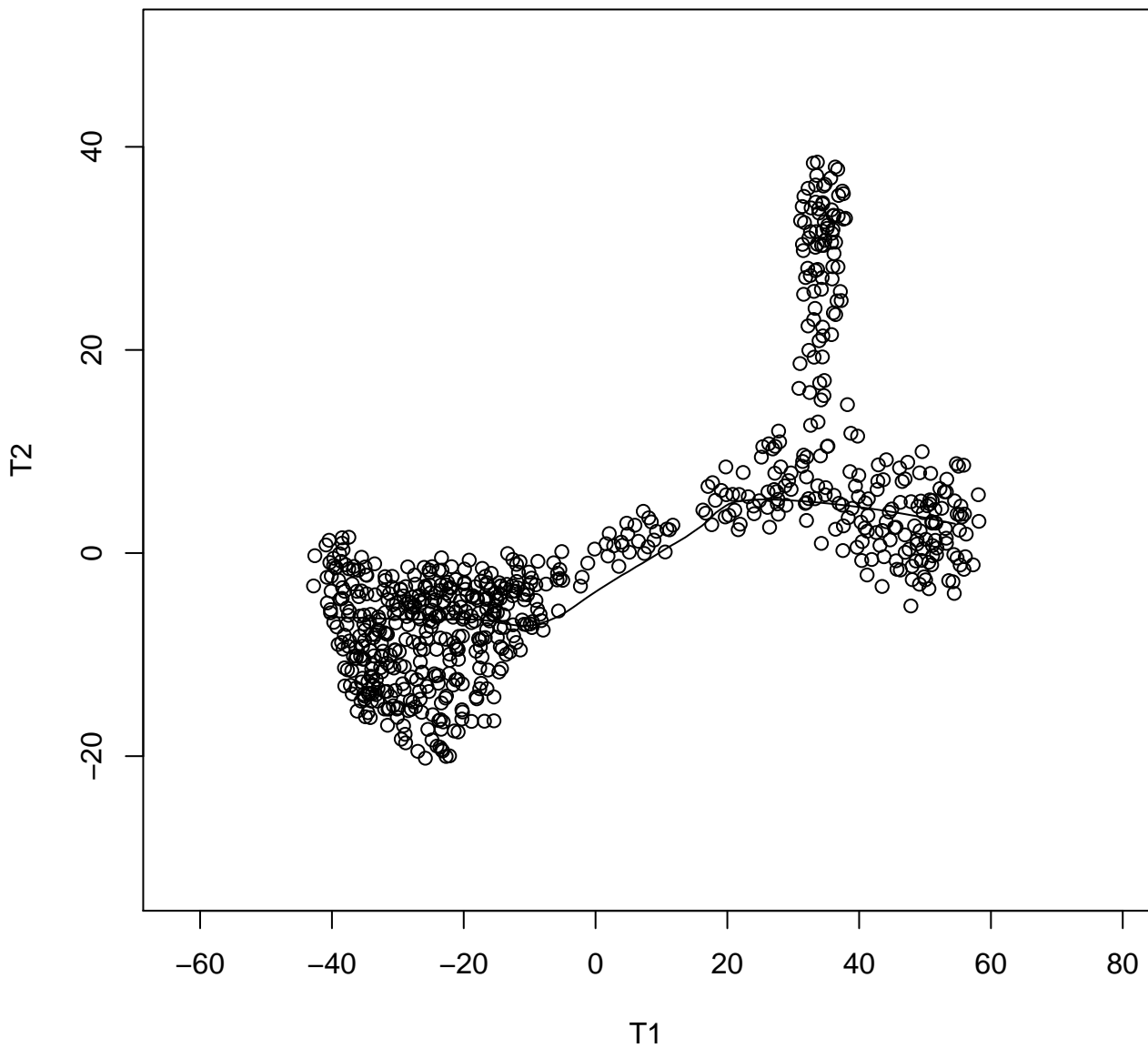

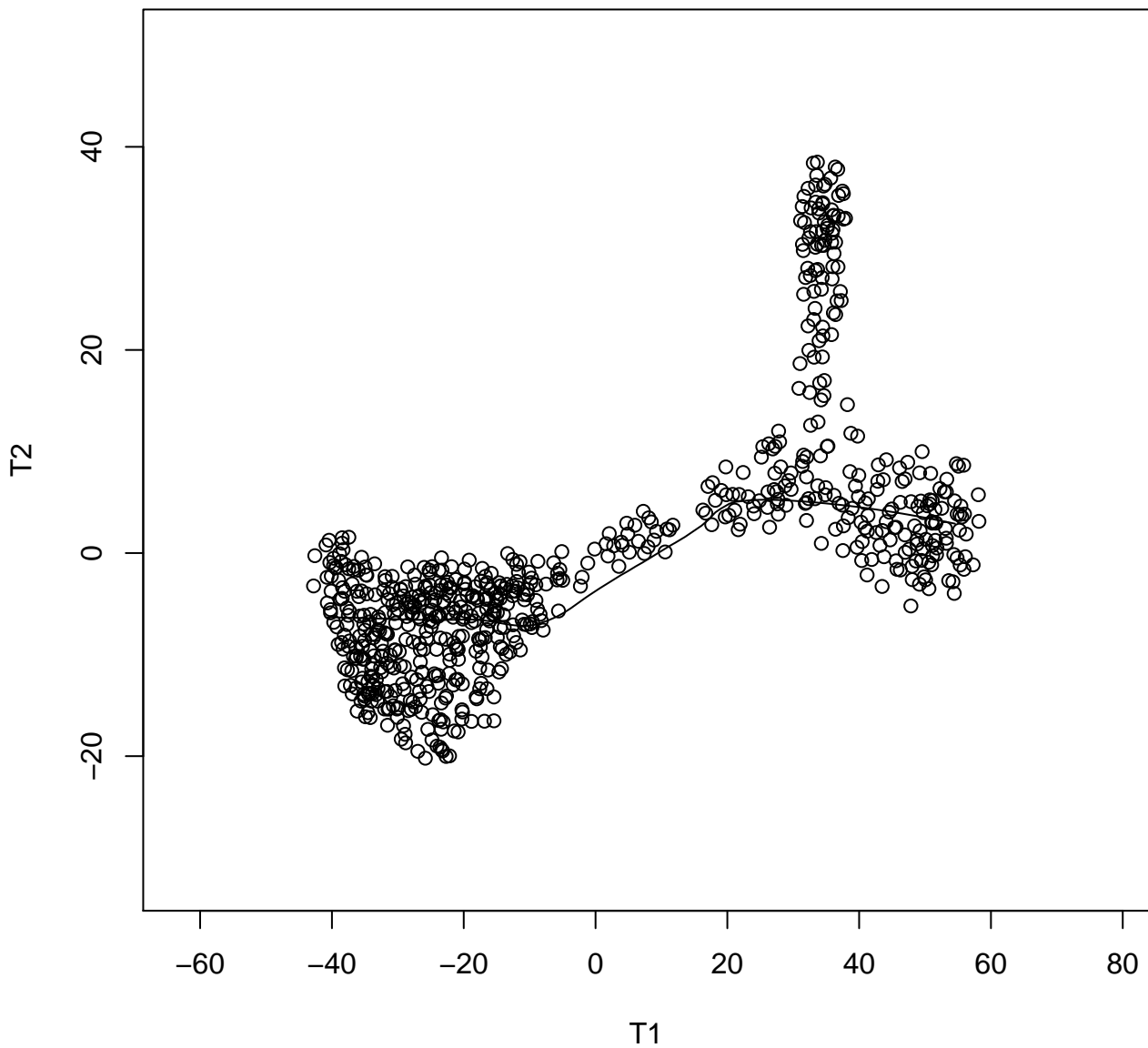

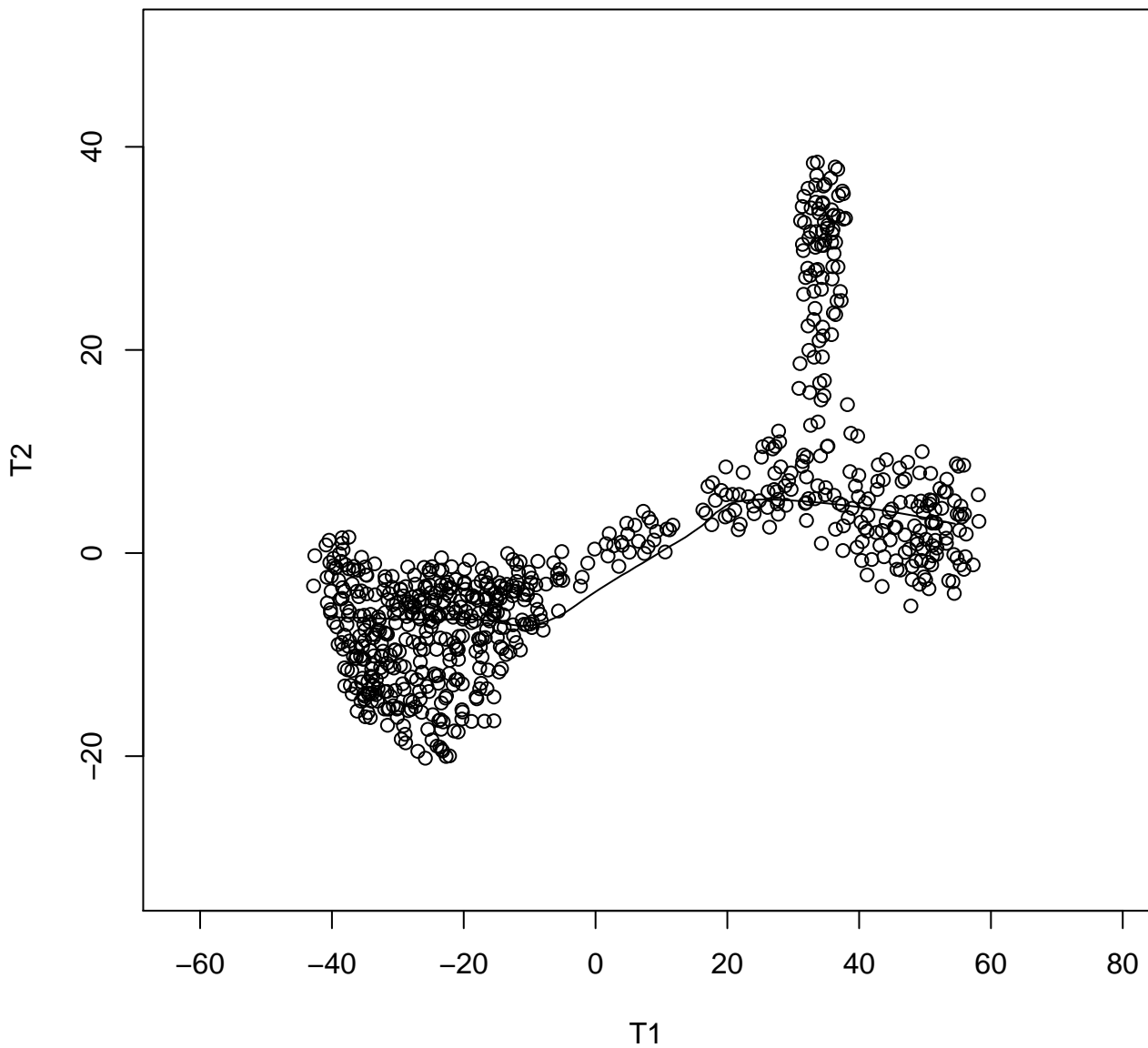

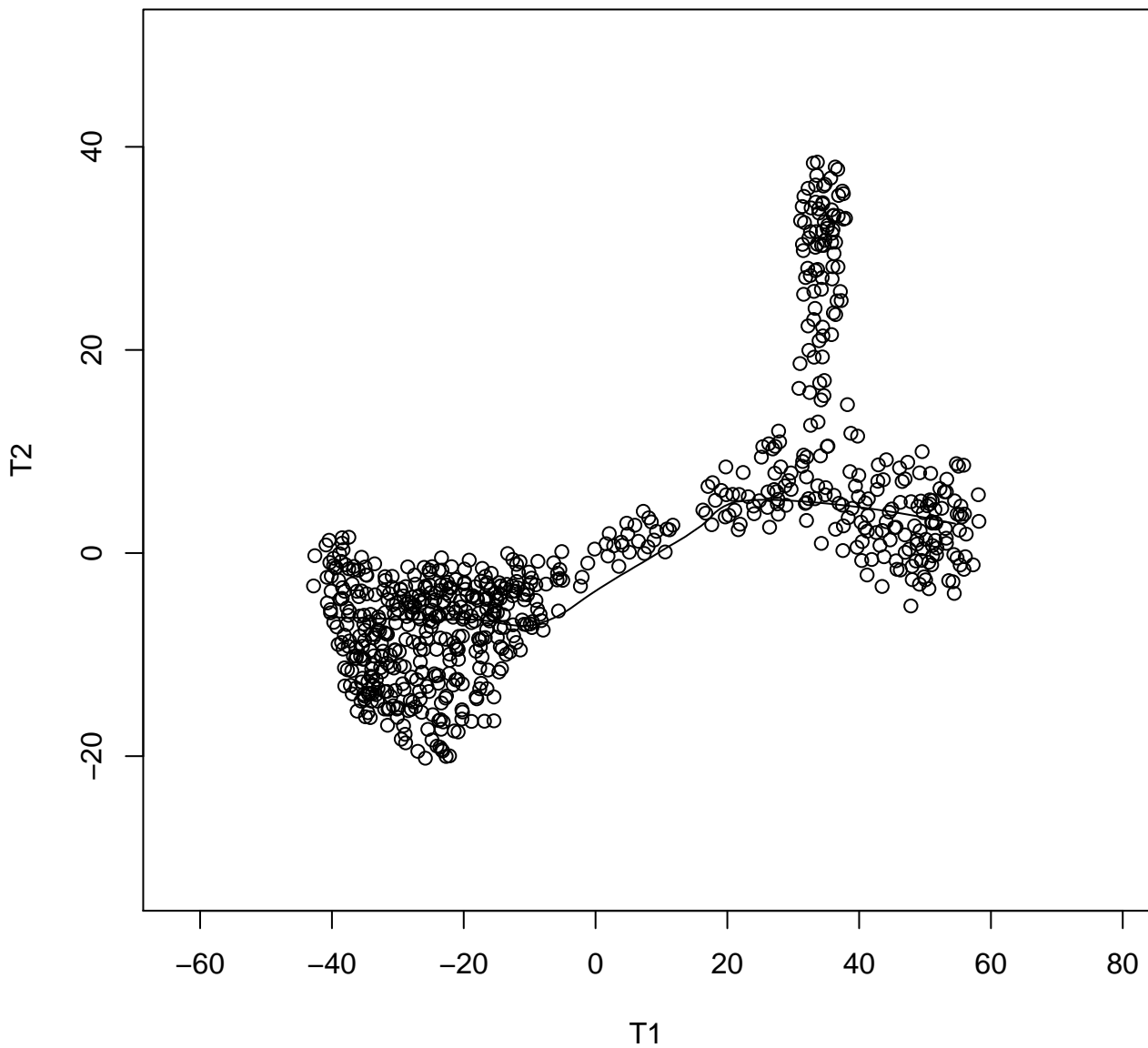

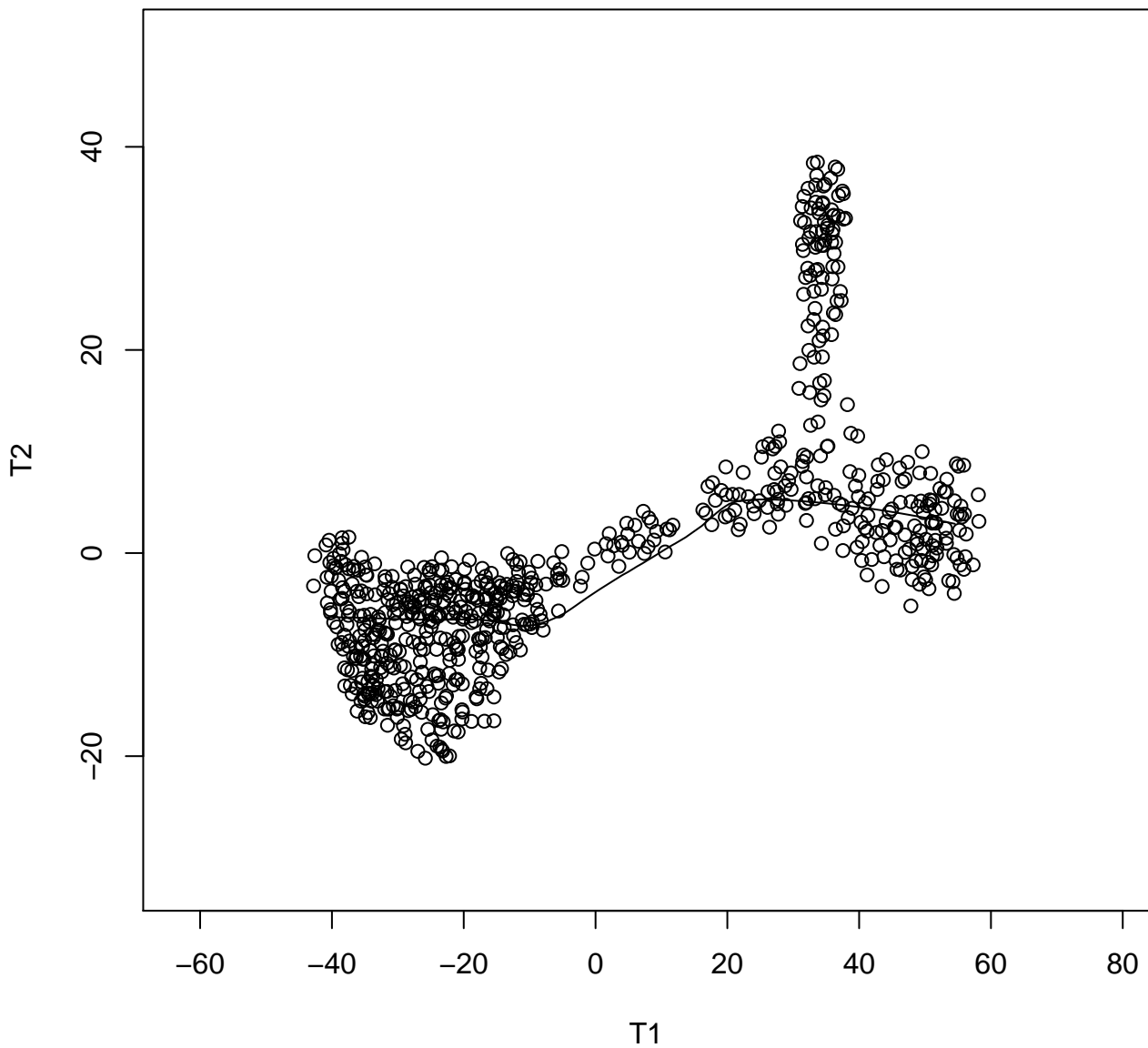

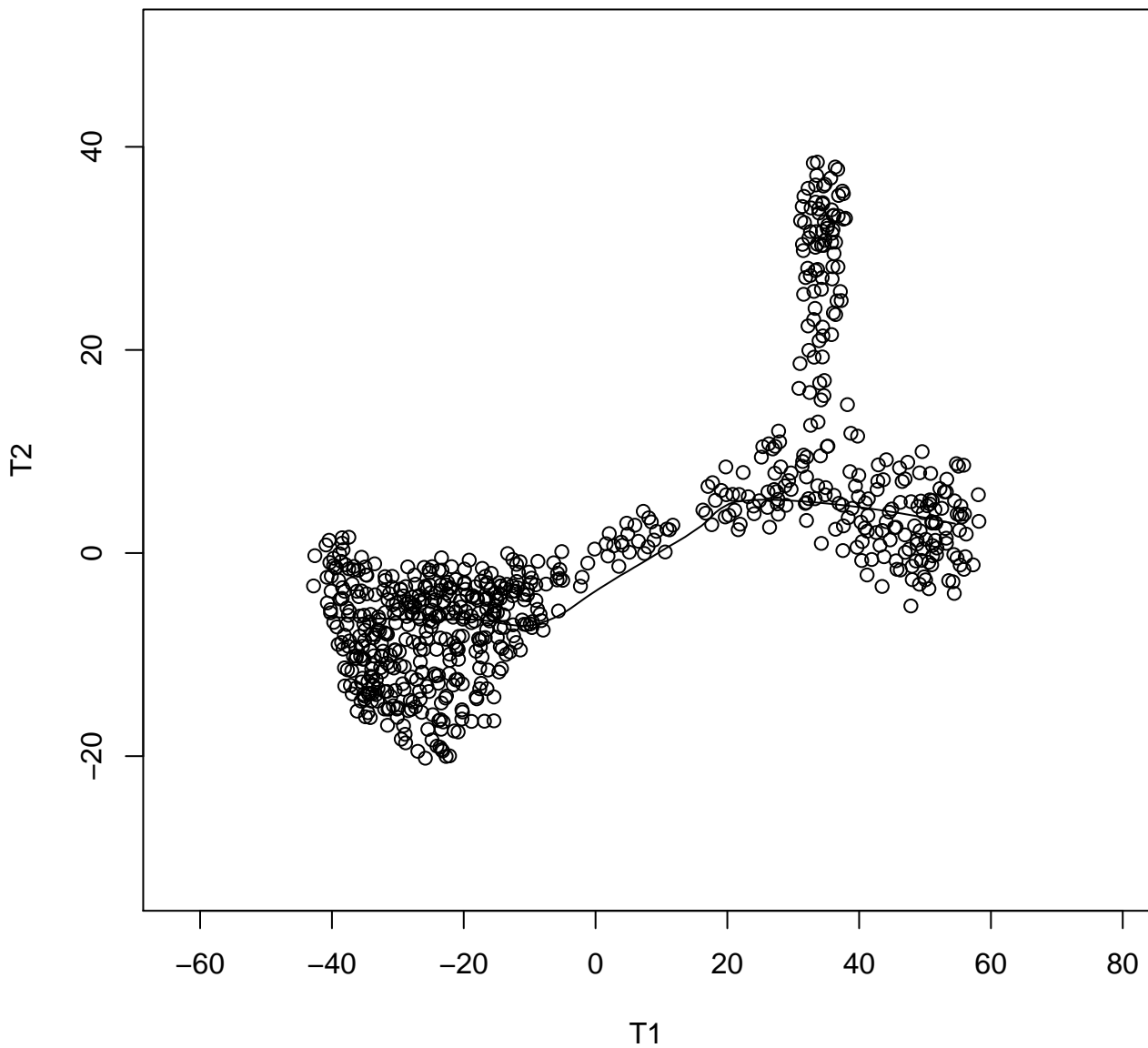

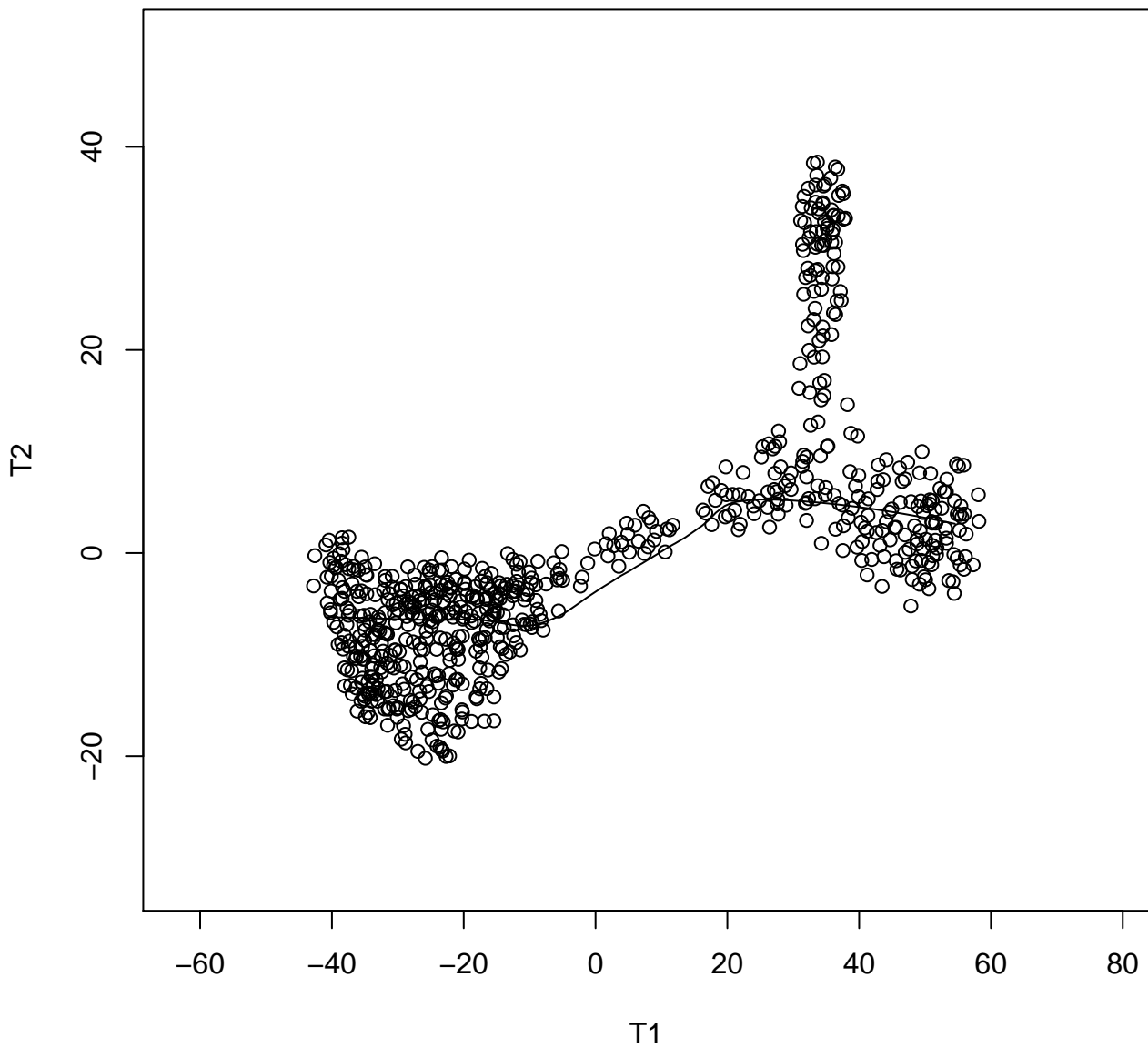

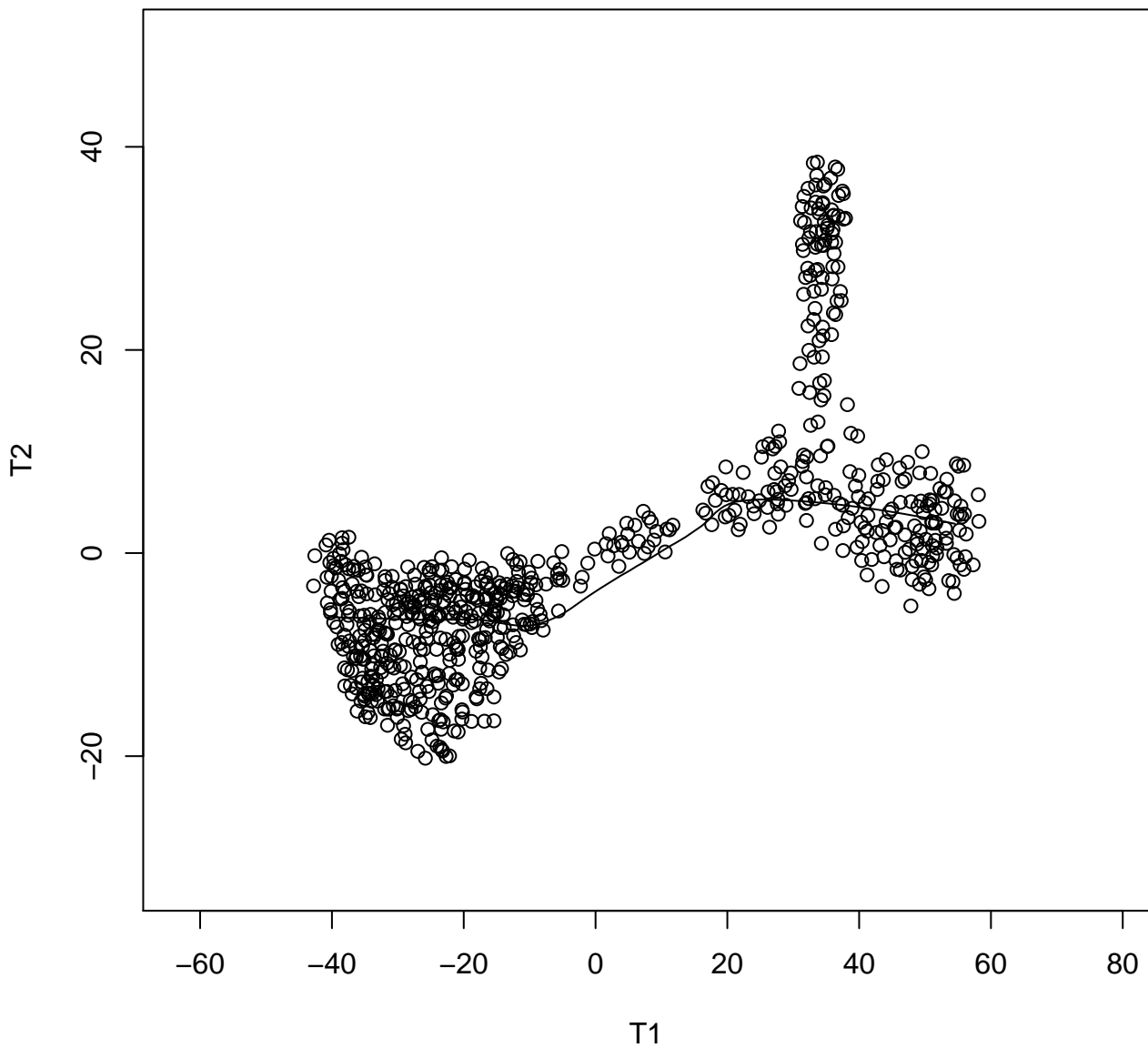

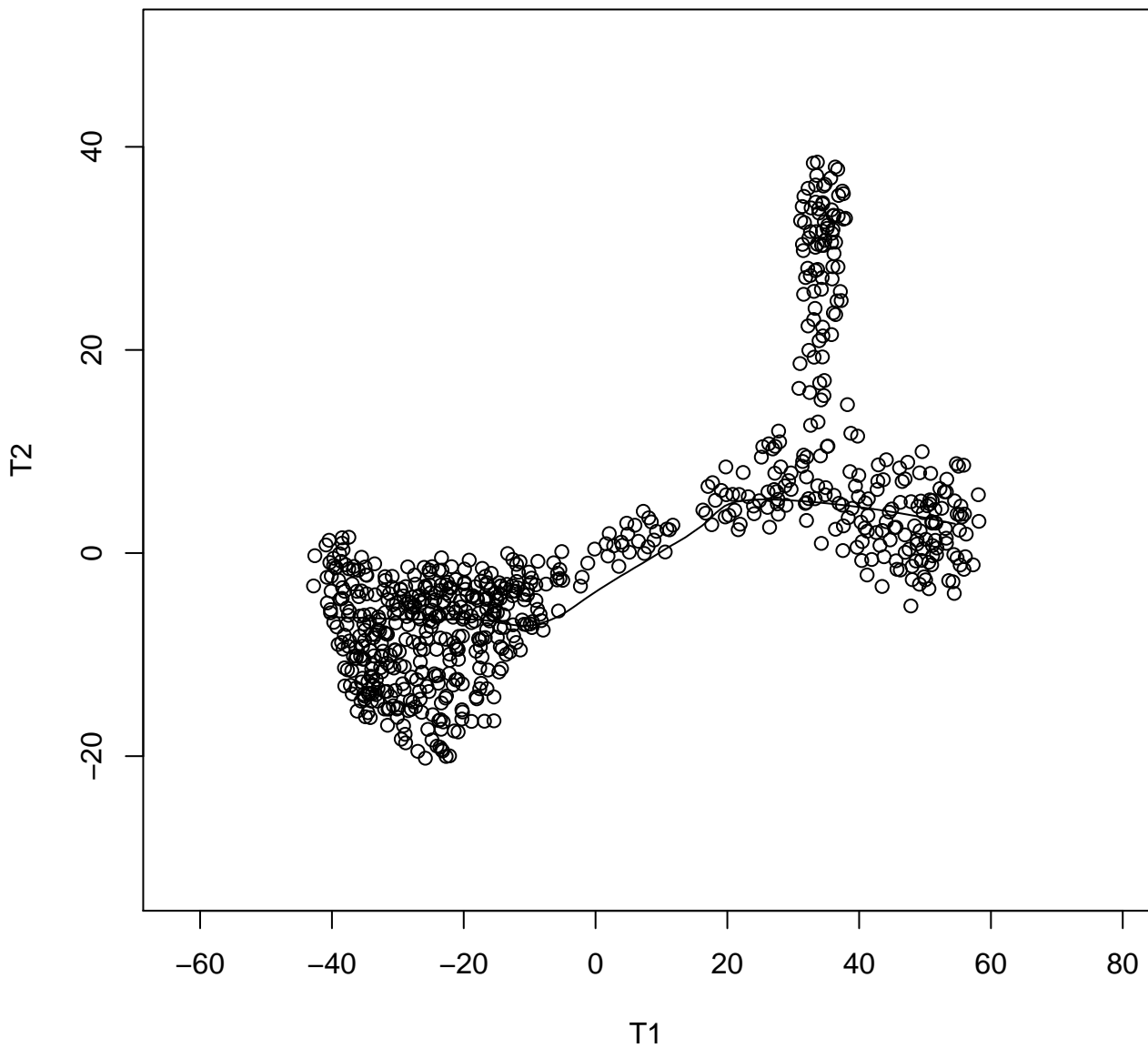

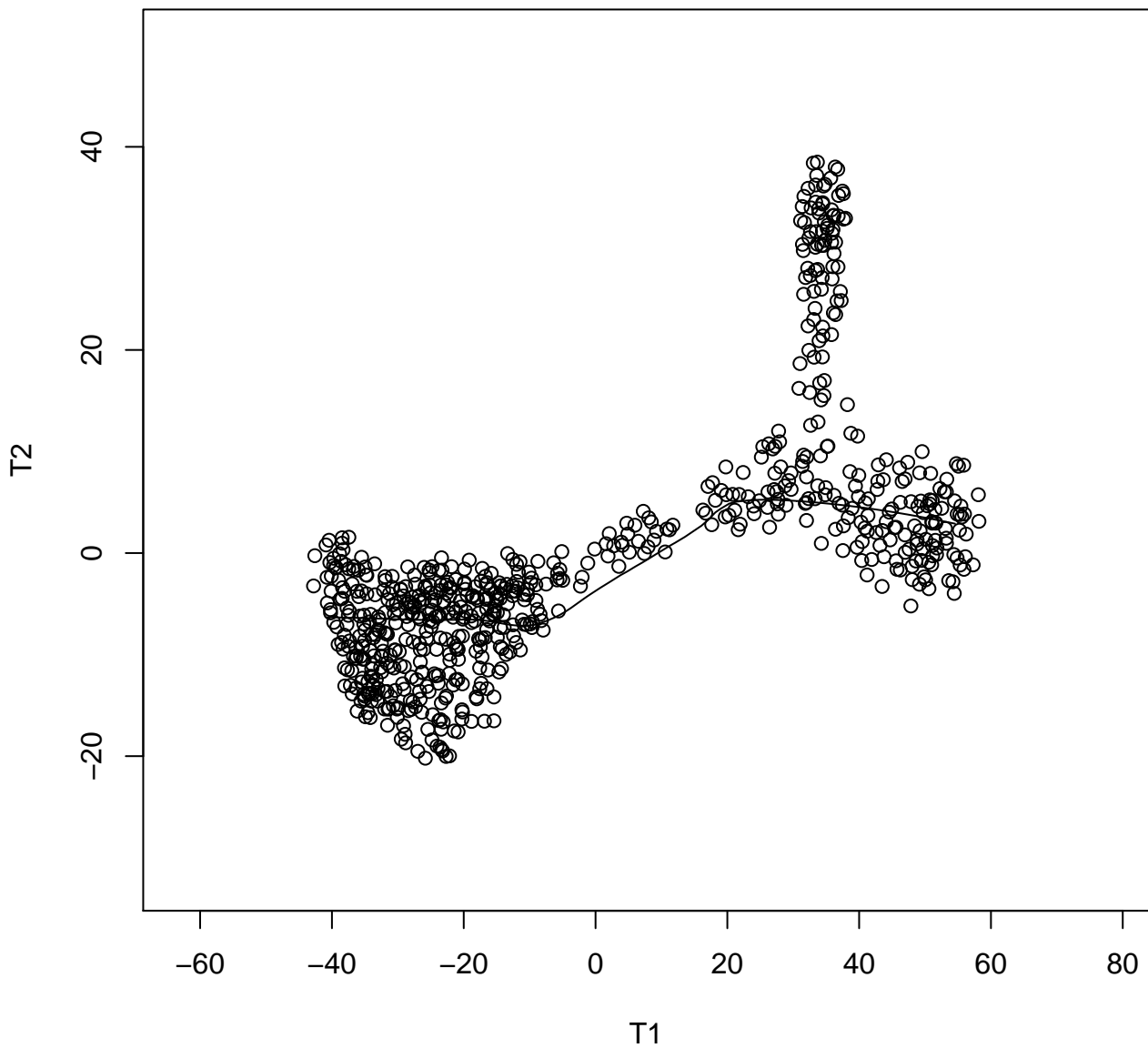

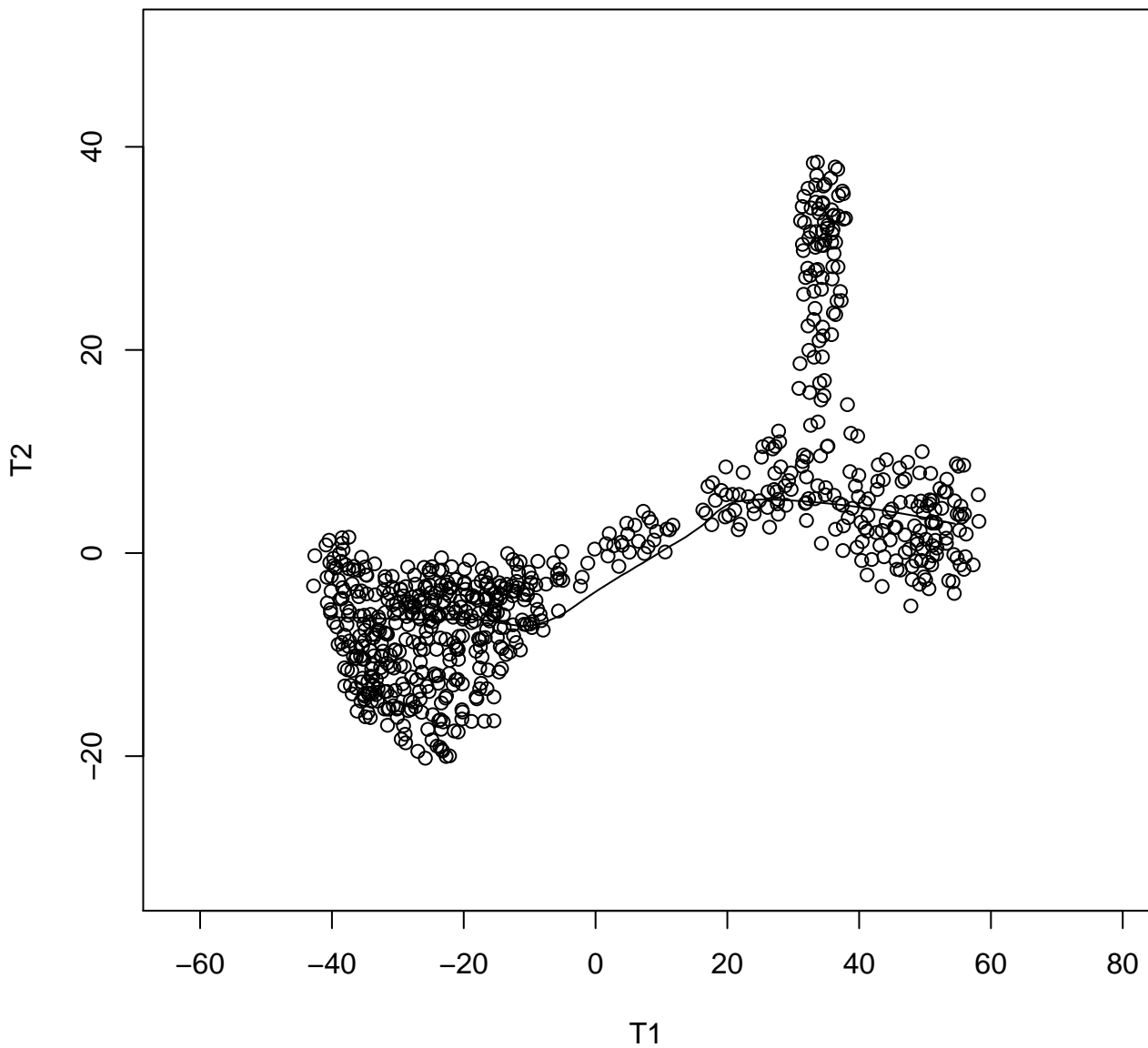

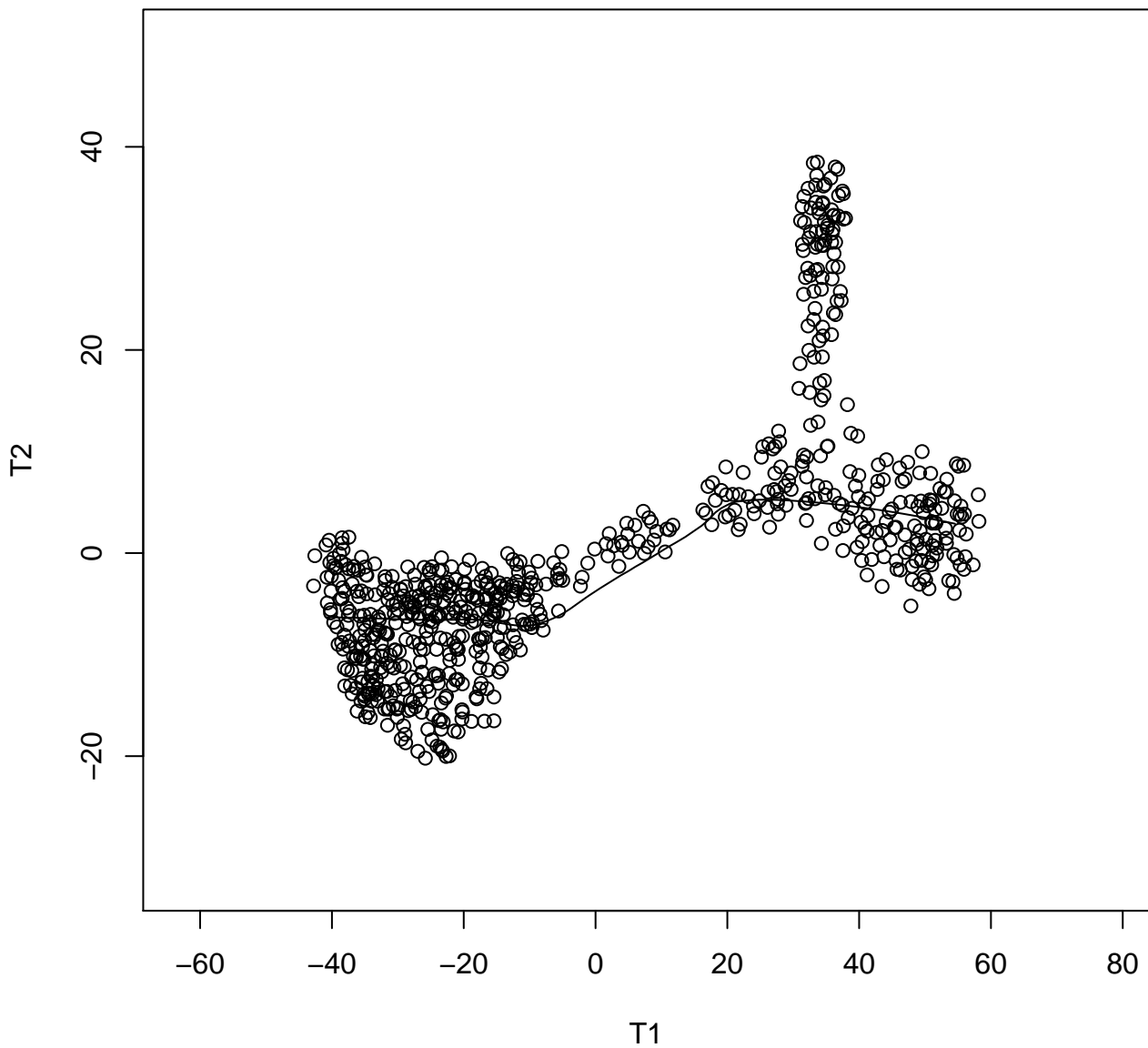

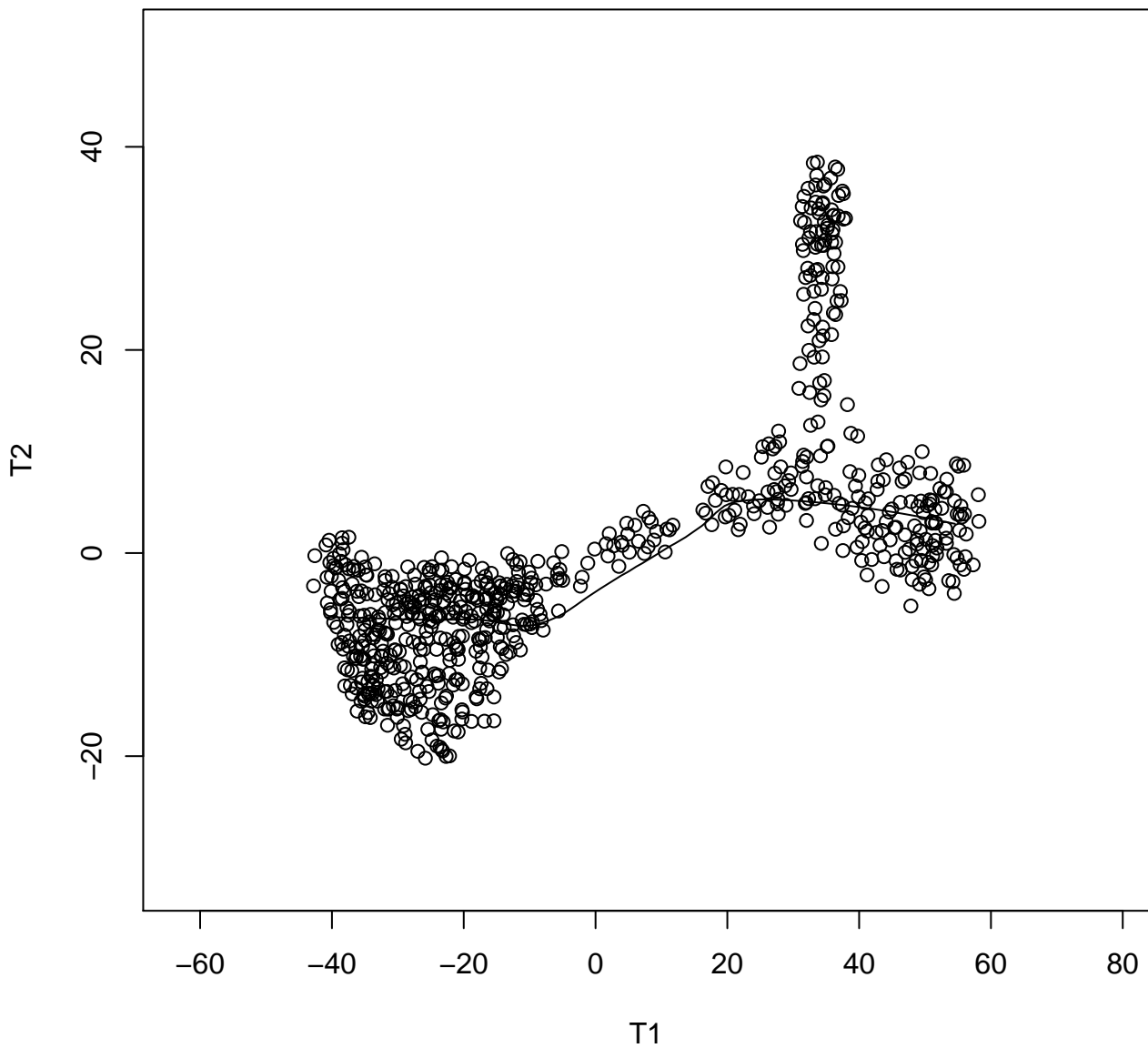

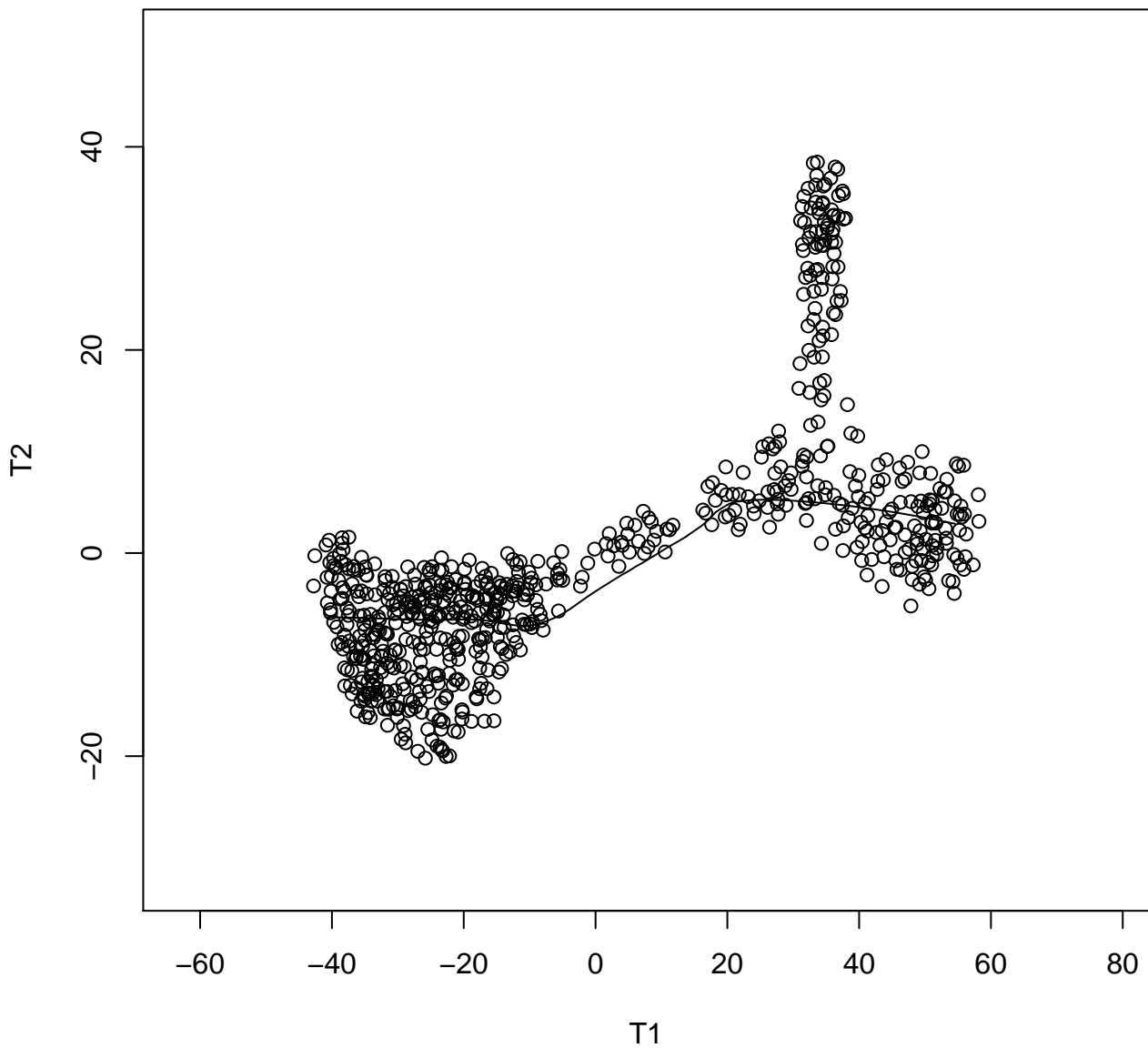

Supplement: Supplementary file 5 — Supplementary Data 2 [file 41467_2019_9670_MOESM5_ESM.zip › Sup_data2/Synthetic/scuba/Result_run1/Rplots.pdf]

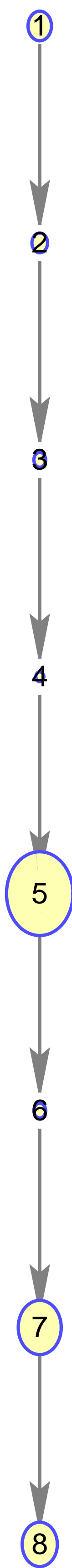

Supplement: Supplementary file 5 — Supplementary Data 2 [file 41467_2019_9670_MOESM5_ESM.zip › Sup_data2/Synthetic/scuba/sample_data/simulation/figures/tree.pdf]
