# Supplementary material for: Single-cell trajectories reconstruction, exploration and mapping of omics data with STREAM
Source: Nat Commun. 2019 Apr 23;10:1903. doi: 10.1038/s41467-019-09670-4 (PMC6478907; doi:10.1038/s41467-019-09670-4)
Supplement: Supplementary file 5 — Supplementary Data 2 [file 41467_2019_9670_MOESM5_ESM.zip › Sup_data2/Synthetic/scuba/Rplots.pdf]

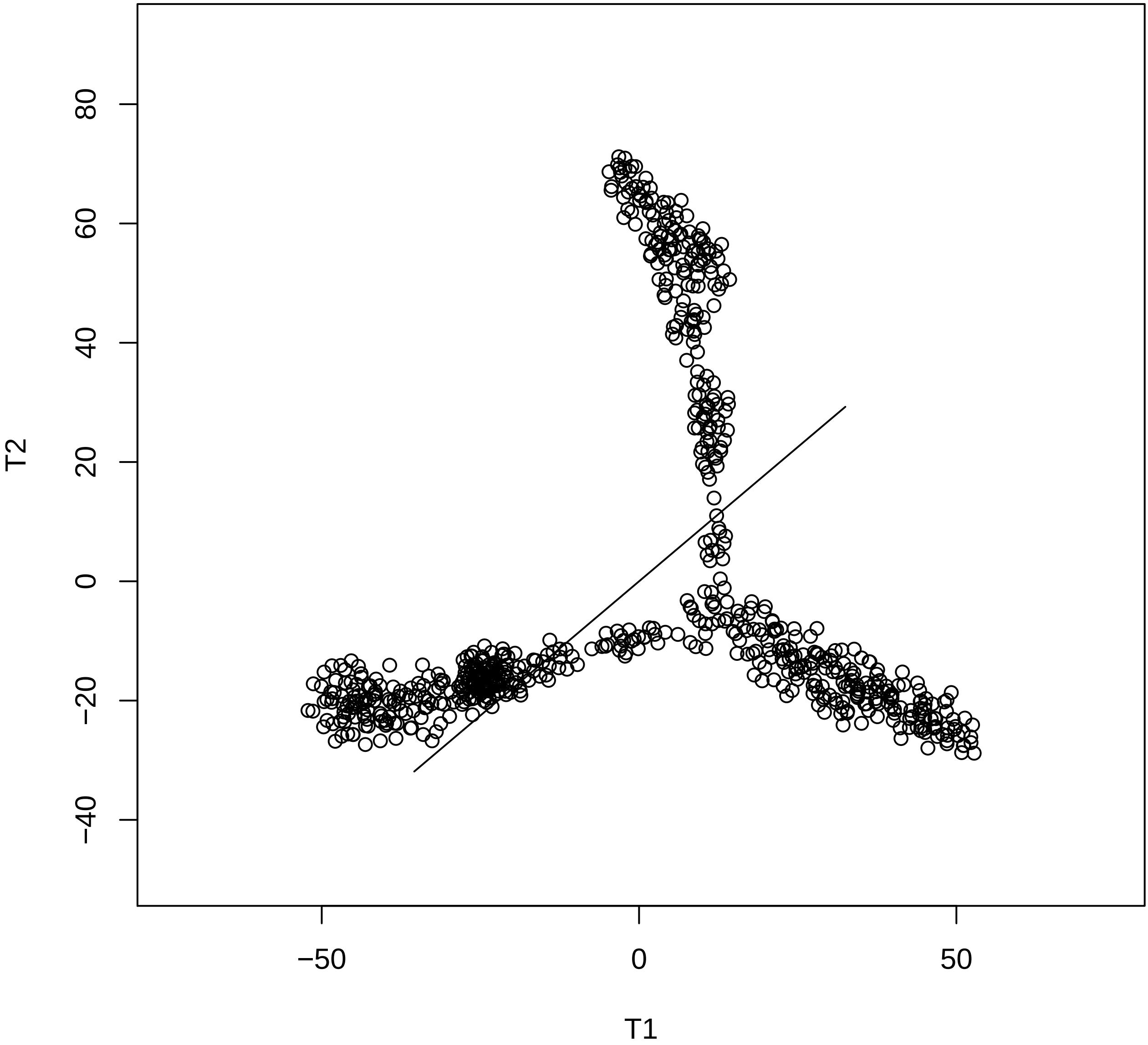

T2
80
60
40
20
0
−20
−40
−50
0
50
T1

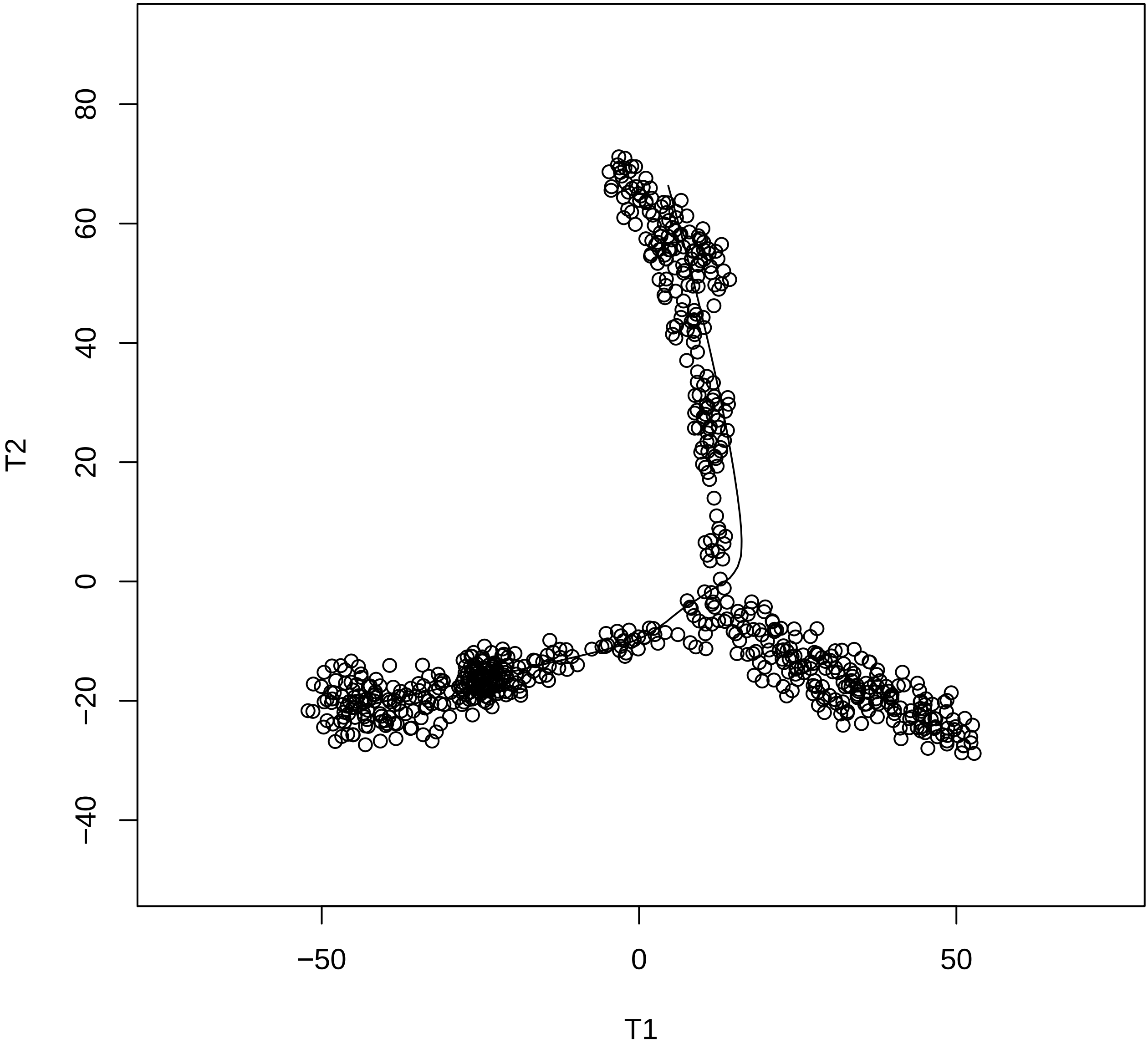

T2
80
60
40
20
0
−20
−40
−50
0
50
T1

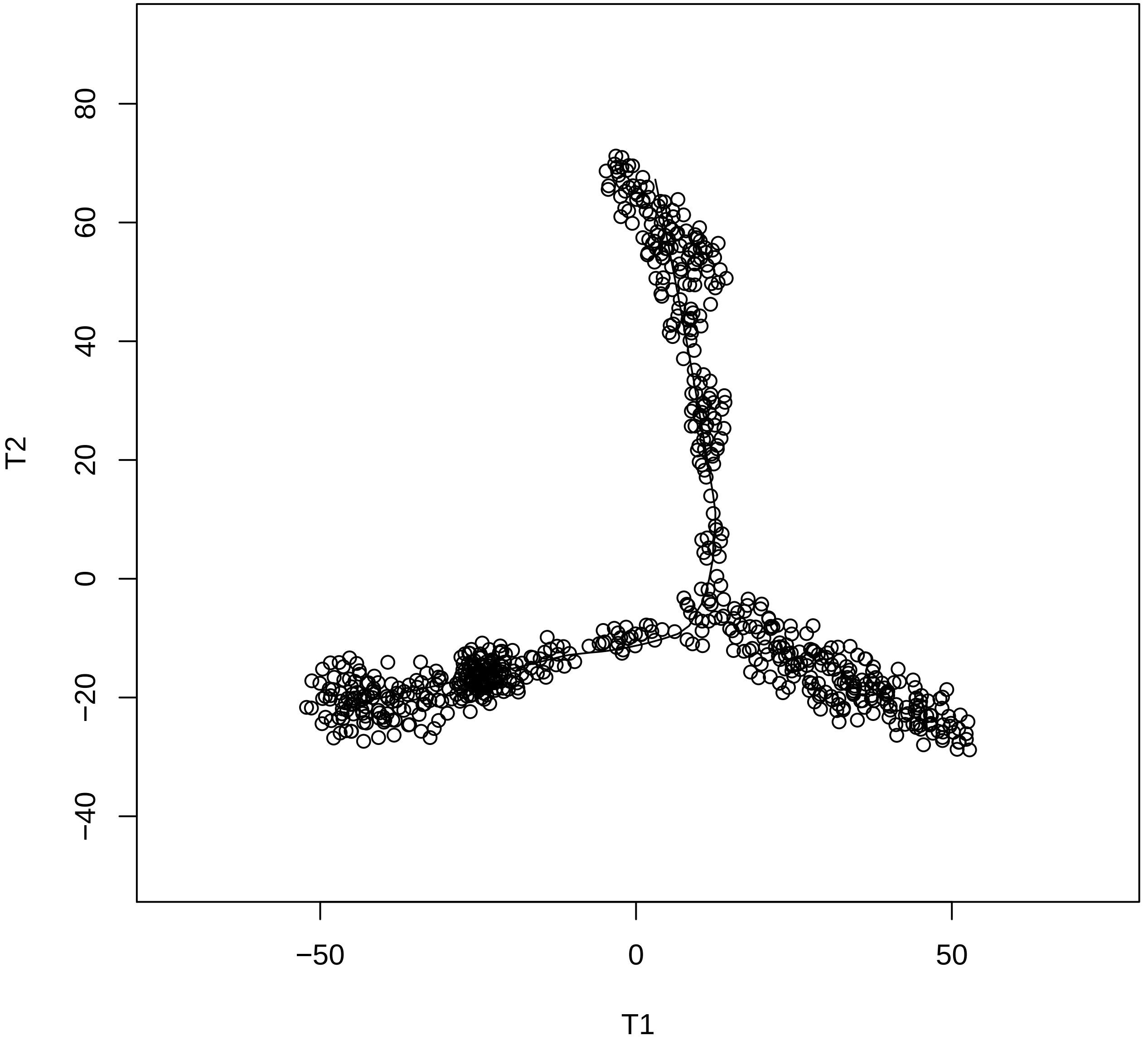

T1
T2
−50
0
50
−40
−20
0
20
40
60
80

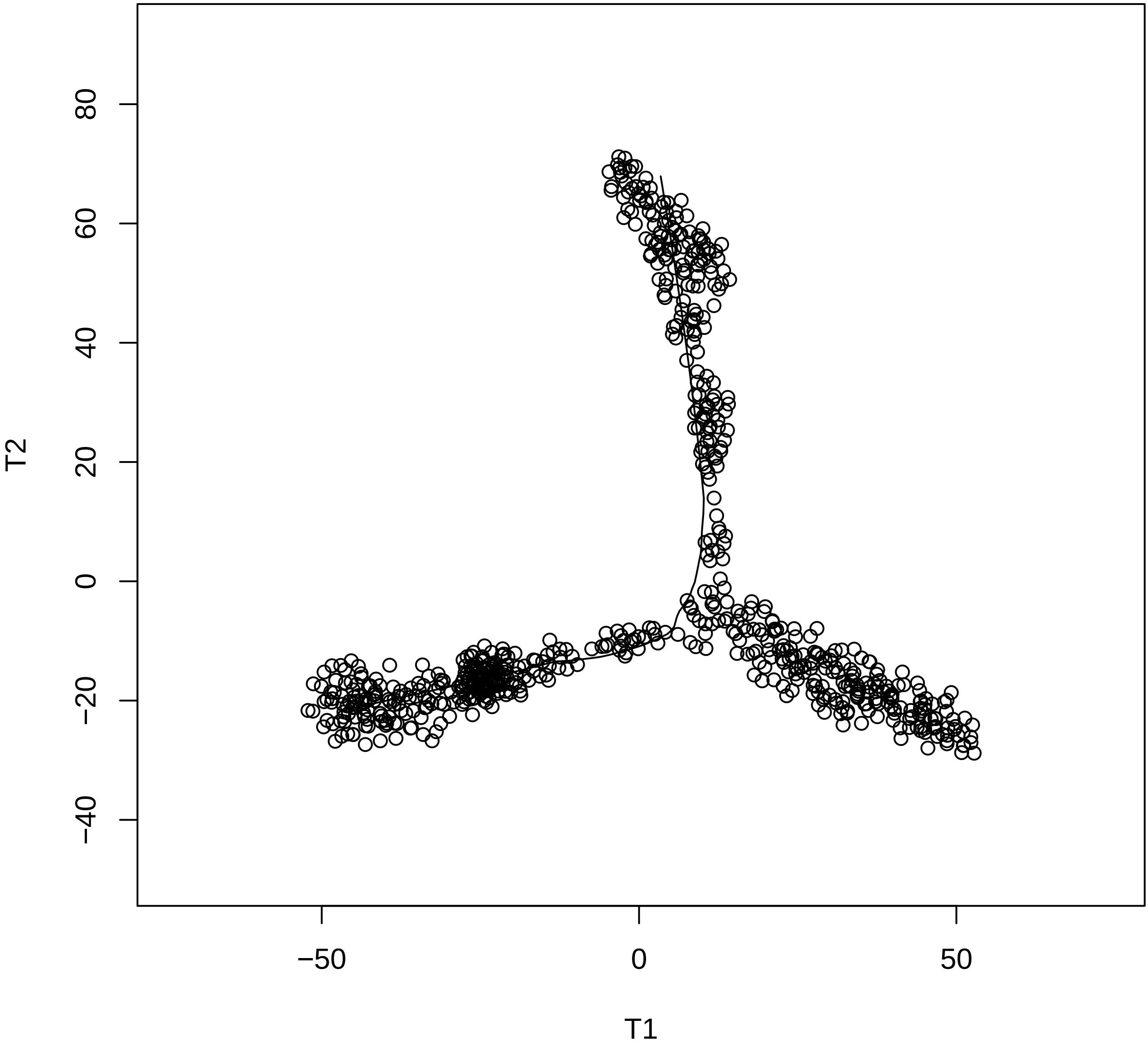

80
60
40
20
0
−20
−40
T2
−50
0
50
T1

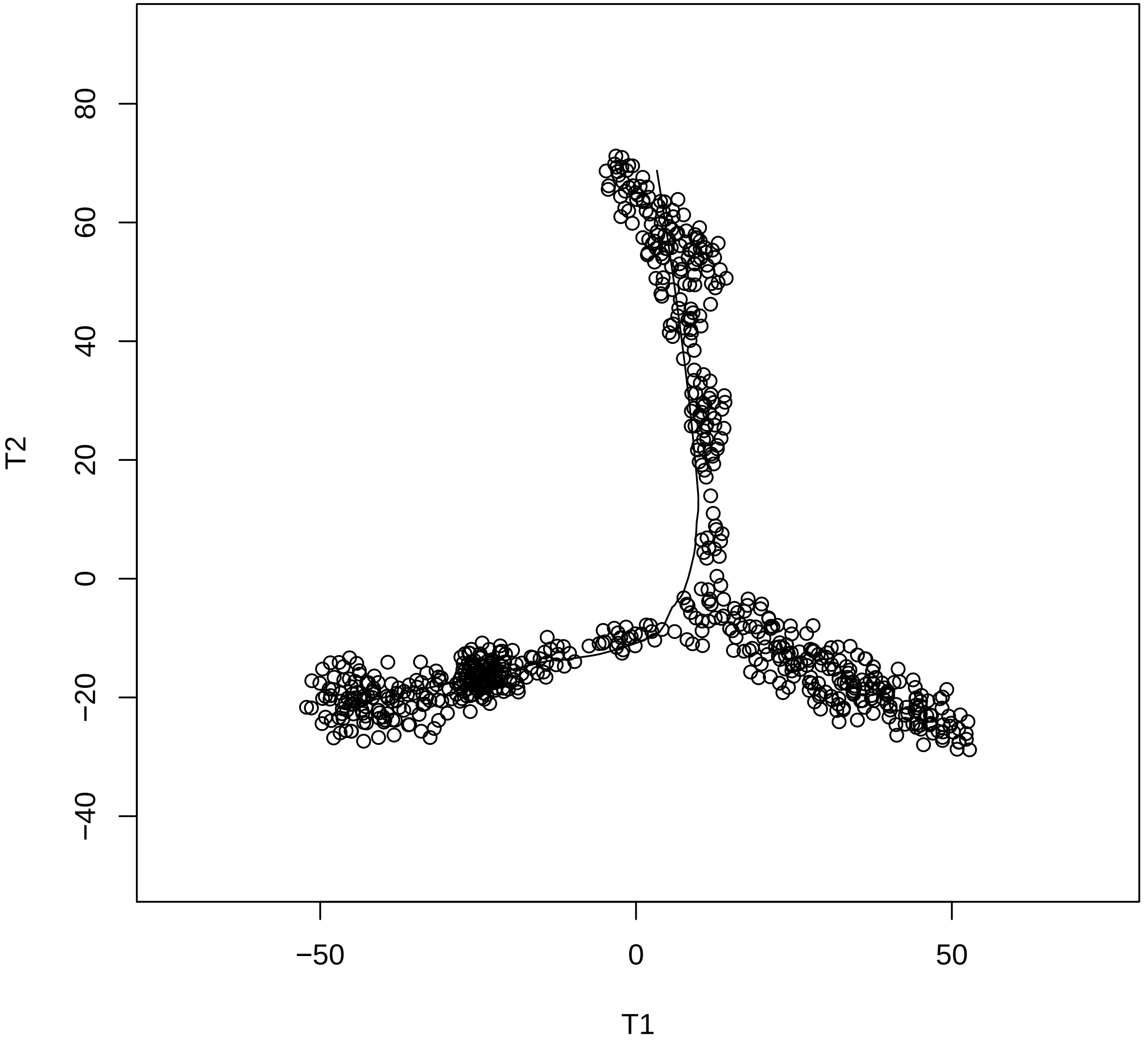

T2
80
60
40
20
0
−20
−40
−50
0
50
T1

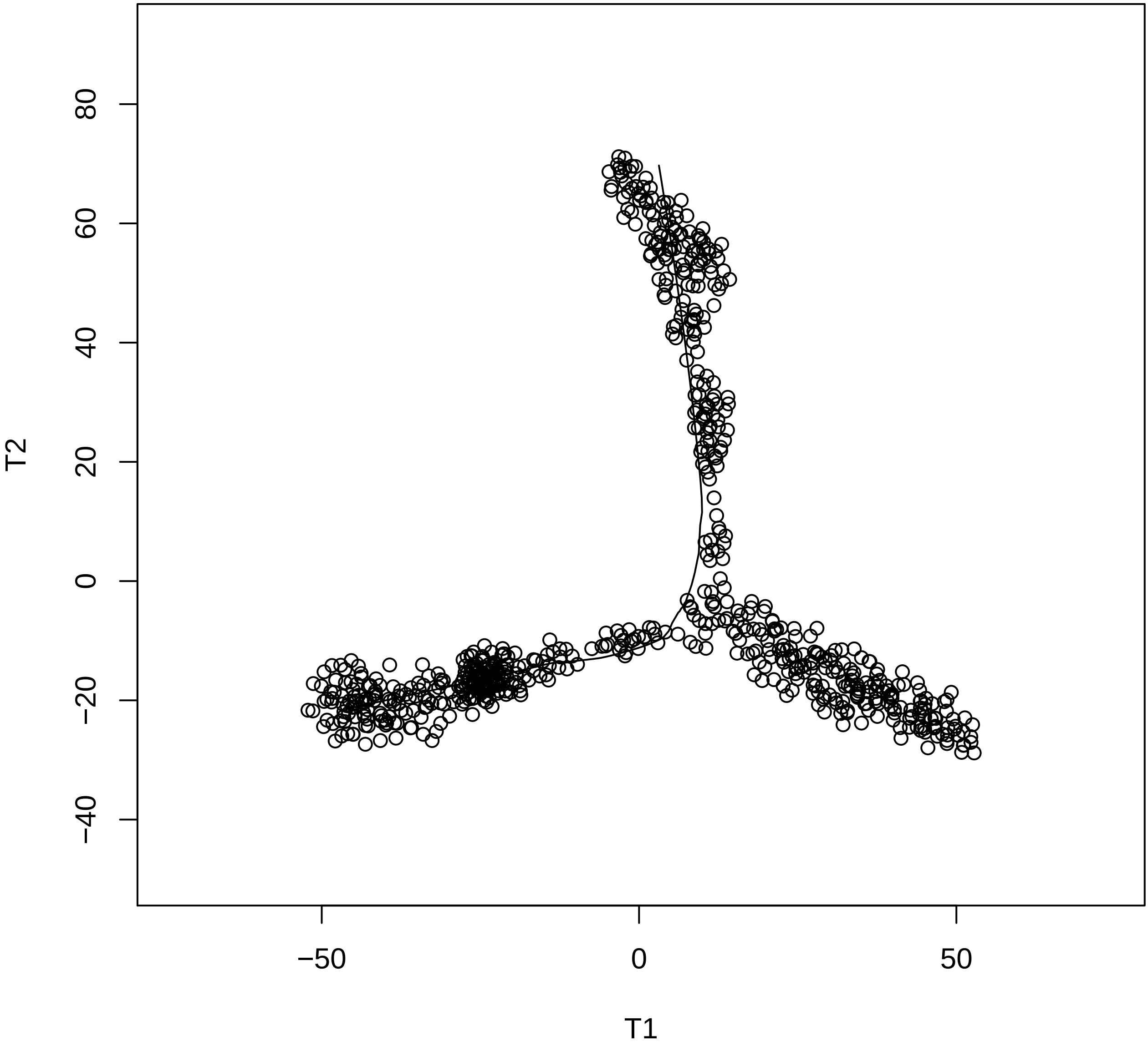
T2
80
60
40
20
0
−20
−40
T1
−50
0
50

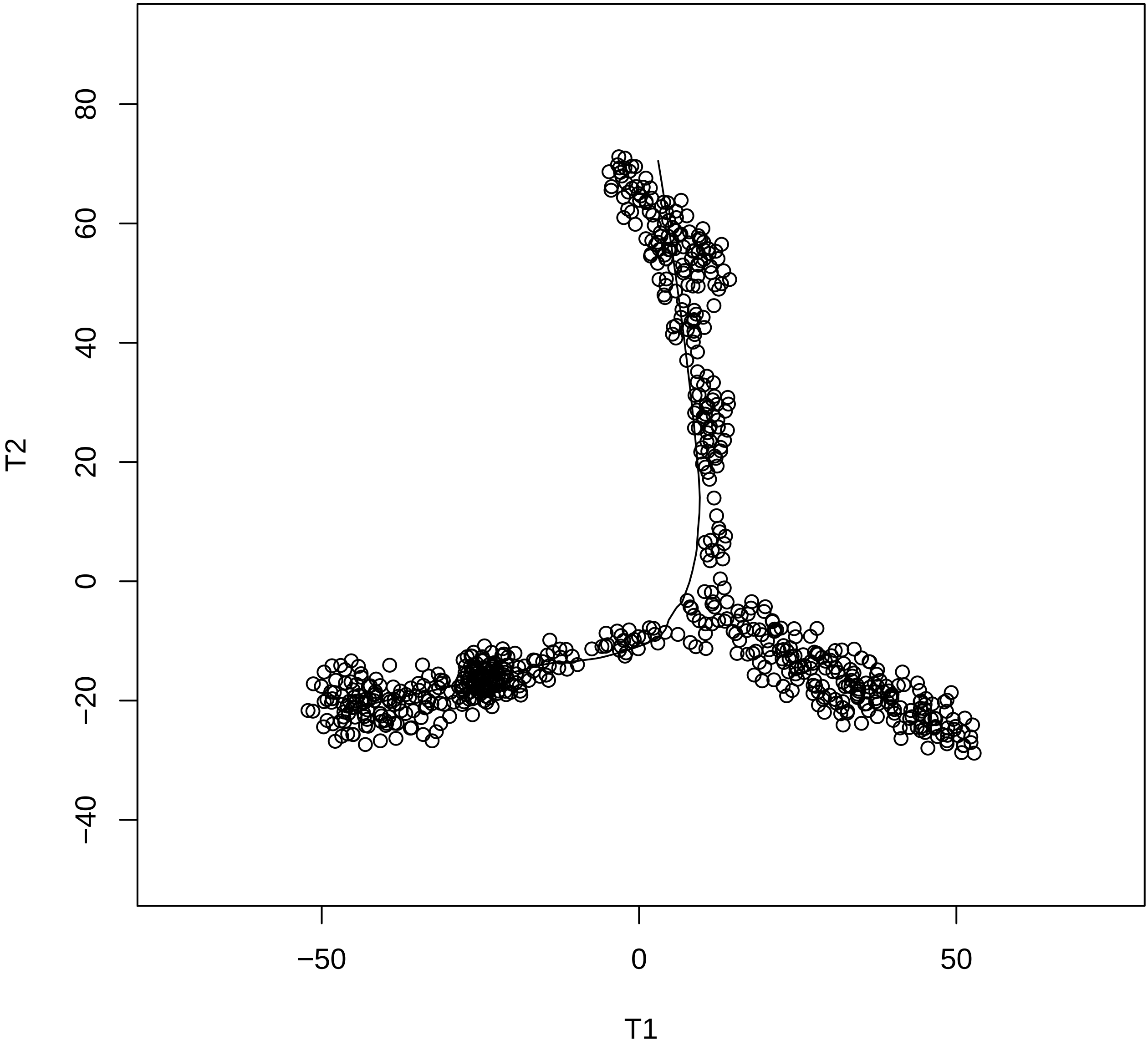

80
60
40
20
0
−20
−40
T2
−50
0
50
T1

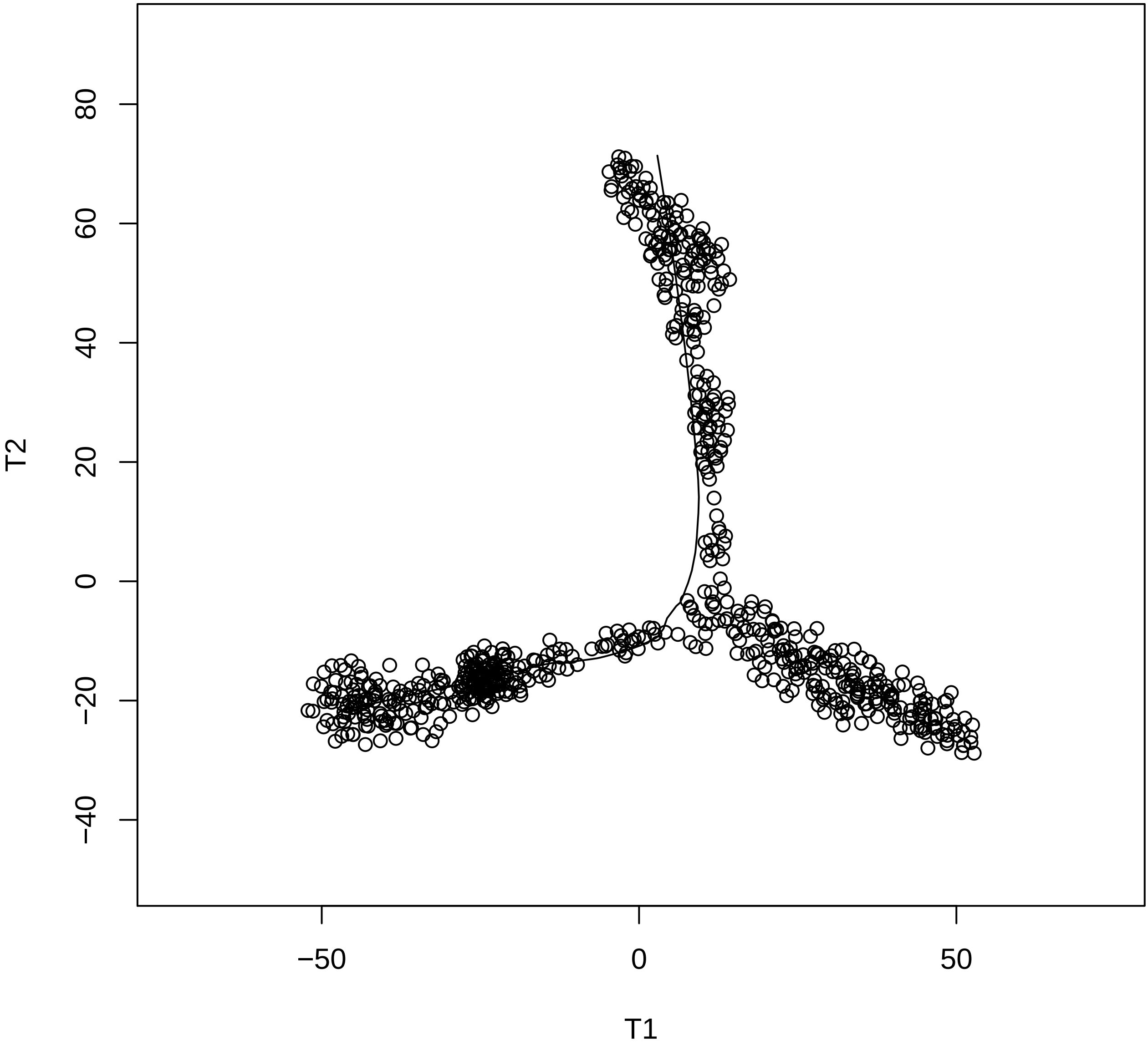

80
60
40
20
0
−20
−40
T2
−50
0
50
T1

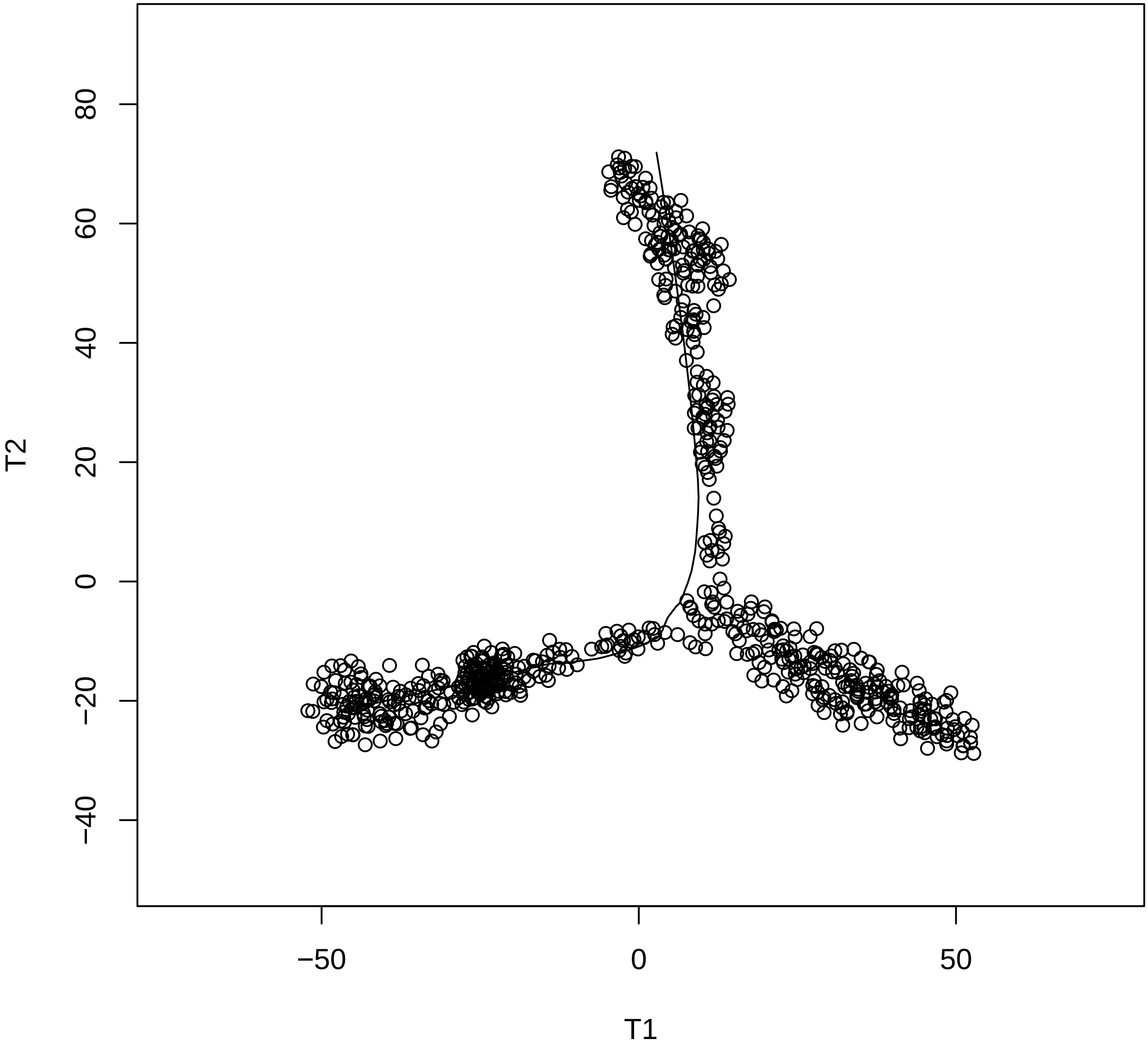

80
60
40
20
0
−20
−40
T2
−50
0
50
T1

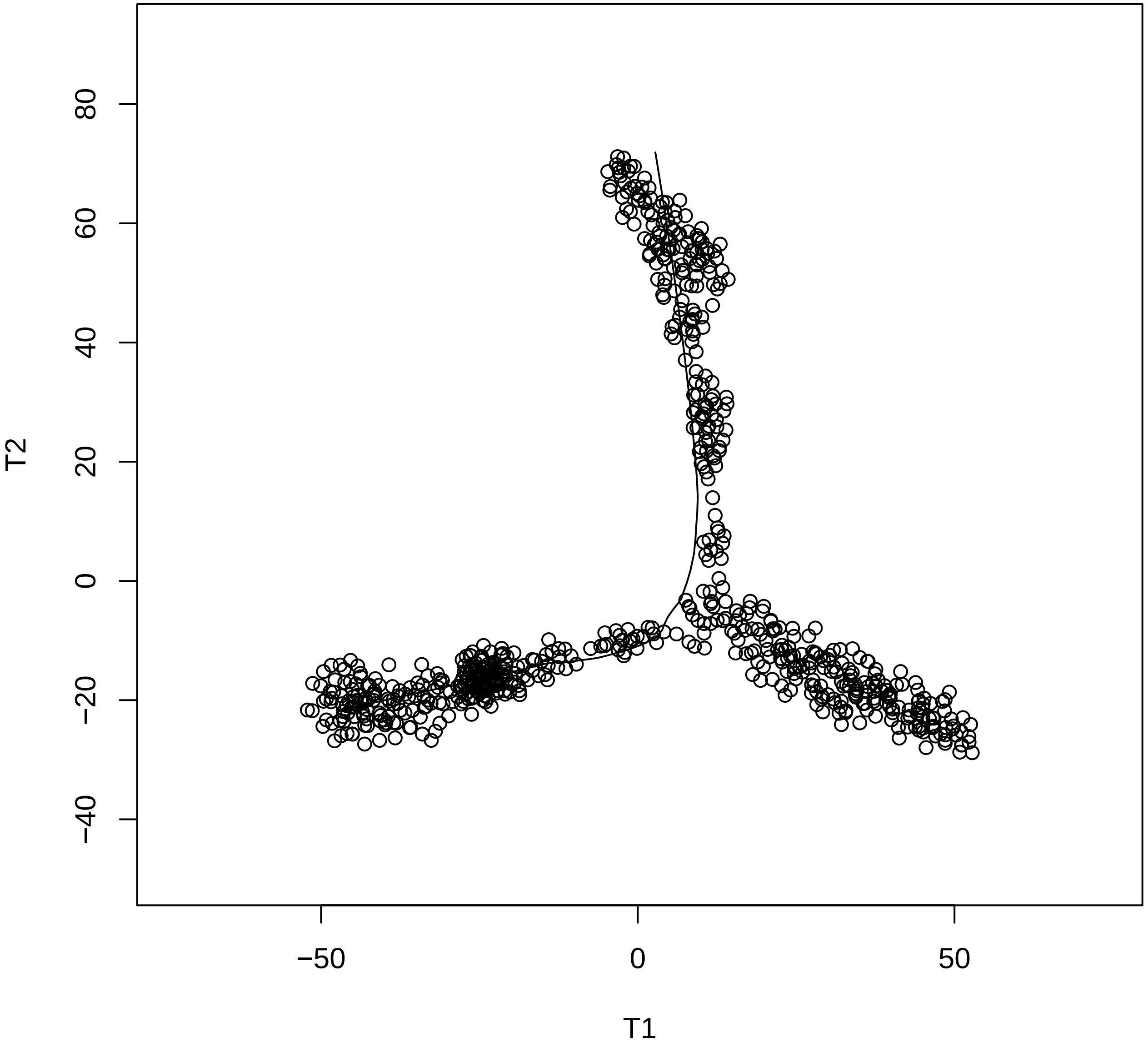

80
60
40
20
0
−20
−40
T2
−50
0
50
T1

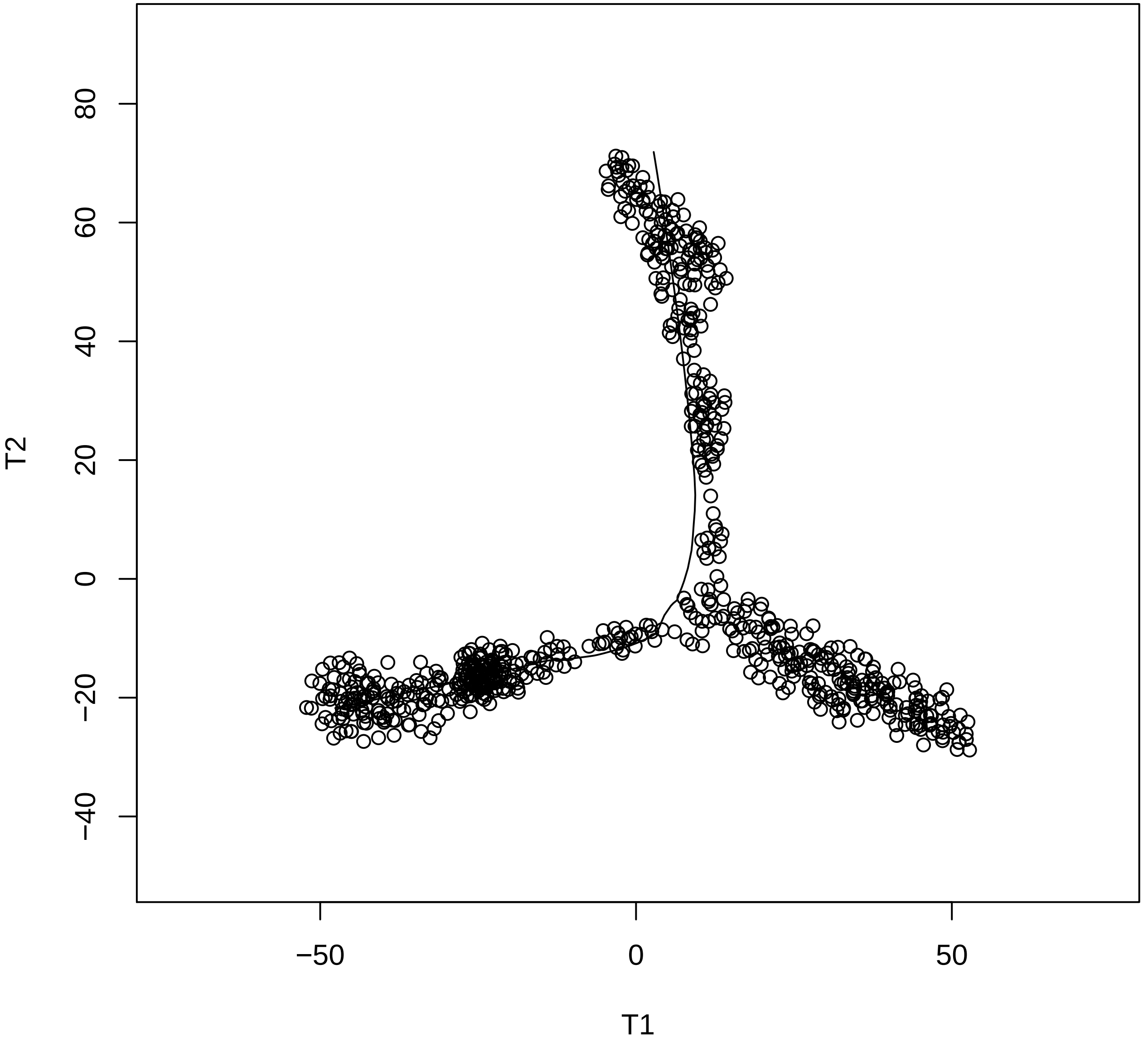
80
60
40
20
0
−20
−40
T2
−50
0
50
T1

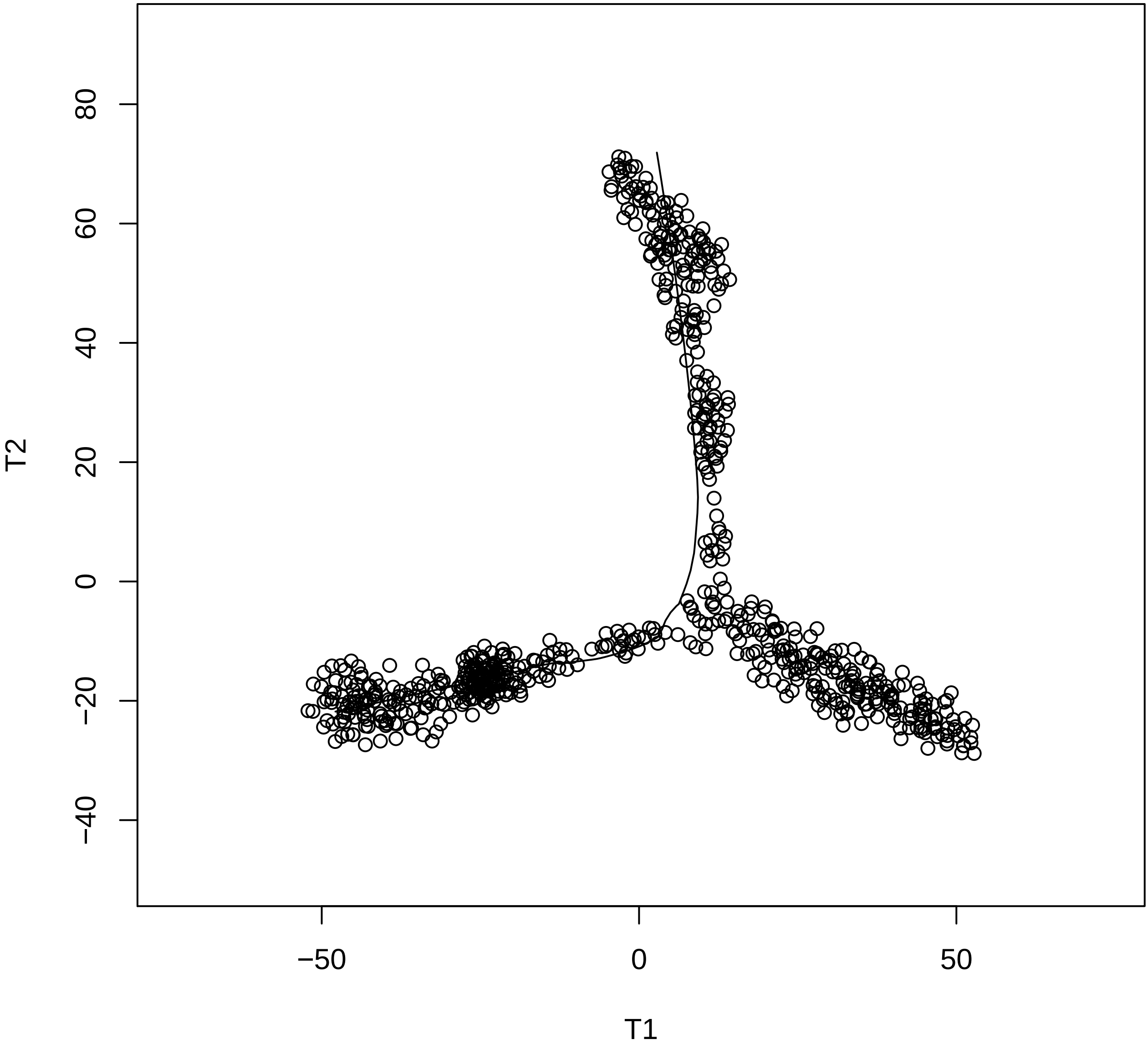

80
60
40
20
0
−20
−40
T2
−50
0
50
T1

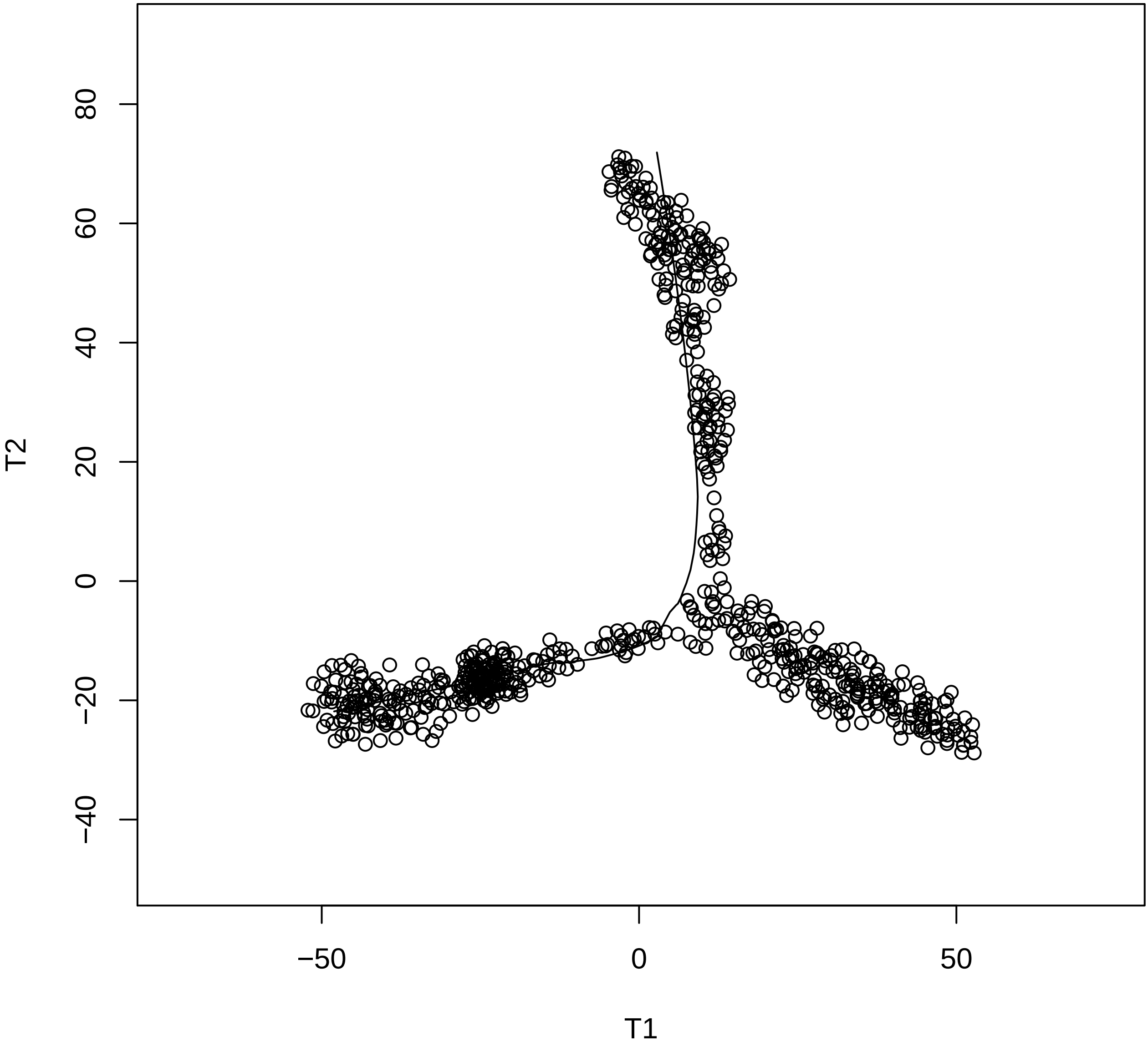

80
60
40
20
0
−20
−40
T2
−50
0
50
T1

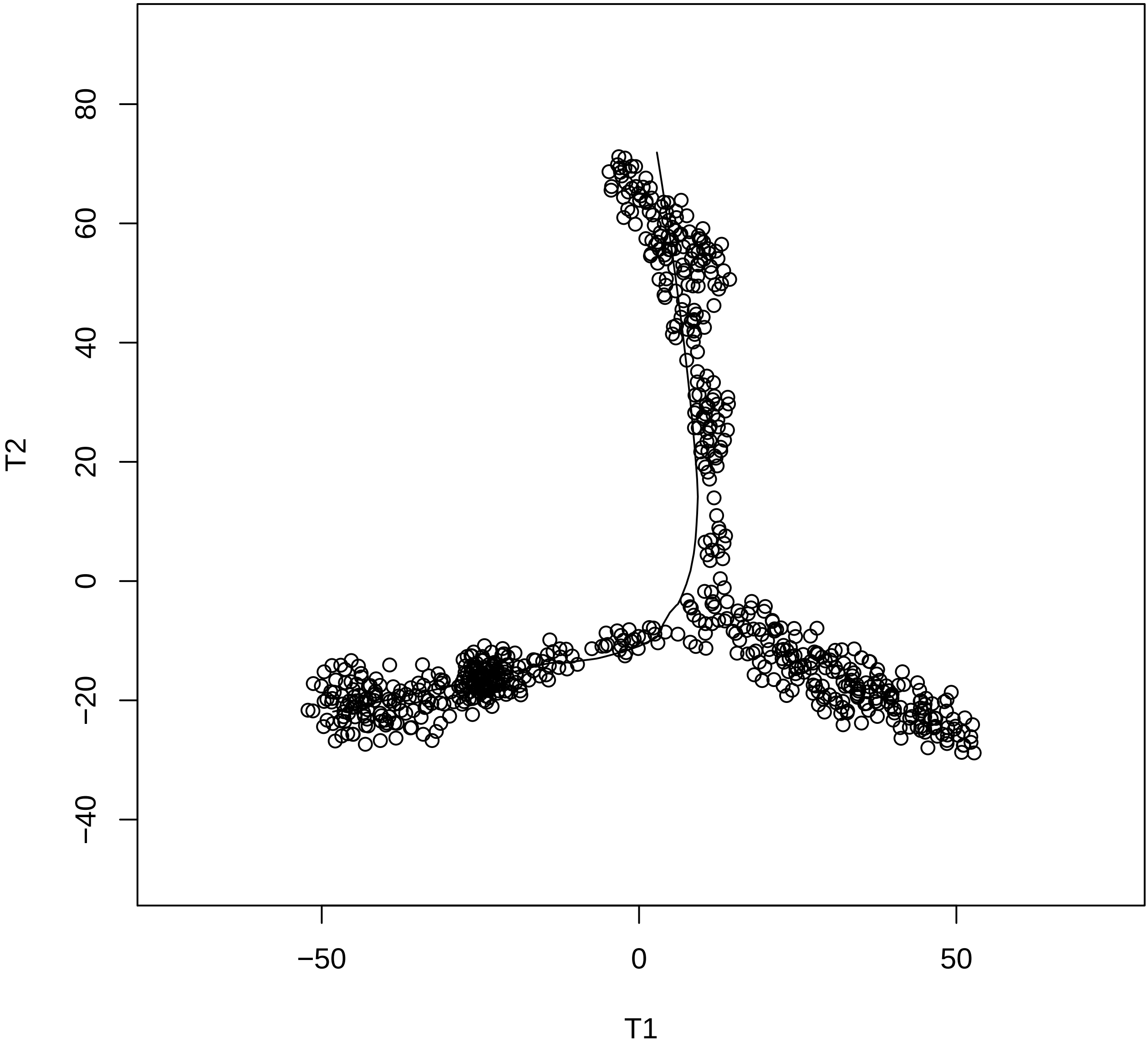

80
60
40
20
0
−20
−40
T2
−50
0
50
T1
