# Supplementary material for: Single-cell trajectories reconstruction, exploration and mapping of omics data with STREAM
Source: Nat Commun. 2019 Apr 23;10:1903. doi: 10.1038/s41467-019-09670-4 (PMC6478907; doi:10.1038/s41467-019-09670-4)
Supplement: Supplementary file 5 — Supplementary Data 2 [file 41467_2019_9670_MOESM5_ESM.zip › Sup_data2/Synthetic/dpt/figures/diffmap_components12.pdf]

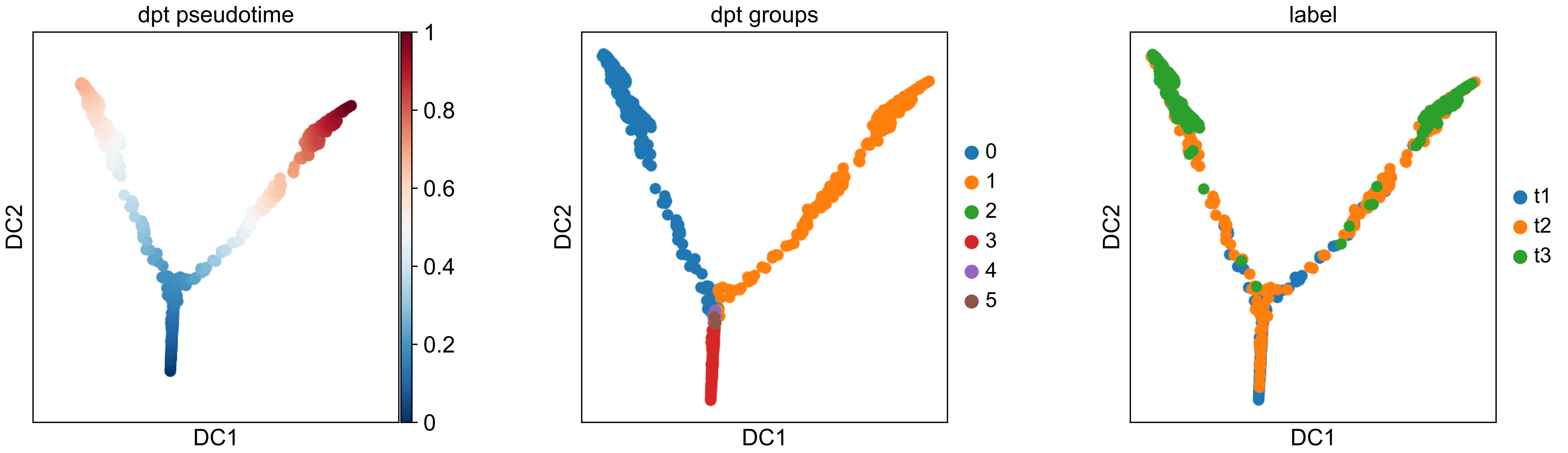
dpt pseudotime
DC2
DC1
1
0.8
0.6
0.4
0.2
0
dpt groups
DC2
DC1
0
1
2
3
4
5
label
DC2
DC1
t1
t2
t3
