# Supplementary material for: Single-cell trajectories reconstruction, exploration and mapping of omics data with STREAM
Source: Nat Commun. 2019 Apr 23;10:1903. doi: 10.1038/s41467-019-09670-4 (PMC6478907; doi:10.1038/s41467-019-09670-4)
Supplement: Supplementary file 5 — Supplementary Data 2 [file 41467_2019_9670_MOESM5_ESM.zip › Sup_data2/Synthetic/gpfates/gpfates_synthetic_fig2.pdf]

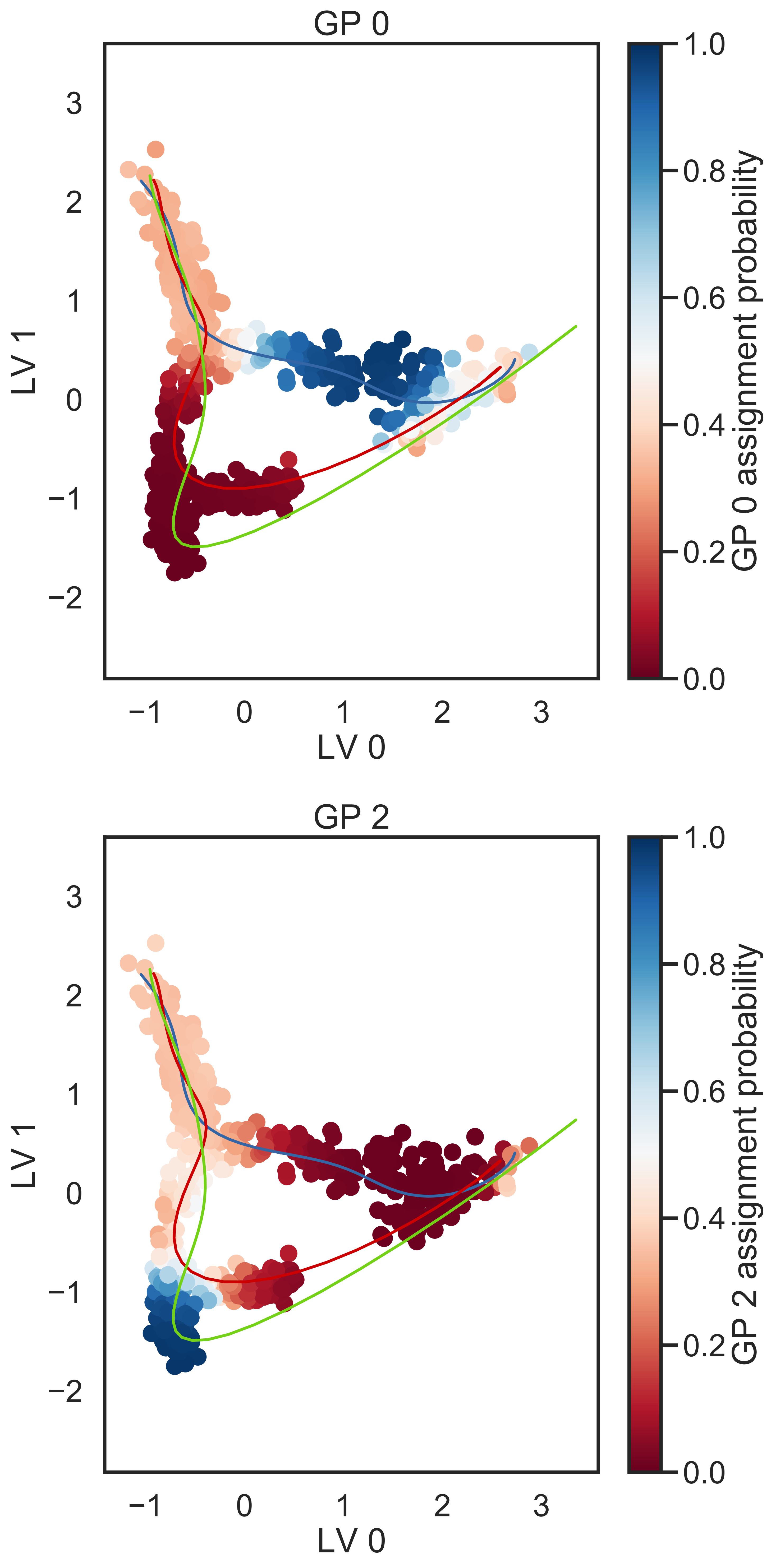
GP 0
GP 1
GP 2
LV 1
LV 0
GP 0 assignment probability
GP 1 assignment probability
GP 2 assignment probability
−2
−1
0
1
2
3
0.0
0.2
0.4
0.6
0.8
1.0
