# Supplementary material for: Single-cell trajectories reconstruction, exploration and mapping of omics data with STREAM
Source: Nat Commun. 2019 Apr 23;10:1903. doi: 10.1038/s41467-019-09670-4 (PMC6478907; doi:10.1038/s41467-019-09670-4)
Supplement: Supplementary file 5 — Supplementary Data 2 [file 41467_2019_9670_MOESM5_ESM.zip › Sup_data2/Trapnell_2014/mpath/Marker_ENSG00000108515.13.pdf]

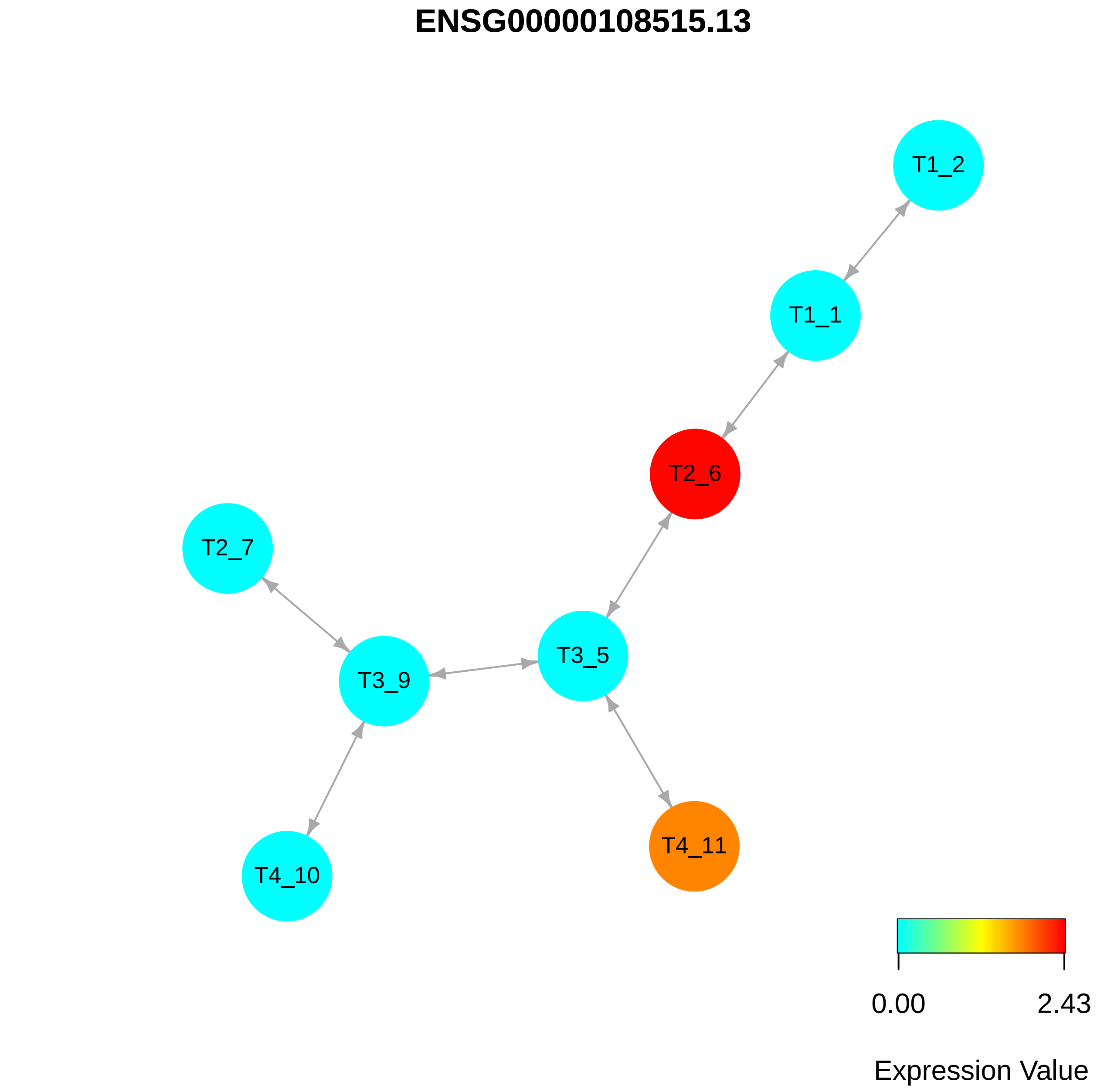
ENSG00000108515.13
T1_2
T1_1
T2_6
T2_7
T3_5
T3_9
T4_10
T4_11
0.00
2.43
Expression Value
