# Supplementary material for: Single-cell trajectories reconstruction, exploration and mapping of omics data with STREAM
Source: Nat Commun. 2019 Apr 23;10:1903. doi: 10.1038/s41467-019-09670-4 (PMC6478907; doi:10.1038/s41467-019-09670-4)
Supplement: Supplementary file 5 — Supplementary Data 2 [file 41467_2019_9670_MOESM5_ESM.zip › Sup_data2/Trapnell_2014/mpath/HSMM_rpkm_DEG_anova_sigGenes_ncluster_vs_nlm.pdf]

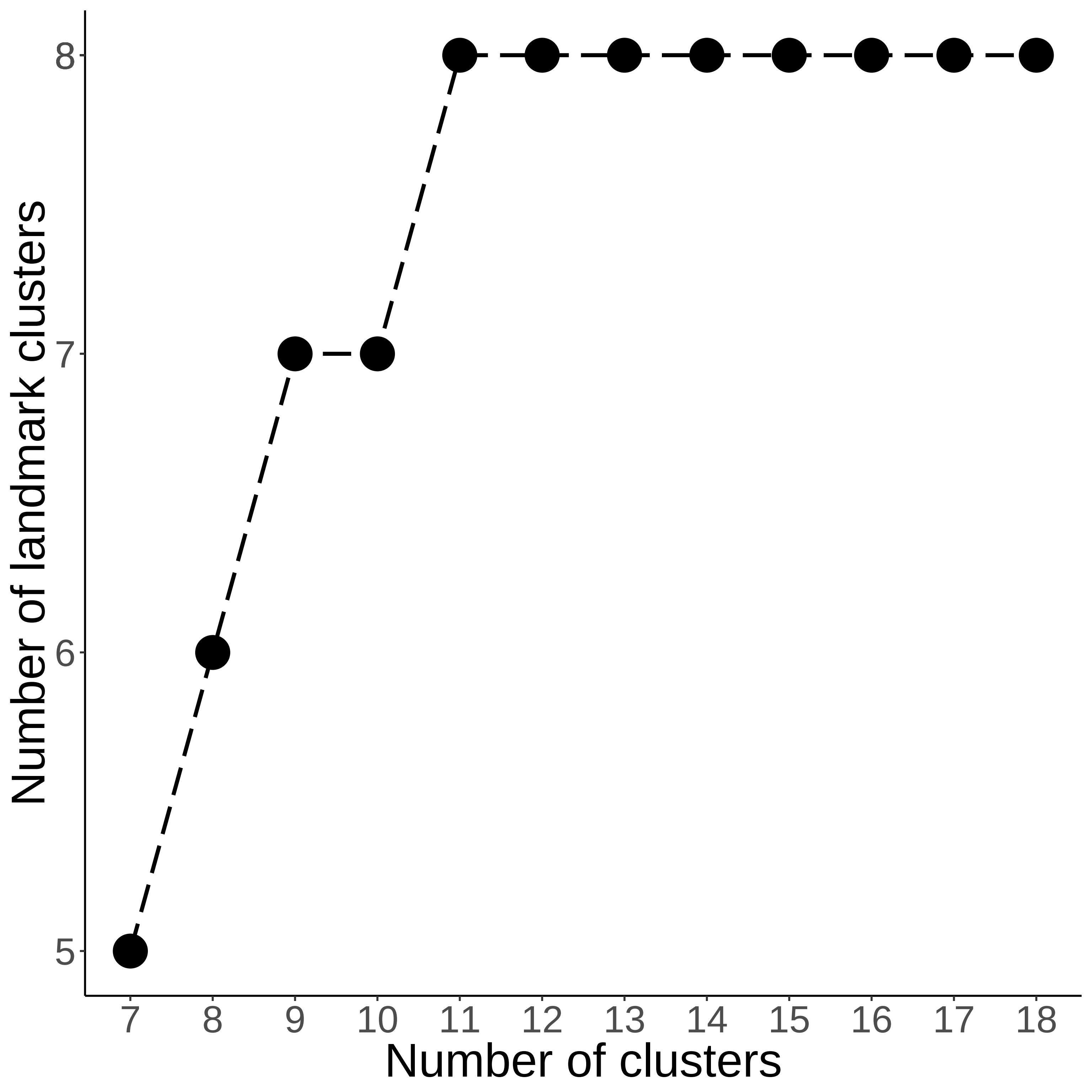

Number of landmark clusters
8
7
6
5
7
8
9
10
11
12
13
14
15
16
17
18
Number of clusters
