# Supplementary material for: Single-cell trajectories reconstruction, exploration and mapping of omics data with STREAM
Source: Nat Commun. 2019 Apr 23;10:1903. doi: 10.1038/s41467-019-09670-4 (PMC6478907; doi:10.1038/s41467-019-09670-4)
Supplement: Supplementary file 5 — Supplementary Data 2 [file 41467_2019_9670_MOESM5_ESM.zip › Sup_data2/Trapnell_2014/mpath/HSMM_rpkm_landmark_cluster.pdf]

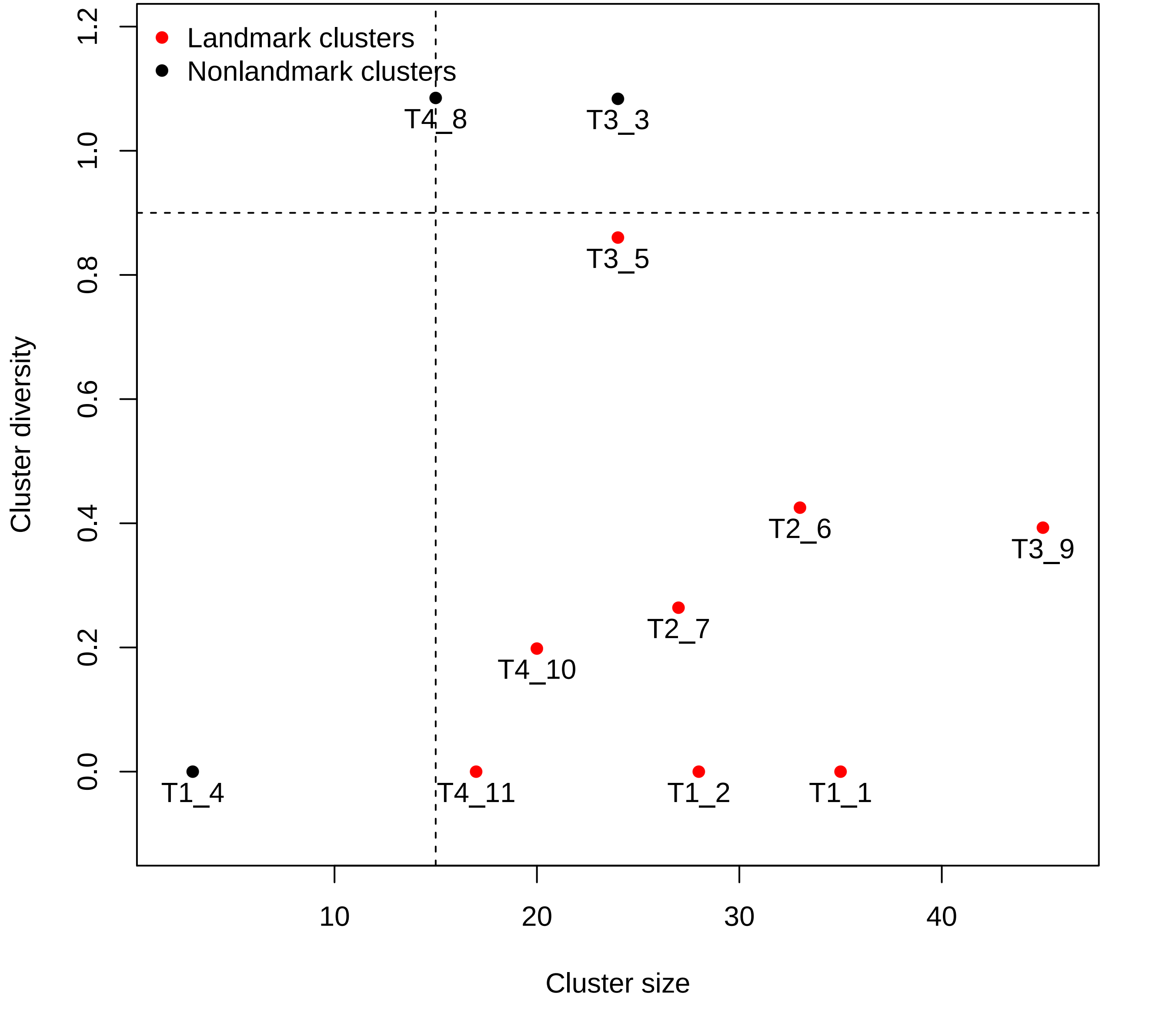
Landmark clusters
Nonlandmark clusters
T4_8
T3_3
T3_5
T2_6
T3_9
T2_7
T4_10
T1_4
T4_11
T1_2
T1_1
Cluster diversity
Cluster size
0.0
0.2
0.4
0.6
0.8
1.0
1.2
10
20
30
40
