# Supplementary material for: Single-cell trajectories reconstruction, exploration and mapping of omics data with STREAM
Source: Nat Commun. 2019 Apr 23;10:1903. doi: 10.1038/s41467-019-09670-4 (PMC6478907; doi:10.1038/s41467-019-09670-4)
Supplement: Supplementary file 5 — Supplementary Data 2 [file 41467_2019_9670_MOESM5_ESM.zip › Sup_data2/Trapnell_2014/monocle2/monocle2_trajectory_by_state.pdf]

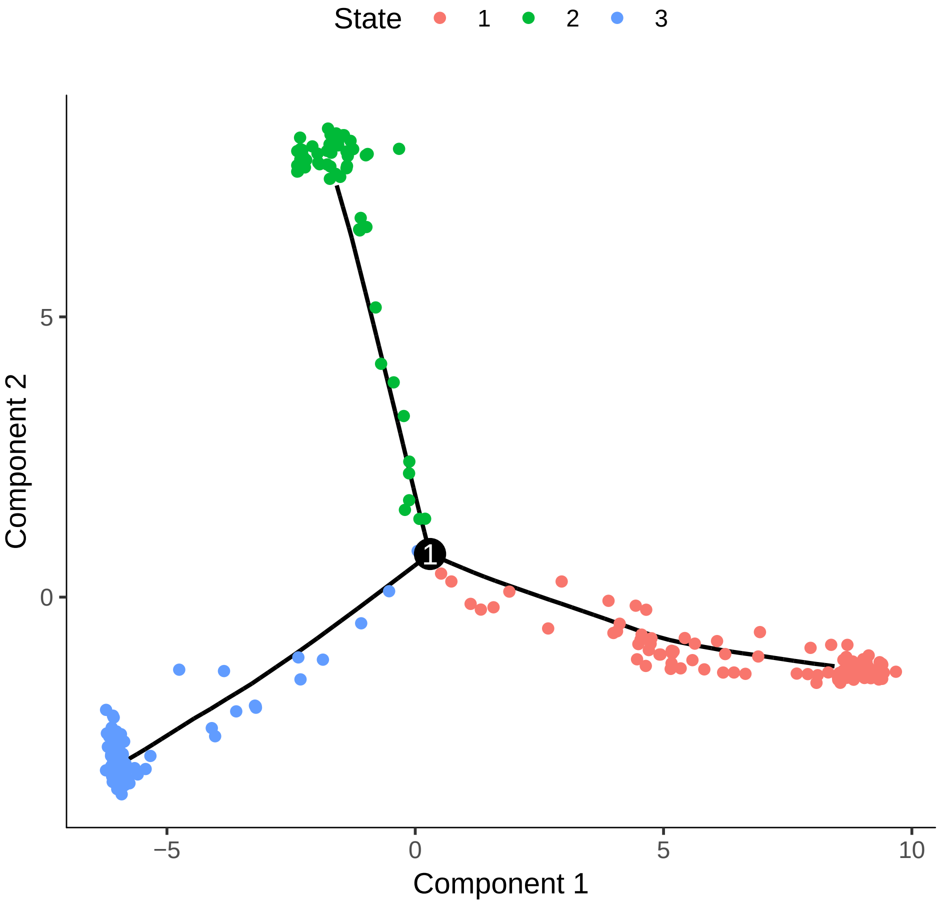

State 1 2 3
Component 2
Component 1
5
0
−5
0
5
10
1
