# Supplementary material for: Single-cell trajectories reconstruction, exploration and mapping of omics data with STREAM
Source: Nat Commun. 2019 Apr 23;10:1903. doi: 10.1038/s41467-019-09670-4 (PMC6478907; doi:10.1038/s41467-019-09670-4)
Supplement: Supplementary file 5 — Supplementary Data 2 [file 41467_2019_9670_MOESM5_ESM.zip › Sup_data2/Trapnell_2014/scuba/Result_run2/Rplots.pdf]

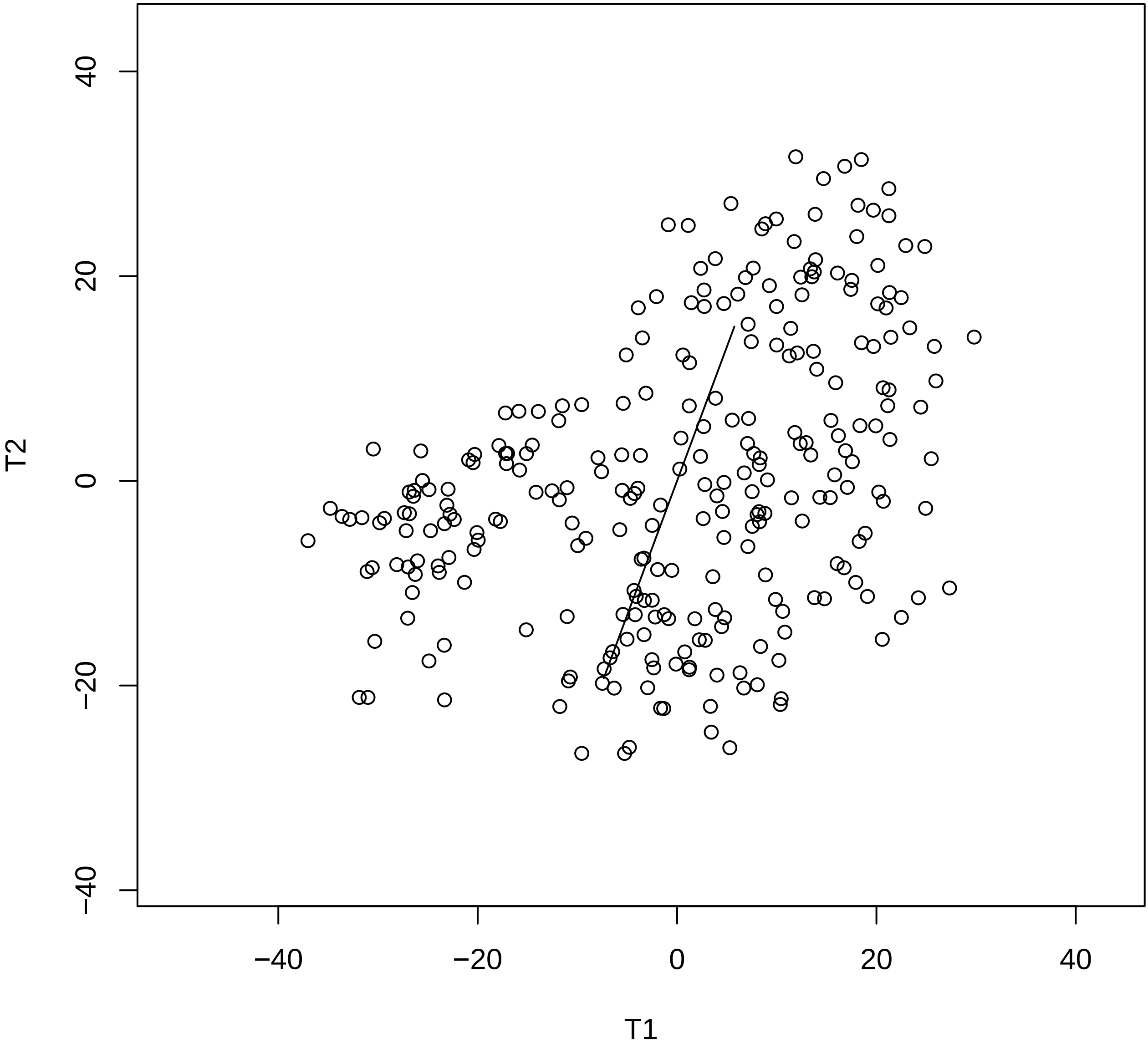

T2
T1
40
20
0
−20
−40
−40
−20
0
20
40

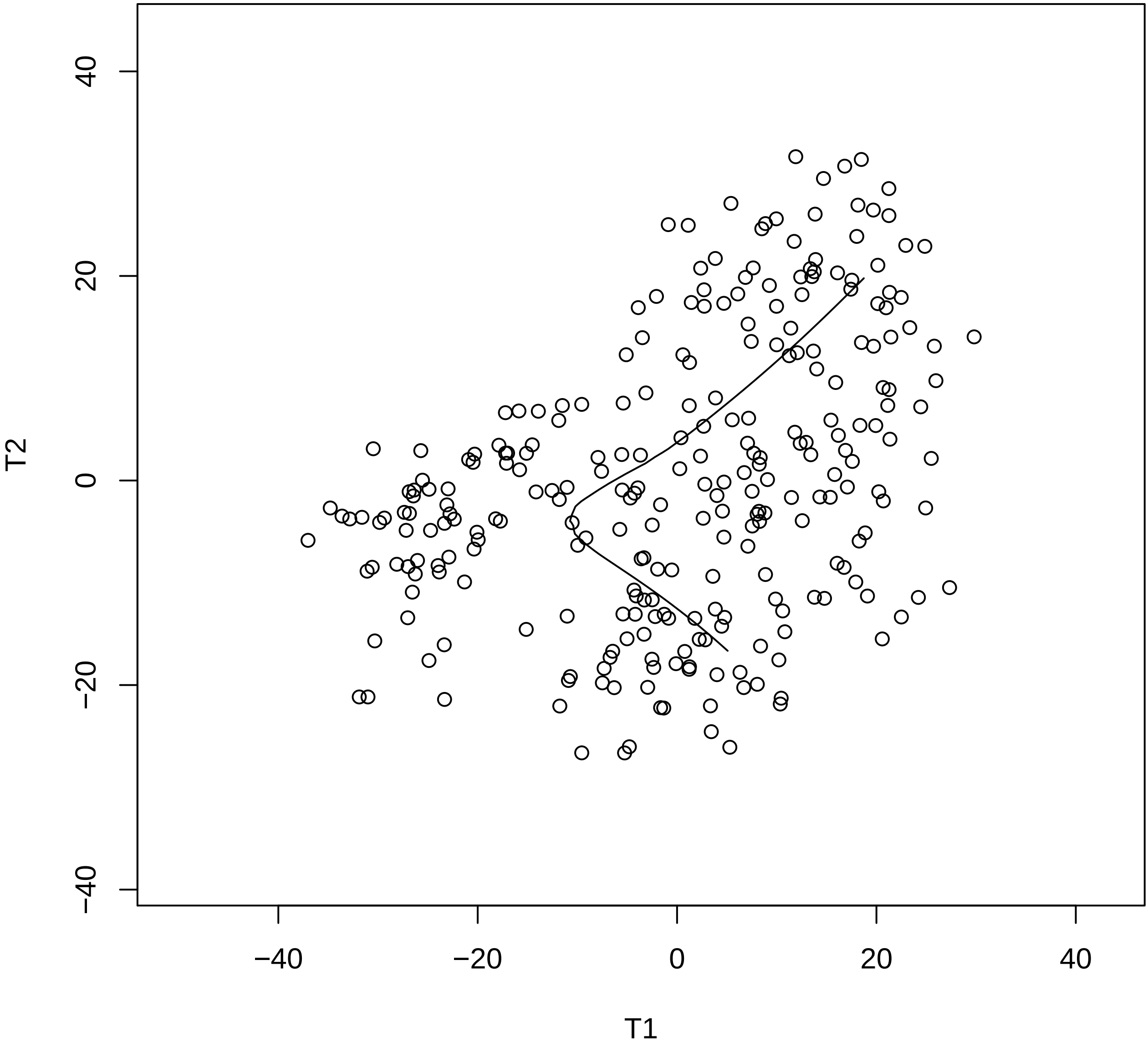

T2
40
20
0
−20
−40
T1
−40
−20
0
20
40

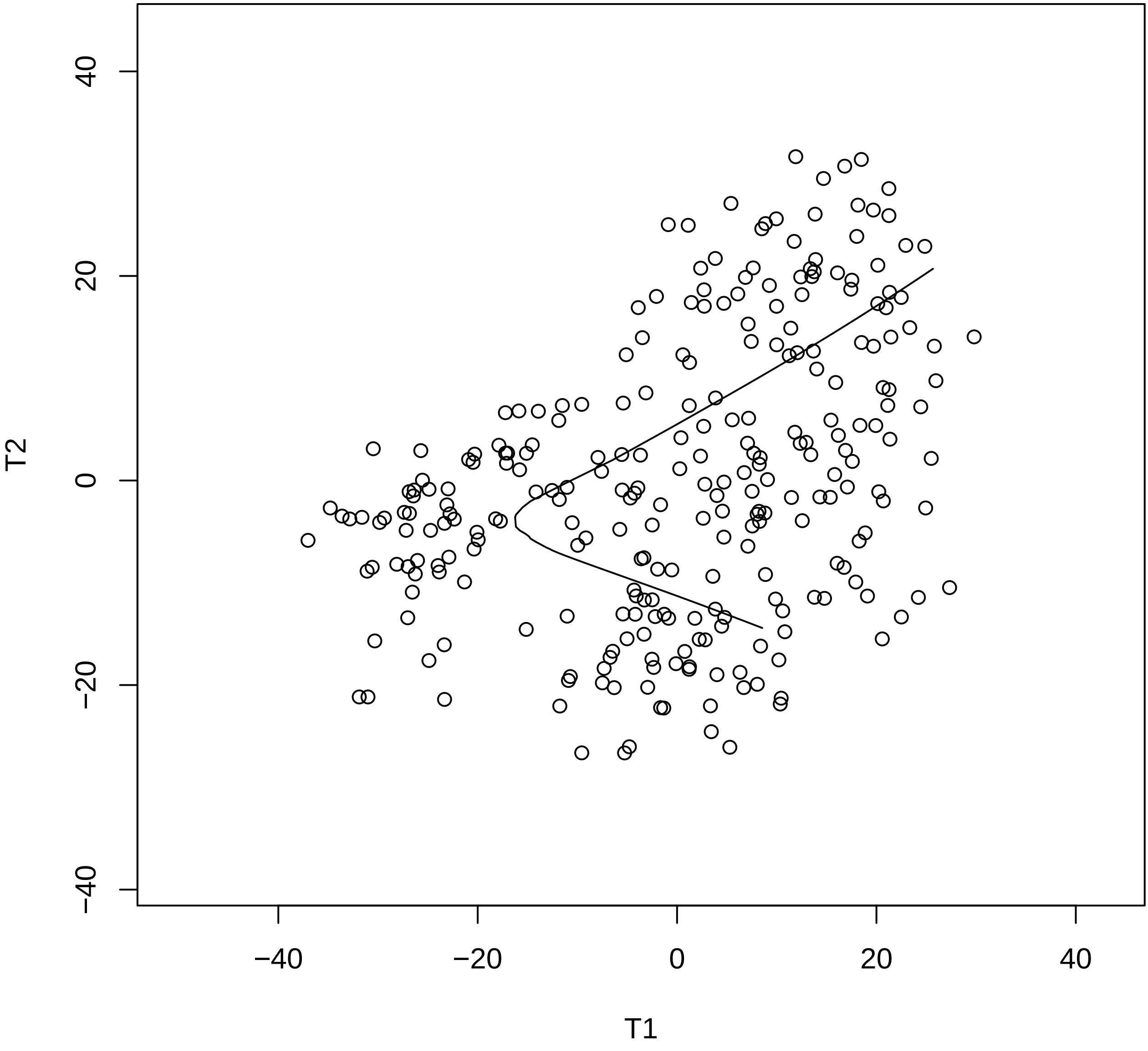

T2
40
20
0
−20
−40
T1
−40
−20
0
20
40

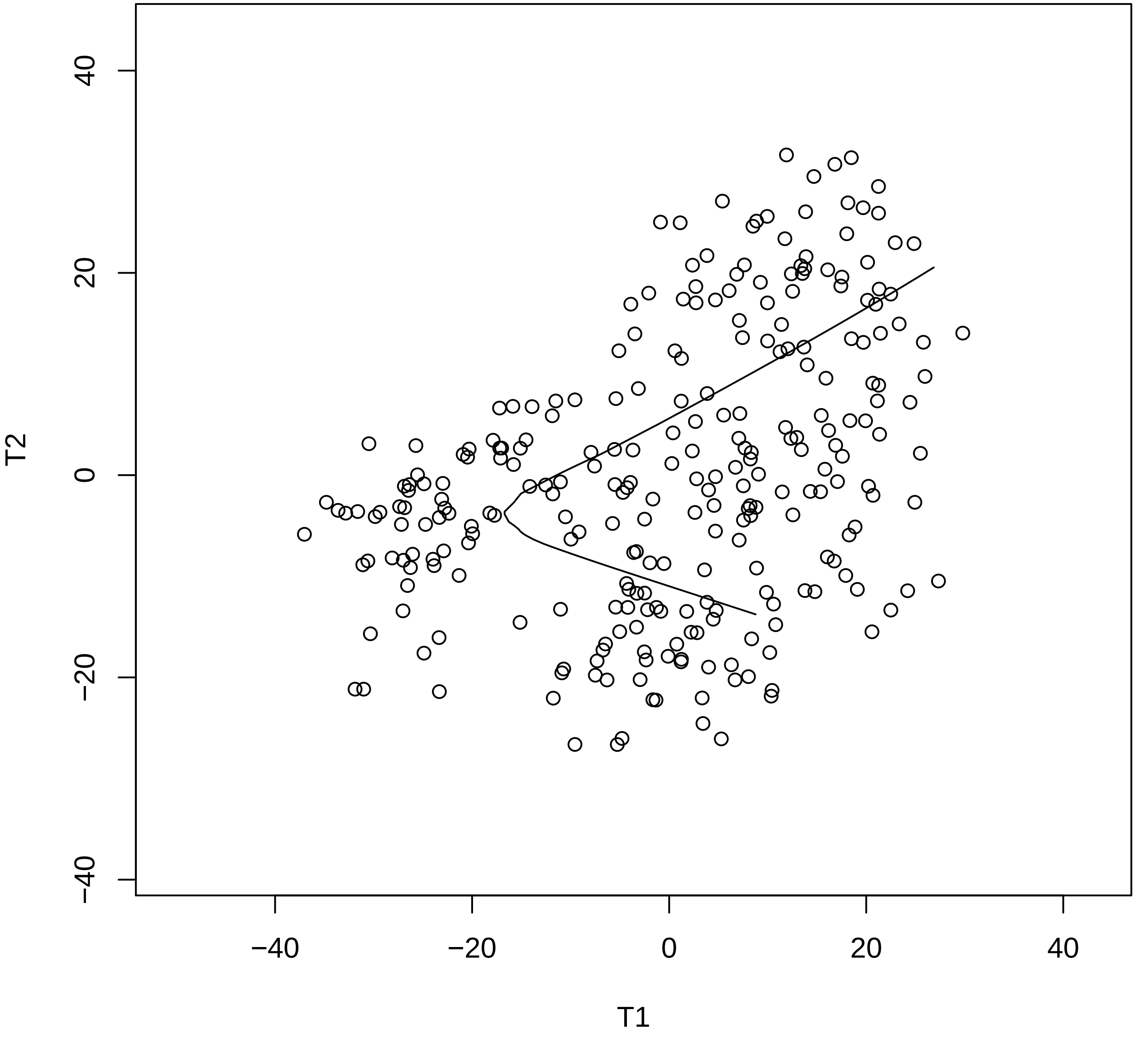

40
20
0
−20
−40
T2
−40
−20
0
20
40
T1

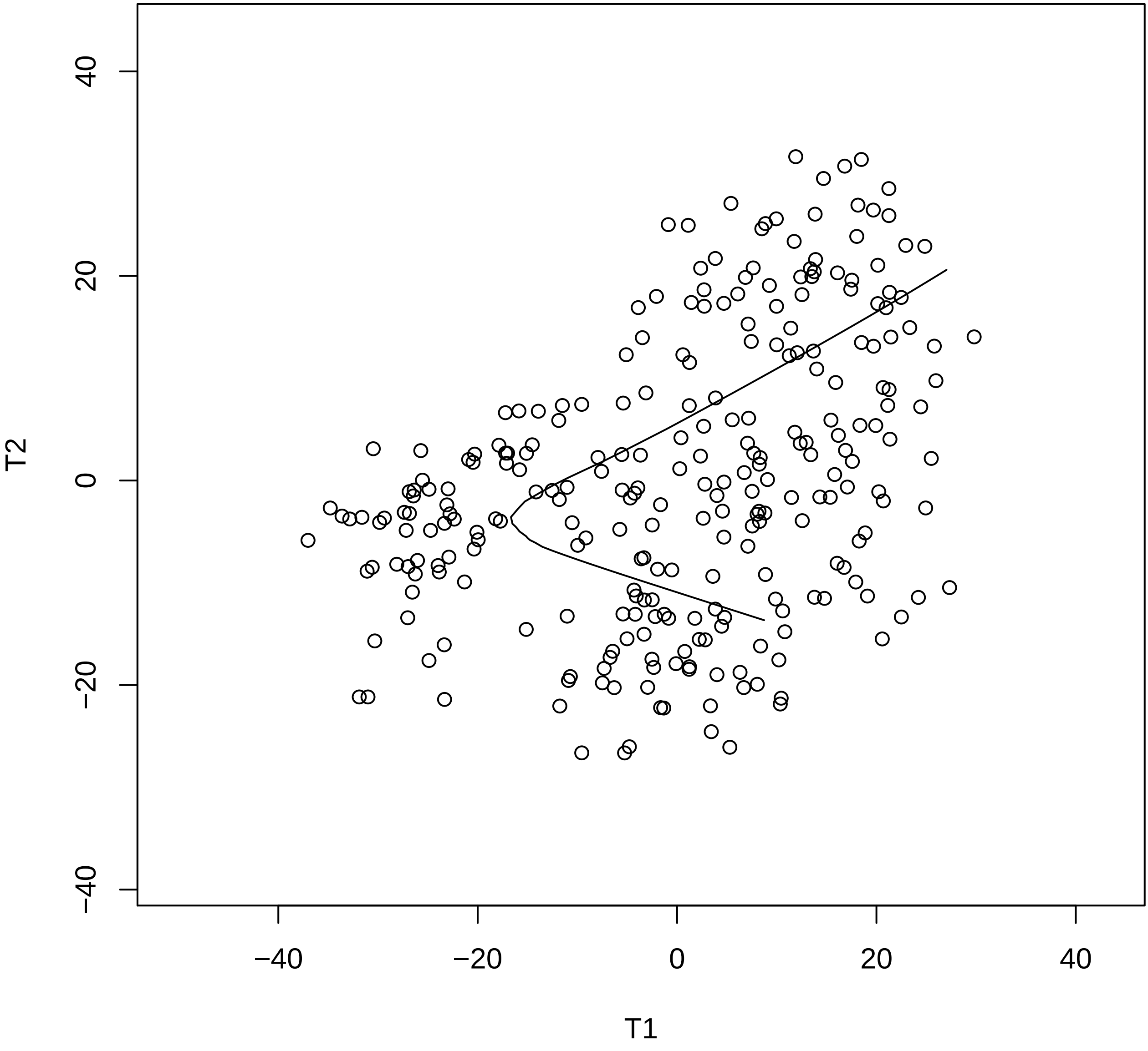

T2
40
20
0
−20
−40
−40
−20
0
20
40
T1

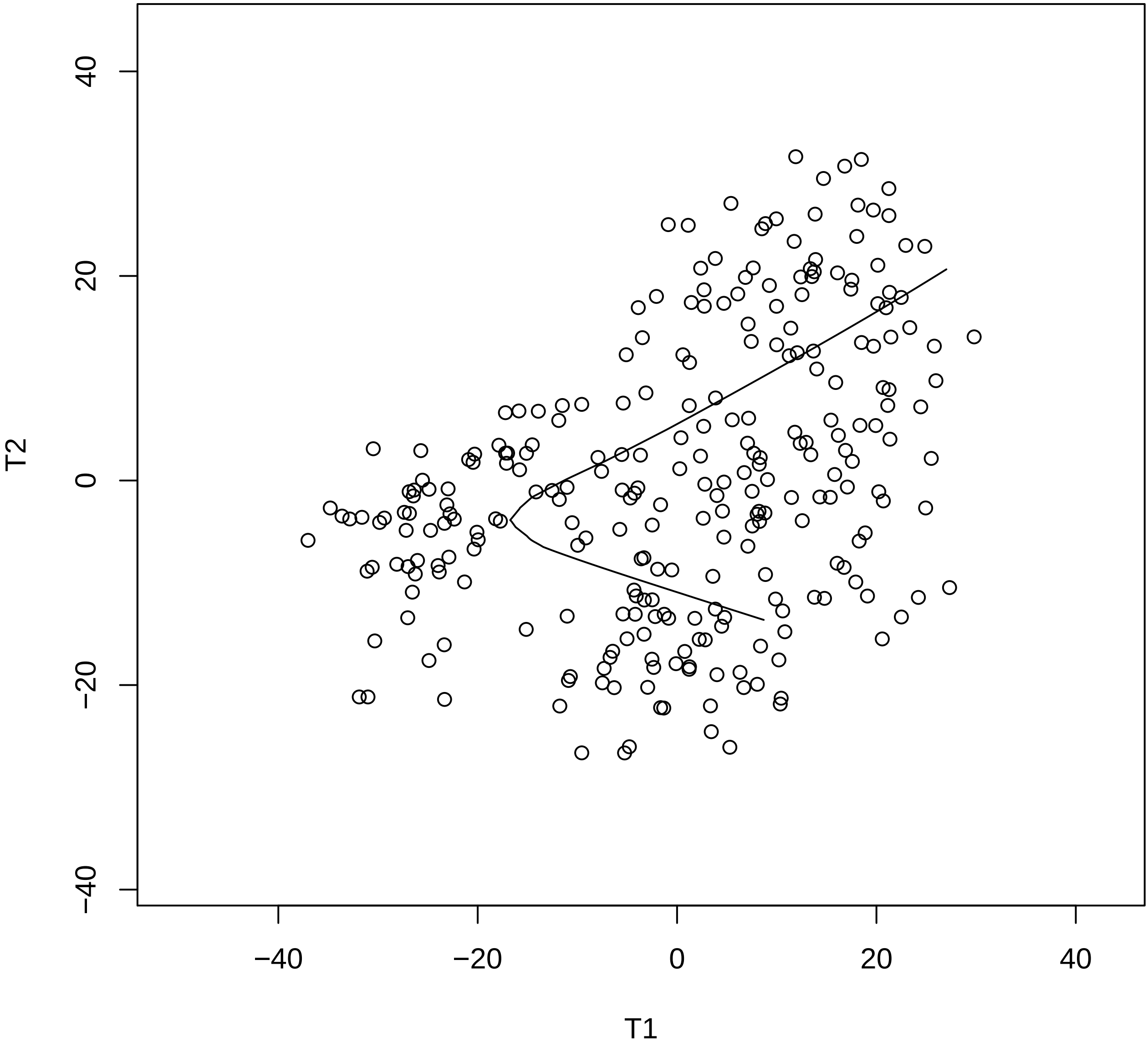
40
20
0
−20
−40
T2
−40
−20
0
20
40
T1
