# Supplementary material for: Single-cell trajectories reconstruction, exploration and mapping of omics data with STREAM
Source: Nat Commun. 2019 Apr 23;10:1903. doi: 10.1038/s41467-019-09670-4 (PMC6478907; doi:10.1038/s41467-019-09670-4)
Supplement: Supplementary file 5 — Supplementary Data 2 [file 41467_2019_9670_MOESM5_ESM.zip › Sup_data2/Trapnell_2014/dpt/figures/diffmap_components12.pdf]

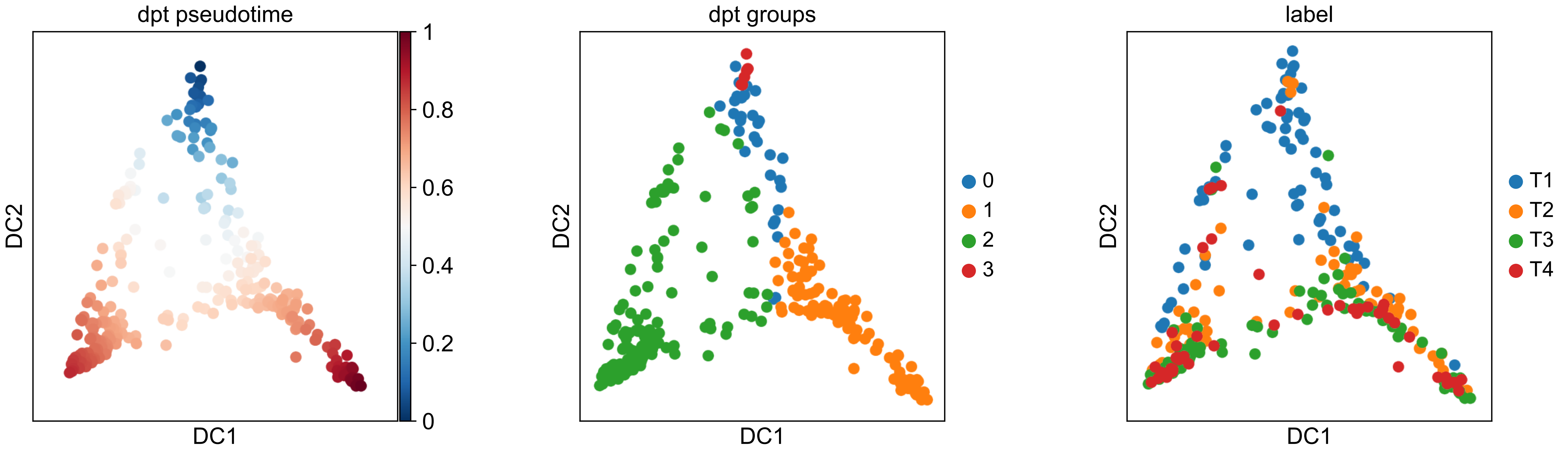

dpt pseudotime
1
0.8
0.6
0.4
0.2
0
DC2
DC1
dpt groups
0
1
2
3
DC2
DC1
label
T1
T2
T3
T4
DC2
DC1
